# Radical‐Dearomative Generation of Cyclohexadienyl Pd(II) toward the 3D Transformation of Nonactivated Phenyl Rings

**DOI:** 10.1002/advs.202307074

**Published:** 2023-12-15

**Authors:** Qi Fan, Kai Jiang, Bo Liu, Huanfeng Jiang, Xiaohui Cao, Biaolin Yin

**Affiliations:** ^1^ Key Laboratory of Functional Molecular Engineering of Guangdong Province School of Chemistry and Chemical Engineering South China University of Technology (SCUT) Guangzhou 510640 China; ^2^ The Second Clinical Medical College and State Key Laboratory of Dampness Syndrome of Chinese Medicine Guangzhou University of Chinese Medicine Guangzhou 510006 China; ^3^ School of Pharmacy Guangdong Pharmaceutical University Guangzhou 510006 China

**Keywords:** carboamination, cyclohexadienyl Pd(II), radical dearomatization, trieneylation

## Abstract

Traditional palladium‐catalyzed dearomatization of (hetero)arenes takes place via an ionic pathway and usually requires elevated temperatures to overcome the energy barrier of the dearomative insertion step. Herein, a combination of the radical and two‐electron pathways is disclosed, which enables room temperature dearomative 3D transformations of nonactivated phenyl rings with Pd(0) as the catalyst. Experimental results together with density functional theory (DFT) calculations indicate a versatile π‐allyl Pd(II) species, cyclohexadienyl Pd(II), possibly is involved in the dearomatization. This species is generated by combining the cyclohexadienyl radical and Pd(I). The cyclohexadienyl Pd(II) provides chemoselective (carboamination and trieneylation), regioselective (1,2‐carboamination), and diastereoselective (carbonyl‐group directed face selectivity) conversions.

## Graphical abstract

Room temperature dearomative 3D transformations of nonactivated phenyl rings are developed possibly involving a versatile π‐allyl Pd(II) species, cyclohexadienyl Pd(II), generated by combining the cyclohexadienyl radical and Pd(I). The cyclohexadienyl Pd(II) provides chemoselective (carboamination and trieneylation), regioselective (1,2‐carboamination), and diastereoselective (carbonyl‐group directed face selectivity) conversions.

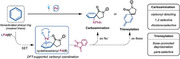

## 1 Introduction

**Scheme 1:**
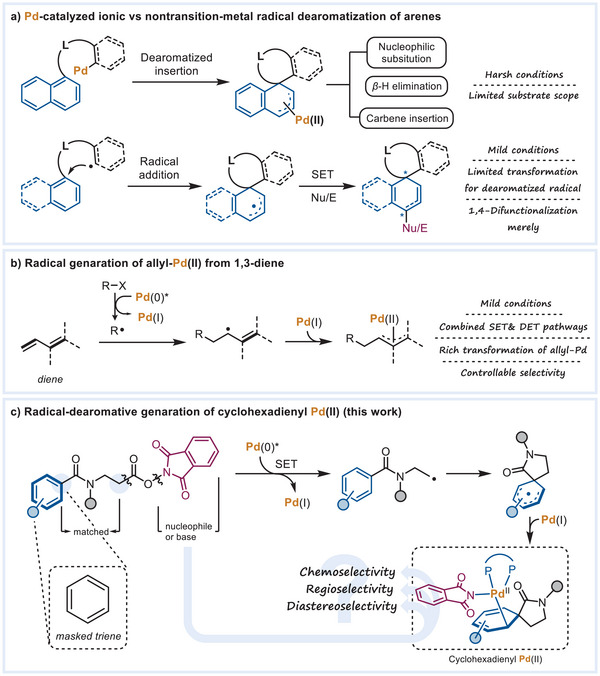
Developments of Pd‐catalyzed dearomatization, radical‐type dearomatization, and emerging excited palladium chemistry.

Dearomatization reactions provide fundamental methods for converting abundant planar aromatics into elaborated 3D molecules.^[^ ^1^, ^2^, ^3^, ^4^, ^5^, ^6^ ^]^ After receiving considerable attention in the synthetic community, Pd(0) and π‐allyl Pd(II) have clearly defined mechanisms.^[^ ^7^, ^8^, ^9^, ^10^, ^11^ ^]^ The current pathways for Pd‐catalyzed dearomatization of (hetero)arenes are mostly two‐electron processes, requiring harsh reaction conditions, such as high temperature, to overcome the energy barrier of the dearomative insertion step. The preference for more active (hetero)arenes and fused aromatic rings,^[^ ^12^, ^13^, ^14^, ^15^, ^16^, ^17^, ^18^, ^19^, ^20^, ^21^, ^22^, ^23^, ^24^, ^25^, ^26^, ^27^, ^28^, ^29^, ^30^, ^31^, ^32^, ^33^, ^34^, ^35^, ^36^, ^37^, ^38^, ^39^, ^40^ ^]^ instead of nonactivated phenyl rings, represents the present state of Pd‐catalyzed dearomatizations (**Scheme** **1**a). Recently, visible light‐induced radical pathways have provided benign methods for the dearomatization of a broader scope of (hetero)arenes including a few examples of nonactivated phenyl rings.^[^ ^41^, ^42^, ^43^, ^44^, ^45^, ^46^, ^47^, ^48^, ^49^, ^50^, ^51^, ^52^, ^53^, ^54^, ^55^, ^56^, ^57^, ^58^, ^59^, ^60^ ^]^ However, these transformations of the dearomatized radical are limited to single‐electron reduction or oxidation, along with radical coupling that produces 1,4‐difunctionalized products. Therefore, enriching the transformation derived from the dearomatized radical to nonactivated phenyl rings is highly desirable. The combination of a dearomatized radical with a palladium catalyst is a potential solution for the diversification of transforming nonactivated phenyl rings. In this case, an important and novel allyl palladium intermediate may be produced, that is, cyclohexadienyl Pd(II). This species is worth exploring, particularly with respect to its further transformations including its chemoselectivity between *β*‐H elimination or nucleophilic substitution, its regioselectivity affording 1,2‐ or 1,4‐difunctionalization, and diastereoselective product formation.

Recent reports on excited‐palladium catalysis have developed novel and powerful reaction patterns beyond ground‐state palladium.^[^ ^61^, ^62^, ^63^, ^64^, ^65^ ^]^ One of these patterns is the radical generation of π‐allyl Pd(II) including the single‐electron transfer (SET) release of Pd(I) and the recombination of the allylic radical with the Pd(I) species.^[^ ^66^, ^67^, ^68^, ^69^, ^70^, ^71^, ^72^, ^73^, ^74^, ^75^, ^76^, ^77^ ^]^ To date, this strategy has been successful with various 1,3‐dienes^[^ ^66^, ^67^, ^68^, ^69^, ^70^, ^71^, ^72^, ^73^, ^75^, ^76^, ^77^ ^]^ as the precursor for the π‐allyl Pd(II) via radical addition (Scheme 1b). However, using a nonactivated benzene ring as the cyclic triene to generate the cyclohexadienyl Pd(II) species, an analog of allyl Pd(II), for the dearomative transformation of the phenyl ring is still not exploited.

To form the cyclohexadienyl Pd(II), we devised redox esters to serve as the precursors of both the cyclohexadienyl radical and Pd(I) (Scheme 1c). The *N*‐hydroxyphthalimide (NHP) ester of *β*‐amino acid produces a nucleophilic alkyl radical whose polarity allows it to react with the terminal benzamide moiety. The released phthalimide anion acts as both a nucleophile and base to manipulate the conversion of the cyclohexadienyl Pd(II) intermediate. This method guarantees a pure reaction system and moderate conditions for exploring the cyclohexadienyl Pd(II) species.

## 2 Results and Discussion

### 2.1 Reaction Insights

**Scheme 2:**
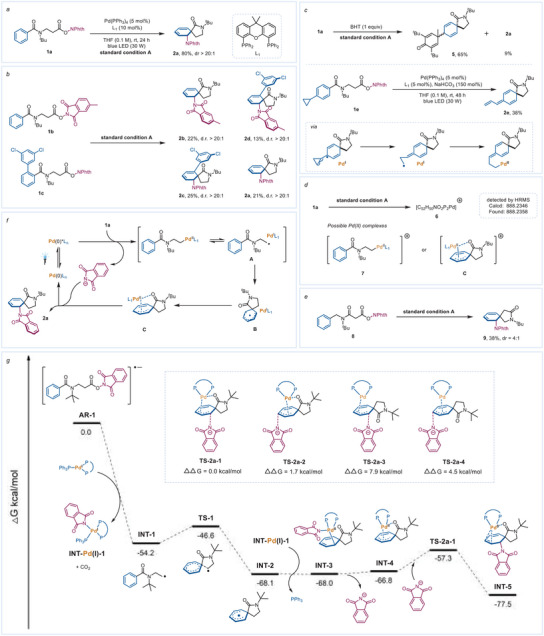
a) Reaction of 1a to 2a under standard conditions A: 0.2 mmol 1a, 5 mmol% Pd(PPh_3_)_4_, 10 mmol% Xantphos (L_1_), THF (0.1 m of 1a), 30 W blue LEDs irradiation, room temperature, and argon atmosphere. b) Crossover experiment. c) Radical trapping experiments. d) HRMS of reaction mixture. e) Reaction of control compound 8. f) Proposed mechanism. g) Computed energy profile of the proposed mechanism. Free energies in solution (in kcal mol^−1^) at the M06‐D3(SMD)/SDD/6‐311+G(d, p) //B3LYP‐D3/LanL2DZ/6‐31G(d) level are displayed.

Compound 1a was used as the model substrate to explore the dearomative reaction. The dearomative carboamination of 1a occurred as expected, and the 3D product 2a was afforded with 80% isolated yield and >20:1 diastereomeric ratio under the optimized reaction conditions: 5 mmol% Pd(PPh_3_)_4_, 10 mmol% Xantphos(L_1_), THF as the solvent (0.1 m of 1a), and 30 W blue LEDs irradiation at room temperature (**Scheme** **2**a, see more optimization details in the Supporting Information). A crossover experiment was conducted, and four products were obtained, demonstrating that the phthalimide anion might dissociate after the SET process (Scheme 2b). To ascertain the reaction mechanism, we carried out radical trapping assays under suitable conditions (Scheme 2c). First, the reaction was carried out with the addition of 1 equiv. 2,6‐di‐tert‐butyl‐4‐methylphenol, and the cyclohexadienyl radical was captured as 5 in 65% isolated yield. When the *para* cyclopropyl substrate 1e was used in the reaction, the ring‐opened triene product 2e was isolated in a 32% yield. The above assays confirm that the excited‐Pd‐catalyzed dearomatization of the benzene ring goes through a radical pathway involving the cyclohexadienyl radical. High resolution mass spectrometry (HRMS) results reflect the production of the cationic Pd(II) complex (7 or C) in the reaction (Scheme 2d). We then prepared the control substrate 8 which has the carbonyl group with a single‐atom bridge to the phenyl ring versus the direct attachment in 1a (Scheme 2e). Under the standard conditions, as expected, the reaction of 8 delivered the dearomative 1,2‐carboanimation product 9 but with a decreased dr value (4:1). This indicates that the carbonyl group directly connected to the phenyl ring of 2a may contribute to the diastereoselectivity of carboamination via a face‐directing effect. This directing effect on π‐allyl palladium generated from Pd(I) has not been documented, that we were aware of, before this work. According to the literature^[^ ^67^, ^68^, ^80^, ^81^, ^82^, ^83^ ^]^ and the above experimental results, a possible reaction cycle is proposed (Scheme 2f). The NHP ester 1a is reduced via a SET process by the excited Pd(0)* catalyst with the expulsion of CO_2_ and phthalimide anion to afford the hybrid alkyl Pd(I) radical species A. The resulting nucleophilic *β*‐amide radical undergoes *ipso* cyclization to generate a hybrid cyclohexadienyl Pd(I) radical species B. After the combination of Pd(I) with the cyclohexadienyl radical, carbonyl‐coordinated vinyl π‐allyl Pd(II) C is formed, which is then attacked by the phthalimide anion to deliver product 2a and regenerate the Pd(0) catalyst.

We further analyzed the reaction pathway by density functional theory (DFT) calculations (Scheme 2g). First, 1a undergoes SET with the excited photocatalyst PC‐S1 to produce anionic radical AR‐1 and Pd(I) species PC‐cation (Δ*E* = −21.5 kcal mol^−1^) (see Figure S7, Supporting Information). The unstable AR‐1 then decomposes into INT‐1, CO_2_, and phthalimide anion. The phthalimide anion is captured by PC‐cation to generate INT‐Pd(I)‐1. The calculation results demonstrate that the conversion of AR‐1 to INT‐1 is a very thermodynamically favored process (Δ*G* = −54.2 kcal mol^−1^). The generated INT‐1 then converts to a more stable cyclohexadienyl radical INT‐2 via a rapid *ispo*‐cyclization process and transition state TS‐1 (only 7.6 kcal mol^−1^ energy barrier, INT‐1→INT‐2: Δ*G* = −13.9 kcal mol^−1^). After ligand exchange between INT‐Pd(I)‐1 and INT‐2, the cyclohexadienyl Pd(II) intermediate INT‐3 is generated. The relatively crowded environment around Pd in INT‐3 (see Figure S7, Supporting Information) and the close distance from Pd to the oxygen atom of the carbonyl (Pd─O distance: 2.79 Å) allows the phthalimide anion on INT‐3 to facilely dissociate forming the cationic Pd(II) intermediate INT‐4 (INT‐3→ INT‐4: Δ*G* = 1.2 kcal mol^−1^). In INT‐4, only one of the dearomatized double bonds is coordinated with Pd. This newly formed Pd complex is a vinyl π‐allyl Pd(II) species (the total cyclohexadienyl is not coplanar). Subsequently, the dissociative phthalimide anion attacks the *ortho*‐carbon atom of INT‐4 as the nucleophile via transition state TS‐2a (the energy barrier is 10.7 kcal mol^−1^) to produce INT‐5. Finally, INT‐5 undergoes ligand exchange with PPh_3_ to release product 2a and regenerate the Pd(0).

Next, we delved deeper into the transition state of the NPhth anion nucleophilic attack (INT‐4) to explain the specific reasons for the regioselectivity and stereoselectivity in the reaction. As shown in Scheme 2g, when the NPhth anion attacks from the ipsilateral side of the carbonyl, Pd is on the opposite side of the carbonyl group. Due to the lack of coordination between Pd(I) and O, the energy of transition states TS‐2a‐3 and TS‐2a‐4 are significantly higher than that of TS‐2a‐1 (ΔΔ*G*: 7.9, 4.5 vs 0.0 kcal mol^−1^), which is consistent with the high dr value observed in the experiment. Further calculations show that when the NPhth anion attacks the *ortho* carbon atom of INT‐4, Pd coordinates with C3, C4 in TS‐2a‐1, and when attacking the para carbon atom, Pd coordinates with C2, C3 in TS‐2a‐2. The Pd─O distance is significantly shorter in TS‐2a‐1 than in TS‐2a‐2 (2.73 vs 2.98 Å). The C─Pd─O bond angle in TS‐2a‐1 and TS‐2a‐2 is 89.9° and 70.5°, respectively, and the distortion of the PdL_4_ tetrahedron in TS‐2a‐2 is significantly higher (see Figure S7, Supporting Information). This implies TS‐2a‐1 is a more favorable transition state and accounts for the 1,2‐regioselectivity.

### 2.2 Carboamination

**Scheme 3:**
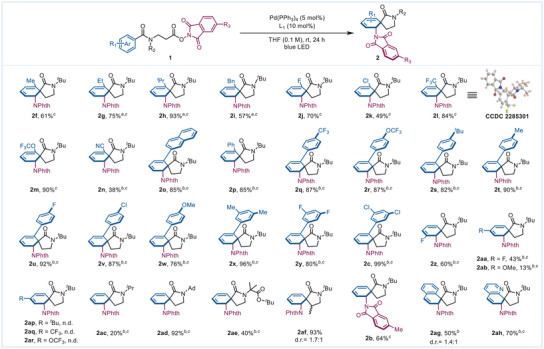
The substrate scope of the dearomative 1,2‐carboamination. *^a^* reaction time: 36 h. *^b^* reaction time: 48 h. *^c^* dr >20:1.

Following the above calculations that unveiled how the coordination mode of cyclohexadienyl Pd(II) and vinyl π‐allyl Pd(II) relates to the diastereoselective 1,2‐carboamination, we investigated the substrate scope of this highly diastereoselective 1,2‐carboamination (**Scheme** **3**). Substrates bearing different electron‐withdrawing and electron‐donating *ortho*‐substituents gave the corresponding products 2f‐2n in moderate to excellent yields (38–93%). Electron‐rich and electron‐deficient aryl groups at the *ortho* position were also well tolerated (2o‐2y and 2e, yielding 76–99%). The *meta‐3‐*fluoro substrate produced 1,2‐carboamination product 2z in 60% yield, while the *para*‐4‐fluoro substrate produced a lower yield (2aa, 43%). The 4‐MeO‐substituted substrate affords a poor yield of the 1,2‐carboamination product (2ab, 13%), which may be ascribed to the polarity of the phenyl ring being unsuitable for reaction with the *β*‐amino radical. Other para substrates (1ap‐1ar) were also conducted under the standard conditions, however, no desired carboamination products were generated. The influence of branched substituents on the *N* atom of the amide was also evaluated. The smaller group (isopropyl, 2ac) provided diminished yield (20%), while the larger two (1‐adamantyl, *tert*‐butoxycarbonyl dimethyl) provided higher yields (92% for 2ad, 40% for 2ae). Moreover, the substrate as the precursor of a secondary alkyl radical also went through the reaction smoothly, giving the desired product 2af (93% yield, dr = 1.7:1, the low dr value for 2af refers to the spiro carbon and the carbon atom attached to methyl groups, not refers to spiro carbon and the carbon atom attached to NPhth groups). Methyl on the phthalimide moiety was tolerated (2b, 64% yield). However, the substrate 1ao carrying a more carbon chain did not afford *ipso*‐cyclization dearomative product but only led to an *ortho*‐cyclization product (see Supporting Information). In addition, 1,2‐carboamination also occurred in other aromatic substrates with good yields such as naphthalene and quinoline (2ag and 2ah, 50% and 70% respectively). All the above phenyl samples exhibited excellent diastereoselective 1,2‐carboamination (dr > 20:1), demonstrating the good groups‐tolerance of the carbonyl‐directing effect of the cyclohexadienyl Pd(II) species.

**Scheme 4:**
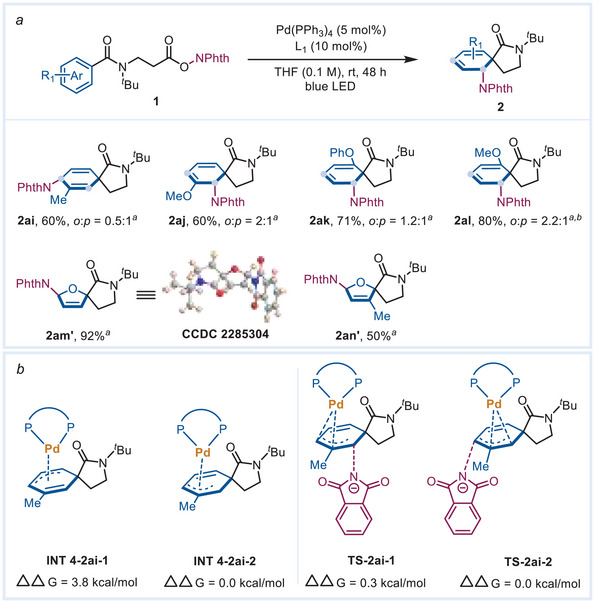
a) Substrates scope; Mixture were provided for some of the products; *^a^* dr >20:1; *^b^* reaction time: 24 h; b) Calculated relative energy of the intermediates for 2ai and calculated relative energy of the transition states for 2ai.

During the screen of other different substrates (**Scheme** **4**a), we found that several *m*‐ and *o*‐substituents gave the 1,4‐carboamination product including the *m*‐methyl, *m*‐methoxyl, *o*‐phenoxyl, and *o*‐methyl. For the sample that provided the 1,4‐carboaminaton product 2ai′, we conducted DFT calculations to help elucidate the regioselectivity from the simulated structure of the cyclohexadienyl Pd(II) (Scheme 4b). When a methyl is at the *meta* position, Pd tends to bind with C3 (INT 4‐2ai‐2) instead of C5 (INT 4‐2ai‐1), which may be due to the more favored interaction between the electrophilic cationic Pd(II) and electron‐donating methyl group. Meanwhile, due to the increased steric hindrance from the methyl group, the *para*‐nucleophilic attack of phthalimide anion (TS‐2ai‐2) is slightly more favorable than *ortho*‐position (TS‐2ai‐1), generating the 1,4‐carboamination product as the major one (see Figure S7, Supporting Information). In addition, substrates bearing *ortho* methoxy or phenoxy also produced mixtures of products with excellent yields (2ak and 2al). We rationalize that the lone pair electrons from the methoxyl or phenoxyl groups may participate in the coordination of the cyclohexadienyl Pd(II), with the carbonyl on the lactam ring, which deteriorates the regioselectivity. Interestingly, the substrates with a furan core also smoothly proceed through carboamination under the standard conditions, but affords inverse diastereoselectivity and regioselectivity in the products (2am′ and 2an′) compared to the cyclohexadienyl Pd(II). The involvement of furonium intermediate instead of π‐allyl Pd species may account for the formation of anti‐addition products 2am′ and 2an′

### 2.3 Trieneylation

**Scheme 5:**
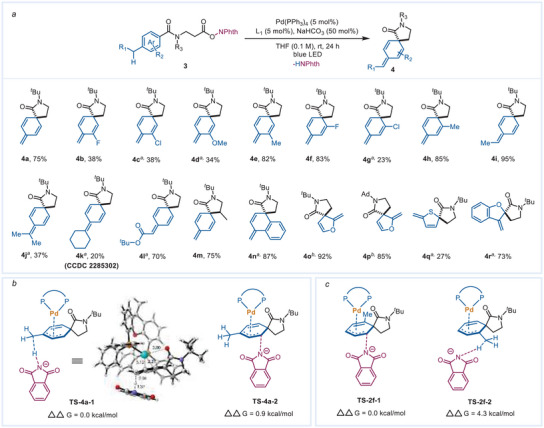
a) Scope of the trieneylation. *^a^* reaction time: 48 h. *^b^* 10 mol% L_1_ was added and the reaction time was 40 h; b) Calculated energy level of the transition states for *β*‐H elimination and nucleophilic substitution of 4a. c) Calculated energy level of the transition states for *β*‐H elimination and nucleophilic substitution of 2f.

When the *para*‐position was substituted by methyl (3a), the reaction carried out under standard conditions did not give any carboamination product. Instead, a dearomative conjugated triene product 4a was isolated with a 55% yield. This specific chemoselectivity for the trieneylation of *para*‐ methyl substrate 3a presumably is due to the role of the dissociative phthalimide anion changing from a nucleophile to a base. Therefore, after inhibiting the trace amount of H^+^ in the reaction system by adding a weak base, we achieved a better yield for the trieneylation (see more details for the optimization in the Supporting Information). We next investigated the substrate scope of the trieneylation (**Scheme** **5**a). For the *para*‐methyl substrates, those with *meta*‐F, *meta*‐Cl, *ortho*‐Cl, and *meta*‐OMe afforded acceptable yields (4b‐4d, 4g, 23–38%), while those with *meta*‐Me, *ortho*‐F, *ortho*‐Cl, and *ortho*‐Me gave satisfactory yields (4e, 4f, 4h, 82–85%). The *para*‐ethyl substrate provided an excellent yield (4i, 95%), but the larger *i*‐Pr and cyclohexyl obviously decreased the yield (4j, 37%; 4k, 20%). A terminal ester group and secondary alkyl radical were also well tolerated (4l, 70%; 4m, 75%). Other (hetero)aromatic substrates (naphthene, furan, thiophene, and benzofuran) also deliver the remote‐desaturation products in acceptable to excellent yields (4n‐4r, 27–92%).

To better understand the reasons 3a has good selectivity toward trieneylation, the energy level of the transition states for nucleophilic substitution and β‐H elimination were calculated. The process of extracting the β‐H by the dissociated phthalimide anion is more kinetically advantageous than its nucleophilic attack (TS‐4a‐1 vs TS‐4a‐2, Scheme 4b). The traditional Pd─H elimination pathway shows a higher energy barrier (TS‐4a‐4: 9.4 kcal mol^−1^, see Table S7, Supporting Information), which supports that the direct deprotonation by the phthalimide anion is the predominant path. However, the energy of the nucleophile attack is much lower than that of β‐H elimination for the o‐methyl substrate 1f, which is consistent with the above experimental results (TS‐2f‐2 vs TS‐2f‐1, Scheme 4b). This may be attributed to the steric hindrance of the o‐methyl toward the cyclohexadienyl Pd(II) in the β‐H elimination process. Therefore, 1f displays inverse chemoselectivity relative to 3a. In brief, the specific chemoselectivity of cyclohexadienyl Pd(II) to trieneylation can be achieved via fine‐tuning the structure of the substrate (installing benzyl C─H on the 4‐position of the benzene ring).

### 2.4 Derivatizations and Applications

**Scheme 6:**
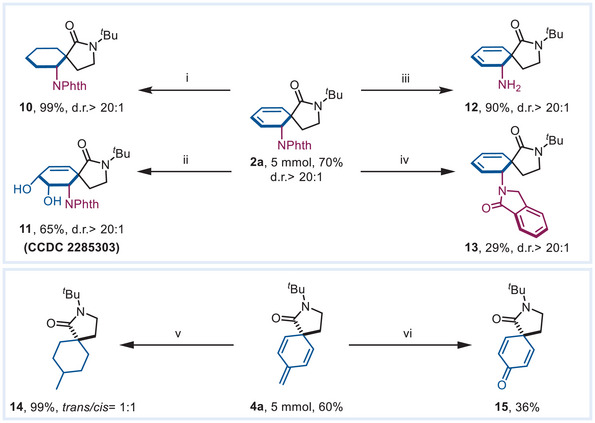
Gram‐scale synthesis and product derivatization. Conditions: i) Pd/C, H_2_ in balloon, 1 atm; ii) NMO/K_2_OsO_4_·2H_2_O; iii) N_2_H_4_·H_2_O; iv) Ph_2_SiH_2_; vi) O_3_ atmosphere. v) Pd/C, H_2_ in balloon, 1 atm.

The cyclohexadiene skeleton with 1,3 diene structure widely exists in valuable molecules, such as natural products and bioactive compounds, and its construction has been intelligently reported by the transition‐metal mediated dearomatized difunctionalization of nonactivated phenyl rings.^[^ ^78^, ^79^ ^]^ Our method can also afford such skeleton (2a), as well as its gram‐scale synthesis, with good yield (1.23 g, 70%). The dearomatized triene 4a can also be scale‐up produced with satisfactory yields (0.65 g, 60%), demonstrating the scalability of these reactions. Subsequently, we completed several transformations of these spiro products (**Scheme** **6**). In the presence of Pd/C and hydrogen, the conjugated alkene moieties of 2a and 4a were fully hydrogenated to afford products 10 and 14 with excellent yields (99%), albeit 14 is obtained as the mixture of *trans/cis* isomers (*trans*/*cis* = 1:1). Dihydroxylation of the cyclohexadiene moiety in 2a proceeded in the presence of NMO and K_2_OsO_4_·2H_2_O, giving the product 11 in 65% yield. In addition, allylamine 12 can be obtained with a 90% yield through the deprotection of phthalimide in N_2_H_4_·H_2_O. Reduction of a carbonyl group on the phthalimide of 2a can be achieved by using Ph_2_SiH_2_, giving the corresponding product 13 in 29% yield. Moreover, oxidative cleavage of the terminal olefin in the conjugated triene product 4a proceeding under an O_3_ atmosphere produces the cyclohexadienone product 15 in 36% yield. The above transformations of the products denote the facile decoration and diversification of the 3D skeleton generated using this Pd‐catalyzed mild dearomatization.

## 3 Conclusion

In summary, the nonactivated phenyl ring as a masked cyclic triene with an excited Pd catalyst at room temperature undergoes radical‐dearomatization leading to selective difunctionalization or trieneylation. These transformations involve the generation of cyclohexadienyl Pd(II) via the combination of the cyclohexadienyl radical with Pd(I). The carbonyl group directs the coordination of the Pd(I), which is responsible for diastereoselectivity and regioselectivity. Moreover, the unprecedented dual role of the phthalimide anion allows for chemically selective trieneylation. This radical transition‐metal‐catalyzed dearomatization will greatly enrich the transformation of nonactivated phenyl rings under mild conditions.

## Conflict of Interest

The authors declare no conflict of interest.

## Supporting information

Supporting Information

## Data Availability

The data that support the findings of this study are available in the supplementary material of this article.
