# Supplementary material for: Radical‐Dearomative Generation of Cyclohexadienyl Pd(II) toward the 3D Transformation of Nonactivated Phenyl Rings
Source: Adv Sci (Weinh). 2023 Dec 15;11(9):2307074. doi: 10.1002/advs.202307074 (PMC10916580; doi:10.1002/advs.202307074)

ADVANCED SCIENCE
Open Access

Supporting Information

for *Adv. Sci.*, DOI 10.1002/advs.202307074

Radical-Dearomative Generation of Cyclohexadienyl Pd(II) toward the 3D Transformation of Nonactivated Phenyl Rings

Qi Fan, Kai Jiang, Bo Liu, Huanfeng Jiang, Xiaohui Cao* and Biaolin Yin**

Radical-Dearomative Generation of Cyclohexadienyl Pd(II) towards the 3-Dimensional Transformation of Nonactivated Phenyl Rings

Qi Fan,[‡a] Kai Jiang,[‡a] Bo Liu,[*c] Huanfeng Jiang,[a] Xiaohui Cao,[*b] Biaolin Yin[*a]

[a]Key Laboratory of Functional Molecular Engineering of Guangdong Province, School of Chemistry and Chemical Engineering, South China University of Technology (SCUT), Guangzhou 510640, China

[b]School of Pharmacy, Guangdong Pharmaceutical University, Guangzhou 510006, China

[c] The Second Clinical Medical College, and State Key Laboratory of Dampness Syndrome of Chinese Medicine, Guangzhou University of Chinese Medicine, Guangzhou, 510006, China.

*Corresponding authors.

Email: blyin@scut.edu.cn. (Biaolin Yin);

Email: caoxiaohui@gdpu.edu.cn. (Xiaohui Cao);

E-mail: doctliu@gzucm.edu.cn. (Bo Liu).

Table of contents

1 General information 1
2 Reaction optimization 2
    2.1 Reaction optimization for dearomatizing carboamination reaction 2
    2.2 Reaction optimization for dearomatizing trieneylation reaction 3
3 General procedure **A-E** for preparation of the starting materials 5
4 Characterization of the starting materials 7
5 General procedure for the synthesis of carboamination products and characterization of the carboamination products 31
    5.1 General procedure **F** for the synthesis of carboamination products 31
    5.2 Characterization of the carboamination products 31
    5.3 Failed substrates 50
6 General procedure for the synthesis of trieneylation products and characterization data. 51
    6.1 General procedure **G** for the synthesis of trieneylation products 51
    6.2 Characterization of trieneylation products 51
7 Mechanistic studies 59
    7.1 Radical trapping experiment 59
    7.2 Radical clock experiment 60
    7.3 Crossover experiment 61
    7.4 Control experiment 62
    7.5 ESI-MS Analysis 63
    7.6 On-off light experiment 63
8 Gram-scale synthesis and derivatization of the products 65
    8.1 Gram-scale synthesis 65
    8.2 Derivatization of the products 65
9 X-Ray diffraction analysis 70
    9.1 X-Ray diffraction analysis of **2l** 70
    9.2 X-Ray diffraction analysis of **2am’** 71
    9.3 X-Ray diffraction analysis of **4k** 72
    9.4 X-Ray diffraction analysis of **11** 73
10 Computational details 75
    10.1 Pathway and structure 77
    10.2 Cartesian coordinates for all calculated structure 80
11 Reference 176
12. NMR spectra of the starting materials and products 177

1 General information

All reactions were carried out in dried tubes with magnetic stirrer. Purifications of reaction products were carried out by flash chromatography using Qingdao Haiyang Chemical Co. Ltd silica gel (200-300 mesh). ^{1}H, ^{13}C NMR spectra were recorded on a Bruker AVANCE (500 MHz or 400 MHz for ^{1}H; 126 MHz or 101 MHz for ^{13}C, 471 MHz or 376 MHz for ^{19}F), ^{1}H NMR and ^{13}C NMR shifts were determined relative to internal standard TMS at δ 0.0. ^{19}F NMR shifts were determined relative to internal standard $CFCl_3$ at δ 0.0. Chemical shifts (δ) are reported in ppm, and coupling constants (J) are in Hertz (Hz). The following abbreviations were used to explain the multiplicities: s = singlet, d = doublet, t = triplet, q = quartet, m = multiplet, p = quintet, h = heptet, br = broad. Mass spectra (MS) were obtained using thermo fisher Q Exactive HR-MS. Melting points were determined using WRS-1C from INESA intelligent technology. Infrared spectra (IR) were recorded on a Brucker TENSOR 27 FTIR spectrophotometer and were reported as wavelength numbers (cm^{-1}). Infrared spectra were recorded by preparing a KBr pellet containing the title compounds. All reagents were used as received from commercial sources, unless specified otherwise, or prepared as described in the literature.

Photochemical reactor setup: A 25 *mL* Schlenk tube was placed on top of the stir plate. Reactions were irradiated from a 10 cm distance using two 30 W blue LEDs. While reactions were irradiated, they were cooled using a cooling fan.

2 Reaction optimization

2.1 Reaction optimization for dearomatizing carboamination reaction

Table S1: Reaction optimization for dearomatizing carboamination reaction

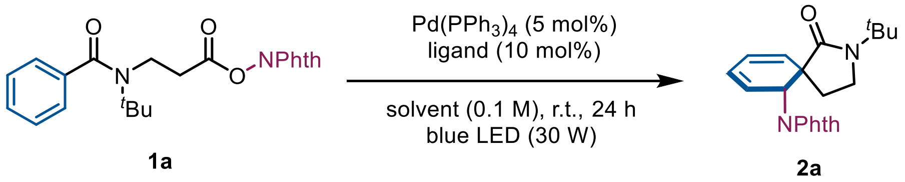

Entry	Ligand	Solvent	2a/%[a]
**1**	**L_1**	**THF**	**85 (80)[f]**
2	L_1	1,4-dioxane	<5
3	L_1	DMA	<5
4	L_1	DCE	<5
5[b]	L_2	THF	11
6[b]	L_3	THF	25
7[b]	L_4	THF	30
8[b]	L_5	THF	5
9[b]	L_6	THF	26
10[b]	L_7	THF	16
11[b]	L_8	THF	52
12[b]	L_9	THF	<5
13[b]	L_{10}	THF	<5
14[b]	L_{11}	THF	8
15[b]	L_{12}	THF	0
16[b]	L_{13}	THF	6
17[b]	L_{14}	THF	0
18[b]	L_{15}	THF	0
19[b]	L_{16}	THF	0
20[b]	L_{17}	THF	18
21[c]	L_1	THF	70
22	-	THF	<5
23[d]	L_1	THF	0
24[e]	L_1	THF	0

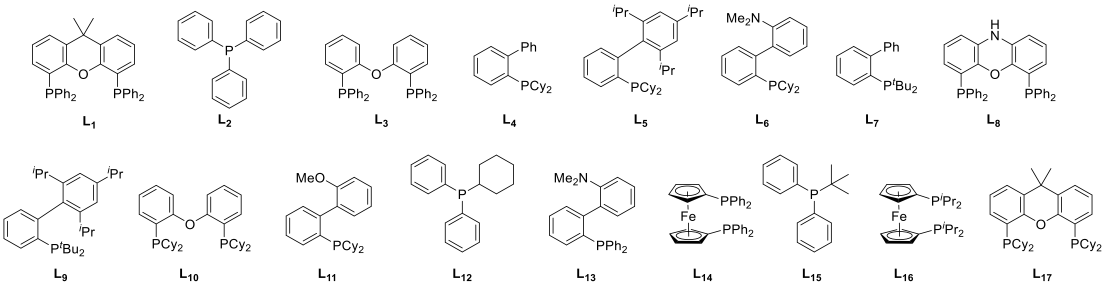

Reaction conditions: redox ester **1** (0.2 mmol), $Pd(PPh_3)_4$ (5 mol%), ligand (10 mol%),

solvent (2 mL) at room temperature under blue LEDs (30 W; 460 nm) for 24 h under argon. [a]Yield was determined by ^{1}H NMR spectroscopy analysis with dibromomethane as the internal standard. [b]Reaction time: 48 h. [c]5 mol% ligand was added. [d]Without $Pd(PPh_3)_4$. [e]Without blue LEDs, and at room temperature, 50 ℃ or 100℃. [f]Isolated yields.

2.2 Reaction optimization for dearomatizing trieneylation reaction

Table S2: Reaction optimization for dearomatizing trieneylation reaction

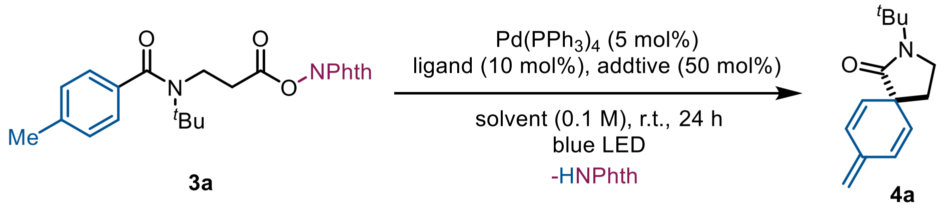

Entry	Ligand	Solvent	Additive	**4a**/%[a]
1	L_1	THF	-	55
2	L_1	THF	Li_2CO_3	25
3	L_1	THF	KF	54
4	L_1	THF	$KHCO_3$	53
5	L_1	THF	HCOONa	46
6	L_1	THF	Na_2CO_3	57
7	L_1	THF	NaOAc	5
8	L_1	THF	Et_3N	65
9	L_1	THF	DIPEA	52
10	L_1	THF	K_2CO_3	26
11	L_1	THF	Cs_2CO_3	10
12	L_1	THF	HOAc	trace
13	L_1	THF	$NaHCO_3$	67
14	L_1	DMA	$NaHCO_3$	<5
15	L_1	DMF	$NaHCO_3$	<5
16	L_1	DMSO	$NaHCO_3$	<5
17	L_1	MeCN	$NaHCO_3$	<5
18	L_1	1,4-dioxane	$NaHCO_3$	<5
19	L_1	Acetone	$NaHCO_3$	<5
20	L_1	DCE	$NaHCO_3$	<5
21	L_1	1,4-difluorobenzene	$NaHCO_3$	<5
22	L_1	NMP	$NaHCO_3$	29
23	L_3	THF	$NaHCO_3$	62
24	L_4	THF	$NaHCO_3$	58
25	L_5	THF	$NaHCO_3$	41
26	L_6	THF	$NaHCO_3$	44
27	L_{11}	THF	$NaHCO_3$	61
28	-	THF	$NaHCO_3$	31
29[b]	L_1	THF	$NaHCO_3$	64

30[c]	L_1	THF	$NaHCO_3$	63
31[d]	L_1	THF	$NaHCO_3$	54
32[e]	L_1	THF	$NaHCO_3$	66
**33[f]**	**L_1**	**THF**	**$NaHCO_3$**	**75 (70)[h]**
34[g]	L_1	THF	$NaHCO_3$	0

Reaction conditions: redox ester **3** (0.2 mmol), $Pd(PPh_3)_4$ (5 mol%), ligand (10 mol%), Additive (50 mol%), solvent (2 mL) at room temperature under blue LEDs (30 W; 460 nm) for 24 h under argon. [a]Yield was determined by ^1H NMR spectroscopy analysis with dibromomethane as the internal standard. [b]7 mol% $Pd(PPh_3)_4$ was added. [c]10 mol% $Pd(PPh_3)_4$ was added. [d]100 mol% $NaHCO_3$ was added. [e]200 mol% $NaHCO_3$ was added. [f]5 mol% ligand was added. [g]Without blue LEDs, and at room temperature, 80 ℃. [h]Isolated yields.

3 General procedure A-E for preparation of the starting materials

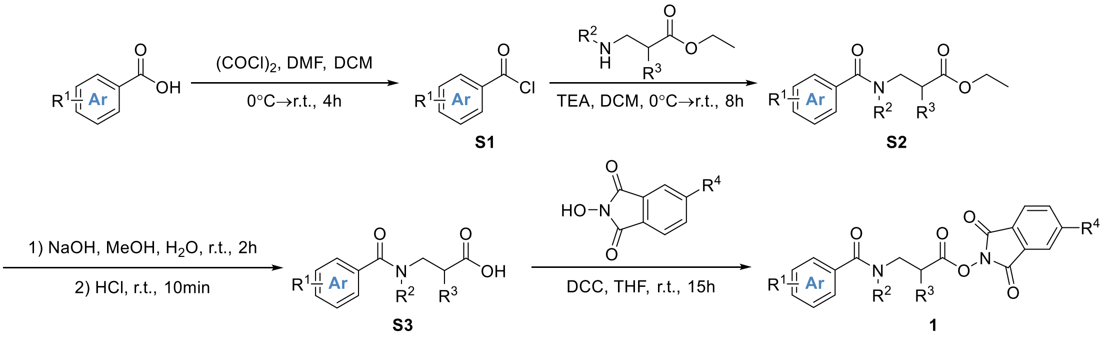

General procedure A[1]: Synthetic procedure of S1: A 100 *mL* round-bottom flask was charged with acid (10 mmol), dry DCM (20 *mL*, 0.5 M) and one drop of DMF. The reaction mixture was cooled to 0 ℃ and stirred for 5 minutes. Then, $(COCl)_2$ (1.1 *mL*, 1.3 equiv) was added dropwise to the reaction mixture and stirred at room temperature for 4 h. The resulting mixture was concentrated under reduced pressure to afford acid chloride quantitatively which was used directly without further purification for the next step.

General procedure B[1]: Synthetic procedure of S2: To a solution of β-aminocarboxylates (1.9 g, 1.1 equiv) and Et_3N (2.1 *mL*,1.5 equiv) in dry DCM (20 *mL*, 0.5 M), acid chloride (1.0 equiv) was added dropwise at 0 °C and the reaction mixture was stirred at room temperature for 12 h. Then water (40 *mL*) was added, the organic layer was separated and the aqueous layer was extracted with DCM (3 x 30 *mL*). The combined organic layer was washed with saturated aqueous $NaHCO_3$ (30 *mL*) solution followed by water (30 *mL*). After that, the organic layer was dried over Na_2SO_4 and concentrated under reduced pressure. The crude mass was purified by silica gel column chromatography using petroleum ether/ethyl acetate as eluent to afford **S2** for the next step.

General procedure C[1]: Synthetic procedure of S3: To a solution of ester **S2** in methanol (0.1 M), 0.1 M NaOH solution (1.1 equiv) was added. Reaction mixture was stirred at room temperature for 2 h and methanol was evaporated. Aqueous solution in residue was acidified with 30 *mL* of 1M HCl and stirred additionally for 10 min. Formed precipitate was collected, washed with water and dried in vacuo. Beige solid **S3** was obtained.

General procedure D[1]: Synthetic procedure of S4: DCC (1.5 equiv) was added to a solution of acid **S3** (1 equiv) and N-hydroxyphthalimide (1.05 equiv) in THF (0.2 M). Reaction mixture was stirred at room temperature for 15 h, filtered and evaporated. Purification by recrystallization from petrol ether and ethyl acetate gave **1** of white solid.

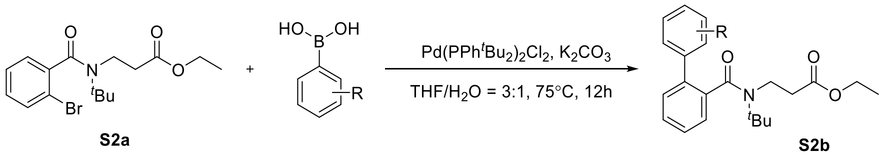

General procedure E: Synthetic procedure of S2b: A 100 *mL* Schlenk tube was charged with phenylboronic acid (5.25 mmol, 1.05 equiv), $Pd(PPh^tBu_2)_2Cl_2$ (0.025 mmol, 0.5 mol %) and K_2CO_3 (7.5 mmol, 1.5 equiv). The Schlenk tube was put on vacuum and backfilled with argon

three times. Afterwards, **S2a** (5 mmol, 1 equiv) dissolved in 15 *mL* THF and 5 *mL* water (0.25 M) were added into the Schlenk tube. The mixture was stirred at 75 ℃ for 12 hours until **S2a** was completely consumed. Then water (30 *mL*) and DCM (30 *mL*) were added, the organic layer was separated and the aqueous layer was extracted with DCM (3×30 *mL*). The combined organic layer was dried over Na_2SO_4 and concentrated under reduced pressure. The crude mass was purified by silica gel column chromatography using petroleum ether/ethyl acetate as eluent to afford **S2b** for the next step.

4 Characterization of the starting materials

1,3-Dioxoisoindolin-2-yl 3-(*N*-(tert-butyl)benzamido)propanoate (1a)

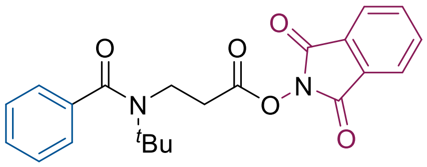

The title compound was prepared through general procedure **A-D** and purified by recrystallization from petroleum ether /ethyl acetate (v/v, = 6/1) as white solid (3.0 g, 76%);

M.p. 98.4 - 100.4 ℃.

^{1}H NMR (400 MHz, CDCl$_3$) *δ* 7.87 (dd, *J* = 5.5, 3.1 Hz, 2H), 7.79 (dd, *J* = 5.6, 3.1 Hz, 2H), 7.46 – 7.35 (m, 5H), 3.81 – 3.73 (m, 2H), 2.91 – 2.85 (m, 2H), 1.59 (s, 9H).

^{13}C NMR (101 MHz, CDCl$_3$) *δ* 173.7, 167.0, 161.6, 139.0, 134.9, 129.3, 128.8, 128.7, 126.0, 124.0, 57.5, 42.3, 33.2, 29.0.

IR (KBr) 2966, 2832, 1601, 1363, 1079, 775, 522 cm^{-1}.

HRMS (ESI-MS) *m/z* calcd for $C_{22}H_{22}N_2O_5$ [M+H]$^+$: 395.1601. found: 395.1597.

5-Methyl-1,3-Dioxoisoindolin-2-yl 3-(*N*-(tert-butyl)benzamido)propanoate (1b)

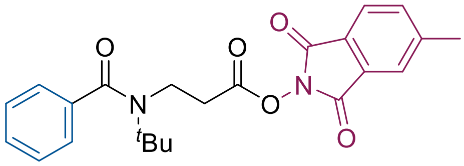

The title compound was prepared through general procedure **A-D** and purified by recrystallization from petroleum ether /ethyl acetate (v/v, = 6/1) as white solid (3.19 g, 78%).

M.p. 121.2 - 122.3 ℃.

^{1}H NMR (400 MHz, CDCl$_3$) *δ* 7.73 (d, *J* = 7.7 Hz, 1H), 7.65 (s, 1H), 7.56 (d, *J* = 7.7 Hz, 1H), 7.45 – 7.31 (m, 5H), 3.80 – 3.69 (m, 2H), 2.91 – 2.79 (m, 2H), 2.51 (s, 3H), 1.58 (s, 9H).

^{13}C NMR (101 MHz, CDCl$_3$) *δ* 173.7, 167.0, 161.9, 161.8, 146.4, 139.0, 135.4, 129.3, 129.0, 128.7, 126.0, 126.0, 124.5, 124.0, 57.5, 42.3, 33.2, 29.0, 22.2.

IR (KBr) 3647, 3324, 2929, 1742, 1627, 1365, 1195, 1126, 1082, 1025, 982, 922, 840, 784, 709, 646, 531, 501 cm^{-1}.

HRMS (ESI-MS) *m/z* calcd for $C_{23}H_{24}N_2O_5$ [M+H]$^+$: 409.1758. found: 409.1751.

1,3-Dioxoisoindolin-2-yl 3-(*N*-(tert-butyl)-3',5'-dichloro-[1,1'-biphenyl]-2-carboxamido)propanoate (1c)

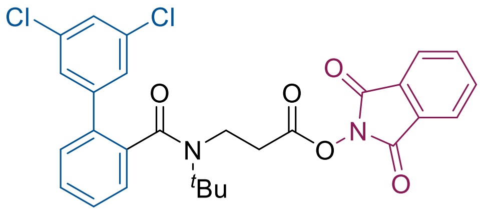

The title compound was prepared through general procedure **A-B,** general procedure **E** and general procedure **C-D** and purified by recrystallization from petroleum ether /ethyl acetate (v/v, = 6/1) as white solid (3.78 g, 70%).

M.p. 169.4 - 171.0 ℃.

^{1}H NMR (400 MHz, CDCl$_3$) *δ* 7.86 (dd, *J* = 5.5, 3.2 Hz, 2H), 7.79 (dd, *J* = 5.5, 3.1 Hz, 2H), 7.51 – 7.44 (m, 4H), 7.42 – 7.36 (m, 3H), 3.47 – 3.31 (m, 2H), 2.63 (dd, *J* = 10.0, 6.1 Hz, 2H), 1.44 (s, 9H).

^{13}C NMR (101 MHz, CDCl$_3$) δ 171.5, 166.7, 161.6, 142.5, 137.7, 135.0, 134.9, 134.7, 129.8, 129.3, 128.9, 128.7, 127.8, 127.5, 126.8, 124.0, 57.8, 41.6, 32.9, 28.6.

IR (KBr) 3694, 3068, 2969, 2833, 2713, 1786, 1745, 1599, 1475, 1363, 1190, 1126, 1079, 1027, 966, 872, 771, 697, 642, 561, 515 cm^{-1}.

HRMS (ESI-MS) *m/z* calcd for $C_{28}H_{24}Cl_2N_2O_5$ $[M+H]^+$: 539.1135. found: 539.1128.

1,3-Dioxoisoindolin-2-yl 3-(*N*-(tert-butyl)-4-cyclopropylbenzamido)propanoate (1e)

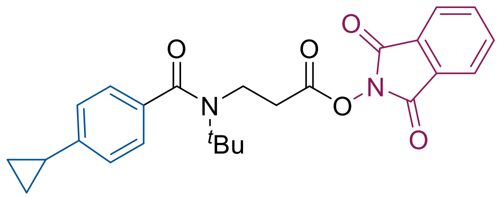

The title compound was prepared through general procedure **A-D** and purified by recrystallization from petroleum ether /ethyl acetate (v/v, = 6/1) as white solid (1.74 g, 40%).

M.p. 115.0 - 117.0 ℃.

^{1}H NMR (400 MHz, CDCl$_3$) δ 7.81 (dd, *J* = 5.5, 3.1 Hz, 2H), 7.73 (dd, *J* = 5.5, 3.1 Hz, 2H), 7.24 (d, *J* = 8.0 Hz, 2H), 7.06 (d, *J* = 7.9 Hz, 2H), 3.77 – 3.70 (m, 2H), 2.87 – 2.81 (m, 2H), 1.86 (tt, *J* = 8.5, 5.1 Hz, 1H), 1.53 (s, 9H), 0.97 – 0.90 (m, 2H), 0.69 (dt, *J* = 6.7, 4.6 Hz, 2H).

^{13}C NMR (101 MHz, CDCl$_3$) δ 173.9, 167.1, 161.6, 145.6, 136.0, 134.9, 128.7, 126.2, 125.8, 124.0, 57.3, 42.3, 33.2, 29.0, 15.4, 9.6.

IR (KBr) 3665, 3528, 2970, 2833, 2716, 1785, 1745, 1602, 1470, 1362, 1190, 1127, 1078, 968, 885, 834, 775, 731, 703, 584, 510 cm^{-1}.

HRMS (ESI-MS) *m/z* calcd for $C_{25}H_{26}N_2O_5$ $[M+H]^+$: 435.1914. found: 435.1907.

1,3-Dioxoisoindolin-2-yl 3-(*N*-(tert-butyl)-2-methylbenzamido)propanoate (1f)

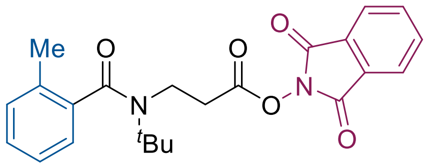

The title compound was prepared through general procedure **A-D** and purified by recrystallization from petroleum ether /ethyl acetate (v/v, = 6/1) as white solid (3.02 g, 74%).

M.p. 110.7 - 111.9 ℃.

^{1}H NMR (400 MHz, CDCl$_3$) δ 7.86 (dd, *J* = 5.5, 3.1 Hz, 2H), 7.78 (dd, *J* = 5.5, 3.1 Hz, 2H), 7.32 – 7.12 (m, 4H), 3.93 – 3.64 (m, 1H), 3.64 – 3.37 (m, 1H), 2.82 (td, *J* = 7.6, 3.6 Hz, 2H), 2.32 (s, 3H), 1.61 (s, 9H).

^{13}C NMR (101 MHz, CDCl$_3$) δ 172.7, 166.9, 161.6, 138.5, 134.9, 133.1, 130.7, 128.7, 128.7, 126.1, 125.1, 124.0, 57.7, 41.7, 33.0, 29.0, 18.8.

IR (KBr) 2966, 2832, 1600, 1363, 1080, 775, 521 cm^{-1}.

HRMS (ESI-MS) *m/z* calcd for $C_{23}H_{24}N_2O_5$ $[M+H]^+$: 409.1758. found: 409.1751.

1,3-Dioxoisoindolin-2-yl 3-(*N*-(tert-butyl)-2-ethylbenzamido)propanoate (1g)

The title compound was prepared through general procedure **A-D** and purified by

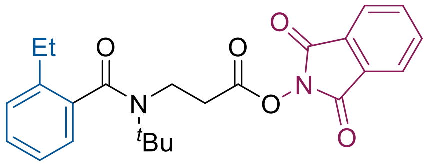

recrystallization from petroleum ether /ethyl acetate (v/v, = 6/1) as white solid (3.21g, 76%).

M.p. 118.5 - 120.1 ℃.

^{1}H NMR (400 MHz, $CDCl_3$) δ 7.88 – 7.82 (m, 2H), 7.77 (dd, J = 6.4, 2.8 Hz, 2H), 7.34 – 7.21 (m, 3H), 7.17 (d, J = 7.4 Hz, 1H), 3.73 (dt, J = 15.8, 7.4 Hz, 1H), 3.55 (dd, J = 16.0, 8.1 Hz, 1H), 2.83 (td, J = 7.7, 2.9 Hz, 2H), 2.64 (qd, J = 7.6, 2.5 Hz, 2H), 1.61 (s, 9H), 1.27 (t, J = 7.5 Hz, 3H).
^{13}C NMR (101 MHz, $CDCl_3$) δ 172.7, 166.9, 161.6, 139.2, 138.0, 134.9, 129.0, 128.9, 128.7, 126.1, 125.1, 124.0, 57.6, 41.9, 33.1, 29.0, 25.8, 15.0.
IR (KBr) 2966, 2832, 1601, 1363, 1080, 878, 775, 522 cm^{-1}.
HRMS (ESI-MS) *m/z* calcd for $C_{24}H_{26}N_2O_5$ $[M+H]^+$: 423.1914. found: 423.1907.

1,3-Dioxoisoindolin-2-yl 3-(*N*-(tert-butyl)-2-isopropylbenzamido)propanoate (1h)

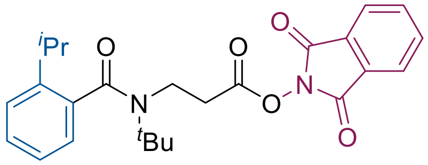

The title compound was prepared through general procedure **A-D** and purified by recrystallization from petroleum ether /ethyl acetate (v/v, = 6/1) as white solid (3.06g, 70%).

M.p. 124.4 - 126.9 ℃.
^{1}H NMR (400 MHz, $CDCl_3$) δ 7.85 (dd, J = 5.5, 3.1 Hz, 2H), 7.77 (dd, J = 5.5, 3.1 Hz, 2H), 7.38 – 7.31 (m, 2H), 7.23 (ddd, J = 8.4, 5.5, 3.1 Hz, 1H), 7.14 (d, J = 7.1 Hz, 1H), 3.73 (dt, J = 15.3, 7.3 Hz, 1H), 3.57 (dt, J = 15.8, 8.1 Hz, 1H), 2.96 (p, J = 6.8 Hz, 1H), 2.84 (t, J = 7.8 Hz, 2H), 1.61 (s, 9H), 1.31 (d, J = 6.9 Hz, 3H), 1.26 (d, J = 6.8 Hz, 3H).
^{13}C NMR (126 MHz, $CDCl_3$) δ 172.7, 166.9, 161.6, 144.1, 137.4, 134.9, 129.1, 128.7, 126.1, 126.0, 125.0, 124.0, 57.6, 42.0, 33.1, 30.7, 29.0, 24.9, 23.0.
IR (KBr) 2964, 2832, 1601, 1362, 1079, 877, 772, 521 cm^{-1}.
HRMS (ESI-MS) *m/z* calcd for $C_{25}H_{28}N_2O_5$ $[M+H]^+$: 437.2071. found: 437.2065.

1,3-Dioxoisoindolin-2-yl 3-(2-benzyl-*N*-(tert-butyl)benzamido)propanoate (1i)

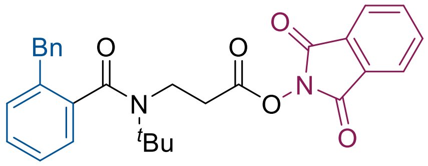

The title compound was prepared through general procedure **A-D** and purified by recrystallization from petroleum ether /ethyl acetate (v/v, = 6/1) as white solid (3.63g, 75%).

M.p. 106.2 - 108.2 ℃.
^{1}H NMR (400 MHz, $CDCl_3$) δ 7.87 (m, 2H), 7.78 (m, 2H), 7.34 – 7.18 (m, 9H), 4.18 – 3.93 (m, 2H), 3.53 – 3.28 (m, 2H), 2.80 – 2.47 (m, 2H), 1.56 (s, 9H).
^{13}C NMR (101 MHz, $CDCl_3$) δ 172.7, 166.9, 161.6, 139.9, 138.3, 137.1, 134.8, 130.9, 129.4, 129.0, 128.8, 128.6, 126.5, 126.4, 125.7, 124.0, 57.6, 41.8, 38.6, 32.9, 28.9.
IR (KBr) 2970, 2834, 1598, 1358, 1079, 878, 775, 519 cm^{-1}.
HRMS (ESI-MS) *m/z* calcd for $C_{29}H_{28}N_2O_5$ $[M+H]^+$: 485.2071. found: 485.2064.

1,3-Dioxoisoindolin-2-yl 3-(*N*-(tert-butyl)-2-fluorobenzamido)propanoate (1j)

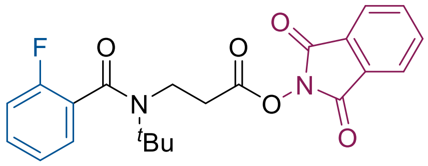

The title compound was prepared through general procedure **A-D** and purified by recrystallization from petroleum ether /ethyl acetate (v/v, = 6/1) as white solid (3.3 g, 80%).

M.p. 99.5 - 100.8 ℃.

^{1}H NMR (500 MHz, CDCl$_3$) *δ* 7.85 (dd, *J* = 5.5, 3.1 Hz, 2H), 7.77 (dd, *J* = 5.6, 3.1 Hz, 2H), 7.39 – 7.27 (m, 2H), 7.20 (t, *J* = 7.5 Hz, 1H), 7.11 (t, *J* = 8.9 Hz, 1H), 3.73 (t, *J* = 7.8 Hz, 2H), 2.87 (t, *J* = 8.1 Hz, 2H), 1.58 (s, 9H).

^{13}C NMR (126 MHz, CDCl$_3$) *δ* 167.9, 166.9, 161.6, 157.6 (d, *J* = 245.9 Hz), 134.9, 130.8 (d, *J* = 7.8 Hz), 128.7, 127.9 (d, *J* = 3.8 Hz), 126.9 (d, *J* = 18.5 Hz), 124.8 (d, *J* = 3.3 Hz), 124.0, 116.1 (d, *J* = 21.4 Hz), 58.0, 42.0, 33.0, 29.0.

^{19}F NMR (471 MHz, CDCl$_3$) *δ* -116.57.

IR (KBr) 2974, 2832, 1745, 1604, 1487, 1363, 1080, 968, 878, 773, 701, 517 cm^{-1}.

HRMS (ESI-MS) *m/z* calcd for $C_{22}H_{21}FN_2O_5$ [M+H]$^+$: 413.1507. found: 413.1502.

1,3-Dioxoisoindolin-2-yl 3-(*N*-(tert-butyl)-2-chlorobenzamido)propanoate (1k)

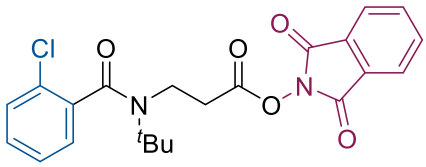

The title compound was prepared through general procedure **A-D** and purified by recrystallization from petroleum ether /ethyl acetate (v/v, = 6/1) as white solid (3.3 g, 77%).

M.p. 120.6 - 121.5 ℃.

^{1}H NMR (400 MHz, CDCl$_3$) *δ* 7.86 (dd, *J* = 5.5, 3.2 Hz, 2H), 7.79 (dd, *J* = 5.5, 3.1 Hz, 2H), 7.45 – 7.38 (m, 1H), 7.38 – 7.27 (m, 3H), 3.78 (ddd, *J* = 15.9, 10.5, 4.8 Hz, 1H), 3.59 (ddd, *J* = 15.9, 10.4, 5.5 Hz, 1H), 3.04 – 2.87 (m, 1H), 2.80 (ddd, *J* = 16.5, 10.5, 5.5 Hz, 1H), 1.61 (s, 9H).

^{13}C NMR (101 MHz, CDCl$_3$) *δ* 169.3, 166.9, 161.6, 137.9, 134.9, 134.9, 130.0, 129.7, 128.7, 127.3, 127.1, 124.0, 58.1, 41.8, 32.9, 28.9.

IR (KBr) 2832, 1600, 1363, 1080, 774, 523 cm^{-1}.

HRMS (ESI-MS) *m/z* calcd for $C_{22}H_{21}ClN_2O_5$ [M+H]$^+$: 429.1212. found: 429.1207.

1,3-Dioxoisoindolin-2-yl 3-(*N*-(tert-butyl)-2-(trifluoromethyl)benzamido)propanoate (1l)

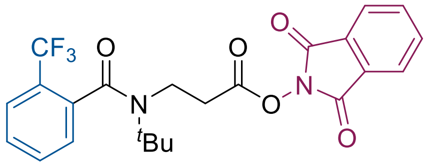

The title compound was prepared through general procedure **A-D** and purified by recrystallization from petroleum ether /ethyl acetate (v/v, = 6/1) as white solid (3.61 g, 78%).

M.p. 134.4 - 136.1 ℃.

^{1}H NMR (500 MHz, CDCl$_3$) *δ* 7.85 (dd, *J* = 5.5, 3.1 Hz, 2H), 7.77 (dd, *J* = 5.5, 3.1 Hz, 2H), 7.70 (d, *J* = 8.0 Hz, 1H), 7.62 (t, *J* = 7.6 Hz, 1H), 7.50 (t, *J* = 7.7 Hz, 1H), 7.35 (d, *J* = 7.6 Hz, 1H), 3.72 (ddd, *J* = 16.1, 11.1, 5.2 Hz, 1H), 3.59 – 3.42 (m, 1H), 2.99 – 2.85 (m, 1H), 2.78 (ddd, *J* = 16.4, 10.4, 5.3 Hz, 1H), 1.58 (s, 9H).

^{13}C NMR (126 MHz, CDCl$_3$) *δ* ^{13}C NMR (126 MHz, CDCl$_3$) δ 169.7, 166.8, 161.6, 136.8, 134.9, 132.3, 129.0, 128.7, 127.0 (q, *J* = 4.6 Hz), 126.7, 126.0 (q, *J* = 32.1 Hz), 124.0, 123.8 (q, *J* = 273.7 Hz), 42.0, 32.8, 28.6.

^{19}F NMR (471 MHz, CDCl$_3$) *δ* -59.6.

IR (KBr) 3777, 2927, 2781, 1788, 1747, 1626, 1369, 1314, 1265, 1124, 1084, 967, 881, 771, 699, 570 cm^{-1}.

HRMS (ESI-MS) *m/z* calcd for $C_{23}H_{21}F_3N_2O_5$ $[M+Na]^+$: 485.1295. found: 485.1311.

1,3-Dioxoisoindolin-2-yl 3-(*N*-(tert-butyl)-2-(trifluoromethoxy)benzamido)propanoate (1m)

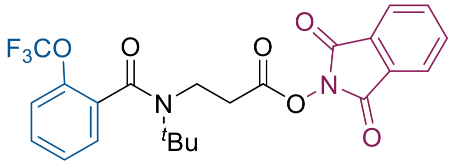

The title compound was prepared through general procedure **A-D** and purified by recrystallization from petroleum ether /ethyl acetate (v/v, = 6/1) as white solid (3.44 g, 72%).

M.p. 140.8 - 142.3 ℃.
^{1}H NMR (500 MHz, CDCl$_3$) δ 7.86 (dd, *J* = 5.5, 3.1 Hz, 2H), 7.78 (dd, *J* = 5.6, 3.1 Hz, 2H), 7.46 (t, *J* = 7.9 Hz, 1H), 7.33 – 7.21 (m, 3H), 3.79 – 3.69 (m, 2H), 2.92 – 2.82 (m, 2H), 1.57 (s, 9H).
^{13}C NMR (126 MHz, CDCl$_3$) δ 171.8, 166.8, 161.6, 149.3, 140.8, 134.9, 130.4, 128.8, 124.5, 124.0, 121.7, 120.4 (q, *J* = 258.5 Hz), 119.0, 57.8, 42.3, 33.0, 28.9.
^{19}F NMR (471 MHz, CDCl$_3$) δ -57.76.
IR (KBr) 2832, 1600, 1363, 1080, 775, 523 cm^{-1}.
HRMS (ESI-MS) *m/z* calcd for $C_{23}H_{21}F_3N_2O_6$ $[M+H]^+$: 479.1424. found: 479.1418.

1,3-Dioxoisoindolin-2-yl 3-(*N*-(tert-butyl)-2-cyanobenzamido)propanoate (1n)

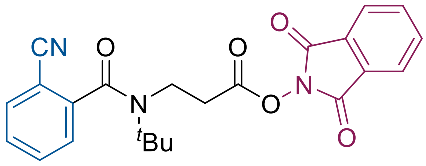

The title compound was prepared through general procedure **A-D** and purified by recrystallization from petroleum ether /ethyl acetate (v/v, = 6/1) as white solid (2.94 g, 70%).

M.p. 119.8 - 121.4 ℃.
^{1}H NMR (400 MHz, CDCl$_3$) δ 7.85 (dd, *J* = 5.6, 3.1 Hz, 2H), 7.78 (dd, *J* = 5.6, 3.1 Hz, 2H), 7.73 – 7.62 (m, 2H), 7.48 (dd, *J* = 7.8, 5.8 Hz, 2H), 3.77 – 3.66 (m, 2H), 2.91 (t, *J* = 7.5 Hz, 2H), 1.61 (s, 9H).
^{13}C NMR (101 MHz, CDCl$_3$) δ 169.1, 166.8, 161.5, 142.3, 135.0, 133.4, 133.0, 129.3, 128.7, 127.0, 124.0, 116.8, 109.3, 58.3, 42.2, 32.9, 28.8.
IR (KBr) 2978, 2832, 2229, 1743, 1601, 1362, 1080, 968, 878, 774, 519 cm^{-1}.
HRMS (ESI-MS) *m/z* calcd for $C_{23}H_{21}N_3O_5$ $[M+H]^+$: 420.1554. found: 420.1544.

1,3-Dioxoisoindolin-2-yl 3-(*N*-(tert-butyl)-2-(naphthalen-2-yl)benzamido)propanoate (1o)

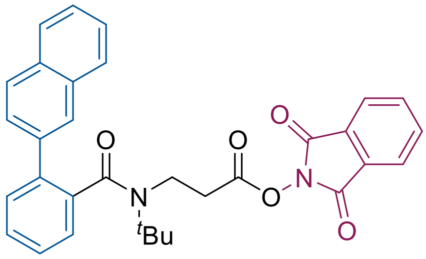

The title compound was prepared through general procedure **A-B,** general procedure **E** and general procedure **C-D** and purified by recrystallization from petroleum ether /ethyl acetate (v/v, = 6/1) as white solid (3.90 g, 75%).

M.p. 97.2 - 99.0.
^{1}H NMR (500 MHz, CDCl$_3$) δ 8.10 (s, 1H), 7.98 – 7.84 (m, 5H), 7.75 (ddd, *J* = 15.9, 7.1, 2.4 Hz, 3H), 7.59 – 7.38 (m, 6H), 3.44 (ddd, *J* = 16.2, 10.4, 6.0 Hz, 1H), 3.33 – 3.28 (m, 1H), 2.60 – 2.39 (m, 2H), 1.35 (s, 9H).

^{13}C NMR (126 MHz, CDCl$_3$)** δ 172.4, 166.9, 161.6, 138.0, 137.4, 137.1, 134.9, 133.2, 132.7, 130.3, 129.2, 128.8, 128.4, 128.3, 127.9, 127.7, 127.3, 126.9, 126.4, 126.3, 124.0, 68.0, 57.6, 41.6, 28.6.

IR (KBr) 3668, 2970, 2833, 1742, 1602, 1363, 1080, 967, 876, 775, 520 cm^{-1}.

HRMS (ESI-MS) *m/z* calcd for $C_{32}H_{28}N_2O_5$ [M+H]$^+$: 521.2071. found: 521.2065.

1,3-Dioxoisoindolin-2-yl 3-(*N*-(tert-butyl)-[1,1'-biphenyl]-2-carboxamido)propanoate (1p)

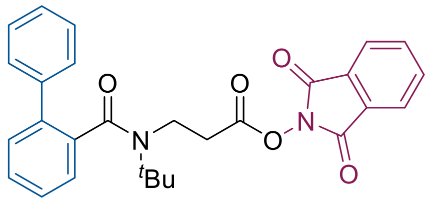

The title compound was prepared through general procedure **A-D** and purified by recrystallization from petroleum ether /ethyl acetate (v/v, = 6/1) as white solid (3.53 g, 75%).

M.p. 132.2 - 134.3 ℃.

^{1}H NMR (400 MHz, CDCl$_3$) δ 7.85 (dd, *J* = 5.5, 3.1 Hz, 2H), 7.77 (dd, *J* = 5.5, 3.1 Hz, 2H), 7.61 – 7.55 (m, 2H), 7.46 – 7.33 (m, 7H), 3.36 (dtt, *J* = 22.1, 16.2, 8.9 Hz, 2H), 2.54 (td, *J* = 10.2, 4.9 Hz, 1H), 2.49 – 2.34 (m, 1H), 1.39 (s, 9H).

^{13}C NMR (101 MHz, CDCl$_3$) δ 172.2, 166.9, 161.6, 139.8, 137.8, 137.6, 134.9, 129.9, 129.3, 129.1, 128.8, 128.5, 127.8, 127.7, 126.7, 124.0, 57.5, 41.6, 32.8, 28.6.

IR (KBr) 3653, 3056, 2976, 1745, 1642, 1474, 1396, 1295, 1192, 1080, 1025, 967, 879, 782, 743, 701, 647, 595, 519 cm^{-1}.

HRMS (ESI-MS) *m/z* calcd for $C_{28}H_{26}N_2O_5$ [M+H]$^+$: 471.1914. found: 471.1908.

1,3-Dioxoisoindolin-2-yl 3-(*N*-(tert-butyl)-4'-(trifluoromethyl)-[1,1'-biphenyl]-2-carboxamido)pro- panoate (1q)

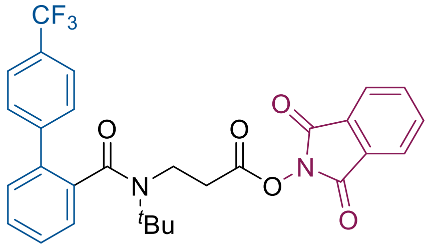

The title compound was prepared through general procedure **A-B,** general procedure **E** and general procedure **C-D** and purified by recrystallization from petroleum ether /ethyl acetate (v/v, = 6/1) as white solid (3.77 g, 70%).

M.p. 127.2 - 129.5 ℃.

^{1}H NMR (400 MHz, CDCl$_3$) δ 7.87 (dd, *J* = 5.5, 3.1 Hz, 2H), 7.80 (dd, *J* = 5.5, 3.1 Hz, 2H), 7.71 (s, 4H), 7.51 – 7.45 (m, 2H), 7.44 – 7.36 (m, 2H), 3.43 – 3.30 (m, 2H), 2.64 – 2.42 (m, 2H), 1.39 (s, 9H).

^{13}C NMR (126 MHz, CDCl$_3$) δ 171.8, 166.7, 161.6, 143.4, 137.8, 136.1, 134.9, 129.9, 129.9 (q, *J* = 32.4 Hz), 129.7, 129.3, 128.7, 128.6, 126.7, 125.4 (q, *J* = 3.8 Hz), 124.1 (q, *J* = 272.5 Hz), 124.0, 57.7, 41.7, 32.9, 28.6.

^{19}F NMR (376 MHz, CDCl$_3$) δ -62.5.

IR (KBr) 3670, 2976, 1746, 1643, 1470, 1368, 1325, 1165, 1024, 966, 877, 847, 773, 701, 607, 518 cm^{-1}.

HRMS (ESI-MS) *m/z* calcd for $C_{29}H_{25}F_3N_2O_5$ [M+H]$^+$: 539.1788, found: 539.1780.

1,3-Dioxoisoindolin-2-yl 3-(*N*-(tert-butyl)-4'-(trifluoromethoxy)-[1,1'-biphenyl]-2-carboxamido)p-ropanoate (1r)

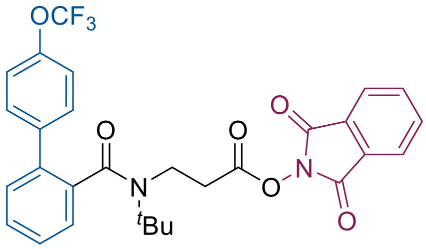

The title compound was prepared through general procedure **A-B,** general procedure **E** and general procedure **C-D** and purified by recrystallization from petroleum ether /ethyl acetate (v/v, = 6/1) as white solid (4.21 g, 77%).

M.p. 128.6 - 130.5 ℃.

^{1}H NMR (400 MHz, CDCl$_3$) δ 7.86 (dd, J = 5.5, 3.1 Hz, 2H), 7.79 (dd, J = 5.5, 3.1 Hz, 2H), 7.61 (d, J = 8.7 Hz, 2H), 7.45 (dd, J = 5.7, 3.3 Hz, 2H), 7.37 (ddd, J = 8.8, 5.8, 3.4 Hz, 2H), 7.29 (d, J = 8.0 Hz, 2H), 3.35 (t, J = 8.2 Hz, 2H), 2.65 – 2.51 (m, 1H), 2.46 (q, J = 9.4, 8.2 Hz, 1H), 1.38 (s, 9H).

^{13}C NMR (101 MHz, CDCl$_3$) δ 171.9, 166.8, 161.6, 148.9 (q, J = 1.8 Hz), 138.5, 137.8, 136.1, 134.9, 130.8, 129.8, 129.2, 128.7, 128.3, 126.6, 124.0, 121.0, 120.5 (q, J = 257.3 Hz), 57.6, 41.7, 32.9, 28.6.

^{19}F NMR (376 MHz, CDCl$_3$) δ -57.8.

IR (KBr) 3667, 2979, 1746, 1647, 1513, 1476, 1398, 1267, 1025, 966, 878, 739, 701, 521 cm^{-1}.

HRMS (ESI-MS) *m/z* calcd for $C_{29}H_{25}F_3N_2O_6$ $[M+H]^+$: 555.1737. found: 555.1728.

1,3-Dioxoisoindolin-2-yl 3-(*N*,4'-di-tert-butyl-[1,1'-biphenyl]-2-carboxamido)propanoate (1s)

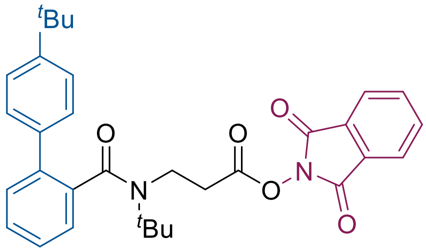

The title compound was prepared through general procedure **A-B,** general procedure **E** and general procedure **C-D** and purified by recrystallization from petroleum ether /ethyl acetate (v/v, = 6/1) as white solid (3.79 g, 72%).

M.p. 134.7 - 137.3 ℃.

^{1}H NMR (400 MHz, CDCl$_3$) δ 7.87 (dd, J = 5.5, 3.1 Hz, 2H), 7.78 (dd, J = 5.5, 3.1 Hz, 2H), 7.48 (q, J = 8.6 Hz, 4H), 7.42 – 7.35 (m, 4H), 3.45 – 3.24 (m, 2H), 2.52 (ddd, J = 16.3, 10.3, 5.9 Hz, 1H), 2.43 – 2.29 (m, 1H), 1.39 – 1.35 (m, 18H).

^{13}C NMR (126 MHz, CDCl$_3$) δ 172.3, 166.9, 161.6, 150.8, 137.5, 136.8, 134.9, 129.7, 129.0, 128.9, 128.9, 128.7, 127.6, 126.6, 125.4, 124.0, 57.5, 41.6, 34.6, 31.4, 28.6.

IR (KBr) 3669, 3271, 2961, 2308, 1745, 1641, 1519, 1476, 1366, 1192, 1079, 1025, 966, 879, 840, 772, 583, 521 cm^{-1}.

HRMS (ESI-MS) *m/z* calcd for $C_{32}H_{34}N_2O_5$ $[M+H]^+$: 527.2540. found: 527.2531.

1,3-Dioxoisoindolin-2-yl 3-(*N*-(tert-butyl)-4'-methyl-[1,1'-biphenyl]-2-carboxamido)propanoate (1t)

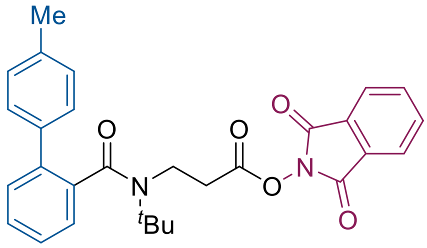

The title compound was prepared through general procedure **A-B,** general procedure **E** and general procedure **C-D** and purified by recrystallization from petroleum ether /ethyl acetate (v/v, = 6/1) as white solid (3.15 g, 65%).

M.p. 103.4 - 105.9 ℃.

^{1}H NMR (400 MHz, CDCl$_3$) δ 7.86 (dd, J = 5.4, 3.1 Hz, 2H), 7.78 (dd, J = 5.5, 3.1 Hz, 2H), 7.48 (d, J = 7.7 Hz, 2H), 7.41 (d, J = 6.4 Hz, 3H), 7.36 – 7.31 (m, 1H), 7.24 (d, J = 7.8 Hz, 2H), 3.42-3.33 (m, 2H), 2.58-2.41 (m, 5H), 1.42 (s, 9H).

^{13}C NMR (101 MHz, CDCl$_3$) δ 172.4, 166.9, 161.6, 137.8, 137.5, 136.9, 134.9, 129.9, 129.2, 129.1, 129.0, 128.8, 127.5, 126.7, 124.0, 57.5, 41.6, 34.0, 28.7, 21.2.

IR (KBr) 3668, 2925, 1788, 1746, 1641, 1475, 1396, 1192, 1079, 1025, 966, 879, 826, 760, 701, 520 cm^{-1}.

HRMS (ESI-MS) *m/z* calcd for $C_{29}H_{28}N_2O_5$ [M+H]$^+$: 485.2071. found: 485.2064.

1,3-Dioxoisoindolin-2-yl 3-(N-(tert-butyl)-4'-fluoro-[1,1'-biphenyl]-2-carboxamido)propanoate (1u)

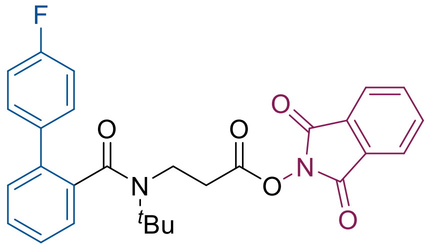

The title compound was prepared through general procedure **A-B,** general procedure **E** and general procedure **C-D** and purified by recrystallization from petroleum ether /ethyl acetate (v/v, = 6/1) as white solid (3.42 g, 70%).

M.p. 120.2 - 122.1 ℃.

^{1}H NMR (400 MHz, CDCl$_3$) δ 7.86 (dd, J = 5.5, 3.1 Hz, 2H), 7.79 (dd, J = 5.5, 3.1 Hz, 2H), 7.60 – 7.52 (m, 2H), 7.47 – 7.31 (m, 4H), 7.13 (t, J = 8.7 Hz, 2H), 3.36 (t, J = 8.5 Hz, 2H), 2.64 – 2.51 (m, 1H), 2.50 – 2.34 (m, 1H), 1.41 (s, 9H).

^{13}C NMR (101 MHz, CDCl$_3$) δ 172.1, 166.8, 162.6 (d, J = 247.3 Hz), 161.6, 137.8, 136.5, 135.8 (d, J = 3.3 Hz), 134.9, 131.0 (d, J = 8.0 Hz), 129.9, 129.1, 128.8, 127.9, 126.6, 124.0, 115.4 (d, J = 21.3 Hz), 57.6, 41.6, 32.9, 28.6.

^{19}F NMR (376 MHz, CDCl$_3$) δ -114.5.

IR (KBr) 3668, 3061, 2978, 1745, 1644, 1514, 1477, 1397, 1225, 1192, 1080, 1024, 966, 878, 843, 737, 701, 646, 568, 522 cm^{-1}.

HRMS (ESI-MS) *m/z* calcd for $C_{28}H_{25}FN_2O_5$ [M+H]$^+$: 489.1820. found: 489.1810.

1,3-Dioxoisoindolin-2-yl 3-(*N*-(tert-butyl)-4'-chloro-[1,1'-biphenyl]-2-carboxamido)propanoate (1v)

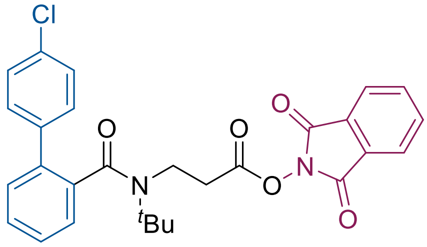

The title compound was prepared through general procedure **A-B,** general procedure **E** and general procedure **C-D** and purified by recrystallization from petroleum ether /ethyl acetate (v/v, = 6/1) as white solid (3.59 g, 71%).

M.p. 113.5 - 115.8 ℃.

^{1}H NMR (400 MHz, CDCl$_3$) δ 7.87 (dd, J = 5.5, 3.1 Hz, 2H), 7.79 (dd, J = 5.5, 3.1 Hz, 2H), 7.53 (d, J = 8.5 Hz, 2H), 7.47 – 7.33 (m, 6H), 3.36 (t, J = 8.0 Hz, 2H), 2.58 (dt, J = 16.3, 8.1 Hz, 1H), 2.52 – 2.40 (m, 1H), 1.42 (s, 9H).

^{13}C NMR (101 MHz, CDCl$_3$) δ 172.0, 166.8, 161.6, 137.7, 138.2, 136.2, 134.9, 134.0, 130.6, 129.98, 129.1, 128.8, 128.7, 128.2, 126.7, 124.0, 57.6, 41.7, 32.9, 28.7.

IR (KBr) 2976, 1742, 1640, 1474, 1365, 1081, 1024, 966, 877, 836, 771, 521 cm^{-1}.

HRMS (ESI-MS) *m/z* calcd for $C_{28}H_{25}ClN_2O_5$ [M+H]$^+$: 505.1525. found: 505.1515.

1,3-Dioxoisoindolin-2-yl 3-(N-(tert-butyl)-4'-methoxy-[1,1'-biphenyl]-2-carboxamido)propanoate (1w)

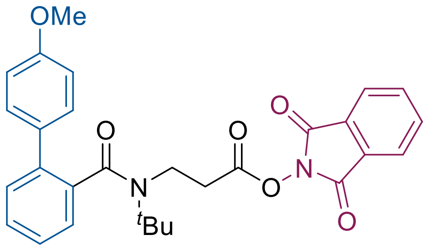

The title compound was prepared through general procedure **A-B,** general procedure **E** and general procedure **C-D** and purified by recrystallization from petroleum ether /ethyl acetate (v/v, = 6/1) as white solid (3.25 g, 65%).

M.p. 127.5 - 129.8 ℃.

^{1}H NMR (400 MHz, CDCl$_3$) δ 7.86 (dd, J = 5.5, 3.1 Hz, 2H), 7.78 (dd, J = 5.5, 3.1 Hz, 2H), 7.52 (d, J = 8.7 Hz, 2H), 7.45 – 7.36 (m, 3H), 7.35 – 7.21 (m, 1H), 7.01 – 6.93 (m, 2H), 3.86 (s, 3H), 3.37 (t, J = 8.2 Hz, 2H), 2.61 – 2.48 (m, 1H), 2.46 – 2.32 (m, 1H), 1.42 (s, 9H).

^{13}C NMR (101 MHz, CDCl$_3$) δ 172.5, 166.9, 161.6, 159.3, 137.8, 137.1, 134.9, 132.3, 130.5, 129.8, 129.0, 128.8, 127.4, 126.7, 124.0, 113.9, 57.5, 55.3, 41.6, 32.8, 28.7.

IR (KBr) 3670, 2965, 2833, 1743, 1622, 1517, 1475, 1365, 1080, 966, 878, 838, 773, 523 cm^{-1}.

HRMS (ESI-MS) *m/z* calcd for $C_{29}H_{28}N_2O_6$ [M+H]$^+$: 501.2020. found: 501.2012.

1,3-Dioxoisoindolin-2-yl 3-(*N*-(tert-butyl)-3',5'-dimethyl-[1,1'-biphenyl]-2-carboxamido)propanoate (1x)

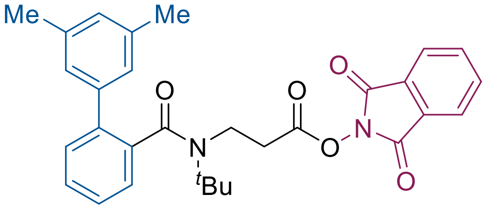

The title compound was prepared through general procedure **A-B,** general procedure **E** and general procedure **C-D** and purified by recrystallization from petroleum ether /ethyl acetate (v/v, = 6/1) as white solid (3.09 g, 62%).

M.p. 166.5 - 168.3 ℃.

^{1}H NMR (400 MHz, CDCl$_3$) δ 7.85 (dd, J = 5.5, 3.2 Hz, 2H), 7.77 (dd, J = 5.5, 3.1 Hz, 2H), 7.44 – 7.37 (m, 3H), 7.36 – 7.31 (m, 1H), 7.22 (s, 2H), 7.01 (s, 1H), 3.47 – 3.25 (m, 2H), 2.61 – 2.41 (m, 2H), 2.35 (s, 6H), 1.42 (s, 9H).

^{13}C NMR (101 MHz, CDCl$_3$) δ 172.4, 166.9, 161.6, 139.7, 138.0, 137.7, 137.7, 134.9, 129.9, 129.4, 129.0, 128.8, 127.6, 127.0, 126.7, 124.0, 57.4, 41.7, 32.7, 28.6, 21.3.

IR (KBr) 3678, 2917, 1744, 1467, 1288, 1194, 1023, 966, 874, 791, 516 cm^{-1}.

HRMS (ESI-MS) *m/z* calcd for $C_{30}H_{30}N_2O_5$ [M+H]$^+$: 499.2227. found: 499.2219.

1,3-Dioxoisoindolin-2-yl 3-(*N*-(tert-butyl)-3',5'-difluoro-[1,1'-biphenyl]-2-carboxamido)propanoate (1y)

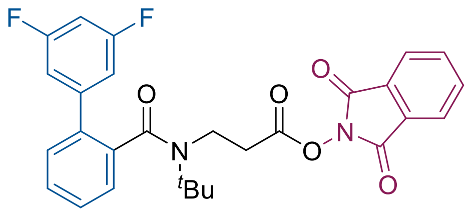

The title compound was prepared through general procedure **A-B,** general procedure **E** and general procedure **C-D** and purified by recrystallization from petroleum ether /ethyl acetate (v/v, = 6/1) as white solid (3.60 g, 71%).

M.p. 122.5 - 124.9 ℃.

^{1}H NMR (400 MHz, $CDCl_3$) δ 7.85 (dd, J = 5.5, 3.1 Hz, 2H), 7.78 (dd, J = 5.6, 3.1 Hz, 2H), 7.50 – 7.43 (m, 2H), 7.42 – 7.36 (m, 2H), 7.14 (dd, J = 8.4, 2.1 Hz, 2H), 6.82 (tt, J = 8.9, 2.3 Hz, 1H), 3.42 – 3.34 (m, 2H), 2.65 – 2.56 (m, 2H), 1.43 (s, 9H).

^{13}C NMR (101 MHz, $CDCl_3$) δ 171.59, 166.68, 162.79 (dd, J = 249.1, 12.8 Hz), 161.55, 142.82 (t, J = 9.8 Hz), 137.63, 135.16, 134.89, 129.66, 129.29, 128.84, 128.71, 126.76, 124.01, 112.2 (dd, J = 18.7 Hz, 7.2Hz), 103.10 (t, J = 25.2 Hz), 57.69, 41.65, 32.94, 28.57.

^{19}F NMR (376 MHz, $CDCl_3$) δ -109.3.

IR (KBr) 3671, 2977, 1744, 1646, 1427, 1333, 1193, 1125, 1081, 1023, 965, 877, 738, 698, 515.

HRMS (ESI-MS) *m/z* calcd for $C_{28}H_{24}F_2N_2O_5$ $[M+H]^+$: 507.1726. found: 507.1718.

1,3-Dioxoisoindolin-2-yl 3-(*N*-(tert-butyl)-3-fluorobenzamido)propanoate (1z)

The title compound was prepared through general procedure **A-B,** general procedure **E** and general procedure **C-D** and purified by recrystallization from petroleum ether /ethyl acetate (v/v, = 6/1) as white solid (3.09 g, 75%).

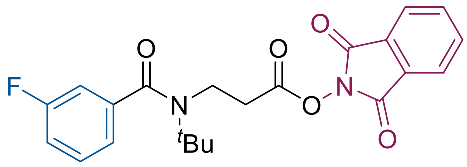

M.p. 101.4 - 103.4 ℃.

^{1}H NMR (400 MHz, $CDCl_3$) δ 7.87 (dd, J = 5.5, 3.1 Hz, 2H), 7.79 (dd, J = 5.5, 3.1 Hz, 2H), 7.41 (tdd, J = 7.5, 5.7, 1.3 Hz, 1H), 7.18 – 7.06 (m, 3H), 3.79 – 3.73 (m, 2H), 2.92 – 2.84 (m, 2H), 1.58 (s, 9H).

^{13}C NMR (101 MHz, $CDCl_3$) δ 172.0 (d, J = 2.1 Hz), 166.9, 162.7 (d, J = 248.4 Hz), 161.6, 140.9 (d, J = 6.6 Hz), 134.9, 130.6 (d, J = 8.0 Hz), 128.8, 124.0, 121.7 (d, J = 3.0 Hz), 116.4 (d, J = 20.9 Hz), 113.5 (d, J = 22.7 Hz), 57.7, 42.3, 33.1, 29.0.

^{19}F NMR (376 MHz, $CDCl_3$) δ -111.4.

IR (KBr) 3523, 3065, 2978, 2786, 1746, 1648, 1584, 1438, 1396, 1269, 1192, 1134, 1080, 1032, 967, 920, 880, 842, 792, 738, 700, 643, 593, 519, 458 cm^{-1}.

HRMS (ESI-MS) *m/z* calcd for $C_{22}H_{21}FN_2O_5$ $[M+H]^+$: 413.1507. found: 413.1498.

1,3-Dioxoisoindolin-2-yl 3-(*N*-(tert-butyl)-4-fluorobenzamido)propanoate (1aa)

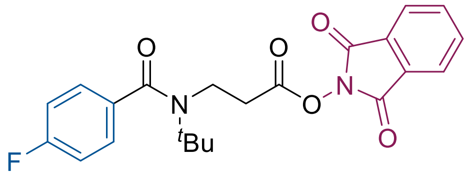

The title compound was prepared through general procedure **A-D** and purified by recrystallization from petroleum ether /ethyl acetate (v/v, = 6/1) as white solid (3.09 g, 75%).

M.p. 130.1 - 132.3 ℃.

^{1}H NMR (400 MHz, CDCl$_3$) δ 7.87 (dd, J = 5.5, 3.1 Hz, 2H), 7.79 (dd, J = 5.5, 3.1 Hz, 2H), 7.44 – 7.34 (m, 2H), 7.11 (t, J = 8.6 Hz, 2H), 3.81 – 3.73 (m, 2H), 2.92 – 2.84 (m, 2H), 1.57 (s, 9H).

^{13}C NMR (101 MHz, CDCl$_3$) δ 172.8, 166.9, 163.1 (d, J = 249.1 Hz), 161.6, 135.1 (d, J = 3.4 Hz), 134.9, 128.7, 128.4 (d, J = 8.4 Hz), 124.1, 115.8 (d, J = 21.7 Hz), 57.6, 42.4, 33.1, 29.0.

^{19}F NMR (376 MHz, CDCl$_3$) δ -110.8.

IR (KBr) 3518, 2971, 1747, 1648, 1398, 1193, 1080, 1029, 970, 878, 848, 700, 593, 496 cm^{-1}.

HRMS (ESI-MS) *m/z* calcd for $C_{22}H_{21}FN_2O_5$ [M+H]$^+$: 413.1507. found: 413.1499.

1,3-Dioxoisoindolin-2-yl 3-(*N*-(tert-butyl)-4-methoxybenzamido)propanoate (1ab)

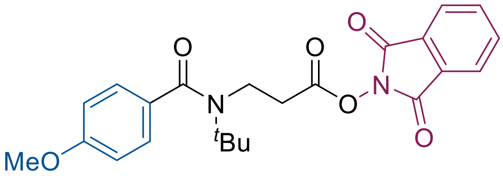

The title compound was prepared through general procedure **A-D** and purified by recrystallization from petroleum ether /ethyl acetate (v/v, = 6/1) as white solid (3.39 g, 80%). The NMR data is consistent with reported literature data[2].

M.p. 105.9 - 107.7 ℃.

IR (KBr) 2934, 2832, 1788, 1746, 1612, 1364, 1251, 1183, 1081, 1029, 968, 879, 842, 775, 701, 520 cm^{-1}.

HRMS (ESI-MS) *m/z* calcd for $C_{23}H_{24}N_2O_6$ [M+H]$^+$: 425.1707. found: 425.1697.

1,3-Dioxoisoindolin-2-yl 3-(*N*-isopropylbenzamido)propanoate (1ac)

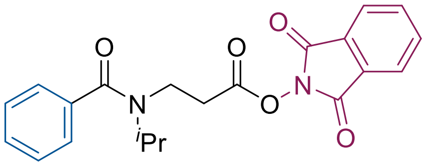

The title compound was prepared through general procedure **A-D** and purified by recrystallization from petroleum ether /ethyl acetate (v/v, = 6/1) as white solid (2.74 g, 72%).

M.p. 109.5 - 111.3 ℃.

^{1}H NMR (400 MHz, CDCl$_3$) δ 7.85 (dd, J = 5.5, 3.2 Hz, 2H), 7.76 (dd, J = 5.5, 3.1 Hz, 2H), 7.40 (s, 5H), 4.11-4.03 (m, 1H), 3.72 (t, J = 7.3 Hz, 2H), 3.16 (s, 2H), 1.16 (d, J = 6.8 Hz, 6H).

^{13}C NMR (101 MHz, CDCl$_3$) δ 172.3, 168.2, 161.7, 136.8, 134.8, 129.5, 128.8, 128.6, 126.3, 124.0, 50.6, 36.4, 30.6, 20.9.

IR (KBr) 3520, 2980, 1787, 1745, 1628, 1420, 1369, 1185, 1136, 1080, 1022, 968, 878, 786, 737, 701, 620, 518 cm^{-1}.

HRMS (ESI-MS) *m/z* calcd for $C_{21}H_{20}N_2O_5$ [M+H]$^+$: 381.1445. found: 381.1436.

1,3-Dioxoisoindolin-2-yl 3-(*N*-((3r)-adamantan-1-yl)benzamido)propanoate (1ad)

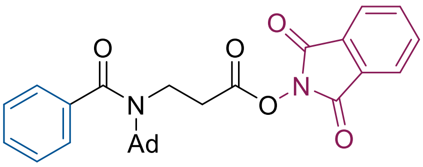

The title compound was prepared through general procedure **A-D** and purified by recrystallization from petroleum ether /ethyl acetate (v/v, = 6/1) as white solid (3.40 g, 72%).

M.p. 171.7 - 173.0 ℃.

^{1}H NMR (500 MHz, CDCl$_3$) δ 7.85 (dd, J = 5.4, 3.2 Hz, 2H), 7.77 (dd, J = 5.4, 3.2 Hz, 2H), 7.43 – 7.32 (m, 5H), 3.77 – 3.71 (m, 2H), 2.89 – 2.82 (m, 2H), 2.29 (s, 6H), 2.17 (s, 3H), 1.82 – 1.67 (m, 6H).

^{13}C NMR (126 MHz, CDCl$_3$) δ 173.7, 167.0, 161.7, 139.3, 134.9, 129.2, 128.8, 128.7, 126.0, 124.0, 59.0, 41.1, 40.4, 36.3, 33.7, 30.1.

IR (KBr) 3619, 2911, 2855, 1788, 1746, 1640, 1463, 1366, 1271, 1137, 1079, 1022, 967, 879, 785, 736, 701, 517 cm^{-1}.

HRMS (ESI-MS) *m/z* calcd for $C_{28}H_{28}N_2O_5$ [M+H]$^+$: 473.2071. found: 473.2063.

Tert-butyl 2-(*N*-(3-((1,3-dioxoisoindolin-2-yl)oxy)-3-oxopropyl)benzamido)-2-methylpropanoate (1ae)

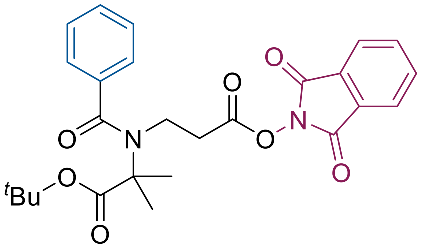

The title compound was prepared through general procedure **A-D** and purified by recrystallization from petroleum ether /ethyl acetate (v/v, = 6/1) as white solid (3.36 g, 70%).

M.p. 139.2 - 140.1 ℃.

^{1}H NMR (400 MHz, CDCl$_3$) δ 7.87 (dd, J = 5.5, 3.1 Hz, 2H), 7.80 (dd, J = 5.5, 3.1 Hz, 2H), 7.45 (dd, J = 5.2, 1.8 Hz, 3H), 7.40 – 7.35 (m, 2H), 3.82 – 3.75 (m, 2H), 2.97 – 2.91 (m, 2H), 1.62 (s, 6H), 1.52 (s, 9H).

^{13}C NMR (101 MHz, CDCl$_3$) δ 172.8, 172.0, 167.0, 161.6, 136.8, 134.9, 129.7, 128.9, 128.7, 125.9, 124.1, 81.0, 62.0, 40.9, 32.9, 27.9, 24.2.

IR (KBr) 3649, 2980, 2832, 1744, 1628, 1473, 1365, 1288, 1144, 1081, 1022, 966, 880, 848, 779, 737, 701, 516 cm^{-1}.

HRMS (ESI-MS) *m/z* calcd for $C_{26}H_{28}N_2O_7$ [M+H]$^+$: 481.1969. found: 481.1959.

1,3-Dioxoisoindolin-2-yl 3-(*N*-(tert-butyl)benzamido)-2-methylpropanoate (1af)

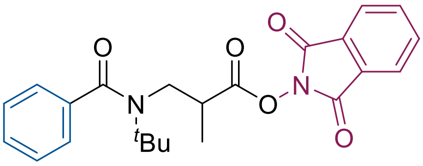

The title compound was prepared through general procedure **A-D** and purified by recrystallization from petroleum ether /ethyl acetate (v/v, = 6/1) as white solid (3.19 g, 78%).

M.p. 147.5 - 149.0 ℃.

^{1}H NMR (400 MHz, CDCl$_3$) δ 7.88 (dd, J = 5.5, 3.1 Hz, 2H), 7.80 (dd, J = 5.5, 3.1 Hz, 2H), 7.44 – 7.35 (m, 5H), 3.97 (dd, J = 15.1, 5.2 Hz, 1H), 3.74 (dd, J = 15.1, 8.7 Hz, 1H), 3.24 – 3.14 (m, 1H), 1.61 (s, 9H), 1.22 (d, J = 7.0 Hz, 3H).

^{13}C NMR (101 MHz, CDCl$_3$) δ 174.4, 170.7, 161.7, 138.9, 134.8, 129.6, 128.8, 128.5, 127.6, 124.0, 57.4, 49.8, 38.8, 29.1, 14.4.

IR (KBr) 3670, 2979, 1745, 1628, 1464, 1367, 1263, 1195, 1030, 965, 878, 837, 793, 701, 647, 602, 518 cm^{-1}.

HRMS (ESI-MS) *m/z* calcd for $C_{23}H_{24}N_2O_5$ [M+H]$^+$: 409.1758. found: 409.1750.

1,3-Dioxoisoindolin-2-yl 3-(*N*-(tert-butyl)-1-naphthamido)propanoate (1ag)

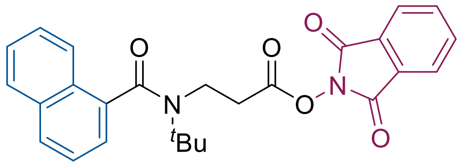

The title compound was prepared through general procedure **A-D** and purified by recrystallization from petroleum ether /ethyl acetate (v/v, = 6/1) as white solid (3.33 g, 75%).

M.p. 121.2 - 123.8 ℃.

^{1}H NMR (400 MHz, CDCl$_3$) *δ* 7.85 (dd, *J* = 7.4, 4.3 Hz, 3H), 7.77 (dd, *J* = 5.5, 3.1 Hz, 2H), 7.69 (dd, *J* = 5.5, 3.1 Hz, 2H), 7.51 (q, *J* = 8.2, 7.6 Hz, 3H), 7.43 (d, *J* = 6.9 Hz, 1H), 3.76 (dt, *J* = 15.3, 7.6 Hz, 1H), 3.54 (dt, *J* = 15.9, 8.0 Hz, 1H), 2.83 (t, *J* = 7.8 Hz, 2H), 1.69 (s, 9H).

^{13}C NMR (101 MHz, CDCl$_3$) *δ* 172.1, 166.8, 161.6, 136.4, 134.9, 133.6, 129.3, 129.0, 128.6, 128.6, 127.2, 126.5, 125.2, 124.3, 123.9, 122.9, 58.1, 42.1, 33.5, 29.1.

IR (KBr) 3668, 2974, 2832, 1744, 1628, 1466, 1366, 1191, 1082, 967, 878, 780, 737, 701, 519 cm^{-1}.

HRMS (ESI-MS) *m/z* calcd for $C_{26}H_{24}N_2O_5$ [M+H]$^+$: 445.1758. found: 445.1748.

1,3-Dioxoisoindolin-2-yl 3-(*N*-(tert-butyl)quinoline-8-carboxamido)propanoate (1ah)

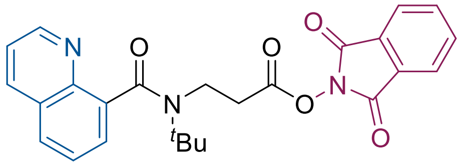

The title compound was prepared through general procedure **A-D** and purified by recrystallization from petroleum ether /ethyl acetate (v/v, = 2/1) as white solid (1.78 g, 40%).

M.p. 192.2 - 194.7 ℃.

^{1}H NMR (400 MHz, CDCl$_3$) *δ* 8.97 (dd, *J* = 4.4, 1.7 Hz, 1H), 8.15 (dd, *J* = 8.3, 1.8 Hz, 1H), 7.86 – 7.77 (m, 3H), 7.74 (dd, *J* = 6.8, 2.9 Hz, 2H), 7.64 – 7.54 (m, 2H), 7.43 (dd, *J* = 8.4, 4.2 Hz, 1H), 3.80 – 3.55 (m, 2H), 3.18 – 3.02 (m, 1H), 2.95 – 2.81 (m, 1H), 1.71 (s, 9H).

^{13}C NMR (101 MHz, CDCl$_3$) *δ* 171.7, 167.1, 161.5, 151.1, 144.6, 138.7, 136.1, 134.8, 128.7, 128.5, 128.4, 126.2, 126.1, 123.9, 121.8, 57.8, 42.4, 33.9, 29.1.

IR (KBr) 3680, 2832, 1742, 1622, 1516, 1365, 774 cm^{-1}.

HRMS (ESI-MS) *m/z* calcd for $C_{25}H_{23}N_3O_5$ [M+H]$^+$: 446.1710. found: 446.1701.

1,3-Dioxoisoindolin-2-yl 3-(N-(tert-butyl)-3-methylbenzamido)propanoate (1ai)

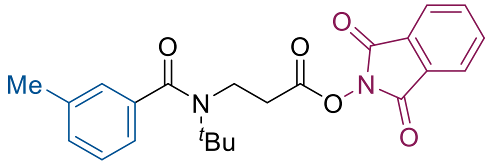

The title compound was prepared through general procedure **A-D** and purified by recrystallization from petroleum ether /ethyl acetate (v/v, = 6/1) as white solid (2.98 g, 73%).

M.p. 96.5 - 98.5 ℃.

^{1}H NMR (500 MHz, CDCl$_3$) *δ* 7.85 (dd, *J* = 5.5, 3.2 Hz, 2H), 7.77 (dd, *J* = 5.5, 3.1 Hz, 2H), 7.28 (t, *J* = 7.6 Hz, 1H), 7.19 (d, *J* = 7.7 Hz, 1H), 7.17 – 7.11 (m, 2H), 3.79 – 3.69 (m, 2H), 2.90 – 2.81 (m, 2H), 2.37 (s, 3H), 1.57 (s, 9H).

^{13}C NMR (126 MHz, CDCl$_3$) *δ* 173.8, 167.0, 161.6, 138.9, 138.6, 134.9, 130.0, 128.8, 128.6, 126.6, 124.0, 122.9, 57.4, 42.3, 33.2, 29.0, 21.4.

IR (KBr) 3651, 2975, 1746, 1652, 1396, 1192, 1033, 970, 879, 843, 795, 738, 701, 517, 493, 441 cm^{-1}.

HRMS (ESI-MS) *m/z* calcd for $C_{23}H_{24}N_2O_5$ [M+H]$^+$: 409.1758. found: 405.1749.

1,3-Dioxoisoindolin-2-yl 3-(*N*-(tert-butyl)-3-methoxybenzamido)propanoate (1aj)

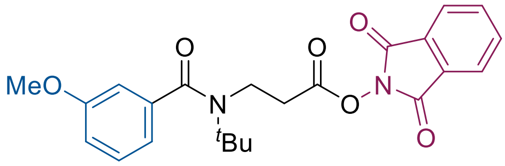

The title compound was prepared through general procedure **A-D** and purified by recrystallization from petroleum ether /ethyl acetate (v/v, = 6/1) as white solid (2.97 g, 70%).

M.p. 118.8 - 120.8 ℃.

^{1}H NMR (400 MHz, CDCl$_3$) δ 7.86 (dd, J = 5.5, 3.1 Hz, 2H), 7.79 (dd, J = 5.6, 3.1 Hz, 2H), 7.33 (t, J = 7.9 Hz, 1H), 6.98 – 6.86 (m, 3H), 3.87 – 3.71 (m, 5H), 2.93 – 2.85 (m, 2H), 1.58 (s, 9H).

^{13}C NMR (101 MHz, CDCl$_3$) δ 173.3, 167.0, 161.6, 159.8, 140.2, 134.9, 129.9, 128.8, 124.0, 118.1, 115.2, 111.5, 57.5, 55.4, 42.3, 33.3, 29.0.

IR (KBr) 3648, 2972, 1788, 1745, 1647, 1462, 1396, 1289, 1191, 1135, 1079, 1031, 967, 879, 791, 737, 701, 521 cm^{-1}.

HRMS (ESI-MS) *m/z* calcd for $C_{23}H_{24}N_2O_6$ [M+H]$^+$: 425.1707. found: 425.1698.

1,3-Dioxoisoindolin-2-yl 3-(*N*-(tert-butyl)-2-phenoxybenzamido)propanoate (1ak)

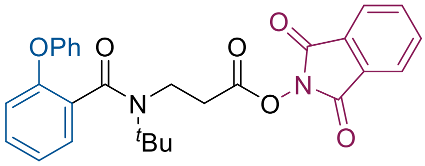

The title compound was prepared through general procedure **A-D** and purified by recrystallization from petroleum ether /ethyl acetate (v/v, = 6/1) as white solid (3.70 g, 76%).

M.p. 105.6 - 107.9 ℃.

^{1}H NMR (400 MHz, CDCl$_3$) δ 7.87 (dd, J = 5.5, 3.1 Hz, 2H), 7.79 (dd, J = 5.5, 3.1 Hz, 2H), 7.38 – 7.26 (m, 4H), 7.20 – 7.06 (m, 4H), 6.92 (d, J = 8.2 Hz, 1H), 3.86 – 3.77 (m, 2H), 3.05 (dt, J = 15.2, 8.2 Hz, 1H), 2.86 (dt, J = 16.8, 8.2 Hz, 1H), 1.54 (s, 9H).

^{13}C NMR (101 MHz, CDCl$_3$) δ 169.7, 167.1, 161.6, 156.7, 152.3, 134.9, 130.9, 130.1, 129.8, 128.8, 127.7, 124.0, 123.9, 123.6, 119.0, 118.8, 57.6, 42.1, 33.2, 28.9.

IR (KBr) 3729, 2832, 1601, 1363, 1080, 775, 521 cm^{-1}.

HRMS (ESI-MS) *m/z* calcd for $C_{28}H_{26}N_2O_6$ [M+H]$^+$: 487.1864. found: 487.1857.

1,3-Dioxoisoindolin-2-yl 3-(*N*-(tert-butyl)-2-methoxybenzamido)propanoate (1al)

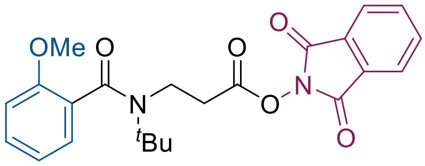

The title compound was prepared through general procedure **A-D** and purified by recrystallization from petroleum ether /ethyl acetate (v/v, = 6/1) as white solid (2.42 g, 57%).

M.p. 115.6 - 117.6 ℃.

^{1}H NMR (400 MHz, CDCl$_3$) δ 7.86 (dd, J = 5.5, 3.1 Hz, 2H), 7.79 (dd, J = 5.5, 3.1 Hz, 2H), 7.27 (ddd, J = 8.9, 7.5, 1.7 Hz, 1H), 7.16 (dd, J = 7.4, 1.7 Hz, 1H), 6.96 (td, J = 7.5, 0.9 Hz, 1H), 6.91 (d, J = 8.4 Hz, 1H), 3.88 (s, 3H), 3.72 (td, J = 10.7, 5.3 Hz, 1H), 3.66 – 3.57 (m, 1H), 3.01 – 2.89 (m, 1H), 2.84 (ddd, J = 16.5, 10.5, 6.0 Hz, 1H), 1.59 (s, 9H).

^{13}C NMR (101 MHz, CDCl$_3$) δ 170.9, 167.2, 161.7, 154.6, 134.9, 133.8, 130.1, 128.8, 126.8, 124.0, 121.0, 111.3, 57.7, 55.6, 42.0, 33.1, 29.0.

IR (KBr) 3699, 2833, 1602, 1363, 1080, 879, 774, 519 cm^{-1}.
HRMS (ESI-MS) *m/z* calcd for $C_{23}H_{24}N_2O_6$ $[M+H]^+$: 425.1707. found: 425.1700.

1,3-Dioxoisoindolin-2-yl 3-(*N*-(tert-butyl)furan-2-carboxamido)propanoate (1am)

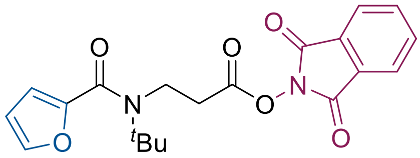

The title compound was prepared through general procedure **A-D** and purified by recrystallization from petroleum ether /ethyl acetate (v/v, = 6/1) as white solid (3.08 g, 80%).

M.p. 134.9 - 136.4 ℃.
^{1}H NMR (400 MHz, $CDCl_3$) δ 7.90 (dd, J = 5.7, 2.8 Hz, 2H), 7.81 (q, J = 2.7 Hz, 2H), 7.62 (s, 1H), 7.08 (t, J = 2.8 Hz, 1H), 6.47 (d, J = 2.5 Hz, 1H), 3.91 – 3.83 (m, 2H), 3.25 (t, J = 8.0 Hz, 2H), 1.54 (s, 9H).
^{13}C NMR (101 MHz, $CDCl_3$) δ 167.9, 162.2, 161.9, 149.7, 144.1, 134.9, 128.8, 124.0, 116.9, 111.4, 58.2, 41.2, 34.8, 28.4.
IR (KBr) 3677, 1742, 1639, 1478, 1375, 1293, 1181, 962, 783, 698, 515 cm^{-1}.
HRMS (ESI-MS) *m/z* calcd for $C_{20}H_{20}N_2O_6$ $[M+H]^+$: 385.1394. found: 385.1384.

1,3-Dioxoisoindolin-2-yl 3-(*N*-(tert-butyl)-3-methylfuran-2-carboxamido)propanoate (1an)

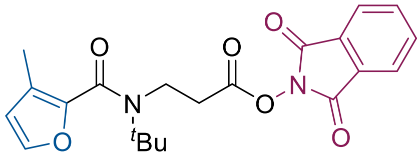

The title compound was prepared through general procedure **A-D** and purified by recrystallization from petroleum ether /ethyl acetate (v/v, = 6/1) as white solid (3.23 g, 82%).

M.p. 156.2 - 158.0 ℃.
^{1}H NMR (400 MHz, $CDCl_3$) δ 7.89 (dd, J = 5.5, 3.1 Hz, 2H), 7.80 (dd, J = 5.5, 3.1 Hz, 2H), 7.45 (d, J = 1.8 Hz, 1H), 6.31 (d, J = 1.7 Hz, 1H), 3.77 – 3.69 (m, 2H), 3.28 – 3.20 (m, 2H), 2.27 (s, 3H), 1.53 (s, 9H).
^{13}C NMR (101 MHz, $CDCl_3$) δ 168.1, 163.8, 161.9, 144.0, 142.2, 134.9, 128.8, 128.0, 124.0, 114.7, 57.7, 41.5, 34.9, 28.5, 11.4.
IR (KBr) 3678, 2831, 1743, 1621, 1366, 1187, 774 cm^{-1}.
HRMS (ESI-MS) *m/z* calcd for $C_{21}H_{22}N_2O_6$ $[M+H]^+$: 399.1551. found: 399.1543.

1,3-dioxoisoindolin-2-yl 4-(N-(tert-butyl)benzamido)butanoate (1ao)

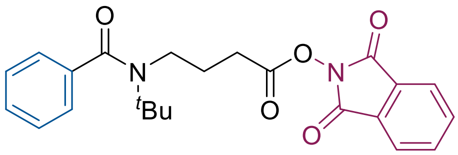

Ethyl 4-bromobutyrate (9.08 g, 46.54 mmol, 1 equiv) and tert-butylamine (10.21 g, 140 mmol, 3 equiv) were dissolved in 20 mL of cyclohexane and left for 2 days at 50 °C. Then water (40 *mL*) was added, the organic layer was separated and the aqueous layer was extracted with DCM (3 x 30 *mL*). The extracts were dried over Na_2SO_4 and concentrated to give 1.91 g (10.20 mmol, 22 %) of the product as colorless liquid. The title compound was then prepared through general procedure **A-D** and purified by recrystallization from petroleum ether /ethyl acetate (v/v, = 6/1) as white solid (2.45 g, 60%).

^{1}H NMR (500 MHz, CDCl$_3$) *δ* 7.88 (dd, *J* = 5.5, 3.1 Hz, 2H), 7.80 (dd, *J* = 5.5, 3.1 Hz, 2H), 7.44 – 7.31 (m, 5H), 3.55 – 3.12 (m, 2H), 2.38 (t, *J* = 7.2 Hz, 2H), 1.95 (dt, *J* = 15.0, 7.3 Hz, 2H), 1.57 (s, 9H).
^{13}C NMR (126 MHz, CDCl$_3$) *δ* 173.4, 168.7, 161.8, 139.4, 134.9, 128.9, 128.8, 128.6, 126.0, 124.0, 57.2, 46.4, 29.0, 28.4, 26.7.

1,3-dioxoisoindolin-2-yl 3-(N,4-di-tert-butylbenzamido)propanoate (1ap)

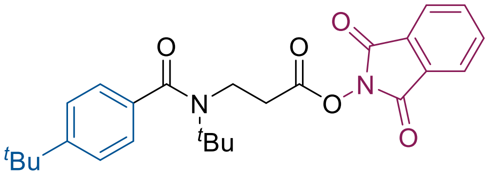

The title compound was prepared through general procedure **A-D** and purified by recrystallization from petroleum ether /ethyl acetate (v/v, = 6/1) as white solid (2.52 g, 56%).

1H NMR (400 MHz, CDCl$_3$) *δ* 7.87 (dd, *J* = 5.5, 3.2 Hz, 2H), 7.79 (dd, *J* = 5.5, 3.2 Hz, 2H), 7.43 (d, *J* = 8.2 Hz, 2H), 7.31 (d, *J* = 8.1 Hz, 2H), 3.78 (dd, *J* = 9.1, 6.2 Hz, 2H), 2.88 (dd, *J* = 8.9, 6.2 Hz, 2H), 1.58 (s, 9H), 1.33 (s, 9H).
^{13}C NMR (101 MHz, CDCl$_3$) *δ* 174.0, 167.1, 161.7, 152.5, 136.0, 134.9, 128.8, 126.0, 125.6, 124.0, 57.4, 42.3, 34.8, 33.2, 31.2, 29.0.

1,3-dioxoisoindolin-2-yl 3-(N-(tert-butyl)-4-(trifluoromethyl)benzamido)propanoate (1aq)

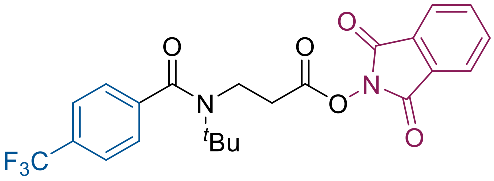

The title compound was prepared through general procedure **A-D** and purified by recrystallization from petroleum ether /ethyl acetate (v/v, = 6/1) as white solid (2.77 g, 60%).

^{1}H NMR (500 MHz, CDCl$_3$) *δ* 7.86 (dd, *J* = 5.5, 3.1 Hz, 2H), 7.78 (dd, *J* = 5.5, 3.1 Hz, 2H), 7.68 (d, *J* = 8.0 Hz, 2H), 7.49 (d, *J* = 7.9 Hz, 2H), 3.91 – 3.44 (m, 2H), 2.96 – 2.80 (m, 2H), 1.57 (s, 9H).
^{13}C NMR (126 MHz, CDCl$_3$) *δ* 172.1, 166.8, 161.6, 142.4, 134.9, 131.3 (q, *J* = 32.6 Hz), 128.7, 126.5, 125.9 (q, *J* = 3.8 Hz), 124.0, 123.8 (q, *J* = 272.3 Hz), 57.8, 42.2, 33.1, 29.0.
^{19}F NMR (471 MHz, CDCl$_3$) *δ* -62.81.

1,3-dioxoisoindolin-2-yl 3-(N-(tert-butyl)-4-(trifluoromethoxy)benzamido)propanoate (1ar)

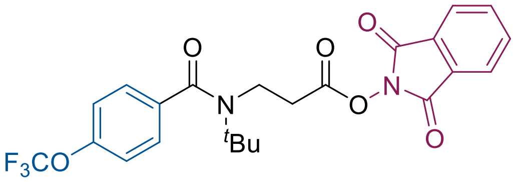

The title compound was prepared through general procedure **A-D** and purified by recrystallization from petroleum ether /ethyl acetate (v/v, = 6/1) as white solid (2.96 g, 62%).

^{1}H NMR (400 MHz, CDCl$_3$) *δ* 7.85 (dd, *J* = 5.5, 3.1 Hz, 2H), 7.78 (dd, *J* = 5.6, 3.1 Hz, 2H), 7.51 – 7.40 (m, 2H), 7.26 (d, *J* = 7.7 Hz, 2H), 3.83 – 3.70 (m, 2H), 2.97 – 2.81 (m, 2H), 1.56 (s, 9H).
^{13}C NMR (101 MHz, CDCl$_3$) *δ* 172.4, 166.9, 161.6, 149.7, 137.5, 134.9, 128.7, 128.0, 124.0, 121.1, 120.4 (q, *J* = 257.5 Hz), 57.6, 42.3, 33.1, 28.9.
^{19}F NMR (376 MHz, CDCl$_3$) *δ* -57.76.

1,3-Dioxoisoindolin-2-yl 3-(*N*-(tert-butyl)-4-methylbenzamido)propanoate (3a)

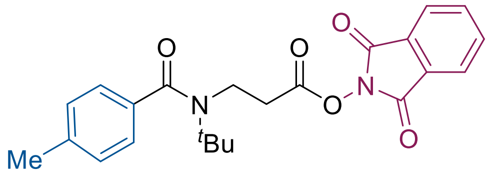

The title compound was prepared through general procedure **A-D** and purified by recrystallization from petroleum ether /ethyl acetate (v/v, = 6/1) as white solid (2.94 g, 72%).

M.p. 106.9 - 108.8 ℃.

^{1}H NMR (400 MHz, CDCl$_3$) δ 7.87 (dd, *J* = 5.4, 3.1 Hz, 2H), 7.79 (dd, *J* = 5.5, 3.1 Hz, 2H), 7.27 (d, *J* = 9.3 Hz, 2H), 7.21 (d, *J* = 7.9 Hz, 2H), 3.81 – 3.73 (m, 2H), 2.91 – 2.82 (m, 2H), 2.37 (s, 3H), 1.57 (s, 9H).

^{13}C NMR (101 MHz, CDCl$_3$) δ 173.9, 167.1, 161.7, 139.4, 136.1, 134.9, 129.3, 128.8, 126.1, 124.0, 57.4, 42.3, 33.2, 29.0, 21.4.

IR (KBr) 3677, 2928, 1746, 1636, 1399, 1191, 1080, 1025, 967, 879, 833, 701 cm^{-1}.

HRMS (ESI-MS) *m/z* calcd for $C_{23}H_{24}N_2O_5$ [M+H]$^+$: 409.1758. found: 409.1750.

1,3-Dioxoisoindolin-2-yl 3-(*N*-(tert-butyl)-3-fluoro-4-methylbenzamido)propanoate (3b)

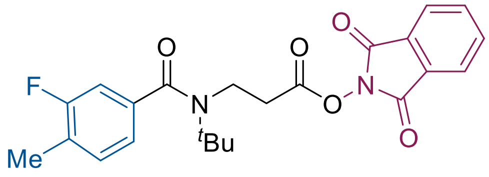

The title compound was prepared through general procedure **A-D** and purified by recrystallization from petroleum ether /ethyl acetate (v/v, = 6/1) as white solid (2.77 g, 65%).

M.p. 97.7 - 98.3 ℃.

^{1}H NMR (400 MHz, CDCl$_3$) δ 7.89 (dd, *J* = 5.5, 3.1 Hz, 2H), 7.81 (dd, *J* = 5.5, 3.1 Hz, 2H), 7.24 (t, *J* = 7.7 Hz, 1H), 7.09 – 7.01 (m, 2H), 3.82 – 3.74 (m, 2H), 2.92 – 2.83 (m, 2H), 2.31 (d, *J* = 1.9 Hz, 3H), 1.58 (s, 9H).

^{13}C NMR (101 MHz, CDCl$_3$) δ 172.4, 167.0, 161.6, 161.1 (d, *J* = 247.2 Hz), 138.2 (d, *J* = 6.9 Hz), 134.9, 131.9 (d, *J* = 5.2 Hz), 128.8, 126.4 (d, *J* = 17.3 Hz), 124.0, 121.6 (d, *J* = 3.4 Hz), 113.2 (d, *J* = 23.8 Hz), 57.6, 42.3, 33.1, 29.0, 14.5 (d, *J* = 3.6 Hz).

^{19}F NMR (376 MHz, CDCl$_3$) δ -116.0.

IR (KBr) 2979, 1788, 1746, 1647, 1575, 1388, 1345, 1191, 1079, 1034, 967, 879, 791, 701 cm^{-1}.

HRMS (ESI-MS) *m/z* calcd for $C_{23}H_{23}FN_2O_5$ [M+H]$^+$: 427.1664. found: 427.1657.

1,3-Dioxoisoindolin-2-yl 3-(*N*-(tert-butyl)-3-chloro-4-methylbenzamido)propanoate (3c)

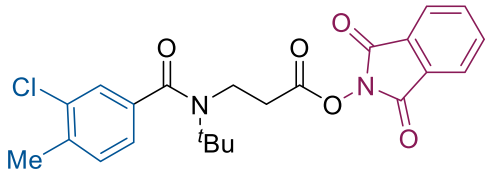

The title compound was prepared through general procedure **A-D** and purified by recrystallization from petroleum ether /ethyl acetate (v/v, = 6/1) as white solid (3.23 g, 73%).

M.p. 118.5 - 120.5 ℃.

^{1}H NMR (400 MHz, CDCl$_3$) δ 7.88 (dd, *J* = 5.5, 3.1 Hz, 2H), 7.80 (dd, *J* = 5.5, 3.1 Hz, 2H), 7.36 (d, *J* = 1.7 Hz, 1H), 7.28 (d, *J* = 7.8 Hz, 1H), 7.17 (dd, *J* = 7.7, 1.7 Hz, 1H), 3.82 – 3.74 (m, 2H), 2.90 – 2.83 (m, 2H), 2.40 (s, 3H), 1.57 (s, 9H).

^{13}C NMR (101 MHz, CDCl$_3$) δ 172.2, 167.0, 161.6, 138.0, 137.5, 134.9, 134.7, 131.2, 128.8, 126.9, 124.4, 124.0, 57.6, 42.3, 33.1, 29.0, 20.0.

IR (KBr) 3650, 2976, 1788, 1745, 1631, 1466, 1366, 1193, 1134, 1081, 967, 881, 835, 775, 736, 698, 593, 517 cm^{-1}.
HRMS (ESI-MS) *m/z* calcd for $C_{23}H_{23}ClN_2O_5$ $[M+H]^+$: 443.1368. found: 443.1362.

1,3-Dioxoisoindolin-2-yl 3-(*N*-(tert-butyl)-3-methoxy-4-methylbenzamido)propanoate (3d)

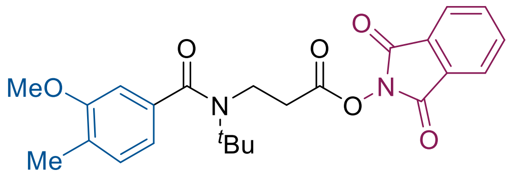

The title compound was prepared through general procedure **A-D** and purified by recrystallization from petroleum ether /ethyl acetate (v/v, = 6/1) as white solid (3.17 g, 73%).

M.p. 87.8 - 89.1 ℃.
^{1}H NMR (400 MHz, $CDCl_3$) δ 7.88 (dd, J = 5.5, 3.1 Hz, 2H), 7.80 (dd, J = 5.6, 3.1 Hz, 2H), 7.16 (d, J = 8.2 Hz, 1H), 6.85 (dd, J = 5.6, 1.8 Hz, 2H), 3.87 (s, 3H), 3.82 – 3.76 (m, 2H), 2.94 – 2.86 (m, 2H), 2.24 (s, 3H), 1.59 (s, 9H).
^{13}C NMR (101 MHz, $CDCl_3$) δ 173.9, 167.1, 161.7, 157.9, 137.6, 134.9, 130.6, 128.8, 128.3, 124.0, 117.8, 108.0, 57.4, 55.4, 42.4, 33.4, 29.0, 16.2.
IR (KBr) 3648, 2970, 1788, 1746, 1640, 1464, 1405, 1266, 1190, 1134, 1080, 1036, 967, 876, 736, 701, 518 cm^{-1}.
HRMS (ESI-MS) *m/z* calcd for $C_{24}H_{26}N_2O_6$ $[M+H]^+$: 439.1864. found: 439.1857.

1,3-Dioxoisoindolin-2-yl 3-(*N*-(tert-butyl)-3,4-dimethylbenzamido)propanoate (3e)

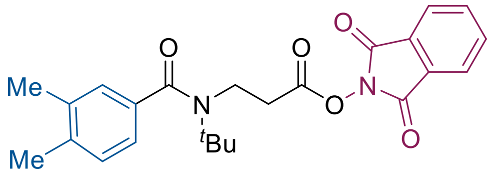

The title compound was prepared through general procedure **A-D** and purified by recrystallization from petroleum ether /ethyl acetate (v/v, = 6/1) as white solid (3.17 g, 75%).

M.p. 101.5 - 103.5 ℃.
^{1}H NMR (400 MHz, $CDCl_3$) δ 7.87 (dd, J = 5.5, 3.2 Hz, 2H), 7.80 (dd, J = 5.5, 3.1 Hz, 2H), 7.16 (d, J = 7.7 Hz, 1H), 7.14 (s, 1H), 7.09 (dd, J = 7.5, 1.8 Hz, 1H), 3.81 – 3.73 (m, 2H), 2.90 – 2.84 (m, 2H), 2.29 (s, 3H), 2.28 (s, 3H), 1.58 (s, 9H).
^{13}C NMR (101 MHz, $CDCl_3$) δ 174.1, 167.1, 161.7, 138.0, 137.1, 136.5, 134.9, 129.8, 128.8, 127.3, 124.0, 123.5, 57.4, 42.3, 33.2, 29.0, 19.7, 19.7.
IR (KBr) 3647, 2974, 1788, 1746, 1639, 1463, 1367, 1189, 1135, 1080, 1035, 967, 881, 831, 778, 736, 701, 592, 517 cm^{-1}.
HRMS (ESI-MS) *m/z* calcd for $C_{24}H_{26}N_2O_5$ $[M+H]^+$: 423.1914. found: 423.1907.

1,3-Dioxoisoindolin-2-yl 3-(*N*-(tert-butyl)-2-fluoro-4-methylbenzamido)propanoate (3f)

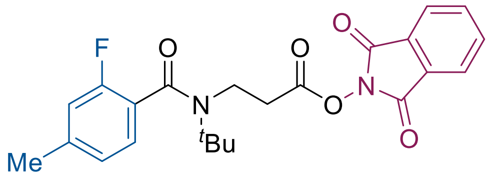

The title compound was prepared through general procedure **A-D** and purified by recrystallization from petroleum ether /ethyl acetate (v/v, = 6/1) as white solid (3.41 g, 80%).

M.p. 113.4 - 115.2 ℃.

^{1}H NMR (400 MHz, CDCl$_3$) δ 7.86 (dd, J = 5.5, 3.1 Hz, 2H), 7.79 (dd, J = 5.5, 3.1 Hz, 2H), 7.19 (t, J = 7.4 Hz, 1H), 7.01 (d, J = 7.7 Hz, 1H), 6.93 (d, J = 10.4 Hz, 1H), 3.78 – 3.65 (m, 2H), 2.86 (t, J = 7.7 Hz, 2H), 2.36 (s, 3H), 1.58 (s, 9H).
^{13}C NMR (101 MHz, CDCl$_3$) δ 168.2, 167.0, 161.7, 157.5 (d, J = 245.5 Hz), 141.7 (d, J = 7.7 Hz), 134.9, 128.7, 127.7 (d, J = 9.8 Hz), 125.5 (d, J = 10.8 Hz), 124.0, 123.9, 116.5 (d, J = 21.1 Hz), 57.9, 42.0, 32.9 (d, J = 6.3 Hz), 29.0 (d, J = 9.9 Hz), 21.3 (d, J = 12.1 Hz).
^{19}F NMR (376 MHz, CDCl$_3$) δ -117.5.
IR (KBr) 3650, 2976, 1788, 1746, 1627, 1465, 1366, 1193, 1141, 1079, 1027, 967, 877, 827, 781, 735, 701, 593, 520 cm^{-1}.
HRMS (ESI-MS) *m/z* calcd for $C_{23}H_{23}FN_2O_5$ [M+H]$^+$: 427.1664. found: 427.1656.

1,3-Dioxoisoindolin-2-yl 3-(*N*-(tert-butyl)-2-chloro-4-methylbenzamido)propanoate (3g)

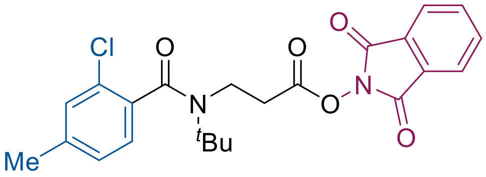

The title compound was prepared through general procedure **A-D** and purified by recrystallization from petroleum ether /ethyl acetate (v/v, = 6/1) as white solid (3.19 g, 72%).

M.p. 133.4 - 135.5 ℃.
^{1}H NMR (400 MHz, CDCl$_3$) δ 7.86 (dd, J = 5.5, 3.1 Hz, 2H), 7.78 (dd, J = 5.5, 3.1 Hz, 2H), 7.22 (s, 1H), 7.17 (d, J = 7.7 Hz, 1H), 7.13 (d, J = 7.7 Hz, 1H), 3.77 (ddd, J = 15.6, 10.1, 4.7 Hz, 1H), 3.59 (ddd, J = 15.9, 10.4, 5.7 Hz, 1H), 2.98 – 2.87 (m, 1H), 2.78 (ddd, J = 16.5, 10.4, 5.6 Hz, 1H), 2.34 (s, 3H), 1.59 (s, 9H).
^{13}C NMR (101 MHz, CDCl$_3$) δ 169.5, 167.0, 161.6, 140.4, 134.9, 130.4, 129.4, 128.7, 128.1, 127.0, 124.0, 58.0, 41.8, 32.9, 28.9, 21.1.
IR (KBr) 3667, 2976, 1787, 1746, 1642, 1466, 1366, 1192, 1135, 1081, 1022, 966, 880, 828, 779, 736, 700, 590, 516 cm^{-1}.
HRMS (ESI-MS) *m/z* calcd for $C_{23}H_{23}ClN_2O_5$ [M+H]$^+$: 443.1368. found: 443.1360.

1,3-Dioxoisoindolin-2-yl 3-(*N*-(tert-butyl)-2,4-dimethylbenzamido)propanoate (3h)

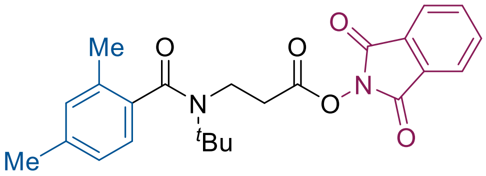

The title compound was prepared through general procedure **A-D** and purified by recrystallization from petroleum ether /ethyl acetate (v/v, = 6/1) as white solid (3.17 g, 75%).
M.p. 137.8 - 139.1 ℃.
^{1}H NMR (400 MHz, CDCl$_3$) δ 7.87 (dd, J = 5.5, 3.1 Hz, 2H), 7.79 (dd, J = 5.6, 3.1 Hz, 2H), 7.08 (d, J = 7.6 Hz, 1H), 7.04 (d, J = 8.7 Hz, 2H), 3.76-3.56 (m, 2H), 2.81 (t, J = 7.8 Hz, 2H), 2.33 (s, 3H), 2.29 (s, 3H), 1.60 (s, 9H).
^{13}C NMR (101 MHz, CDCl$_3$) δ 173.0, 167.0, 161.7, 138.5, 135.7, 134.9, 133.0, 131.4, 128.8, 126.7, 125.1, 124.0, 57.6, 41.7, 33.0, 29.1, 21.2, 18.8.
IR (KBr) 3677, 2831, 1745, 1627, 1366, 1193, 1079, 966, 879, 775, 701 cm^{-1}.
HRMS (ESI-MS) *m/z* calcd for $C_{24}H_{26}N_2O_5$ [M+H]$^+$: 423.1914. found: 423.1907.

1,3-Dioxoisoindolin-2-yl 3-(*N*-(tert-butyl)-4-ethylbenzamido)propanoate (3i)

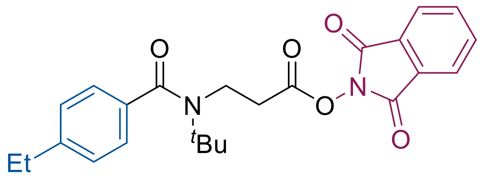

The title compound was prepared through general procedure **A-D** and purified by recrystallization from petroleum ether /ethyl acetate (v/v, = 6/1) as white solid (2.53 g, 60%).

M.p. 81.6 - 82.6 ℃.

^{1}H NMR (500 MHz, CDCl$_3$) δ 7.86 (dd, J = 5.5, 3.1 Hz, 2H), 7.78 (dd, J = 5.5, 3.1 Hz, 2H), 7.28 (d, J = 8.1 Hz, 2H), 7.22 (d, J = 7.9 Hz, 2H), 3.79 – 3.74 (m, 2H), 2.88 – 2.83 (m, 2H), 2.66 (q, J = 7.6 Hz, 2H), 1.56 (s, 9H), 1.23 (t, J = 7.6 Hz, 3H).

^{13}C NMR (126 MHz, CDCl$_3$) δ 174.0, 167.1, 161.6, 145.7, 136.3, 134.8, 128.8, 128.1, 126.2, 124.0, 57.4, 42.3, 33.2, 29.1, 28.7, 15.3.

IR (KBr) 3651, 2969, 1745, 1626, 1465, 1366, 1191, 1080, 1025, 967, 878, 842, 778, 701, 588, 515 cm^{-1}.

HRMS (ESI-MS) *m/z* calcd for $C_{24}H_{26}N_2O_5$ [M+H]$^+$: 423.1914. found: 423.1906.

1,3-Dioxoisoindolin-2-yl 3-(*N*-(tert-butyl)-4-isopropylbenzamido)propanoate (3j)

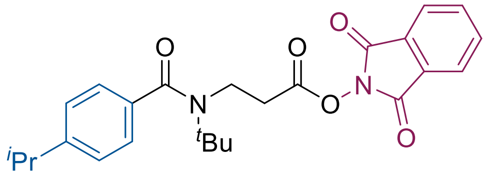

The title compound was prepared through general procedure **A-D** and purified by recrystallization from petroleum ether /ethyl acetate (v/v, = 6/1) as white solid (3.40 g, 78%).

M.p. 115.0 - 116.4 ℃.

^{1}H NMR (500 MHz, CDCl$_3$) δ 7.87 (dd, J = 5.5, 3.1 Hz, 2H), 7.80 (dd, J = 5.5, 3.1 Hz, 2H), 7.31 (d, J = 8.3 Hz, 2H), 7.27 (d, J = 8.3 Hz, 2H), 3.81 – 3.75 (m, 2H), 2.97 – 2.90 (m, 1H), 2.90 – 2.85 (m, 2H), 1.58 (s, 9H), 1.26 (d, J = 6.9 Hz, 6H).

^{13}C NMR (126 MHz, CDCl$_3$) δ 174.0, 167.1, 161.7, 150.3, 136.4, 134.9, 128.8, 126.8, 126.2, 124.0, 57.4, 42.3, 34.0, 33.2, 29.0, 23.8.

IR (KBr) 3667, 2964, 1745, 1630, 1465, 1366, 1192, 1080, 967, 879, 843, 778, 701, 591, 516 cm^{-1}.

HRMS (ESI-MS) *m/z* calcd for $C_{25}H_{28}N_2O_5$ [M+H]$^+$: 437.2071. found: 437.2065.

1,3-Dioxoisoindolin-2-yl 3-(*N*-(tert-butyl)-4-cyclohexylbenzamido)propanoate (3k)

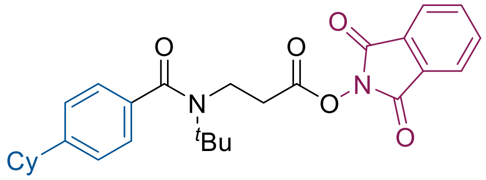

The title compound was prepared through general procedure **A-D** and purified by recrystallization from petroleum ether /ethyl acetate (v/v, = 6/1) as white solid (3.43 g, 72%).

M.p. 123.3 - 125.8 ℃.

^{1}H NMR (500 MHz, CDCl$_3$) δ 7.86 (dd, J = 5.5, 3.1 Hz, 2H), 7.78 (dd, J = 5.5, 3.1 Hz, 2H), 7.28 (d, J = 8.2 Hz, 2H), 7.23 (d, J = 8.2 Hz, 2H), 3.81 – 3.72 (m, 2H), 2.90 – 2.82 (m, 2H), 2.55 – 2.46 (m, 1H), 1.89 – 1.79 (m, 4H), 1.77 – 1.69 (m, 1H), 1.56 (s, 9H), 1.46 – 1.22 (m, 5H).

^{13}C NMR (126 MHz, CDCl$_3$) δ 174.0, 167.1, 161.7, 149.4, 136.4, 134.9, 128.8, 127.2, 126.2, 124.0, 57.4, 44.4, 42.3, 34.2, 33.2, 29.0, 26.8, 26.1.

IR (KBr) 3647, 2929, 2852, 1744, 1615, 1464, 1364, 1191, 1079, 1031, 967, 878, 837, 776, 736, 701, 580, 515 cm^{-1}.
HRMS (ESI-MS) *m/z* calcd for $C_{28}H_{32}N_2O_5$ $[M+H]^+$: 477.2384. found: 477.2378.

1,3-Dioxoisoindolin-2-yl 3-(4-(2-(tert-butoxy)-2-oxoethyl)-*N*-(tert-butyl)benzamido)propanoate (3l)

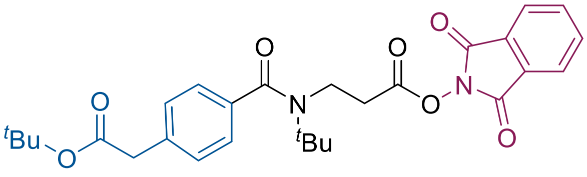

Add methyl 4-iodobenzoate (5 g, 19.1 mmol, 1 equiv), CuI (363.5 mg, 1.91 mmol, 0.1 equiv), Cs_2CO_3 (18.7 g, 57.3 mmol, 3 equiv) and 2-picolinic acid (466.0 mg, 7.6mmol, 0.2 equiv) into a 250 *mL* flask. Exchange the flask with Ar into a 250 *mL* flask. Add dioxane (50 *mL*) and tert-butyl 3-oxobutanoate (6.2 *mL*, 38.2 mmol, 2 equiv). Stir the resulting mixture for 72 hours at 70°C. Evaporate the solvent under reduced pressure and dilute the mixture with ethyl acetate (EtOAc) (75 *mL*). Wash the mixture with saturated NH_4Cl (75 *mL*). Dry the organic layer over anhydrous Na_2SO_4 and concentrate under vacuum. Purify the residue with silica gel column chromatography (petroleum ether/ethyl acetate from 100:0 to 70:30) to obtain methyl 4-(2-(tert-butoxy)-2-oxoethyl)benzoate. 0.1 M NaOH solution (1.1 equiv) was added to a solution of the obtained methyl benzoate in methanol (maintaining 1/1 methanol/water ratio). Reaction mixture was stirred at 65 ℃ for 8 h and methanol was evaporated. Aqueous solution in residue was acidified with 30 *mL* of 1 M HCl and stirred additionally for 10 min. Formed precipitate was collected, washed with water and dried in vacuo. Beige solid 4-(2-(tert-butoxy)-2-oxoethyl)benzoic acid was obtained (2.2 g, 9.32 mmol). The title compound was then prepared through general procedure **A-D** and purified by recrystallization from petroleum ether /ethyl acetate (v/v, = 6/1) as white solid (2.46 g, 52%).

M.p. 122.2 - 125.0 ℃.
^{1}H NMR (400 MHz, $CDCl_3$) δ 7.87 (dd, *J* = 5.5, 3.1 Hz, 2H), 7.79 (dd, *J* = 5.6, 3.1 Hz, 2H), 7.33 (s, 4H), 3.77 (t, *J* = 7.6 Hz, 2H), 3.54 (s, 2H), 2.91 – 2.83 (m, 2H), 1.57 (s, 9H), 1.40 (s, 9H).
^{13}C NMR (101 MHz, $CDCl_3$) δ 173.6, 170.3, 167.0, 161.6, 137.5, 136.1, 134.8, 129.5, 128.8, 126.3, 124.0, 81.0, 57.5, 42.6, 42.3, 33.2, 29.0, 28.0.
IR (KBr) 3646, 2976, 2832, 1788, 1745, 1625, 1466, 1364, 1141, 1080, 967, 877, 775, 701, 522 cm^{-1}.
HRMS (ESI-MS) *m/z* calcd for $C_{28}H_{32}N_2O_7$ $[M+H]^+$: 509.2282. found: 509.2276.

1,3-Dioxoisoindolin-2-yl 3-(*N*-(tert-butyl)-4-methylbenzamido)-2-methylpropanoate (3m)

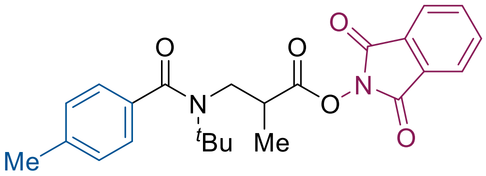

The title compound was prepared through general procedure **A-D** and purified by recrystallization from petroleum ether /ethyl acetate (v/v, = 6/1) as white solid (3.38 g, 80%).

M.p. 130.0 - 131.4 ℃.

^{1}H NMR (400 MHz, CDCl$_3$) δ 7.87 (dd, J = 5.5, 3.1 Hz, 2H), 7.79 (dd, J = 5.5, 3.1 Hz, 2H), 7.32 (d, J = 7.8 Hz, 2H), 7.18 (d, J = 7.8 Hz, 2H), 3.97 (dd, J = 15.1, 5.1 Hz, 1H), 3.76 (dd, J = 15.1, 8.9 Hz, 1H), 3.18 (ddd, J = 8.8, 7.0, 5.0 Hz, 1H), 2.36 (s, 3H), 1.60 (s, 9H), 1.22 (d, J = 7.0 Hz, 3H).

^{13}C NMR (101 MHz, CDCl$_3$) δ 174.6, 170.7, 161.8, 139.7, 136.0, 134.8, 129.1, 128.8, 127.6, 124.0, 57.2, 49.8, 38.8, 29.2, 21.4, 14.2.

IR (KBr) 3743, 3365, 2868, 2650, 1678, 1477, 1429, 1274, 1202, 1098, 1051, 896, 785, 750, 681, 613, 547, 423 cm^{-1}.

HRMS (ESI-MS) *m/z* calcd for $C_{24}H_{26}N_2O_5$ [M+H]$^+$: 423.1914. found: 423.1905.

1,3-Dioxoisoindolin-2-yl 3-(*N*-(tert-butyl)-4-methyl-1-naphthamido)propanoate (3n)

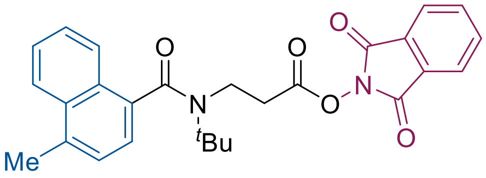

The title compound was prepared through general procedure **A-D** and purified by recrystallization from petroleum ether /ethyl acetate (v/v, = 6/1) as white solid (3.67 g, 80%).

M.p. 147.0 - 149.0 ℃.

^{1}H NMR (400 MHz, CDCl$_3$) δ 8.05 – 7.99 (m, 1H), 7.85 (dd, J = 6.4, 3.3 Hz, 1H), 7.78 (dd, J = 5.5, 3.1 Hz, 2H), 7.70 (dd, J = 5.5, 3.1 Hz, 2H), 7.55 (dd, J = 6.4, 3.3 Hz, 2H), 7.32 (t, J = 5.9 Hz, 2H), 3.77 (dt, J = 15.5, 6.8 Hz, 1H), 3.55 (dt, J = 15.9, 7.3 Hz, 1H), 2.82 (td, J = 7.6, 3.1 Hz, 2H), 2.70 (s, 3H), 1.70 (s, 9H).

^{13}C NMR (101 MHz, CDCl$_3$) δ 172.4, 166.9, 161.6, 135.6, 134.9, 134.9, 132.8, 129.4, 128.6, 126.8, 126.3, 125.9, 124.9, 124.8, 123.9, 122.7, 58.0, 42.1, 33.4, 29.1, 19.6.

IR (KBr) 3672, 2831, 1616, 1364, 1129, 773, 517 cm^{-1}.

HRMS (ESI-MS) *m/z* calcd for $C_{27}H_{26}N_2O_5$ [M+H]$^+$: 459.1914. found: 459.1907.

1,3-Dioxoisoindolin-2-yl 3-(N-(tert-butyl)-2-methylfuran-3-carboxamido)propanoate (3o)

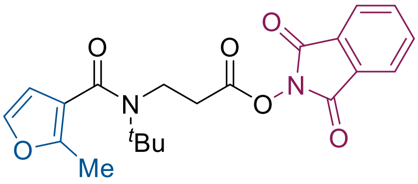

The title compound was prepared through general procedure **A-D** and purified by recrystallization from petroleum ether /ethyl acetate (v/v, = 6/1) as white solid (3.39 g, 85%).

M.p. 120.0 - 121.6 ℃.

^{1}H NMR (400 MHz, CDCl$_3$) δ 7.87 (dd, J = 5.5, 3.1 Hz, 2H), 7.79 (dd, J = 5.6, 3.2 Hz, 2H), 7.26 (d, J = 2.0 Hz, 1H), 6.35 (d, J = 2.0 Hz, 1H), 3.92 – 3.83 (m, 2H), 2.91 – 2.79 (m, 2H), 2.35 (s, 3H), 1.55 (s, 9H).

^{13}C NMR (101 MHz, CDCl$_3$) δ 168.3, 167.0, 161.7, 151.9, 140.7, 134.9, 128.8, 124.0, 118.6, 109.5, 57.4, 42.1, 33.3, 29.1, 12.6.

IR (KBr) 3776, 3666, 3320, 2970, 2929, 2833, 2718, 1784, 1745, 1603, 1464, 1411, 1363, 1188, 1131, 1078, 1018, 775, 700, 602, 519 cm^{-1}.

HRMS (ESI-MS) *m/z* calcd for $C_{21}H_{22}N_2O_6$ [M+H]$^+$: 399.1551. found: 399.1543.

1,3-Dioxoisoindolin-2-yl 3-(N-((3r)-adamantan-1-yl)-2-methylfuran-3-carboxamido)propanoate (3p)

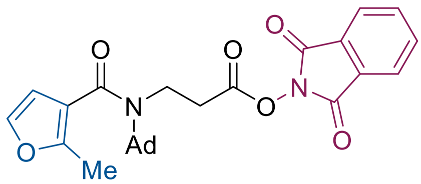

The title compound was prepared through general procedure **A-D** and purified by recrystallization from petroleum ether /ethyl acetate (v/v, = 6/1) as white solid (3.91 g, 82%).

M.p. 153.6 - 155.5 ℃.

^{1}H NMR (400 MHz, CDCl$_3$) δ 7.89 (dd, J = 5.5, 3.1 Hz, 2H), 7.81 (dd, J = 5.5, 3.1 Hz, 2H), 7.27 (d, J = 2.0 Hz, 1H), 6.35 (d, J = 2.0 Hz, 1H), 3.93 – 3.83 (m, 2H), 2.90 – 2.81 (m, 2H), 2.37 (s, 3H), 2.29 (d, J = 3.0 Hz, 6H), 2.18 (s, 3H), 1.75 (q, J = 12.7, 12.2 Hz, 6H).

^{13}C NMR (101 MHz, CDCl$_3$) δ 168.3, 167.0, 161.7, 151.7, 140.7, 134.9, 128.8, 124.0, 119.1, 109.6, 58.9, 41.0, 40.5, 36.4, 33.9, 30.1, 12.6.

IR (KBr) 3647, 2911, 2854, 1787, 1746, 1625, 1414, 1365, 1080, 1012, 969, 877, 840, 776, 737, 700, 603, 519. cm^{-1}.

HRMS (ESI-MS) *m/z* calcd for $C_{27}H_{28}N_2O_6$ [M+H]$^+$: 477.2020. found: 477.2013.

1,3-Dioxoisoindolin-2-yl 3-(*N*-(tert-butyl)-5-methylthiophene-2-carboxamido)propanoate (3q)

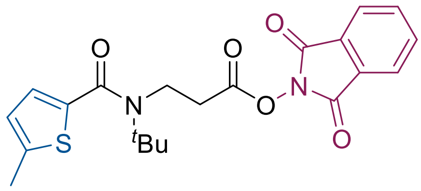

The title compound was prepared through general procedure **A-D** and purified by recrystallization from petroleum ether /ethyl acetate (v/v, = 6/1) as white solid (2.49 g, 60%).

M.p. 134.4 - 136.8 ℃.

^{1}H NMR (400 MHz, CDCl$_3$) δ 7.89 (dd, J = 5.5, 3.1 Hz, 2H), 7.81 (dd, J = 5.6, 3.1 Hz, 2H), 7.13 (d, J = 3.6 Hz, 1H), 6.70 (dd, J = 3.6, 1.1 Hz, 1H), 4.04 – 3.97 (m, 2H), 3.08 – 3.00 (m, 2H), 2.50 (s, 3H), 1.55 (s, 9H).

^{13}C NMR (101 MHz, CDCl$_3$) δ 167.2, 166.9, 161.7, 143.5, 137.5, 134.9, 128.8, 128.5, 125.3, 124.1, 58.1, 42.4, 33.9, 28.8, 15.3.

IR (KBr) 3650, 2831, 1744, 1617, 1365, 1189, 777, 696, 518, 483 cm^{-1}.

HRMS (ESI-MS) *m/z* calcd for $C_{21}H_{22}N_2O_5S$ [M+H]$^+$: 415.1322. found: 415.1315.

1,3-Dioxoisoindolin-2-yl 3-(N-(tert-butyl)-3-methylbenzofuran-2-carboxamido)propanoate (3r)

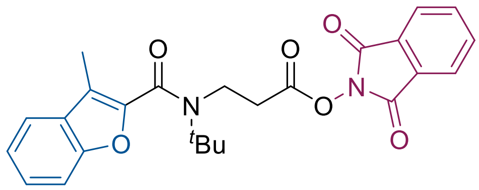

The title compound was prepared through general procedure **A-D** and purified by recrystallization from petroleum ether /ethyl acetate (v/v, = 6/1) as white solid (3.36 g, 75%).

M.p. 137.4 - 139.6 ℃.

^{1}H NMR (400 MHz, CDCl$_3$) δ 7.88 (dd, J = 5.6, 3.1 Hz, 2H), 7.79 (dd, J = 5.6, 3.1 Hz, 2H), 7.58 (d, J = 8.0 Hz, 2H), 7.40 (t, J = 7.8 Hz, 1H), 7.28 (t, J = 7.5 Hz, 1H), 3.83 (dd, J = 9.4, 5.8 Hz, 2H), 3.37 (dd, J = 9.2, 6.1 Hz, 2H), 2.48 (s, 3H), 1.60 (s, 9H).

^{13}C NMR (101 MHz, CDCl$_3$) δ 167.6, 164.1, 161.8, 153.2, 145.2, 134.8, 129.1, 128.8, 126.6, 124.0, 123.0, 121.8, 120.3, 111.9, 58.2, 41.8, 34.6, 28.6, 9.2.

IR (KBr) 3678, 2832, 1744, 1625, 1366, 1192, 966, 877, 772, 701 cm^{-1}.
HRMS (ESI-MS) *m/z* calcd for $C_{25}H_{24}N_2O_6$ $[M+H]^+$: 449.1707. found: 449.1699.

1,3-Dioxoisoindolin-2-yl 3-(benzyl(tert-butyl)amino)-3-oxopropanoate (8)

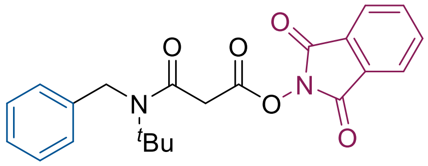

To a round bottomed flask charged with benzaldehyde (10 mmol, 1.0 equiv) was added MeOH (0.2 M) and tert-butylamine (50 mmol, 5.0 equiv). After stirring for 16 hours, $NaBH_4$ (15 mmol, 1.5 equiv) was added slowly, and the resultant mixture was stirred until bubbling ceased. The reaction mixture was quenched with 1 M NaOH (aq.) and extracted with EtOAc (3x). The combined organic layers were washed with 1 M HCl (aq.), and the resulting aqueous layer was brought to pH 14 with 2 M NaOH (aq.) and extracted with EtOAc (3x). The combined organic layers were dried over Na_2SO_4, filtered, and concentrated under reduced pressure to afford N-benzyl-2-methylpropan-2-amine. After that, DCM (0.25 M), Et_3N (1.2 equiv), and methyl 3-chloro-3-oxopropanoate (1.2 equiv) was added. After stirring for 4 hours, the reaction mixture was quenched with MeOH, diluted with H_2O, and extracted with DCM (3x). The combined organic layers were dried over Na_2SO_4, filtered, and concentrated under reduced pressure. The crude residue was purified on silica gel using petroleum ether /EtOAc (v/v = 12/1) to afford methyl 3-(benzyl(tert-butyl)amino)-3-oxopropanoate. Then 0.1M NaOH solution (1.1 equiv) was added to a solution of 3-(benzyl(tert-butyl)amino)-3-oxopropanoate in methanol (maintaining 1/1 methanol/water ratio). Reaction mixture was stirred at room temperature for 2 h and methanol was evaporated. Aqueous solution in residue was acidified with 30 *mL* of 1M HCl and stirred additionally for 10 min. Formed precipitate was collected, washed with water and dried in vacuo. Beige solid 3-(benzyl(tert-butyl)amino)-3-oxopropanoic acid was obtained. Next, DCC (1.5 equiv) was added to a solution of acid (1 equiv), N-hydroxyphthalimide (1.05 equiv) and DMAP (10 mol%) in DCM (0.2 M) under ice bath. Reaction mixture was stirred at room temperature for 15 h, filtered and evaporated. Purification by recrystallization from petrol ether/ EtOAc (v/v, = 5/1) gave **8** of white solid (1.77 g, 45 %).

M.p. 145.6 - 147.2 ℃.
^{1}H NMR (400 MHz, $CDCl_3$) δ 7.91 (dd, J = 5.5, 3.1 Hz, 2H), 7.81 (dd, J = 5.5, 3.1 Hz, 2H), 7.42 (t, J = 7.9 Hz, 2H), 7.35 – 7.28 (m, 3H), 4.67 (s, 2H), 3.72 (s, 2H), 1.51 (s, 9H).
^{13}C NMR (101 MHz, $CDCl_3$) δ 165.1, 164.4, 161.6, 138.2, 134.9, 129.1, 128.9, 127.5, 125.5, 124.0, 58.9, 49.3, 41.0, 28.5.
IR (KBr) 2780, 1745, 1620, 1402, 1366, 1080, 567 cm^{-1}.
HRMS (ESI-MS) *m/z* calcd for $C_{22}H_{22}N_2O_5$ $[M+H]^+$: 395.1601. found: 395.1598.

5 General procedure for the synthesis of carboamination products and characterization of the carboamination products

5.1 General procedure F for the synthesis of carboamination products

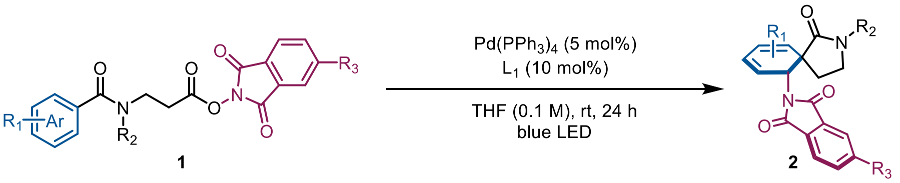

In a Schlenk tube with a magnetic bar under Ar atmosphere was added $Pd(PPh_3)_4$ (5 mol %, 11.5 mg), L_1 (10 mol%, 11.5 mg) and then the substrates **1** (0.2 mmol) were added in THF (2 *mL*). The mixture was stirred at room temperature under Ar atmosphere and under blue LEDs (30 W×2) irradition for 24 h until the starting material was completely consumed (monitored by TLC). The corresponding reaction mixture was filtered through a pad of celite, washed with EtOAc and concentrated under reduced pressure. The residue was purified by flash chromatography on silica gel using petroleum ether/ethyl acetate as eluent to afford the desired products.

5.2 Characterization of the carboamination products

2-(2-(Tert-butyl)-1-oxo-2-azaspiro[4.5]deca-7,9-dien-6-yl)isoindoline-1,3-dione (2a)

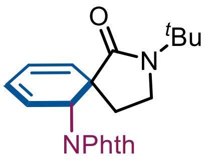

According to the general procedure **F**, using the corresponding substrate **1a** (0.2 mmol, 1.0 equiv), $Pd(PPh_3)_4$ (0.01 mmol, 5 mol%) , L_1 (0.02 mmol, 10 mol%) and stirring at room temperature for 24 h under 30 W blue LEDs irradiation. The reaction mixture was filtered through a pad of silica gel and concentrated in vacuo, then purified by chromatography (petroleum ether/ethyl acetate = 7/1) to give **2a** (56.1 mg, 0.16 mmol, 80%, d.r. > 20:1) as white solid.

M.p. 129.4 - 130.8 ℃.
^{1}H NMR (500 MHz, $CDCl_3$) δ 7.86 (dd, J = 5.4, 3.1 Hz, 2H), 7.75 (dd, J = 5.5, 3.1 Hz, 2H), 6.18 – 6.09 (m, 2H), 5.71 (d, J = 8.6 Hz, 1H), 5.65 – 5.60 (m, 1H), 5.53 (dd, J = 4.0, 2.2 Hz, 1H), 3.34 (ddd, J = 9.9, 7.8, 5.7 Hz, 1H), 3.10 (ddd, J = 9.9, 7.9, 5.5 Hz, 1H), 2.36 (ddd, J = 13.3, 7.8, 5.7 Hz, 1H), 1.75 (ddd, J = 13.2, 7.9, 5.5 Hz, 1H), 1.31 (s, 9H).
^{13}C NMR (126 MHz, $CDCl_3$) δ 175.3, 167.8, 134.2, 131.8, 128.3, 125.0, 123.5, 123.4, 122.9, 54.3, 51.3, 49.4, 42.6, 27.6, 27.0.
IR (KBr) 3638, 3476, 3049, 2969, 2791, 1775, 1718, 1462, 1387, 1329, 1287, 1251, 1097, 975, 895, 802, 728, 613, 535 cm^{-1}.
HRMS (ESI-MS) *m/z* calcd for $C_{21}H_{22}N_2O_3$ $[M+H]^+$: 351.1703. found: 351.1697.

2-(2-(Tert-butyl)-1-oxo-2-azaspiro[4.5]deca-7,9-dien-6-yl)-5-methylisoindoline-1,3-dione (2b)

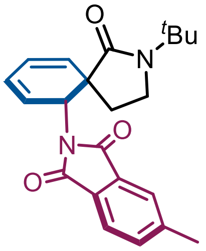

According to the general procedure **F**, using the corresponding substrate **1b** (0.2 mmol, 1.0 equiv), $Pd(PPh_3)_4$ (0.01 mmol, 5 mol%) , L_1 (0.02 mmol, 10 mol%) and stirring at room temperature for 24 h under 30 W blue LEDs irradiation. The reaction mixture was filtered through a pad of silica gel and concentrated in vacuo, then purified by chromatography (petroleum ether/ethyl acetate = 7/1) to give **2b** (46.6 mg, 0.128 mmol, 64%, d.r. = 1.5: 1) as colorless oil.

^{1}H NMR (400 MHz, $CDCl_3$) δ 7.74 (d, J = 7.6 Hz, 1H), 7.65 (s, 1H), 7.53 (d, J = 7.6 Hz, 1H), 6.13 (tdd, J = 9.3, 3.8, 2.7 Hz, 2H), 5.75 – 5.66 (m, 1H), 5.63 (ddd, J = 9.2, 3.9, 1.0 Hz, 1H), 5.51 (dd, J = 3.7, 2.1 Hz, 1H), 3.33 (ddd, J = 9.9, 7.9, 5.6 Hz, 1H), 3.10 (ddd, J = 9.9, 7.8, 5.6 Hz, 1H), 2.53 (s, 3H), 2.37 (ddd, J = 13.2, 7.8, 5.5 Hz, 1H), 1.75 (ddd, J = 13.3, 7.9, 5.6 Hz, 1H), 1.32 (s, 9H).
^{13}C NMR (101 MHz, $CDCl_3$) δ 175.4, 168.0, 167.9, 145.6, 134.8, 132.1, 129.2, 128.4, 124.9, 123.9, 123.5, 123.4, 123.1, 54.3, 51.3, 49.4, 42.6, 27.6, 27.0, 22.1.
IR (KBr) 3694, 3472, 2927, 1772, 1717, 1409, 1331, 1288, 1250, 1150, 1100, 1043, 849, 738, 691, 614, 502 cm^{-1}.
HRMS (ESI-MS) *m/z* calcd for $C_{22}H_{24}N_2O_3$ $[M+H]^+$: 365.1860. found: 365.1852

2-(2-(Tert-butyl)-10-(3,5-dichlorophenyl)-1-oxo-2-azaspiro[4.5]deca-7,9-dien-6-yl)isoindoline-1,3-dione (2c)

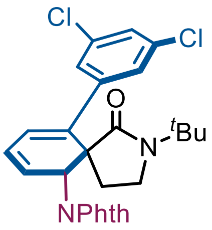

According to the general procedure **F**, using the corresponding substrate **1c** (0.2 mmol, 1.0 equiv), $Pd(PPh_3)_4$ (0.01 mmol, 5 mol%) , L_1 (0.02 mmol, 10 mol%) and stirring at room temperature for 48 h under 30 W blue LEDs irradiation. The reaction mixture was filtered through a pad of silica gel and concentrated in vacuo, then purified by chromatography (petroleum ether/ethyl acetate = 7/1) to give **2c** (98.1 mg, 0.198 mmol, 99%, d.r. > 20:1) as colorless oil.

^{1}H NMR (400 MHz, $CDCl_3$) δ 7.88 (dd, J = 5.4, 3.1 Hz, 2H), 7.77 (dd, J = 5.5, 3.1 Hz, 2H), 7.27 – 7.22 (m, 3H), 6.25 (ddd, J = 9.3, 5.7, 2.6 Hz, 1H), 6.18 (d, J = 5.6 Hz, 1H), 5.82 (ddd, J = 9.5, 3.6, 0.9 Hz, 1H), 5.67 (t, J = 3.2 Hz, 1H), 3.18 (td, J = 9.7, 3.2 Hz, 1H), 3.01 (dt, J = 9.9, 7.9 Hz, 1H), 2.82 (ddd, J = 14.3, 8.2, 3.2 Hz, 1H), 1.99 – 1.91 (m, 1H), 1.22 (s, 9H).
^{13}C NMR (101 MHz, $CDCl_3$) δ 173.5, 142.8, 139.4, 134.5, 134.4, 131.6, 127.4, 126.2, 125.2, 125.1, 124.8, 123.5, 55.5, 54.5, 52.4, 43.0, 27.3, 24.2.
IR (KBr) 3692, 2881, 1724, 1417, 1295, 804, 747, 477 cm^{-1}.
HRMS (ESI-MS) *m/z* calcd for $C_{27}H_{24}Cl_2N_2O_3$ $[M+H]^+$: 495.1237. found: 495.1230.

2-(2-(Tert-butyl)-10-methyl-1-oxo-2-azaspiro[4.5]deca-7,9-dien-6-yl)isoindoline-1,3-dione (2f)

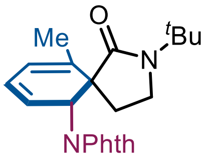

According to the general procedure **F**, using the corresponding substrate **1f** (0.2 mmol, 1.0 equiv), $Pd(PPh_3)_4$ (0.01 mmol, 5 mol%) , L_1 (0.02 mmol, 10 mol%) and stirring at room temperature for 24 h under 30 W blue LEDs irradiation. The reaction mixture was filtered through a pad of silica gel and concentrated in vacuo, then purified by chromatography (petroleum ether/ethyl acetate = 7/1) to give **2f** (44.5 mg, 0.122 mmol, 61%, d.r. > 20:1) as white solid.

M.p. 161.9 - 163.4 ℃.

^{1}H NMR (400 MHz, $CDCl_3$) δ 7.87 (dd, J = 5.5, 3.1 Hz, 2H), 7.75 (dd, J = 5.5, 3.1 Hz, 2H), 6.01 (ddd, J = 9.1, 5.5, 3.1 Hz, 1H), 5.83 (d, J = 5.4 Hz, 1H), 5.69 – 5.61 (m, 2H), 3.34 – 3.27 (m, 1H), 2.98 – 2.85 (m, 2H), 1.83 (s, 3H), 1.71 (ddd, J = 11.8, 9.7, 6.9 Hz, 1H), 1.21 (s, 9H).

^{13}C NMR (101 MHz, $CDCl_3$) δ 174.7, 139.5, 134.2, 131.7, 124.6, 123.4, 122.8, 120.9, 56.4, 54.2, 53.1, 43.7, 27.4, 23.2, 18.4.

IR (KBr) 3669, 3482, 3051, 2920, 1777, 1720, 1459, 1408, 1331, 1286, 1252, 1117, 1080, 1041, 974, 884, 798, 731, 620, 564, 538, 472 cm^{-1}.

HRMS (ESI-MS) *m/z* calcd for $C_{22}H_{24}N_2O_3$ $[M+H]^+$: 365.1860. found: 365.1854.

2-(2-(Tert-butyl)-10-ethyl-1-oxo-2-azaspiro[4.5]deca-7,9-dien-6-yl)isoindoline-1,3-dione (2g)

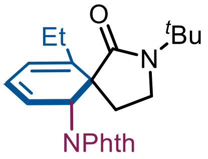

According to the general procedure **F**, using the corresponding substrate **1g** (0.2 mmol, 1.0 equiv), $Pd(PPh_3)_4$ (0.01 mmol, 5 mol%) , L_1 (0.02 mmol, 10 mol%) and stirring at room temperature for 24 h under 30 W blue LEDs irradiation. The reaction mixture was filtered through a pad of silica gel and concentrated in vacuo, then purified by chromatography (petroleum ether/ethyl acetate = 7/1) to give **2g** (56.8 mg, 0.15 mmol, 75%, d.r. > 20:1) as white solid.

M.p. 131.2 - 132.7 ℃.

^{1}H NMR (400 MHz, $CDCl_3$) δ 7.91 – 7.82 (m, 2H), 7.75 (dd, J = 5.5, 3.0 Hz, 2H), 6.11 – 6.02 (m, 1H), 5.81 (d, J = 5.4 Hz, 1H), 5.70 – 5.62 (m, 2H), 3.30 (td, J = 9.8, 2.0 Hz, 1H), 3.04 (ddd, J = 14.1, 8.0, 2.0 Hz, 1H), 2.92 – 2.81 (m, 1H), 2.48 – 2.36 (m, 1H), 2.03 – 1.91 (m, 1H), 1.66 (dt, J = 13.9, 9.3 Hz, 1H), 1.18 (s, 9H), 1.08 (t, J = 7.4 Hz, 3H).

^{13}C NMR (101 MHz, $CDCl_3$) δ 174.7, 145.5, 134.2, 131.7, 124.6, 123.3, 123.1, 117.8, 56.9, 54.1, 53.5, 43.6, 27.3, 23.6, 23.4, 11.5.

IR (KBr) 3671, 3054, 2880, 1777, 1720, 1463, 1413, 1329, 1291, 1251, 1117, 1044, 881, 798, 730, 621, 535, 474 cm^{-1}.

HRMS (ESI-MS) *m/z* calcd for $C_{23}H_{26}N_2O_3$ $[M+H]^+$: 379.2016. found: 379.2010.

2-(2-(Tert-butyl)-10-isopropyl-1-oxo-2-azaspiro[4.5]deca-7,9-dien-6-yl)isoindoline-1,3-dione (2h)

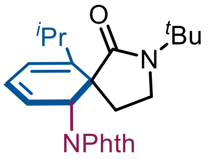

According to the general procedure **F**, using the corresponding substrate **1h** (0.2 mmol, 1.0 equiv), $Pd(PPh_3)_4$ (0.01 mmol, 5 mol%) , L_1 (0.02 mmol, 10 mol%) and stirring at room temperature for 36 h under 30 W blue LEDs irradiation. The reaction mixture was filtered through a pad of silica gel and

concentrated in vacuo, then purified by chromatography (petroleum ether/ethyl acetate = 7/1) to give **2h** (73 mg, 0.186 mmol, 93%, d.r. > 20:1) as white solid.

M.p. 120.8 - 123.3 ℃.
^{1}H NMR (400 MHz, CDCl$_3$) δ 7.86 (dd, J = 5.4, 3.1 Hz, 2H), 7.74 (dd, J = 5.5, 3.1 Hz, 2H), 6.07 (ddd, J = 9.1, 5.6, 3.2 Hz, 1H), 5.88 (d, J = 5.6 Hz, 1H), 5.67 (t, J = 3.0 Hz, 1H), 5.65 – 5.58 (m, 1H), 3.30 (td, J = 9.8, 1.9 Hz, 1H), 3.08 (ddd, J = 14.2, 8.1, 1.9 Hz, 1H), 2.86 (dt, J = 9.7, 8.3 Hz, 1H), 2.38 (p, J = 6.8 Hz, 1H), 1.71 (ddd, J = 14.2, 10.0, 8.7 Hz, 1H), 1.19 – 1.18 (m, 12H), 1.09 (d, J = 6.8 Hz, 3H).
^{13}C NMR (101 MHz, CDCl$_3$) δ 174.7, 150.6, 134.2, 131.7, 124.7, 123.5, 123.1, 117.9, 57.5, 54.1, 53.9, 43.4, 30.3, 27.3, 24.5, 23.5, 23.5.
IR (KBr) 3635, 3056, 2964, 2875, 2716, 1776, 1721, 1608, 1462, 1366, 1289, 1251, 1111, 1027, 977, 883, 810, 777, 729. 625, 534, 477 cm^{-1}.
HRMS (ESI-MS) *m/z* calcd for $C_{24}H_{28}N_2O_3$ [M+H]$^+$: 393.2173. found: 393.2164.

2-(10-Benzyl-2-(tert-butyl)-1-oxo-2-azaspiro[4.5]deca-7,9-dien-6-yl)isoindoline-1,3-dione (2i)

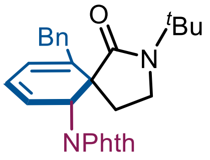

According to the general procedure **F**, using the corresponding substrate **1i** (0.2 mmol, 1.0 equiv), Pd(PPh$_3$)$_4$ (0.01 mmol, 5 mol%) , L$_1$ (0.02 mmol, 10 mol%) and stirring at room temperature for 36 h under 30 W blue LEDs irradiation. The reaction mixture was filtered through a pad of silica gel and concentrated in vacuo, then purified by chromatography (petroleum ether/ethyl acetate = 7/1) to give **2i** (50.2 mg, 0.114 mmol, 57%, d.r. > 20:1) as colorless oil.

^{1}H NMR (400 MHz, CDCl$_3$) δ 7.88 (dd, J = 5.5, 3.1 Hz, 2H), 7.76 (dd, J = 5.5, 3.0 Hz, 2H), 7.33 – 7.27 (m, 2H), 7.23 (d, J = 7.3 Hz, 3H), 5.98 (ddd, J = 9.2, 5.5, 3.2 Hz, 1H), 5.73 (d, J = 3.1 Hz, 1H), 5.68 (dd, J = 9.6, 2.7 Hz, 1H), 5.45 (d, J = 5.4 Hz, 1H), 3.72 (d, J = 17.0 Hz, 1H), 3.33 – 3.18 (m, 2H), 3.06 (ddd, J = 14.2, 8.0, 1.9 Hz, 1H), 2.85 (dt, J = 9.5, 8.2 Hz, 1H), 1.67 (ddd, J = 14.1, 9.9, 8.7 Hz, 1H), 1.20 (s, 9H).
^{13}C NMR (101 MHz, CDCl$_3$) δ 174.6, 144.0, 138.6, 134.3, 131.7, 129.9, 128.3, 126.3, 124.5, 123.7, 123.4, 121.5, 56.8, 54.3, 53.7, 43.6, 37.8, 27.4, 23.2.
IR (KBr) 3692, 2883, 2357, 1778, 1719, 1461, 1390, 1328, 1288, 1116, 1042, 887, 798, 729, 470. cm^{-1}.
HRMS (ESI-MS) *m/z* calcd for $C_{28}H_{28}N_2O_3$ [M+H]$^+$: 441.2173. found: 441.2166.

2-(2-(Tert-butyl)-10-fluoro-1-oxo-2-azaspiro[4.5]deca-7,9-dien-6-yl)isoindoline-1,3-dione (2j)

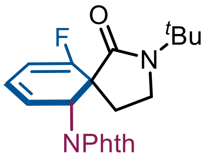

According to the general procedure **F**, using the corresponding substrate **1j** (0.2 mmol, 1.0 equiv), Pd(PPh$_3$)$_4$ (0.01 mmol, 5 mol%) , L$_1$ (0.02 mmol, 10 mol%) and stirring at room temperature for 24 h under 30 W blue LEDs irradiation. The reaction mixture was filtered through a pad of silica gel and concentrated in vacuo, then purified by chromatography (petroleum ether/ethyl acetate = 7/1) to give **2j** (51.6 mg, 0.14 mmol, 70%, d.r. > 20:1) as white solid.

M.p. 145.3 - 147.5 ℃.

^{1}H NMR (500 MHz, $CDCl_3$) δ 7.87 (dd, J = 5.4, 3.1 Hz, 2H), 7.76 (dd, J = 5.5, 3.1 Hz, 2H), 6.03 (dtd, J = 9.5, 6.0, 2.2 Hz, 1H), 5.74 (dd, J = 12.7, 6.4 Hz, 1H), 5.61 (td, J = 4.1, 2.2 Hz, 1H), 5.42 (dd, J = 9.6, 4.1 Hz, 1H), 3.43 – 3.37 (m, 1H), 3.14 (dddd, J = 9.5, 8.1, 5.2, 1.4 Hz, 1H), 2.27 – 2.14 (m, 2H), 1.35 (s, 9H).

^{13}C NMR (126 MHz, $CDCl_3$) δ 172.3 (d, J = 1.2 Hz), 167.6, 161.5 (d, J = 270.9 Hz), 123.2 (d, J = 8.6 Hz), 118.7 (d, J = 4.8 Hz), 101.9 (d, J = 19.9 Hz), 134.4, 131.6, 123.6, 54.7, 53.3 (d, J = 22.2 Hz), 51.9 (d, J = 5.2 Hz), 43.3 (d, J = 2.2 Hz), 27.4, 23.9.

^{19}F NMR (471 MHz, $CDCl_3$) δ -118.2.

IR (KBr) 3670, 3057, 2971, 1776, 1721, 1462, 1409, 1327, 1288, 1216, 1115, 1045, 940, 885, 798, 733, 619, 533, 499 cm^{-1}.

HRMS (ESI-MS) *m/z* calcd for $C_{21}H_{21}FN_2O_3$ [M+H]$^+$: 369.1609. found: 369.1603.

2-(2-(Tert-butyl)-10-chloro-1-oxo-2-azaspiro[4.5]deca-7,9-dien-6-yl)isoindoline-1,3-dione (2k)

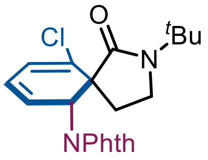

According to the general procedure **F**, using the corresponding substrate **1k** (0.2 mmol, 1.0 equiv), $Pd(PPh_3)_4$ (0.01 mmol, 5 mol%) , L_1 (0.02 mmol, 10 mol%) and stirring at room temperature for 24 h under 30 W blue LEDs irradiation. The reaction mixture was filtered through a pad of silica gel and concentrated in vacuo, then purified by chromatography (petroleum ether/ethyl acetate = 7/1) to give **2k** (37.7 mg, 0.098 mmol, 49%, d.r. > 20:1) as white solid.

M.p. 161.4 - 162.7 ℃.

^{1}H NMR (500 MHz, $CDCl_3$) δ 7.87 (dd, J = 5.4, 3.1 Hz, 2H), 7.76 (dd, J = 5.5, 3.0 Hz, 2H), 6.25 (d, J = 6.1 Hz, 1H), 6.04 (ddd, J = 9.0, 6.1, 2.5 Hz, 1H), 5.71 – 5.62 (m, 2H), 3.43 (td, J = 9.2, 4.4 Hz, 1H), 3.08 – 2.99 (m, 1H), 2.52 (ddd, J = 13.2, 8.3, 4.4 Hz, 1H), 2.14 (ddd, J = 13.8, 9.0, 6.2 Hz, 1H), 1.31 (s, 9H).

^{13}C NMR (126 MHz, $CDCl_3$) δ 172.5, 167.5, 135.9, 134.4, 131.6, 123.8, 123.6, 123.2, 122.5, 57.3, 54.7, 52.4, 43.5, 27.4, 24.3.

IR (KBr) 3689, 2923, 1777, 1719, 1460, 1380, 1330, 1291, 1252, 1218, 1103, 1007, 794, 724, 475 cm^{-1}.

HRMS (ESI-MS) *m/z* calcd for $C_{21}H_{21}ClN_2O_3$ [M+H]$^+$: 385.1313. found: 385.1308.

2-(2-(Tert-butyl)-1-oxo-10-(trifluoromethyl)-2-azaspiro[4.5]deca-7,9-dien-6-yl)isoindoline-1,3 dione (2l)

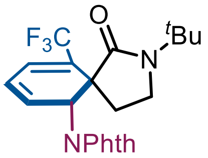

According to the general procedure **F**, using the corresponding substrate **1l** (0.2 mmol, 1.0 equiv), $Pd(PPh_3)_4$ (0.01 mmol, 5 mol%) , L_1 (0.02 mmol, 10 mol%) and stirring at room temperature for 24 h under 30 W blue LEDs irradiation. The reaction mixture was filtered through a pad of silica gel and concentrated in vacuo, then purified by chromatography (petroleum ether/ethyl acetate = 7/1) to give **2l** (70.3 mg, 0.168 mmol, 84%, d.r. > 20:1) as white solid.

M.p. 158.5 - 160.2 ℃.

^{1}H NMR (400 MHz, $CDCl_3$) δ 7.91 – 7.82 (m, 2H), 7.78 (dd, J = 5.5, 3.1 Hz, 2H), 6.79 (d, J = 5.9 Hz, 1H), 6.30 (t, J = 7.8 Hz, 1H), 5.87 (dd, J = 9.6, 4.3 Hz, 1H), 5.42 (d, J = 4.1 Hz, 1H),

3.38 (td, J = 9.5, 2.6 Hz, 1H), 3.16 (q, J = 8.4 Hz, 1H), 2.43 (ddd, J = 14.3, 7.8, 2.3 Hz, 1H), 2.21 – 2.12 (m, 1H), 1.31 (s, 9H).

^{13}C NMR (126 MHz, CDCl$_3$) δ 171.8, 134.5, 131.5, 128.2 (q, J = 28.6 Hz), 128.2 (q, J = 5.8 Hz), 126.6, 124.2, 123.9 (q, J = 273.1 Hz), 123.7, 54.5, 51.1, 50.5, 42.5, 27.3, 24.3.

^{19}F NMR (471 MHz, CDCl$_3$) δ -61.5.

IR (KBr) 3846, 3772, 3484, 3379, 2968, 2717, 1779, 1718, 1463, 1383, 1294, 1251, 1161, 1117, 1012, 915, 790, 720, 656, 537 cm^{-1}.

HRMS (ESI-MS) *m/z* calcd for $C_{22}H_{21}F_3N_2O_3$ [M+H]$^+$: 419.1577. found: 419.1569.

2-(2-(Tert-butyl)-1-oxo-10-(trifluoromethoxy)-2-azaspiro[4.5]deca-7,9-dien-6-yl)isoindoline-1,3-dione (2m)

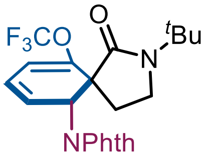

According to the general procedure **F**, using the corresponding substrate **1m** (0.2 mmol, 1.0 equiv), Pd(PPh$_3$)$_4$ (0.01 mmol, 5 mol%) , L$_1$ (0.02 mmol, 10 mol%) and stirring at room temperature for 24 h under 30 W blue LEDs irradiation. The reaction mixture was filtered through a pad of silica gel and concentrated in vacuo, then purified by chromatography (petroleum ether/ethyl acetate = 7/1) to give **2m** (78.2 mg, 0.18 mmol, 90%, d.r. > 20:1) as colorless oil.

^{1}H NMR (400 MHz, CDCl$_3$) δ 7.87 (dd, J = 5.5, 3.1 Hz, 2H), 7.77 (dd, J = 5.5, 3.1 Hz, 2H), 6.09 (ddd, J = 8.8, 6.6, 1.7 Hz, 1H), 5.96 – 5.91 (m, 1H), 5.59 – 5.49 (m, 2H), 3.39 (ddd, J = 9.4, 8.2, 6.0 Hz, 1H), 3.16 (ddd, J = 9.4, 8.4, 4.9 Hz, 1H), 2.25 (ddd, J = 14.2, 8.3, 5.9 Hz, 1H), 2.13 (ddd, J = 13.4, 8.1, 4.9 Hz, 1H), 1.34 (s, 9H).

^{13}C NMR (126 MHz, CDCl$_3$) δ 172.0, 167.5, 148.4, 134.4, 131.6, 123.6, 123.1, 120.3 (q, J = 259.7 Hz), 120.2, 106.9 (q, J = 2.3 Hz), 54.7, 53.4, 51.5, 43.3, 27.3, 23.9.

^{19}F NMR (376 MHz, CDCl$_3$) δ -57.1.

IR (KBr) 3694, 3630, 2875, 1778, 1720, 1389, 1248, 913, 796, 738 cm^{-1}.

HRMS (ESI-MS) *m/z* calcd for $C_{22}H_{21}F_3N_2O_4$ [M+H]$^+$: 435.1526. found: 435.1519.

2-(Tert-butyl)-10-(1,3-Dioxoisoindolin-2-yl)-1-oxo-2-azaspiro[4.5]deca-6,8-diene-6-carbonitrile (2n)

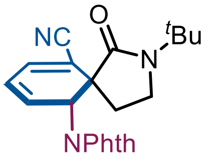

According to the general procedure **F**, using the corresponding substrate **1n** (0.2 mmol, 1.0 equiv), Pd(PPh$_3$)$_4$ (0.01 mmol, 5 mol%) , L$_1$ (0.02 mmol, 10 mol%) and stirring at room temperature for 48 h under 30 W blue LEDs irradiation. The reaction mixture was filtered through a pad of silica gel and concentrated in vacuo, then purified by chromatography (petroleum ether/ethyl acetate = 7/1) to give **2n** (28.5 mg, 0.076 mmol, 38%, d.r. > 20:1) as white solid.

M.p. 152.6 - 154.3 ℃.

^{1}H NMR (400 MHz, CDCl$_3$) δ 7.87 (dd, J = 5.5, 3.1 Hz, 2H), 7.79 (dd, J = 5.5, 3.1 Hz, 2H), 6.89 (d, J = 5.9 Hz, 1H), 6.29 (ddd, J = 9.6, 5.9, 2.2 Hz, 1H), 5.93 (dd, J = 9.5, 4.6 Hz, 1H), 5.41 (dd, J = 4.7, 2.2 Hz, 1H), 3.60 (ddd, J = 10.0, 8.1, 5.5 Hz, 1H), 3.23 (ddd, J = 10.0, 8.3, 5.3 Hz, 1H), 2.28 (ddd, J = 13.8, 8.3, 5.5 Hz, 1H), 2.14 (ddd, J = 13.7, 8.2, 5.3 Hz, 1H), 1.38 (s, 9H).

^{13}C NMR (101 MHz, CDCl$_3$) δ 171.9, 167.3, 138.2, 134.6, 131.4, 127.8, 124.3, 123.7, 117.7, 112.6, 54.9, 50.7, 48.4, 42.9, 27.4, 25.7.

IR (KBr) 3666, 3059, 2972, 2358, 2210, 1777, 1719, 1561, 1462, 1378, 1331, 1289, 1255, 1217, 1164, 1114, 1045, 1008, 911, 888, 786, 730, 619, 563, 533, 469 cm^{-1}.
HRMS (ESI-MS) *m/z* calcd for $C_{22}H_{21}N_3O_3$ $[M+H]^+$: 376.1656. found: 376.1650.

2-(2-(Tert-butyl)-10-(naphthalen-2-yl)-1-oxo-2-azaspiro[4.5]deca-7,9-dien-6-yl)isoindoline-1,3-dione (2o)

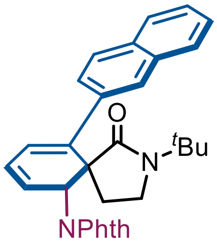

According to the general procedure **F**, using the corresponding substrate **1o** (0.2 mmol, 1.0 equiv), $Pd(PPh_3)_4$ (0.01 mmol, 5 mol%) , L_1 (0.02 mmol, 10 mol%) and stirring at room temperature for 48 h under 30 W blue LEDs irradiation. The reaction mixture was filtered through a pad of silica gel and concentrated in vacuo, then purified by chromatography (petroleum ether/ethyl acetate = 7/1) to give **2o** (81.0 mg, 0.170 mmol, 85%, d.r. > 20:1) as white solid.

M.p. 196.2 - 197.9 ℃.
^{1}H NMR (400 MHz, CDCl$_3$) δ 7.90 (dd, J = 5.5, 3.1 Hz, 2H), 7.87 – 7.85 (m, 1H), 7.83 – 7.73 (m, 5H), 7.52 (dd, J = 8.5, 1.8 Hz, 1H), 7.48 – 7.43 (m, 2H), 6.33 (ddd, J = 9.2, 5.7, 2.4 Hz, 1H), 6.27 (d, J = 5.6 Hz, 1H), 5.78 (ddd, J = 9.4, 3.9, 1.0 Hz, 1H), 5.71 (dd, J = 3.9, 2.4 Hz, 1H), 3.07 – 3.00 (m, 2H), 2.67 (ddd, J = 14.1, 7.2, 4.6 Hz, 1H), 2.16 – 2.06 (m, 1H), 1.24 (s, 9H).
^{13}C NMR (101 MHz, CDCl$_3$) δ 174.1, 141.6, 137.5, 134.3, 133.1, 132.7, 131.8, 128.1, 127.6, 127.4, 126.9, 126.7, 126.1, 125.9, 125.8, 124.4, 123.5, 122.9, 55.5, 54.3, 52.1, 43.0, 27.4, 24.6.
IR(KBr) 3872, 3636, 3054, 2968, 1776, 1717, 1463, 1383, 1327, 1281, 1117, 1011, 889, 820, 732, 621, 538, 480 cm^{-1}.
HRMS (ESI-MS) *m/z* calcd for $C_{31}H_{28}N_2O_3$ $[M+H]^+$: 477.2173. found: 477.2165.

2-(2-(Tert-butyl)-1-oxo-10-phenyl-2-azaspiro[4.5]deca-7,9-dien-6-yl)isoindoline-1,3-dione (2p)

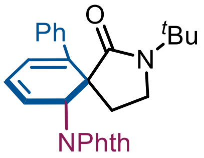

According to the general procedure **F**, using the corresponding substrate **1p** (0.2 mmol, 1.0 equiv), $Pd(PPh_3)_4$ (0.01 mmol, 5 mol%) , L_1 (0.02 mmol, 10 mol%) and stirring at room temperature for 48 h under 30 W blue LEDs irradiation. The reaction mixture was filtered through a pad of silica gel and concentrated in vacuo, then purified by chromatography (petroleum ether/ethyl acetate = 7/1) to give **2p** (72.5 mg, 0.170 mmol, 85%, d.r. > 20:1) as white solid.

M.p. 173.2 - 175.1 ℃.
^{1}H NMR (400 MHz, CDCl$_3$) δ 7.87 (dd, J = 5.5, 3.0 Hz, 2H), 7.75 (dd, J = 5.5, 3.1 Hz, 2H), 7.37 – 7.33 (m, 2H), 7.27 (ddt, J = 8.7, 4.0, 2.5 Hz, 4H), 6.26 (ddd, J = 9.5, 5.7, 2.5 Hz, 1H), 6.13 (d, J = 5.7 Hz, 1H), 5.75 – 5.69 (m, 1H), 5.64 (dd, J = 3.9, 2.5 Hz, 1H), 3.04 – 2.97 (m, 2H), 2.61 (dt, J = 14.0, 6.1 Hz, 1H), 2.04 (dt, J = 14.1, 8.0 Hz, 1H), 1.21 (s, 9H).
^{13}C NMR (101 MHz, CDCl$_3$) δ 174.0, 141.5, 140.0, 134.3, 131.7, 128.1, 127.9, 127.4, 125.7, 123.7, 123.4, 122.7, 55.2, 54.3, 52.0, 42.9, 27.3, 24.5.
IR(KBr) 3634, 3051, 2966, 1776, 1717, 1462, 1388, 1333, 1285, 1091, 885, 817, 709, 621, 531 cm^{-1}.
HRMS (ESI-MS) *m/z* calcd for $C_{27}H_{26}N_2O_3$ $[M+H]^+$: 427.2016. found: 427.2010.

2-(2-(Tert-butyl)-1-oxo-10-(4-(trifluoromethyl)phenyl)-2-azaspiro[4.5]deca-7,9-dien-6-yl)isoindoline-1,3-dione (2q)

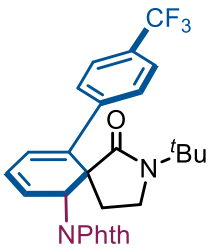

According to the general procedure **F**, using the corresponding substrate **1q** (0.2 mmol, 1.0 equiv), $Pd(PPh_3)_4$ (0.01 mmol, 5 mol%) , L_1 (0.02 mmol, 10 mol%) and stirring at room temperature for 48 h under 30 W blue LEDs irradiation. The reaction mixture was filtered through a pad of silica gel and concentrated in vacuo, then purified by chromatography (petroleum ether/ethyl acetate = 7/1) to give **2q** (86.0 mg, 0.174 mmol, 87%, d.r. > 20:1) as white solid.

M.p. 197.3 - 199.8 ℃.

^{1}H NMR (500 MHz, $CDCl_3$) δ 7.80 (dd, J = 5.4, 3.1 Hz, 2H), 7.68 (dd, J = 5.5, 3.0 Hz, 2H), 7.46 (d, J = 8.1 Hz, 2H), 7.40 (d, J = 8.2 Hz, 2H), 6.20 (ddd, J = 9.1, 5.7, 2.5 Hz, 1H), 6.10 (d, J = 5.7 Hz, 1H), 5.71 (dd, J = 9.5, 3.8 Hz, 1H), 5.57 (t, J = 3.2 Hz, 1H), 3.02 (td, J = 9.6, 3.3 Hz, 1H), 2.95 (dt, J = 9.8, 7.8 Hz, 1H), 2.60 (ddd, J = 14.1, 8.0, 3.2 Hz, 1H), 1.91 (ddd, J = 14.2, 9.2, 7.6 Hz, 1H), 1.13 (s, 9H).

^{13}C NMR (126 MHz, $CDCl_3$) δ 173.6, 143.7, 140.4, 134.4, 131.7, 129.4 (q, J = 32.3 Hz), 128.4, 125.4, 125.0, 124.8 (q, J = 3.8 Hz), 124.2 (q, J = 271.9 Hz), 123.8, 123.5, 55.2, 54.4, 52.0, 42.9, 27.3, 24.4.

^{19}F NMR (471 MHz, $CDCl_3$) δ -62.5.

IR(KBr) 3692, 2881, 1778, 1720, 1465, 1411, 1324, 1167, 1122, 1017, 842, 729 cm^{-1}.

HRMS (ESI-MS) *m/z* calcd for $C_{28}H_{25}F_3N_2O_3$ $[M+H]^+$: 495.1890. found: 495.1880.

2-(2-(Tert-butyl)-1-oxo-10-(4-(trifluoromethoxy)phenyl)-2-azaspiro[4.5]deca-7,9-dien-6-yl)isoindoline-1,3-dione (2r)

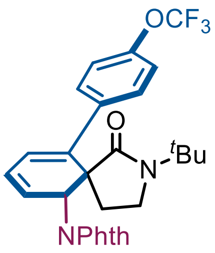

According to the general procedure **F**, using the corresponding substrate **1r** (0.2 mmol, 1.0 equiv), $Pd(PPh_3)_4$ (0.01 mmol, 5 mol%) , L_1 (0.02 mmol, 10 mol%) and stirring at room temperature for 48 h under 30 W blue LEDs irradiation. The reaction mixture was filtered through a pad of silica gel and concentrated in vacuo, then purified by chromatography (petroleum ether/ethyl acetate = 7/1) to give **2r** (88.8 mg, 0.174 mmol, 87%, d.r. > 20:1) as colorless oil.

^{1}H NMR (400 MHz, $CDCl_3$) δ 7.86 (dd, J = 5.4, 3.0 Hz, 2H), 7.75 (dd, J = 5.4, 3.1 Hz, 2H), 7.42 – 7.35 (m, 2H), 7.15 – 7.09 (m, 2H), 6.24 (ddd, J = 9.5, 5.7, 2.5 Hz, 1H), 6.12 (d, J = 5.6 Hz, 1H), 5.75 (ddd, J = 9.5, 3.8, 0.9 Hz, 1H), 5.65 (dd, J = 3.8, 2.6 Hz, 1H), 3.10 – 2.96 (m, 2H), 2.66 (ddd, J = 14.1, 7.9, 3.7 Hz, 1H), 1.98 (ddd, J = 14.1, 9.1, 7.4 Hz, 1H), 1.19 (s, 9H).

^{13}C NMR (126 MHz, $CDCl_3$) δ 173.7, 148.5 (q, J = 2.3 Hz), 140.4, 138.6, 134.3, 131.7, 129.7, 125.5, 124.5, 123.5, 123.4, 120.4(q, J = 258.1 Hz), 120.3, 55.3, 54.3, 52.0, 42.9, 27.3, 24.5.

^{19}F NMR (376 MHz, $CDCl_3$) δ -57.8.

IR(KBr) 3628, 2882, 1776, 1718, 1385, 1258, 1216, 1165, 912, 796, 735, 659 cm^{-1}.

HRMS (ESI-MS) *m/z* calcd for $C_{28}H_{25}F_3N_2O_4$ $[M+H]^+$: 511.1839. found: 511.1831.

2-(2-(Tert-butyl)-10-(4-(tert-butyl)phenyl)-1-oxo-2-azaspiro[4.5]deca-7,9-dien-6-yl)isoindoline-1,3-dione (2s)

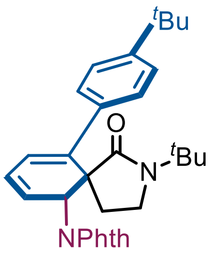

According to the general procedure **F**, using the corresponding substrate **1s** (0.2 mmol, 1.0 equiv), $Pd(PPh_3)_4$ (0.01 mmol, 5 mol%) , L_1 (0.02 mmol, 10 mol%) and stirring at room temperature for 48 h under 30 W blue LEDs irradiation. The reaction mixture was filtered through a pad of silica gel and concentrated in vacuo, then purified by chromatography (petroleum ether/ethyl acetate = 7/1) to give **2s** (79.2 mg, 0.164 mmol, 82%, d.r. > 20:1) as colorless oil.

^{1}H NMR (400 MHz, $CDCl_3$) δ 7.87 (dd, *J* = 5.4, 3.1 Hz, 2H), 7.75 (dd, *J* = 5.5, 3.1 Hz, 2H), 7.30 (s, 4H), 6.26 (ddd, *J* = 9.4, 5.8, 2.5 Hz, 1H), 6.15 (d, *J* = 5.7 Hz, 1H), 5.72 (dd, *J* = 9.4, 3.9 Hz, 1H), 5.65 (dd, *J* = 3.8, 2.5 Hz, 1H), 3.08 – 2.97 (m, 2H), 2.61 (ddd, *J* = 14.1, 7.9, 4.1 Hz, 1H), 2.06 (ddd, *J* = 14.0, 9.0, 7.1 Hz, 1H), 1.31 (s, 9H), 1.22 (s, 9H).
^{13}C NMR (101 MHz, $CDCl_3$) δ 174.0, 150.3, 141.6, 136.9, 134.2, 131.8, 127.8, 125.8, 124.7, 123.4, 123.4, 122.6, 55.3, 54.2, 52.1, 42.9, 34.5, 31.3, 27.3, 24.6.
IR(KBr) 3695, 2962, 1721, 1466, 1390, 1334, 834, 729 cm^{-1}.
HRMS (ESI-MS) *m/z* calcd for $C_{31}H_{34}N_2O_3$ $[M+H]^+$: 483.2642. found: 483.2634.

2-(2-(Tert-butyl)-1-oxo-10-(p-tolyl)-2-azaspiro[4.5]deca-7,9-dien-6-yl)isoindoline-1,3-dione (2t)

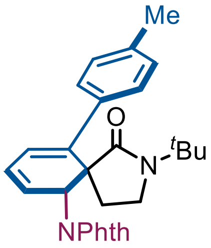

According to the general procedure **F**, using the corresponding substrate **1t** (0.2 mmol, 1.0 equiv), $Pd(PPh_3)_4$ (0.01 mmol, 5 mol%) , L_1 (0.02 mmol, 10 mol%) and stirring at room temperature for 48 h under 30 W blue LEDs irradiation. The reaction mixture was filtered through a pad of silica gel and concentrated in vacuo, then purified by chromatography (petroleum ether/ethyl acetate = 7/1) to give **2t** (79.3 mg, 0.180 mmol, 90%, d.r. > 20:1) as colorless oil.

^{1}H NMR (400 MHz, $CDCl_3$) δ 7.88 (dd, *J* = 5.4, 3.1 Hz, 2H), 7.75 (dd, *J* = 5.5, 3.1 Hz, 2H), 7.26 (d, *J* = 8.2 Hz, 2H), 7.09 (d, *J* = 7.9 Hz, 2H), 6.26 (ddd, *J* = 9.4, 5.8, 2.5 Hz, 1H), 6.12 (d, *J* = 5.7 Hz, 1H), 5.71 (ddd, *J* = 9.4, 3.9, 0.9 Hz, 1H), 5.65 (dd, *J* = 3.9, 2.5 Hz, 1H), 3.07 – 2.96 (m, 2H), 2.62 (ddd, *J* = 14.1, 7.7, 4.3 Hz, 1H), 2.33 (s, 3H), 2.07 – 2.00 (m, 1H), 1.23 (s, 9H).
^{13}C NMR (101 MHz, $CDCl_3$) δ 174.1, 141.6, 137.0, 137.0, 134.3, 131.8, 128.6, 128.0, 125.8, 123.4, 123.4, 122.5, 55.3, 54.2, 52.2, 43.0, 27.3, 24.5, 21.2.
IR (KBr) 3694, 2968, 1777, 1719, 1464, 1388, 1332, 818, 726, 533 cm^{-1}.
HRMS (ESI-MS) *m/z* calcd for $C_{28}H_{28}N_2O_3$ $[M+H]^+$: 441.2173. found: 441.2166.

2-(2-(Tert-butyl)-10-(4-fluorophenyl)-1-oxo-2-azaspiro[4.5]deca-7,9-dien-6-yl)isoindoline-1,3-dione (2u)

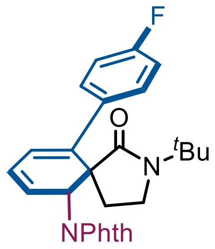

According to the general procedure **F**, using the corresponding substrate **1u** (0.2 mmol, 1.0 equiv), $Pd(PPh_3)_4$ (0.01 mmol, 5 mol%) , L_1 (0.02 mmol, 10 mol%) and stirring at room temperature for 48 h under 30 W blue LEDs irradiation. The reaction mixture was filtered through a pad of silica gel and concentrated in vacuo, then purified by chromatography (petroleum ether/ethyl acetate = 7/1) to give **2u** (81.8 mg, 0.184 mmol, 92%, d.r. > 20:1) as colorless oil.

^{1}H NMR (400 MHz, $CDCl_3$) *δ* 7.87 (dd, *J* = 5.4, 3.1 Hz, 2H), 7.75 (dd, *J* = 5.5, 3.1 Hz, 2H), 7.38 – 7.30 (m, 2H), 6.96 (t, *J* = 8.7 Hz, 2H), 6.24 (ddd, *J* = 9.6, 5.7, 2.5 Hz, 1H), 6.08 (d, *J* = 5.7 Hz, 1H), 5.73 (dd, *J* = 9.5, 3.9 Hz, 1H), 5.67 – 5.61 (m, 1H), 3.03 – 2.93 (m, 2H), 2.62 (ddd, *J* = 14.1, 6.9, 5.0 Hz, 1H), 1.97 (dt, *J* = 14.1, 8.1 Hz, 1H), 1.20 (s, 9H).
^{13}C NMR (101 MHz, $CDCl_3$) *δ* 173.8, 162.2 (d, *J* = 246.4 Hz), 140.7, 136.0 (d, *J* = 3.3 Hz), 134.3, 131.7, 130.0 (d, *J* = 7.9 Hz), 125.5, 124.0, 123.4, 123.0, 114.7 (d, *J* = 21.2 Hz), 55.3, 54.3, 52.0, 42.9, 27.3, 24.5.
^{19}F NMR (376 MHz, $CDCl_3$) *δ* -115.0.
IR (KBr) 3673, 3052, 2969, 1777, 1719, 1504, 1464, 1388, 1332, 1285, 1226, 1094, 1016, 831, 728, 538 cm^{-1}.
HRMS (ESI-MS) *m/z* calcd for $C_{27}H_{25}FN_2O_3$ $[M+H]^+$: 445.1922. found: 445.1915.

2-(2-(Tert-butyl)-10-(4-chlorophenyl)-1-oxo-2-azaspiro[4.5]deca-7,9-dien-6-yl)isoindoline-1,3-dione (2v)

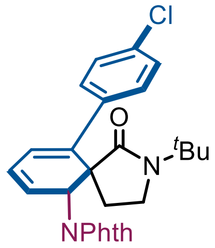

According to the general procedure **F**, using the corresponding substrate **1v** (0.2 mmol, 1.0 equiv), $Pd(PPh_3)_4$ (0.01 mmol, 5 mol%) , L_1 (0.02 mmol, 10 mol%) and stirring at room temperature for 48 h under 30 W blue LEDs irradiation. The reaction mixture was filtered through a pad of silica gel and concentrated in vacuo, then purified by chromatography (petroleum ether/ethyl acetate = 7/1) to give **2v** (80.2 mg, 0.174 mmol, 87%, d.r. > 20:1) as white solid.

M.p. 217.8 - 218.9 ℃.
^{1}H NMR (400 MHz, $CDCl_3$) *δ* 7.88 (dd, *J* = 5.4, 3.1 Hz, 2H), 7.76 (dd, *J* = 5.5, 3.1 Hz, 2H), 7.33 – 7.29 (m, 2H), 7.28 – 7.24 (m, 2H), 6.26 (ddd, *J* = 9.4, 5.7, 2.5 Hz, 1H), 6.13 (d, *J* = 5.7 Hz, 1H), 5.75 (ddd, *J* = 9.4, 3.8, 0.8 Hz, 1H), 5.64 (dd, *J* = 3.8, 2.6 Hz, 1H), 3.10 – 2.97 (m, 2H), 2.65 (ddd, *J* = 14.1, 7.9, 3.8 Hz, 1H), 1.97 (ddd, *J* = 14.0, 9.1, 7.3 Hz, 1H), 1.22 (s, 9H).
^{13}C NMR (101 MHz, $CDCl_3$) *δ* 173.8, 140.5, 138.4, 134.3, 133.3, 131.7, 129.6, 128.1, 125.5, 124.3, 123.5, 123.3, 55.3, 54.3, 52.0, 43.0, 27.3, 24.4.
IR (KBr) 3694, 2964, 2837, 2718, 1776, 1718, 1681, 1616, 1484, 1367, 1285, 1253, 1090, 1005, 884, 829, 778, 728, 531, 479 cm^{-1}.
HRMS (ESI-MS) *m/z* calcd for $C_{27}H_{25}ClN_2O_3$ $[M+H]^+$: 461.1626. found: 461.1620.

2-(2-(Tert-butyl)-10-(4-methoxyphenyl)-1-oxo-2-azaspiro[4.5]deca-7,9-dien-6-yl)isoindoline-1,3-dione (2w)

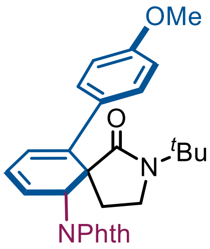

According to the general procedure **F**, using the corresponding substrate **1w** (0.2 mmol, 1.0 equiv), $Pd(PPh_3)_4$ (0.01 mmol, 5 mol%) , L_1 (0.02 mmol, 10 mol%) and stirring at room temperature for 48 h under 30 W blue LEDs irradiation. The reaction mixture was filtered through a pad of silica gel and concentrated in vacuo, then purified by chromatography (petroleum ether/ethyl acetate = 7/1) to give **2w** (69.4 mg, 0.152 mmol, 76%, d.r. > 20:1) as colorless oil.

^{1}H NMR (400 MHz, $CDCl_3$) δ 7.87 (dd, J = 5.4, 3.1 Hz, 2H), 7.75 (dd, J = 5.5, 3.0 Hz, 2H), 7.33 – 7.28 (m, 2H), 6.85 – 6.79 (m, 2H), 6.24 (ddd, J = 9.3, 5.7, 2.4 Hz, 1H), 6.09 (d, J = 5.7 Hz, 1H), 5.73 – 5.67 (m, 1H), 5.66 (dd, J = 3.8, 2.4 Hz, 1H), 3.80 (s, 3H), 3.05 – 2.96 (m, 2H), 2.63 (ddd, J = 13.9, 7.8, 4.3 Hz, 1H), 2.05 – 1.97 (m, 1H), 1.22 (s, 9H).
^{13}C NMR (101 MHz, $CDCl_3$) δ 174.1, 159.0, 141.4, 134.2, 132.4, 131.8, 129.5, 125.7, 123.4, 123.2, 122.4, 113.3, 55.5, 55.2, 54.2, 52.2, 43.0, 27.3, 24.5.
IR (KBr) 3695, 2966, 1777, 1718, 1508, 1462, 1389, 1330, 1282, 1250, 1184, 1112, 1038, 832, 728, 479 cm^{-1}.
HRMS (ESI-MS) *m/z* calcd for $C_{28}H_{28}N_2O_4$ $[M+H]^+$: 457.2122. found: 457.2115.

2-(2-(Tert-butyl)-10-(3,5-dimethylphenyl)-1-oxo-2-azaspiro[4.5]deca-7,9-dien-6-yl)isoindoline-1,3-dione (2x)

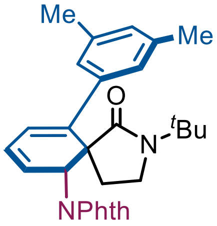

According to the general procedure **F**, using the corresponding substrate **1x** (0.2 mmol, 1.0 equiv), $Pd(PPh_3)_4$ (0.01 mmol, 5 mol%) , L_1 (0.02 mmol, 10 mol%) and stirring at room temperature for 48 h under 30 W blue LEDs irradiation. The reaction mixture was filtered through a pad of silica gel and concentrated in vacuo, then purified by chromatography (petroleum ether/ethyl acetate = 7/1) to give **2x** (87.3 mg, 0.192 mmol, 96%, d.r. > 20:1) as colorless oil.

^{1}H NMR (400 MHz, $CDCl_3$) δ 7.88 (dd, J = 5.5, 3.1 Hz, 2H), 7.75 (dd, J = 5.5, 3.0 Hz, 2H), 6.98 (s, 2H), 6.89 (s, 1H), 6.25 (ddd, J = 9.4, 5.7, 2.4 Hz, 1H), 6.12 (d, J = 5.7 Hz, 1H), 5.72 (dd, J = 9.3, 3.8 Hz, 1H), 5.66 (dd, J = 3.8, 2.5 Hz, 1H), 3.02 (td, J = 9.3, 3.6 Hz, 2H), 2.64 (ddd, J = 14.0, 7.8, 4.4 Hz, 1H), 2.28 (s, 6H), 2.05 (td, J = 8.1, 7.2, 6.0 Hz, 1H), 1.23 (s, 9H).
^{13}C NMR (101 MHz, $CDCl_3$) δ 174.1, 141.9, 139.9, 137.2, 134.2, 131.8, 129.0, 125.9, 125.7, 123.4, 123.3, 122.6, 55.3, 54.2, 52.3, 42.9, 27.3, 24.6, 21.3.
IR (KBr) 3693, 2967, 2916, 1775, 1720, 1464, 1384, 1331, 1288, 1102, 1042, 887, 850, 721cm^{-1}.
HRMS (ESI-MS) *m/z* calcd for $C_{29}H_{30}N_2O_3$ $[M+H]^+$: 455.2329. found: 455.2321.

2-(2-(Tert-butyl)-10-(3,5-difluorophenyl)-1-oxo-2-azaspiro[4.5]deca-7,9-dien-6-yl)isoindoline-1,3-dione (2y)

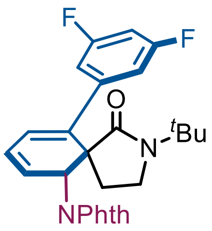

According to the general procedure **F**, using the corresponding substrate **1y** (0.2 mmol, 1.0 equiv), $Pd(PPh_3)_4$ (0.01 mmol, 5 mol%) , L_1 (0.02 mmol, 10 mol%) and stirring at room temperature for 48 h under 30 W blue LEDs irradiation. The reaction mixture was filtered through a pad of silica gel and concentrated in vacuo, then purified by chromatography (petroleum ether/ethyl acetate = 7/1) to give **2y** (74.0 mg, 0.160 mmol, 80%, d.r. > 20:1) as white solid.

M.p. 189.4 - 190.8 ℃.
^{1}H NMR (400 MHz, CDCl$_3$) δ 7.89 (dd, J = 5.4, 3.1 Hz, 2H), 7.77 (dd, J = 5.5, 3.0 Hz, 2H), 6.94 – 6.86 (m, 2H), 6.71 (tt, J = 8.9, 2.3 Hz, 1H), 6.26 (ddd, J = 9.3, 5.7, 2.6 Hz, 1H), 6.21 (d, J = 5.6 Hz, 1H), 5.81 (ddd, J = 9.4, 3.7, 1.0 Hz, 1H), 5.68 – 5.64 (m, 1H), 3.20 (td, J = 9.6, 3.1 Hz, 1H), 3.03 (dt, J = 9.9, 7.9 Hz, 1H), 2.79 (ddd, J = 14.3, 8.2, 3.0 Hz, 1H), 2.02 – 1.95 (m, 1H), 1.23 (s, 9H).
^{13}C NMR (101 MHz, CDCl$_3$) δ 173.6, 162.6 (dd, J = 247.9, 13.1 Hz), 143.13 (t, J = 9.8 Hz), 139.7, 134.4, 131.6, 125.2, 125.0, 124.5, 123.5, 110.7 (dd, J = 18.5, 7.1 Hz), 102.7 (t, J = 25.5 Hz), 55.4, 54.4, 52.2, 43.0, 27.2, 24.3.
^{19}F NMR (376 MHz, CDCl$_3$) δ -110.4.
IR (KBr) 3696, 2900, 1778, 1721, 1464, 1323, 1116, 994, 730 cm^{-1}.
HRMS (ESI-MS) *m/z* calcd for $C_{27}H_{24}F_2N_2O_3$ [M+H]$^+$: 463.1828. found: 463.1820.

2-(2-(Tert-butyl)-9-fluoro-1-oxo-2-azaspiro[4.5]deca-7,9-dien-6-yl)isoindoline-1,3-dione (2z)

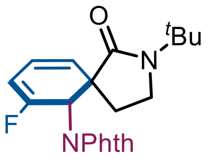

According to the general procedure **F**, using the corresponding substrate **1z** (0.2 mmol, 1.0 equiv), $Pd(PPh_3)_4$ (0.01 mmol, 5 mol%) , L_1 (0.02 mmol, 10 mol%) and stirring at room temperature for 48 h under 30 W blue LEDs irradiation. The reaction mixture was filtered through a pad of silica gel and concentrated in vacuo, then purified by chromatography (petroleum ether/ethyl acetate = 7/1) to give **2z** (44.2 mg, 0.120 mmol, 60%, d.r. > 20:1) as white solid.

M.p. 152.6-153.8 ℃.
^{1}H NMR (500 MHz, CDCl$_3$) δ 7.87 (dd, J = 5.4, 3.1 Hz, 2H), 7.77 (dd, J = 5.5, 3.1 Hz, 2H), 6.11 (tt, J = 7.1, 2.7 Hz, 1H), 5.75 (dt, J = 10.0, 4.8 Hz, 1H), 5.52 (d, J = 3.2 Hz, 1H), 5.19 (dd, J = 11.9, 2.2 Hz, 1H), 3.31 (dt, J = 9.9, 7.1 Hz, 1H), 3.11 (ddd, J = 10.0, 7.8, 5.2 Hz, 1H), 2.31 (dt, J = 13.6, 6.9 Hz, 1H), 1.81 – 1.76 (m, 1H), 1.32 (s, 9H).
^{13}C NMR (126 MHz, CDCl$_3$) δ 174.7 (d, J = 3.2 Hz), 167.6, 157.2 (d, J = 250.9 Hz), 134.4, 131.6, 126.9 (d, J = 9.4 Hz), 123.6, 122.1 (d, J = 36.8 Hz), 103.5 (d, J = 18.8 Hz), 54.4, 51.1, 51.0, 49.0, 42.3, 27.52.
^{19}F NMR (471 MHz, CDCl$_3$) δ -116.07.
IR (KBr) 3615, 3051, 2969, 1774, 1720, 1464, 1389, 1334, 1287, 974, 891, 823, 722, 532 cm^{-1}.
HRMS (ESI-MS) *m/z* calcd for $C_{21}H_{21}FN_2O_3$ [M+H]$^+$: 369.1609. found: 369.1603.

2-(2-(Tert-butyl)-8-fluoro-1-oxo-2-azaspiro[4.5]deca-7,9-dien-6-yl)isoindoline-1,3-dione (2aa)

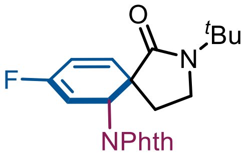

According to the general procedure **F**, using the corresponding substrate **1aa** (0.2 mmol, 1.0 equiv), $Pd(PPh_3)_4$ (0.01 mmol, 5 mol%) , L_1 (0.02 mmol, 10 mol%) and stirring at room temperature for 48 h under 30 W blue LEDs irradiation. The reaction mixture was filtered through a pad of silica gel and concentrated in vacuo, then purified by chromatography (petroleum ether/ethyl acetate = 7/1) to give **2aa** (31.7 mg, 0.086 mmol, 43%, d.r. > 20:1) as white solid.

M.p. 165.9 - 167.1 ℃.

^{1}H NMR (400 MHz, $CDCl_3$) δ 7.86 (dd, J = 5.5, 3.1 Hz, 2H), 7.76 (dd, J = 5.5, 3.0 Hz, 2H), 6.14 (ddd, J = 10.1, 7.0, 2.1 Hz, 1H), 5.80 (dd, J = 10.1, 5.7 Hz, 1H), 5.47 (t, J = 5.1 Hz, 1H), 5.08 (ddd, J = 11.5, 5.3, 2.0 Hz, 1H), 3.38 (dt, J = 10.0, 7.0 Hz, 1H), 3.27 (ddd, J = 10.0, 7.9, 4.5 Hz, 1H), 2.15 – 2.05 (m, 1H), 1.82 (ddd, J = 12.9, 7.2, 4.5 Hz, 1H), 1.38 (s, 9H).

^{13}C NMR (101 MHz, $CDCl_3$) δ 173.8, 167.6, 159.5 (d, J = 251.6 Hz), 134.4, 131.7, 131.1 (d, J = 10.1 Hz), 123.5, 120.8 (d, J = 36.2 Hz), 97.0 (d, J = 20.0 Hz), 54.5, 50.6, 48.2 (d, J = 11.0 Hz), 42.1, 27.6, 27.4.

^{19}F NMR (376 MHz, $CDCl_3$) δ -113.42.

IR (KBr) 3696, 2907, 1771, 1713, 1461, 1291, 937, 834, 753, 718, 525 cm^{-1}.

HRMS (ESI-MS) *m/z* calcd for $C_{21}H_{21}FN_2O_3$ $[M+H]^+$: 369.1609. found: 369.1603.

2-(2-(Tert-butyl)-8-methoxy-1-oxo-2-azaspiro[4.5]deca-7,9-dien-6-yl)isoindoline-1,3-dione (2ab)

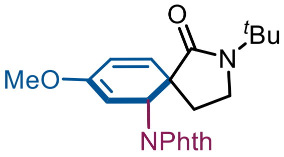

According to the general procedure **F**, using the corresponding substrate **1ab** (0.2 mmol, 1.0 equiv), $Pd(PPh_3)_4$ (0.01 mmol, 5 mol%) , L_1 (0.02 mmol, 10 mol%) and stirring at room temperature for 48 h under 30 W blue LEDs irradiation. The reaction mixture was filtered through a pad of silica gel and concentrated in vacuo, then purified by chromatography (petroleum ether/ethyl acetate = 7/1) to give **2ab** (9.9 mg, 0.026 mmol, 13%, d.r. > 20:1) as colorless oil.

^{1}H NMR (400 MHz, $CDCl_3$) δ 7.85 (dd, J = 5.4, 3.1 Hz, 2H), 7.75 (dd, J = 5.5, 3.0 Hz, 2H), 6.07 (dd, J = 10.0, 2.0 Hz, 1H), 5.75 (d, J = 9.9 Hz, 1H), 5.49 (d, J = 5.1 Hz, 1H), 4.51 (dd, J = 5.2, 2.0 Hz, 1H), 3.56 (s, 3H), 3.37 (dt, J = 9.9, 7.0 Hz, 1H), 3.25 (ddd, J = 9.9, 7.9, 4.5 Hz, 1H), 2.14 (dt, J = 12.7, 7.3 Hz, 1H), 1.80 (ddd, J = 12.3, 7.2, 4.5 Hz, 1H), 1.37 (s, 9H).

^{13}C NMR (101 MHz, $CDCl_3$) δ 174.5, 167.9, 155.5, 134.1, 131.9, 129.1, 124.4, 123.4, 88.7, 54.4, 54.3, 50.5, 49.2, 42.1, 27.6, 27.6.

IR (KBr) 3850, 3747, 2827, 1605, 1362, 1079, 767 cm^{-1}.

HRMS (ESI-MS) *m/z* calcd for $C_{22}H_{24}N_2O_4$ $[M+H]^+$: 381.1809. found: 381.1803.

2-(2-Isopropyl-1-oxo-2-azaspiro[4.5]deca-7,9-dien-6-yl)isoindoline-1,3-dione (2ac)

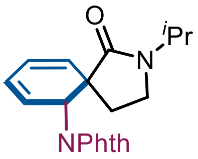

According to the general procedure **F**, using the corresponding substrate **1ac** (0.2 mmol, 1.0 equiv), $Pd(PPh_3)_4$ (0.01 mmol, 5 mol%) , L_1 (0.02 mmol, 10 mol%) and stirring at room temperature for 48 h under 30 W blue LEDs irradiation. The reaction mixture was filtered through a pad of silica gel and concentrated in vacuo, then purified by chromatography (petroleum ether/ethyl acetate = 7/1) to give **2ac** (13.5 mg, 0.040 mmol, 20%, d.r. > 20:1) as colorless oil.

^{1}H NMR (400 MHz, $CDCl_3$) *δ* 7.87 (dd, *J* = 5.5, 3.1 Hz, 2H), 7.76 (dd, *J* = 5.5, 3.0 Hz, 2H), 6.18 – 6.11 (m, 2H), 5.76 – 5.67 (m, 2H), 5.63 – 5.59 (m, 1H), 4.34 (h, *J* = 6.6 Hz, 1H), 3.21 (ddd, *J* = 9.8, 8.3, 4.8 Hz, 1H), 2.92 (ddd, *J* = 9.9, 7.8, 6.2 Hz, 1H), 2.59 (ddd, *J* = 12.8, 7.7, 4.7 Hz, 1H), 1.83 – 1.76 (m, 1H), 1.09 (d, *J* = 6.8 Hz, 3H), 0.91 (d, *J* = 6.8 Hz, 3H).
^{13}C NMR (101 MHz, $CDCl_3$) *δ* 174.7, 167.9, 134.3, 131.7, 128.6, 124.6, 123.8, 123.7, 123.5, 51.2, 50.2, 42.9, 39.1, 27.0, 19.7, 19.4.
IR (KBr) 3700, 2882, 1867, 1450, 1304, 1030, 752, 678 cm^{-1}.
HRMS (ESI-MS) *m/z* calcd for $C_{20}H_{20}N_2O_3$ $[M+H]^+$: 337.1547. found: 337.1540.

2-(2-((3S)-Adamantan-1-yl)-1-oxo-2-azaspiro[4.5]deca-7,9-dien-6-yl)isoindoline-1,3-dione (2ad)

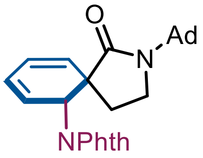

According to the general procedure **F**, using the corresponding substrate **1ad** (0.2 mmol, 1.0 equiv), $Pd(PPh_3)_4$ (0.01 mmol, 5 mol%) , L_1 (0.02 mmol, 10 mol%) and stirring at room temperature for 24 h under 30 W blue LEDs irradiation. The reaction mixture was filtered through a pad of silica gel and concentrated in vacuo, then purified by chromatography (petroleum ether/ethyl acetate = 7/1) to give **2ad** (78.8 mg, 0.184 mmol, 92%, d.r. > 20:1) as colorless oil.

^{1}H NMR (400 MHz, $CDCl_3$) *δ* 7.85 (dd, *J* = 5.5, 3.0 Hz, 2H), 7.75 (dd, *J* = 5.5, 3.0 Hz, 2H), 6.18 – 6.06 (m, 2H), 5.74 – 5.66 (m, 1H), 5.66 – 5.57 (m, 1H), 5.50 (dd, *J* = 4.1, 2.0 Hz, 1H), 3.34 (ddd, *J* = 9.9, 7.7, 6.0 Hz, 1H), 3.13 (ddd, *J* = 9.9, 7.8, 5.2 Hz, 1H), 2.27 (ddd, *J* = 13.5, 7.8, 6.0 Hz, 1H), 2.04 (s, 9H), 1.74 (ddd, *J* = 13.0, 7.7, 5.2 Hz, 1H), 1.63 (s, 6H).
^{13}C NMR (101 MHz, $CDCl_3$) *δ* 175.2, 167.8, 134.2, 131.8, 128.2, 125.1, 123.5, 123.4, 122.8, 55.4, 51.5, 49.2, 41.4, 39.4, 36.3, 29.5, 27.2.
IR (KBr) 3948, 3667, 3343, 3051, 2910, 2857, 2673, 2551, 2421, 2140, 1775, 1720, 1456, 1384, 1324, 1259, 1197, 1099, 1049, 978, 894, 807, 731, 608, 573, 533, 465 cm^{-1}.
HRMS (ESI-MS) *m/z* calcd for $C_{27}H_{28}N_2O_3$ $[M+H]^+$: 429.2173. found: 429.2164.

Tert-butyl 2-(10-(1,3-Dioxoisoindolin-2-yl)-1-oxo-2-azaspiro[4.5]deca-6,8-dien-2-yl)-2-methylpropanoate (2ae)

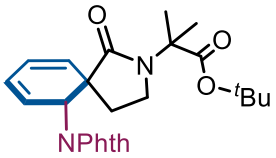

According to the general procedure **F**, using the corresponding substrate **1ae** (0.2 mmol, 1.0 equiv), $Pd(PPh_3)_4$ (0.01 mmol, 5 mol%) , L_1 (0.02 mmol, 10 mol%) and stirring at room temperature for 48 h under 30 W blue LEDs irradiation. The reaction mixture was filtered through a pad of silica gel and concentrated in vacuo, then purified by chromatography (petroleum ether/ethyl acetate = 7/1) to give **2ae** (34.9 mg, 0.080 mmol, 40%, d.r. > 20:1) as white solid.

M.p. 141.5 - 143.2 ℃.

^{1}H NMR (400 MHz, CDCl$_3$) δ 7.85 (dd, J = 5.5, 3.1 Hz, 2H), 7.74 (dd, J = 5.5, 3.0 Hz, 2H), 6.18 – 6.11 (m, 2H), 5.76 – 5.71 (m, 1H), 5.64 – 5.59 (m, 1H), 5.53 (dd, J = 4.0, 2.0 Hz, 1H), 3.41 (dt, J = 9.3, 7.1 Hz, 1H), 3.21 (ddd, J = 9.4, 7.9, 4.6 Hz, 1H), 2.35 (ddd, J = 13.0, 7.9, 6.5 Hz, 1H), 1.91 – 1.87 (m, 1H), 1.43 (s, 9H), 1.39 (s, 6H).

^{13}C NMR (101 MHz, CDCl$_3$) δ 174.3, 172.5, 167.7, 134.2, 131.8, 127.7, 125.2, 123.6, 123.4, 122.9, 59.1, 50.1, 48.7, 41.7, 28.1, 27.8, 23.8, 23.5.

IR (KBr) 3609, 3484, 3049, 2983, 2933, 1728, 1462, 1388, 1280, 1149, 968, 900, 852, 798, 730, 616, 469 cm^{-1}.

HRMS (ESI-MS) *m/z* calcd for $C_{25}H_{28}N_2O_5$ $[M+H]^+$: 437.2071. found: 437.2063.

2-(2-(Tert-butyl)-4-methyl-1-oxo-2-azaspiro[4.5]deca-7,9-dien-6-yl)isoindoline-1,3-dione (2af)

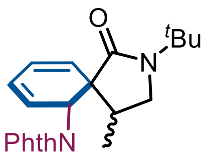

According to the general procedure **F**, using the corresponding substrate **1af** (0.2 mmol, 1.0 equiv), $Pd(PPh_3)_4$ (0.01 mmol, 5 mol%) , L_1 (0.02 mmol, 10 mol%) and stirring at room temperature for 24 h under 30 W blue LEDs irradiation. The reaction mixture was filtered through a pad of silica gel and concentrated in vacuo, then purified by chromatography (petroleum ether/ethyl acetate = 7/1) to give **2af** (67.8 mg, 0.186 mmol, 93%, d.r. = 1.5:1:0:0) as colorless oil.

Diastereoisomer 1:

^{1}H NMR (400 MHz, CDCl$_3$) δ 7.85 (dd, J = 5.4, 3.1 Hz, 2H), 7.74 (dd, J = 5.5, 3.0 Hz, 2H), 6.19 – 6.08 (m, 2H), 5.91 – 5.83 (m, 1H), 5.68 – 5.61 (m, 1H), 5.39 (d, J = 6.0 Hz, 1H), 3.69 (dd, J = 10.0, 5.5 Hz, 1H), 2.93 (dd, J = 10.0, 2.2 Hz, 1H), 2.27 – 2.18 (m, 1H), 1.37 (s, 9H), 1.03 (d, J = 6.9 Hz, 3H).

^{13}C NMR (101 MHz, CDCl$_3$) δ 174.3, 167.7, 134.2, 132.0, 126.2, 125.5, 123.4, 123.2, 121.7, 54.0, 52.7, 50.5, 45.8, 35.6, 27.6, 15.9.

IR (KBr) 3695, 2918, 1772, 1716, 1390, 1317, 1106, 961, 798, 752, 723. cm^{-1}.

HRMS (ESI-MS) *m/z* calcd for $C_{22}H_{24}N_2O_3$ $[M+H]^+$: 365.1860. found: 365.1854.

Diastereoisomer 2:

^{1}H NMR (400 MHz, CDCl$_3$) δ 7.88 (dd, J = 5.4, 3.0 Hz, 2H), 7.75 (dd, J = 5.5, 3.1 Hz, 2H), 6.22 (dd, J = 9.8, 5.3 Hz, 1H), 6.08 (dddd, J = 9.5, 5.4, 3.0, 1.0 Hz, 1H), 5.85 (t, J = 3.2 Hz, 1H), 5.71 (ddt, J = 9.6, 3.3, 0.9 Hz, 1H), 5.58 (d, J = 9.8 Hz, 1H), 3.25 (dd, J = 9.7, 7.7 Hz, 1H), 2.89 (dd, J = 9.7, 7.9 Hz, 1H), 2.37 (h, J = 7.2 Hz, 1H), 1.33 (s, 9H), 1.05 (d, J = 6.8 Hz, 3H).

^{13}C NMR (101 MHz, CDCl$_3$) δ 175.1, 168.0, 134.2, 131.8, 125.5, 125.5, 124.5, 124.0, 123.4, 54.7, 54.1, 50.3, 49.6, 32.4, 27.6, 14.3.

IR (KBr) 3694, 2925, 1777, 1719, 1464, 1387, 1330, 1255, 1094, 1032, 969, 886, 831, 729, 622, 532, 473 cm^{-1}.

HRMS (ESI-MS) *m/z* calcd for $C_{22}H_{24}N_2O_3$ $[M+H]^+$: 365.1860. found: 365.1853.

2-(1'-(Tert-butyl)-2'-oxo-2H-spiro[naphthalene-1,3'-pyrrolidin]-2-yl)isoindoline-1,3-dione (2ag)

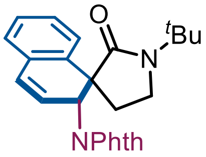

According to the general procedure **F**, using the corresponding substrate **1ag** (0.2 mmol, 1.0 equiv), $Pd(PPh_3)_4$ (0.01 mmol, 5 mol%) , L_1 (0.02 mmol, 10 mol%) and stirring at room temperature for 48 h under 30 W blue LEDs irradiation. The reaction mixture was filtered through a pad of silica gel and concentrated in vacuo, then purified by chromatography (petroleum ether/ethyl acetate = 7/1) to give **2ag** (40.0 mg, 0.100 mmol, 50%, d.r. = 0.75: 1) as clear oil.

^{1}H NMR (400 MHz, $CDCl_3$) diastereoisomer 1: δ 7.87 (dd, J = 5.5, 3.1 Hz, 2H), 7.75 (dd, J = 5.5, 3.1 Hz, 2H), 7.35 – 7.02 (m, 4H), 6.64 (dd, J = 9.7, 2.8 Hz, 1H), 5.92 (dd, J = 9.7, 3.1 Hz, 1H), 5.70 (t, J = 3.0 Hz, 1H), 3.39 (td, J = 9.1, 4.1 Hz, 1H), 3.11 (ddd, J = 9.6, 8.2, 6.5 Hz, 1H), 3.00 (ddd, J = 13.7, 8.1, 4.1 Hz, 1H), 1.92 (ddd, J = 13.6, 8.8, 6.5 Hz, 1H), 1.30 (s, 9H). **diastereoisomer 2:** δ 7.81 (dd, J = 5.5, 3.1 Hz, 2H), 7.71 (dd, J = 5.5, 3.1 Hz, 2H), 7.35 – 7.02 (m, 4H), 6.12 (dd, J = 3.9, 1.9 Hz, 1H), 6.07 (dd, J = 9.9, 1.9 Hz, 1H), 5.93 (dd, J = 9.9, 3.8 Hz, 1H), 3.78 – 3.62 (m, 2H), 2.61 (td, J = 6.7, 5.9, 1.7 Hz, 2H), 1.50 (s, 9H).

^{13}C NMR (101 MHz, $CDCl_3$) δ 175.9, 174.5, 167.7, 138.9, 138.9, 134.3, 134.1, 132.6, 132.2, 132.0, 131.9, 131.7, 128.8, 128.4, 127.3, 127.3, 127.2, 127.0, 126.2, 125.3, 125.2, 123.5, 123.5, 123.3, 55.7, 54.6, 54.4, 52.3, 51.8, 46.4, 43.2, 34.6, 28.6, 27.7, 27.5.

IR (KBr) 3692, 2882, 1774, 1719, 1459, 1411, 1284, 1112, 893, 797, 752, 536 cm^{-1}.

HRMS (ESI-MS) *m/z* calcd for $C_{25}H_{24}N_2O_3$ $[M+H]^+$: 401.1860. found: 401.1855.

2-(1-(Tert-butyl)-2-oxo-7'H-spiro[pyrrolidine-3,8'-quinolin]-7'-yl)isoindoline-1,3-dione (2ah)

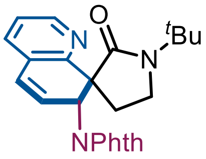

According to the general procedure **F**, using the corresponding substrate **1ah** (0.2 mmol, 1.0 equiv), $Pd(PPh_3)_4$ (0.01 mmol, 5 mol%) , L_1 (0.02 mmol, 10 mol%) and stirring at room temperature for 48 h under 30 W blue LEDs irradiation. The reaction mixture was filtered through a pad of silica gel and concentrated in vacuo, then purified by chromatography (petroleum ether/ethyl acetate = 2/1) to give **2ah** (56.2 mg, 0.140 mmol, 70%, d.r. > 20: 1) as colorless oil.

^{1}H NMR (400 MHz, $CDCl_3$) δ 8.40 (dd, J = 4.9, 1.7 Hz, 1H), 7.96 – 7.61 (m, 4H), 7.45 (dd, J = 7.6, 1.7 Hz, 1H), 7.18 (ddd, J = 7.5, 5.0, 1.0 Hz, 1H), 6.74 (dd, J = 9.6, 1.3 Hz, 1H), 5.85 (dd, J = 9.6, 5.4 Hz, 1H), 5.39 (dd, J = 5.4, 1.3 Hz, 1H), 3.96 – 3.82 (m, 1H), 3.42 (td, J = 8.8, 3.3 Hz, 1H), 2.76 (ddd, J = 12.5, 7.7, 3.3 Hz, 1H), 2.06 – 1.98 (m, 1H), 1.34 (s, 9H).

^{13}C NMR (101 MHz, $CDCl_3$) δ 174.0, 167.6, 156.3, 147.9, 134.3, 133.8, 131.7, 129.7, 127.9, 123.5, 122.3, 122.1, 54.4, 53.9, 49.6, 44.0, 27.5, 26.1.

IR (KBr) 3670, 3051, 2972, 2925, 2791, 1773, 1720, 1559, 1392, 1329, 1255, 1115, 1041, 986, 942, 889, 818, 784, 732, 624, 531, 465 cm^{-1}.

HRMS (ESI-MS) *m/z* calcd for $C_{24}H_{23}N_3O_3$ $[M+H]^+$: 402.1812. found: 402.1807.

According to the general procedure **F**, using the corresponding substrate **1ai** (0.2 mmol, 1.0 equiv), $Pd(PPh_3)_4$ (0.01 mmol, 5 mol%) , L_1 (0.02 mmol, 10 mol%) and stirring at room temperature for 48 h under 30 W blue LEDs irradiation. The reaction mixture was filtered

through a pad of silica gel and concentrated in vacuo, then purified by chromatography (petroleum ether/ethyl acetate = 7/1) to give **2ai** (14.6 mg, 0.040 mmol, 20%, d.r. > 20:1) and **2ai'** (29.2 mg, 0.080 mmol, 40%, d.r. > 20:1) as colorless oil.

2-(2-(Tert-butyl)-9-methyl-1-oxo-2-azaspiro[4.5]deca-7,9-dien-6-yl)isoindoline-1,3-dione (2ai)

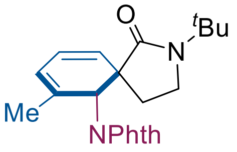

^{1}H NMR (400 MHz, $CDCl_3$) δ 7.85 (dd, J = 5.5, 3.1 Hz, 2H), 7.75 (dd, J = 5.5, 3.0 Hz, 2H), 6.00 (dt, J = 10.0, 2.5 Hz, 1H), 5.72 (dd, J = 9.9, 3.5 Hz, 1H), 5.49 (t, J = 3.1 Hz, 1H), 4.61 (d, J = 2.2 Hz, 1H), 3.60 (s, 3H), 3.29 (ddd, J = 9.8, 8.2, 4.7 Hz, 1H), 3.00 (ddd, J = 9.9, 7.6, 6.4 Hz, 1H), 2.52 (ddd, J = 12.7, 7.6, 4.7 Hz, 1H), 1.73 (ddd, J = 13.2, 8.2, 6.4 Hz, 1H), 1.27 (s, 9H).

^{13}C NMR (101 MHz, $CDCl_3$) δ 175.8, 167.9, 134.2, 131.8, 131.4, 129.0, 123.4, 123.0, 123.0, 54.2, 51.7, 49.8, 42.6, 27.6, 27.3, 21.4.

IR (KBr) 3636, 3475, 2925, 1775, 1719, 1458, 1388, 1334, 1286, 1252, 1099, 1043, 798, 726, 531 cm^{-1}.

HRMS (ESI-MS) *m/z* calcd for $C_{22}H_{24}N_2O_3$ $[M+H]^+$: 365.1860. found: 365.1855.

2-(2-(Tert-butyl)-7-methyl-1-oxo-2-azaspiro[4.5]deca-6,9-dien-8-yl)isoindoline-1,3-dione (2ai')

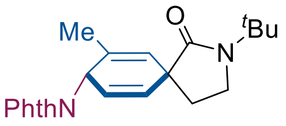

^{1}H NMR (400 MHz, $CDCl_3$) δ 7.84 (dd, J = 5.4, 3.1 Hz, 2H), 7.70 (dd, J = 5.5, 3.0 Hz, 2H), 5.88 (dt, J = 9.9, 2.1 Hz, 1H), 5.74 (dd, J = 9.9, 3.0 Hz, 1H), 5.38 (q, J = 2.3 Hz, 1H), 4.83 (d, J = 1.7 Hz, 1H), 3.54 (s, 3H), 3.49 (dd, J = 7.4, 6.1 Hz, 2H), 2.07 – 2.00 (m, 2H), 1.45 (s, 9H).

^{13}C NMR (101 MHz, $CDCl_3$) δ 174.2, 167.6, 134.0, 131.9, 130.9, 130.2, 126.1, 125.1, 123.4, 54.3, 49.6, 47.7, 41.9, 33.9, 27.8, 19.9.

IR (KBr) 3616, 3473, 3349, 2926, 2863, 2801, 1772, 1718, 1456, 1390, 1332, 1278, 1105, 910, 860, 728, 664, 610, 533 cm^{-1}.

HRMS (ESI-MS) *m/z* calcd for $C_{22}H_{24}N_2O_3$ $[M+H]^+$: 365.1860. found: 365.1853.

According to the general procedure **F**, using the corresponding substrate **1aj** (0.2 mmol, 1.0 equiv), $Pd(PPh_3)_4$ (0.01 mmol, 5 mol%) , L_1 (0.02 mmol, 10 mol%) and stirring at room temperature for 48 h under 30 W blue LEDs irradiation. The reaction mixture was filtered through a pad of silica gel and concentrated in vacuo, then purified by chromatography (petroleum ether/ethyl acetate = 7/1) to give **2aj** (30.4 mg, 0.080 mmol, 40%, d.r. > 20:1) as colorless oil and **2aj'** (15.2 mg, 0.040mmol, 20%, d.r. > 20:1) as white solid.

2-(2-(Tert-butyl)-9-methoxy-1-oxo-2-azaspiro[4.5]deca-7,9-dien-6-yl)isoindoline-1,3-dione (2aj)

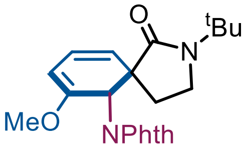

^{1}H NMR (400 MHz, $CDCl_3$) δ 7.86 (dd, J = 5.4, 3.1 Hz, 2H), 7.75 (dd, J = 5.5, 3.1 Hz, 2H), 6.03 (ddd, J = 9.6, 2.6, 1.5 Hz, 1H), 5.65 (dd, J = 9.6, 3.8 Hz, 1H), 5.54 – 5.47 (m, 1H), 5.40 (d, J = 2.3 Hz, 1H), 3.32 (ddd, J = 9.9, 7.9, 5.5 Hz, 1H), 3.07 (ddd, J = 9.8, 7.8, 5.7 Hz, 1H), 2.36 (ddd, J = 13.2, 7.8, 5.5 Hz, 1H), 1.88 (s, 3H), 1.77 – 1.71 (m, 1H), 1.31 (s, 9H).

^{13}**C NMR (101 MHz, CDCl$_3$)** δ 176.0, 167.8, 153.3, 134.3, 131.7, 125.7, 125.3, 123.4, 96.5, 54.5, 54.2, 51.7, 50.5, 42.7, 28.1, 27.5.

IR (KBr) 2960, 2828, 1774, 1717, 1606, 1459, 1364, 1286, 1222, 1165, 1092, 885, 777, 725, 537 cm^{-1}.

HRMS (ESI-MS) *m/z* calcd for $C_{22}H_{24}N_2O_4$ [M+H]$^+$: 381.1809. found: 381.1805.

2-(2-(Tert-butyl)-7-methoxy-1-oxo-2-azaspiro[4.5]deca-6,9-dien-8-yl)isoindoline-1,3-dione (2aa')

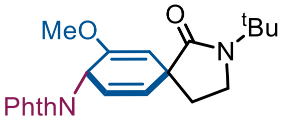

M.p. 193.9-195.1 ℃.

^{1}**H NMR (400 MHz, CDCl$_3$)** δ 7.85 (dd, J = 5.5, 3.0 Hz, 2H), 7.71 (dd, J = 5.5, 3.0 Hz, 2H), 5.89 (dt, J = 9.9, 2.3 Hz, 1H), 5.78 (dd, J = 9.9, 2.8 Hz, 1H), 5.64 (q, J = 1.9 Hz, 1H), 5.23 (tt, J = 2.4, 1.1 Hz, 1H), 3.49 (td, J = 6.9, 1.4 Hz, 2H), 1.99 (t, J = 7.0 Hz, 2H), 1.68 (s, 3H), 1.44 (s, 9H).

^{13}**C NMR (101 MHz, CDCl$_3$)** δ 174.6, 167.5, 151.4, 133.8, 132.1, 130.3, 124.2, 123.3, 98.7, 54.5, 54.3, 50.2, 45.7, 41.9, 34.8, 27.8.

IR (KBr) 2822, 1715, 1602, 1456, 1364, 1268, 1227, 1163, 1089, 775, 721, 535 cm^{-1}.

HRMS (ESI-MS) *m/z* calcd for $C_{22}H_{24}N_2O_4$ [M+H]$^+$: 381.1809. found: 381.1806.

According to the general procedure **F**, using the corresponding substrate **1ak** (0.2 mmol, 1.0 equiv), Pd(PPh$_3$)$_4$ (0.01 mmol, 5 mol%) , L$_1$ (0.02 mmol, 10 mol%) and stirring at room temperature for 48 h under 30 W blue LEDs irradiation. The reaction mixture was filtered through a pad of silica gel and concentrated in vacuo, then purified by chromatography (petroleum ether/ethyl acetate = 7/1) to give **2ak** (34.5 mg, 0.078 mmol, 39%, d.r. > 20:1) and **2ak'** (28.3 mg, 0.064 mmol, 32%, d.r. > 20:1) as colorless oil.

2-(2-(Tert-butyl)-1-oxo-10-phenoxy-2-azaspiro[4.5]deca-7,9-dien-6-yl)isoindoline-1,3-dione (2ak)

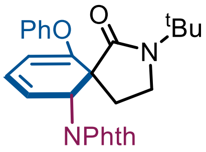

^{1}**H NMR (400 MHz, CDCl$_3$)** δ 7.88 (dd, J = 5.5, 3.1 Hz, 2H), 7.76 (dd, J = 5.5, 3.0 Hz, 2H), 7.40 – 7.33 (m, 2H), 7.23 – 7.12 (m, 3H), 6.04 – 5.96 (m, 1H), 5.58 (dd, J = 4.4, 2.0 Hz, 1H), 5.33 (dd, J = 9.5, 4.4 Hz, 1H), 5.08 (d, J = 6.5 Hz, 1H), 3.53 – 3.46 (m, 1H), 3.21 (td, J = 8.8, 4.1 Hz, 1H), 2.35 (ddd, J = 12.3, 7.9, 4.1 Hz, 1H), 2.24 (ddd, J = 13.2, 8.5, 6.7 Hz, 1H), 1.37 (s, 9H).

^{13}**C NMR (101 MHz, CDCl$_3$)** δ 173.7, 167.8, 158.9, 154.8, 134.3, 131.8, 129.7, 124.8, 124.6, 123.5, 121.1, 115.8, 99.8, 54.5, 53.6, 51.7, 43.8, 27.5, 24.5.

IR (KBr) 3696, 2914, 1776, 1719, 1484, 1411, 1324, 1222, 1115, 846, 798, 729, 490 cm^{-1}.

HRMS (ESI-MS) *m/z* calcd for $C_{27}H_{26}N_2O_4$ [M+H]$^+$: 443.1965. found: 443.1958.

2-(2-(Tert-butyl)-1-oxo-6-phenoxy-2-azaspiro[4.5]deca-6,9-dien-8-yl)isoindoline-1,3-dione (2ak')

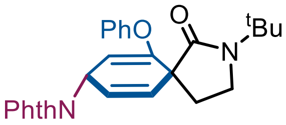

^{1}**H NMR (400 MHz, CDCl$_3$)** δ 7.79 (dd, J = 5.4, 3.1 Hz, 2H), 7.67 (dd, J = 5.4, 3.1 Hz, 2H), 7.32 – 7.26 (m, 2H), 7.18 – 7.12 (m, 2H), 7.07 (tt, J = 7.3, 1.2 Hz, 1H), 5.91 – 5.79 (m, 2H), 5.50 (td, J = 3.0, 2.0 Hz, 1H), 4.72 (dd, J = 3.3, 1.5 Hz, 1H), 3.61 (dt, J = 9.1, 7.7 Hz,

1H), 3.52 (td, J = 8.9, 3.5 Hz, 1H), 2.53 (ddd, J = 13.0, 7.9, 3.5 Hz, 1H), 2.12 – 2.03 (m, 1H), 1.41 (s, 9H).

^{13}C NMR (101 MHz, $CDCl_3$) δ 172.5, 167.3, 158.2, 154.9, 133.8, 132.0, 130.8, 129.6, 124.5, 124.2, 123.2, 121.0, 100.7, 54.5, 51.0, 46.4, 43.6, 31.5, 27.5.

IR (KBr) 3692, 2899, 1773, 1715, 1484, 1391, 1291, 1220, 1116, 924, 798, 725 cm^{-1}.

HRMS (ESI-MS) *m/z* calcd for $C_{27}H_{26}N_2O_4$ [M+H]$^+$: 443.1965. found: 443.1959.

According to the general procedure **F**, using the corresponding substrate **1al** (0.2 mmol, 1.0 equiv), $Pd(PPh_3)_4$ (0.01 mmol, 5 mol%) , L_1 (0.02 mmol, 10 mol%) and stirring at room temperature for 48 h under 30 W blue LEDs irradiation. The reaction mixture was filtered through a pad of silica gel and concentrated in vacuo, then purified by chromatography (petroleum ether/ethyl acetate = 7/1) to give **2al** (41.8 mg, 0.110 mmol, 55%, d.r. > 20:1) and **2al'** (19.0 mg, 0.050 mmol, 25%, d.r. > 20:1) as colorless oil.

2-(2-(Tert-butyl)-10-methoxy-1-oxo-2-azaspiro[4.5]deca-7,9-dien-6-yl)isoindoline-1,3-dione (2al)

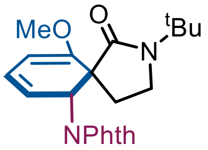

^{1}H NMR (500 MHz, $CDCl_3$) δ 7.85 (dd, J = 5.4, 3.0 Hz, 2H), 7.73 (dd, J = 5.5, 3.0 Hz, 2H), 6.12 (ddd, J = 9.1, 6.5, 2.1 Hz, 1H), 5.51 (dd, J = 4.1, 2.2 Hz, 1H), 5.29 (dd, J = 9.4, 3.9 Hz, 1H), 5.19 (d, J = 6.5 Hz, 1H), 3.66 (s, 3H), 3.37 (td, J = 8.7, 5.7 Hz, 1H), 3.09 (td, J = 8.7, 5.3 Hz, 1H), 2.26 (ddd, J = 13.9, 8.4, 5.8 Hz, 1H), 2.11 (ddd, J = 13.4, 8.2, 5.4 Hz, 1H), 1.31 (s, 9H).

^{13}C NMR (126 MHz, $CDCl_3$) δ 173.7, 167.8, 159.6, 134.2, 131.8, 125.2, 123.4, 114.9, 93.4, 55.5, 54.6, 54.4, 52.0, 43.6, 27.5, 24.4.

IR (KBr) 3054, 2961, 2836, 2716, 1775, 1715, 1684, 1597, 1460, 1362, 1287, 1248, 1084, 1017, 777, 729, 533 cm^{-1}.

HRMS (ESI-MS) *m/z* calcd for $C_{22}H_{24}N_2O_4$ [M+H]$^+$: 381.1809. found: 381.1801.

2-(2-(Tert-butyl)-6-methoxy-1-oxo-2-azaspiro[4.5]deca-6,9-dien-8-yl)isoindoline-1,3-dione (2al')

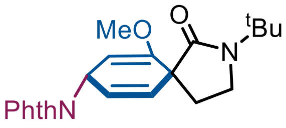

^{1}H NMR (500 MHz, $CDCl_3$) δ 7.81 (dd, J = 5.4, 2.7 Hz, 2H), 7.68 (dt, J = 5.1, 2.1 Hz, 2H), 5.84 – 5.77 (m, 2H), 5.55 (q, J = 2.4 Hz, 1H), 4.72 – 4.69 (m, 1H), 3.56 – 3.40 (m, 5H), 2.28 (ddd, J = 12.6, 8.0, 3.8 Hz, 1H), 1.92 (dt, J = 12.5, 7.9 Hz, 1H), 1.42 (s, 9H).

^{13}C NMR (126 MHz, $CDCl_3$) δ 173.1, 167.5, 158.4, 133.8, 132.2, 130.6, 124.3, 123.2, 93.1, 54.6, 54.4, 51.0, 46.6, 43.4, 31.5, 27.6.

IR (KBr) 2963, 2835, 2717, 1767, 1602, 1460, 1362, 1290, 1215, 1111, 917, 831, 775, 726, 538 cm^{-1}.

HRMS (ESI-MS) *m/z* calcd for $C_{22}H_{24}N_2O_4$ [M+H]$^+$: 381.1809. found: 381.1801.

2-(7-(Tert-butyl)-6-oxo-1-oxa-7-azaspiro[4.4]non-3-en-2-yl)isoindoline-1,3-dione (2am')

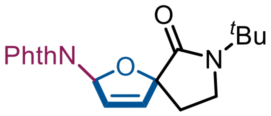

According to the general procedure **F**, using the corresponding substrate **1am** (0.2 mmol, 1.0 equiv), $Pd(PPh_3)_4$ (0.01 mmol, 5 mol%) , L_1 (0.02 mmol, 10 mol%) and stirring at room temperature for 48 h under 30 W blue LEDs irradiation. The reaction mixture was filtered

through a pad of silica gel and concentrated in vacuo, then purified by chromatography (petroleum ether/ethyl acetate = 2/1) to give **2am’** (62.6 mg, 0.184 mmol, 92%, d.r. > 20: 1) as white solid.

M.p. 196.7 - 197.8 ℃.
^{1}H NMR (400 MHz, $CDCl_3$) δ 7.88 (dd, J = 5.5, 3.1 Hz, 2H), 7.72 (dd, J = 5.5, 3.1 Hz, 2H), 6.66 (t, J = 1.8 Hz, 1H), 6.30 (dd, J = 6.0, 1.4 Hz, 1H), 6.11 (dd, J = 6.0, 2.2 Hz, 1H), 3.49 (ddd, J = 9.7, 8.3, 4.0 Hz, 1H), 3.36 (dt, J = 9.7, 7.1 Hz, 1H), 2.30 (ddd, J = 13.1, 8.4, 6.9 Hz, 1H), 2.15 (ddd, J = 13.1, 7.3, 4.0 Hz, 1H), 1.38 (s, 9H).
^{13}C NMR (101 MHz, $CDCl_3$) δ 170.8, 167.4, 134.1, 132.2, 132.0, 131.4, 126.2, 123.7, 93.4, 85.9, 54.5, 41.1, 31.3, 27.5.
IR (KBr) 3696, 2918, 1779, 1724, 1465, 1407, 1314, 1108, 1016, 874, 723, 542 cm^{-1}.
HRMS (ESI-MS) *m/z* calcd for $C_{19}H_{20}N_2O_4$ $[M+H]^+$: 341.1496. found: 341.1492.

2-(7-(Tert-butyl)-4-methyl-6-oxo-1-oxa-7-azaspiro[4.4]non-3-en-2-yl)isoindoline-1,3-dione (2an)

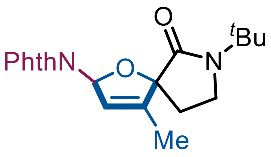

According to the general procedure **F**, using the corresponding substrate **1an** (0.2 mmol, 1.0 equiv), $Pd(PPh_3)_4$ (0.01 mmol, 5 mol%) , L_1 (0.02 mmol, 10 mol%) and stirring at room temperature for 48 h under 30 W blue LEDs irradiation. The reaction mixture was filtered through a pad of silica gel and concentrated in vacuo, then purified by chromatography (petroleum ether/ethyl acetate = 2/1) to give **2an’** (35.4 mg, 0.100 mmol, 50%, d.r. > 20: 1) as colorless oil.

^{1}H NMR (400 MHz, $CDCl_3$) δ 7.86 (dd, J = 5.4, 3.1 Hz, 2H), 7.71 (dd, J = 5.5, 3.1 Hz, 2H), 6.58 (t, J = 1.8 Hz, 1H), 5.84 (t, J = 1.7 Hz, 1H), 3.50 (dt, J = 9.2, 4.8 Hz, 1H), 3.35 (dd, J = 9.7, 7.4 Hz, 1H), 2.32 – 2.21 (m, 2H), 1.85 (s, 3H), 1.39 (s, 9H).
^{13}C NMR (101 MHz, $CDCl_3$) δ 170.7, 167.5, 141.3, 134.0, 132.1, 123.5, 112.0, 94.0, 85.2, 54.6, 41.4, 29.8, 27.5, 27.4, 11.6.
IR (KBr) 3922, 3606, 3486, 3437, 3069, 2972, 1777, 1721, 1451, 1406, 1298, 1166, 1113, 1072, 982, 936, 810, 722, 633, 534 cm^{-1}.
HRMS (ESI-MS) *m/z* calcd for $C_{20}H_{22}N_2O_4$ $[M+H]^+$: 355.1652. found: 355.1645.

5.3 Failed substrates

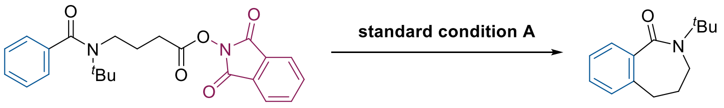

1ao **2ao**, 44%

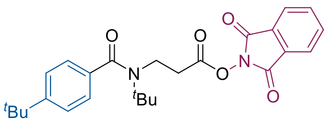

1ap, n.d.

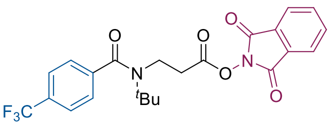

1aq, n.d.

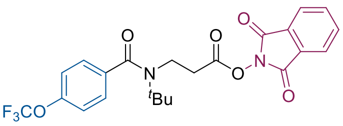

1ar, n.d.

6 General procedure for the synthesis of trieneylation products and characterization data.

6.1 General procedure G for the synthesis of trieneylation products

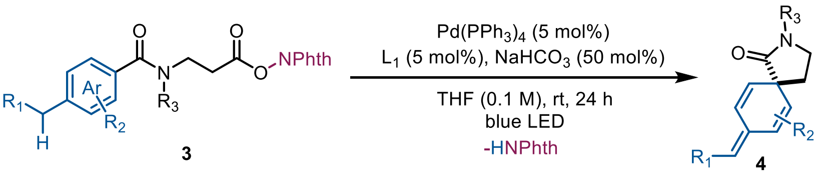

In a Schlenk tube with a magnetic bar under Ar atmosphere was added $Pd(PPh_3)_4$ (5 mol %, 11.5 mg), L_1 (5 mol%, 5.8 mg), $NaHCO_3$ (50 mol%, 8.4 mg) and then the substrates **3** (0.2 mmol) were added in THF (2 mL). The mixture was stirred at room temperature under Ar atmosphere and under blue LEDs (30 W×2) irradition for 24 h until the starting material was completely consumed (monitored by TLC). The corresponding reaction mixture was filtered through a pad of celite, washed with EtOAc and concentrated under reduced pressure. The residue was purified by flash chromatography on silica gel using petroleum ether/ethyl acetate as eluent to afford the desired products.

6.2 Characterization of trieneylation products

2-(Tert-butyl)-8-methylene-2-azaspiro[4.5]deca-6,9-dien-1-one (4a)

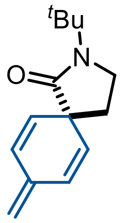

According to the general procedure **G**, using the corresponding substrate **3a** (0.2 mmol, 1.0 equiv), $Pd(PPh_3)_4$ (0.01 mmol, 5 mol%) , L_1 (0.01 mmol, 5 mol%), $NaHCO_3$ (50 mol%, 8.4 mg) and stirring at room temperature for 24 h under 30 W blue LEDs irradiation. The reaction mixture was filtered through a pad of silica gel and concentrated in vacuo, then purified by chromatography (petroleum ether/ethyl acetate = 12/1) to give **4a** (32.6 mg, 0.150 mmol, 75%) as white solid.

M.p. 90.4 - 92.1 ℃.
^{1}H NMR (400 MHz, CDCl$_3$) δ 6.38 (d, J = 9.9 Hz, 2H), 5.68 (d, J = 10.0 Hz, 2H), 4.96 (s, 2H), 3.49 (t, J = 6.8 Hz, 2H), 2.08 – 1.99 (m, 2H), 1.41 (s, 9H).
^{13}C NMR (101 MHz, CDCl$_3$) δ 174.1, 136.8, 129.5, 128.8, 114.2, 54.4, 51.6, 42.1, 33.4, 27.6.
IR (KBr) 3025, 2966, 2926, 1682, 1453, 1400, 1280, 1222, 874, 800, 687, 561 cm^{-1}.
HRMS (ESI-MS) *m/z* calcd for $C_{14}H_{19}NO$ $[M+H]^+$: 218.1539. found: 218.1538.

2-(Tert-butyl)-7-fluoro-8-methylene-2-azaspiro[4.5]deca-6,9-dien-1-one (4b)

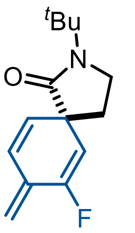

According to the general procedure **G**, using the corresponding substrate **3b** (0.2 mmol, 1.0 equiv), $Pd(PPh_3)_4$ (0.01 mmol, 5 mol%) , L_1 (0.01 mmol, 5 mol%), $NaHCO_3$ (50 mol%, 8.4 mg) and stirring at room temperature for 48 h under 30 W blue LEDs irradiation. The reaction mixture was filtered through a pad of silica gel and concentrated in vacuo, then purified by chromatography (petroleum ether/ethyl acetate = 12/1) to give **4b** (17.9 mg, 0.076 mmol, 38%) as white solid.

M.p. 102.7 - 103.8 ℃.
^{1}H NMR (400 MHz, CDCl$_3$) δ 6.35 (dd, J = 9.6, 7.4 Hz, 1H), 5.63 (d, J = 9.9 Hz, 1H), 5.36 (s, 1H), 5.32 (dd, J = 15.0, 2.0 Hz, 1H), 5.04 (s, 1H), 3.49 (td, J = 6.7, 1.4 Hz, 2H), 2.08 (q, J = 6.9 Hz, 2H), 1.42 (s, 9H).
^{13}C NMR (101 MHz, CDCl$_3$) δ 173.3 (d, J = 2.2 Hz), 157.2 (d, J = 254.7 Hz), 131.7 (d, J = 22.7 Hz), 128.8 (d, J = 1.7 Hz), 128.3 (d, J = 5.1 Hz), 110.3 (d, J = 3.2 Hz), 107.2 (d, J = 18.3 Hz), 54.6, 53.2 (d, J = 7.4 Hz), 42.1, 33.2 (d, J = 1.9 Hz), 27.6.
^{19}F NMR (376 MHz, CDCl$_3$) δ -123.9.
IR (KBr) 2967, 2926, 2834, 1685, 1597, 1362, 1245, 1076, 873, 779 cm^{-1}.
HRMS (ESI-MS) *m/z* calcd for $C_{14}H_{18}FNO$ [M+H]$^+$: 236.1445. found: 236.1442.

2-(Tert-butyl)-7-chloro-8-methylene-2-azaspiro[4.5]deca-6,9-dien-1-one (4c)

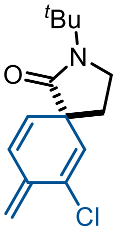

According to the general procedure **G**, using the corresponding substrate **3c** (0.2 mmol, 1.0 equiv), $Pd(PPh_3)_4$ (0.01 mmol, 5 mol%) , L_1 (0.01 mmol, 5 mol%), $NaHCO_3$ (50 mol%, 8.4 mg) and stirring at room temperature for 48 h under 30 W blue LEDs irradiation. The reaction mixture was filtered through a pad of silica gel and concentrated in vacuo, then purified by chromatography (petroleum ether/ethyl acetate = 12/1) to give **4c** (19.1 mg, 0.076 mmol, 38%) as colorless oil.

^{1}H NMR (400 MHz, CDCl$_3$) δ 6.41 (d, J = 9.7 Hz, 1H), 5.92 (t, J = 1.9 Hz, 1H), 5.65 (dt, J = 9.6, 1.9 Hz, 1H), 5.55 (s, 1H), 5.14 (s, 1H), 3.50 (t, J = 6.8 Hz, 2H), 2.11 – 2.03 (m, 2H), 1.42 (s, 9H).
^{13}C NMR (101 MHz, CDCl$_3$) δ 172.8, 135.2, 131.9, 128.7, 128.2, 128.2, 114.9, 54.7, 54.3, 42.1, 33.0, 27.6.
IR (KBr) 2970, 2832, 1597, 1362, 1256, 1099, 887, 775, 736 cm^{-1}.
HRMS (ESI-MS) *m/z* calcd for $C_{14}H_{18}ClNO$ [M+H]$^+$: 252.1150. found: 252.1147.

2-(Tert-butyl)-7-methoxy-8-methylene-2-azaspiro[4.5]deca-6,9-dien-1-one (4d)

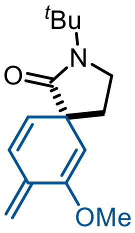

According to the general procedure **G**, using the corresponding substrate **3d** (0.2 mmol, 1.0 equiv), $Pd(PPh_3)_4$ (0.01 mmol, 5 mol%) , L_1 (0.01 mmol, 5 mol%), $NaHCO_3$ (50 mol%, 8.4 mg) and stirring at room temperature for 48 h under 30 W blue LEDs irradiation. The reaction mixture was filtered through a pad of silica gel and concentrated in vacuo, then purified by chromatography (petroleum ether/ethyl acetate = 12/1) to give **4d** (16.5 mg, 0.068 mmol, 34%) as colorless oil.

^{1}H NMR (400 MHz, CDCl$_3$) *δ* 6.37 (d, *J* = 9.6 Hz, 1H), 5.63 (dd, *J* = 9.5, 1.9 Hz, 1H), 5.46 (d, *J* = 1.8 Hz, 1H), 4.97 (s, 1H), 4.76 (s, 1H), 3.67 (s, 3H), 3.50 (t, *J* = 6.7 Hz, 2H), 2.12 – 1.99 (m, 2H), 1.43 (s, 9H).

^{13}C NMR (101 MHz, CDCl$_3$) *δ* 174.9, 152.7, 134.3, 129.1, 127.9, 110.5, 100.0, 54.4, 54.3, 52.7, 42.1, 34.1, 27.7.

IR (KBr) 2957, 2832, 2717, 2357, 1598, 1203, 1075, 774, 559 cm^{-1}.

HRMS (ESI-MS) *m/z* calcd for $C_{15}H_{21}NO_2$ $[M+H]^+$: 248.1645. found: 248.1641.

2-(Tert-butyl)-7-methyl-8-methylene-2-azaspiro[4.5]deca-6,9-dien-1-one (4e)

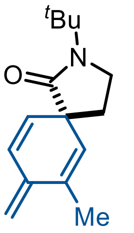

According to the general procedure **G**, using the corresponding substrate **3e** (0.2 mmol, 1.0 equiv), Pd(PPh$_3$)$_4$ (0.01 mmol, 5 mol%) , L$_1$ (0.01 mmol, 5 mol%), NaHCO$_3$ (50 mol%, 8.4 mg) and stirring at room temperature for 24 h under 30 W blue LEDs irradiation. The reaction mixture was filtered through a pad of silica gel and concentrated in vacuo, then purified by chromatography (petroleum ether/ethyl acetate = 12/1) to give **4e** (37.9 mg, 0.164 mmol, 82%) as colorless oil.

^{1}H NMR (400 MHz, CDCl$_3$) *δ* 6.39 (d, *J* = 9.6 Hz, 1H), 5.66 (dt, *J* = 9.5, 2.0 Hz, 1H), 5.54 (s, 1H), 5.09 (s, 1H), 4.96 (s, 1H), 3.48 (t, *J* = 6.8 Hz, 2H), 2.01 (t, *J* = 6.8 Hz, 2H), 1.93 (d, *J* = 1.3 Hz, 3H), 1.41 (s, 9H).

^{13}C NMR (101 MHz, CDCl$_3$) *δ* 174.45, 138.49, 132.74, 129.89, 128.67, 127.63, 111.52, 54.40, 52.31, 42.10, 33.26, 27.65, 19.28.

IR (KBr) 2962, 2832, 2719, 1598, 1362, 1208, 1078, 872, 775, 736 cm^{-1}.

HRMS (ESI-MS) *m/z* calcd for $C_{15}H_{21}NO$ $[M+H]^+$: 232.1696. found: 232.1694.

2-(Tert-butyl)-6-fluoro-8-methylene-2-azaspiro[4.5]deca-6,9-dien-1-one (4f)

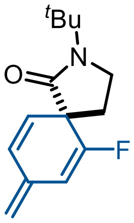

According to the general procedure **G**, using the corresponding substrate **3f** (0.2 mmol, 1.0 equiv), Pd(PPh$_3$)$_4$ (0.01 mmol, 5 mol%) , L$_1$ (0.01 mmol, 5 mol%), NaHCO$_3$ (50 mol%, 8.4 mg) and stirring at room temperature for 24 h under 30 W blue LEDs irradiation. The reaction mixture was filtered through a pad of silica gel and concentrated in vacuo, then purified by chromatography (petroleum ether/ethyl acetate = 12/1) to give **4f** (39.1 mg, 0.166 mmol, 83%) as colorless oil.

^{1}H NMR (500 MHz, CDCl$_3$) *δ* 6.29 (dt, *J* = 9.6, 1.8 Hz, 1H), 5.99 (dd, *J* = 15.1, 1.3 Hz, 1H), 5.61 (td, *J* = 9.4, 1.2 Hz, 1H), 4.97 (s, 2H), 3.56 – 3.42 (m, 2H), 2.41 (ddd, *J* = 13.4, 8.2, 5.5 Hz, 1H), 2.00 (ddd, *J* = 13.1, 8.2, 5.9 Hz, 1H), 1.41 (s, 9H).

^{13}C NMR (126 MHz, CDCl$_3$) *δ* 171.3 (d, *J* = 1.8 Hz), 161.3 (d, *J* = 262.6 Hz), 137.4 (d, *J* = 8.6 Hz), 128.8 (d, *J* = 6.0 Hz), 128.0 (d, *J* = 1.8 Hz), 114.4 (d, *J* = 9.2 Hz), 107.5 (d, *J* = 18.4 Hz), 54.9, 53.1 (d, *J* = 24.3 Hz), 42.7, 29.8, 27.5.

^{19}F NMR (471 MHz, CDCl$_3$) *δ* -116.7.

IR (KBr) 2959, 2832, 2718, 1598, 1362, 1199, 1076, 774 cm^{-1}.

HRMS (ESI-MS) *m/z* calcd for $C_{14}H_{18}FNO$ $[M+H]^+$: 236.1445. found: 236.1441.

2-(Tert-butyl)-6-chloro-8-methylene-2-azaspiro[4.5]deca-6,9-dien-1-one (4g)

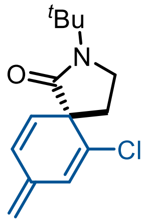

According to the general procedure **G**, using the corresponding substrate **3g** (0.2 mmol, 1.0 equiv), $Pd(PPh_3)_4$ (0.01 mmol, 5 mol%) , L_1 (0.01 mmol, 5 mol%), $NaHCO_3$ (50 mol%, 8.4 mg) and stirring at room temperature for 48 h under 30 W blue LEDs irradiation. The reaction mixture was filtered through a pad of silica gel and concentrated in vacuo, then purified by chromatography (petroleum ether/ethyl acetate = 12/1) to give **4g** (16.1 mg, 0.046 mmol, 23%) as colorless oil.

^{1}H NMR (400 MHz, CDCl$_3$) δ 6.51 (d, J = 1.4 Hz, 1H), 6.31 (dd, J = 9.7, 1.4 Hz, 1H), 5.69 (dt, J = 9.5, 1.0 Hz, 1H), 5.00 (s, 2H), 3.60 (ddd, J = 9.5, 8.8, 5.5 Hz, 1H), 3.51 (ddd, J = 9.6, 8.6, 5.4 Hz, 1H), 2.51 (ddd, J = 13.3, 8.8, 5.4 Hz, 1H), 2.00 (ddd, J = 13.8, 8.6, 5.5 Hz, 1H), 1.44 (s, 9H).
^{13}C NMR (101 MHz, CDCl$_3$) δ 171.7, 137.2, 135.9, 130.4, 128.3, 127.0, 115.0, 56.4, 54.9, 43.0, 30.9, 27.4.
IR (KBr) 2971, 2833, 1600, 1459, 1363, 1291, 1214, 1068, 885, 734 cm^{-1}.
HRMS (ESI-MS) *m/z* calcd for $C_{14}H_{18}ClNO$ $[M+H]^+$: 252.1150. found: 252.1145.

2-(Tert-butyl)-6-methyl-8-methylene-2-azaspiro[4.5]deca-6,9-dien-1-one (4h)

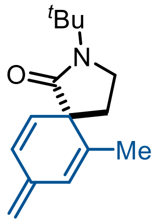

According to the general procedure **G**, using the corresponding substrate **3h** (0.2 mmol, 1.0 equiv), $Pd(PPh_3)_4$ (0.01 mmol, 5 mol%) , L_1 (0.01 mmol, 5 mol%), $NaHCO_3$ (50 mol%, 8.4 mg) and stirring at room temperature for 24 h under 30 W blue LEDs irradiation. The reaction mixture was filtered through a pad of silica gel and concentrated in vacuo, then purified by chromatography (petroleum ether/ethyl acetate = 12/1) to give **4h** (39.3 mg, 0.170 mmol, 85%) as colorless oil.

^{1}H NMR (400 MHz, CDCl$_3$) δ 6.31 (dd, J = 9.7, 1.6 Hz, 1H), 6.19 – 6.14 (m, 1H), 5.67 (d, J = 9.5 Hz, 1H), 4.85 (d, J = 4.9 Hz, 2H), 3.51 (dddd, J = 23.6, 9.7, 8.5, 5.6 Hz, 2H), 2.26 (ddd, J = 13.3, 8.7, 6.2 Hz, 1H), 1.93 (ddd, J = 13.4, 8.4, 5.1 Hz, 1H), 1.80 (s, 2H), 1.43 (s, 9H).
^{13}C NMR (101 MHz, CDCl$_3$) δ 174.0, 137.8, 136.8, 130.7, 127.6, 126.7, 112.1, 54.5, 54.3, 42.9, 30.8, 27.5, 19.3.
IR (KBr) 2960, 2832, 2718, 1598, 1362, 1203, 1075, 888, 773, 552 cm^{-1}.
HRMS (ESI-MS) *m/z* calcd for $C_{15}H_{21}NO$ $[M+H]^+$: 232.1696. found: 232.1692.

2-(Tert-butyl)-8-ethylidene-2-azaspiro[4.5]deca-6,9-dien-1-one (4i)

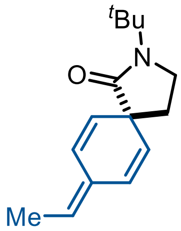

According to the general procedure **G**, using the corresponding substrate **3i** (0.2 mmol, 1.0 equiv), $Pd(PPh_3)_4$ (0.01 mmol, 5 mol%) , L_1 (0.01 mmol, 5 mol%), $NaHCO_3$ (50 mol%, 8.4 mg) and stirring at room temperature for 24 h under 30 W blue LEDs irradiation. The reaction mixture was filtered through a pad of silica gel and concentrated in vacuo, then purified by chromatography (petroleum ether/ethyl acetate = 12/1) to give **4i** (44.0 mg, 0.190 mmol, 95%) as white solid.

M.p. 70.0 - 72.1 ℃.

^{1}H NMR (500 MHz, CDCl$_3$) δ 6.65 (d, J = 10.0 Hz, 1H), 6.25 (d, J = 9.8 Hz, 1H), 5.66 (d, J = 10.0 Hz, 1H), 5.50 – 5.41 (m, 2H), 3.46 (td, J = 6.8, 2.0 Hz, 2H), 2.00 (t, J = 6.8 Hz, 2H), 1.79 (d, J = 7.4 Hz, 3H), 1.40 (s, 9H).
^{13}C NMR (126 MHz, CDCl$_3$) δ 174.5, 130.5, 130.2, 128.8, 126.0, 125.1, 123.5, 54.3, 51.9, 42.0, 33.8, 27.6, 12.9.
IR (KBr) 2966, 2864, 2838, 2719, 1601, 1454, 1364, 1279, 1249, 1095, 887, 782, 566 cm^{-1}.
HRMS (ESI-MS) *m/z* calcd for $C_{15}H_{21}NO$ [M+H]$^+$: 232.1696. found: 232.1692.

2-(Tert-butyl)-8-(propan-2-ylidene)-2-azaspiro[4.5]deca-6,9-dien-1-one (4j)

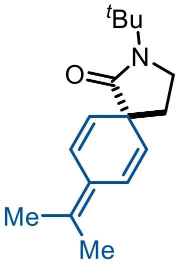

According to the general procedure **G**, using the corresponding substrate **3j** (0.2 mmol, 1.0 equiv), Pd(PPh$_3$)$_4$ (0.01 mmol, 5 mol%) , L$_1$ (0.01 mmol, 5 mol%), NaHCO$_3$ (50 mol%, 8.4 mg) and stirring at room temperature for 48 h under 30 W blue LEDs irradiation. The reaction mixture was filtered through a pad of silica gel and concentrated in vacuo, then purified by chromatography (petroleum ether/ethyl acetate = 12/1) to give **4j** (18.2 mg, 0.074 mmol, 37%) as white solid.

M.p. 99.8 - 101.3 ℃.
^{1}H NMR (500 MHz, CDCl$_3$) δ 6.70 (d, J = 10.2 Hz, 2H), 5.55 (d, J = 10.0 Hz, 2H), 3.48 (t, J = 6.8 Hz, 2H), 2.00 (t, J = 6.8 Hz, 2H), 1.87 (s, 6H), 1.42 (s, 9H).
^{13}C NMR (126 MHz, CDCl$_3$) δ 174.8, 131.8, 126.2, 125.4, 124.1, 54.3, 51.2, 42.0, 34.0, 27.6, 20.3.
IR (KBr) 2966, 2836, 2720, 1598, 1363, 1279, 1088, 932, 779, 733 cm^{-1}.
HRMS (ESI-MS) *m/z* calcd for $C_{16}H_{23}NO$ [M+H]$^+$: 246.1852. found: 246.1850.

2-(Tert-butyl)-8-cyclohexylidene-2-azaspiro[4.5]deca-6,9-dien-1-one (4k)

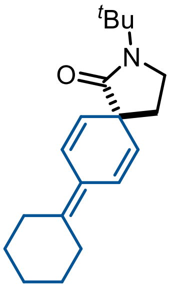

According to the general procedure **G**, using the corresponding substrate **3k** (0.2 mmol, 1.0 equiv), Pd(PPh$_3$)$_4$ (0.01 mmol, 5 mol%) , L$_1$ (0.01 mmol, 5 mol%), NaHCO$_3$ (50 mol%, 8.4 mg) and stirring at room temperature for 48 h under 30 W blue LEDs irradiation. The reaction mixture was filtered through a pad of silica gel and concentrated in vacuo, then purified by chromatography (petroleum ether/ethyl acetate = 12/1) to give **4k** (11.4 mg, 0.040 mmol, 20%) as white solid.

M.p. 106.7 - 108.1 ℃.
^{1}H NMR (500 MHz, CDCl$_3$) δ 6.75 (d, J = 10.2 Hz, 2H), 5.54 (d, J = 10.2 Hz, 2H), 3.45 (t, J = 6.8 Hz, 2H), 2.38 – 2.31 (m, 4H), 1.99 (t, J = 6.8 Hz, 2H), 1.57-1.56 (m, 6H), 1.40 (s, 9H).
^{13}C NMR (126 MHz, CDCl$_3$) δ 174.9, 140.5, 126.6, 124.9, 121.1, 54.3, 51.2, 42.0, 34.1, 30.1, 28.0, 27.6, 26.9.
IR (KBr) 2929, 2839, 1602, 1452, 1363, 1248, 1083, 887, 779, 733 cm^{-1}.
HRMS (ESI-MS) *m/z* calcd for $C_{19}H_{27}NO$ [M+H]$^+$: 286.2165. found: 286.2162.

Tert-butyl 2-(2-(tert-butyl)-1-oxo-2-azaspiro[4.5]deca-6,9-dien-8-ylidene)acetate (4l)

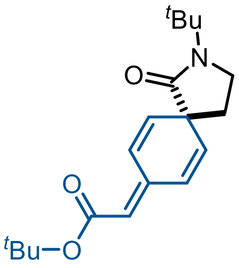

According to the general procedure **G**, using the corresponding substrate **3l** (0.2 mmol, 1.0 equiv), $Pd(PPh_3)_4$ (0.01 mmol, 5 mol%) , L_1 (0.01 mmol, 5 mol%), $NaHCO_3$ (50 mol%, 8.4 mg) and stirring at room temperature for 48 h under 30 W blue LEDs irradiation. The reaction mixture was filtered through a pad of silica gel and concentrated in vacuo, then purified by chromatography (petroleum ether/ethyl acetate = 12/1) to give **4l** (44.4 mg, 0.140 mmol, 70%) as white solid.

M.p. 150.3 - 151.6 ℃.

^{1}H NMR (500 MHz, CDCl$_3$) δ 7.77 (d, J = 10.2 Hz, 1H), 6.32 (d, J = 9.7 Hz, 1H), 6.04 (d, J = 10.1 Hz, 1H), 5.98 (d, J = 9.7 Hz, 1H), 5.49 (s, 1H), 3.53 (t, J = 6.9 Hz, 2H), 2.08 (t, J = 6.8 Hz, 2H), 1.48 (s, 9H), 1.40 (s, 9H).

^{13}C NMR (126 MHz, CDCl$_3$) δ 172.5, 166.3, 141.4, 135.4, 134.9, 129.9, 124.3, 117.1, 80.0, 54.7, 52.9, 42.2, 32.4, 28.3, 27.6.

IR (KBr) 3048, 2970, 2868, 2836, 2718, 1596, 1461, 1362, 1257, 1139, 979, 865, 770, 736, 567 cm^{-1}.

HRMS (ESI-MS) *m/z* calcd for $C_{19}H_{27}NO_3$ $[M+H]^+$: 318.2064. found: 318.2060.

2-(Tert-butyl)-4-methyl-8-methylene-2-azaspiro[4.5]deca-6,9-dien-1-one (4m)

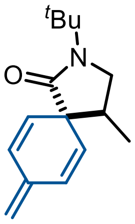

According to the general procedure **G**, using the corresponding substrate **3m** (0.2 mmol, 1.0 equiv), $Pd(PPh_3)_4$ (0.01 mmol, 5 mol%) , L_1 (0.01 mmol, 5 mol%), $NaHCO_3$ (50 mol%, 8.4 mg) and stirring at room temperature for 24 h under 30 W blue LEDs irradiation. The reaction mixture was filtered through a pad of silica gel and concentrated in vacuo, then purified by chromatography (petroleum ether/ethyl acetate = 12/1) to give **4m** (34.7 mg, 0.150 mmol, 75%) as white solid.

M.p. 80.5 - 81.2 ℃.

^{1}H NMR (400 MHz, CDCl$_3$) δ 6.45 (dd, J = 13.7, 10.8 Hz, 2H), 5.65 – 5.52 (m, 2H), 4.96 (s, 2H), 3.56 (dd, J = 9.7, 7.6 Hz, 1H), 3.11 (t, J = 9.7 Hz, 1H), 2.28 – 2.17 (m, 1H), 1.41 (s, 9H), 0.94 (d, J = 6.9 Hz, 3H).

^{13}C NMR (101 MHz, CDCl$_3$) δ 174.4, 137.1, 130.4, 130.3, 130.2, 125.1, 114.2, 55.9, 54.3, 49.4, 39.3, 27.7, 12.9.

IR (KBr) 3084, 3022, 2966, 2926, 2835, 2718, 1678, 1598, 1459, 1363, 1244, 1079, 984, 873, 780, 569 cm^{-1}.

HRMS (ESI-MS) *m/z* calcd for $C_{15}H_{21}NO$ $[M+H]^+$: 232.1696. found: 232.1693.

1'-(Tert-butyl)-4-methylene-4H-spiro[naphthalene-1,3'-pyrrolidin]-2'-one (4n)

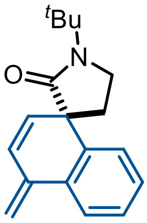

According to the general procedure **G**, using the corresponding substrate **3n** (0.2 mmol, 1.0 equiv), $Pd(PPh_3)_4$ (0.01 mmol, 5 mol%) , L_1 (0.01 mmol, 5 mol%), $NaHCO_3$ (50 mol%, 8.4 mg) and stirring at room temperature for 48 h under 30 W blue LEDs irradiation. The reaction mixture was filtered through a pad of silica gel and concentrated in vacuo, then purified by chromatography (petroleum ether/ethyl acetate = 12/1) to give **4n** (46.5 mg, 0.174 mmol, 87%) as white solid.

M.p. 110.5 - 112.1 ℃.

^{1}H NMR (400 MHz, $CDCl_3$) δ 7.80 (dd, J = 7.7, 1.6 Hz, 1H), 7.36 – 7.25 (m, 2H), 7.19 (dd, J = 7.5, 1.6 Hz, 1H), 6.56 (d, J = 9.8 Hz, 1H), 5.82 (dt, J = 9.8, 1.1 Hz, 1H), 5.75 (d, J = 1.5 Hz, 1H), 5.16 – 5.12 (m, 1H), 3.70 – 3.59 (m, 2H), 2.41 (ddd, J = 13.3, 8.3, 7.0 Hz, 1H), 2.26 (ddd, J = 13.0, 7.4, 5.3 Hz, 1H), 1.48 (s, 9H).

^{13}C NMR (101 MHz, $CDCl_3$) δ 175.4, 138.1, 137.0, 131.9, 129.4, 128.9, 128.6, 127.0, 126.7, 123.4, 110.6, 54.6, 53.1, 42.8, 35.9, 27.7.

IR (KBr) 2967, 2833, 2718, 1681, 1599, 1362, 1245, 1080, 880, 771, 548 cm^{-1}.

HRMS (ESI-MS) *m/z* calcd for $C_{18}H_{21}NO$ $[M+H]^+$: 268.1696. found: 268.1692.

7-(Tert-butyl)-1-methylene-2-oxa-7-azaspiro[4.4]non-3-en-6-one (4o)

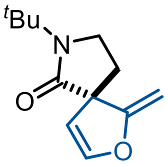

According to the general procedure **G**, using the corresponding substrate **3o** (0.2 mmol, 1.0 equiv), $Pd(PPh_3)_4$ (0.01 mmol, 5 mol%) , L_1 (0.02 mmol, 10 mol%), $NaHCO_3$ (50 mol%, 8.4 mg) and stirring at room temperature for 40 h under 30 W blue LEDs irradiation. The reaction mixture was filtered through a pad of silica gel and concentrated in vacuo, then purified by chromatography (petroleum ether/ethyl acetate = 12/1) to give **4o** (40.0 mg, 0.184 mmol, 92%) as white solid.

M.p. 68.0 - 69.2 ℃.

^{1}H NMR (500 MHz, $CDCl_3$) δ 6.55 (d, J = 2.9 Hz, 1H), 5.07 (d, J = 2.3 Hz, 1H), 4.60 (d, J = 1.5 Hz, 1H), 4.09 (d, J = 2.9 Hz, 1H), 3.46 (ddd, J = 9.9, 7.9, 6.4 Hz, 1H), 3.37 (ddd, J = 9.8, 8.3, 5.0 Hz, 1H), 2.12 (ddd, J = 12.9, 8.2, 6.3 Hz, 1H), 2.03 (ddd, J = 13.0, 7.9, 4.9 Hz, 1H), 1.35 (s, 9H).

^{13}C NMR (126 MHz, $CDCl_3$) δ 173.6, 166.1, 145.7, 106.9, 84.6, 58.5, 54.5, 42.3, 33.6, 27.6.

IR (KBr) 3107, 2959, 2835, 2717, 1602, 1362, 1138, 1053, 885, 820, 773, 730, 545 cm^{-1}.

HRMS (ESI-MS) *m/z* calcd for $C_{12}H_{17}NO_2$ $[M+H]^+$: 208.1332. found: 208.1331.

7-(Adamantan-1-yl)-1-methylene-2-oxa-7-azaspiro[4.4]non-3-en-6-one (4p)

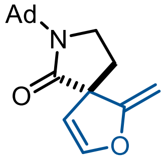

According to the general procedure **G**, using the corresponding substrate **3p** (0.2 mmol, 1.0 equiv), $Pd(PPh_3)_4$ (0.01 mmol, 5 mol%) , L_1 (0.02 mmol, 10 mol%), $NaHCO_3$ (50 mol%, 8.4 mg) and stirring at room temperature for 40 h under 30 W blue LEDs irradiation. The reaction mixture was filtered through a pad of silica gel and concentrated in vacuo, then purified by chromatography (petroleum ether/ethyl acetate = 12/1) to give **4p** (48.5 mg, 0.17 mmol, 85%) as white solid.

M.p. 192.9-194.1 ℃.

^{1}H NMR (400 MHz, $CDCl_3$) δ 6.62 (d, J = 2.9 Hz, 1H), 5.14 (d, J = 1.9 Hz, 1H), 4.68 (dd, J = 2.9, 1.3 Hz, 1H), 4.18 (d, J = 2.9 Hz, 1H), 3.54 (ddd, J = 9.9, 7.8, 6.4 Hz, 1H), 3.46 (ddd, J = 9.9, 8.1, 5.0 Hz, 1H), 2.19 – 2.06 (m, 11H), 1.70 (d, J = 3.3 Hz, 6H).

^{13}C NMR (101 MHz, $CDCl_3$) δ 173.6, 166.1, 145.7, 107.0, 84.5, 58.8, 55.6, 41.3, 39.5, 36.3, 33.6, 29.6.

IR (KBr) 2914, 2833, 2716, 1664, 1599, 1361, 1250, 1140, 1038, 884, 811, 774, 657 cm^{-1}.

HRMS (ESI-MS) *m/z* calcd for $C_{18}H_{23}NO_2$ $[M+H]^+$: 286.1802. found: 286.1797.

7-(Tert-butyl)-2-methylene-1-thia-7-azaspiro[4.4]non-3-en-6-one (4q)

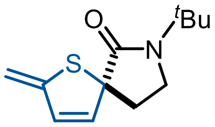

According to the general procedure **G**, using the corresponding substrate **3q** (0.2 mmol, 1.0 equiv), $Pd(PPh_3)_4$ (0.01 mmol, 5 mol%) , L_1 (0.01 mmol, 5 mol%), $NaHCO_3$ (50 mol%, 8.4 mg) and stirring at room temperature for 48 h under 30 W blue LEDs irradiation. The reaction mixture was filtered through a pad of silica gel and concentrated in vacuo, then purified by chromatography (petroleum ether/ethyl acetate = 12/1) to give **4q** (12.1 mg, 0.054 mmol, 27%) as colorless oil.

^{1}H NMR (500 MHz, CDCl$_3$) δ 6.37 (d, J = 6.0 Hz, 1H), 5.91 (d, J = 6.0 Hz, 1H), 5.17 (s, 1H), 5.02 (s, 1H), 3.47 (ddd, J = 10.1, 7.9, 3.3 Hz, 1H), 3.40 (ddd, J = 10.0, 7.8, 6.7 Hz, 1H), 2.43 (dt, J = 13.4, 7.8 Hz, 1H), 2.35 (ddd, J = 13.3, 6.7, 3.2 Hz, 1H), 1.41 (s, 9H).
^{13}C NMR (126 MHz, CDCl$_3$) δ 172.3, 149.7, 136.9, 134.4, 103.2, 70.4, 54.8, 42.8, 34.3, 27.5.
IR (KBr) 3050, 2968, 2833, 2717, 1599, 1402, 1362, 1287, 1075, 887, 774, 735 cm^{-1}.
HRMS (ESI-MS) *m/z* calcd for $C_{12}H_{17}NOS$ [M+H]$^+$: 224.1104. found: 224.1101.

1'-(Tert-butyl)-3-methylene-3a,7a-dihydro-3H-spiro[benzofuran-2,3'-pyrrolidin]-2'-one (4r)

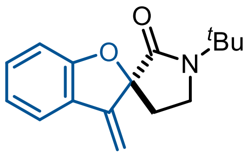

According to the general procedure **G**, using the corresponding substrate **3r** (0.2 mmol, 1.0 equiv), $Pd(PPh_3)_4$ (0.01 mmol, 5 mol%) , L_1 (0.01 mmol, 5 mol%), $NaHCO_3$ (50 mol%, 8.4 mg) and stirring at room temperature for 48 h under 30 W blue LEDs irradiation. The reaction mixture was filtered through a pad of silica gel and concentrated in vacuo, then purified by chromatography (petroleum ether/ethyl acetate = 12/1) to give **4r** (37.9 mg, 0.146 mmol, 73%) as colorless oil.

^{1}H NMR (500 MHz, CDCl$_3$) δ 7.37 (d, J = 7.6 Hz, 1H), 7.21 (t, J = 7.7 Hz, 1H), 6.90 (dd, J = 12.9, 7.8 Hz, 2H), 5.50 (s, 1H), 4.91 (s, 1H), 3.61 (td, J = 9.3, 4.1 Hz, 1H), 3.53 (dt, J = 9.7, 7.0 Hz, 1H), 2.48 (ddd, J = 14.5, 8.6, 6.3 Hz, 1H), 2.30 (ddd, J = 13.2, 7.7, 4.1 Hz, 1H), 1.46 (s, 9H).
^{13}C NMR (126 MHz, CDCl$_3$) δ 171.1, 161.7, 148.5, 130.9, 125.0, 121.1, 121.1, 110.6, 100.9, 91.4, 54.7, 41.6, 33.0, 27.5.
IR (KBr) 2961, 2926, 2838, 2718, 1697, 1600, 1464, 1363, 1254, 1211 10799, 866, 764, 580 cm^{-1}.
HRMS (ESI-MS) *m/z* calcd for $C_{16}H_{19}NO_2$ [M+H]$^+$: 258.1489. found: 258.1486.

7 Mechanistic studies

7.1 Radical trapping experiment

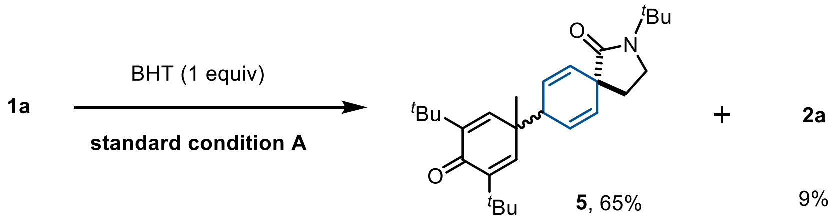

An oven-dried Schlenk tube containing a stir bar was charged with the redox ester **1a** (0.2 mmol, 1.0 equiv), BHT (1.0 equiv), $Pd(PPh_3)_4$ (5 mol%) and L_1 (10 mol%) in THF (0.1 M) under argon. The reaction was stirred at room temperate under blue LEDs irradiation (30 W, 460 nm) for 24 h. The corresponding reaction mixture was filtered through a pad of celite, washed with EtOAc and concentrated under reduced pressure. The residue was purified by flash chromatography on silica gel using petroleum ether/ethyl acetate (20:1, v:v) as eluent to afford the BHT adduct **5** (65%, 0.130mmol, 55 mg) as colorless oil with 9% carboamination product **2a**.

2-(Tert-butyl)-8-(3,5-di-tert-butyl-1-methyl-4-oxocyclohexa-2,5-dien-1-yl)-2-azaspiro[4.5]deca-6,9-dien-1-one (5)

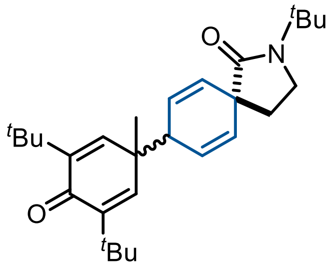

^{1}H NMR (400 MHz, $CDCl_3$) δ 6.62 (s, 2H), 5.78 (dd, J = 10.4, 3.3 Hz, 2H), 5.63 (dd, J = 10.4, 1.9 Hz, 2H), 3.43 (t, J = 6.7 Hz, 2H), 2.80 (tt, J = 3.5, 1.9 Hz, 1H), 1.92 (t, J = 6.7 Hz, 2H), 1.39 (s, 9H), 1.30 (s, 3H), 1.25 (s, 18H).
^{13}C NMR (101 MHz, $CDCl_3$) δ 186.8, 174.3, 146.8, 146.1, 129.2, 125.8, 54.1, 48.8, 44.8, 42.8, 41.8, 34.8, 34.3, 29.5, 27.6, 23.4.
IR (KBr) 2957, 2831, 2717, 1602, 1456, 1363, 1204, 1080, 773, 727, 566 cm^{-1}.
HRMS (ESI-MS) *m/z* calcd for $C_{28}H_{41}NO_2$ $[M+H]^+$: 424.3210. found: 424.3204.

7.2 Radical clock experiment

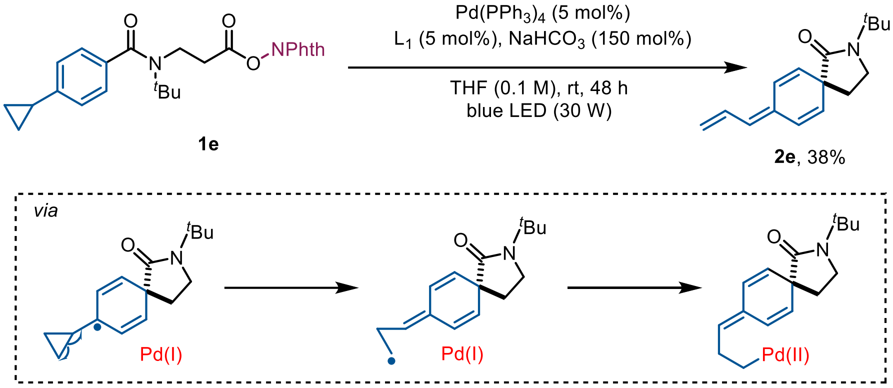

8-Allylidene-2-(tert-butyl)-2-azaspiro[4.5]deca-6,9-dien-1-one (2e)

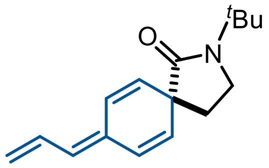

According to the general procedure **G**, using the corresponding substrate **1e** (0.2 mmol, 1.0 equiv), $Pd(PPh_3)_4$ (0.01 mmol, 5 mol%) , L_1 (0.01 mmol, 5 mol%), $NaHCO_3$ (50 mol%, 8.4 mg) and stirring at room temperature for 48 h under 30 W blue LEDs irradiation. The reaction mixture was filtered through a pad of silica gel and concentrated in vacuo, then purified by chromatography (petroleum ether/ethyl acetate = 12/1) to give **2e** (15.6 mg, 0.064 mmol, 32%) as colorless oil.

^{1}H NMR (400 MHz, CDCl$_3$) δ 6.88 – 6.75 (m, 2H), 6.31 (dd, J = 9.7, 1.7 Hz, 1H), 5.95 (d, J = 11.6 Hz, 1H), 5.76 (dt, J = 10.0, 1.9 Hz, 1H), 5.67 (dd, J = 9.7, 2.2 Hz, 1H), 5.28 (d, J = 16.7 Hz, 1H), 5.17 (d, J = 10.2 Hz, 1H), 3.49 (t, J = 6.8 Hz, 2H), 2.04 (t, J = 6.8 Hz, 2H), 1.42 (s, 9H).
^{13}C NMR (101 MHz, CDCl$_3$) δ 173.9, 131.8, 130.3, 130.3, 129.9, 129.0, 128.7, 123.5, 118.3, 54.5, 52.5, 42.1, 33.6, 27.6.
IR (KBr) 2967, 2832, 2718, 1598, 1362, 1256, 1207, 1078, 906, 775, 734, 560 cm^{-1}.
HRMS (ESI-MS) *m/z* calcd for $C_{16}H_{21}NO$ $[M+H]^+$: 244.1696. found: 244.1692.

7.3 Crossover experiment

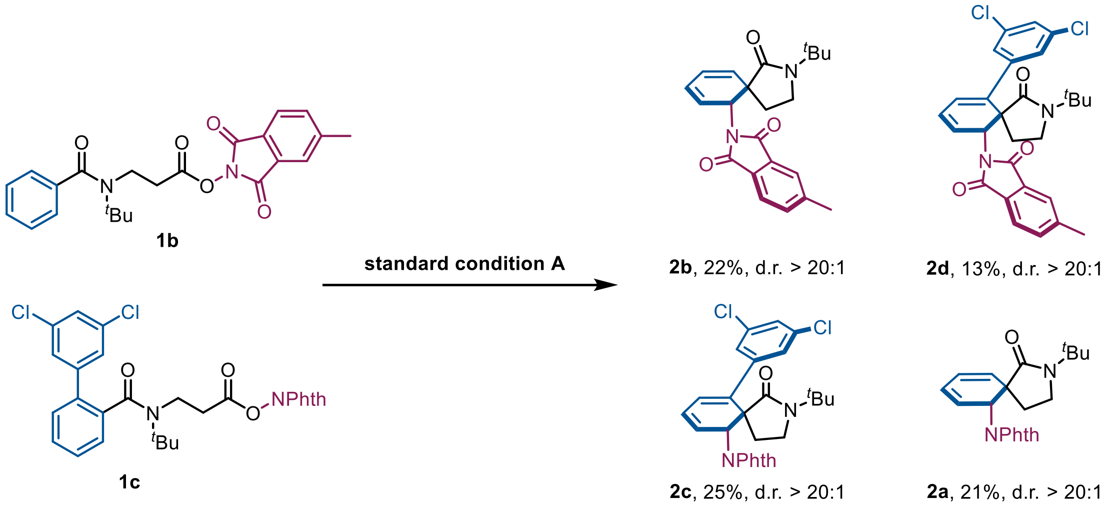

An oven-dried Schlenk tube containing a stir bar was charged with the redox esters **1b** (0.1 mmol) and **1c** (0.1 mmol), $Pd(PPh_3)_4$ (5 mol%) and L_1 (10 mol%) in THF (0.1 M) under argon. The reaction was stirred at room temperate under blue LEDs irradiation (30 W, 460 nm) for 48 h. The corresponding reaction mixture was filtered through a pad of celite, washed with EtOAc and concentrated under reduced pressure. The residue was purified by flash chromatography on silica gel using petroleum ether/ethyl acetate (6:1, v:v) as eluent to afford **2b** (22%, 0.044 mmol, 16.0 mg), **2d** (13%, 0.026mmol, 13.2 mg), **2c** (25%, 0.050 mmol, 24.8 mg) and **2a** (21%, 0.042 mmol, 14.7 mg) as colorless oil.

2-(2-(Tert-butyl)-10-(3,5-dichlorophenyl)-1-oxo-2-azaspiro[4.5]deca-7,9-dien-6-yl)-5-methylisoindoline-1,3-dione (2d)

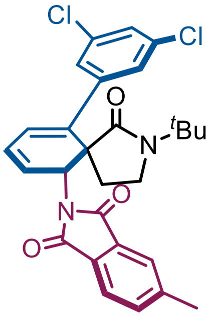

^{1}H NMR (400 MHz, CDCl$_3$) δ 7.76 (d, J = 7.6 Hz, 1H), 7.68 (s, 1H), 7.55 (d, J = 7.6 Hz, 1H), 7.28 – 7.24 (m, 3H), 6.25 (ddd, J = 9.4, 5.7, 2.7 Hz, 1H), 6.19 (d, J = 5.6 Hz, 1H), 5.85 – 5.79 (m, 1H), 5.66 (t, J = 3.1 Hz, 1H), 3.19 (td, J = 9.6, 3.1 Hz, 1H), 3.02 (dt, J = 10.0, 8.0 Hz, 1H), 2.83 (ddd, J = 14.3, 8.2, 3.1 Hz, 1H), 2.54 (s, 3H), 1.95 (ddd, J = 14.3, 9.4, 7.6 Hz, 1H), 1.23 (s, 9H).

^{13}C NMR (101 MHz, CDCl$_3$) δ 173.5, 145.7, 142.9, 139.4, 135.0, 134.5, 132.0, 129.1, 127.4, 126.2, 125.2, 125.0, 124.9, 124.0, 123.4, 55.5, 54.5, 52.3, 43.0, 27.3, 24.3, 22.1.

IR (KBr) 2809, 1774, 1714, 1681, 1584, 1556, 1457, 1406, 1381, 1346, 1283, 1249, 1217, 1153, 1119, 1095, 1054, 1015, 992, 931, 889, 852, 799, 738, 700, 625, 557, 503 cm^{-1}.

HRMS (ESI-MS) *m/z* calcd for $C_{28}H_{26}Cl_2N_2O_3$ $[M+H]^+$: 509.1393. found: 509.1389.

7.4 Control experiment

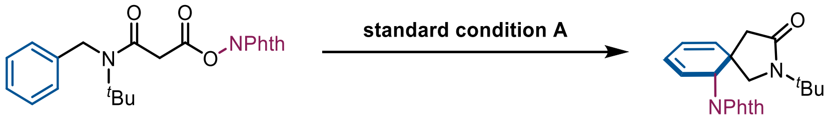

8

9, 38%, dr = 4:1

An oven-dried Schlenk tube containing a stir bar was charged with the redox esters **8** (0.4 mmol), $Pd(PPh_3)_4$ (5 mol%) and L_1 (10 mol%) in THF (0.1 M) under argon. The reaction was stirred at room temperate under blue LEDs irradiation (30 W, 460 nm) for 48 h. The corresponding reaction mixture was filtered through a pad of celite, washed with EtOAc and concentrated under reduced pressure. The residue was purified by flash chromatography on silica gel using petroleum ether/ethyl acetate (6:1, v:v) as eluent to afford **9** (38%, 0.152 mmol, 53.5 mg, d.r.= 4:1) as colorless oil.

2-(2-(Tert-butyl)-3-oxo-2-azaspiro[4.5]deca-7,9-dien-6-yl)isoindoline-1,3-dione (9)

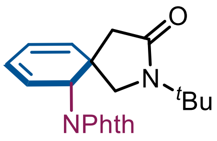

Major diastereoisomer:

^{1}H NMR (500 MHz, $CDCl_3$) δ 7.79 (dd, J = 5.5, 3.1 Hz, 2H), 7.66 (dd, J = 5.9, 3.0 Hz, 2H), 6.02 (ddd, J = 18.5, 9.5, 4.2 Hz, 2H), 5.76 (d, J = 9.4 Hz, 1H), 5.72 (dd, J = 9.5, 3.1 Hz, 1H), 5.16 (t, J = 3.1 Hz, 1H), 4.07 (d, J = 10.5 Hz, 1H), 2.98 (d, J = 10.5 Hz, 1H), 2.56 (d, J = 17.0 Hz, 1H), 2.38 (d, J = 16.9 Hz, 1H), 1.10 (s, 9H).

^{13}C NMR (126 MHz, $CDCl_3$) δ 173.3, 168.4, 134.8, 134.3, 125.3, 125.0, 124.3, 123.2, 122.9, 54.2, 53.9, 50.3, 45.3, 40.5, 27.3.

Minor diastereoisomer:

^{1}H NMR (500 MHz, $CDCl_3$) δ 7.76 (dd, J = 5.9, 2.9 Hz, 2H), 7.67 – 7.63 (m, 2H), 5.97 – 5.91 (m, 2H), 5.62 (dd, J = 9.9, 3.4 Hz, 2H), 5.31 – 5.24 (m, 1H), 3.50 (s, 2H), 2.34 (s, 2H), 1.37 (s, 9H).

^{13}C NMR (126 MHz, $CDCl_3$) δ 173.0, 167.7, 134.1, 134.0, 131.9, 131.7, 123.6, 55.9, 46.6, 44.1, 36.8, 29.7, 27.8.

IR (KBr) 2925, 2818, 2780, 1712, 1617, 1366, 1079, 765, 567 cm^{-1}.

HRMS (ESI-MS) *m/z* calcd for $C_{21}H_{22}N_2O_3$ $[M+H]^+$: 351.1703. found: 351.1701.

7.5 ESI-MS Analysis

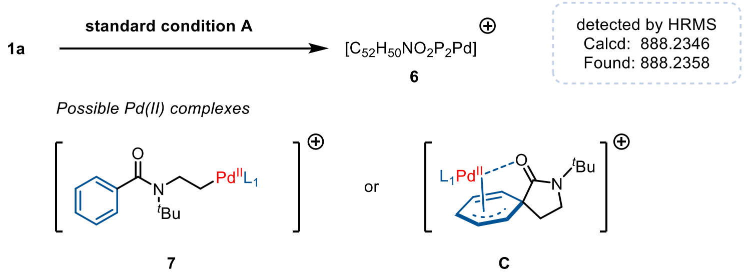

An oven-dried Schlenk tube containing a stir bar was charged with the redox ester **1a** (0.2 mmol, 1.0 equiv), $Pd(PPh_3)_4$ (5 mol%) and L_1 (10 mol%) in THF (0.1 M) under argon. The reaction was stirred at room temperate under blue LEDs irradiation (30 W) for 2 h. Then the possible palladium (II) complexes were detected and confirmed by high-resolution mass spectrometry (HRMS). HRMS (ESI-MS): calcd. for $C_{52}H_{50}NO_2P_2Pd$ (M+): 888.2346. Found: 888.2358.

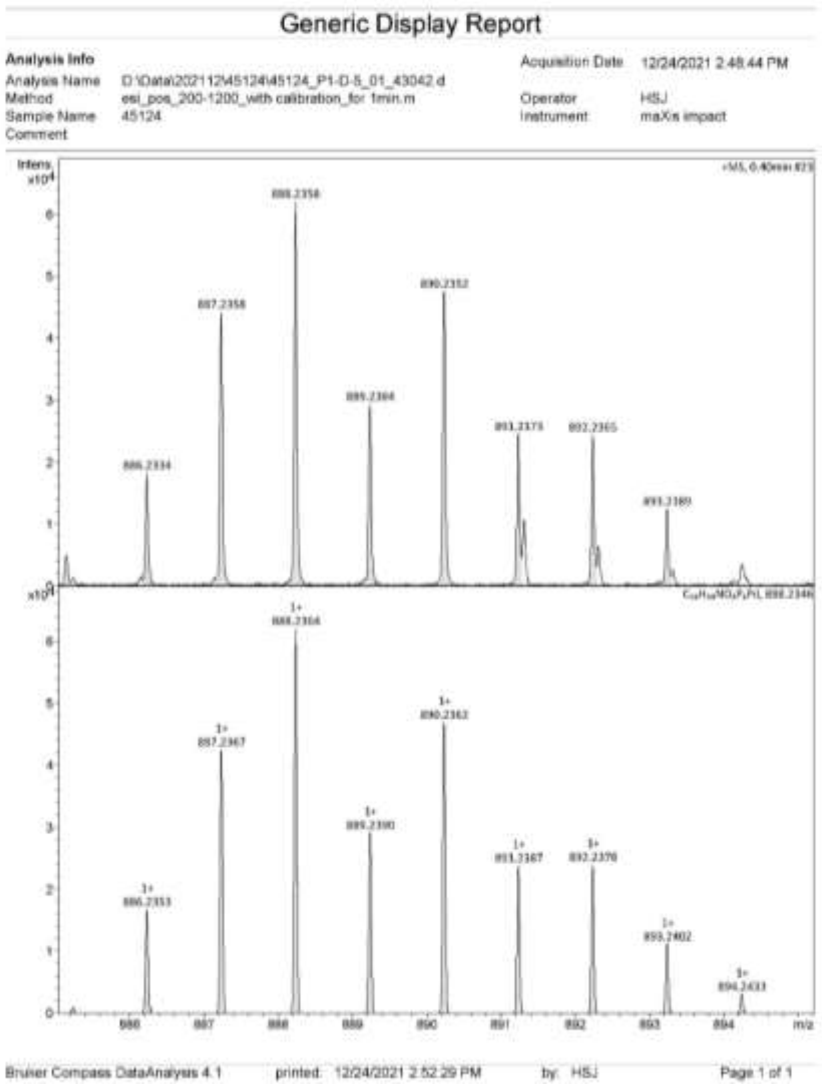

Figure S1: High-resolution mass spectra of possible palladium(II) complex

7.6 On-off light experiment

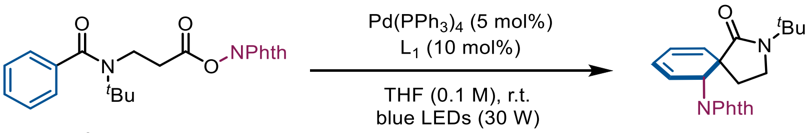

1a, 0.2 mmol

2a

Five oven-dried Schlenk tubes containing a stir bar were charged with the redox ester **1a** (0.2 mmol, 1.0 equiv), $Pd(PPh_3)_4$ (5 mol%) and L_1 (10 mol%) in THF (0.1 M) under argon. The reactions were stirred at room temperate under blue LEDs irradiation (30 W) The light was kept off during the off-periods and the yields of the reaction products were determined by ^1H NMR using CH_2Br_2 as an internal standard. No reaction was observed during the light off-cycles which confirms that the reaction is not proceeding by radical chain propagation mechanism.

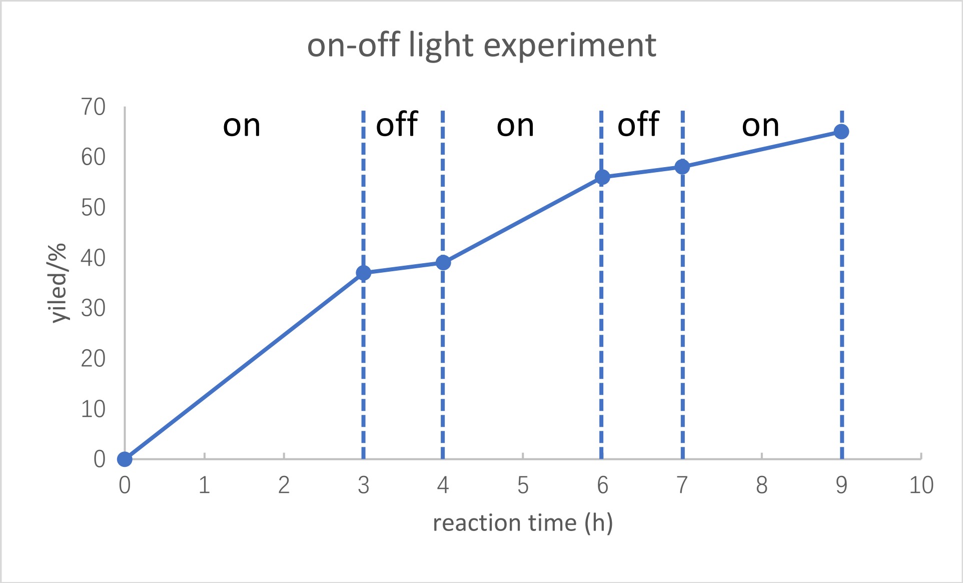

Figure S2 On-off light experiment

8 Gram-scale synthesis and derivatization of the products

8.1 Gram-scale synthesis

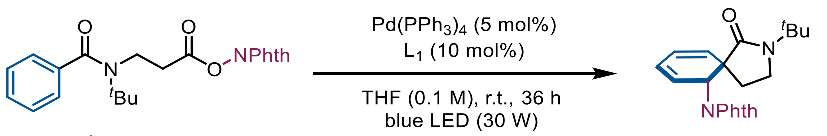

1a, 5 mmol scale

2a, 70%, dr > 20:1

In a 200 *mL* Schlenk tube with a magnetic bar under Ar atmosphere was added $Pd(PPh_3)_4$ (5 mol%, 289 mg), L_1 (10 mol%, 289 mg) and then the substrates **1a** (5 mmol) were added in THF (50 *mL*). The mixture was stirred at room temperature under Ar atmosphere and under blue LEDs (30 W×2) irradition for 36 h until the starting material was completely consumed (monitored by TLC). The corresponding reaction mixture was filtered through a pad of celite, washed with EtOAc and concentrated under reduced pressure. The residue was purified by flash chromatography on silica gel using petroleum ether/ethyl acetate (6:1, v:v) as eluent to afford the desired products **2a** (70%, 3.5 mmol, 1.23 g).

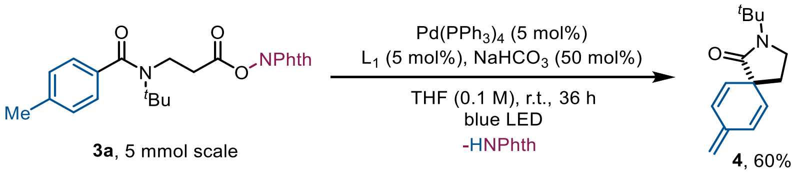

3a, 5 mmol scale

4, 60%

In a 200 *mL* Schlenk tube with a magnetic bar under Ar atmosphere was added $Pd(PPh_3)_4$ (5 mol%, 289 mg), L_1 (5 mol%, 289 mg), $NaHCO_3$ (50 mol%, 210 mg) and then the substrates **3a** (5 mmol) were added in THF (50 *mL*). The mixture was stirred at room temperature under Ar atmosphere and under blue LEDs (30 W×2) irradition for 36 h until the starting material was completely consumed (monitored by TLC). The corresponding reaction mixture was filtered through a pad of celite, washed with EtOAc and concentrated under reduced pressure. The residue was purified by flash chromatography on silica gel using petroleum ether/ethyl acetate (12:1, v:v) as eluent to afford the desired products **4a** (60%, 3.0 mmol, 0.65 g).

8.2 Derivatization of the products

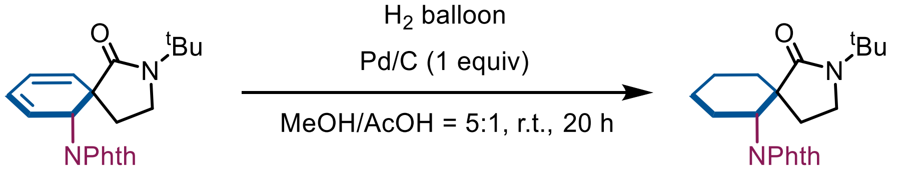

2a

10, 99%, d.r.> 20:1

In a round bottom flask equipped with a stir bar equipped with a rubber plug, 2-(2-(tert-butyl)-1-oxo-2-azaspiro[4.5]deca-7,9-dien-6-yl)isoindoline-1,3-dione **2a** (70.0 mg, 0.20 mmol, 1.0 equiv), Pd/C (21.0 mg, 0.20 mmol, 10 wt%) MeOH (5 *mL*) and AcOH (1 *mL*) were

sequentially added. The reaction mixture was degassed and a H_2 balloon was inserted on the flask, then the reaction mixture was stirred at room temperature for 20 h. The reaction mixture was filtered over a plug of silica, which was additionally rinsed with EtOAc. Then the reaction mixture was washed with saturated aqueous $NaHCO_3$ solution (10 *mL*). After that, the organic layer was dried over Na_2SO_4 and concentrated under reduced pressure, affording the product **10** as white solid (70.0 mg, 0.198 mmol, 99%).

2-(2-(Tert-butyl)-1-oxo-2-azaspiro[4.5]decan-6-yl)isoindoline-1,3-dione (10)

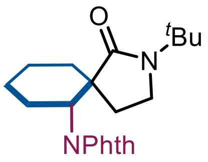

M.p. 143.3 - 145.1 ℃.

^{1}H NMR (400 MHz, CDCl$_3$) δ 7.79 (dd, J = 5.4, 3.1 Hz, 2H), 7.69 (dd, J = 5.5, 3.1 Hz, 2H), 4.49 (dd, J = 13.2, 3.6 Hz, 1H), 3.28 – 3.18 (m, 1H), 2.97 – 2.90 (m, 2H), 2.70 (qd, J = 13.1, 3.9 Hz, 1H), 1.98 – 1.80 (m, 2H), 1.74 – 1.63 (m, 2H), 1.53 – 1.20 (m, 4H), 1.08 (s, 9H).

^{13}C NMR (101 MHz, CDCl$_3$) δ 175.9, 168.5, 134.0, 131.6, 123.1, 54.9, 53.6, 52.1, 43.0, 36.6, 27.3, 26.4, 26.0, 25.7, 21.5.

IR (KBr) 3055, 2935, 2840, 2717, 1771, 1714, 1603, 1457, 1362, 1289, 1216, 1081, 1040, 921, 878, 804, 776, 726, 642, 567, 536, 482 cm^{-1}.

HRMS (ESI-MS) *m/z* calcd for $C_{21}H_{26}N_2O_3$ [M+H]$^+$: 355.2016. found: 355.2011.

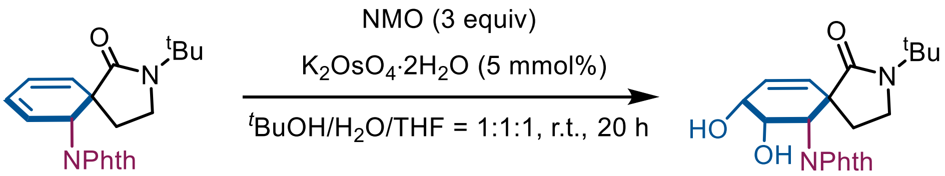

2a **11**, 65%, d.r.> 20:1

Following an adapted reported procedure[3], in a round bottom flask equipped with a stir bar and a glass stopper, 2-(2-(tert-butyl)-1-oxo-2-azaspiro[4.5]deca-7,9-dien-6-yl)isoindoline-1,3-dione **2a** (70.0 mg, 0.20 mmol, 1.0 equiv), ^{t}BuOH: H_2O: THF (9 *mL*, 1:1:1 mixture), $K_2OsO_4{\cdot}2H_2O$ (4 mg, 0.01 mmol, 5 mol%) and 4-methylmorpholine N-oxide (70.3 mg, 0.60 mmol, 3.0 equiv) were sequentially added. The resulting mixture was stirred at room temperature for 20 h. The reaction crude was quenched with saturated aqueous Na_2SO_3 (6 *mL*) and stirred for 1 h. The mixture was extracted with EtOAc (3×10 *mL*), the combined organic layers washed with KOH (10 *mL*, 1 M), brine (10 *mL*), dried over Na_2SO_4, filtered and the solvent removed in vacuo. The crude product was purified by column chromatography on silica gel (1:2 petroleum ether/EtOAc) to give **8** (50.0 mg, 0.13 mmol, 65%) as a white solid.

2-((5S,6R,7S,8R)-2-(Tert-butyl)-7,8-dihydroxy-1-oxo-2-azaspiro[4.5]dec-9-en-6-yl)isoindoline-1,3-dione (11)

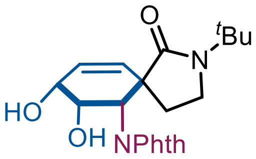

M.p. 216.9 - 217.5 ℃.

^{1}H NMR (400 MHz, CDCl$_3$) δ 7.88 (d, J = 7.0 Hz, 1H), 7.79 - 7.67 (m, 3H), 6.06 (dd, J = 9.9, 4.7 Hz, 1H), 5.62 (d, J = 9.9 Hz, 1H), 4.89 (d, J = 10.7 Hz, 1H), 4.67 - 4.60 (m, 1H), 4.37 – 4.28 (m, 2H), 3.67 (s, 1H), 3.34 (ddd, J = 10.0, 8.9, 3.7 Hz, 1H), 3.04 (dt, J = 10.0, 7.6 Hz, 1H), 2.69 (ddd, J = 13.6, 7.7, 3.6 Hz, 1H), 1.91 (ddd, J = 13.7, 8.9, 7.4 Hz, 1H), 1.23 (s, 9H).

^{13}C NMR (101 MHz, CDCl$_3$) δ 175.1, 169.4, 168.4, 134.2, 134.1, 132.7, 131.9, 131.4, 128.2, 123.5, 123.4, 66.4, 66.3, 54.6, 54.4, 53.4, 43.7, 27.4, 27.0.
IR (KBr) 3694, 2928, 2827, 1772, 1715, 1602, 1410, 1364, 1076, 895, 772, 725. 541 cm^{-1}.
HRMS (ESI-MS) *m/z* calcd for $C_{21}H_{24}N_2O_5$ [M+H]$^+$: 385.1758. found: 385.1757.

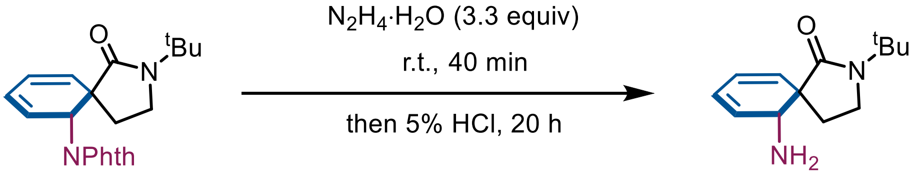

2a **12**, 90%, d.r.> 20:1

Following an adapted literature procedure[3], a Schlenk tube containing a stir bar was charged, under argon, with 2-(2-(tert-butyl)-1-oxo-2-azaspiro[4.5]deca-7,9-dien-6-yl)isoindoline-1,3-dione **2a** (70.0 mg, 0.20 mmol, 1.0 equiv), methanol (0.7 *mL*, 0.3 M) and hydrazine monohydrate (32 µL, 0.65 mmol, 3.3 equiv). The reaction mixture was stirred for 40 min at room temperature. Then, 5% HCl (0.9 *mL*) was added and the reaction mixture stirred for an additional 16 h. The resulting suspension was filtered through a short pad of silica gel (1 cm) and washed with CH_2Cl_2 (10 *mL*). The obtained solution was acidified to pH < 2, washed with DCM (2 × 5 *mL*) and the organic layer discarded. The aqueous layer was then basified using solid KOH until pH > 10, extracted with DCM (3 × 10 *mL*), the combined organic layers were washed with brine, dried over $MgSO_4$ and concentrated in vacuo. No further purification was needed and the desired product **12** was obtained as clear oil (40.0 mg, 0.18 mmol, 90%).

10-Amino-2-(tert-butyl)-2-azaspiro[4.5]deca-6,8-dien-1-one (12)

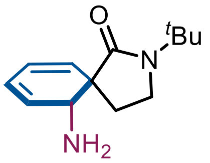

^{1}H NMR (400 MHz, CDCl$_3$) δ 5.94 (dd, J = 9.4, 4.9 Hz, 1H), 5.89 – 5.84 (m, 1H), 5.72 (d, J = 8.9 Hz, 1H), 5.62 (dd, J = 9.5, 2.6 Hz, 1H), 4.19 (s, 1H), 3.41 – 3.34 (m, 2H), 2.48 (dt, J = 12.7, 8.1 Hz, 1H), 1.64 – 1.61 (m, 1H), 1.42 (s, 9H).
^{13}C NMR (101 MHz, CDCl$_3$) δ 177.2, 132.7, 130.5, 124.2, 123.4, 54.3, 52.9, 51.4, 42.8, 27.7, 23.56.
IR (KBr) 3038, 2967, 2835, 2717, 1598, 1461, 1407, 1362, 1289, 1219, 1076, 818, 772, 737, 702, 535, 475 cm^{-1}.
HRMS (ESI-MS) *m/z* calcd for $C_{13}H_{20}N_2O$ [M+H]$^+$: 221.1648. found: 221.1646.

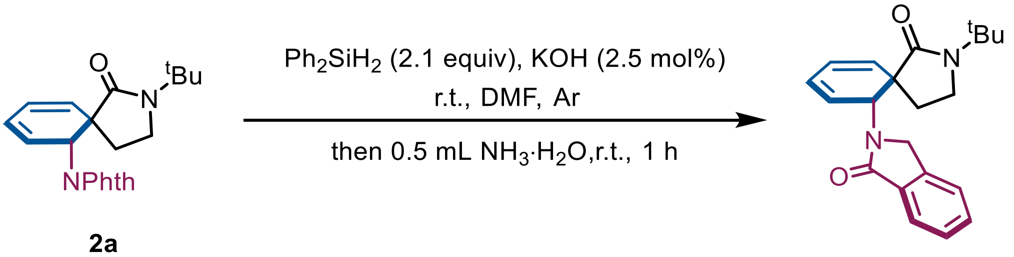

2a

13, 29%, d.r.> 20:1

A schlenk tube containing a stir bar was charged, under argon, with 2-(2-(tert-butyl)-1-oxo-2-azaspiro[4.5]deca-7,9-dien-6-yl)isoindoline-1,3-dione **2a** (70.0 mg, 0.20 mmol, 1.0 equiv),

KOH (2.5 mol%) and DMF (2 *mL*). Ph_2SiH_2 (2.1 equiv) was added slowly to the reaction mixture. After that, the reaction mixture stirred at room temperature until the starting material was consumed completely. Then, $NH_3{\cdot}H_2O$ (0.5 *mL*) was added and the reaction mixture stirred for an additional 1 h. Then water (15 *mL*) was added, the reaction mixture was extracted with DCM (3 × 10 *mL*). The organic layer was combined and dried over Na_2SO_4 and concentrated under reduced pressure. The crude mass was purified by silica gel column chromatography using petroleum ether/ethyl acetate (4:1) as eluent to afford **13** (29%, 0.058 mmol, 20.0 mg) as colorless oil.

2-(Tert-butyl)-10-(1-oxoisoindolin-2-yl)-2-azaspiro[4.5]deca-6,8-dien-1-one (13)

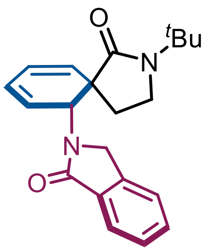

^{1}H NMR (400 MHz, $CDCl_3$) δ 7.87 (d, J = 7.5 Hz, 1H), 7.56 (td, J = 7.4, 1.2 Hz, 1H), 7.52 – 7.44 (m, 1H), 7.42 (d, J = 7.1 Hz, 1H), 6.33 (ddt, J = 9.5, 5.5, 1.0 Hz, 1H), 6.25 (ddd, J = 9.4, 5.5, 1.0 Hz, 1H), 5.75 (dd, J = 9.5, 5.7 Hz, 1H), 5.55 (dd, J = 9.4, 1.1 Hz, 1H), 5.15 (d, J = 5.6 Hz, 1H), 4.49 (d, J = 17.6 Hz, 1H), 4.23 (d, J = 17.6 Hz, 1H), 3.58 (dt, J = 9.8, 7.1 Hz, 1H), 3.37 (ddd, J = 9.8, 7.7, 4.4 Hz, 1H), 2.03 – 1.93 (m, 1H), 1.77 – 1.71 (m, 1H), 1.43 (s, 9H).

^{13}C NMR (101 MHz, $CDCl_3$) δ 173.3, 168.1, 141.5, 131.9, 131.7, 128.4, 128.1, 126.3, 125.8, 124.0, 122.8, 122.6, 54.42, 50.3, 47.3, 46.9, 42.0, 28.3, 27.6.

IR (KBr) 2963, 2831, 2717, 1602, 1460, 1363, 1291, 1210, 1082, 773, 729, 569 cm^{-1}.

HRMS (ESI-MS) *m/z* calcd for $C_{21}H_{24}N_2O_2$ $[M+H]^+$: 337.1911. found: 337.1906.

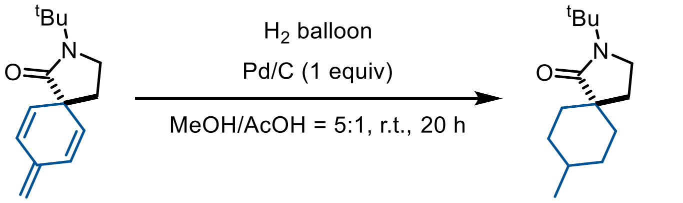

4a **14**, 99%, E/Z= 1:1

In a round bottom flask equipped with a stir bar equipped with a rubber plug, 2-(tert-butyl)-8-methylene-2-azaspiro[4.5]deca-6,9-dien-1-one **4a** (43.5 mg, 0.20 mmol, 1.0 equiv), Pd/C (21.0 mg, 0.20 mmol, 10 wt%) MeOH (5 *mL*) and AcOH (1 *mL*) were sequentially added. The reaction mixture was degassed and a H_2 balloon was inserted on the flask, then the reaction mixture was stirred at room temperature for 20 h. The reaction mixture was filtered over a plug of silica, which was additionally rinsed with EtOAc. Then the reaction mixture was washed with saturated aqueous $NaHCO_3$ solution (10 *mL*). After that, the organic layer was dried over Na_2SO_4 and concentrated under reduced pressure, affording the product **14** (44.2 mg, 0.198 mmol, 99%, *E/Z* = 1:1).

2-(Tert-butyl)-8-methyl-2-azaspiro[4.5]decan-1-one (14)

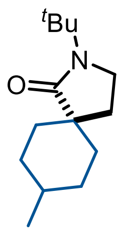

Cis-trans-isomer 1:

White solid, **m.p.** 60.4 - 61.2 ℃.

^{1}H NMR (400 MHz, CDCl$_3$) δ 3.31 (t, J = 6.8 Hz, 2H), 1.84 (ddd, J = 13.4, 7.4, 3.3 Hz, 2H), 1.78 – 1.70 (m, 3H), 1.63 – 1.57 (m, 2H), 1.55 – 1.47 (m, 2H), 1.38 (s, 9H), 1.24 – 1.17 (m, 2H), 0.98 (d, J = 5.9 Hz, 3H).

^{13}C NMR (101 MHz, CDCl$_3$) δ 179.9, 53.5, 44.2, 42.0, 32.6, 31.1, 29.9, 29.3, 27.7, 20.1.

IR (KBr) 2921, 2827, 1610, 1453, 1401, 1363, 1284, 1242, 1086, 574 cm^{-1}.

HRMS (ESI-MS) *m/z* calcd for $C_{14}H_{25}NO$ [M+H]$^+$: 224.2009. found: 224.2006.

Cis-trans-isomer 2:

Colorless oil.

^{1}H NMR (400 MHz, CDCl$_3$) δ 3.32 (t, J = 6.9 Hz, 2H), 1.82 – 1.77 (m, 3H), 1.69 – 1.59 (m, 4H), 1.41 – 1.37 (m, 11H), 1.03 – 0.92 (m, 2H), 0.89 (d, J = 6.5 Hz, 3H).

^{13}C NMR (101 MHz, CDCl$_3$) δ 180.0, 53.6, 45.8, 42.3, 32.1, 31.9, 31.0, 28.8, 27.7, 22.6.

IR (KBr) 2926, 2832, 2717, 1603, 1452, 1363, 1284, 1241, 1082, 773, 571 cm^{-1}.

HRMS (ESI-MS) *m/z* calcd for $C_{14}H_{25}NO$ [M+H]$^+$: 224.2009. found: 224.2005.

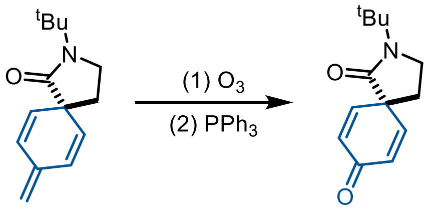

4a **15**, 36%

To a solution of **4a** (43.5 mg, 0.2 mmol) in the mixed solvent of DCM/MeOH (4 *mL*, v/v = 1:1) at 78 ℃ was purged with O_3 for 10 min until **4a** was all consumed (monitored by TLC). Then PPh_3 (2.0 mmol) was added and the mixture was stirred at room temperature for 1 h, the residue was concentrated under vacuum and purified with flash chromatography on silica gel, eluting with ethyl acetate/petroleum ether (v/v = 1:3) to afford **15** (15.8 mg, 0.072 mmol, 36%) as a clear oil.

2-(Tert-butyl)-2-azaspiro[4.5]deca-6,9-diene-1,8-dione (15)

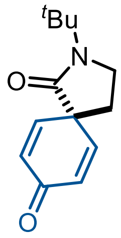

^{1}H NMR (500 MHz, CDCl$_3$) δ 6.80 (d, J = 10.0 Hz, 2H), 6.41 (d, J = 10.0 Hz, 2H), 3.65 (t, J = 6.7 Hz, 2H), 2.28 (t, J = 6.7 Hz, 2H), 1.44 (s, 9H).

^{13}C NMR (126 MHz, CDCl$_3$) δ 185.5, 169.6, 146.7, 130.9, 55.3, 53.8, 42.5, 30.4, 27.6.

HRMS (ESI-MS) *m/z* calcd for $C_{13}H_{17}NO_2$ [M+Na]$^+$: 242.1152. found: 242.1159.

9 X-Ray diffraction analysis

9.1 X-Ray diffraction analysis of 2l

Single crystals of $C_{22}H_{21}F_3N_2O_3$ were **2l**. A suitable crystal was selected and **2l** on a **Xcalibur, Eos, Gemini** diffractometer. The crystal was kept at 293(2) K during data collection. Using Olex2 [Dolomanov, O.V., Bourhis, L.J., Gildea, R.J, Howard, J.A.K. & Puschmann, H. (2009), J. Appl. Cryst. 42, 339-341.], the structure was solved with the SHELXT [Sheldrick, G.M. (2015). Acta Cryst. A71, 3-8.] structure solution program using Intrinsic Phasing and refined with the SHELXL [Sheldrick, G.M. (2015). Acta Cryst. C71, 3-8.] refinement package using Least Squares minimisation.

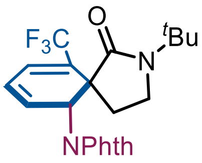

Table S3 Crystal data and structure refinement for 2l

Identification code	**2l**
Empirical formula	$C_{22}H_{21}F_3N_2O_3$
Formula weight	418.41
Temperature/K	293(2)
Crystal system	monoclinic
Space group	$P2_1/n$
a/Å	10.1611(7)
b/Å	12.2619(10)
c/Å	16.3549(11)
α/°	90
β/°	92.104(7)
γ/°	90
Volume/Å^3	2036.4(3)
Z	4
ρ_{calc}g/cm^3	1.365
μ/mm^{-1}	0.109
F(000)	872.0
Crystal size/mm^3	0.25 × 0.2 × 0.15
Radiation	Mo Kα (λ = 0.71073)
2Θ range for data collection/°	4.152 to 53.984
Index ranges	-12 ≤ h ≤ 12, -15 ≤ k ≤ 14, -20 ≤ l ≤ 13
Reflections collected	9550
Independent reflections	4384 [R_{int} = 0.0320, R_{sigma} = 0.0574]
Data/restraints/parameters	4384/0/274
Goodness-of-fit on F^2	1.042
Final R indexes [I>=2σ (I)]	R_1 = 0.0616, wR_2 = 0.1039
Final R indexes [all data]	R_1 = 0.1067, wR_2 = 0.1229
Largest diff. peak/hole / e Å^{-3}	0.16/-0.19

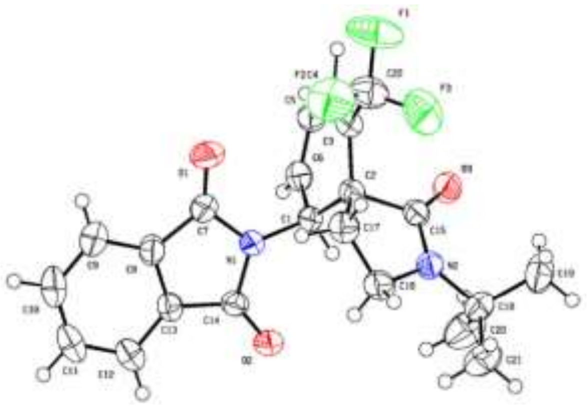

Figure S3: Crystal structure of compound 2l (CCDC 2285301)

9.2 X-Ray diffraction analysis of 2am’

Single crystals of $C_{19}H_{20}N_2O_4$ were **2am’**. A suitable crystal was selected and **2am’** on a **Xcalibur**, **Eos**, **Gemini** diffractometer. The crystal was kept at 293(2) K during data collection. Using Olex2 [Dolomanov, O.V., Bourhis, L.J., Gildea, R.J, Howard, J.A.K. & Puschmann, H. (2009), J. Appl. Cryst. 42, 339-341.], the structure was solved with the SHELXT [Sheldrick, G.M. (2015). Acta Cryst. A71, 3-8.] structure solution program using Intrinsic Phasing and refined with the SHELXL [Sheldrick, G.M. (2015). Acta Cryst. C71, 3-8.] refinement package using Least Squares minimisation.

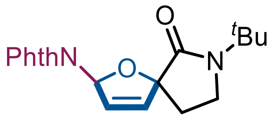

Table S4 Crystal data and structure refinement for 2am’

Identification code	**2am’**
Empirical formula	$C_{19}H_{20}N_2O_4$
Formula weight	340.37
Temperature/K	293(2)
Crystal system	monoclinic
Space group	$P2_1/c$
a/Å	8.3972(10)
b/Å	17.514(2)
c/Å	11.8059(15)
α/°	90
β/°	93.049(10)
γ/°	90
Volume/Å^3	1733.8(4)
Z	4
ρ_{calc}g/cm^3	1.304
μ/mm^{-1}	0.092
F(000)	720.0
Crystal size/mm^3	0.27 × 0.18 × 0.12
Radiation	Mo Kα (λ = 0.71073)
2Θ range for data collection/°	4.164 to 49.988
Index ranges	-9 ≤ h ≤ 9, -20 ≤ k ≤ 20, -14 ≤ l ≤ 13
Reflections collected	8044
Independent reflections	3035 [R_{int} = 0.0481, R_{sigma} = 0.0832]

Data/restraints/parameters	3035/0/229
Goodness-of-fit on F^2	1.032
Final R indexes [I>=2σ (I)]	R_1 = 0.0638, wR_2 = 0.1328
Final R indexes [all data]	R_1 = 0.1515, wR_2 = 0.1731
Largest diff. peak/hole / e $Å^{-3}$	0.21/-0.16

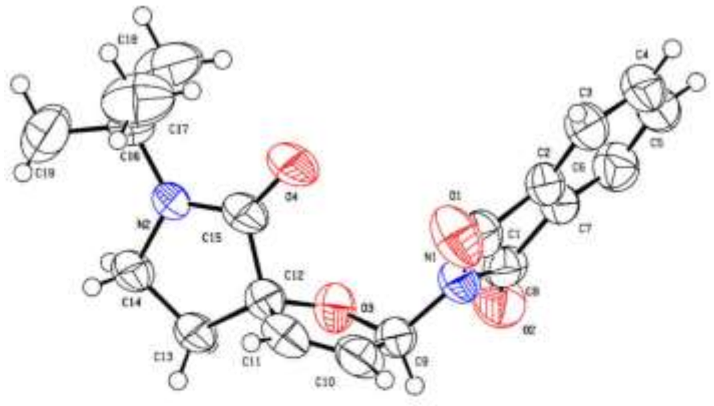

Figure S4: Crystal structure of compound 2am' (CCDC 2285304)

9.3 X-Ray diffraction analysis of 4k

Single crystals of $C_{19}H_{27}NO$ were **4k**. A suitable crystal was selected and **4k** on a **Xcalibur, Eos, Gemini** diffractometer. The crystal was kept at 293(2) K during data collection. Using Olex2 [Dolomanov, O.V., Bourhis, L.J., Gildea, R.J, Howard, J.A.K. & Puschmann, H. (2009), J. Appl. Cryst. 42, 339-341.], the structure was solved with the SHELXT [Sheldrick, G.M. (2015). Acta Cryst. A71, 3-8.] structure solution program using Intrinsic Phasing and refined with the SHELXL [Sheldrick, G.M. (2015). Acta Cryst. C71, 3-8.] refinement package using Least Squares minimisation.

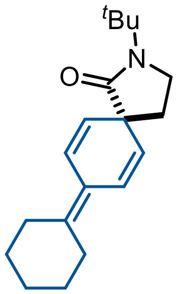

Table S5 Crystal data and structure refinement for 4k

Identification code	**4k**
Empirical formula	$C_{19}H_{27}NO$
Formula weight	285.41
Temperature/K	293(2)
Crystal system	monoclinic
Space group	$P2_1/n$
a/Å	6.0183(6)
b/Å	10.5530(13)
c/Å	26.951(4)
α/°	90
β/°	90.269(10)

γ/°	90
Volume/Å^3	1711.7(4)
Z	4
ρ_{calc}g/cm^3	1.108
μ/mm^{-1}	0.067
F(000)	624.0
Crystal size/mm^3	0.22 × 0.16 × 0.15
Radiation	Mo Kα (λ = 0.71073)
2Θ range for data collection/°	6.93 to 49.992
Index ranges	-6 ≤ h ≤ 7, -12 ≤ k ≤ 12, -32 ≤ l ≤ 24
Reflections collected	8609
Independent reflections	3009 [R_{int} = 0.0528, R_{sigma} = 0.0793]
Data/restraints/parameters	3009/1/193
Goodness-of-fit on F^2	1.034
Final R indexes [I>=2σ (I)]	R_1 = 0.0693, wR_2 = 0.1336
Final R indexes [all data]	R_1 = 0.1327, wR_2 = 0.1651
Largest diff. peak/hole / e Å^{-3}	0.20/-0.14

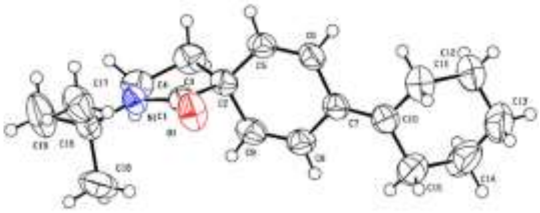

Figure S5: Crystal structure of compound 4k (CCDC 2285302)

9.4 X-Ray diffraction analysis of 11

Single crystals of $C_{22}H_{24}Cl_3DN_2O_5$ were **11**. A suitable crystal was selected and **11** on a **Xcalibur, Eos, Gemini** diffractometer. The crystal was kept at 293(2) K during data collection. Using Olex2 [Dolomanov, O.V., Bourhis, L.J., Gildea, R.J, Howard, J.A.K. & Puschmann, H. (2009), J. Appl. Cryst. 42, 339-341.], the structure was solved with the SHELXT [Sheldrick, G.M. (2015). Acta Cryst. A71, 3-8.] structure solution program using Intrinsic Phasing and refined with the SHELXL [Sheldrick, G.M. (2015). Acta Cryst. C71, 3-8.] refinement package using Least Squares minimisation.

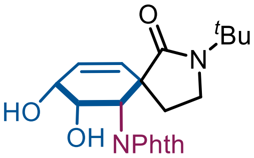

Table S6 Crystal data and structure refinement for 11

Identification code	**11**
Empirical formula	$C_{22}H_{24}Cl_3DN_2O_5$
Formula weight	504.79
Temperature/K	293(2)
Crystal system	monoclinic
Space group	P2$_1$/n
a/Å	15.0861(10)
b/Å	9.0898(5)
c/Å	17.9598(13)
α/°	90

β/°	92.866(7)
γ/°	90
Volume/Å^3	2459.7(3)
Z	4
ρ_{calc}g/cm^3	1.363
μ/mm^{-1}	0.407
F(000)	1048.0
Crystal size/mm^3	0.18 × 0.12 × 0.08
Radiation	synchrotron (λ = 0.71073)
2Θ range for data collection/°	4.542 to 49
Index ranges	-17 ≤ h ≤ 16, -7 ≤ k ≤ 10, -20 ≤ l ≤ 20
Reflections collected	11085
Independent reflections	4092 [R_{int} = 0.0346, R_{sigma} = 0.0473]
Data/restraints/parameters	4092/0/298
Goodness-of-fit on F^2	1.033
Final R indexes [I>=2σ (I)]	R_1 = 0.0761, wR_2 = 0.1868
Final R indexes [all data]	R_1 = 0.1076, wR_2 = 0.2132
Largest diff. peak/hole / e Å^{-3}	0.71/-0.57

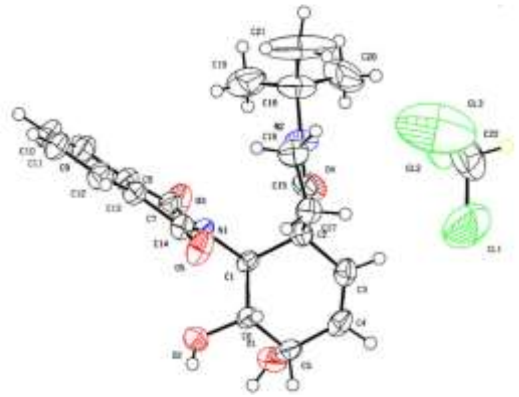

Figure S6: Crystal structure of compound 11 (CCDC 2285303)

10 Computational details

Quantum chemistry calculations were conducted with the Gaussian 09 software package[4]. The structures were optimized by the density functional theory (DFT)[5] with B3LYP-D3 functional[6,7] with basis set I (BSI, Lanl2dz[8] for Pd atom and 6-31G(d) for nonmetal atoms) using SMD[9] continuum solvent model (solvent =THF). Frequency analysises were perfomed at the same level of theory to verify the stationary points to be real minima or saddle points and to obtain the thermodyanamic energy corrections. All transition states were confirmed by intrinsic reaction coordinate (IRC) calculations were performed to confirm the connection between two correctminima for a transition state. In order to get more accurate electronic energies, the single point energy were calculated at the M06[10]-D3 /BSII level of theory (BSII, SDD[11] for Pd atom and 6-311+G(d,p)[11] for nonmetal atoms) using SMD continuum solvent model (solvent =THF).

Table S7. Calculated energy data and imaginary frequencies for all structure.

	Energy (au)	Thermal correction to Enthalpy（au）	Thermal correction to Gibbs Free Energy (au))	Imaginary frequency (cm^{-1})
	M06-D3 /BSII/SMD	B3LYP-D3 /BSI/SMD	B3LYP-D3 /BSI/SMD	B3LYP-D3 /BSI/SMD
**Substrate**	-1337.294970	0.435948	0.349527	None
**PC-S1**	-3426.433565			
**AR-1**	-1336.824010	0.433513	0.346998	None
**PC-cation**	-3426.365622	0.932631	0.785511	None
**INT-1**	-635.859314	0.299732	0.240607	None
**INT-Pd(I)-1**	-3938.835422	1.046338	0.87996	None
**CO2**	-188.560018	0.015074	-0.00927	None
**TS-1**	-635.848641	0.298882	0.242009	-428.78
**INT-2**	-635.886202	0.301498	0.245437	None
**INT-3**	-3538.800261	1.057845	0.901465	None
**INT-4**	-3026.346267	0.944207	0.804605	None
**NPhth-anion**	-512.426314	0.111575	0.071066	None
**TS-2a-1**	-3538.778533	1.05659	0.896842	-122.38
**TS-2a-2**	-3538.774297	1.058713	0.895317	-165.32
**TS-2a-3**	-3538.767445	1.057363	0.898422	-185.20
**TS-2a-4**	-3538.772131	1.057114	0.897434	-150.49
**INT-4-2ai-1**	-3065.648994	0.97249	0.832818	None
**INT-4-2ai-2**	-3065.642316	0.974085	0.832236	None

**INT-5**	-3538.812823	1.058799	0.898963	None
**TS-2ai-1**	-3578.081367	1.086445	0.924213	-124.61
**TS-2ai-2**	-3578.079035	1.086336	0.921489	-223.90
**TS-2ai-3**	-3578.077027	1.085696	0.921404	-102.71
**TS-2ai-4**	-3578.076148	1.086702	0.924684	-157.60
**TS-2ai-5**	-3578.069776	1.086226	0.923779	-213.14
**TS-2ai-6**	-3578.070833	1.086298	0.922536	-166.83
**TS-4a-2**	-3578.078142	1.085135	0.924288	-112.76
**TS-4a-1**	-3578.075079	1.081661	0.919884	-1673.09
**TS-4a-3**	-3578.073359	1.081066	0.918692	-1417.10
**TS-4a-4**	-3578.060437	1.081544	0.920174	-463.62
**TS-2f-1**	-3578.076716	1.086486	0.923475	-113.70
**TS-2f-2**	-3578.06609	1.081889	0.919776	-1689.35

10.1 Pathway and structure

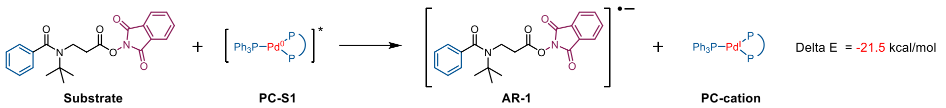

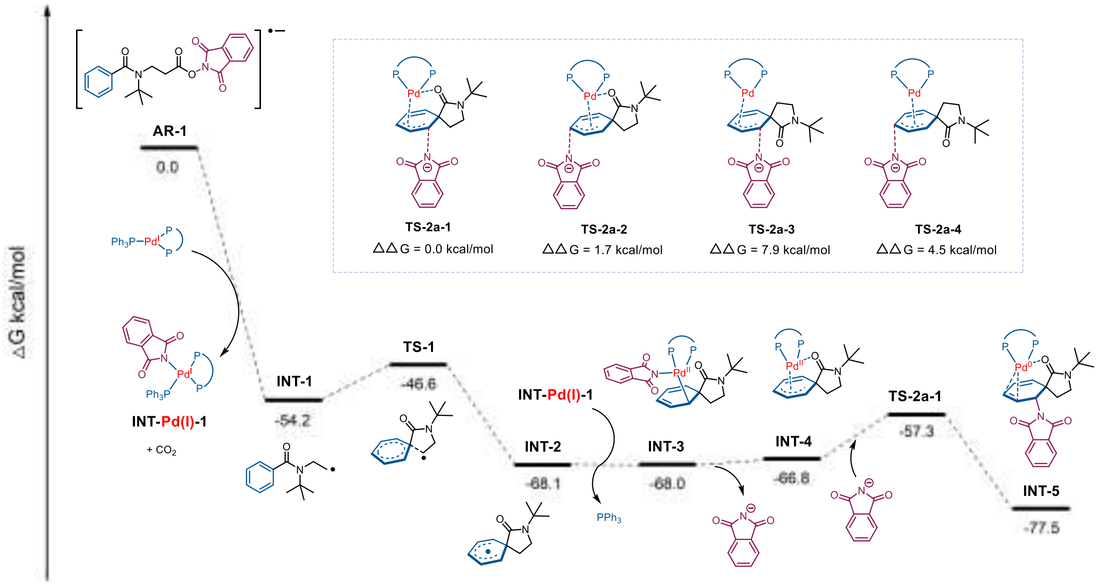

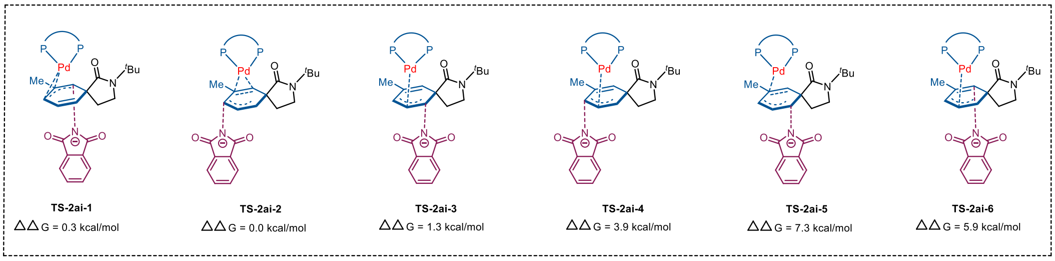

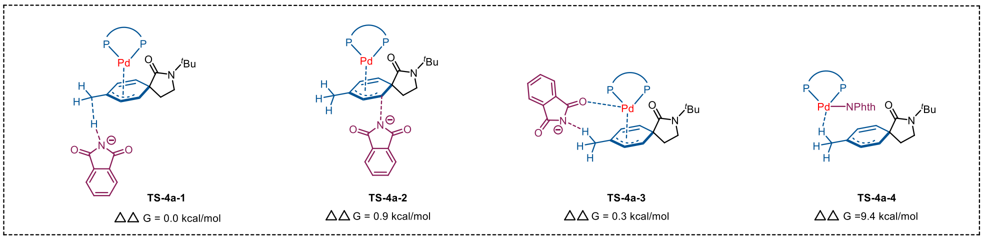

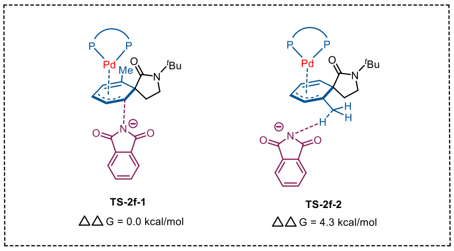

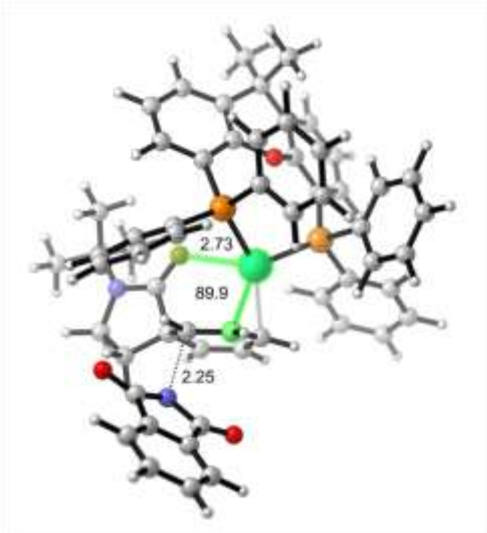

TS-2a-1

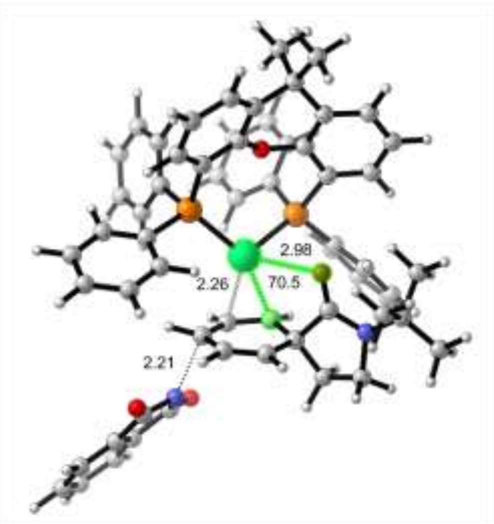

TS-2a-2

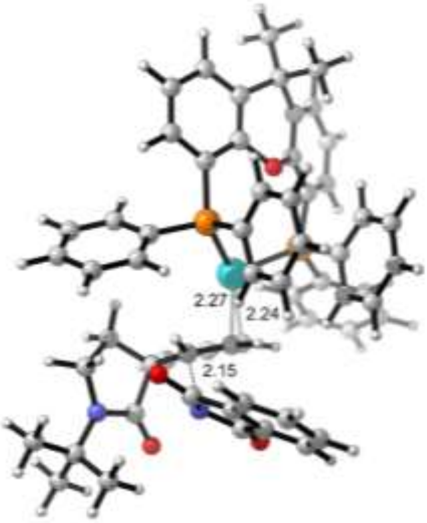

TS-2a-3

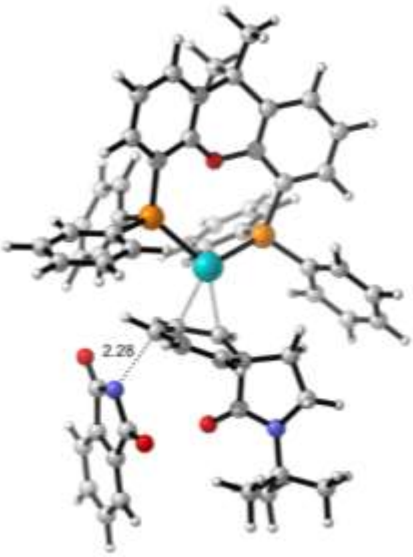

TS-2a-4

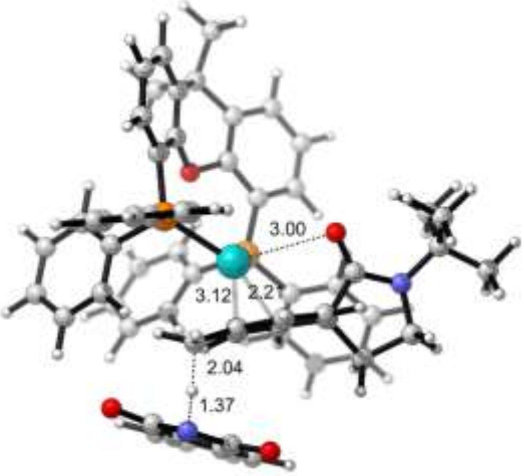

TS-4a-1

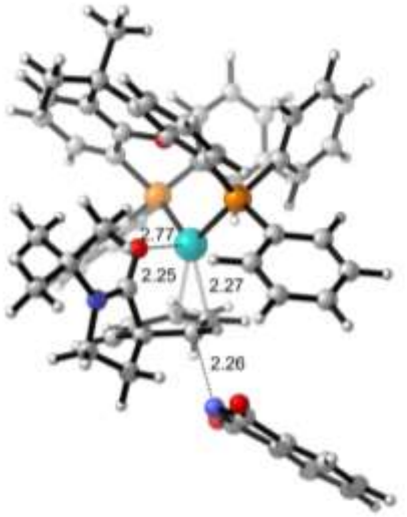

TS-4a-2

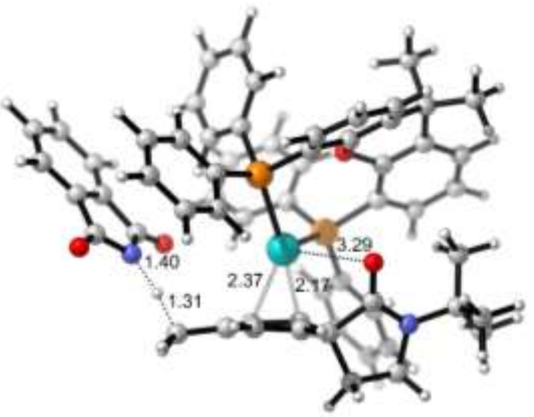

TS-4a-3

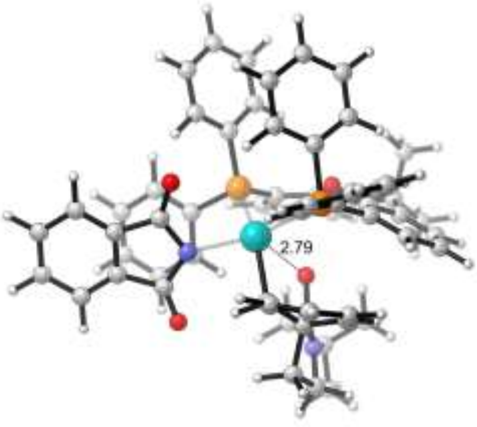

INT-3

Figure S7 Pathway and structure

10.2 Cartesian coordinates for all calculated structure

Substrate

Charge = 0   Multiplicity = 1

Center Number	Atomic Number	Atomic Type	Coordinates (Angstroms) X	Y	Z
1	6	0	2.683329	3.673544	-0.911824
2	6	0	2.655584	4.239565	0.365484
3	6	0	3.030585	3.475320	1.473975
4	6	0	3.417605	2.144436	1.308837
5	6	0	3.441406	1.573000	0.029752
6	6	0	3.087734	2.348309	-1.081138
7	1	0	2.396244	4.265033	-1.777163
8	1	0	2.346212	5.273077	0.496482
9	1	0	3.020036	3.914574	2.467960
10	1	0	3.701274	1.544934	2.170213
11	1	0	3.130418	1.910625	-2.074715
12	6	0	3.960839	0.172552	-0.199582
13	8	0	5.011973	0.049995	-0.833781
14	7	0	3.248763	-0.880161	0.325077
15	6	0	3.783514	-2.288550	0.166585
16	6	0	3.850732	-2.672925	-1.324662
17	6	0	5.177749	-2.370386	0.818682
18	6	0	2.878118	-3.299471	0.890772
19	1	0	2.852347	-2.662555	-1.776209
20	1	0	4.492900	-1.985925	-1.877456
21	1	0	4.254831	-3.686835	-1.427391
22	1	0	5.122143	-2.068409	1.871126
23	1	0	5.539101	-3.404242	0.779595
24	1	0	5.899245	-1.732229	0.308958
25	1	0	3.321082	-4.293846	0.773466
26	1	0	2.807665	-3.095215	1.964729

27	1	0	1.867827	-3.345773	0.471908
28	6	0	1.848508	-0.667755	0.719280
29	1	0	1.748105	0.305795	1.193833
30	1	0	1.565782	-1.398668	1.472839
31	6	0	0.887941	-0.738262	-0.475952
32	1	0	1.139998	0.032974	-1.214467
33	1	0	0.954194	-1.700996	-0.993481
34	6	0	-0.535514	-0.528320	-0.047057
35	8	0	-0.958439	-0.311297	1.056455
36	8	0	-1.367415	-0.620747	-1.169285
37	6	0	-3.507157	-1.438772	-0.334057
38	6	0	-3.223505	0.884827	-0.699484
39	6	0	-4.765479	-0.733545	0.033912
40	6	0	-4.597391	0.641182	-0.182344
41	6	0	-5.954608	-1.248443	0.526956
42	6	0	-5.613187	1.546126	0.084804
43	6	0	-6.986385	-0.339579	0.802412
44	1	0	-6.078285	-2.313504	0.695616
45	6	0	-6.818745	1.033544	0.585006
46	1	0	-5.476128	2.609557	-0.083931
47	1	0	-7.930997	-0.706324	1.192995
48	1	0	-7.635717	1.712696	0.809976
49	7	0	-2.697687	-0.414421	-0.871154
50	8	0	-2.635478	1.915463	-0.939716
51	8	0	-3.188871	-2.602297	-0.229233

PC-S1

Charge = 0 Multiplicity = 1

Center Number	Atomic Number	Atomic Type	Coordinates (Angstroms) X	Y	Z
1	6	0	-3.529571	0.893724	1.960764

2	6	0	-2.298349	1.110119	1.295772
3	6	0	-1.137654	1.178064	2.115522
4	6	0	-1.162777	1.051381	3.501788
5	6	0	-2.416604	0.837539	4.114628
6	6	0	-3.580334	0.754376	3.343150
7	6	0	0.111063	1.253859	4.330204
8	6	0	1.344773	1.047343	3.445937
9	6	0	1.249170	1.130565	2.055417
10	6	0	2.360747	0.957475	1.196080
11	6	0	3.615884	0.748124	1.791677
12	6	0	3.745411	0.703218	3.178700
13	6	0	2.624061	0.841136	3.993840
14	1	0	-4.443720	0.818901	1.382658
15	1	0	-2.485658	0.759920	5.193457
16	1	0	-4.539478	0.597998	3.831295
17	1	0	4.492820	0.609094	1.169063
18	1	0	4.724388	0.550963	3.625417
19	1	0	2.745945	0.803359	5.070761
20	8	0	0.050747	1.415770	1.444069
21	6	0	0.139872	0.267312	5.520289
22	1	0	0.131589	-0.768798	5.167243
23	1	0	1.032889	0.415459	6.133986
24	1	0	-0.720028	0.418679	6.178441
25	6	0	0.124100	2.705992	4.877979
26	1	0	1.025735	2.882780	5.476604
27	1	0	0.109453	3.432867	4.057825
28	1	0	-0.753282	2.885349	5.510330
29	15	0	-2.117533	0.971024	-0.532719
30	15	0	2.083129	0.852487	-0.621264
31	6	0	-3.759267	0.522766	-1.193202
32	6	0	-3.926039	-0.727988	-1.811104
33	6	0	-4.863701	1.397248	-1.118481
34	6	0	-5.166374	-1.110021	-2.320548
35	1	0	-3.078327	-1.401936	-1.887737

36	6	0	-6.103253	1.014949	-1.637321
37	1	0	-4.749869	2.375496	-0.660099
38	6	0	-6.260319	-0.236393	-2.238492
39	1	0	-5.278422	-2.086336	-2.784307
40	1	0	-6.945927	1.699325	-1.574748
41	1	0	-7.225199	-0.530048	-2.643678
42	6	0	-1.908643	2.722131	-1.101591
43	6	0	-2.207153	3.035154	-2.439539
44	6	0	-1.410543	3.730548	-0.264551
45	6	0	-2.042708	4.335862	-2.917844
46	1	0	-2.585586	2.261669	-3.103552
47	6	0	-1.228050	5.027597	-0.750659
48	1	0	-1.163282	3.510266	0.767834
49	6	0	-1.552317	5.336545	-2.073734
50	1	0	-2.297214	4.567327	-3.949257
51	1	0	-0.828621	5.795398	-0.093354
52	1	0	-1.414396	6.347764	-2.447772
53	6	0	3.664460	0.226275	-1.320472
54	6	0	4.834427	1.003028	-1.388869
55	6	0	3.684631	-1.081133	-1.831557
56	6	0	6.003519	0.468251	-1.934526
57	1	0	4.830162	2.024903	-1.019849
58	6	0	4.855247	-1.616641	-2.371313
59	1	0	2.777364	-1.675800	-1.817986
60	6	0	6.018552	-0.842430	-2.421656
61	1	0	6.902891	1.076902	-1.982027
62	1	0	4.851059	-2.631939	-2.759214
63	1	0	6.930078	-1.254189	-2.846892
64	6	0	2.068302	2.616675	-1.164452
65	6	0	1.629587	2.899875	-2.468125
66	6	0	2.508713	3.667140	-0.346974
67	6	0	1.660168	4.206077	-2.954977
68	1	0	1.257640	2.093390	-3.097184
69	6	0	2.531038	4.976534	-0.834907

70	1	0	2.838786	3.463388	0.667457
71	6	0	2.115255	5.247163	-2.140279
72	1	0	1.316457	4.413644	-3.964607
73	1	0	2.874529	5.784084	-0.193290
74	1	0	2.132029	6.266267	-2.517563
75	46	0	-0.063895	-0.076904	-1.118805
76	15	0	-0.102750	-2.285153	-0.256944
77	6	0	0.755763	-2.588438	1.343883
78	6	0	2.146585	-2.379196	1.392894
79	6	0	0.090590	-2.971939	2.520184
80	6	0	2.852941	-2.562620	2.578299
81	1	0	2.684229	-2.083851	0.498310
82	6	0	0.800695	-3.135578	3.712998
83	1	0	-0.976212	-3.160860	2.515045
84	6	0	2.180646	-2.931617	3.748840
85	1	0	3.926575	-2.401925	2.591464
86	1	0	0.268254	-3.433040	4.612645
87	1	0	2.729622	-3.059932	4.677691
88	6	0	-1.793224	-2.963172	-0.041180
89	6	0	-2.276957	-4.020678	-0.826039
90	6	0	-2.663944	-2.328145	0.864932
91	6	0	-3.608199	-4.431438	-0.710836
92	1	0	-1.622895	-4.520909	-1.533125
93	6	0	-3.989081	-2.746886	0.981248
94	1	0	-2.312601	-1.503168	1.473108
95	6	0	-4.467370	-3.793510	0.188722
96	1	0	-3.972158	-5.248671	-1.327948
97	1	0	-4.648631	-2.237811	1.678633
98	1	0	-5.504184	-4.108760	0.269265
99	6	0	0.735098	-3.441429	-1.420519
100	6	0	1.373900	-4.614042	-0.987000
101	6	0	0.732497	-3.128483	-2.789754
102	6	0	2.009795	-5.449251	-1.907434
103	1	0	1.384528	-4.871341	0.067637

104	6	0	1.365745	-3.967232	-3.708302
105	1	0	0.249297	-2.213049	-3.127379
106	6	0	2.009282	-5.127198	-3.267918
107	1	0	2.507323	-6.351056	-1.560475
108	1	0	1.363622	-3.709553	-4.764053
109	1	0	2.508423	-5.777692	-3.981262

AR-1

Charge = -1 Multiplicity = 2

Center Number	Atomic Number	Atomic Type	Coordinates (Angstroms) X	Y	Z
1	6	0	2.744711	3.659728	-0.936038
2	6	0	2.738756	4.232817	0.338341
3	6	0	3.113254	3.468539	1.446961
4	6	0	3.477960	2.131093	1.284751
5	6	0	3.479842	1.552580	0.008635
6	6	0	3.126981	2.327613	-1.102375
7	1	0	2.454990	4.250238	-1.801141
8	1	0	2.443968	5.270864	0.467312
9	1	0	3.117020	3.912270	2.439082
10	1	0	3.758138	1.530677	2.146667
11	1	0	3.149269	1.883074	-2.093584
12	6	0	3.973930	0.142161	-0.216271
13	8	0	5.022484	0.000238	-0.852917
14	7	0	3.246575	-0.893447	0.316468
15	6	0	3.758998	-2.310133	0.170150
16	6	0	3.817000	-2.710107	-1.317560
17	6	0	5.153944	-2.407853	0.819277
18	6	0	2.840715	-3.300783	0.906484
19	1	0	2.818288	-2.686385	-1.767377
20	1	0	4.469290	-2.038585	-1.877606

21	1	0	4.203879	-3.731701	-1.411782
22	1	0	5.105948	-2.095223	1.869073
23	1	0	5.499532	-3.447546	0.789190
24	1	0	5.883432	-1.785182	0.301683
25	1	0	3.267629	-4.303113	0.796189
26	1	0	2.777495	-3.086248	1.978839
27	1	0	1.828240	-3.334367	0.492717
28	6	0	1.849015	-0.656391	0.715881
29	1	0	1.767266	0.325060	1.177510
30	1	0	1.563798	-1.370266	1.484509
31	6	0	0.873998	-0.730512	-0.464758
32	1	0	1.124960	0.028520	-1.215429
33	1	0	0.926644	-1.700116	-0.971323
34	6	0	-0.548761	-0.509151	-0.012321
35	8	0	-0.923151	-0.331109	1.119696
36	8	0	-1.379363	-0.545176	-1.108199
37	6	0	-3.542833	-1.424827	-0.362949
38	6	0	-3.254877	0.955609	-0.627871
39	6	0	-4.766540	-0.745509	-0.007919
40	6	0	-4.594254	0.677700	-0.165656
41	6	0	-5.992890	-1.258355	0.450874
42	6	0	-5.652735	1.552224	0.137861
43	6	0	-7.025483	-0.380493	0.745743
44	1	0	-6.120640	-2.332045	0.571988
45	6	0	-6.855186	1.028161	0.588771
46	1	0	-5.519787	2.625833	0.020766
47	1	0	-7.978285	-0.764663	1.102930
48	1	0	-7.680959	1.694090	0.828717
49	7	0	-2.718998	-0.351188	-0.803622
50	8	0	-2.632549	2.005028	-0.856481
51	8	0	-3.193224	-2.616156	-0.341966

PC-cation

Charge = 1 Multiplicity = 2

Center Number	Atomic Number	Atomic Type	Coordinates (Angstroms) X	Y	Z
1	6	0	3.338060	-1.213221	1.962930
2	6	0	2.116476	-1.297181	1.279978
3	6	0	0.935162	-1.369420	2.049936
4	6	0	0.941082	-1.359083	3.449412
5	6	0	2.185073	-1.261432	4.085225
6	6	0	3.370867	-1.190051	3.355436
7	6	0	-0.367524	-1.563411	4.223423
8	6	0	-1.553594	-1.123209	3.357450
9	6	0	-1.435055	-1.102456	1.964843
10	6	0	-2.496565	-0.731228	1.113287
11	6	0	-3.733439	-0.436279	1.700744
12	6	0	-3.889864	-0.486478	3.084407
13	6	0	-2.807227	-0.811121	3.899215
14	1	0	4.264566	-1.140293	1.405272
15	1	0	2.236944	-1.256707	5.167993
16	1	0	4.322803	-1.120292	3.873354
17	1	0	-4.573982	-0.152115	1.077205
18	1	0	-4.854143	-0.260063	3.529798
19	1	0	-2.949029	-0.834298	4.973493
20	8	0	-0.253122	-1.477868	1.357437
21	6	0	-0.346014	-0.788742	5.556955
22	1	0	-0.243107	0.287627	5.384215
23	1	0	-1.262682	-0.962901	6.126634
24	1	0	0.477732	-1.121609	6.193909
25	6	0	-0.517423	-3.081167	4.512881
26	1	0	-1.445659	-3.272225	5.063594
27	1	0	-0.547305	-3.658637	3.581976
28	1	0	0.326906	-3.439683	5.112855

29	15	0	2.016736	-1.161068	-0.554851
30	15	0	-2.208262	-0.572409	-0.699455
31	6	0	3.706639	-0.823159	-1.171448
32	6	0	3.945612	0.394308	-1.824386
33	6	0	4.744138	-1.766655	-1.065629
34	6	0	5.210267	0.677423	-2.345465
35	1	0	3.146061	1.122221	-1.918015
36	6	0	6.006264	-1.479487	-1.584334
37	1	0	4.561664	-2.723749	-0.584853
38	6	0	6.240803	-0.256302	-2.222605
39	1	0	5.386186	1.626463	-2.843756
40	1	0	6.804613	-2.211423	-1.497344
41	1	0	7.224769	-0.036844	-2.628267
42	6	0	1.677363	-2.860924	-1.171352
43	6	0	1.947678	-3.141370	-2.523550
44	6	0	1.093612	-3.854505	-0.370847
45	6	0	1.658299	-4.397159	-3.054694
46	1	0	2.399340	-2.383574	-3.158655
47	6	0	0.798084	-5.107415	-0.910977
48	1	0	0.870505	-3.662213	0.672384
49	6	0	1.081341	-5.384018	-2.249902
50	1	0	1.881811	-4.604171	-4.097809
51	1	0	0.344427	-5.867342	-0.280843
52	1	0	0.848683	-6.360216	-2.666151
53	6	0	-3.704387	0.271321	-1.350059
54	6	0	-4.921089	-0.399763	-1.557947
55	6	0	-3.609935	1.635668	-1.666968
56	6	0	-6.026428	0.291088	-2.056745
57	1	0	-5.004583	-1.459069	-1.332602
58	6	0	-4.719356	2.325315	-2.160373
59	1	0	-2.667567	2.157799	-1.537127
60	6	0	-5.928808	1.654158	-2.354796
61	1	0	-6.963673	-0.235629	-2.214991
62	1	0	-4.631118	3.381329	-2.400412

63	1	0	-6.791324	2.187639	-2.745056
64	6	0	-2.363991	-2.295594	-1.323926
65	6	0	-1.875744	-2.574914	-2.611396
66	6	0	-2.951140	-3.324744	-0.573073
67	6	0	-1.991743	-3.857563	-3.145499
68	1	0	-1.399470	-1.788615	-3.193167
69	6	0	-3.057899	-4.610531	-1.108974
70	1	0	-3.326599	-3.125552	0.425989
71	6	0	-2.582801	-4.877913	-2.394579
72	1	0	-1.607554	-4.064431	-4.140133
73	1	0	-3.513666	-5.402017	-0.520031
74	1	0	-2.663067	-5.879780	-2.807356
75	46	0	0.082377	0.118789	-1.055242
76	15	0	0.430389	2.287291	-0.127377
77	6	0	-0.330134	2.551014	1.524184
78	6	0	-1.730248	2.458102	1.620058
79	6	0	0.415513	2.792026	2.688387
80	6	0	-2.368332	2.614706	2.848285
81	1	0	-2.324908	2.275685	0.732231
82	6	0	-0.227902	2.930770	3.920941
83	1	0	1.493956	2.886319	2.646121
84	6	0	-1.618536	2.843692	4.005452
85	1	0	-3.449801	2.541805	2.902002
86	1	0	0.363428	3.118868	4.812914
87	1	0	-2.116072	2.953589	4.964970
88	6	0	2.169276	2.853592	0.009580
89	6	0	2.664418	3.916817	-0.761423
90	6	0	3.051570	2.147113	0.845966
91	6	0	4.015846	4.265334	-0.689875
92	1	0	2.004380	4.473585	-1.418437
93	6	0	4.394245	2.509910	0.927419
94	1	0	2.692988	1.306355	1.428339
95	6	0	4.882141	3.567544	0.154870
96	1	0	4.388012	5.088474	-1.293758

97	1	0	5.060826	1.953069	1.580110
98	1	0	5.931976	3.842221	0.206729
99	6	0	-0.384185	3.494473	-1.241257
100	6	0	-1.025926	4.650862	-0.773100
101	6	0	-0.370469	3.223140	-2.620865
102	6	0	-1.657233	5.512809	-1.672581
103	1	0	-1.043075	4.876051	0.288442
104	6	0	-0.993113	4.092181	-3.516634
105	1	0	0.115598	2.321303	-2.987961
106	6	0	-1.643530	5.235418	-3.042240
107	1	0	-2.158530	6.402385	-1.301257
108	1	0	-0.980956	3.871255	-4.580251
109	1	0	-2.138785	5.907095	-3.737956

INT-1

Charge = 0 Multiplicity = 2

Center Number	Atomic Number	Atomic Type	Coordinates (Angstroms) X	Y	Z
1	6	0	3.701591	-0.308823	-0.912252
2	6	0	4.175528	0.204548	0.298745
3	6	0	3.283865	0.454956	1.345244
4	6	0	1.920158	0.210304	1.175680
5	6	0	1.440046	-0.299859	-0.039082
6	6	0	2.341823	-0.574171	-1.074737
7	1	0	4.392575	-0.509581	-1.726663
8	1	0	5.236138	0.403299	0.428027
9	1	0	3.649424	0.839665	2.293650
10	1	0	1.223718	0.404777	1.987102
11	1	0	1.968709	-0.996804	-2.002986
12	6	0	-0.005661	-0.699966	-0.213722
13	8	0	-0.238832	-1.846321	-0.609067

14	7	0	-0.980743	0.204846	0.140997
15	6	0	-2.415078	-0.256088	0.245149
16	6	0	-2.971947	-0.640887	-1.138002
17	6	0	-2.488535	-1.450204	1.218019
18	6	0	-3.286079	0.866177	0.837338
19	1	0	-2.974033	0.226388	-1.807578
20	1	0	-2.372573	-1.433612	-1.588788
21	1	0	-4.005239	-0.995192	-1.038992
22	1	0	-2.060948	-1.175619	2.189579
23	1	0	-3.537088	-1.727445	1.374284
24	1	0	-1.955761	-2.320687	0.835873
25	1	0	-4.304439	0.482257	0.956947
26	1	0	-2.931138	1.180873	1.825187
27	1	0	-3.342915	1.743558	0.185654
28	6	0	-0.709851	1.655663	0.002366
29	6	0	-1.147616	2.220379	-1.307872
30	1	0	0.369607	1.797629	0.118036
31	1	0	-1.166090	2.196142	0.834519
32	1	0	-0.905448	1.697200	-2.228303
33	1	0	-1.538106	3.231624	-1.371247

CO_2

Charge = 0 Multiplicity = 1

Center Number	Atomic Number	Atomic Type	Coordinates (Angstroms) X	Y	Z
1	6	0	0.000000	0.000000	0.000000
2	8	0	0.000000	0.000000	1.169078
3	8	0	0.000000	0.000000	-1.169078

TS-1

Charge = 0 Multiplicity = 2

Center Number	Atomic Number	Atomic Type	Coordinates (Angstroms) X	Y	Z
1	6	0	3.603148	-0.713475	-0.609504
2	6	0	4.036522	0.109428	0.442992
3	6	0	3.090365	0.718026	1.279631
4	6	0	1.732047	0.550408	1.045437
5	6	0	1.276293	-0.192247	-0.085006
6	6	0	2.250223	-0.904083	-0.842806
7	1	0	4.333832	-1.224977	-1.231404
8	1	0	5.098394	0.245680	0.628268
9	1	0	3.419792	1.313743	2.127491
10	1	0	0.999921	1.011300	1.703291
11	1	0	1.912485	-1.571125	-1.630656
12	6	0	-0.156053	-0.710397	-0.066451
13	8	0	-0.366394	-1.919699	0.049238
14	7	0	-1.120781	0.254873	-0.121131
15	6	0	-2.558268	-0.052451	0.174035
16	6	0	-3.135997	-0.919538	-0.957628
17	6	0	-2.658886	-0.774871	1.531816
18	6	0	-3.366823	1.252498	0.283671
19	1	0	-3.054407	-0.397502	-1.918688
20	1	0	-2.595398	-1.866223	-1.027358
21	1	0	-4.196196	-1.130501	-0.773668
22	1	0	-2.243365	-0.145834	2.328226
23	1	0	-3.711571	-0.970839	1.765111
24	1	0	-2.123533	-1.724538	1.523572
25	1	0	-4.379185	1.004062	0.618082
26	1	0	-2.931516	1.940091	1.017075
27	1	0	-3.462198	1.775339	-0.672718
28	6	0	-0.701155	1.571914	-0.648917

29	6	0	0.576143	1.402765	-1.406970
30	1	0	-0.589314	2.299851	0.163512
31	1	0	-1.487936	1.943488	-1.315153
32	1	0	0.520956	0.873557	-2.356302
33	1	0	1.340519	2.170048	-1.336479

INT-2

Charge = 0 Multiplicity = 2

Center Number	Atomic Number	Atomic Type	Coordinates (Angstroms) X	Y	Z
1	6	0	3.470438	-0.469847	-0.913747
2	6	0	3.889666	-0.510595	0.441454
3	6	0	3.001454	-0.072888	1.456568
4	6	0	1.746155	0.380521	1.158978
5	6	0	1.212725	0.427265	-0.251573
6	6	0	2.223083	-0.032321	-1.260393
7	1	0	4.159545	-0.792581	-1.691483
8	1	0	4.883686	-0.863821	0.697861
9	1	0	3.331857	-0.087842	2.493166
10	1	0	1.080301	0.716620	1.950900
11	1	0	1.914236	-0.011743	-2.303134
12	6	0	-0.069986	-0.480347	-0.280005
13	8	0	-0.040920	-1.704494	-0.346327
14	7	0	-1.174467	0.313142	-0.168060
15	6	0	-2.538707	-0.239297	0.095813
16	6	0	-2.939828	-1.171076	-1.061919
17	6	0	-2.517709	-1.001983	1.432907
18	6	0	-3.558174	0.906805	0.182872
19	1	0	-2.935180	-0.621851	-2.010962
20	1	0	-2.251438	-2.013697	-1.144352
21	1	0	-3.951899	-1.557098	-0.893949

22	1	0	-2.248834	-0.328146	2.255414
23	1	0	-3.507610	-1.421787	1.645585
24	1	0	-1.793211	-1.819198	1.400609
25	1	0	-4.546006	0.480091	0.385658
26	1	0	-3.325595	1.604421	0.994237
27	1	0	-3.623892	1.468005	-0.755554
28	6	0	-0.834245	1.735040	-0.027065
29	6	0	0.590071	1.813132	-0.585676
30	1	0	-0.878213	2.045137	1.025790
31	1	0	-1.525290	2.363314	-0.592043
32	1	0	0.552672	1.934813	-1.674354
33	1	0	1.163836	2.642868	-0.165637

INT-3

Charge = 0 Multiplicity = 1

Center Number	Atomic Number	Atomic Type	Coordinates (Angstroms) X	Y	Z
1	6	0	-4.208925	0.455496	-1.032153
2	6	0	-3.037100	0.725295	-0.312424
3	6	0	-2.952574	0.214617	0.987432
4	6	0	-3.970831	-0.515073	1.607374
5	6	0	-5.120447	-0.771126	0.854170
6	6	0	-5.236720	-0.290227	-0.453422
7	6	0	-3.762110	-0.906740	3.073710
8	6	0	-2.279690	-1.258029	3.249263
9	6	0	-1.343726	-0.504750	2.530047
10	6	0	0.039602	-0.671023	2.639325
11	6	0	0.496217	-1.630262	3.557257
12	6	0	-0.406824	-2.407371	4.282064
13	6	0	-1.783915	-2.230572	4.121470
14	1	0	-4.306404	0.789476	-2.057253

15	1	0	-5.935503	-1.344121	1.283064
16	1	0	-6.135942	-0.498550	-1.026390
17	1	0	1.560938	-1.788281	3.687977
18	1	0	-0.036215	-3.155947	4.976686
19	1	0	-2.468500	-2.848201	4.692901
20	8	0	-1.780735	0.468044	1.662011
21	6	0	-4.689435	-2.050046	3.507407
22	1	0	-4.518650	-2.955650	2.916762
23	1	0	-4.537874	-2.290571	4.563934
24	1	0	-5.738342	-1.757442	3.398651
25	6	0	-4.064209	0.343144	3.947944
26	1	0	-3.895488	0.114400	5.006735
27	1	0	-3.420189	1.185654	3.674034
28	1	0	-5.107853	0.652938	3.818258
29	15	0	-1.515700	1.532429	-0.954644
30	15	0	1.168083	0.313743	1.545029
31	6	0	-1.999637	2.557953	-2.411126
32	6	0	-0.990325	2.880210	-3.330744
33	6	0	-3.255862	3.173148	-2.541276
34	6	0	-1.237831	3.773183	-4.375441
35	1	0	-0.006555	2.435783	-3.221209
36	6	0	-3.507282	4.051917	-3.596315
37	1	0	-4.036447	2.991633	-1.810210
38	6	0	-2.500974	4.350248	-4.519573
39	1	0	-0.443245	4.010819	-5.077890
40	1	0	-4.486056	4.515944	-3.686219
41	1	0	-2.697490	5.038739	-5.337263
42	6	0	-1.238558	2.948693	0.208899
43	6	0	0.006228	3.593636	0.178004
44	6	0	-2.252725	3.454300	1.035374
45	6	0	0.224777	4.734076	0.953522
46	1	0	0.815282	3.195161	-0.430015
47	6	0	-2.022442	4.582106	1.824333
48	1	0	-3.223752	2.969692	1.070714

49	6	0	-0.783446	5.227564	1.783050
50	1	0	1.195876	5.218463	0.928259
51	1	0	-2.812942	4.956485	2.470078
52	1	0	-0.602880	6.104972	2.398404
53	6	0	2.679505	-0.744795	1.650342
54	6	0	3.884072	-0.373870	2.260711
55	6	0	2.581764	-2.017224	1.059206
56	6	0	4.972447	-1.253485	2.271503
57	1	0	3.985331	0.597048	2.733156
58	6	0	3.660159	-2.897981	1.087217
59	1	0	1.662364	-2.310699	0.561416
60	6	0	4.863935	-2.516327	1.689166
61	1	0	5.903918	-0.945524	2.739371
62	1	0	3.567766	-3.871788	0.614770
63	1	0	5.711364	-3.196650	1.695725
64	6	0	1.554370	1.789407	2.583133
65	6	0	2.593349	2.650621	2.184411
66	6	0	0.802151	2.119601	3.720647
67	6	0	2.898483	3.785756	2.935576
68	1	0	3.152404	2.442766	1.277678
69	6	0	1.090907	3.274606	4.451599
70	1	0	-0.010642	1.477436	4.044646
71	6	0	2.146520	4.104925	4.069738
72	1	0	3.713609	4.432650	2.620887
73	1	0	0.493038	3.517915	5.326276
74	1	0	2.376088	4.997655	4.645722
75	46	0	0.483474	0.289409	-1.089800
76	6	0	4.857400	0.408427	-1.294263
77	6	0	4.765140	-0.933439	-1.656590
78	6	0	5.891959	-1.730046	-1.787622
79	6	0	7.137599	-1.132892	-1.536895
80	6	0	7.230399	0.216910	-1.171480
81	6	0	6.080750	1.013019	-1.047278
82	6	0	3.452109	0.932444	-1.226291

83	6	0	3.301138	-1.247162	-1.800430
84	1	0	5.810888	-2.777432	-2.063459
85	1	0	8.044900	-1.724858	-1.623701
86	1	0	8.208064	0.651420	-0.980283
87	1	0	6.144527	2.059031	-0.761375
88	8	0	3.132783	2.091539	-0.947216
89	8	0	2.839642	-2.341462	-2.121370
90	7	0	2.583500	-0.099992	-1.508068
91	6	0	-0.111154	-0.151428	-4.045098
92	6	0	-1.325542	0.049547	-4.624637
93	6	0	-2.519766	-0.486797	-4.000017
94	6	0	-2.437992	-1.378424	-2.989893
95	6	0	-1.092512	-1.939635	-2.583765
96	6	0	0.038136	-0.938061	-2.833107
97	1	0	0.780192	0.320611	-4.452930
98	1	0	-1.426464	0.676476	-5.505693
99	1	0	-3.490823	-0.141815	-4.348852
100	1	0	-3.330525	-1.766525	-2.507857
101	1	0	0.995239	-1.445811	-2.765018
102	6	0	-1.162577	-2.492440	-1.142617
103	8	0	-1.067942	-1.815461	-0.116696
104	7	0	-1.387119	-3.833740	-1.180351
105	6	0	-1.692523	-4.630151	0.045623
106	6	0	-0.512989	-4.522012	1.031377
107	6	0	-2.982653	-4.086051	0.686755
108	6	0	-1.891631	-6.105294	-0.331009
109	1	0	0.414290	-4.861852	0.555034
110	1	0	-0.376281	-3.495997	1.372776
111	1	0	-0.702422	-5.156883	1.904747
112	1	0	-3.823863	-4.159643	-0.013230
113	1	0	-3.230304	-4.666024	1.583282
114	1	0	-2.856412	-3.040922	0.972733
115	1	0	-2.135620	-6.667936	0.576261
116	1	0	-2.715977	-6.243782	-1.038977

117	1	0	-0.983435	-6.543122	-0.759358
118	6	0	-1.528236	-4.336475	-2.553835
119	6	0	-0.823609	-3.258190	-3.381745
120	1	0	-2.587909	-4.435840	-2.826100
121	1	0	-1.053375	-5.312394	-2.667899
122	1	0	0.253970	-3.455800	-3.388603
123	1	0	-1.179940	-3.204377	-4.412625

INT-4

Charge = 1 Multiplicity = 1

Center Number	Atomic Number	Atomic Type	Coordinates (Angstroms) X	Y	Z
1	6	0	-0.928481	2.401267	-2.285080
2	6	0	-0.287278	3.253506	-1.350685
3	6	0	-1.095143	3.869806	-0.297967
4	6	0	-2.353729	3.460817	-0.053608
5	6	0	-2.946710	2.249401	-0.730323
6	6	0	-2.053400	1.699681	-1.829095
7	1	0	-0.480267	2.177155	-3.249195
8	1	0	0.656045	3.722022	-1.610885
9	1	0	-0.664666	4.703481	0.247109
10	1	0	-2.972157	3.963449	0.685777
11	1	0	-2.504209	0.954492	-2.475633
12	6	0	-3.200683	1.242963	0.440062
13	8	0	-2.310921	0.594357	0.992591
14	7	0	-4.514919	1.267758	0.774361
15	6	0	-5.034822	0.655247	2.041581
16	6	0	-4.738447	-0.854998	2.046825
17	6	0	-4.358444	1.349835	3.236839
18	6	0	-6.555719	0.854161	2.122005
19	1	0	-5.216233	-1.345454	1.190745

20	1	0	-3.666552	-1.054329	2.014744
21	1	0	-5.145506	-1.300779	2.961419
22	1	0	-4.564212	2.426882	3.224135
23	1	0	-4.742631	0.940169	4.177555
24	1	0	-3.275913	1.199577	3.212611
25	1	0	-6.917757	0.390699	3.045376
26	1	0	-6.837382	1.911675	2.150170
27	1	0	-7.071932	0.374111	1.283441
28	6	0	-5.270475	2.264780	-0.000139
29	6	0	-4.393000	2.497162	-1.235146
30	1	0	-5.404021	3.182507	0.586519
31	1	0	-6.257014	1.886153	-0.271272
32	1	0	-4.635161	1.752328	-2.000212
33	1	0	-4.524217	3.492764	-1.665353
34	6	0	3.266228	1.036420	2.757257
35	6	0	2.521425	0.392690	1.755487
36	6	0	2.019116	-0.880422	2.044907
37	6	0	2.166967	-1.511162	3.285485
38	6	0	2.885342	-0.822795	4.267131
39	6	0	3.442515	0.430997	4.000256
40	6	0	1.597228	-2.926123	3.440926
41	6	0	0.308425	-3.000777	2.614320
42	6	0	0.278433	-2.308254	1.402540
43	6	0	-0.779472	-2.370815	0.490394
44	6	0	-1.861241	-3.201144	0.812149
45	6	0	-1.878980	-3.884987	2.029226
46	6	0	-0.810477	-3.781586	2.924005
47	1	0	3.691762	2.016638	2.574370
48	1	0	3.026444	-1.266751	5.246482
49	1	0	4.013162	0.941568	4.770463
50	1	0	-2.690896	-3.314184	0.124747
51	1	0	-2.729200	-4.513712	2.277177
52	1	0	-0.846057	-4.333475	3.856853
53	8	0	1.349000	-1.522352	1.028098

54	6	0	1.367602	-3.300474	4.912216
55	1	0	0.658310	-2.620854	5.396584
56	1	0	0.982979	-4.321268	4.995068
57	1	0	2.309602	-3.274055	5.468013
58	6	0	2.615394	-3.923303	2.817980
59	1	0	2.228072	-4.946660	2.880926
60	1	0	2.805410	-3.693037	1.763951
61	1	0	3.567998	-3.875491	3.357840
62	15	0	2.126577	1.176486	0.134839
63	15	0	-0.586004	-1.352956	-1.024606
64	6	0	2.588815	2.927565	0.471854
65	6	0	1.738405	3.656287	1.321181
66	6	0	3.731988	3.548112	-0.048059
67	6	0	2.022642	4.983276	1.638249
68	1	0	0.852354	3.181191	1.733604
69	6	0	4.007836	4.884116	0.262177
70	1	0	4.411544	2.999537	-0.691612
71	6	0	3.157036	5.603318	1.102744
72	1	0	1.358354	5.534051	2.298816
73	1	0	4.894482	5.357153	-0.151352
74	1	0	3.376249	6.640025	1.343265
75	6	0	3.400503	0.563325	-1.032423
76	6	0	3.429685	1.117969	-2.324055
77	6	0	4.311364	-0.445909	-0.696724
78	6	0	4.379854	0.693273	-3.250853
79	1	0	2.714186	1.889508	-2.598368
80	6	0	5.243976	-0.888967	-1.638463
81	1	0	4.299600	-0.887826	0.294735
82	6	0	5.287165	-0.315633	-2.910255
83	1	0	4.404487	1.138954	-4.241615
84	1	0	5.940514	-1.678989	-1.371770
85	1	0	6.018877	-0.657372	-3.637127
86	6	0	-2.111641	-1.670431	-2.010752
87	6	0	-2.042419	-2.030824	-3.364999

88	6	0	-3.372782	-1.416382	-1.442273
89	6	0	-3.208357	-2.133221	-4.129869
90	1	0	-1.083998	-2.232126	-3.831988
91	6	0	-4.534317	-1.527836	-2.205593
92	1	0	-3.446772	-1.130776	-0.402311
93	6	0	-4.456191	-1.882908	-3.555644
94	1	0	-3.136716	-2.414907	-5.176995
95	1	0	-5.498220	-1.331749	-1.743126
96	1	0	-5.359869	-1.964810	-4.153243
97	6	0	0.730856	-2.237850	-1.955233
98	6	0	1.415569	-1.550250	-2.967853
99	6	0	1.052899	-3.579377	-1.698470
100	6	0	2.399354	-2.194984	-3.718882
101	1	0	1.183726	-0.507702	-3.164384
102	6	0	2.048859	-4.216679	-2.439882
103	1	0	0.532442	-4.128167	-0.919705
104	6	0	2.722045	-3.527262	-3.451936
105	1	0	2.925758	-1.649754	-4.495851
106	1	0	2.296438	-5.253252	-2.227225
107	1	0	3.498117	-4.025110	-4.027023
108	46	0	-0.086436	1.013901	-0.803848

NPhth-anion

Charge = -1 Multiplicity = 1

Center Number	Atomic Number	Atomic Type	Coordinates (Angstroms) X	Y	Z
1	6	0	0.000000	0.695536	-0.143143
2	6	0	0.000000	-0.695536	-0.143143
3	6	0	0.000000	-1.420497	-1.325766
4	6	0	0.000000	-0.700530	-2.532227
5	6	0	0.000000	0.700530	-2.532227

6	6	0	0.000000	1.420497	-1.325766
7	6	0	0.000000	1.114345	1.318768
8	6	0	0.000000	-1.114345	1.318768
9	1	0	0.000000	-2.507720	-1.317742
10	1	0	0.000000	-1.234142	-3.479754
11	1	0	0.000000	1.234142	-3.479754
12	1	0	0.000000	2.507720	-1.317742
13	8	0	0.000000	2.295464	1.684656
14	8	0	0.000000	-2.295464	1.684656
15	7	0	0.000000	0.000000	2.118416

TS-2a-1

Charge = 0 Multiplicity = 1

Center Number	Atomic Number	Atomic Type	Coordinates (Angstroms) X	Y	Z
1	6	0	-2.115706	-0.993928	-0.683635
2	6	0	-1.400143	-1.485778	-1.817114
3	6	0	-1.509248	-0.747247	-3.072932
4	6	0	-2.078638	0.472565	-3.136537
5	6	0	-2.554103	1.193339	-1.901810
6	6	0	-2.724814	0.269283	-0.724348
7	1	0	-2.340234	-1.646649	0.154814
8	1	0	-1.174076	-2.546065	-1.882389
9	1	0	-1.145893	-1.227595	-3.976595
10	1	0	-2.165825	1.002861	-4.081756
11	1	0	-3.055482	0.732653	0.195264
12	6	0	-1.432352	2.271928	-1.619788
13	8	0	-0.309105	2.020616	-1.185947
14	7	0	-1.882548	3.493845	-2.001890
15	6	0	-0.964626	4.670397	-2.142281
16	6	0	-0.270918	4.957237	-0.796950

17	6	0	0.072557	4.360250	-3.236907
18	6	0	-1.782802	5.907878	-2.542354
19	1	0	-1.014268	5.134654	-0.010944
20	1	0	0.371799	4.131684	-0.491459
21	1	0	0.344011	5.859536	-0.892017
22	1	0	-0.424920	4.150595	-4.191390
23	1	0	0.738829	5.218418	-3.381094
24	1	0	0.681066	3.494078	-2.962709
25	1	0	-1.104796	6.763469	-2.625843
26	1	0	-2.278144	5.783764	-3.510728
27	1	0	-2.540059	6.150936	-1.788812
28	6	0	-3.188498	3.419447	-2.671419
29	6	0	-3.776276	2.104726	-2.155788
30	1	0	-3.054792	3.419299	-3.761815
31	1	0	-3.814782	4.272697	-2.406263
32	1	0	-4.301247	2.266158	-1.211661
33	1	0	-4.478685	1.650460	-2.855838
34	6	0	4.543830	-0.911068	-2.289786
35	6	0	3.668092	-0.743788	-1.204322
36	6	0	3.987399	0.254627	-0.274280
37	6	0	5.088520	1.110807	-0.401854
38	6	0	5.915332	0.931608	-1.514378
39	6	0	5.652263	-0.079409	-2.442234
40	6	0	5.330900	2.127069	0.720057
41	6	0	3.957999	2.616682	1.193102
42	6	0	2.929895	1.673027	1.257996
43	6	0	1.652104	1.943041	1.761143
44	6	0	1.408038	3.240269	2.232247
45	6	0	2.402642	4.217215	2.152923
46	6	0	3.664806	3.910640	1.637195
47	1	0	4.349660	-1.680705	-3.028198
48	1	0	6.777806	1.572927	-1.659727
49	1	0	6.314247	-0.216509	-3.292582
50	1	0	0.440581	3.494419	2.650079

51	1	0	2.195647	5.223296	2.506715
52	1	0	4.424349	4.684090	1.597296
53	8	0	3.157813	0.381602	0.820738
54	6	0	6.238863	3.282053	0.275868
55	1	0	5.808864	3.834409	-0.566581
56	1	0	6.403131	3.981193	1.101412
57	1	0	7.223962	2.908307	-0.019772
58	6	0	6.006377	1.380434	1.905566
59	1	0	6.167688	2.068394	2.743609
60	1	0	5.385270	0.550616	2.259126
61	1	0	6.976002	0.975878	1.592916
62	15	0	2.089410	-1.692484	-1.021903
63	15	0	0.450880	0.550325	1.707316
64	6	0	2.025928	-2.572925	-2.642799
65	6	0	1.588275	-1.832019	-3.753722
66	6	0	2.408425	-3.909229	-2.823987
67	6	0	1.533992	-2.415403	-5.019146
68	1	0	1.287392	-0.796151	-3.623089
69	6	0	2.338556	-4.497241	-4.090826
70	1	0	2.761629	-4.496087	-1.982346
71	6	0	1.903520	-3.753770	-5.189566
72	1	0	1.195962	-1.828763	-5.869367
73	1	0	2.630911	-5.536551	-4.216475
74	1	0	1.851623	-4.213026	-6.173105
75	6	0	2.486275	-3.025079	0.183618
76	6	0	1.506445	-4.007755	0.413465
77	6	0	3.690945	-3.084333	0.895086
78	6	0	1.742841	-5.044921	1.313704
79	1	0	0.559146	-3.960592	-0.118271
80	6	0	3.914949	-4.111036	1.817277
81	1	0	4.459698	-2.335126	0.733137
82	6	0	2.948622	-5.096919	2.021630
83	1	0	0.982212	-5.804780	1.472630
84	1	0	4.850442	-4.140660	2.369527

85	1	0	3.127974	-5.897835	2.733854
86	6	0	-1.093811	1.253843	2.431843
87	6	0	-1.752919	0.635649	3.504582
88	6	0	-1.707998	2.355870	1.807370
89	6	0	-2.998450	1.102983	3.935919
90	1	0	-1.304480	-0.216808	4.004804
91	6	0	-2.943883	2.826073	2.247815
92	1	0	-1.222613	2.837456	0.967276
93	6	0	-3.598594	2.195976	3.310284
94	1	0	-3.498019	0.605572	4.763398
95	1	0	-3.405723	3.670954	1.744713
96	1	0	-4.573024	2.547560	3.636609
97	6	0	1.080536	-0.556931	3.038756
98	6	0	0.709251	-1.909145	3.004556
99	6	0	1.902185	-0.100093	4.080717
100	6	0	1.141471	-2.787281	4.000040
101	1	0	0.090645	-2.274871	2.190033
102	6	0	2.347576	-0.983210	5.065853
103	1	0	2.200399	0.943469	4.122521
104	6	0	1.965788	-2.327268	5.029451
105	1	0	0.849793	-3.832150	3.958259
106	1	0	2.990738	-0.619926	5.863356
107	1	0	2.313547	-3.013684	5.797068
108	46	0	0.100798	-0.571054	-0.421891
109	6	0	-6.279581	-2.007020	-0.047531
110	6	0	-6.553259	-0.855170	0.687870
111	6	0	-7.500398	-0.846128	1.701328
112	6	0	-8.184080	-2.045153	1.959418
113	6	0	-7.912288	-3.203139	1.217901
114	6	0	-6.948750	-3.197635	0.196219
115	6	0	-5.200386	-1.638877	-1.041060
116	6	0	-5.637696	0.219033	0.155857
117	1	0	-7.702843	0.055477	2.273026
118	1	0	-8.934544	-2.078891	2.745166

119	1	0	-8.456553	-4.117727	1.439367
120	1	0	-6.732383	-4.092548	-0.380945
121	7	0	-4.896657	-0.299698	-0.884455
122	8	0	-4.685246	-2.416716	-1.841826
123	8	0	-5.561980	1.366993	0.602061

TS-2a-2

Charge = 0 Multiplicity = 1

Center Number	Atomic Number	Atomic Type	Coordinates (Angstroms) X	Y	Z
1	6	0	-2.181066	0.645462	0.651630
2	6	0	-2.856631	0.229954	-0.525099
3	6	0	-2.807504	1.069480	-1.716922
4	6	0	-2.189259	2.260367	-1.698735
5	6	0	-1.415022	2.780155	-0.509241
6	6	0	-1.354325	1.788311	0.641939
7	1	0	-2.382097	0.121239	1.579557
8	1	0	-3.162256	-0.800453	-0.626765
9	1	0	-3.371970	0.732518	-2.578707
10	1	0	-2.214978	2.901745	-2.577560
11	1	0	-1.031137	2.206187	1.591198
12	6	0	-0.034941	3.183018	-1.108842
13	8	0	0.844634	2.381892	-1.424053
14	7	0	-0.003015	4.532462	-1.300960
15	6	0	1.018450	5.195152	-2.173941
16	6	0	2.435540	4.907897	-1.643564
17	6	0	0.855062	4.662892	-3.609142
18	6	0	0.797148	6.715729	-2.158145
19	1	0	2.550730	5.286492	-0.621332
20	1	0	2.657809	3.840501	-1.649026
21	1	0	3.168792	5.420358	-2.276775
22	1	0	-0.152665	4.875565	-3.985739

23	1	0	1.577348	5.143290	-4.278918
24	1	0	1.020417	3.582469	-3.641324
25	1	0	1.565075	7.187320	-2.780119
26	1	0	-0.178154	6.999991	-2.566050
27	1	0	0.887684	7.125661	-1.145987
28	6	0	-1.289016	5.169322	-0.977368
29	6	0	-1.964275	4.157808	-0.046295
30	1	0	-1.872794	5.339385	-1.891748
31	1	0	-1.137654	6.131967	-0.485970
32	1	0	-1.641217	4.337269	0.984301
33	1	0	-3.054940	4.209075	-0.081352
34	6	0	1.665041	-3.272441	-2.946626
35	6	0	1.750606	-2.540544	-1.751289
36	6	0	2.994280	-1.979763	-1.432330
37	6	0	4.124083	-2.078839	-2.253973
38	6	0	3.987217	-2.790429	-3.449350
39	6	0	2.771432	-3.391185	-3.786532
40	6	0	5.429678	-1.457835	-1.743399
41	6	0	5.062106	-0.165787	-1.005993
42	6	0	3.894221	-0.175562	-0.239171
43	6	0	3.479333	0.892882	0.565482
44	6	0	4.304358	2.025705	0.604926
45	6	0	5.464684	2.079450	-0.169699
46	6	0	5.838878	0.997474	-0.970999
47	1	0	0.725732	-3.733697	-3.231250
48	1	0	4.831916	-2.890474	-4.122243
49	1	0	2.685897	-3.952426	-4.712742
50	1	0	4.040366	2.869760	1.231887
51	1	0	6.088381	2.968484	-0.140398
52	1	0	6.750566	1.060520	-1.555496
53	8	0	3.087091	-1.297523	-0.236555
54	6	0	6.441147	-1.215941	-2.871970
55	1	0	6.044505	-0.534731	-3.632462
56	1	0	7.366702	-0.789303	-2.473529

57	1	0	6.712112	-2.158799	-3.356869
58	6	0	6.053747	-2.434625	-0.706766
59	1	0	6.973190	-2.006989	-0.290272
60	1	0	5.362847	-2.630496	0.120167
61	1	0	6.297806	-3.389832	-1.185853
62	15	0	0.292377	-2.197468	-0.668650
63	15	0	1.871878	0.679999	1.436489
64	6	0	-1.089207	-2.811144	-1.728722
65	6	0	-1.570553	-1.934936	-2.716508
66	6	0	-1.669607	-4.081253	-1.604617
67	6	0	-2.615288	-2.317523	-3.556452
68	1	0	-1.134442	-0.944362	-2.815519
69	6	0	-2.726918	-4.457954	-2.438853
70	1	0	-1.305459	-4.778491	-0.856885
71	6	0	-3.202413	-3.578820	-3.413620
72	1	0	-2.982655	-1.626383	-4.310061
73	1	0	-3.176612	-5.440916	-2.324514
74	1	0	-4.028199	-3.871128	-4.056621
75	6	0	0.406133	-3.446220	0.677257
76	6	0	-0.687591	-3.558468	1.554629
77	6	0	1.538169	-4.241413	0.895018
78	6	0	-0.659478	-4.471311	2.607219
79	1	0	-1.565930	-2.934657	1.406062
80	6	0	1.574568	-5.136158	1.968599
81	1	0	2.394824	-4.168973	0.231955
82	6	0	0.476004	-5.260654	2.820178
83	1	0	-1.517335	-4.557298	3.269054
84	1	0	2.463173	-5.740052	2.132606
85	1	0	0.504392	-5.963040	3.648979
86	6	0	1.665753	2.239299	2.400248
87	6	0	1.450971	2.240475	3.787052
88	6	0	1.570080	3.457434	1.703890
89	6	0	1.148722	3.430520	4.456258
90	1	0	1.512865	1.316084	4.352274

91	6	0	1.278152	4.644762	2.374045
92	1	0	1.727261	3.473801	0.634208
93	6	0	1.061711	4.635298	3.755689
94	1	0	0.983381	3.411927	5.530436
95	1	0	1.210723	5.573103	1.812224
96	1	0	0.825314	5.557251	4.280173
97	6	0	2.285997	-0.562763	2.733184
98	6	0	1.237878	-1.285739	3.322542
99	6	0	3.601878	-0.798147	3.160381
100	6	0	1.498966	-2.219594	4.326620
101	1	0	0.217237	-1.123124	2.987702
102	6	0	3.862796	-1.745178	4.152256
103	1	0	4.425642	-0.246702	2.716938
104	6	0	2.812274	-2.455467	4.739511
105	1	0	0.678240	-2.774694	4.770233
106	1	0	4.887240	-1.924943	4.468222
107	1	0	3.016605	-3.192591	5.511653
108	46	0	-0.062880	0.022304	0.159488
109	6	0	-6.962556	0.013590	1.054478
110	6	0	-7.182034	-0.335597	-0.277214
111	6	0	-8.396722	-0.854428	-0.701252
112	6	0	-9.404281	-1.016337	0.263409
113	6	0	-9.183481	-0.666050	1.602350
114	6	0	-7.948317	-0.142786	2.017854
115	6	0	-5.545784	0.535816	1.138754
116	6	0	-5.901169	-0.033001	-1.018657
117	1	0	-8.560805	-1.125388	-1.740646
118	1	0	-10.370481	-1.418631	-0.030185
119	1	0	-9.981560	-0.802534	2.327630
120	1	0	-7.769985	0.130657	3.054253
121	7	0	-5.006908	0.522860	-0.129944
122	8	0	-4.988183	0.901925	2.172993
123	8	0	-5.701173	-0.261157	-2.212531

TS-2a-3

Charge = 0 Multiplicity = 1

Center Number	Atomic Number	Atomic Type	Coordinates (Angstroms) X	Y	Z
1	6	0	1.501910	1.187616	-0.406386
2	6	0	0.890164	1.994552	-1.390698
3	6	0	1.260022	1.804675	-2.790744
4	6	0	2.190424	0.901655	-3.153149
5	6	0	2.941223	0.046842	-2.172601
6	6	0	2.457289	0.197890	-0.742632
7	1	0	1.331940	1.403136	0.642837
8	1	0	0.409621	2.926854	-1.114728
9	1	0	0.768831	2.423374	-3.536630
10	1	0	2.484403	0.797120	-4.194947
11	1	0	2.547217	-0.686313	-0.128099
12	6	0	2.981145	-1.455134	-2.546240
13	6	0	-5.119810	2.013456	-1.663180
14	6	0	-4.185142	1.237346	-0.962070
15	6	0	-4.588195	-0.036756	-0.544502
16	6	0	-5.859977	-0.566924	-0.777657
17	6	0	-6.757320	0.229172	-1.498204
18	6	0	-6.390115	1.504683	-1.936252
19	6	0	-6.191943	-1.922905	-0.144748
20	6	0	-4.921115	-2.780302	-0.181680
21	6	0	-3.688574	-2.136883	-0.013158
22	6	0	-2.458596	-2.806574	0.019464
23	6	0	-2.492615	-4.208783	-0.048528
24	6	0	-3.702799	-4.882242	-0.207773
25	6	0	-4.904402	-4.174053	-0.289226
26	1	0	-4.852261	3.008225	-2.002906
27	1	0	-7.755604	-0.136481	-1.712856
28	1	0	-7.104692	2.110233	-2.486605

29	1	0	-1.570909	-4.776731	0.006258
30	1	0	-3.710875	-5.966850	-0.268015
31	1	0	-5.832363	-4.720232	-0.418884
32	8	0	-3.650775	-0.770224	0.153006
33	6	0	-7.374770	-2.610456	-0.842006
34	1	0	-7.157847	-2.817286	-1.895568
35	1	0	-7.622213	-3.553847	-0.346603
36	1	0	-8.270970	-1.985246	-0.788253
37	6	0	-6.556764	-1.675700	1.346132
38	1	0	-6.764594	-2.628254	1.847191
39	1	0	-5.736476	-1.181946	1.878569
40	1	0	-7.446537	-1.039580	1.418780
41	15	0	-2.444195	1.738530	-0.636283
42	15	0	-0.900899	-1.813159	0.141590
43	6	0	-2.367349	3.401795	-1.416935
44	6	0	-1.951390	3.472000	-2.756268
45	6	0	-2.714715	4.583656	-0.745718
46	6	0	-1.888991	4.700362	-3.415497
47	1	0	-1.666956	2.561313	-3.277395
48	6	0	-2.639466	5.814006	-1.403155
49	1	0	-3.040675	4.546167	0.289434
50	6	0	-2.228339	5.874843	-2.737278
51	1	0	-1.564919	4.741515	-4.451964
52	1	0	-2.903554	6.724612	-0.871800
53	1	0	-2.168605	6.833612	-3.245357
54	6	0	-2.392167	2.089384	1.177884
55	6	0	-1.208193	2.642145	1.701206
56	6	0	-3.417206	1.731168	2.063486
57	6	0	-1.047838	2.812007	3.074954
58	1	0	-0.404896	2.938190	1.034173
59	6	0	-3.251264	1.895244	3.441832
60	1	0	-4.348698	1.320123	1.689973
61	6	0	-2.066569	2.426995	3.952368
62	1	0	-0.121684	3.231105	3.459567

63	1	0	-4.052411	1.601379	4.114994
64	1	0	-1.935897	2.541627	5.024992
65	6	0	0.370346	-3.065292	-0.336684
66	6	0	1.502811	-3.344638	0.440763
67	6	0	0.241735	-3.684791	-1.594984
68	6	0	2.478634	-4.231515	-0.025270
69	1	0	1.643793	-2.870116	1.405317
70	6	0	1.205184	-4.585075	-2.046389
71	1	0	-0.621066	-3.469803	-2.220177
72	6	0	2.331244	-4.859861	-1.262516
73	1	0	3.356083	-4.423070	0.585461
74	1	0	1.082867	-5.062958	-3.014840
75	1	0	3.089272	-5.552235	-1.618631
76	6	0	-0.707515	-1.590699	1.967185
77	6	0	0.375690	-0.831415	2.442868
78	6	0	-1.632364	-2.098034	2.891653
79	6	0	0.541153	-0.598143	3.807584
80	1	0	1.094929	-0.423422	1.743560
81	6	0	-1.475892	-1.848575	4.257668
82	1	0	-2.479599	-2.685731	2.553289
83	6	0	-0.390728	-1.101351	4.719543
84	1	0	1.391717	-0.017315	4.153500
85	1	0	-2.205889	-2.242146	4.960291
86	1	0	-0.272511	-0.908882	5.782586
87	46	0	-0.530642	0.324736	-0.917277
88	6	0	4.072488	1.889908	2.631754
89	6	0	4.247074	0.529866	2.880354
90	6	0	4.349150	0.032103	4.171711
91	6	0	4.272029	0.954373	5.227838
92	6	0	4.095715	2.322788	4.978348
93	6	0	3.992025	2.811262	3.665907
94	6	0	3.944132	2.046114	1.134569
95	6	0	4.218452	-0.157365	1.540725
96	1	0	4.474089	-1.031202	4.355996

97	1	0	4.345265	0.604600	6.254264
98	1	0	4.035247	3.013454	5.815406
99	1	0	3.847845	3.869379	3.465766
100	7	0	4.075294	0.792623	0.550076
101	8	0	3.709866	3.100510	0.555132
102	8	0	4.262505	-1.379857	1.372242
103	1	0	2.134211	-2.008873	-2.145321
104	1	0	2.978413	-1.556281	-3.637399
105	6	0	4.480968	0.399107	-2.270697
106	8	0	4.917153	1.526833	-2.448983
107	6	0	4.324399	-1.931696	-1.986311
108	1	0	4.696264	-2.799796	-2.532855
109	1	0	4.259582	-2.180493	-0.922274
110	7	0	5.188483	-0.761858	-2.188839
111	6	0	6.676372	-0.786910	-2.064725
112	6	0	7.159544	-2.238804	-1.921797
113	6	0	7.293260	-0.177832	-3.337503
114	6	0	7.106241	0.005370	-0.816253
115	1	0	6.727898	-2.728162	-1.042171
116	1	0	6.927896	-2.839599	-2.808139
117	1	0	8.247951	-2.235037	-1.799417
118	1	0	6.989774	0.864196	-3.454783
119	1	0	8.387528	-0.221811	-3.283374
120	1	0	6.970505	-0.736869	-4.224277
121	1	0	8.200042	0.009207	-0.737422
122	1	0	6.754674	1.037496	-0.869811
123	1	0	6.692129	-0.454245	0.086323

TS-2a-4

Charge = 0 Multiplicity = 1

Center Number	Atomic Number	Atomic Type	Coordinates (Angstroms) X	Y	Z

1	6	0	-1.591052	-0.582151	0.256299
2	6	0	-1.843241	-1.431935	-0.854508
3	6	0	-2.249938	-0.859856	-2.126280
4	6	0	-2.547718	0.449106	-2.219183
5	6	0	-2.466501	1.383019	-1.038170
6	6	0	-1.666626	0.817239	0.105868
7	1	0	-1.394944	-1.036097	1.221073
8	1	0	-1.513009	-2.456066	-0.818175
9	1	0	-2.344821	-1.523499	-2.978464
10	1	0	-2.906429	0.864995	-3.157442
11	1	0	-1.621768	1.443272	0.993469
12	6	0	-2.138295	2.819982	-1.470223
13	6	0	-3.957199	1.559540	-0.552382
14	6	0	4.050503	-3.104440	-1.846205
15	6	0	3.530321	-2.048026	-1.082029
16	6	0	4.401865	-1.003311	-0.750913
17	6	0	5.743111	-0.957923	-1.149911
18	6	0	6.216775	-2.019153	-1.926696
19	6	0	5.379177	-3.084487	-2.267742
20	6	0	6.594220	0.211579	-0.644983
21	6	0	5.698602	1.454938	-0.633636
22	6	0	4.359534	1.286765	-0.262194
23	6	0	3.449807	2.344973	-0.135812
24	6	0	3.939006	3.640248	-0.366088
25	6	0	5.265155	3.840927	-0.749198
26	6	0	6.135817	2.758193	-0.889041
27	1	0	3.413037	-3.937044	-2.123081
28	1	0	7.247453	-2.026972	-2.264026
29	1	0	5.766904	-3.904849	-2.865006
30	1	0	3.283956	4.496323	-0.254639
31	1	0	5.624230	4.849711	-0.932455
32	1	0	7.164235	2.938523	-1.182522
33	8	0	3.885672	0.020335	0.019819
34	6	0	7.860295	0.414555	-1.489310

35	1	0	7.619414	0.630968	-2.535731
36	1	0	8.460115	1.239512	-1.093537
37	1	0	8.493566	-0.477102	-1.456741
38	6	0	7.010780	-0.090899	0.822104
39	1	0	7.599466	0.740597	1.226395
40	1	0	6.134526	-0.231685	1.463480
41	1	0	7.617254	-1.003024	0.863858
42	15	0	1.763832	-1.944622	-0.558326
43	15	0	1.715124	1.942526	0.374456
44	6	0	0.993009	-3.271015	-1.574430
45	6	0	0.481594	-2.898021	-2.828618
46	6	0	0.890599	-4.608770	-1.165288
47	6	0	-0.121573	-3.843692	-3.657687
48	1	0	0.538864	-1.859421	-3.145266
49	6	0	0.274218	-5.551947	-1.991872
50	1	0	1.280899	-4.916161	-0.200169
51	6	0	-0.231798	-5.172696	-3.237298
52	1	0	-0.516802	-3.541056	-4.623694
53	1	0	0.190187	-6.583388	-1.660090
54	1	0	-0.714760	-5.907526	-3.875725
55	6	0	1.768690	-2.603552	1.164535
56	6	0	0.551213	-3.023565	1.732270
57	6	0	2.915345	-2.564268	1.970864
58	6	0	0.488158	-3.392705	3.075638
59	1	0	-0.358622	-3.076042	1.143517
60	6	0	2.846371	-2.931515	3.317475
61	1	0	3.866956	-2.244376	1.559260
62	6	0	1.634852	-3.344731	3.875210
63	1	0	-0.464239	-3.712454	3.489062
64	1	0	3.744039	-2.891017	3.928900
65	1	0	1.583649	-3.626838	4.923539
66	6	0	0.872227	3.576813	0.251105
67	6	0	0.141397	4.113319	1.322053
68	6	0	0.853026	4.246650	-0.985179

69	6	0	-0.585874	5.296378	1.160532
70	1	0	0.134197	3.611655	2.284607
71	6	0	0.144973	5.438002	-1.137657
72	1	0	1.392472	3.834576	-1.834169
73	6	0	-0.582198	5.965026	-0.065538
74	1	0	-1.150693	5.696072	1.998628
75	1	0	0.150074	5.947490	-2.097579
76	1	0	-1.142562	6.888119	-0.186758
77	6	0	1.947944	1.699740	2.194161
78	6	0	1.312561	0.633589	2.846024
79	6	0	2.765681	2.566672	2.939179
80	6	0	1.488581	0.435781	4.218218
81	1	0	0.693336	-0.053924	2.278066
82	6	0	2.937979	2.369920	4.309063
83	1	0	3.270083	3.394281	2.448311
84	6	0	2.300116	1.302826	4.951197
85	1	0	0.998962	-0.403210	4.703681
86	1	0	3.572587	3.046772	4.875219
87	1	0	2.440526	1.147818	6.017795
88	46	0	0.436985	0.081577	-0.418453
89	6	0	-5.921246	-2.036785	0.553664
90	6	0	-5.127030	-2.534325	1.584919
91	6	0	-5.622548	-2.697070	2.870700
92	6	0	-6.961398	-2.341618	3.099932
93	6	0	-7.760979	-1.843206	2.061983
94	6	0	-7.246192	-1.686748	0.764788
95	6	0	-5.051342	-1.999683	-0.681797
96	6	0	-3.764083	-2.787292	0.989803
97	1	0	-4.995162	-3.082898	3.669861
98	1	0	-7.386162	-2.452407	4.094477
99	1	0	-8.794056	-1.573953	2.267621
100	1	0	-7.859531	-1.298500	-0.043854
101	7	0	-3.800226	-2.488905	-0.350501
102	8	0	-5.419414	-1.629084	-1.794708

103	8	0	-2.777152	-3.179253	1.623934
104	1	0	-1.813454	3.389552	-0.597224
105	1	0	-1.353032	2.870955	-2.226877
106	6	0	-3.478065	3.374452	-1.972107
107	1	0	-3.649131	3.142981	-3.032502
108	1	0	-3.534475	4.458191	-1.849540
109	8	0	-4.536223	0.779007	0.189639
110	7	0	-4.461650	2.693827	-1.119912
111	6	0	-5.933114	2.947192	-1.200342
112	6	0	-6.181953	4.316742	-1.849641
113	6	0	-6.570235	1.837982	-2.058438
114	6	0	-6.539301	2.962408	0.215091
115	1	0	-5.736631	5.127784	-1.262413
116	1	0	-5.793870	4.369839	-2.872105
117	1	0	-7.261947	4.491430	-1.897181
118	1	0	-6.357298	0.846629	-1.647116
119	1	0	-7.657374	1.972119	-2.107272
120	1	0	-6.178423	1.872838	-3.082688
121	1	0	-7.600680	3.230171	0.154229
122	1	0	-6.447765	1.988558	0.697174
123	1	0	-6.032936	3.709155	0.838457

INT-4-2ai-1

Charge = 1 Multiplicity = 1

Center Number	Atomic Number	Atomic Type	Coordinates (Angstroms) X	Y	Z
1	6	0	1.007737	2.601733	1.875465
2	6	0	0.381363	3.321539	0.816429
3	6	0	1.202616	3.801972	-0.297491
4	6	0	2.452338	3.345602	-0.495166
5	6	0	3.007619	2.200261	0.313492

6	6	0	2.110704	1.822844	1.479669
7	1	0	-0.552224	3.840282	1.014355
8	1	0	3.080427	3.746674	-1.286421
9	1	0	2.555608	1.157793	2.212953
10	6	0	3.186045	1.052374	-0.734674
11	8	0	2.251905	0.395660	-1.197831
12	7	0	4.493609	0.965088	-1.085816
13	6	0	4.953998	0.209114	-2.297279
14	6	0	4.530704	-1.267156	-2.188740
15	6	0	4.335816	0.865312	-3.544741
16	6	0	6.486108	0.269790	-2.383145
17	1	0	4.960858	-1.727669	-1.291982
18	1	0	3.446432	-1.374846	-2.152301
19	1	0	4.905513	-1.813933	-3.061228
20	1	0	4.638238	1.916794	-3.617494
21	1	0	4.673427	0.349839	-4.450755
22	1	0	3.243713	0.816829	-3.507689
23	1	0	6.808528	-0.294849	-3.263813
24	1	0	6.856625	1.293849	-2.493997
25	1	0	6.957866	-0.182286	-1.503585
26	6	0	5.311704	2.009939	-0.450576
27	6	0	4.473123	2.438333	0.759268
28	1	0	5.480172	2.839249	-1.149552
29	1	0	6.283209	1.616584	-0.147737
30	1	0	4.700686	1.786430	1.608759
31	1	0	4.660362	3.473155	1.055440
32	6	0	-3.274540	0.626763	-2.919091
33	6	0	-2.539628	0.125842	-1.832313
34	6	0	-2.105950	-1.201758	-1.905686
35	6	0	-2.314452	-2.028009	-3.015821
36	6	0	-3.024055	-1.482483	-4.089499
37	6	0	-3.511116	-0.173482	-4.035460
38	6	0	-1.822000	-3.478178	-2.934001
39	6	0	-0.524484	-3.484447	-2.117649

40	6	0	-0.427918	-2.591004	-1.049005
41	6	0	0.650854	-2.545726	-0.160499
42	6	0	1.678359	-3.481305	-0.342714
43	6	0	1.623656	-4.378531	-1.410701
44	6	0	0.539045	-4.376997	-2.292108
45	1	0	-3.647022	1.644568	-2.900749
46	1	0	-3.213800	-2.080671	-4.973992
47	1	0	-4.075349	0.226280	-4.872925
48	1	0	2.520114	-3.511530	0.338990
49	1	0	2.429116	-5.093406	-1.551451
50	1	0	0.521141	-5.092181	-3.107338
51	8	0	-1.446979	-1.694238	-0.801410
52	6	0	-1.636614	-4.103276	-4.324320
53	1	0	-0.894148	-3.558661	-4.917415
54	1	0	-1.317434	-5.146434	-4.241070
55	1	0	-2.582842	-4.109231	-4.873224
56	6	0	-2.880317	-4.299347	-2.144494
57	1	0	-2.544183	-5.336248	-2.030380
58	1	0	-3.043018	-3.879966	-1.145604
59	1	0	-3.836700	-4.297102	-2.679867
60	15	0	-2.077157	1.152978	-0.373407
61	15	0	0.562702	-1.253549	1.140394
62	6	0	-2.494720	2.837919	-0.988476
63	6	0	-1.605618	3.412989	-1.912734
64	6	0	-3.649159	3.542362	-0.622145
65	6	0	-1.862597	4.671404	-2.453773
66	1	0	-0.710025	2.873265	-2.208223
67	6	0	-3.897704	4.810821	-1.156966
68	1	0	-4.360451	3.110934	0.074279
69	6	0	-3.008295	5.377463	-2.071239
70	1	0	-1.167439	5.102135	-3.169374
71	1	0	-4.793697	5.349989	-0.861360
72	1	0	-3.206302	6.361552	-2.487107
73	6	0	-3.336966	0.780435	0.906867

74	6	0	-3.363477	1.584832	2.060172
75	6	0	-4.232198	-0.290833	0.797427
76	6	0	-4.292644	1.340185	3.069167
77	1	0	-2.663633	2.410259	2.162298
78	6	0	-5.146319	-0.547879	1.822994
79	1	0	-4.225419	-0.925522	-0.083090
80	6	0	-5.185327	0.269139	2.953600
81	1	0	-4.313440	1.977024	3.949449
82	1	0	-5.832491	-1.385195	1.731001
83	1	0	-5.901683	0.070077	3.745799
84	6	0	2.124500	-1.444791	2.099830
85	6	0	2.114843	-1.525554	3.500739
86	6	0	3.360432	-1.348597	1.436184
87	6	0	3.314668	-1.506729	4.218143
88	1	0	1.176190	-1.599249	4.040544
89	6	0	4.556025	-1.339716	2.154058
90	1	0	3.388866	-1.274543	0.357449
91	6	0	4.537308	-1.413232	3.550269
92	1	0	3.289302	-1.569031	5.302798
93	1	0	5.500010	-1.267031	1.620426
94	1	0	5.467402	-1.398771	4.111700
95	6	0	-0.734037	-1.888785	2.277978
96	6	0	-1.358948	-0.987782	3.152677
97	6	0	-1.093659	-3.244641	2.316164
98	6	0	-2.317276	-1.437073	4.062461
99	1	0	-1.102371	0.066287	3.114743
100	6	0	-2.064917	-3.687858	3.215356
101	1	0	-0.621017	-3.955177	1.644732
102	6	0	-2.675288	-2.786710	4.091862
103	1	0	-2.796743	-0.729247	4.731153
104	1	0	-2.341725	-4.738595	3.232258
105	1	0	-3.430378	-3.134539	4.791594
106	46	0	0.144222	1.066215	0.545286
107	1	0	0.785301	4.576075	-0.933843

108	6	0	0.453217	2.565652	3.275178
109	1	0	-0.636663	2.665565	3.278126
110	1	0	0.726991	1.639336	3.790112
111	1	0	0.866666	3.404311	3.851645

INT-4-2ai-2

Charge = 1 Multiplicity = 1

Center Number	Atomic Number	Atomic Type	Coordinates (Angstroms) X	Y	Z
1	6	0	-0.954329	2.354582	-2.205343
2	6	0	-0.456321	3.273177	-1.250994
3	6	0	-1.396095	3.898768	-0.307241
4	6	0	-2.631483	3.374342	-0.165307
5	6	0	-3.049235	2.066587	-0.785344
6	6	0	-2.055189	1.571441	-1.819783
7	1	0	-0.425858	2.164342	-3.135567
8	1	0	0.478781	3.787752	-1.448995
9	1	0	-3.367478	3.872121	0.462542
10	1	0	-2.416263	0.806555	-2.498790
11	6	0	-3.219024	1.120519	0.448774
12	8	0	-2.275206	0.611347	1.056950
13	7	0	-4.535136	1.029473	0.764723
14	6	0	-5.012825	0.499237	2.084631
15	6	0	-4.532877	-0.951365	2.273606
16	6	0	-4.462740	1.406029	3.199642
17	6	0	-6.548352	0.512925	2.115229
18	1	0	-4.940133	-1.600828	1.490360
19	1	0	-3.444734	-1.020169	2.256919
20	1	0	-4.889106	-1.326248	3.239783
21	1	0	-4.798262	2.440087	3.055868
22	1	0	-4.820731	1.063516	4.176896

23	1	0	-3.369361	1.390649	3.208889
24	1	0	-6.881283	0.102896	3.074113
25	1	0	-6.958031	1.524260	2.028076
26	1	0	-6.973457	-0.108951	1.319640
27	6	0	-5.376896	1.869778	-0.102491
28	6	0	-4.497529	2.111223	-1.334918
29	1	0	-5.634683	2.805703	0.409310
30	1	0	-6.304334	1.356950	-0.362048
31	1	0	-4.634496	1.292354	-2.047931
32	1	0	-4.728102	3.053510	-1.837639
33	6	0	3.348445	1.018228	2.697035
34	6	0	2.594554	0.357657	1.713873
35	6	0	2.110677	-0.916682	2.025377
36	6	0	2.291549	-1.539832	3.264558
37	6	0	3.020359	-0.835793	4.227670
38	6	0	3.554150	0.423633	3.941367
39	6	0	1.745067	-2.961803	3.437253
40	6	0	0.442220	-3.059143	2.635081
41	6	0	0.373736	-2.366814	1.424263
42	6	0	-0.704650	-2.443461	0.537570
43	6	0	-1.761402	-3.298053	0.880776
44	6	0	-1.739317	-3.984892	2.095497
45	6	0	-0.654956	-3.860553	2.968020
46	1	0	3.759762	2.001355	2.499980
47	1	0	3.187057	-1.271480	5.206733
48	1	0	4.131893	0.947393	4.697339
49	1	0	-2.602448	-3.428060	0.210972
50	1	0	-2.570912	-4.631751	2.359285
51	1	0	-0.660739	-4.413044	3.901097
52	8	0	1.424421	-1.568067	1.023999
53	6	0	1.546654	-3.331562	4.914244
54	1	0	0.834544	-2.660798	5.406718
55	1	0	1.180358	-4.357971	5.009595
56	1	0	2.497232	-3.286425	5.453964

57	6	0	2.766144	-3.947609	2.801399
58	1	0	2.395204	-4.976216	2.876614
59	1	0	2.933467	-3.720482	1.742803
60	1	0	3.727770	-3.882553	3.323082
61	15	0	2.141781	1.112881	0.092328
62	15	0	-0.577835	-1.408862	-0.975172
63	6	0	2.760361	2.830800	0.337316
64	6	0	1.974532	3.683903	1.127016
65	6	0	3.994336	3.290318	-0.144636
66	6	0	2.411561	4.972612	1.430252
67	1	0	1.022071	3.330026	1.510994
68	6	0	4.422905	4.588803	0.146418
69	1	0	4.628244	2.642704	-0.740561
70	6	0	3.635992	5.430798	0.934467
71	1	0	1.794363	5.620496	2.045962
72	1	0	5.378401	4.935923	-0.237552
73	1	0	3.973981	6.437731	1.163699
74	6	0	3.310302	0.399546	-1.130563
75	6	0	3.350171	0.983258	-2.408963
76	6	0	4.150737	-0.682506	-0.843084
77	6	0	4.246757	0.517206	-3.368401
78	1	0	2.691897	1.816222	-2.644042
79	6	0	5.028992	-1.164901	-1.817370
80	1	0	4.132351	-1.147961	0.137006
81	6	0	5.087808	-0.560804	-3.074104
82	1	0	4.282352	0.987230	-4.347514
83	1	0	5.671708	-2.010103	-1.587082
84	1	0	5.779393	-0.932626	-3.825129
85	6	0	-2.133212	-1.759332	-1.906979
86	6	0	-2.114178	-2.111589	-3.265445
87	6	0	-3.375903	-1.547236	-1.284284
88	6	0	-3.308703	-2.243314	-3.979823
89	1	0	-1.173750	-2.285716	-3.776610
90	6	0	-4.566182	-1.688263	-1.996832

91	1	0	-3.417141	-1.276200	-0.238977
92	6	0	-4.537391	-2.031902	-3.351362
93	1	0	-3.273653	-2.517651	-5.030749
94	1	0	-5.514046	-1.525281	-1.490692
95	1	0	-5.463423	-2.136698	-3.909880
96	6	0	0.715423	-2.255357	-1.969017
97	6	0	1.256593	-1.580397	-3.073977
98	6	0	1.143125	-3.561235	-1.686545
99	6	0	2.192126	-2.209552	-3.895661
100	1	0	0.946372	-0.562622	-3.293540
101	6	0	2.096299	-4.179073	-2.497866
102	1	0	0.736506	-4.099558	-0.836349
103	6	0	2.617796	-3.507920	-3.606196
104	1	0	2.603306	-1.677312	-4.747501
105	1	0	2.425859	-5.188085	-2.265061
106	1	0	3.357896	-3.991415	-4.238011
107	46	0	-0.132167	0.980057	-0.715098
108	6	0	-1.002462	5.178438	0.390508
109	1	0	-0.466066	4.981067	1.324357
110	1	0	-0.348174	5.795257	-0.235530
111	1	0	-1.890097	5.767462	0.643760

INT-5

Charge = 0 Multiplicity = 1

Center Number	Atomic Number	Atomic Type	Coordinates (Angstroms) X	Y	Z
1	6	0	2.093364	1.162372	0.408172
2	6	0	1.547005	2.181411	-0.389787
3	6	0	1.815801	2.194763	-1.829039
4	6	0	2.485944	1.212225	-2.454865
5	6	0	2.961630	-0.032157	-1.735950

6	6	0	3.071565	0.165182	-0.189580
7	1	0	2.258387	1.347050	1.468313
8	1	0	1.249309	3.123788	0.064596
9	1	0	1.479849	3.060089	-2.393687
10	1	0	2.681742	1.266181	-3.523913
11	1	0	2.936307	-0.810681	0.278097
12	6	0	1.947535	-1.156487	-2.079217
13	8	0	0.844075	-1.310726	-1.551381
14	7	0	2.453754	-1.926571	-3.083561
15	6	0	1.632991	-2.944789	-3.805607
16	6	0	1.135536	-4.005716	-2.804653
17	6	0	0.445482	-2.240356	-4.487861
18	6	0	2.494170	-3.641236	-4.870348
19	1	0	1.983251	-4.460487	-2.278243
20	1	0	0.460747	-3.570467	-2.066179
21	1	0	0.601299	-4.797895	-3.342273
22	1	0	0.801574	-1.494982	-5.209393
23	1	0	-0.169045	-2.970999	-5.026392
24	1	0	-0.183294	-1.738791	-3.748077
25	1	0	1.875366	-4.376554	-5.395131
26	1	0	2.878951	-2.938871	-5.617419
27	1	0	3.339617	-4.175699	-4.423069
28	6	0	3.749175	-1.426708	-3.560269
29	6	0	4.250013	-0.598157	-2.371982
30	1	0	3.623868	-0.818549	-4.466356
31	1	0	4.428549	-2.247821	-3.795645
32	1	0	4.745871	-1.269403	-1.664396
33	1	0	4.956988	0.180745	-2.669204
34	6	0	-4.275129	1.533436	-2.347567
35	6	0	-3.554256	1.008016	-1.262536
36	6	0	-3.891208	-0.285196	-0.843266
37	6	0	-4.845604	-1.086132	-1.482759
38	6	0	-5.515791	-0.536445	-2.579047
39	6	0	-5.243421	0.768613	-2.997301

40	6	0	-5.111655	-2.476456	-0.895212
41	6	0	-3.767813	-3.022454	-0.400106
42	6	0	-2.893879	-2.118515	0.212046
43	6	0	-1.681051	-2.482533	0.808986
44	6	0	-1.339961	-3.842468	0.775457
45	6	0	-2.171487	-4.768291	0.141684
46	6	0	-3.374666	-4.364296	-0.441941
47	1	0	-4.065983	2.537052	-2.700396
48	1	0	-6.261592	-1.120277	-3.107614
49	1	0	-5.784888	1.189179	-3.840259
50	1	0	-0.419012	-4.182707	1.234906
51	1	0	-1.884348	-5.815907	0.113882
52	1	0	-4.010563	-5.103869	-0.916813
53	8	0	-3.227126	-0.778334	0.260497
54	6	0	-5.784669	-3.414983	-1.906436
55	1	0	-5.163155	-3.564962	-2.795906
56	1	0	-5.982499	-4.390961	-1.452759
57	1	0	-6.751996	-3.012927	-2.223286
58	6	0	-6.046331	-2.313230	0.336868
59	1	0	-6.228662	-3.287793	0.804750
60	1	0	-5.603559	-1.651273	1.088384
61	1	0	-7.008519	-1.887994	0.028154
62	15	0	-2.121021	1.869959	-0.457737
63	15	0	-0.666258	-1.120150	1.547829
64	6	0	-1.909199	3.280167	-1.637182
65	6	0	-1.231774	3.004675	-2.837628
66	6	0	-2.398330	4.576095	-1.420002
67	6	0	-1.049702	3.999978	-3.797542
68	1	0	-0.839927	2.006901	-3.016228
69	6	0	-2.200506	5.578140	-2.375577
70	1	0	-2.938942	4.810659	-0.508573
71	6	0	-1.528416	5.293808	-3.565858
72	1	0	-0.525180	3.768365	-4.721110
73	1	0	-2.579692	6.579780	-2.189001

74	1	0	-1.378448	6.073175	-4.308454
75	6	0	-2.879629	2.669266	1.021804
76	6	0	-2.075681	3.541631	1.778057
77	6	0	-4.186592	2.409990	1.453644
78	6	0	-2.577847	4.157780	2.923291
79	1	0	-1.052589	3.736160	1.466237
80	6	0	-4.681030	3.009950	2.615645
81	1	0	-4.826453	1.739963	0.887719
82	6	0	-3.883624	3.889852	3.348741
83	1	0	-1.946739	4.837189	3.490530
84	1	0	-5.694289	2.790324	2.942174
85	1	0	-4.272123	4.359315	4.248703
86	6	0	0.737551	-2.063822	2.289197
87	6	0	0.973532	-2.124959	3.670879
88	6	0	1.642462	-2.699438	1.418628
89	6	0	2.084491	-2.811553	4.169898
90	1	0	0.291637	-1.641741	4.363413
91	6	0	2.740295	-3.397173	1.920809
92	1	0	1.478524	-2.644790	0.346545
93	6	0	2.966528	-3.454189	3.299085
94	1	0	2.254511	-2.847157	5.243245
95	1	0	3.431527	-3.874805	1.233822
96	1	0	3.829201	-3.987938	3.689306
97	6	0	-1.685202	-0.683084	3.026465
98	6	0	-1.561579	0.608510	3.557953
99	6	0	-2.567005	-1.588476	3.637893
100	6	0	-2.300065	0.988590	4.680613
101	1	0	-0.895815	1.320544	3.079151
102	6	0	-3.314335	-1.204656	4.752791
103	1	0	-2.673929	-2.594275	3.242151
104	6	0	-3.181603	0.084551	5.277276
105	1	0	-2.199200	1.995773	5.073380
106	1	0	-3.999072	-1.913018	5.212481
107	1	0	-3.765963	0.382950	6.143947

108	46	0	-0.097886	0.765863	0.165667
109	6	0	6.427039	1.657221	0.682520
110	6	0	6.588256	0.337497	1.101362
111	6	0	7.766917	-0.103754	1.685653
112	6	0	8.794910	0.836448	1.846114
113	6	0	8.632745	2.164185	1.426456
114	6	0	7.437772	2.595446	0.832099
115	6	0	5.066181	1.797759	0.084039
116	6	0	5.335512	-0.399742	0.782064
117	1	0	7.885138	-1.134427	2.005708
118	1	0	9.733038	0.532326	2.301505
119	1	0	9.447632	2.869320	1.563338
120	1	0	7.306295	3.621103	0.501308
121	7	0	4.455614	0.534640	0.208128
122	8	0	4.584249	2.791491	-0.423105
123	8	0	5.098866	-1.584163	0.946188

TS-2ai-1

Charge = 0 Multiplicity = 1

Center Number	Atomic Number	Atomic Type	Coordinates (Angstroms) X	Y	Z
1	6	0	-2.144744	-0.980624	-0.357703
2	6	0	-1.501082	-1.607445	-1.470989
3	6	0	-1.639875	-1.012292	-2.798291
4	6	0	-2.150089	0.219845	-2.986023
5	6	0	-2.527715	1.093324	-1.819635
6	6	0	-2.699795	0.301422	-0.547218
7	1	0	-1.312165	-2.677102	-1.429470
8	1	0	-1.343516	-1.614698	-3.651733
9	1	0	-2.251403	0.645107	-3.981593
10	1	0	-2.999957	0.872924	0.322889

11	6	0	-1.341936	2.130633	-1.704367
12	8	0	-0.201716	1.854755	-1.333642
13	7	0	-1.760564	3.344105	-2.145237
14	6	0	-0.810039	4.476382	-2.388406
15	6	0	-0.052626	4.803726	-1.087697
16	6	0	0.170122	4.075337	-3.505618
17	6	0	-1.601958	5.720282	-2.818812
18	1	0	-0.757744	5.045796	-0.283837
19	1	0	0.570535	3.970099	-0.766039
20	1	0	0.590901	5.675906	-1.250118
21	1	0	-0.372345	3.833666	-4.427471
22	1	0	0.856703	4.902073	-3.721251
23	1	0	0.761255	3.204359	-3.209965
24	1	0	-0.899820	6.546587	-2.970643
25	1	0	-2.139851	5.565982	-3.759979
26	1	0	-2.319819	6.026731	-2.049837
27	6	0	-3.101582	3.285712	-2.740975
28	6	0	-3.718210	2.038492	-2.105028
29	1	0	-3.026791	3.205110	-3.834108
30	1	0	-3.676041	4.183012	-2.505970
31	1	0	-4.198938	2.296173	-1.158477
32	1	0	-4.465649	1.564781	-2.742203
33	6	0	4.370150	-1.242215	-2.451915
34	6	0	3.591777	-0.937416	-1.323661
35	6	0	3.984728	0.167338	-0.556159
36	6	0	5.060990	0.999953	-0.887441
37	6	0	5.788973	0.679056	-2.037158
38	6	0	5.455073	-0.440228	-2.803512
39	6	0	5.399311	2.147878	0.072025
40	6	0	4.078946	2.681363	0.638922
41	6	0	3.070852	1.752290	0.908013
42	6	0	1.850508	2.076778	1.512400
43	6	0	1.649972	3.413923	1.880332
44	6	0	2.626830	4.373432	1.606806

45	6	0	3.826956	4.013021	0.988438
46	1	0	4.117666	-2.097441	-3.068630
47	1	0	6.630661	1.293678	-2.337709
48	1	0	6.042021	-0.685345	-3.684245
49	1	0	0.727067	3.711891	2.365255
50	1	0	2.454187	5.409534	1.884136
51	1	0	4.572683	4.776226	0.794312
52	8	0	3.258968	0.422005	0.587499
53	6	0	6.224388	3.249332	-0.608479
54	1	0	5.688160	3.693789	-1.453807
55	1	0	6.466652	4.043048	0.104596
56	1	0	7.177287	2.852266	-0.971103
57	6	0	6.222376	1.560391	1.252755
58	1	0	6.463227	2.348774	1.975299
59	1	0	5.665007	0.775757	1.775562
60	1	0	7.158580	1.127446	0.881743
61	15	0	2.036056	-1.842974	-0.888189
62	15	0	0.644360	0.699652	1.703021
63	6	0	1.841537	-2.920534	-2.375229
64	6	0	1.333595	-2.313896	-3.536598
65	6	0	2.189209	-4.278016	-2.415803
66	6	0	1.172620	-3.048339	-4.710742
67	1	0	1.062250	-1.262250	-3.517378
68	6	0	2.013061	-5.017264	-3.589908
69	1	0	2.599920	-4.765302	-1.537558
70	6	0	1.505396	-4.406741	-4.738117
71	1	0	0.779529	-2.563229	-5.600425
72	1	0	2.279847	-6.070877	-3.604635
73	1	0	1.370031	-4.983279	-5.649338
74	6	0	2.541031	-3.016271	0.436831
75	6	0	1.593777	-3.961931	0.868758
76	6	0	3.800506	-2.987001	1.048604
77	6	0	1.913271	-4.877909	1.869335
78	1	0	0.605878	-3.982748	0.415435

79	6	0	4.109039	-3.888609	2.071230
80	1	0	4.547232	-2.266044	0.730864
81	6	0	3.172331	-4.839731	2.478118
82	1	0	1.175502	-5.611063	2.184711
83	1	0	5.086626	-3.848348	2.544359
84	1	0	3.417067	-5.543222	3.269400
85	6	0	-0.823780	1.509380	2.473705
86	6	0	-1.356306	1.089640	3.701533
87	6	0	-1.504628	2.503756	1.746529
88	6	0	-2.545090	1.646299	4.185041
89	1	0	-0.852877	0.325169	4.284299
90	6	0	-2.681999	3.065145	2.236914
91	1	0	-1.113035	2.831642	0.790906
92	6	0	-3.210625	2.633087	3.457238
93	1	0	-2.947754	1.303384	5.134777
94	1	0	-3.196333	3.825449	1.655999
95	1	0	-4.138887	3.056536	3.830487
96	6	0	1.363327	-0.272131	3.092055
97	6	0	0.926194	-1.594037	3.264347
98	6	0	2.303028	0.258467	3.988899
99	6	0	1.407682	-2.367400	4.321820
100	1	0	0.211659	-2.018206	2.565128
101	6	0	2.797598	-0.522503	5.035273
102	1	0	2.651915	1.280249	3.871649
103	6	0	2.348596	-1.834841	5.206302
104	1	0	1.062717	-3.390116	4.440222
105	1	0	3.531038	-0.103557	5.719526
106	1	0	2.733890	-2.440997	6.022043
107	46	0	0.123607	-0.602119	-0.286115
108	6	0	-6.556031	-1.727523	-0.102887
109	6	0	-6.744027	-0.579357	0.664714
110	6	0	-7.796187	-0.469186	1.561898
111	6	0	-8.674243	-1.560037	1.668080
112	6	0	-8.486861	-2.713607	0.894660

113	6	0	-7.416362	-2.811386	-0.009135
114	6	0	-5.329291	-1.485199	-0.954971
115	6	0	-5.629138	0.368484	0.296193
116	1	0	-7.934795	0.428338	2.158542
117	1	0	-9.511436	-1.511736	2.359748
118	1	0	-9.182134	-3.542894	0.997341
119	1	0	-7.264411	-3.702758	-0.611690
120	7	0	-4.859977	-0.215018	-0.685127
121	8	0	-4.849495	-2.295922	-1.744945
122	8	0	-5.444433	1.479211	0.800057
123	6	0	-2.495894	-1.754582	0.893785
124	1	0	-3.508670	-2.170791	0.810535
125	1	0	-1.805881	-2.588934	1.053684
126	1	0	-2.474190	-1.112739	1.780914

TS-2ai-2

Charge = 0 Multiplicity = 1

Center Number	Atomic Number	Atomic Type	Coordinates (Angstroms) X	Y	Z
1	6	0	-2.090680	-0.208799	1.089321
2	6	0	-2.716127	-0.947114	0.036632
3	6	0	-3.043078	-0.280988	-1.222800
4	6	0	-2.868705	1.039739	-1.382357
5	6	0	-2.203027	1.908878	-0.343118
6	6	0	-1.690735	1.120342	0.848027
7	1	0	-2.665607	-2.026571	0.045221
8	1	0	-3.506429	-0.884570	-1.996855
9	1	0	-3.180762	1.526673	-2.304143
10	1	0	-1.412923	1.728720	1.704340
11	6	0	-1.109893	2.689484	-1.132632
12	8	0	-0.041015	2.206072	-1.506300

13	7	0	-1.562551	3.947526	-1.402834
14	6	0	-0.911487	4.835424	-2.416978
15	6	0	0.553665	5.097946	-2.023106
16	6	0	-0.991200	4.152262	-3.794287
17	6	0	-1.645875	6.184315	-2.466162
18	1	0	0.606997	5.592161	-1.046198
19	1	0	1.129597	4.173037	-1.980928
20	1	0	1.014009	5.763203	-2.762692
21	1	0	-2.035488	3.960644	-4.069229
22	1	0	-0.548192	4.794380	-4.564087
23	1	0	-0.451756	3.201217	-3.785397
24	1	0	-1.145451	6.827873	-3.197248
25	1	0	-2.690329	6.080467	-2.777703
26	1	0	-1.616689	6.694446	-1.496722
27	6	0	-2.951859	4.143381	-0.959528
28	6	0	-3.138468	3.064303	0.109470
29	1	0	-3.644882	4.013295	-1.801873
30	1	0	-3.096202	5.145612	-0.552603
31	1	0	-2.782032	3.443337	1.073177
32	1	0	-4.171961	2.738765	0.233045
33	6	0	2.410598	-3.003434	-2.872587
34	6	0	2.371440	-2.178008	-1.737536
35	6	0	3.385205	-1.219013	-1.608801
36	6	0	4.392037	-1.022022	-2.562994
37	6	0	4.377139	-1.847020	-3.691353
38	6	0	3.401106	-2.835928	-3.839245
39	6	0	5.463850	0.030970	-2.257045
40	6	0	4.770996	1.194162	-1.538865
41	6	0	3.765232	0.873704	-0.623562
42	6	0	3.116119	1.815494	0.185073
43	6	0	3.518816	3.152512	0.063482
44	6	0	4.499943	3.511950	-0.862745
45	6	0	5.118125	2.544416	-1.659192
46	1	0	1.653625	-3.767725	-3.009196

47	1	0	5.133219	-1.730719	-4.460459
48	1	0	3.411892	-3.477380	-4.715972
49	1	0	3.062410	3.916495	0.682673
50	1	0	4.792927	4.553936	-0.956649
51	1	0	5.884811	2.848175	-2.363686
52	8	0	3.374090	-0.442542	-0.468608
53	6	0	6.211512	0.486805	-3.517798
54	1	0	5.533455	0.933105	-4.253169
55	1	0	6.980259	1.222548	-3.262593
56	1	0	6.727755	-0.356004	-3.987711
57	6	0	6.485473	-0.595037	-1.265655
58	1	0	7.250412	0.142982	-0.997810
59	1	0	5.993964	-0.927689	-0.345312
60	1	0	6.978906	-1.459554	-1.724806
61	15	0	1.005996	-2.239968	-0.488865
62	15	0	1.773918	1.167432	1.264242
63	6	0	-0.194542	-3.357235	-1.339980
64	6	0	-1.042129	-2.768937	-2.294136
65	6	0	-0.294163	-4.735501	-1.102181
66	6	0	-1.971596	-3.539785	-2.990801
67	1	0	-0.979525	-1.700628	-2.482823
68	6	0	-1.238616	-5.504067	-1.789815
69	1	0	0.359395	-5.215334	-0.380713
70	6	0	-2.078583	-4.910143	-2.733427
71	1	0	-2.621936	-3.069071	-3.723332
72	1	0	-1.313102	-6.569333	-1.586831
73	1	0	-2.814489	-5.508876	-3.263328
74	6	0	1.682705	-3.283762	0.868397
75	6	0	0.792958	-3.703794	1.873374
76	6	0	3.036353	-3.627393	0.970884
77	6	0	1.245135	-4.476387	2.941512
78	1	0	-0.258406	-3.433649	1.811046
79	6	0	3.491782	-4.382899	2.055471
80	1	0	3.740889	-3.312151	0.207464

81	6	0	2.599363	-4.815423	3.037346
82	1	0	0.543484	-4.805679	3.703480
83	1	0	4.546110	-4.637183	2.125929
84	1	0	2.955198	-5.408444	3.875654
85	6	0	1.168917	2.643794	2.190935
86	6	0	1.095381	2.662404	3.592658
87	6	0	0.606745	3.716334	1.475075
88	6	0	0.477595	3.726354	4.256932
89	1	0	1.514199	1.847024	4.173851
90	6	0	-0.001002	4.780970	2.139529
91	1	0	0.643780	3.715781	0.394175
92	6	0	-0.072291	4.788429	3.536122
93	1	0	0.429806	3.722271	5.342912
94	1	0	-0.428290	5.597349	1.562534
95	1	0	-0.553963	5.612053	4.056171
96	6	0	2.718040	0.235069	2.545358
97	6	0	2.055466	-0.774329	3.259413
98	6	0	4.061914	0.510908	2.840942
99	6	0	2.720822	-1.487075	4.258571
100	1	0	1.021898	-1.010158	3.023819
101	6	0	4.730935	-0.214758	3.828116
102	1	0	4.589452	1.289762	2.298058
103	6	0	4.060852	-1.212957	4.540939
104	1	0	2.197452	-2.269847	4.798944
105	1	0	5.774352	0.002370	4.042242
106	1	0	4.582388	-1.777867	5.309247
107	46	0	0.045265	-0.193788	0.280861
108	6	0	-6.885534	-0.321645	0.607694
109	6	0	-6.902322	-1.539612	-0.071633
110	6	0	-8.069015	-2.045398	-0.626349
111	6	0	-9.239525	-1.284231	-0.478446
112	6	0	-9.222532	-0.060295	0.204563
113	6	0	-8.034938	0.439476	0.762060
114	6	0	-5.468826	-0.106974	1.079985

115	6	0	-5.490596	-2.072089	-0.039408
116	1	0	-8.075150	-2.994872	-1.154328
117	1	0	-10.174072	-1.647426	-0.898014
118	1	0	-10.144186	0.507171	0.304278
119	1	0	-8.014181	1.386111	1.294905
120	7	0	-4.717927	-1.201136	0.705367
121	8	0	-5.064260	0.876214	1.700761
122	8	0	-5.094610	-3.094236	-0.595570
123	6	0	-2.088335	-0.785833	2.481889
124	1	0	-3.091613	-0.652529	2.909122
125	1	0	-1.864482	-1.858033	2.480470
126	1	0	-1.364968	-0.277825	3.127931

TS-2ai-3

Charge = 0 Multiplicity = 1

Center Number	Atomic Number	Atomic Type	Coordinates (Angstroms) X	Y	Z
1	6	0	2.040964	0.118991	1.078784
2	6	0	1.369295	0.890775	2.075102
3	6	0	1.535951	2.345886	2.090801
4	6	0	2.066699	2.973109	1.019379
5	6	0	2.497300	2.239215	-0.225394
6	6	0	2.648791	0.762199	-0.007168
7	1	0	1.150529	0.416205	3.027538
8	1	0	2.204102	4.052099	1.021297
9	1	0	2.987898	0.183504	-0.855290
10	6	0	1.385505	2.533260	-1.312199
11	8	0	0.272156	2.009500	-1.346844
12	7	0	1.834281	3.505981	-2.144853
13	6	0	0.936435	4.189560	-3.128267
14	6	0	0.388783	3.149560	-4.123022

15	6	0	-0.210037	4.881736	-2.367371
16	6	0	1.737017	5.241161	-3.911876
17	1	0	1.214601	2.642536	-4.635869
18	1	0	-0.223321	2.400777	-3.618074
19	1	0	-0.226518	3.651159	-4.878869
20	1	0	0.186711	5.612745	-1.652448
21	1	0	-0.862205	5.411468	-3.071024
22	1	0	-0.812766	4.151538	-1.821700
23	1	0	1.065635	5.727397	-4.627146
24	1	0	2.149024	6.019300	-3.260967
25	1	0	2.556589	4.787140	-4.479158
26	6	0	3.151383	4.022015	-1.750768
27	6	0	3.728154	2.893286	-0.895270
28	1	0	3.040756	4.958988	-1.188508
29	1	0	3.772710	4.222568	-2.625184
30	1	0	4.231565	2.153273	-1.521606
31	1	0	4.446473	3.244051	-0.153743
32	6	0	-4.801106	1.654875	0.971804
33	6	0	-3.824927	0.688719	0.674193
34	6	0	-4.086312	-0.174247	-0.397940
35	6	0	-5.237719	-0.091888	-1.194468
36	6	0	-6.168753	0.901191	-0.879908
37	6	0	-5.957006	1.760412	0.201295
38	6	0	-5.409076	-1.131325	-2.309456
39	6	0	-4.019551	-1.393107	-2.899074
40	6	0	-2.939955	-1.415382	-2.011983
41	6	0	-1.623870	-1.695553	-2.395551
42	6	0	-1.394997	-1.996592	-3.745905
43	6	0	-2.449399	-1.976099	-4.660068
44	6	0	-3.747974	-1.670785	-4.243092
45	1	0	-4.648955	2.339077	1.798993
46	1	0	-7.071625	1.007265	-1.471291
47	1	0	-6.696279	2.519570	0.440523
48	1	0	-0.391993	-2.230890	-4.085907

49	1	0	-2.259067	-2.201906	-5.705639
50	1	0	-4.550279	-1.660162	-4.973203
51	8	0	-3.150249	-1.149954	-0.671116
52	6	0	-6.414988	-0.678737	-3.377050
53	1	0	-6.103211	0.255396	-3.856399
54	1	0	-6.526973	-1.444698	-4.150430
55	1	0	-7.406087	-0.530304	-2.937971
56	6	0	-5.917180	-2.453240	-1.668489
57	1	0	-6.007728	-3.233239	-2.433229
58	1	0	-5.229825	-2.811696	-0.895221
59	1	0	-6.900441	-2.298599	-1.209102
60	15	0	-2.233713	0.575114	1.616769
61	15	0	-0.343226	-1.619074	-1.073460
62	6	0	-2.204768	2.237006	2.406703
63	6	0	-1.719995	3.291163	1.612018
64	6	0	-2.644516	2.503331	3.710233
65	6	0	-1.694941	4.592890	2.109787
66	1	0	-1.348340	3.083918	0.611129
67	6	0	-2.598442	3.807911	4.212550
68	1	0	-3.024627	1.699135	4.333206
69	6	0	-2.130646	4.853631	3.413253
70	1	0	-1.319202	5.400101	1.486435
71	1	0	-2.934469	4.005566	5.227138
72	1	0	-2.099268	5.866481	3.805844
73	6	0	-2.613451	-0.625230	2.959614
74	6	0	-1.649213	-0.820958	3.964630
75	6	0	-3.760825	-1.430471	2.953117
76	6	0	-1.835410	-1.793769	4.945579
77	1	0	-0.749708	-0.212845	3.975841
78	6	0	-3.936813	-2.417389	3.927549
79	1	0	-4.518753	-1.297118	2.187412
80	6	0	-2.978016	-2.601699	4.925022
81	1	0	-1.084426	-1.928254	5.719592
82	1	0	-4.827503	-3.039703	3.904630

83	1	0	-3.116908	-3.368867	5.681997
84	6	0	1.237358	-1.760860	-2.000025
85	6	0	2.218720	-2.706620	-1.671284
86	6	0	1.545451	-0.755746	-2.936648
87	6	0	3.482237	-2.649858	-2.268427
88	1	0	2.007635	-3.482392	-0.941516
89	6	0	2.800239	-0.713746	-3.541554
90	1	0	0.811439	0.010944	-3.166545
91	6	0	3.776426	-1.656595	-3.202779
92	1	0	4.240398	-3.376676	-1.988954
93	1	0	3.026517	0.072954	-4.256472
94	1	0	4.766654	-1.597652	-3.641958
95	6	0	-0.576093	-3.256670	-0.254466
96	6	0	-0.685333	-3.308694	1.142587
97	6	0	-0.687365	-4.445220	-0.995055
98	6	0	-0.904058	-4.526452	1.791700
99	1	0	-0.615740	-2.390228	1.718909
100	6	0	-0.897645	-5.661933	-0.345617
101	1	0	-0.614321	-4.418436	-2.079127
102	6	0	-1.008766	-5.703969	1.048835
103	1	0	-1.000781	-4.547201	2.873998
104	1	0	-0.980916	-6.576933	-0.926421
105	1	0	-1.180530	-6.652295	1.551542
106	46	0	-0.161870	0.123387	0.588472
107	6	0	6.005379	-1.188698	1.633977
108	6	0	6.347910	-1.188347	0.283453
109	6	0	7.210576	-2.135810	-0.248899
110	6	0	7.730775	-3.102460	0.626640
111	6	0	7.388877	-3.101561	1.986057
112	6	0	6.515400	-2.135326	2.510814
113	6	0	5.045530	-0.037560	1.833834
114	6	0	5.596419	-0.042379	-0.347790
115	1	0	7.469680	-2.130359	-1.304131
116	1	0	8.408060	-3.863648	0.247800

117	1	0	7.806129	-3.862424	2.640837
118	1	0	6.244691	-2.130454	3.563191
119	7	0	4.859550	0.604127	0.623891
120	8	0	4.516520	0.256122	2.905097
121	8	0	5.632755	0.243622	-1.547071
122	1	0	2.249199	-0.934141	1.240988
123	6	0	1.186220	3.067510	3.365006
124	1	0	0.174827	2.816368	3.700585
125	1	0	1.876534	2.767981	4.165971
126	1	0	1.250344	4.153639	3.249808

TS-2ai-4

Charge = 0 Multiplicity = 1

Center Number	Atomic Number	Atomic Type	Coordinates (Angstroms) X	Y	Z
1	6	0	-2.356125	0.244917	0.163147
2	6	0	-2.848734	-0.150205	-1.102452
3	6	0	-2.725906	0.730885	-2.263145
4	6	0	-2.215596	1.964150	-2.093053
5	6	0	-1.651752	2.485266	-0.793922
6	6	0	-1.645086	1.453856	0.320261
7	1	0	-3.115969	-1.185056	-1.268918
8	1	0	-2.181267	2.650915	-2.937522
9	1	0	-1.520847	1.859082	1.320060
10	6	0	-0.252925	3.036077	-1.199051
11	8	0	0.739453	2.336168	-1.402813
12	7	0	-0.336314	4.386883	-1.365196
13	6	0	0.728749	5.178694	-2.057924
14	6	0	2.067715	5.020767	-1.313556
15	6	0	0.850403	4.678772	-3.508892
16	6	0	0.347154	6.666836	-2.055358

17	1	0	1.986718	5.399902	-0.288315
18	1	0	2.384807	3.978413	-1.278078
19	1	0	2.839918	5.604086	-1.828318
20	1	0	-0.102950	4.796373	-4.038144
21	1	0	1.612457	5.253976	-4.047069
22	1	0	1.135082	3.623481	-3.531340
23	1	0	1.146692	7.233380	-2.544140
24	1	0	-0.579960	6.861365	-2.604171
25	1	0	0.239206	7.053398	-1.035645
26	6	0	-1.710989	4.884623	-1.189629
27	6	0	-2.383573	3.785763	-0.362555
28	1	0	-2.196401	5.021909	-2.165032
29	1	0	-1.716843	5.844449	-0.670257
30	1	0	-2.186481	3.960652	0.700436
31	1	0	-3.462221	3.713544	-0.506274
32	6	0	2.341507	-3.014566	-2.881578
33	6	0	2.226841	-2.324458	-1.664894
34	6	0	3.311017	-1.525881	-1.277366
35	6	0	4.470985	-1.363718	-2.043191
36	6	0	4.533328	-2.049121	-3.260591
37	6	0	3.482505	-2.872835	-3.671030
38	6	0	5.608640	-0.519043	-1.455715
39	6	0	4.977217	0.625419	-0.654804
40	6	0	3.791202	0.360810	0.034692
41	6	0	3.140530	1.291723	0.854752
42	6	0	3.755148	2.539741	1.023668
43	6	0	4.936485	2.840936	0.343763
44	6	0	5.535344	1.898997	-0.496153
45	1	0	1.531805	-3.649866	-3.222742
46	1	0	5.409158	-1.953052	-3.892623
47	1	0	3.553383	-3.407160	-4.614230
48	1	0	3.309239	3.283170	1.673998
49	1	0	5.396159	3.816825	0.472449
50	1	0	6.452646	2.158753	-1.013424

51	8	0	3.201413	-0.884231	-0.061098
52	6	0	6.565004	-0.004489	-2.541697
53	1	0	6.047237	0.631832	-3.267469
54	1	0	7.382013	0.570828	-2.096166
55	1	0	7.026607	-0.839232	-3.077463
56	6	0	6.404739	-1.414106	-0.464871
57	1	0	7.213210	-0.838389	0.000446
58	1	0	5.757578	-1.799146	0.330610
59	1	0	6.843182	-2.268081	-0.994132
60	15	0	0.686123	-2.307345	-0.641028
61	15	0	1.517530	0.776510	1.556175
62	6	0	-0.447488	-3.314692	-1.697072
63	6	0	-1.184604	-2.638305	-2.680166
64	6	0	-0.574188	-4.708407	-1.592322
65	6	0	-2.047984	-3.332970	-3.527788
66	1	0	-1.079173	-1.562080	-2.774719
67	6	0	-1.444151	-5.403155	-2.436495
68	1	0	-0.000492	-5.255800	-0.851568
69	6	0	-2.185411	-4.717885	-3.401909
70	1	0	-2.621552	-2.792764	-4.275399
71	1	0	-1.541109	-6.481253	-2.337705
72	1	0	-2.867853	-5.259006	-4.051834
73	6	0	1.035804	-3.442320	0.766418
74	6	0	-0.056518	-3.885883	1.533168
75	6	0	2.327125	-3.851385	1.121581
76	6	0	0.138914	-4.752343	2.607221
77	1	0	-1.063662	-3.565621	1.277164
78	6	0	2.522394	-4.700182	2.214603
79	1	0	3.185180	-3.518395	0.547439
80	6	0	1.431103	-5.161417	2.952143
81	1	0	-0.716123	-5.100026	3.181129
82	1	0	3.530702	-5.003782	2.483295
83	1	0	1.585200	-5.827736	3.796659
84	6	0	0.960663	2.249769	2.524050

85	6	0	0.612084	2.172360	3.881542
86	6	0	0.753962	3.465748	1.849170
87	6	0	0.071696	3.281137	4.540257
88	1	0	0.757116	1.250522	4.434743
89	6	0	0.225452	4.574167	2.509476
90	1	0	1.016368	3.547314	0.804223
91	6	0	-0.123731	4.484811	3.860306
92	1	0	-0.192749	3.200139	5.591488
93	1	0	0.081005	5.503148	1.963667
94	1	0	-0.544736	5.343591	4.376134
95	6	0	2.000309	-0.407784	2.881263
96	6	0	1.004450	-1.231955	3.428041
97	6	0	3.304030	-0.480114	3.393816
98	6	0	1.301802	-2.094621	4.483649
99	1	0	-0.003958	-1.197951	3.024263
100	6	0	3.604945	-1.362301	4.433373
101	1	0	4.086729	0.151278	2.984265
102	6	0	2.604156	-2.166502	4.984125
103	1	0	0.522069	-2.726942	4.896824
104	1	0	4.620896	-1.415849	4.816216
105	1	0	2.838817	-2.850519	5.795443
106	46	0	-0.128303	-0.145957	0.040021
107	6	0	-6.724427	0.211330	0.758658
108	6	0	-6.799831	-1.070027	0.213172
109	6	0	-7.748579	-1.986926	0.642253
110	6	0	-8.636877	-1.577421	1.649856
111	6	0	-8.561507	-0.289574	2.197508
112	6	0	-7.595528	0.628377	1.754226
113	6	0	-5.583671	0.914419	0.057723
114	6	0	-5.700791	-1.159857	-0.818445
115	1	0	-7.800242	-2.984744	0.215280
116	1	0	-9.394251	-2.267610	2.012707
117	1	0	-9.261305	0.000037	2.977256
118	1	0	-7.530353	1.627905	2.175442

119	7	0	-5.059252	0.057596	-0.886237
120	8	0	-5.201294	2.057647	0.308451
121	8	0	-5.413699	-2.162784	-1.474765
122	1	0	-2.628825	-0.351420	1.030061
123	6	0	-3.262516	0.241534	-3.582972
124	1	0	-2.552608	-0.433902	-4.076408
125	1	0	-4.193849	-0.313120	-3.427369
126	1	0	-3.455759	1.074434	-4.267276

TS-2ai-5

Charge = 0 Multiplicity = 1

Center Number	Atomic Number	Atomic Type	Coordinates (Angstroms) X	Y	Z
1	6	0	1.777881	1.016069	1.414023
2	6	0	2.192551	1.728586	0.245401
3	6	0	2.927430	1.128244	-0.774551
4	6	0	3.351507	-0.199597	-0.632395
5	6	0	2.561842	-1.129481	0.281720
6	6	0	1.755618	-0.386116	1.336449
7	1	0	2.005504	2.798254	0.212482
8	1	0	3.288361	1.725971	-1.605669
9	1	0	3.830484	-0.685696	-1.475191
10	1	0	1.541149	-0.953175	2.238498
11	6	0	1.722525	-1.951183	-0.769832
12	8	0	0.693918	-1.555701	-1.316739
13	7	0	2.362055	-3.128026	-1.009727
14	6	0	2.013905	-4.010583	-2.166385
15	6	0	0.552141	-4.478015	-2.035271
16	6	0	2.226439	-3.224740	-3.473161
17	6	0	2.925125	-5.246752	-2.159914

18	1	0	0.413904	-5.043616	-1.106553
19	1	0	-0.137932	-3.633849	-2.035508
20	1	0	0.300565	-5.137294	-2.874247
21	1	0	3.268099	-2.892483	-3.556766
22	1	0	2.000008	-3.859326	-4.337720
23	1	0	1.575857	-2.347018	-3.510090
24	1	0	2.658771	-5.881693	-3.011398
25	1	0	3.982630	-4.982074	-2.260421
26	1	0	2.795224	-5.839503	-1.247794
27	6	0	3.617694	-3.245977	-0.247299
28	6	0	3.404120	-2.264729	0.903126
29	1	0	4.478043	-2.959655	-0.864984
30	1	0	3.763043	-4.266953	0.109805
31	1	0	2.810947	-2.745693	1.687117
32	1	0	4.336939	-1.892999	1.322707
33	6	0	-2.753792	2.630869	-3.037069
34	6	0	-2.666308	1.820309	-1.893525
35	6	0	-3.416972	0.638152	-1.892262
36	6	0	-4.194021	0.208667	-2.975508
37	6	0	-4.226064	1.028630	-4.106786
38	6	0	-3.523021	2.235978	-4.130839
39	6	0	-4.991737	-1.089591	-2.804894
40	6	0	-4.130610	-2.048387	-1.974811
41	6	0	-3.388279	-1.495420	-0.928271
42	6	0	-2.647124	-2.249101	-0.010700
43	6	0	-2.662904	-3.642391	-0.165387
44	6	0	-3.364833	-4.226722	-1.221545
45	6	0	-4.090540	-3.439357	-2.119654
46	1	0	-2.204909	3.564769	-3.081275
47	1	0	-4.808737	0.735912	-4.973599
48	1	0	-3.571540	2.870660	-5.011167
49	1	0	-2.120566	-4.273822	0.529113
50	1	0	-3.355815	-5.306787	-1.338534
51	1	0	-4.637947	-3.919488	-2.923606

52	8	0	-3.378005	-0.126366	-0.745390
53	6	0	-5.412286	-1.696164	-4.150237
54	1	0	-4.545961	-1.938511	-4.775000
55	1	0	-5.995000	-2.609263	-3.994622
56	1	0	-6.054738	-1.003164	-4.702166
57	6	0	-6.268374	-0.764706	-1.977910
58	1	0	-6.852259	-1.677093	-1.809922
59	1	0	-6.012003	-0.337812	-1.002513
60	1	0	-6.894034	-0.042857	-2.515579
61	15	0	-1.551378	2.193440	-0.462376
62	15	0	-1.699936	-1.296916	1.250437
63	6	0	-0.573530	3.595327	-1.163823
64	6	0	0.455024	3.257191	-2.061289
65	6	0	-0.800853	4.946868	-0.870570
66	6	0	1.239140	4.248036	-2.649890
67	1	0	0.646430	2.212314	-2.288793
68	6	0	-0.002071	5.938895	-1.449269
69	1	0	-1.598896	5.234444	-0.193581
70	6	0	1.016843	5.594044	-2.339013
71	1	0	2.029240	3.969663	-3.342348
72	1	0	-0.184699	6.982728	-1.206993
73	1	0	1.634460	6.367157	-2.788327
74	6	0	-2.660482	2.997227	0.768706
75	6	0	-2.066877	3.580387	1.902158
76	6	0	-4.056439	3.005684	0.653090
77	6	0	-2.851974	4.187522	2.880677
78	1	0	-0.986006	3.563858	2.012959
79	6	0	-4.843532	3.589057	1.650105
80	1	0	-4.536972	2.559460	-0.211914
81	6	0	-4.245636	4.189169	2.759258
82	1	0	-2.377738	4.646392	3.744402
83	1	0	-5.925921	3.578814	1.552579
84	1	0	-4.859829	4.648574	3.529162
85	6	0	-0.952189	-2.616415	2.302043

86	6	0	-1.232192	-2.751103	3.670572
87	6	0	0.009153	-3.465456	1.726022
88	6	0	-0.568279	-3.712748	4.438638
89	1	0	-1.967703	-2.109856	4.145222
90	6	0	0.656067	-4.437262	2.488731
91	1	0	0.245305	-3.372072	0.673401
92	6	0	0.373144	-4.561055	3.852609
93	1	0	-0.796225	-3.800579	5.497868
94	1	0	1.386134	-5.090434	2.017631
95	1	0	0.883183	-5.310502	4.451778
96	6	0	-3.034092	-0.628182	2.333124
97	6	0	-2.719791	0.445071	3.180163
98	6	0	-4.330714	-1.163511	2.362924
99	6	0	-3.678587	0.963851	4.051736
100	1	0	-1.724377	0.879989	3.149076
101	6	0	-5.294330	-0.630906	3.221652
102	1	0	-4.592307	-1.995993	1.716394
103	6	0	-4.969526	0.430459	4.070584
104	1	0	-3.422008	1.797548	4.698084
105	1	0	-6.297678	-1.049085	3.229828
106	1	0	-5.720179	0.843617	4.739244
107	46	0	-0.225003	0.440470	0.484188
108	6	0	7.045355	1.389653	0.629410
109	6	0	7.446076	0.527310	-0.391387
110	6	0	8.708384	0.611659	-0.960497
111	6	0	9.575189	1.601378	-0.469637
112	6	0	9.173459	2.466973	0.556487
113	6	0	7.892528	2.370214	1.124000
114	6	0	5.631819	1.006565	0.995673
115	6	0	6.280223	-0.386975	-0.665823
116	1	0	9.013036	-0.061648	-1.756796
117	1	0	10.572479	1.699701	-0.890434
118	1	0	9.865265	3.224056	0.916558
119	1	0	7.574989	3.038904	1.919225

120	8	0	4.938555	1.561498	1.844073
121	8	0	6.237646	-1.258384	-1.536842
122	7	0	5.263937	-0.072543	0.211966
123	6	0	1.527643	1.756925	2.705408
124	1	0	2.487212	1.918914	3.214048
125	1	0	1.076126	2.738942	2.527100
126	1	0	0.874405	1.191394	3.378658

TS-2ai-6

Charge = 0 Multiplicity = 1

Center Number	Atomic Number	Atomic Type	Coordinates (Angstroms) X	Y	Z
1	6	0	1.623969	0.952162	1.529501
2	6	0	2.239534	1.731594	0.512084
3	6	0	3.072017	1.183008	-0.463082
4	6	0	3.367607	-0.184363	-0.389303
5	6	0	2.500739	-1.141302	0.411828
6	6	0	1.598792	-0.450007	1.419832
7	1	0	2.093927	2.808098	0.536412
8	1	0	3.895657	-0.640171	-1.220228
9	1	0	1.320289	-1.045229	2.284617
10	6	0	1.760285	-1.901166	-0.754933
11	8	0	0.790244	-1.460329	-1.370055
12	7	0	2.422333	-3.061574	-1.006577
13	6	0	2.223281	-3.840581	-2.269727
14	6	0	0.760052	-4.310307	-2.371141
15	6	0	2.596682	-2.944560	-3.464907
16	6	0	3.132184	-5.078666	-2.256334
17	1	0	0.506843	-4.969596	-1.532956
18	1	0	0.068045	-3.467092	-2.374537
19	1	0	0.624245	-4.878336	-3.298752
20	1	0	3.639719	-2.615891	-3.386238

21	1	0	2.482438	-3.498044	-4.404027
22	1	0	1.953234	-2.061509	-3.505867
23	1	0	2.968522	-5.640955	-3.181598
24	1	0	4.193388	-4.814150	-2.211366
25	1	0	2.898839	-5.741888	-1.416179
26	6	0	3.595352	-3.238260	-0.131099
27	6	0	3.291426	-2.309338	1.043359
28	1	0	4.514162	-2.939278	-0.649134
29	1	0	3.687676	-4.278237	0.187831
30	1	0	2.638347	-2.820457	1.756787
31	1	0	4.190998	-1.958812	1.545912
32	6	0	-2.914826	2.892041	-2.755772
33	6	0	-2.808733	1.982759	-1.690931
34	6	0	-3.576971	0.814298	-1.770419
35	6	0	-4.392768	0.494059	-2.863137
36	6	0	-4.444288	1.411977	-3.915827
37	6	0	-3.721499	2.606289	-3.856190
38	6	0	-5.211995	-0.799082	-2.782997
39	6	0	-4.350348	-1.842883	-2.063675
40	6	0	-3.556825	-1.398746	-1.002746
41	6	0	-2.794458	-2.245407	-0.188135
42	6	0	-2.851577	-3.619459	-0.461879
43	6	0	-3.611208	-4.094653	-1.532457
44	6	0	-4.351120	-3.216531	-2.328491
45	1	0	-2.349443	3.816903	-2.733717
46	1	0	-5.057547	1.207013	-4.786505
47	1	0	-3.784610	3.316599	-4.675766
48	1	0	-2.299444	-4.321165	0.152576
49	1	0	-3.635459	-5.160529	-1.741205
50	1	0	-4.941899	-3.612214	-3.147527
51	8	0	-3.510820	-0.052183	-0.699510
52	6	0	-5.677405	-1.280753	-4.164358
53	1	0	-4.831222	-1.486992	-4.828477
54	1	0	-6.274949	-2.192626	-4.071547

55	1	0	-6.318308	-0.531966	-4.639655
56	6	0	-6.461239	-0.522133	-1.899579
57	1	0	-7.058138	-1.435305	-1.792379
58	1	0	-6.173571	-0.181393	-0.899276
59	1	0	-7.085498	0.252123	-2.360542
60	15	0	-1.637447	2.210131	-0.275767
61	15	0	-1.782182	-1.427558	1.118527
62	6	0	-0.639000	3.634118	-0.899735
63	6	0	0.388070	3.338661	-1.812354
64	6	0	-0.875040	4.971578	-0.550719
65	6	0	1.151854	4.361153	-2.375045
66	1	0	0.589825	2.304525	-2.079579
67	6	0	-0.090325	5.992704	-1.095713
68	1	0	-1.671300	5.223939	0.142401
69	6	0	0.919671	5.691736	-2.011576
70	1	0	1.935202	4.117993	-3.087258
71	1	0	-0.278195	7.024443	-0.809753
72	1	0	1.523516	6.487335	-2.439817
73	6	0	-2.669813	2.950913	1.056404
74	6	0	-2.009820	3.413918	2.209067
75	6	0	-4.067531	3.020224	0.998857
76	6	0	-2.732941	3.959753	3.268132
77	1	0	-0.925969	3.350736	2.272301
78	6	0	-4.792573	3.543150	2.073817
79	1	0	-4.597520	2.668910	0.118830
80	6	0	-4.129467	4.020484	3.205265
81	1	0	-2.209043	4.325369	4.147467
82	1	0	-5.877418	3.581202	2.020594
83	1	0	-4.695194	4.432239	4.036775
84	6	0	-0.997623	-2.842650	2.003937
85	6	0	-1.161590	-3.055075	3.381475
86	6	0	-0.112457	-3.673023	1.294950
87	6	0	-0.459587	-4.076224	4.028986
88	1	0	-1.834777	-2.426431	3.955496

89	6	0	0.573289	-4.702565	1.938682
90	1	0	0.036396	-3.516646	0.234316
91	6	0	0.405930	-4.905212	3.312078
92	1	0	-0.596815	-4.225279	5.096929
93	1	0	1.246398	-5.337017	1.367623
94	1	0	0.947194	-5.699958	3.818247
95	6	0	-3.072455	-0.862348	2.310002
96	6	0	-2.752102	0.175871	3.197075
97	6	0	-4.347758	-1.444702	2.373685
98	6	0	-3.685859	0.620350	4.134793
99	1	0	-1.774196	0.645377	3.142302
100	6	0	-5.285972	-0.988569	3.301426
101	1	0	-4.612832	-2.251957	1.697319
102	6	0	-4.956626	0.042943	4.185087
103	1	0	-3.425549	1.430453	4.808907
104	1	0	-6.273381	-1.441889	3.335215
105	1	0	-5.688591	0.398103	4.905791
106	46	0	-0.314828	0.376596	0.523975
107	6	0	7.097511	1.357148	0.823810
108	6	0	7.478212	0.495401	-0.204932
109	6	0	8.708705	0.613926	-0.834400
110	6	0	9.565215	1.636470	-0.395057
111	6	0	9.183533	2.501705	0.638958
112	6	0	7.933601	2.371570	1.266078
113	6	0	5.715281	0.929942	1.258782
114	6	0	6.329068	-0.458690	-0.412371
115	1	0	8.997787	-0.058863	-1.636984
116	1	0	10.538366	1.760886	-0.862959
117	1	0	9.866371	3.284857	0.958152
118	1	0	7.631154	3.040619	2.066941
119	8	0	5.036383	1.483374	2.119706
120	8	0	6.271971	-1.329715	-1.284226
121	7	0	5.347837	-0.177370	0.514955
122	1	0	1.253262	1.449253	2.422231

123	6	0	3.802044	2.046903	-1.459798
124	1	0	3.641119	1.708720	-2.491553
125	1	0	3.484374	3.091212	-1.392653
126	1	0	4.886043	2.015077	-1.278942

TS-4a-1

Charge = 0 Multiplicity = 1

Center Number	Atomic Number	Atomic Type	Coordinates (Angstroms) X	Y	Z
1	6	0	-2.088961	-1.049828	-0.816739
2	6	0	-2.341901	-1.488652	-2.151100
3	6	0	-2.856923	-0.506291	-3.092658
4	6	0	-3.114938	0.762770	-2.729164
5	6	0	-2.881392	1.307987	-1.341744
6	6	0	-2.273203	0.288479	-0.398119
7	1	0	-1.939330	-1.814859	-0.062637
8	1	0	-3.058943	-0.837122	-4.108438
9	1	0	-3.508452	1.463665	-3.462977
10	1	0	-2.477452	0.466765	0.654059
11	6	0	-2.026619	2.592194	-1.589843
12	8	0	-0.819222	2.586335	-1.826131
13	7	0	-2.849404	3.678635	-1.591329
14	6	0	-2.433385	5.004633	-2.149699
15	6	0	-1.205386	5.537854	-1.389842
16	6	0	-2.113434	4.829518	-3.645490
17	6	0	-3.578606	6.015569	-1.982500
18	1	0	-1.436874	5.674236	-0.327779
19	1	0	-0.355283	4.860893	-1.481102
20	1	0	-0.921261	6.513156	-1.801332
21	1	0	-2.990950	4.456628	-4.187267
22	1	0	-1.823617	5.789819	-4.086852
23	1	0	-1.289652	4.124204	-3.786051

24	1	0	-3.253084	6.981213	-2.383222
25	1	0	-4.480662	5.719133	-2.527087
26	1	0	-3.836842	6.162544	-0.927797
27	6	0	-4.261418	3.313900	-1.398873
28	6	0	-4.189779	1.918858	-0.771657
29	1	0	-4.791120	3.304963	-2.360511
30	1	0	-4.763094	4.026778	-0.741734
31	1	0	-4.084295	2.009572	0.314146
32	1	0	-5.068460	1.305917	-0.984951
33	6	0	4.197148	-1.173189	-2.583955
34	6	0	3.408149	-0.815343	-1.479819
35	6	0	3.912894	0.173846	-0.624547
36	6	0	5.126533	0.838377	-0.836147
37	6	0	5.870782	0.467426	-1.960647
38	6	0	5.415002	-0.535795	-2.819996
39	6	0	5.568364	1.866727	0.212729
40	6	0	4.308868	2.583383	0.714768
41	6	0	3.147525	1.820132	0.859911
42	6	0	1.950740	2.314900	1.398003
43	6	0	1.938034	3.651759	1.819293
44	6	0	3.073808	4.448769	1.665986
45	6	0	4.245972	3.922454	1.116826
46	1	0	3.849357	-1.937395	-3.270863
47	1	0	6.817265	0.953923	-2.170911
48	1	0	6.012039	-0.817670	-3.682808
49	1	0	1.040665	4.073913	2.258211
50	1	0	3.048794	5.486538	1.986387
51	1	0	5.117584	4.560018	1.018229
52	8	0	3.148445	0.492587	0.479353
53	6	0	6.612977	2.847301	-0.339128
54	1	0	6.225458	3.414123	-1.192403
55	1	0	6.924389	3.554554	0.435501
56	1	0	7.513620	2.313628	-0.657286
57	6	0	6.185912	1.095170	1.413438

58	1	0	6.484755	1.797086	2.200668
59	1	0	5.469417	0.386063	1.841802
60	1	0	7.071041	0.535516	1.089579
61	15	0	1.703158	-1.461037	-1.188542
62	15	0	0.522109	1.156201	1.430352
63	6	0	1.365621	-2.391507	-2.743782
64	6	0	0.722683	-1.701804	-3.784275
65	6	0	1.708979	-3.739201	-2.928699
66	6	0	0.436890	-2.343835	-4.989711
67	1	0	0.429251	-0.664806	-3.640613
68	6	0	1.409428	-4.384840	-4.131113
69	1	0	2.202953	-4.288566	-2.133003
70	6	0	0.776219	-3.688728	-5.163865
71	1	0	-0.064317	-1.798877	-5.785362
72	1	0	1.671722	-5.431790	-4.259246
73	1	0	0.541947	-4.193951	-6.097178
74	6	0	1.900287	-2.788390	0.072493
75	6	0	0.783611	-3.604414	0.329009
76	6	0	3.067254	-2.976958	0.823363
77	6	0	0.842080	-4.606633	1.294418
78	1	0	-0.138883	-3.462211	-0.225168
79	6	0	3.116951	-3.968858	1.807967
80	1	0	3.940691	-2.356883	0.644784
81	6	0	2.011752	-4.789906	2.039907
82	1	0	-0.036499	-5.222585	1.462928
83	1	0	4.025841	-4.100380	2.389456
84	1	0	2.057643	-5.562537	2.803132
85	6	0	-0.845970	2.123186	2.200578
86	6	0	-1.512303	1.705851	3.363219
87	6	0	-1.342819	3.236515	1.500202
88	6	0	-2.646035	2.389670	3.813273
89	1	0	-1.161348	0.842192	3.917904
90	6	0	-2.465003	3.925157	1.958813
91	1	0	-0.853060	3.553942	0.587274

92	6	0	-3.125314	3.500800	3.116578
93	1	0	-3.151768	2.050353	4.713573
94	1	0	-2.832361	4.782359	1.401562
95	1	0	-4.007534	4.028905	3.468299
96	6	0	0.990047	-0.038978	2.754263
97	6	0	0.251088	-1.228150	2.866666
98	6	0	2.039163	0.197521	3.655318
99	6	0	0.550008	-2.155087	3.865613
100	1	0	-0.560225	-1.430466	2.173823
101	6	0	2.347713	-0.740544	4.642794
102	1	0	2.620929	1.111974	3.589734
103	6	0	1.603414	-1.917470	4.751787
104	1	0	-0.028256	-3.070789	3.935804
105	1	0	3.169118	-0.548126	5.328294
106	1	0	1.845576	-2.647835	5.519301
107	46	0	-0.090441	-0.028873	-0.551368
108	6	0	-4.431100	-2.252793	1.328300
109	6	0	-3.485044	-3.217114	1.676171
110	6	0	-2.879130	-3.223700	2.924392
111	6	0	-3.250283	-2.217317	3.830034
112	6	0	-4.198433	-1.246242	3.479401
113	6	0	-4.806875	-1.253203	2.213912
114	6	0	-4.790601	-2.488897	-0.115115
115	6	0	-3.229634	-4.042875	0.444992
116	1	0	-2.132468	-3.968784	3.182174
117	1	0	-2.786360	-2.180232	4.811766
118	1	0	-4.455896	-0.469974	4.194714
119	1	0	-5.527439	-0.492239	1.928492
120	7	0	-4.059672	-3.584792	-0.571625
121	8	0	-5.558060	-1.813226	-0.790988
122	8	0	-2.380089	-4.923183	0.328682
123	6	0	-2.320880	-2.866664	-2.484636
124	1	0	-1.575518	-3.486300	-1.982600
125	1	0	-3.355584	-3.316881	-1.710323

126	1	0	-2.460077	-3.124640	-3.534314

TS-4a-2

Charge = 0 Multiplicity = 1

Center Number	Atomic Number	Atomic Type	Coordinates (Angstroms) X	Y	Z
1	6	0	-2.161872	-0.943062	-0.699227
2	6	0	-1.429932	-1.544675	-1.774024
3	6	0	-1.304686	-0.759180	-3.006033
4	6	0	-1.682200	0.530383	-3.095858
5	6	0	-2.221494	1.288768	-1.913144
6	6	0	-2.619034	0.377356	-0.785785
7	1	0	-2.548765	-1.564122	0.104284
8	1	0	-0.916811	-1.271648	-3.881725
9	1	0	-1.578466	1.078428	-4.029271
10	1	0	-3.003747	0.862348	0.099309
11	6	0	-1.058599	2.254444	-1.451648
12	8	0	-0.070863	1.912837	-0.804013
13	7	0	-1.299822	3.497900	-1.932853
14	6	0	-0.277269	4.589442	-1.851656
15	6	0	0.040106	4.873422	-0.371807
16	6	0	0.993991	4.151171	-2.604085
17	6	0	-0.837548	5.864975	-2.496962
18	1	0	-0.871319	5.157171	0.167932
19	1	0	0.475885	4.000100	0.112926
20	1	0	0.752809	5.702838	-0.298979
21	1	0	0.765697	3.930901	-3.653877
22	1	0	1.737375	4.956354	-2.579135
23	1	0	1.433594	3.261008	-2.147436
24	1	0	-0.073290	6.647264	-2.443272
25	1	0	-1.089116	5.717098	-3.552414

26	1	0	-1.726085	6.229310	-1.970694
27	6	0	-2.512976	3.555704	-2.758821
28	6	0	-3.301041	2.322993	-2.307216
29	1	0	-2.253373	3.519632	-3.825336
30	1	0	-3.069085	4.477007	-2.576734
31	1	0	-3.905408	2.556351	-1.427403
32	1	0	-3.967838	1.935738	-3.078498
33	6	0	4.379068	-0.564070	-2.366129
34	6	0	3.508360	-0.488044	-1.269715
35	6	0	3.655011	0.609415	-0.405422
36	6	0	4.547781	1.662404	-0.633717
37	6	0	5.378245	1.554750	-1.756091
38	6	0	5.310164	0.447117	-2.601997
39	6	0	4.644935	2.824051	0.366010
40	6	0	3.356920	2.898847	1.195371
41	6	0	2.542908	1.772343	1.321362
42	6	0	1.359994	1.744970	2.072948
43	6	0	1.022105	2.895882	2.793901
44	6	0	1.830701	4.032192	2.720673
45	6	0	2.974210	4.035583	1.920670
46	1	0	4.321914	-1.406621	-3.045613
47	1	0	6.102917	2.334100	-1.966746
48	1	0	5.980326	0.375551	-3.453852
49	1	0	0.124346	2.912307	3.402154
50	1	0	1.564535	4.921416	3.285035
51	1	0	3.583912	4.931842	1.877540
52	8	0	2.878257	0.583177	0.722110
53	6	0	4.888868	4.156402	-0.373705
54	1	0	4.069071	4.379560	-1.064898
55	1	0	4.979717	4.986056	0.332987
56	1	0	5.823068	4.126274	-0.941461
57	6	0	5.830701	2.544302	1.326350
58	1	0	5.923849	3.350440	2.063462
59	1	0	5.684845	1.601968	1.866420

60	1	0	6.769243	2.475540	0.763778
61	15	0	2.134217	-1.697209	-0.971961
62	15	0	0.359204	0.201855	1.973158
63	6	0	2.119672	-2.599928	-2.585708
64	6	0	1.724486	-1.869874	-3.721301
65	6	0	2.431071	-3.958289	-2.732850
66	6	0	1.639915	-2.483483	-4.969664
67	1	0	1.483828	-0.815169	-3.624775
68	6	0	2.331470	-4.576607	-3.983971
69	1	0	2.751654	-4.544638	-1.878591
70	6	0	1.935631	-3.844495	-5.103741
71	1	0	1.336141	-1.901590	-5.835988
72	1	0	2.571214	-5.632573	-4.078651
73	1	0	1.859717	-4.327246	-6.074341
74	6	0	2.828281	-2.920073	0.213828
75	6	0	2.025958	-4.023390	0.555496
76	6	0	4.089423	-2.784053	0.808070
77	6	0	2.494238	-4.990310	1.443368
78	1	0	1.034934	-4.124071	0.119178
79	6	0	4.542106	-3.737235	1.724833
80	1	0	4.723153	-1.938194	0.559416
81	6	0	3.752667	-4.845412	2.036572
82	1	0	1.870323	-5.846224	1.687112
83	1	0	5.517528	-3.614819	2.188362
84	1	0	4.111117	-5.589059	2.743261
85	6	0	-1.241122	0.702703	2.744094
86	6	0	-1.828551	-0.009088	3.799864
87	6	0	-1.957771	1.764148	2.161186
88	6	0	-3.108714	0.326380	4.253088
89	1	0	-1.296134	-0.829929	4.270410
90	6	0	-3.231532	2.099609	2.617510
91	1	0	-1.517818	2.315791	1.335766
92	6	0	-3.814394	1.376037	3.663022
93	1	0	-3.552315	-0.238775	5.068952

94	1	0	-3.783183	2.898743	2.133377
95	1	0	-4.814269	1.626356	4.007165
96	6	0	1.128395	-0.876160	3.251819
97	6	0	0.861841	-2.252293	3.195398
98	6	0	1.942674	-0.380298	4.281952
99	6	0	1.383730	-3.115550	4.160281
100	1	0	0.252180	-2.647792	2.387609
101	6	0	2.478973	-1.247635	5.235428
102	1	0	2.160910	0.681911	4.340728
103	6	0	2.196940	-2.615391	5.179724
104	1	0	1.170904	-4.178898	4.102656
105	1	0	3.114488	-0.853654	6.024442
106	1	0	2.614087	-3.288851	5.923877
107	46	0	0.063827	-0.795626	-0.261534
108	6	0	-1.401344	-3.048887	-1.910675
109	1	0	-1.413621	-3.540920	-0.932520
110	1	0	-2.294218	-3.377048	-2.461138
111	1	0	-0.517167	-3.386791	-2.459501
112	6	0	-6.625949	-0.329803	0.273128
113	6	0	-6.390098	-1.520398	-0.412489
114	6	0	-7.159762	-2.651955	-0.186072
115	6	0	-8.192211	-2.555092	0.761011
116	6	0	-8.429404	-1.357496	1.449523
117	6	0	-7.641320	-0.219755	1.211807
118	6	0	-5.592304	0.654821	-0.214787
119	6	0	-5.214197	-1.264914	-1.327511
120	1	0	-6.968502	-3.577844	-0.721786
121	1	0	-8.816611	-3.420745	0.967054
122	1	0	-9.234402	-1.312132	2.178636
123	1	0	-7.815443	0.710639	1.745454
124	8	0	-5.455326	1.804772	0.213385
125	8	0	-4.696726	-2.106763	-2.060048
126	7	0	-4.819559	0.051231	-1.183201

TS-4a-3

Charge = 0 Multiplicity = 1

Center Number	Atomic Number	Atomic Type	Coordinates (Angstroms) X	Y	Z
1	6	0	0.374603	1.075657	-3.782193
2	6	0	0.181995	1.251235	-2.406466
3	6	0	-0.918827	2.017916	-2.007616
4	6	0	-1.798958	2.647213	-2.891018
5	6	0	-1.588356	2.425595	-4.257247
6	6	0	-0.517735	1.642174	-4.695843
7	6	0	-2.835422	3.614616	-2.301032
8	6	0	-3.301389	3.046817	-0.955084
9	6	0	-2.379205	2.317409	-0.198087
10	6	0	-2.688652	1.697549	1.019077
11	6	0	-3.959428	1.947875	1.562052
12	6	0	-4.884572	2.721717	0.861714
13	6	0	-4.569711	3.239547	-0.398101
14	1	0	1.216010	0.493516	-4.142217
15	1	0	-2.248925	2.877165	-4.989636
16	1	0	-0.365597	1.487869	-5.760531
17	1	0	-4.236606	1.515427	2.516909
18	1	0	-5.866544	2.904274	1.288973
19	1	0	-5.320232	3.804250	-0.940503
20	8	0	-1.089584	2.166566	-0.646819
21	6	0	-4.011874	3.853336	-3.258865
22	1	0	-4.551542	2.924032	-3.471282
23	1	0	-4.716992	4.576411	-2.837607
24	1	0	-3.659370	4.275506	-4.204760
25	6	0	-2.115679	4.965874	-2.031487
26	1	0	-2.810949	5.682446	-1.578698
27	1	0	-1.267755	4.832934	-1.350854
28	1	0	-1.738983	5.388568	-2.970192

29	15	0	1.256028	0.559353	-1.067305
30	15	0	-1.476741	0.479985	1.697770
31	6	0	2.636573	-0.171751	-2.044350
32	6	0	2.412340	-1.421450	-2.646495
33	6	0	3.880786	0.451327	-2.212535
34	6	0	3.405507	-2.024804	-3.416367
35	1	0	1.456990	-1.920432	-2.510866
36	6	0	4.879694	-0.163032	-2.972933
37	1	0	4.080411	1.410667	-1.746417
38	6	0	4.644357	-1.397577	-3.578596
39	1	0	3.219352	-2.992360	-3.875382
40	1	0	5.845498	0.323593	-3.081090
41	1	0	5.426628	-1.878534	-4.159016
42	6	0	2.010361	2.094095	-0.372330
43	6	0	2.641971	2.012411	0.876461
44	6	0	1.997269	3.323136	-1.052008
45	6	0	3.246793	3.140847	1.434771
46	1	0	2.659429	1.070646	1.413331
47	6	0	2.591696	4.451614	-0.485127
48	1	0	1.522963	3.404749	-2.024873
49	6	0	3.218449	4.363056	0.760962
50	1	0	3.729081	3.064640	2.404380
51	1	0	2.568327	5.397927	-1.019756
52	1	0	3.680651	5.241878	1.203378
53	6	0	-2.523212	-0.393402	2.944672
54	6	0	-2.292652	-0.351774	4.326998
55	6	0	-3.594953	-1.166252	2.461248
56	6	0	-3.116572	-1.064312	5.204440
57	1	0	-1.476628	0.239337	4.728970
58	6	0	-4.427482	-1.859508	3.339266
59	1	0	-3.782960	-1.212151	1.392484
60	6	0	-4.187250	-1.815138	4.716562
61	1	0	-2.922825	-1.021144	6.273103
62	1	0	-5.261155	-2.436804	2.948207

63	1	0	-4.829911	-2.361568	5.401617
64	6	0	-0.338037	1.478787	2.738721
65	6	0	0.750984	0.818775	3.333556
66	6	0	-0.527846	2.845844	2.984237
67	6	0	1.609506	1.507445	4.190455
68	1	0	0.945259	-0.225505	3.108762
69	6	0	0.350344	3.538397	3.821317
70	1	0	-1.360383	3.371957	2.526855
71	6	0	1.411183	2.869026	4.436559
72	1	0	2.446415	0.983083	4.643461
73	1	0	0.199358	4.600153	3.997902
74	1	0	2.087663	3.409021	5.094014
75	46	0	-0.145794	-0.934786	0.273116
76	6	0	5.271390	-0.530578	1.287264
77	6	0	6.087201	-1.107828	0.314264
78	6	0	7.326656	-0.572900	-0.003249
79	6	0	7.731981	0.578734	0.690258
80	6	0	6.915307	1.154169	1.673007
81	6	0	5.665757	0.598362	1.989124
82	6	0	4.008198	-1.350061	1.343415
83	6	0	5.346794	-2.306558	-0.224853
84	1	0	7.955726	-1.026129	-0.764196
85	1	0	8.693594	1.032072	0.464323
86	1	0	7.255473	2.044297	2.195571
87	1	0	5.028185	1.036763	2.750466
88	8	0	3.058012	-1.153839	2.103593
89	8	0	5.769701	-3.109067	-1.048423
90	7	0	4.113139	-2.344926	0.402122
91	6	0	0.177391	-3.203113	0.879120
92	6	0	0.994273	-3.861777	-0.077843
93	6	0	0.442535	-4.075704	-1.413099
94	6	0	-0.851203	-3.839042	-1.686290
95	6	0	-1.832385	-3.346145	-0.661922
96	6	0	-1.159099	-2.828897	0.612368

97	1	0	0.560222	-3.113254	1.892504
98	1	0	1.103316	-4.485188	-2.172995
99	1	0	-1.250519	-4.046362	-2.676160
100	1	0	-1.829345	-2.710867	1.459385
101	6	0	-2.861925	-4.462552	-0.314905
102	6	0	-2.762595	-2.273490	-1.290331
103	8	0	-2.379796	-1.426276	-2.091990
104	1	0	-2.546385	-5.071052	0.535959
105	1	0	-2.979232	-5.120965	-1.182298
106	6	0	-4.175426	-3.709756	-0.061174
107	1	0	-4.335408	-3.506615	1.002279
108	1	0	-5.032490	-4.280969	-0.428556
109	7	0	-4.029538	-2.455187	-0.813647
110	6	0	-5.227761	-1.687896	-1.269483
111	6	0	-6.334377	-1.809287	-0.205454
112	6	0	-4.895107	-0.195505	-1.437758
113	6	0	-5.701928	-2.287535	-2.604432
114	1	0	-6.664280	-2.841465	-0.053876
115	1	0	-6.006175	-1.402571	0.757121
116	1	0	-7.205593	-1.232755	-0.533429
117	1	0	-4.204989	-0.022529	-2.261459
118	1	0	-5.823174	0.354766	-1.627800
119	1	0	-4.443491	0.203650	-0.526713
120	1	0	-6.589175	-1.757249	-2.969605
121	1	0	-4.913786	-2.202161	-3.360053
122	1	0	-5.961388	-3.347008	-2.491446
123	6	0	2.297797	-4.335660	0.243289
124	1	0	3.138896	-3.334275	0.221724
125	1	0	2.443193	-4.614112	1.291123
126	1	0	2.725038	-5.059405	-0.454675

TS-4a-4

Charge = 0 Multiplicity = 1

Center Number	Atomic Number	Atomic Type	Coordinates (Angstroms) X	Y	Z
1	6	0	-1.482066	-0.389976	3.071987
2	6	0	-0.356698	-0.501775	2.240256
3	6	0	0.045696	-1.794740	1.886270
4	6	0	-0.660830	-2.954157	2.230769
5	6	0	-1.792349	-2.793383	3.035437
6	6	0	-2.183907	-1.525003	3.472641
7	6	0	-0.103641	-4.309549	1.773158
8	6	0	0.595413	-4.103172	0.424314
9	6	0	1.276606	-2.901499	0.224176
10	6	0	2.038573	-2.613144	-0.914745
11	6	0	2.102841	-3.589664	-1.917125
12	6	0	1.412903	-4.794820	-1.761952
13	6	0	0.670624	-5.049759	-0.605381
14	1	0	-1.821882	0.590173	3.388062
15	1	0	-2.370622	-3.659069	3.339460
16	1	0	-3.053878	-1.421153	4.113780
17	1	0	2.673885	-3.406127	-2.820967
18	1	0	1.456755	-5.543694	-2.547580
19	1	0	0.152243	-5.997477	-0.507910
20	8	0	1.216690	-1.902027	1.167958
21	6	0	-1.201197	-5.381128	1.691358
22	1	0	-1.988838	-5.096735	0.984854
23	1	0	-0.782644	-6.341408	1.375619
24	1	0	-1.657870	-5.545969	2.671696
25	6	0	0.972821	-4.759825	2.799511
26	1	0	1.418999	-5.711911	2.489215
27	1	0	1.773546	-4.016966	2.883802
28	1	0	0.520753	-4.890372	3.789517

29	15	0	0.543987	0.969814	1.561402
30	15	0	2.708302	-0.906634	-1.019748
31	6	0	-0.784972	2.242630	1.693029
32	6	0	-1.914241	2.035454	0.878948
33	6	0	-0.714770	3.402426	2.474237
34	6	0	-2.949854	2.966840	0.852736
35	1	0	-2.001246	1.143677	0.265355
36	6	0	-1.747734	4.345192	2.430319
37	1	0	0.142598	3.584425	3.113520
38	6	0	-2.864477	4.133025	1.620501
39	1	0	-3.812388	2.773158	0.222652
40	1	0	-1.673059	5.247741	3.031726
41	1	0	-3.659536	4.873101	1.582548
42	6	0	1.772914	1.344504	2.881954
43	6	0	2.497370	2.549875	2.821058
44	6	0	2.079710	0.421208	3.894108
45	6	0	3.470653	2.837292	3.778711
46	1	0	2.301342	3.252974	2.018578
47	6	0	3.075642	0.702124	4.832423
48	1	0	1.545398	-0.521363	3.958009
49	6	0	3.766863	1.914348	4.785772
50	1	0	4.010548	3.779499	3.726059
51	1	0	3.304146	-0.027831	5.604752
52	1	0	4.535965	2.134251	5.521647
53	6	0	3.790556	-0.951414	-2.513140
54	6	0	4.869990	-1.848581	-2.595152
55	6	0	3.562742	-0.059056	-3.569742
56	6	0	5.692281	-1.861925	-3.721442
57	1	0	5.069166	-2.535322	-1.777167
58	6	0	4.389928	-0.071813	-4.696318
59	1	0	2.742551	0.649599	-3.503126
60	6	0	5.452398	-0.973739	-4.775420
61	1	0	6.522306	-2.561456	-3.775009
62	1	0	4.203264	0.626402	-5.507904

63	1	0	6.096109	-0.982727	-5.651083
64	6	0	3.931254	-0.813388	0.352548
65	6	0	4.383811	0.457824	0.736568
66	6	0	4.426559	-1.947813	1.012042
67	6	0	5.327230	0.589761	1.756344
68	1	0	3.983573	1.344704	0.252549
69	6	0	5.360483	-1.810766	2.040619
70	1	0	4.080806	-2.938483	0.732106
71	6	0	5.813367	-0.542643	2.413576
72	1	0	5.660977	1.579228	2.052874
73	1	0	5.732201	-2.695535	2.551079
74	1	0	6.536168	-0.436620	3.218139
75	46	0	1.129967	0.785738	-0.973991
76	6	0	-0.179125	4.964860	-0.880959
77	6	0	-1.150808	4.536471	-1.782017
78	6	0	-2.194752	5.361545	-2.171376
79	6	0	-2.242736	6.649912	-1.615190
80	6	0	-1.265919	7.080005	-0.706951
81	6	0	-0.210140	6.235887	-0.327618
82	6	0	0.751947	3.805581	-0.671977
83	6	0	-0.819622	3.110090	-2.136548
84	1	0	-2.951820	5.018187	-2.870714
85	1	0	-3.050677	7.323869	-1.888106
86	1	0	-1.330596	8.081217	-0.288830
87	1	0	0.546796	6.559807	0.380911
88	8	0	1.761371	3.817663	0.036435
89	8	0	-1.431424	2.423995	-2.947215
90	7	0	0.290605	2.732235	-1.401424
91	6	0	-1.420385	-1.991311	-2.073007
92	6	0	-1.207567	-0.984467	-3.096309
93	6	0	-2.380337	-0.284481	-3.579121
94	6	0	-3.617680	-0.570251	-3.129873
95	6	0	-3.897246	-1.583783	-2.063660
96	6	0	-2.644136	-2.263467	-1.577567

97	1	0	-0.554684	-2.526570	-1.697835
98	1	0	-2.236672	0.482510	-4.331399
99	1	0	-4.481018	-0.042211	-3.529363
100	1	0	-2.762836	-3.014013	-0.798395
101	6	0	-4.979283	-2.624418	-2.464976
102	6	0	-4.583012	-0.907360	-0.829026
103	8	0	-4.232559	0.166691	-0.349132
104	1	0	-4.557417	-3.500632	-2.963550
105	1	0	-5.695123	-2.149362	-3.145070
106	6	0	-5.677027	-2.968734	-1.145119
107	1	0	-5.170948	-3.787631	-0.615865
108	1	0	-6.717252	-3.259182	-1.300744
109	7	0	-5.584071	-1.717403	-0.381918
110	6	0	-6.295712	-1.478733	0.911201
111	6	0	-7.336133	-2.584305	1.145834
112	6	0	-5.265694	-1.497863	2.054133
113	6	0	-7.017994	-0.120679	0.848135
114	1	0	-8.106413	-2.593114	0.367055
115	1	0	-6.878090	-3.577723	1.198727
116	1	0	-7.833988	-2.397965	2.103295
117	1	0	-4.518378	-0.713744	1.918835
118	1	0	-5.765264	-1.341881	3.017461
119	1	0	-4.747563	-2.462933	2.089665
120	1	0	-7.559599	0.055082	1.784843
121	1	0	-6.307253	0.693608	0.696883
122	1	0	-7.743253	-0.110265	0.025859
123	6	0	0.075063	-0.665245	-3.532615
124	1	0	0.727222	0.260556	-2.509402
125	1	0	0.858971	-1.410401	-3.422184
126	1	0	0.188148	0.003855	-4.383191

TS-2f-1

Charge = 0 Multiplicity = 1

Center Number	Atomic Number	Atomic Type	Coordinates (Angstroms) X	Y	Z
1	6	0	-2.149746	-0.940642	-0.391051
2	6	0	-1.507019	-1.524703	-1.521691
3	6	0	-1.662839	-0.874437	-2.815345
4	6	0	-2.204847	0.353440	-2.976956
5	6	0	-2.619812	1.161518	-1.756056
6	6	0	-2.757754	0.314048	-0.514934
7	1	0	-2.348533	-1.524384	0.502999
8	1	0	-1.301475	-2.591160	-1.525511
9	1	0	-1.335295	-1.426797	-3.691182
10	1	0	-3.081295	0.838356	0.375537
11	6	0	-1.461273	2.220963	-1.563274
12	8	0	-0.306237	1.939089	-1.246476
13	7	0	-1.921701	3.460069	-1.865117
14	6	0	-1.007120	4.640765	-2.000711
15	6	0	-0.231189	4.853134	-0.687463
16	6	0	-0.041748	4.387276	-3.172456
17	6	0	-1.839346	5.901234	-2.283026
18	1	0	-0.924309	4.990417	0.150613
19	1	0	0.422881	4.010267	-0.465522
20	1	0	0.383251	5.756795	-0.771054
21	1	0	-0.597138	4.258532	-4.109177
22	1	0	0.636372	5.239758	-3.294179
23	1	0	0.559243	3.492104	-2.992734
24	1	0	-1.159318	6.754609	-2.372923
25	1	0	-2.400666	5.826893	-3.219830
26	1	0	-2.540061	6.115081	-1.468588
27	6	0	-3.289853	3.449066	-2.396603
28	6	0	-3.844203	2.091901	-1.951280

29	1	0	-3.272530	3.563049	-3.487074
30	1	0	-3.873863	4.272195	-1.980308
31	1	0	-4.356127	2.197789	-0.993407
32	1	0	-4.562839	1.676132	-2.658142
33	6	0	4.376601	-1.061366	-2.465023
34	6	0	3.589394	-0.845268	-1.321668
35	6	0	3.999705	0.171018	-0.448872
36	6	0	5.098919	1.005233	-0.689964
37	6	0	5.832951	0.779089	-1.857528
38	6	0	5.483395	-0.255730	-2.728742
39	6	0	5.440768	2.054699	0.375055
40	6	0	4.114795	2.579016	0.936690
41	6	0	3.086451	1.650689	1.116177
42	6	0	1.855268	1.953738	1.708057
43	6	0	1.656481	3.271266	2.142205
44	6	0	2.651833	4.232240	1.952353
45	6	0	3.868712	3.891503	1.355036
46	1	0	4.113684	-1.848531	-3.162443
47	1	0	6.689489	1.402342	-2.090118
48	1	0	6.074663	-0.431159	-3.623096
49	1	0	0.721974	3.551894	2.615500
50	1	0	2.481157	5.253694	2.280643
51	1	0	4.630275	4.653750	1.229569
52	8	0	3.265305	0.342745	0.706053
53	6	0	6.324638	3.181063	-0.177894
54	1	0	5.836157	3.712829	-1.001624
55	1	0	6.561846	3.904028	0.608717
56	1	0	7.278431	2.783483	-0.538089
57	6	0	6.197587	1.339575	1.529578
58	1	0	6.428554	2.053537	2.328533
59	1	0	5.596662	0.530003	1.957046
60	1	0	7.136437	0.912545	1.158451
61	15	0	2.008801	-1.753652	-0.993783
62	15	0	0.630254	0.582948	1.792957

63	6	0	1.806800	-2.663856	-2.587018
64	6	0	1.344848	-1.923345	-3.688826
65	6	0	2.107075	-4.022062	-2.759328
66	6	0	1.186743	-2.528251	-4.935161
67	1	0	1.105011	-0.870644	-3.566224
68	6	0	1.932805	-4.630948	-4.006266
69	1	0	2.478830	-4.610694	-1.926939
70	6	0	1.474573	-3.887755	-5.095631
71	1	0	0.830421	-1.941059	-5.777564
72	1	0	2.163352	-5.686601	-4.124120
73	1	0	1.342107	-4.363168	-6.063860
74	6	0	2.475732	-3.066937	0.207049
75	6	0	1.491837	-4.008586	0.557990
76	6	0	3.742257	-3.152051	0.798850
77	6	0	1.781684	-5.032302	1.458130
78	1	0	0.498298	-3.939300	0.121532
79	6	0	4.021315	-4.162462	1.723935
80	1	0	4.515579	-2.434811	0.541785
81	6	0	3.047765	-5.108247	2.048840
82	1	0	1.015750	-5.760725	1.710990
83	1	0	5.004576	-4.211095	2.184293
84	1	0	3.269697	-5.897116	2.762540
85	6	0	-0.811615	1.354965	2.647924
86	6	0	-1.257728	0.941746	3.911417
87	6	0	-1.541363	2.342111	1.961000
88	6	0	-2.412389	1.499110	4.470208
89	1	0	-0.712305	0.182769	4.463077
90	6	0	-2.683768	2.906263	2.526191
91	1	0	-1.211072	2.665359	0.981068
92	6	0	-3.127368	2.480778	3.782594
93	1	0	-2.749654	1.162820	5.447373
94	1	0	-3.236942	3.663222	1.976997
95	1	0	-4.027182	2.907784	4.217011
96	6	0	1.347188	-0.518400	3.082367

97	6	0	0.888926	-1.842918	3.141501
98	6	0	2.312763	-0.087796	4.004714
99	6	0	1.378603	-2.718867	4.111501
100	1	0	0.154713	-2.189683	2.419689
101	6	0	2.814991	-0.970418	4.963042
102	1	0	2.677269	0.934657	3.974687
103	6	0	2.347496	-2.286143	5.020093
104	1	0	1.019016	-3.742893	4.142080
105	1	0	3.569157	-0.628138	5.667069
106	1	0	2.739676	-2.972319	5.766221
107	46	0	0.096093	-0.567387	-0.280496
108	6	0	-6.304245	-2.024479	0.162507
109	6	0	-6.581910	-0.883589	0.913301
110	6	0	-7.534149	-0.890798	1.922097
111	6	0	-8.217589	-2.094374	2.159344
112	6	0	-7.940777	-3.241271	1.402750
113	6	0	-6.972705	-3.219377	0.385631
114	6	0	-5.224265	-1.641007	-0.824055
115	6	0	-5.663761	0.198294	0.400226
116	1	0	-7.741270	0.001868	2.506001
117	1	0	-8.971826	-2.140247	2.940834
118	1	0	-8.484704	-4.159739	1.608435
119	1	0	-6.752454	-4.105161	-0.204028
120	7	0	-4.922149	-0.303714	-0.646283
121	8	0	-4.710997	-2.407074	-1.637046
122	8	0	-5.587079	1.339861	0.863651
123	6	0	-2.306240	0.994968	-4.334370
124	1	0	-3.337200	1.276240	-4.584042
125	1	0	-1.702298	1.911746	-4.392305
126	1	0	-1.946950	0.313825	-5.112616

TS-2f-2

Charge = 0 Multiplicity = 1

Center Number	Atomic Number	Atomic Type	Coordinates (Angstroms) X	Y	Z
1	6	0	2.088142	0.730620	-0.111513
2	6	0	3.074416	-0.275779	0.003624
3	6	0	3.536537	-0.701889	1.389126
4	6	0	2.990826	0.140211	2.513988
5	6	0	2.063339	1.095531	2.342690
6	6	0	1.534446	1.396917	1.018487
7	1	0	1.898487	1.132304	-1.100032
8	1	0	3.380047	-0.088798	3.503554
9	1	0	1.086538	2.376396	0.873473
10	6	0	-2.424219	-3.594057	-2.128774
11	6	0	-2.342471	-2.328561	-1.527039
12	6	0	-3.458169	-1.892352	-0.800254
13	6	0	-4.625793	-2.646635	-0.640554
14	6	0	-4.659488	-3.908778	-1.242275
15	6	0	-3.570751	-4.374805	-1.982903
16	6	0	-5.791379	-2.004943	0.121062
17	6	0	-5.190997	-1.169016	1.256709
18	6	0	-3.992149	-0.493853	1.002821
19	6	0	-3.364651	0.347490	1.930421
20	6	0	-4.005191	0.539941	3.164071
21	6	0	-5.200792	-0.119744	3.449271
22	6	0	-5.783519	-0.972255	2.508356
23	1	0	-1.585532	-3.978521	-2.698505
24	1	0	-5.539607	-4.534414	-1.142823
25	1	0	-3.616262	-5.354969	-2.449168
26	1	0	-3.562511	1.193925	3.907207
27	1	0	-5.683574	0.031839	4.410618
28	1	0	-6.712737	-1.474759	2.754546

29	8	0	-3.371684	-0.639792	-0.221650
30	6	0	-6.789857	-3.051041	0.636482
31	1	0	-6.314119	-3.759784	1.322858
32	1	0	-7.621927	-2.568605	1.157896
33	1	0	-7.224574	-3.612370	-0.195777
34	6	0	-6.529139	-1.039594	-0.848476
35	1	0	-7.349853	-0.533178	-0.327368
36	1	0	-5.850973	-0.274823	-1.241747
37	1	0	-6.943745	-1.598137	-1.695618
38	15	0	-0.813970	-1.291476	-1.522478
39	15	0	-1.749239	1.105114	1.461440
40	6	0	0.380743	-2.346345	-2.443053
41	6	0	1.338788	-3.058399	-1.707400
42	6	0	0.360789	-2.465481	-3.843773
43	6	0	2.256861	-3.883005	-2.363916
44	1	0	1.377113	-2.947510	-0.627164
45	6	0	1.284613	-3.282467	-4.495370
46	1	0	-0.374310	-1.917473	-4.425470
47	6	0	2.233751	-3.994385	-3.755105
48	1	0	3.000048	-4.424718	-1.788380
49	1	0	1.263635	-3.363107	-5.579033
50	1	0	2.956319	-4.627922	-4.262995
51	6	0	-1.151965	0.059220	-2.738847
52	6	0	-0.044651	0.757746	-3.254869
53	6	0	-2.440664	0.473899	-3.100133
54	6	0	-0.221748	1.842322	-4.111673
55	1	0	0.966579	0.451238	-3.007714
56	6	0	-2.616582	1.568517	-3.952487
57	1	0	-3.313391	-0.049591	-2.725245
58	6	0	-1.512119	2.256129	-4.459294
59	1	0	0.657320	2.348715	-4.499092
60	1	0	-3.622903	1.880450	-4.219863
61	1	0	-1.654923	3.105437	-5.122319
62	6	0	-1.170586	1.835444	3.048820

63	6	0	-0.876784	3.198668	3.189531
64	6	0	-0.867413	0.959806	4.107529
65	6	0	-0.293022	3.677188	4.367203
66	1	0	-1.094316	3.891522	2.382805
67	6	0	-0.305601	1.442698	5.287885
68	1	0	-1.063901	-0.104427	4.003058
69	6	0	-0.010113	2.804486	5.419344
70	1	0	-0.063890	4.735688	4.459543
71	1	0	-0.083740	0.754699	6.099364
72	1	0	0.440533	3.179599	6.334279
73	6	0	-2.265242	2.564327	0.456028
74	6	0	-1.458486	2.978421	-0.614031
75	6	0	-3.430013	3.289922	0.755885
76	6	0	-1.802062	4.105126	-1.364384
77	1	0	-0.571754	2.408578	-0.871410
78	6	0	-3.774930	4.411616	0.000883
79	1	0	-4.068384	2.978383	1.577933
80	6	0	-2.959711	4.823120	-1.058621
81	1	0	-1.169294	4.407852	-2.192960
82	1	0	-4.679782	4.964644	0.239397
83	1	0	-3.230914	5.696503	-1.646062
84	46	0	0.008521	-0.064706	0.386750
85	6	0	3.803537	3.659454	-0.814555
86	6	0	3.117628	3.518366	-2.020755
87	6	0	2.115697	4.403483	-2.393202
88	6	0	1.816106	5.452013	-1.508907
89	6	0	2.506790	5.594052	-0.297509
90	6	0	3.517433	4.690847	0.067939
91	6	0	4.732792	2.482525	-0.699476
92	6	0	3.609929	2.246262	-2.657154
93	1	0	1.571532	4.279595	-3.324465
94	1	0	1.032318	6.160800	-1.761625
95	1	0	2.248579	6.411574	0.370287
96	1	0	4.044887	4.783834	1.012935

97	7	0	4.601431	1.705511	-1.846555
98	8	0	5.450083	2.215419	0.259532
99	8	0	3.165503	1.733569	-3.681864
100	6	0	3.737764	-0.743291	-1.160447
101	1	0	3.128778	-0.790711	-2.065074
102	1	0	4.398186	0.401935	-1.535904
103	1	0	4.422268	-1.584935	-1.062981
104	1	0	1.700557	1.673574	3.186331
105	6	0	3.013805	-2.161024	1.641733
106	8	0	1.821316	-2.464657	1.654087
107	6	0	5.080287	-0.863952	1.514494
108	6	0	5.358654	-2.375399	1.519856
109	7	0	4.069149	-2.983980	1.869583
110	1	0	5.695151	-2.739862	0.540849
111	1	0	5.610068	-0.332094	0.724489
112	1	0	5.402638	-0.429707	2.464198
113	1	0	6.122271	-2.635011	2.254359
114	6	0	3.909046	-4.440348	2.177159
115	6	0	2.989936	-4.599851	3.401655
116	6	0	3.322530	-5.158867	0.951010
117	6	0	5.277816	-5.052117	2.516100
118	1	0	3.400240	-4.052882	4.258684
119	1	0	1.985521	-4.225605	3.198955
120	1	0	2.920863	-5.659606	3.671683
121	1	0	4.001911	-5.067725	0.096195
122	1	0	3.189074	-6.225380	1.165661
123	1	0	2.351892	-4.737433	0.680543
124	1	0	5.136667	-6.115009	2.738243
125	1	0	5.984265	-4.980430	1.682477
126	1	0	5.726507	-4.583528	3.398943

----

11 Reference

1. Kachkovskyi, G.; Faderl, C.; Reiser, O., *Adv. Syn. Catal*. **2013**, *355* 11-12, 2240-2248.

2. Faderl, C.; Budde, S.; Kachkovskyi, G.; Rackl, D.; Reiser, O., *J. Org. Chem.* **2018**, *83* 19, 12192-12206.

3. Huang, H.-M.; Koy, M.; Serrano, E.; Pflüger, P. M.; Schwarz, J. L.; Glorius, F., *Nat. Catal.* **2020**, *3* 4, 393-400.

4. Frisch, M. J. *et al. Gaussian 09, Revision D.01* (Gaussian, Inc.: Wallingford, CT, 2009).

5. Hohenberg, P.; Kohn, W. *Phys. Rev.***1964**, 136(3B), 864

6. Becke, A. D. *J. Chem. Phys.* **1993,** 98, 5648

7. Grimme, S., Ehrlich, S., Goerigk, L. *J. Comput. Chem.* **2011**, 32, 1456

8. Hay, P. J.; Wadt, W. R. *J. Chem. Phys.***1985**, 82(1), 299–310

9. Marenich, A. V.; Cramer, C. J.; Truhlar, D. G. *J. Phys. Chem. B* **2009**, 113, 6378

10. Zhao, Y.; Truhlar, D. G. *Acc. Chem. Res.* **2008**, 41, 157

12. NMR spectra of the starting materials and products

^{1}H NMR (400 MHz, $CDCl_3$) of **1a**

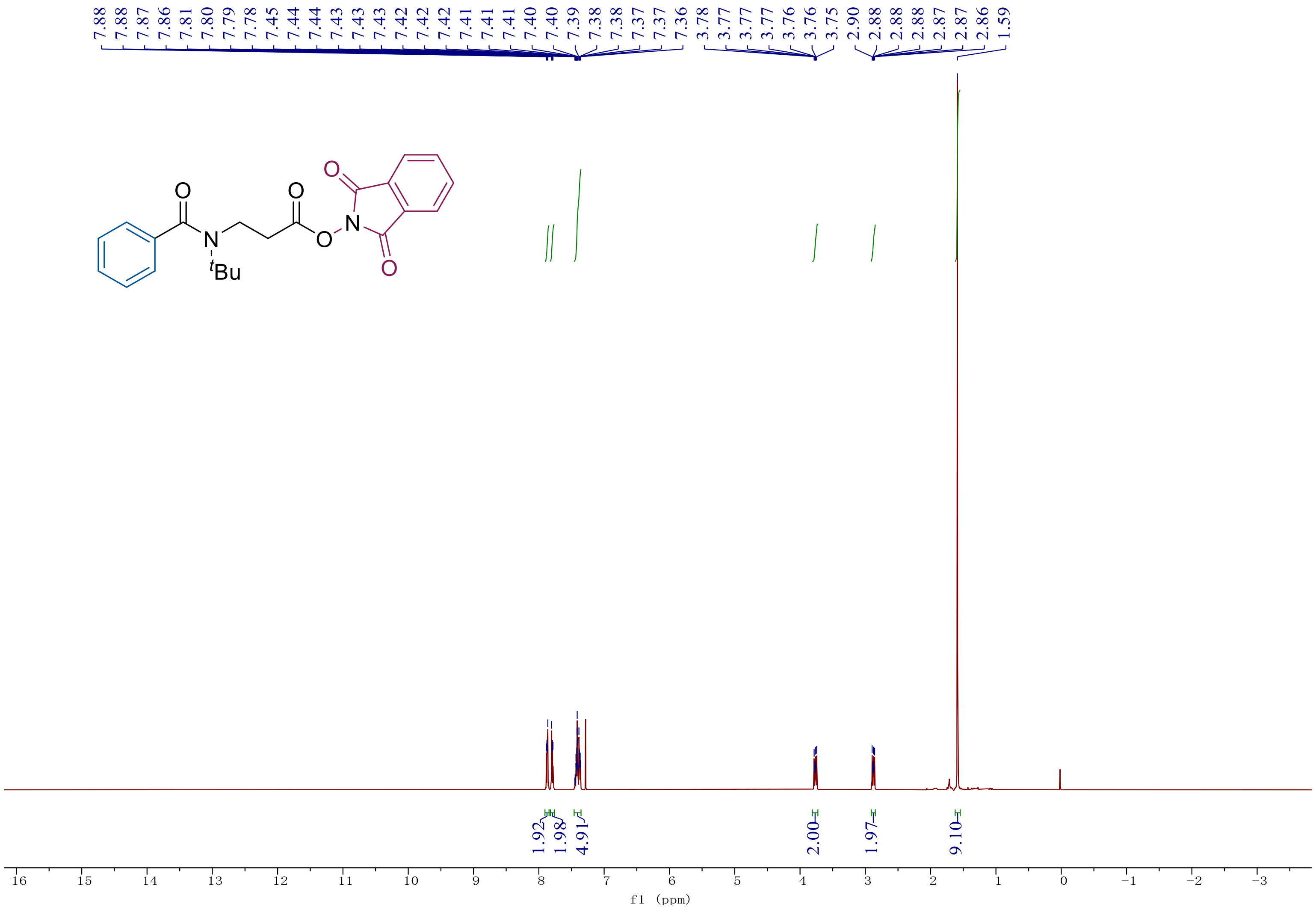

^{13}C NMR (101 MHz, $CDCl_3$) of **1a**

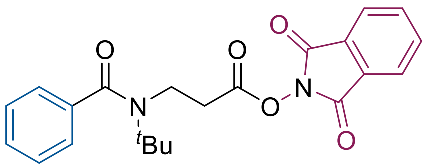

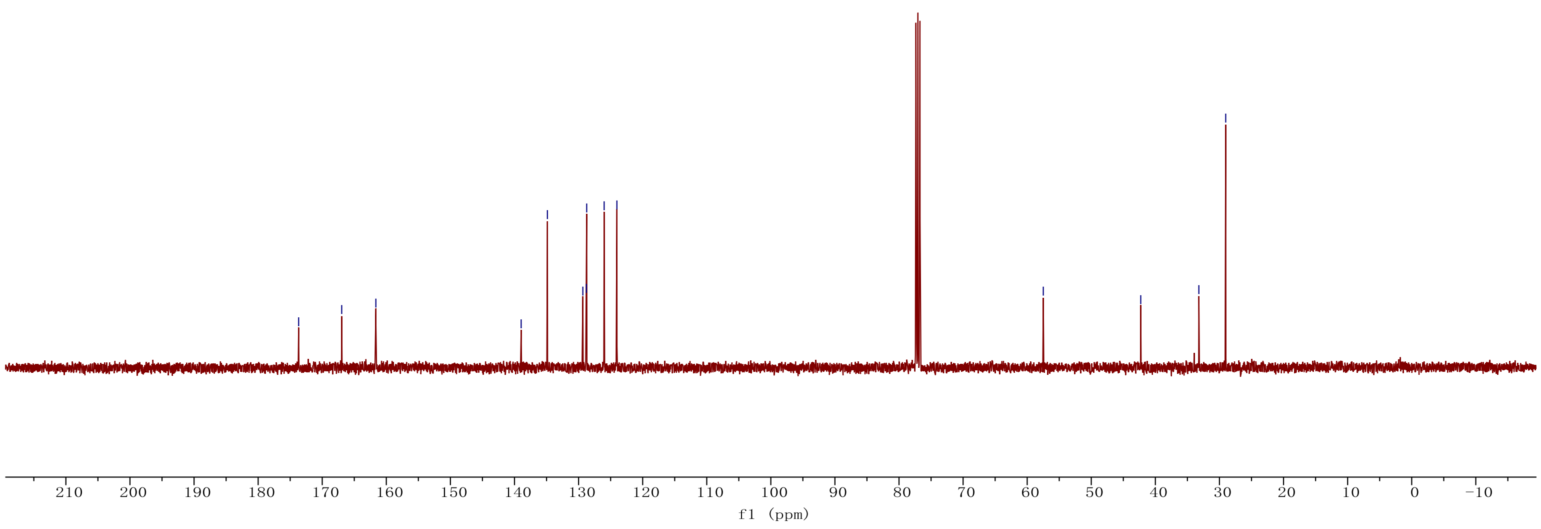

^{1}H NMR (400 MHz, $CDCl_3$) of **1b**

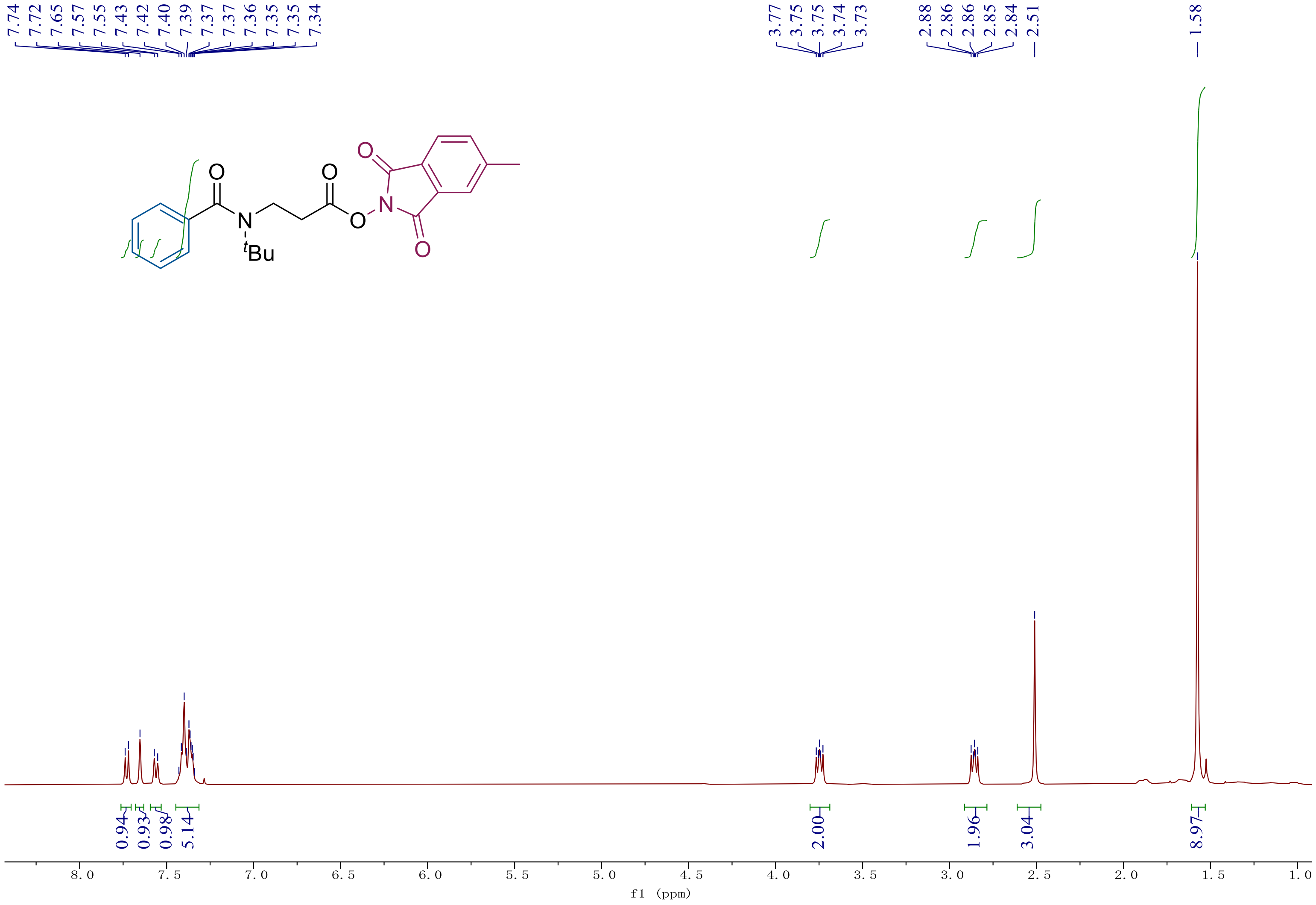

^{13}C NMR (101 MHz, $CDCl_3$) of **1b**

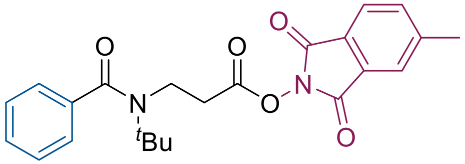

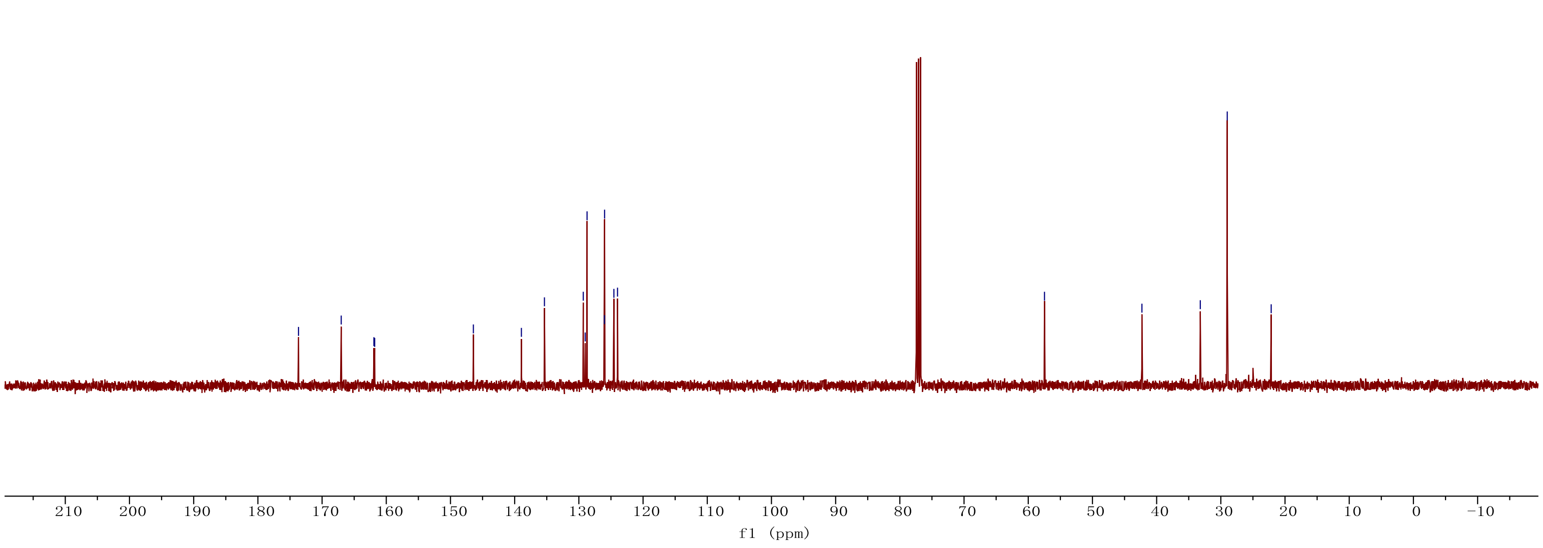

^{1}H NMR (400 MHz, $CDCl_3$) of **1c**

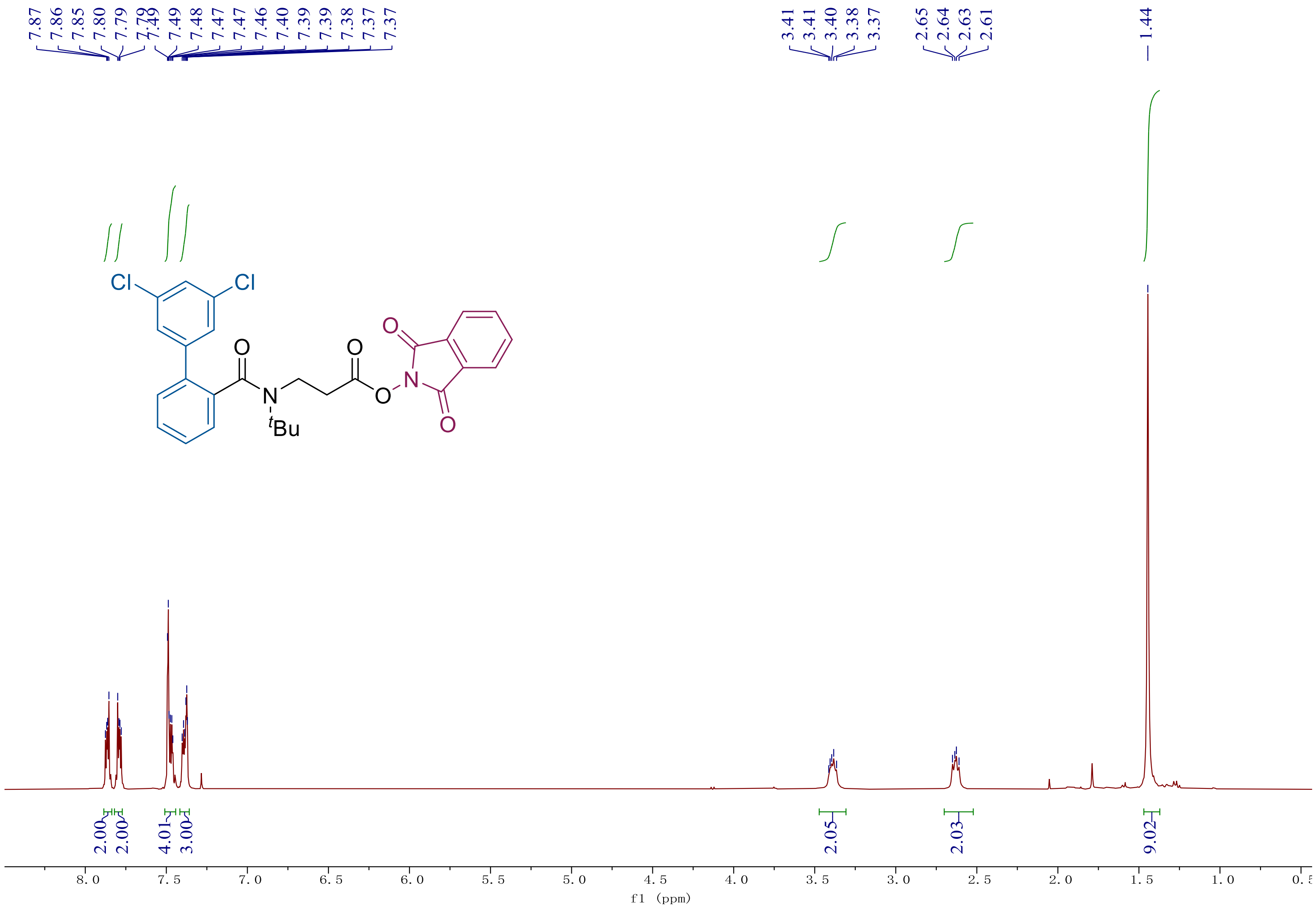

^{13}C NMR (101 MHz, $CDCl_3$) of **1c**

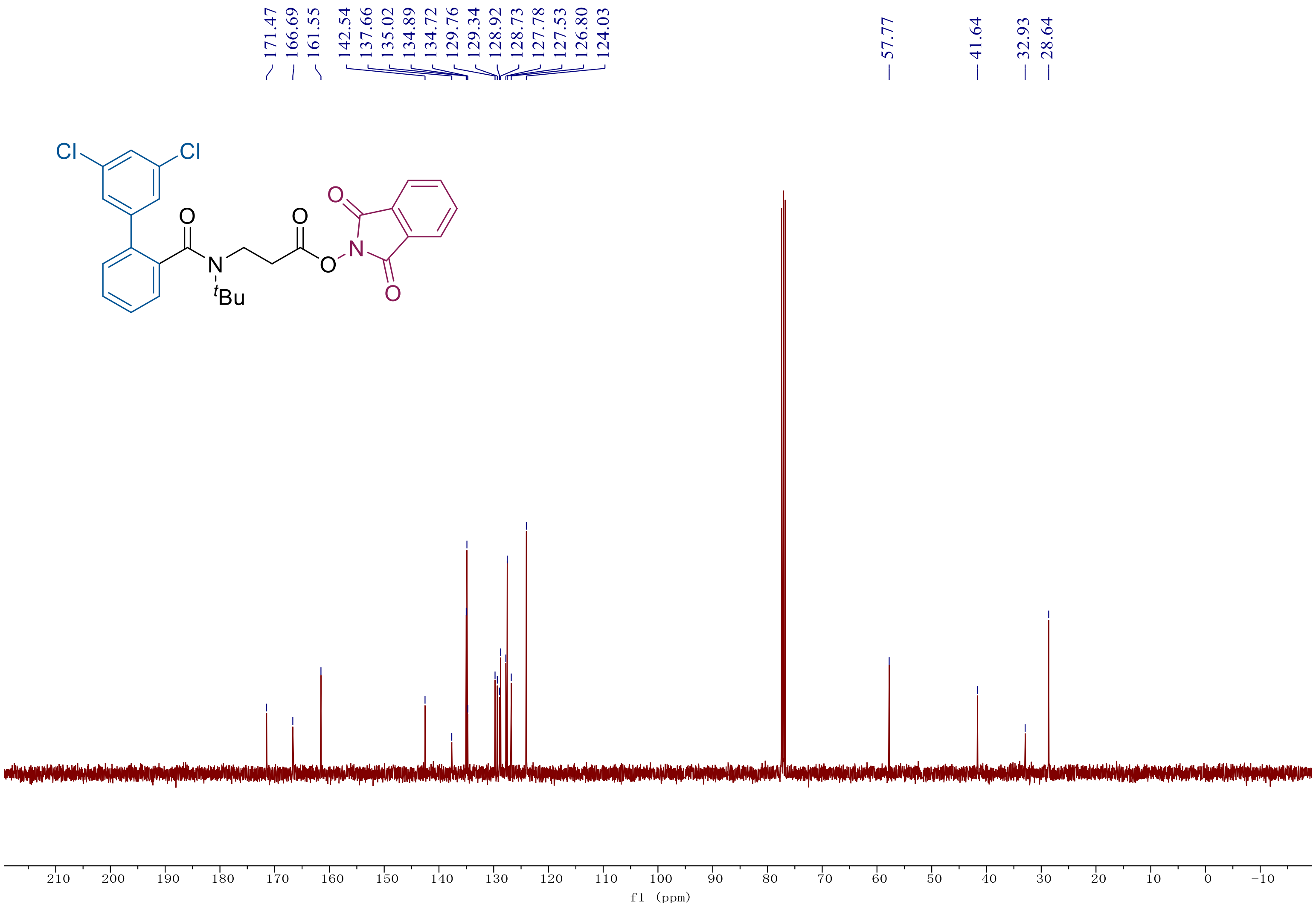

^{1}H NMR (400 MHz, $CDCl_3$) of **1e**

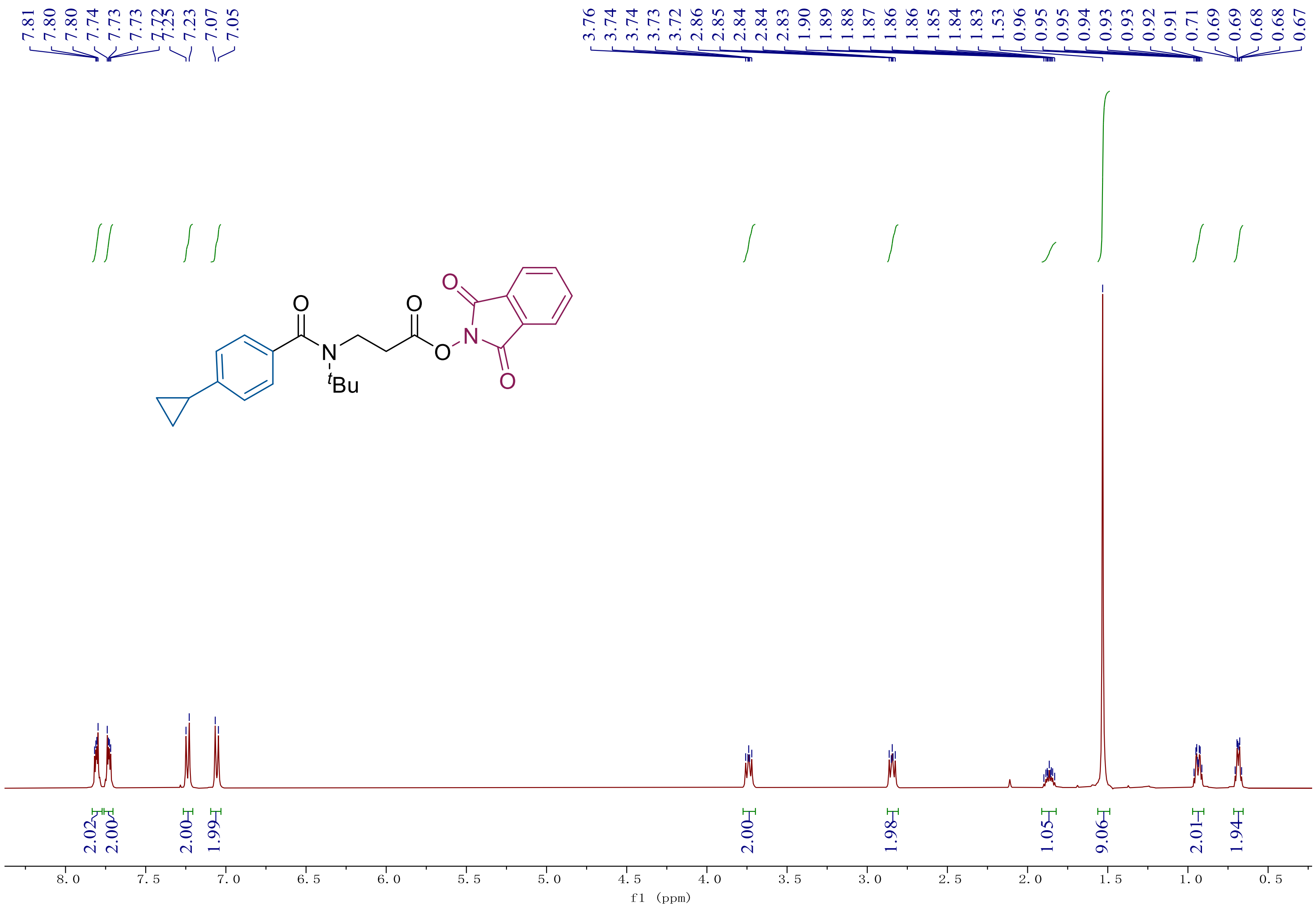

^{13}C NMR (101 MHz, $CDCl_3$) of **1e**

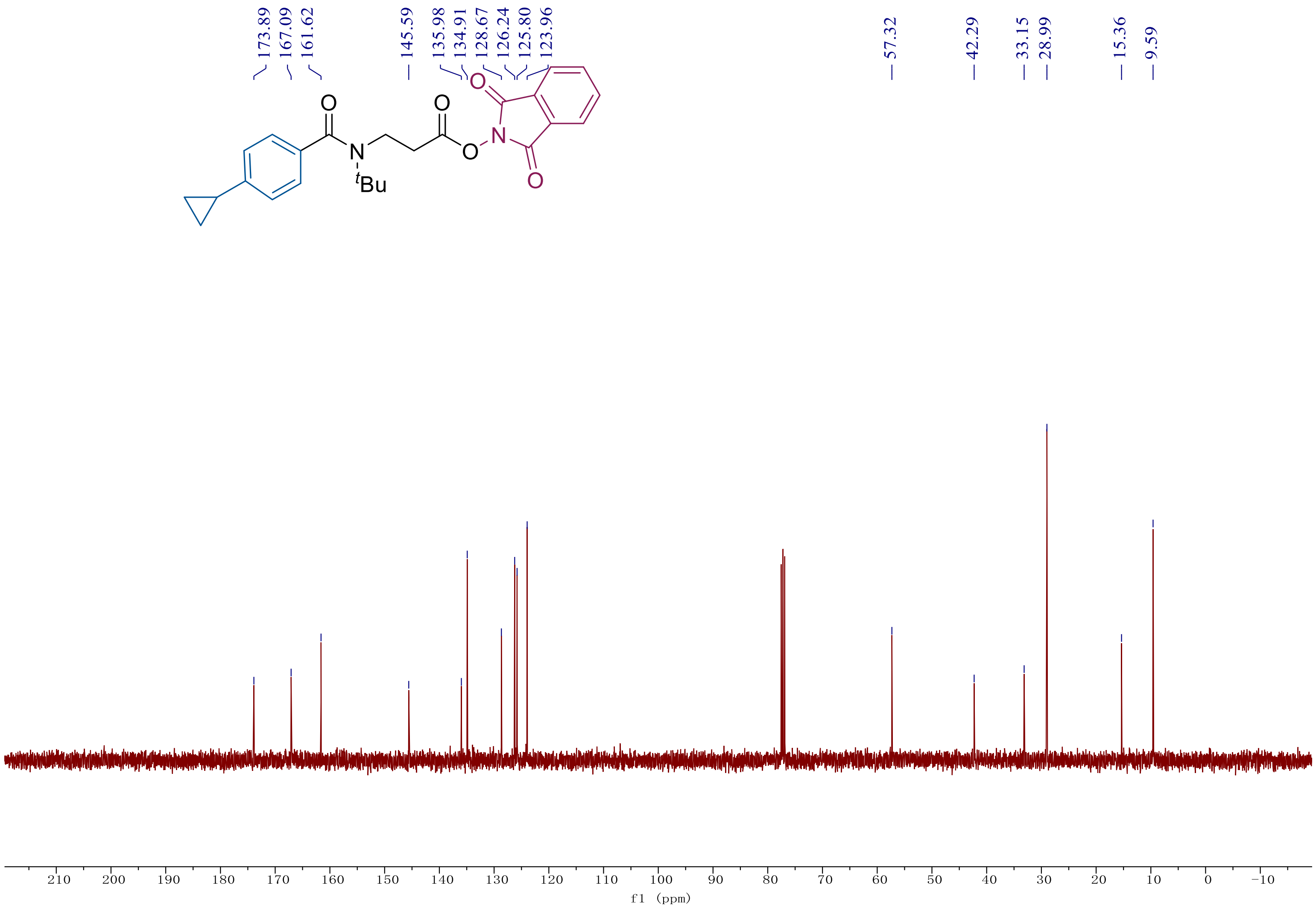

^{1}H NMR (400 MHz, $CDCl_3$) of **1f**

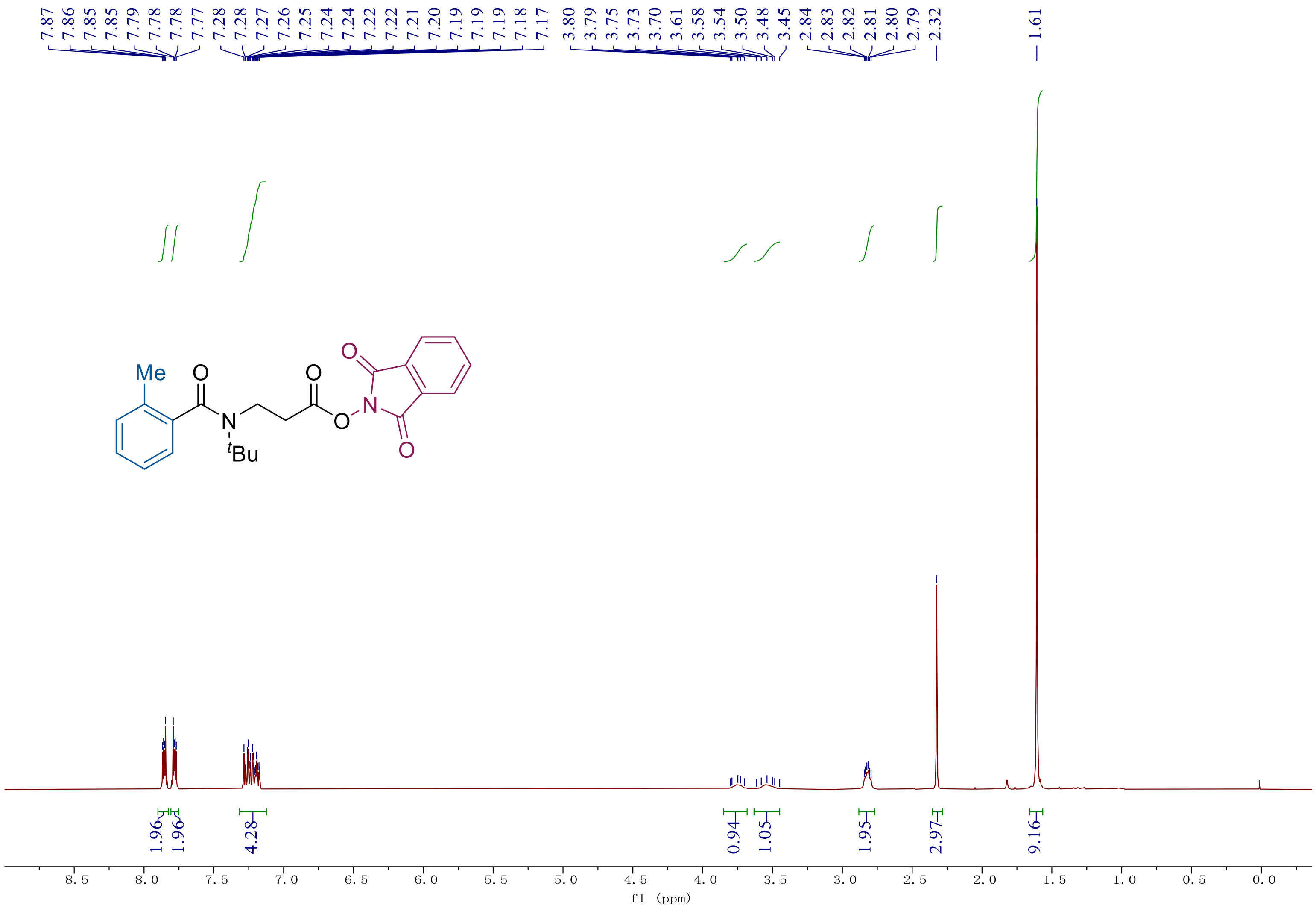

^{13}C NMR (101 MHz, CDCl$_3$) of **1f**

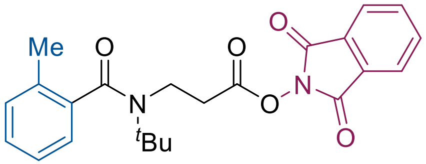

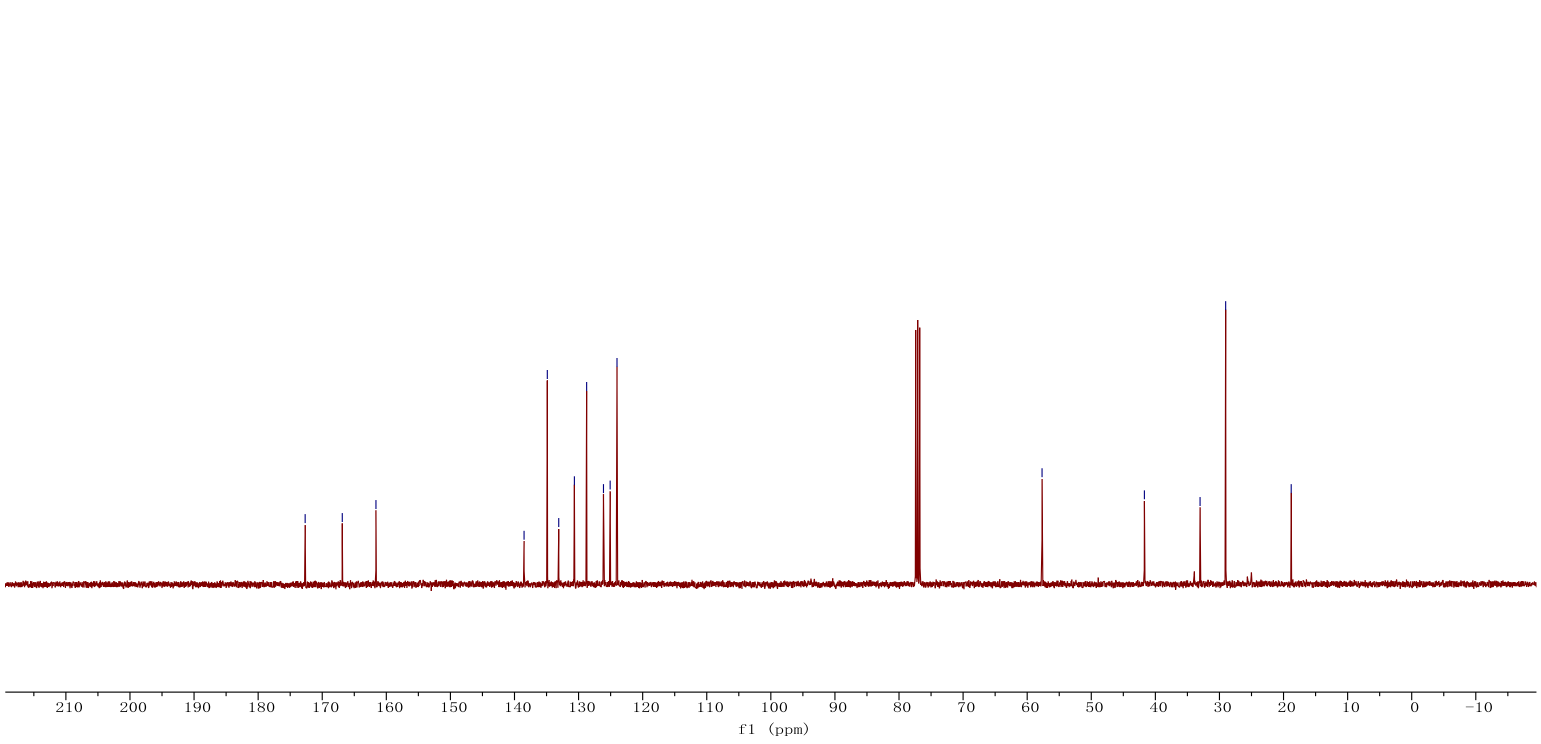

^{1}H NMR (400 MHz, $CDCl_3$) of **1g**

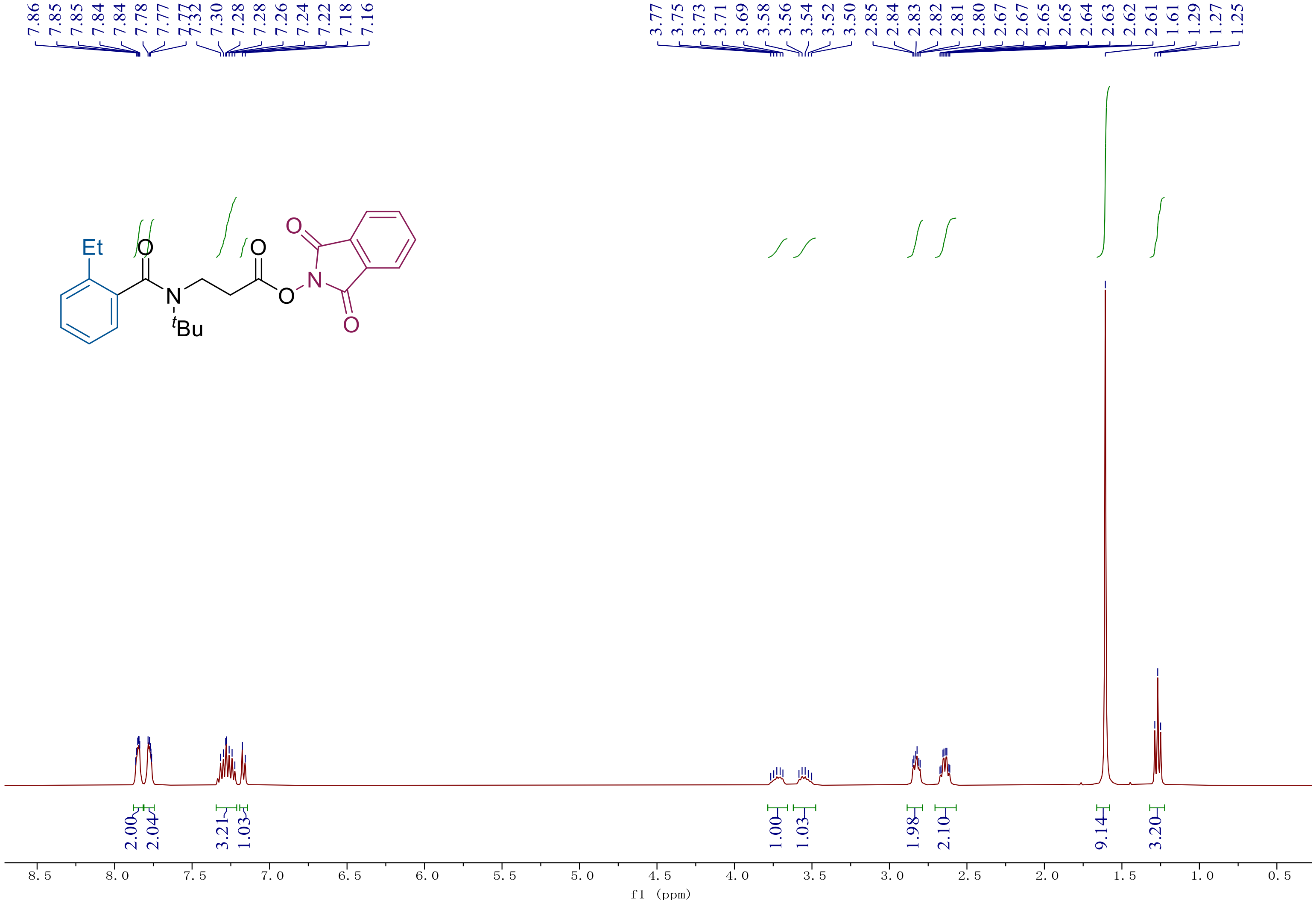

^{13}C NMR (101 MHz, CDCl$_3$) of **1g**

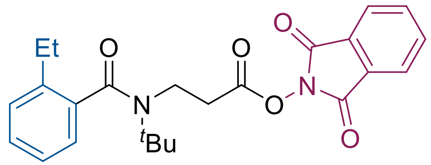

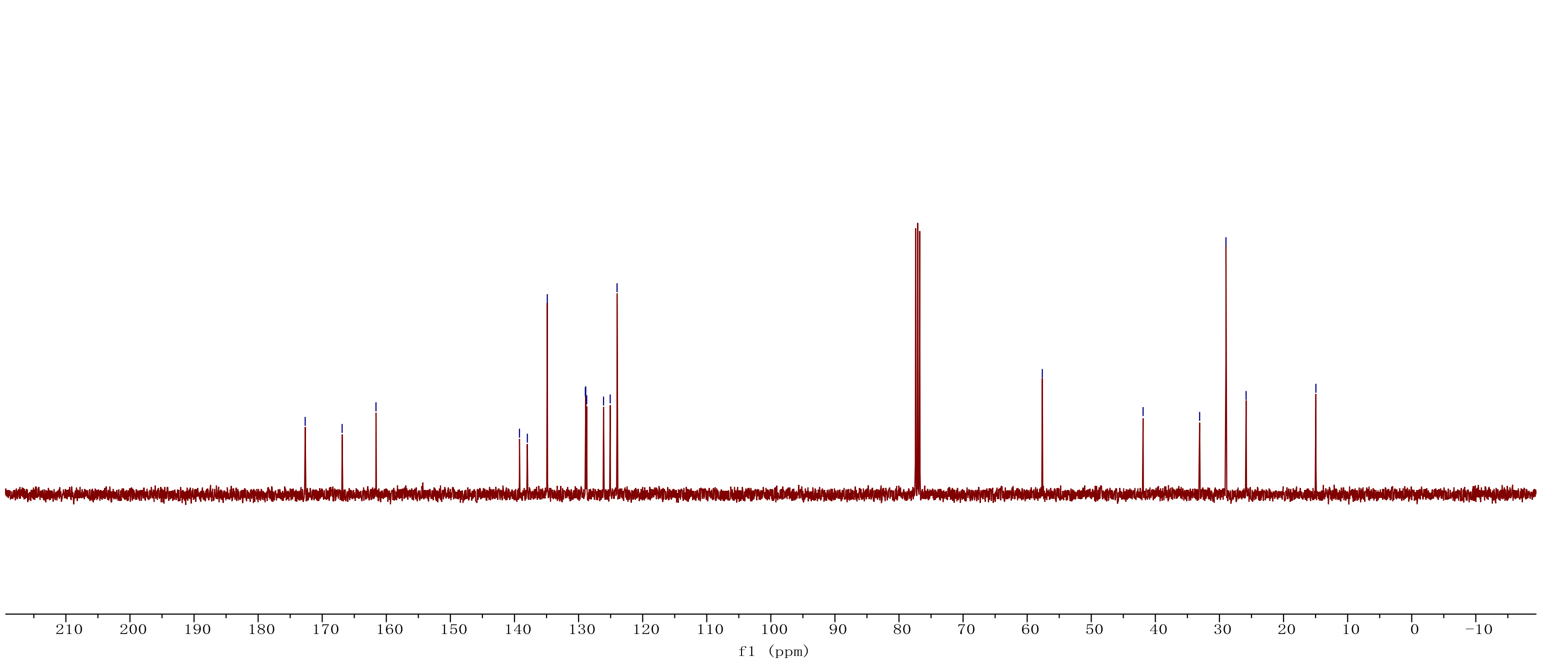

^{1}H NMR (400 MHz, $CDCl_3$) of **1h**

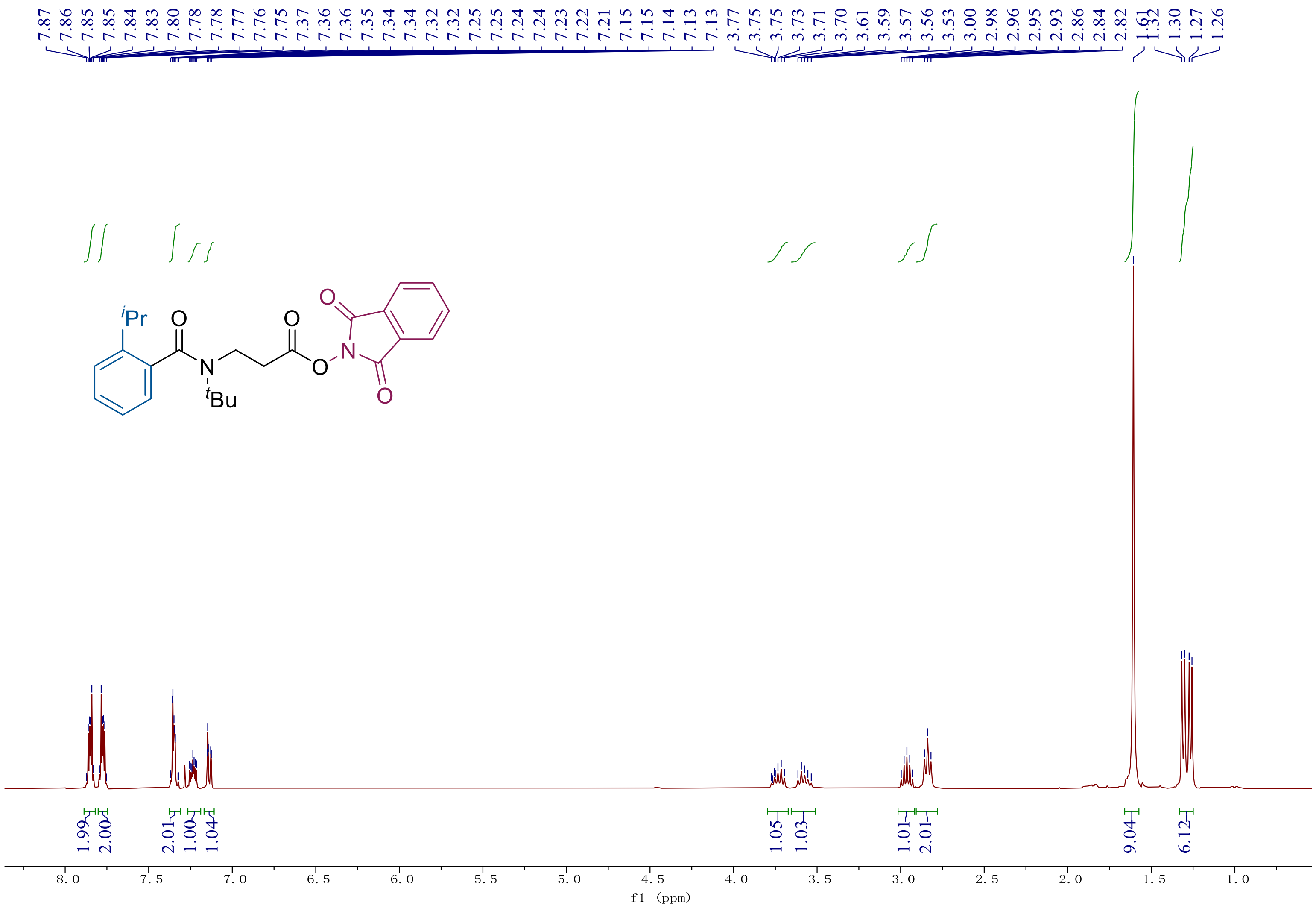

^{13}C NMR (126 MHz, $CDCl_3$) of **1h**

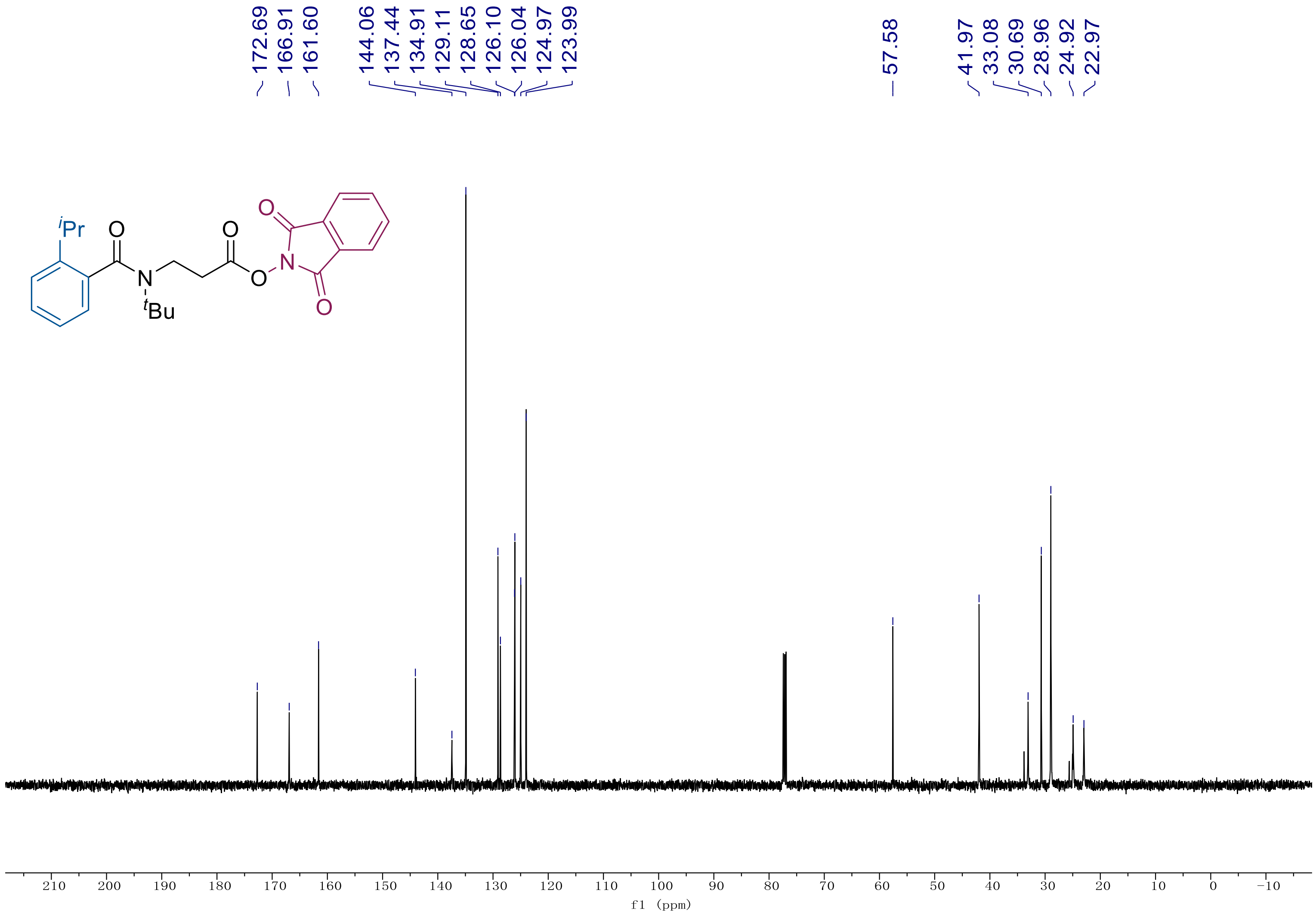

^{1}H NMR (400 MHz, $CDCl_3$) of **1i**

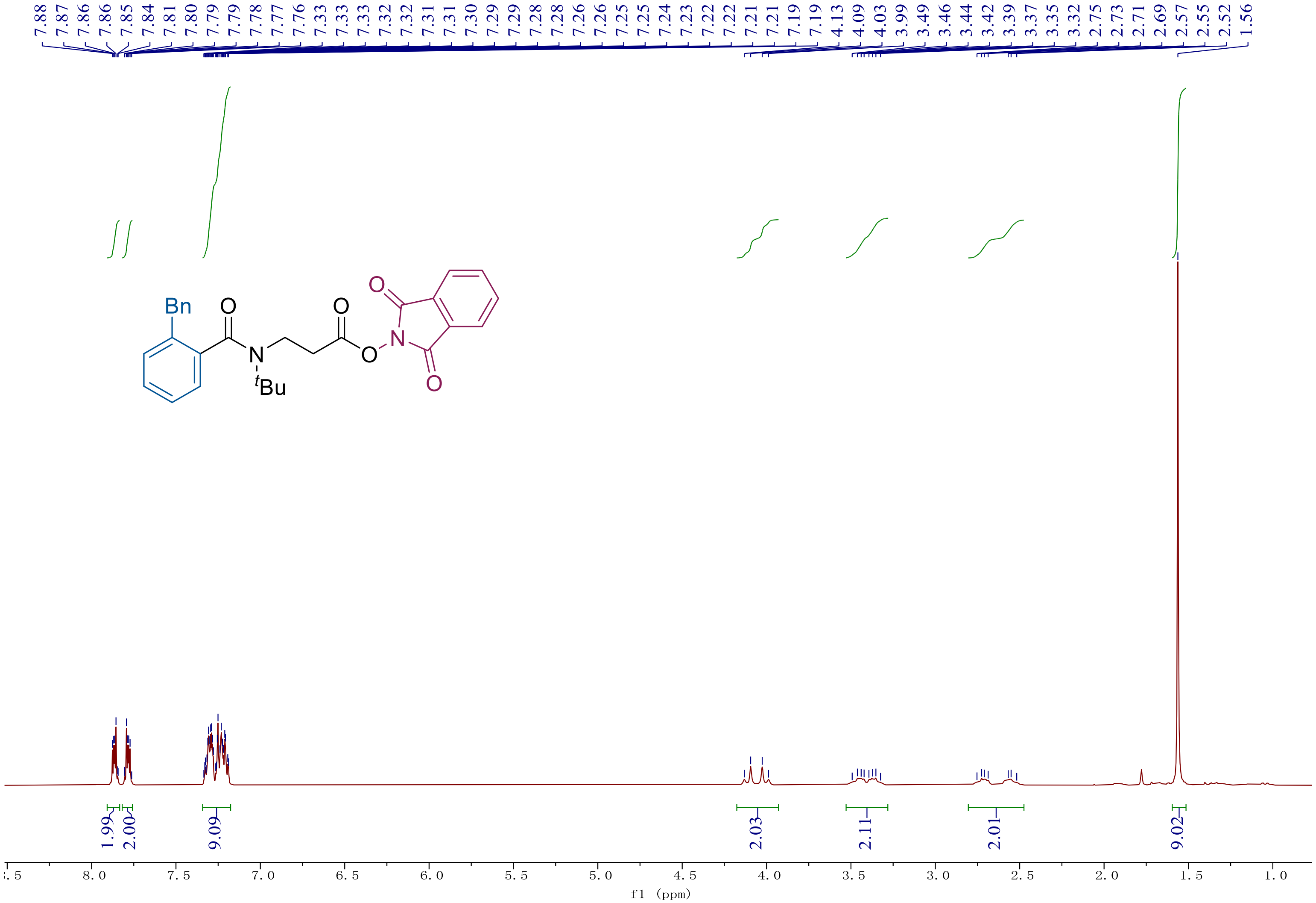

^{13}C NMR (101 MHz, $CDCl_3$) of **1i**

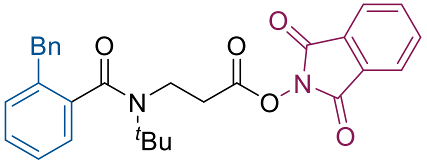

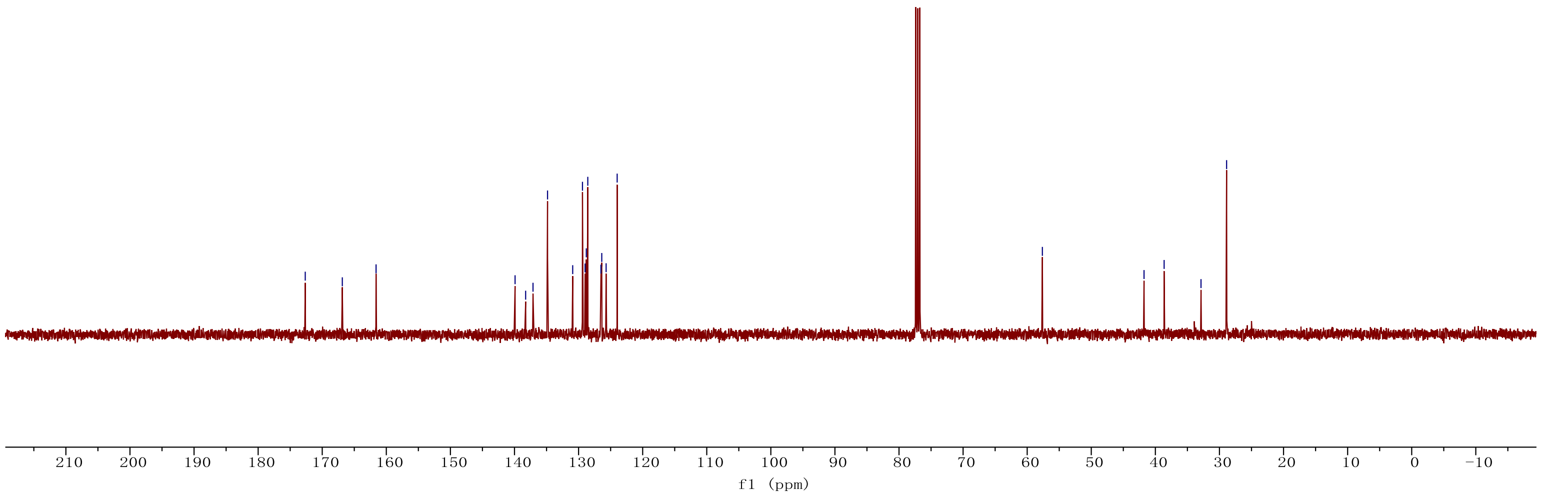

^{1}H NMR (500 MHz, CDCl3) of **1j**

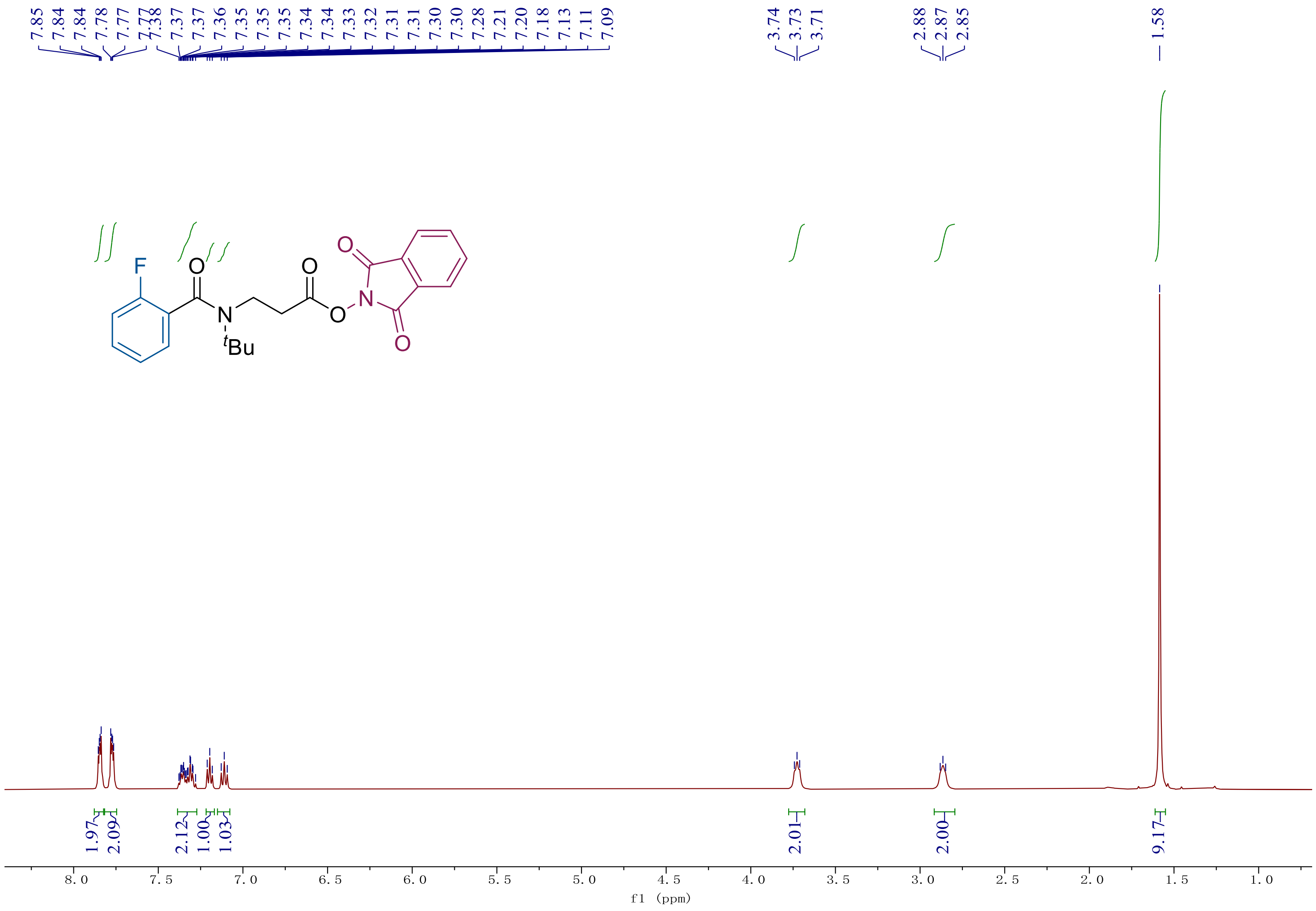

^{13}C NMR (126 MHz, $CDCl_3$) of **1j**

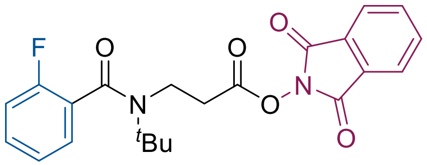

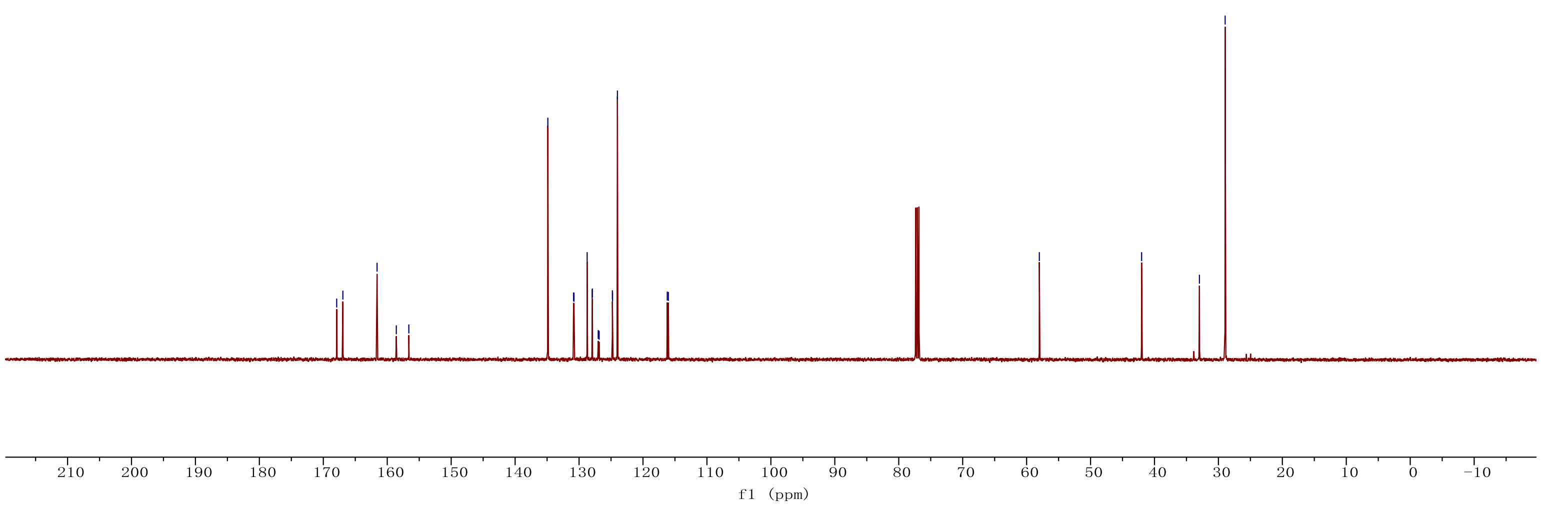

^{19}F NMR (471 MHz, $CDCl_3$) of **1j**

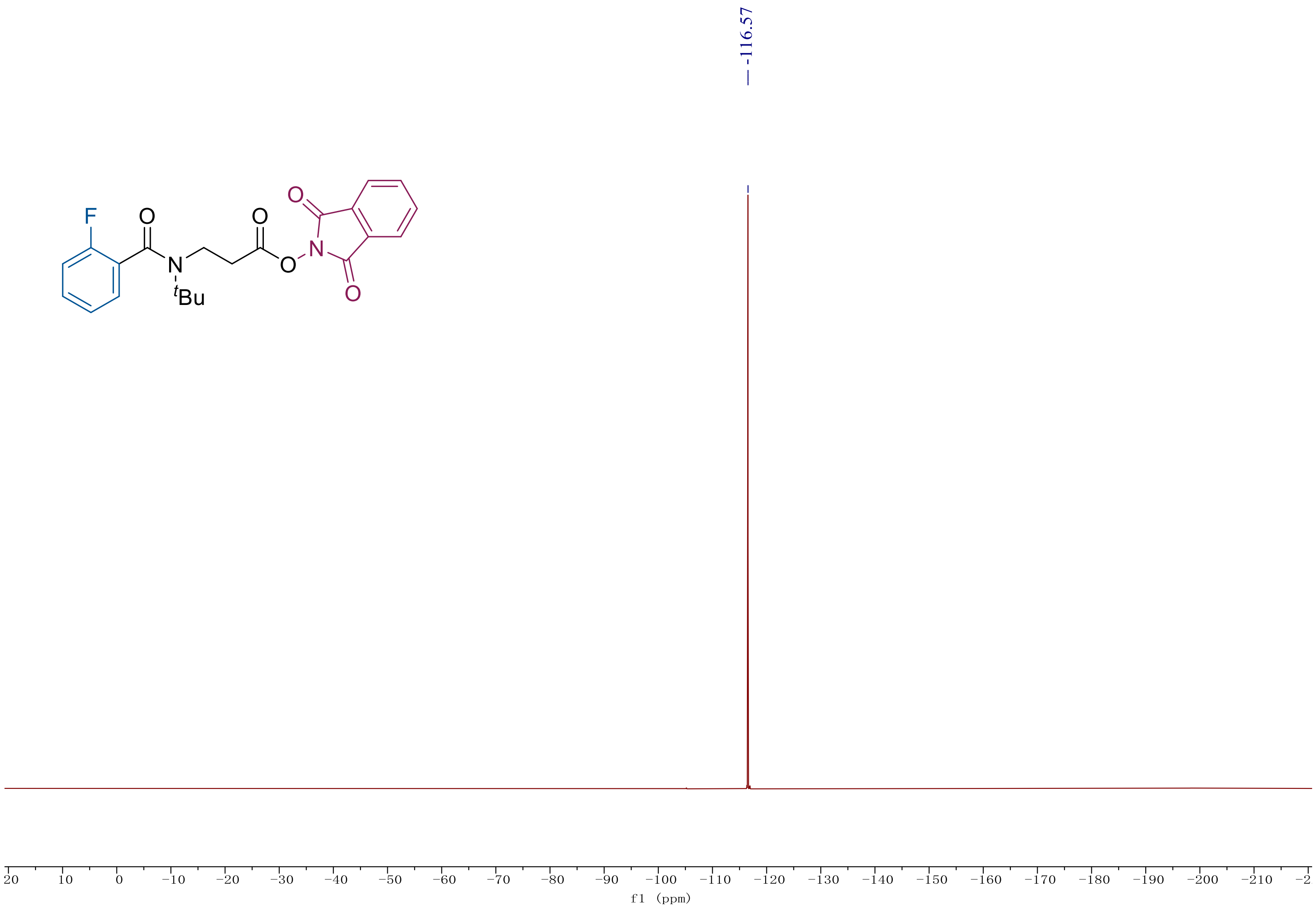

^{1}H NMR (400 MHz, $CDCl_3$) of **1k**

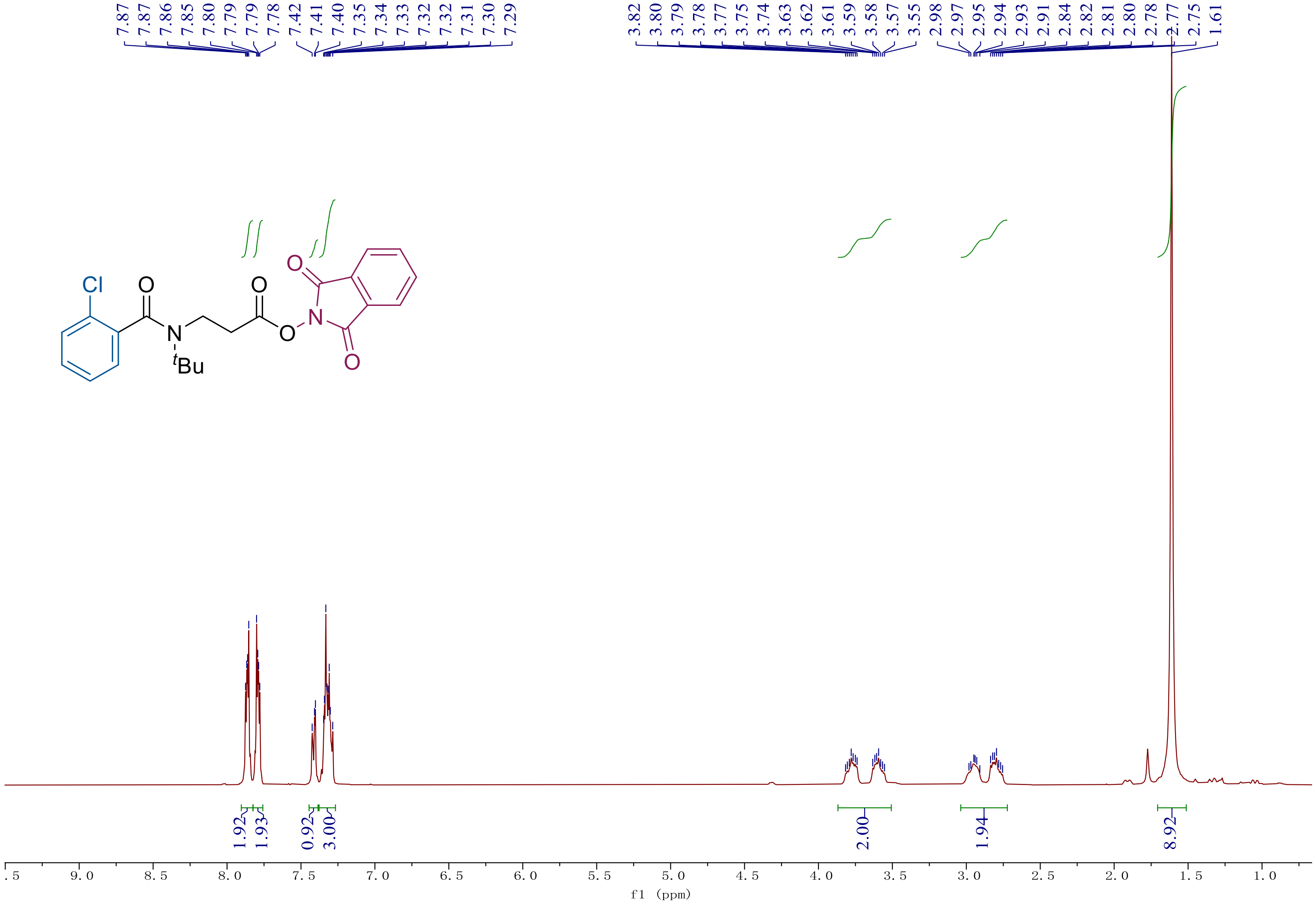

^{13}C NMR (101 MHz, CDCl$_3$) of **1k**

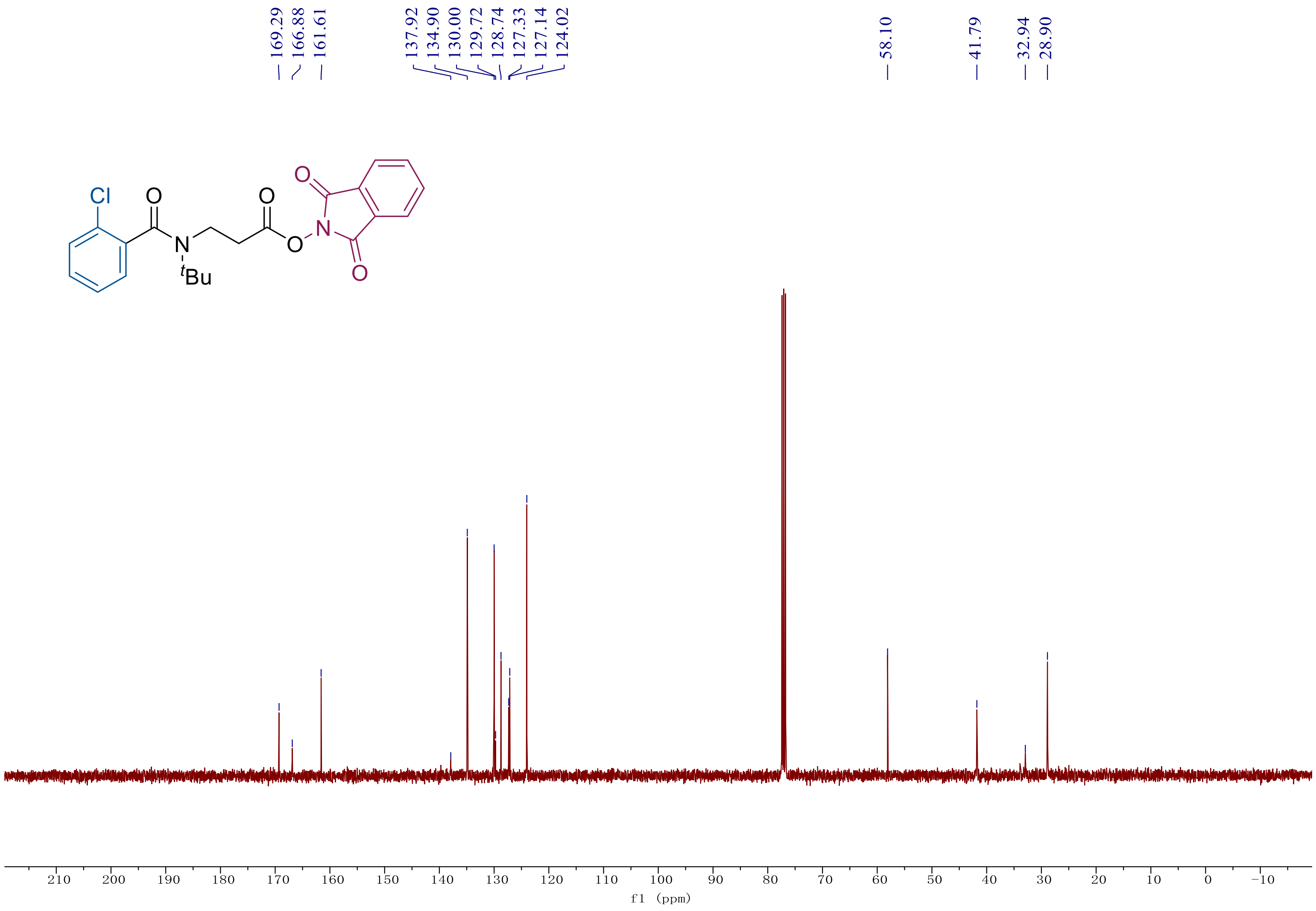

^{1}H NMR (500 MHz, $CDCl_3$) of **1l**

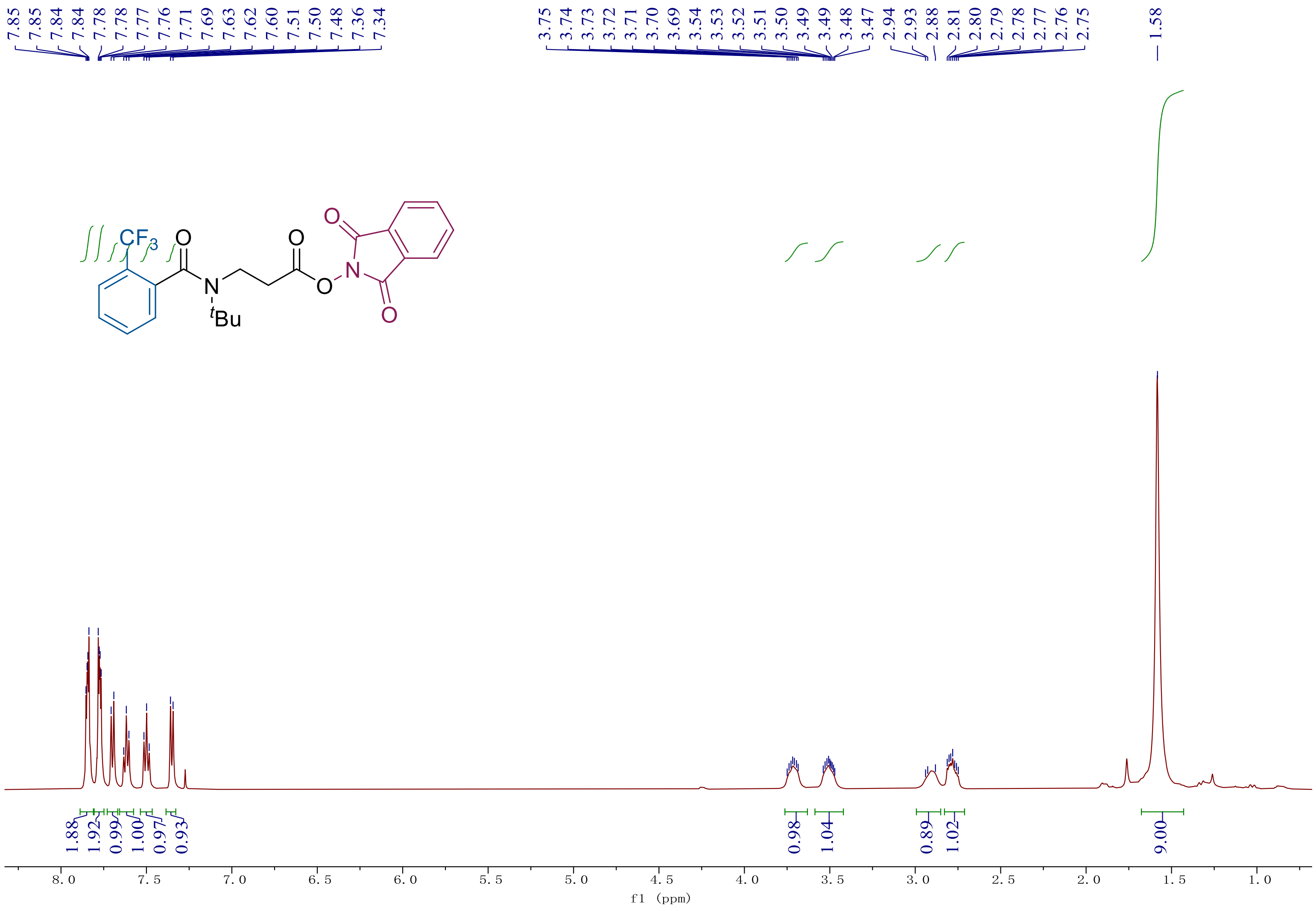

^{13}C NMR (126 MHz, CDCl₃) of **1l**

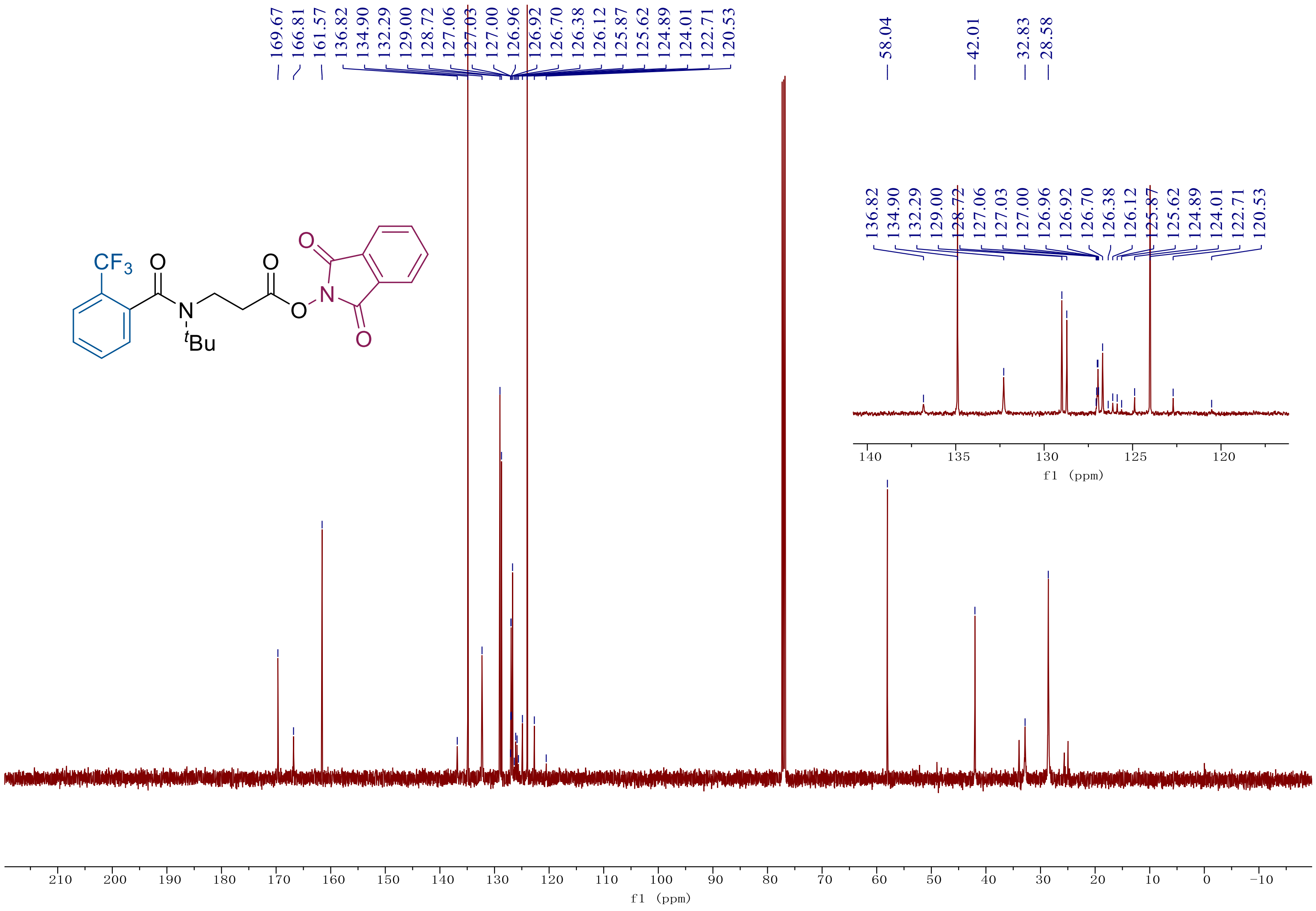

^{19}F NMR (471 MHz, $CDCl_3$) of **1l**

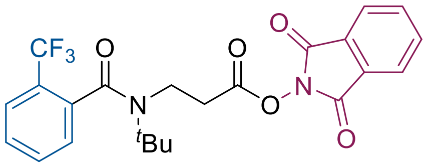

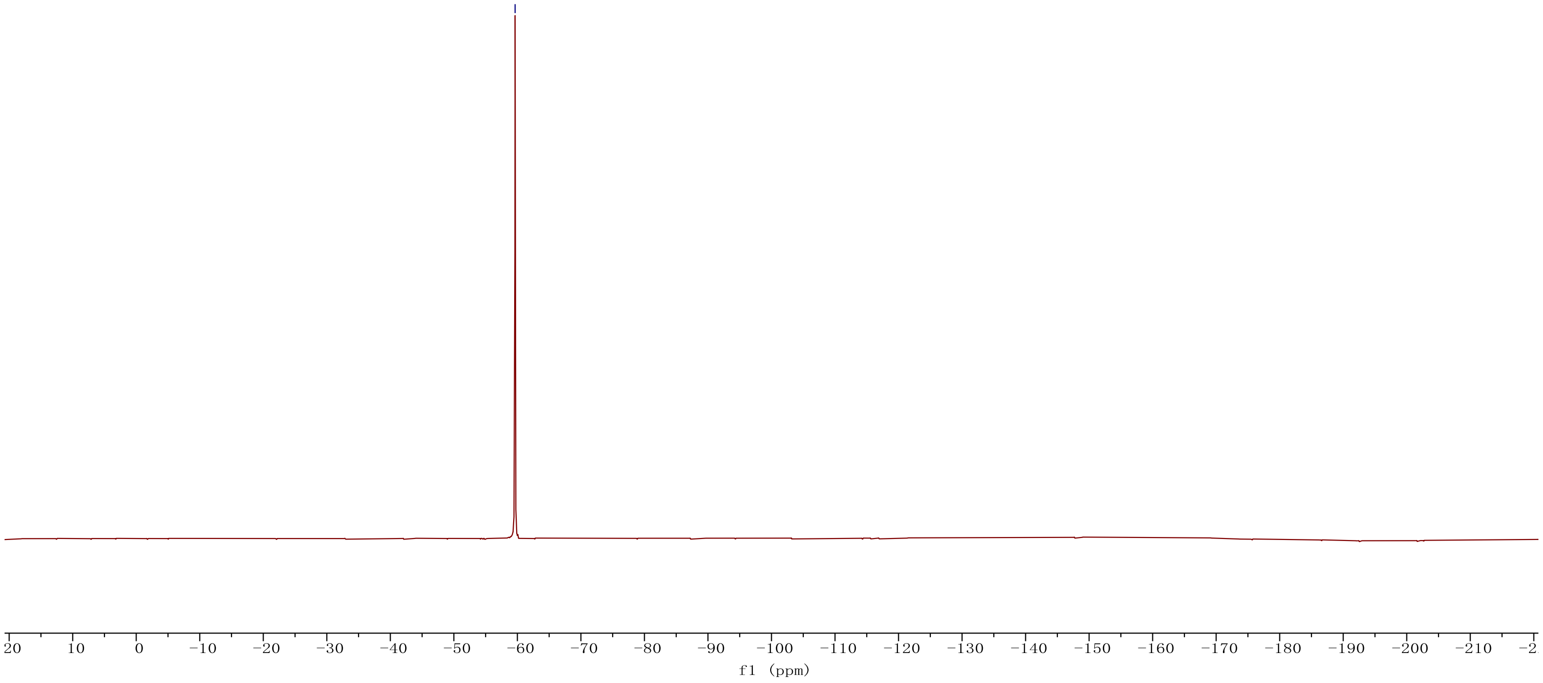

^{1}H NMR (500 MHz, $CDCl_3$) of **1m**

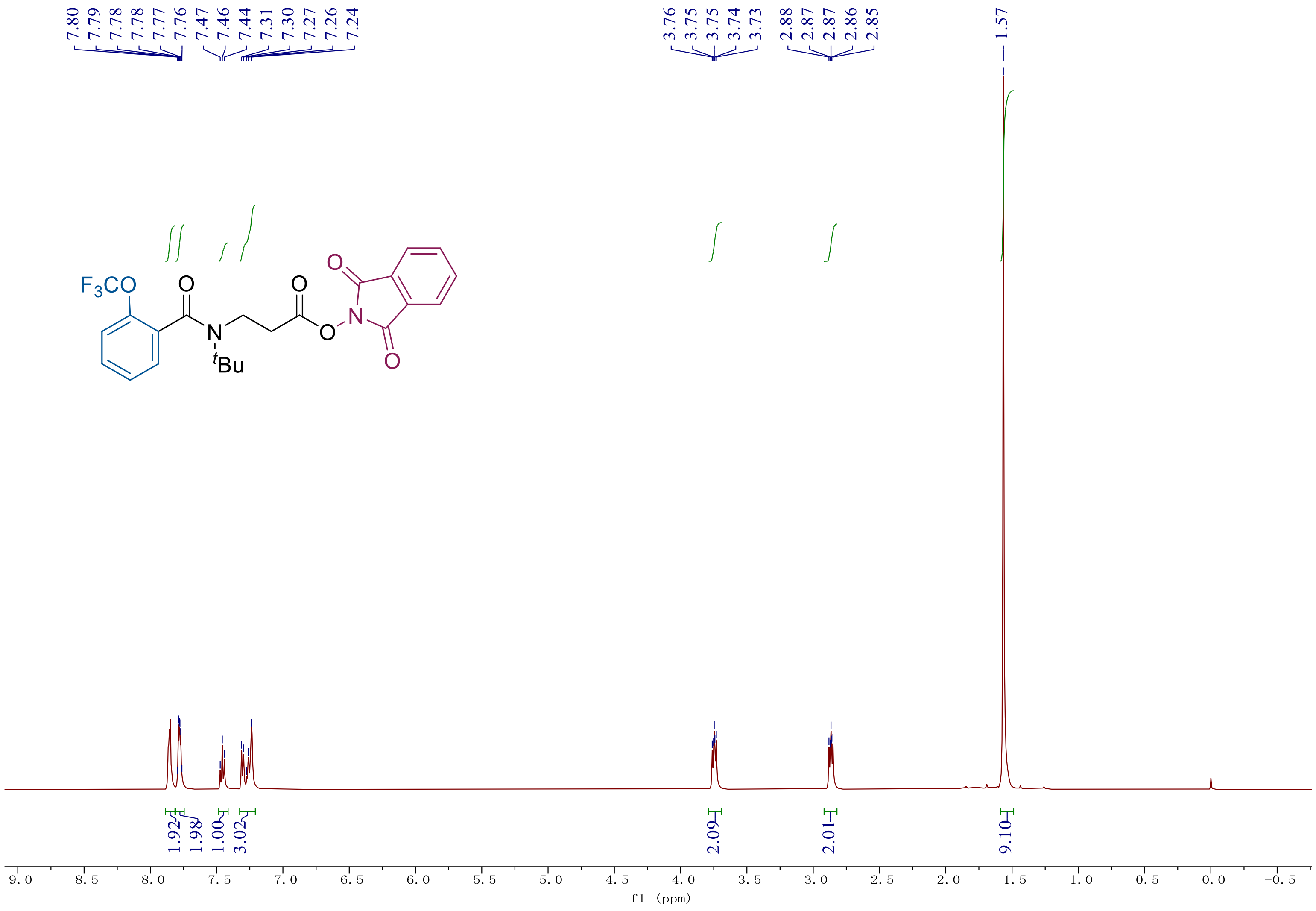

^{13}C NMR (126 MHz, $CDCl_3$) of **1m**

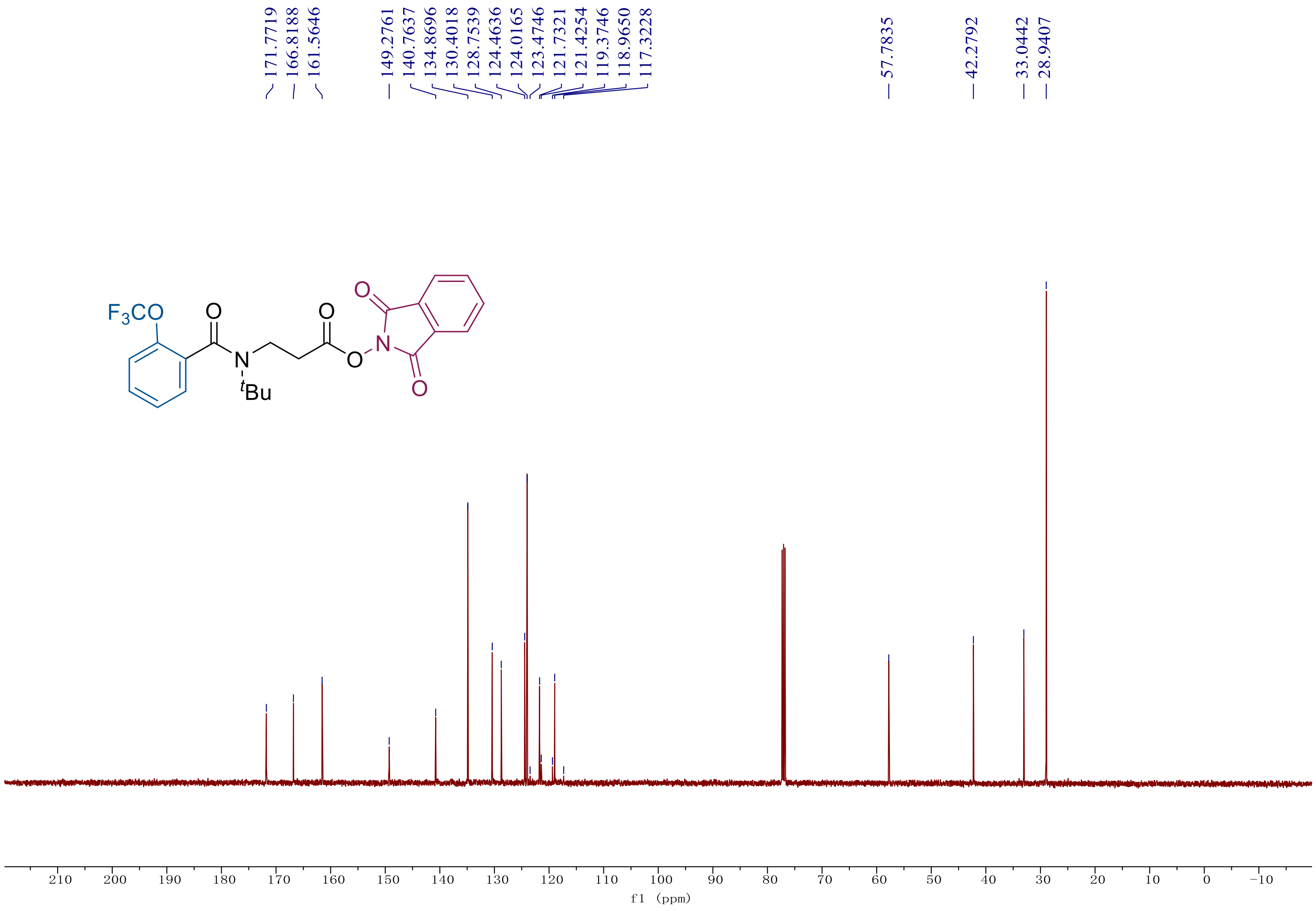

^{19}F NMR (471 MHz, $CDCl_3$) of **1m**

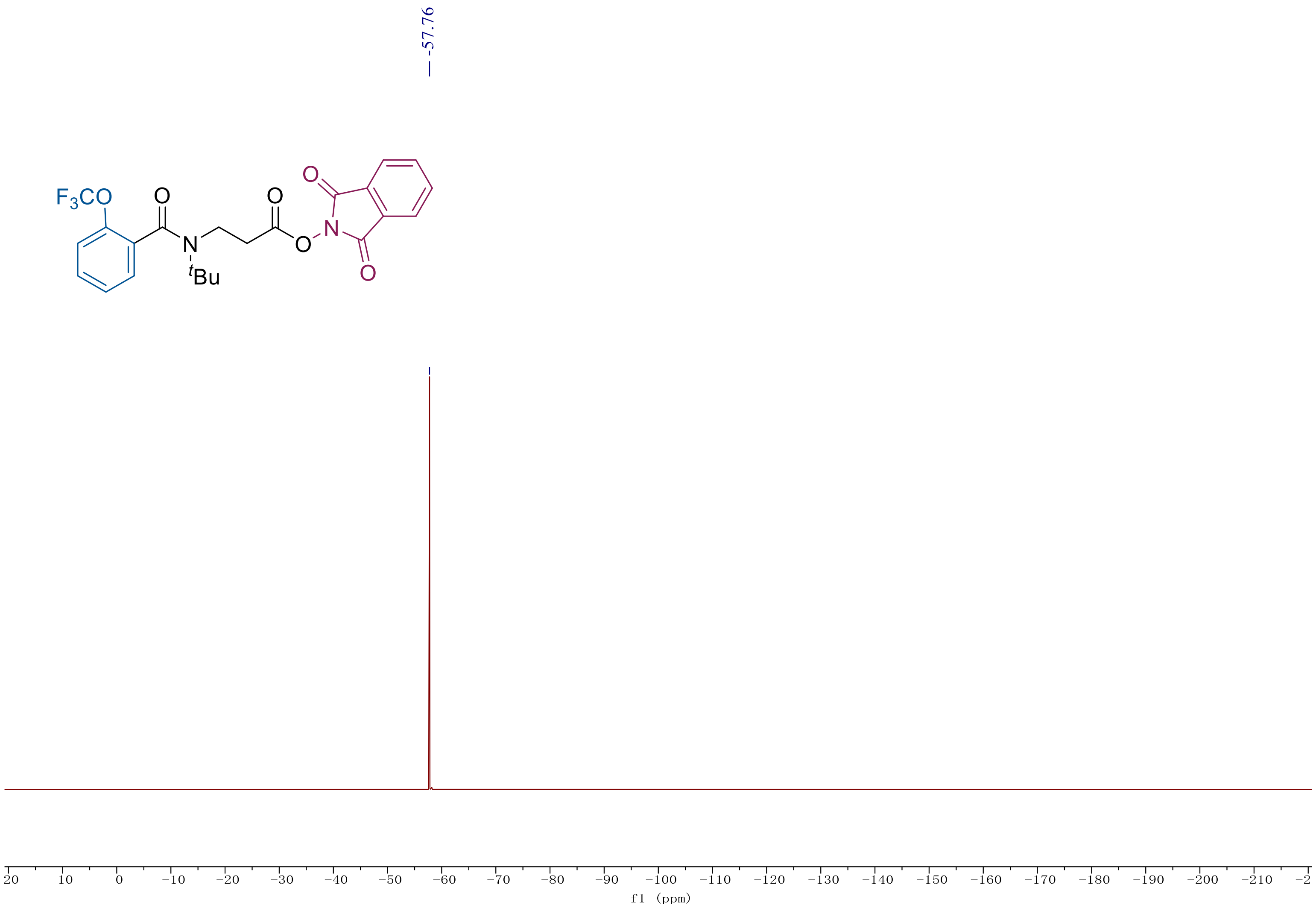

^{1}H NMR (400 MHz, $CDCl_3$) of **1n**

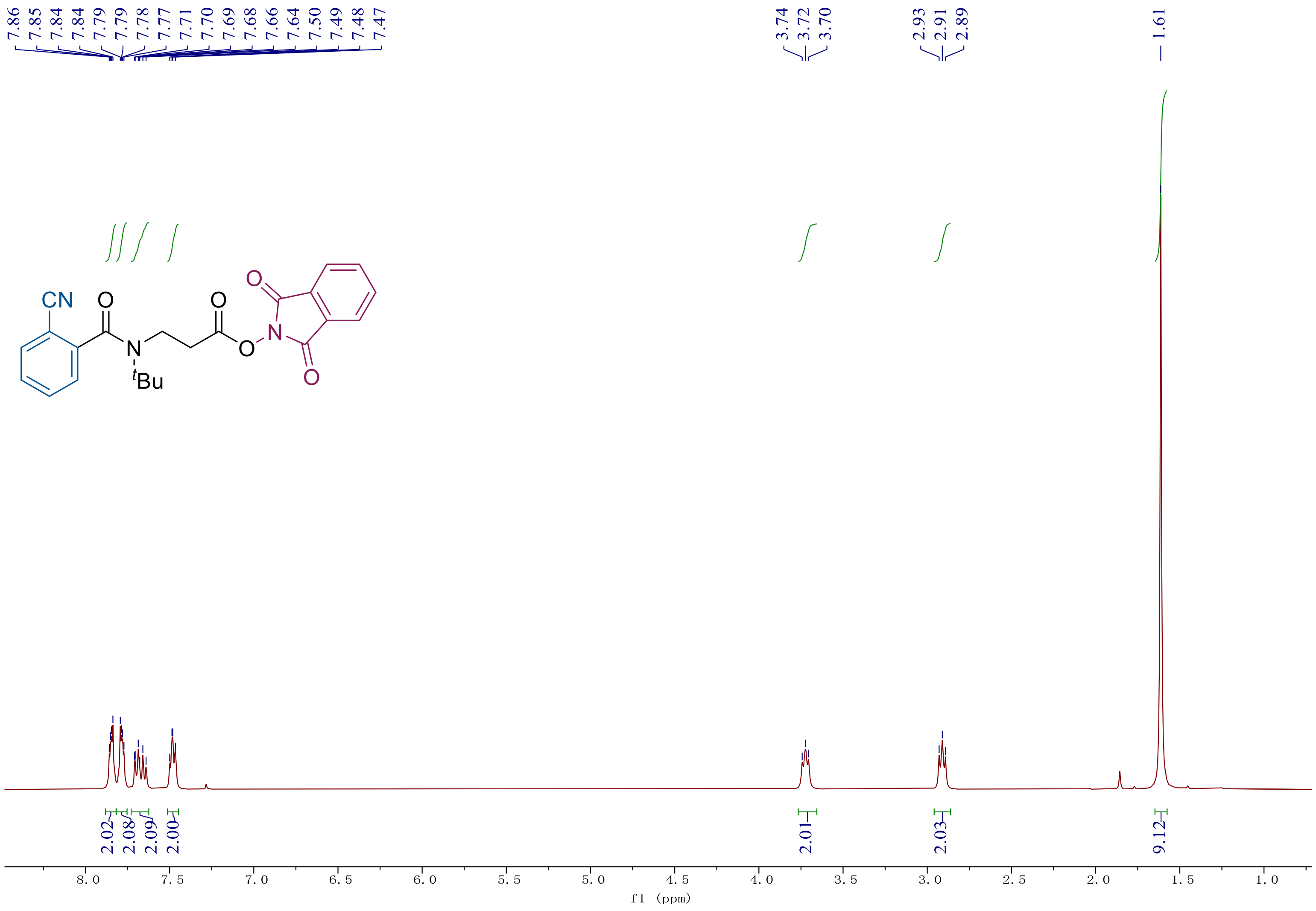

^{13}C NMR (101 MHz, $CDCl_3$) of **1n**

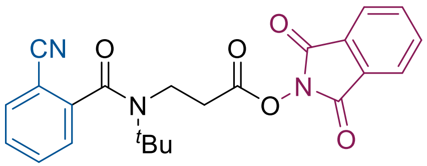

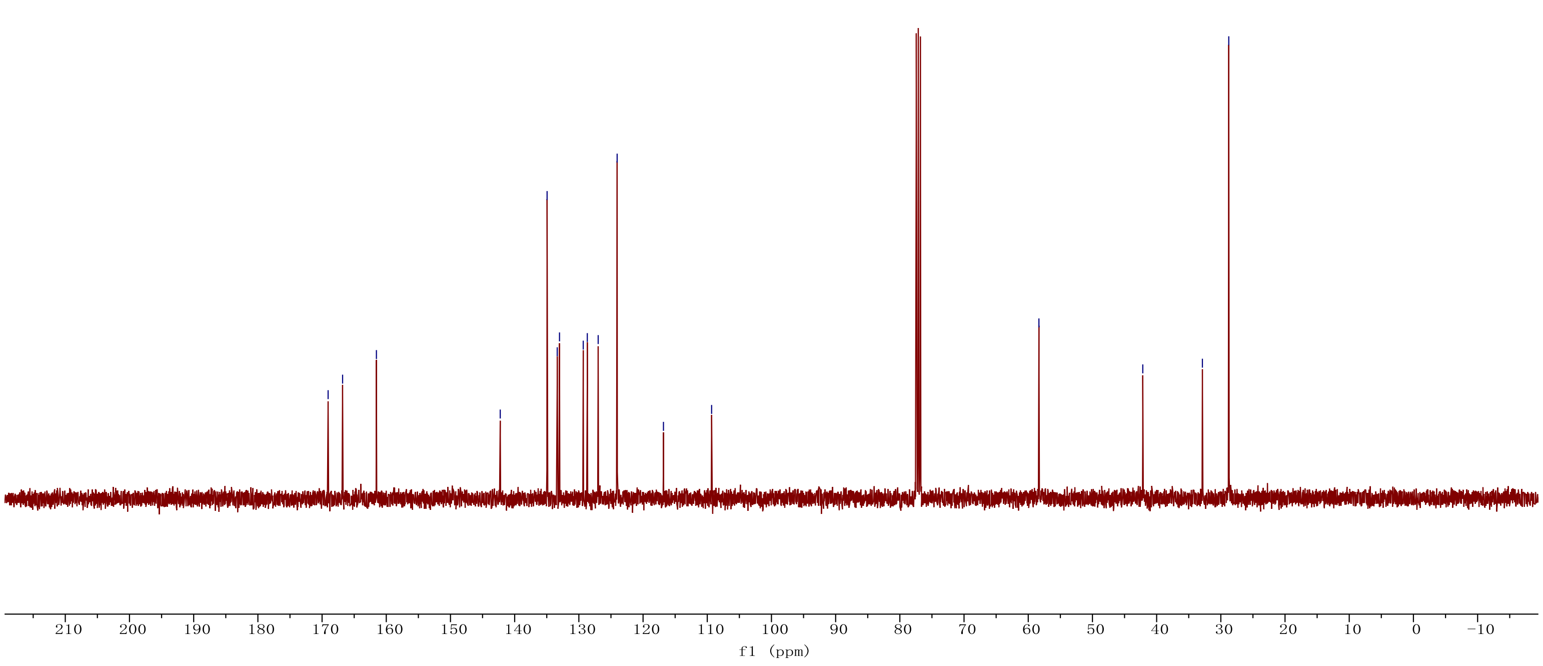

^{1}H NMR (500 MHz, $CDCl_3$) of **1o**

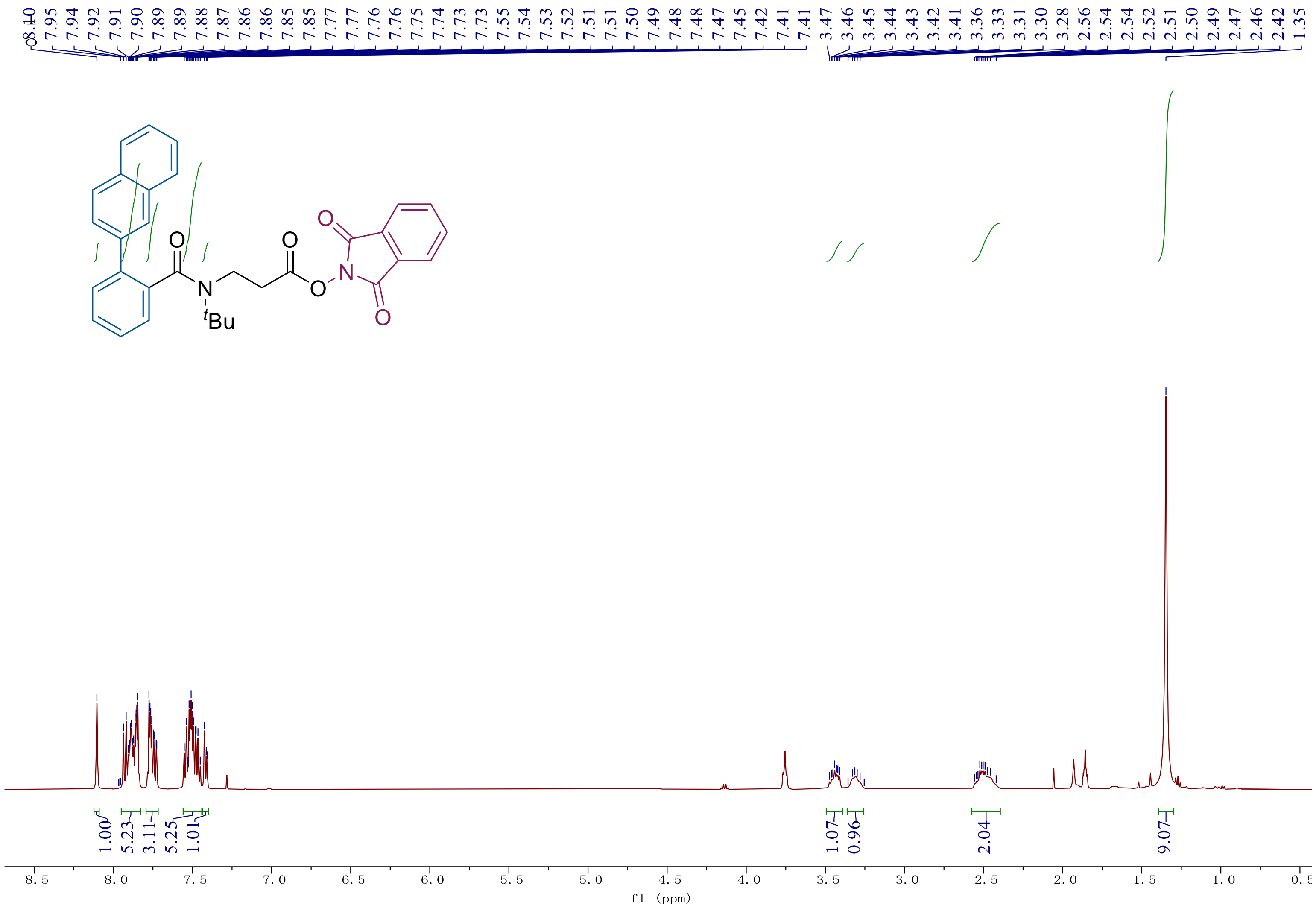

^{13}C NMR (126 MHz, CDCl$_3$) of **1o**

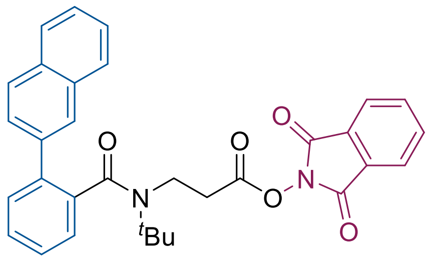

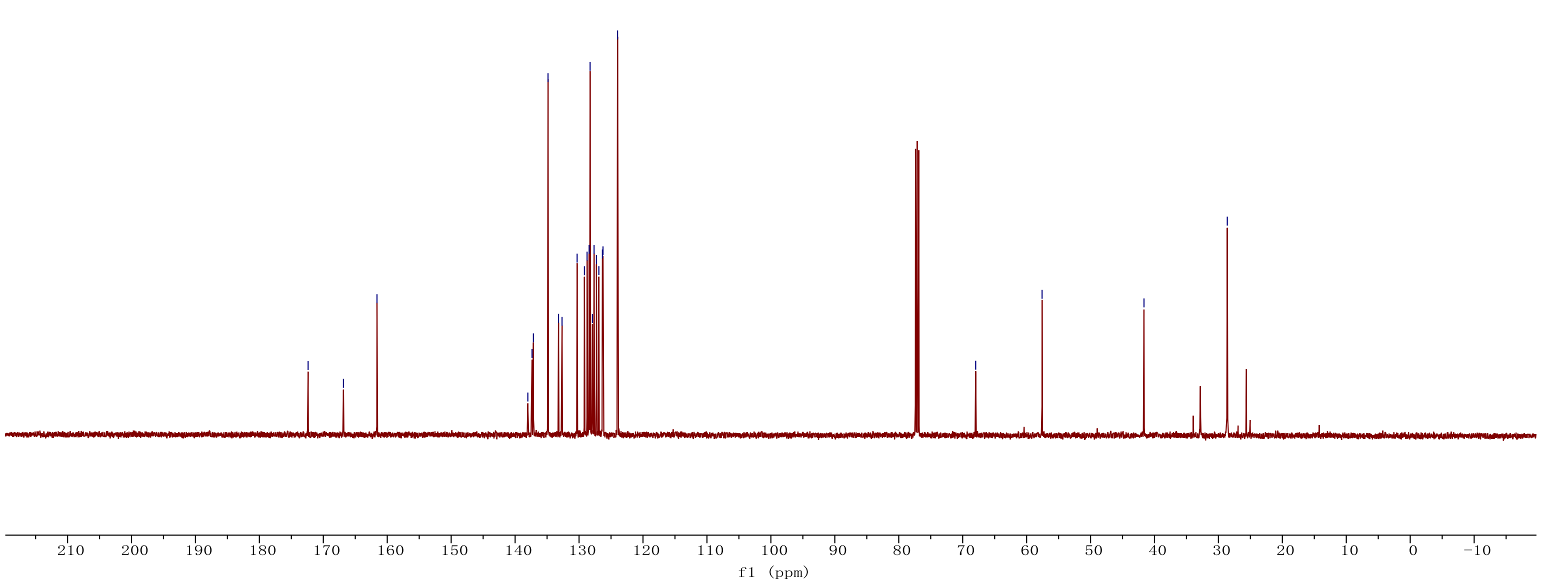

^{1}H NMR (400 MHz, $CDCl_3$) of **1p**

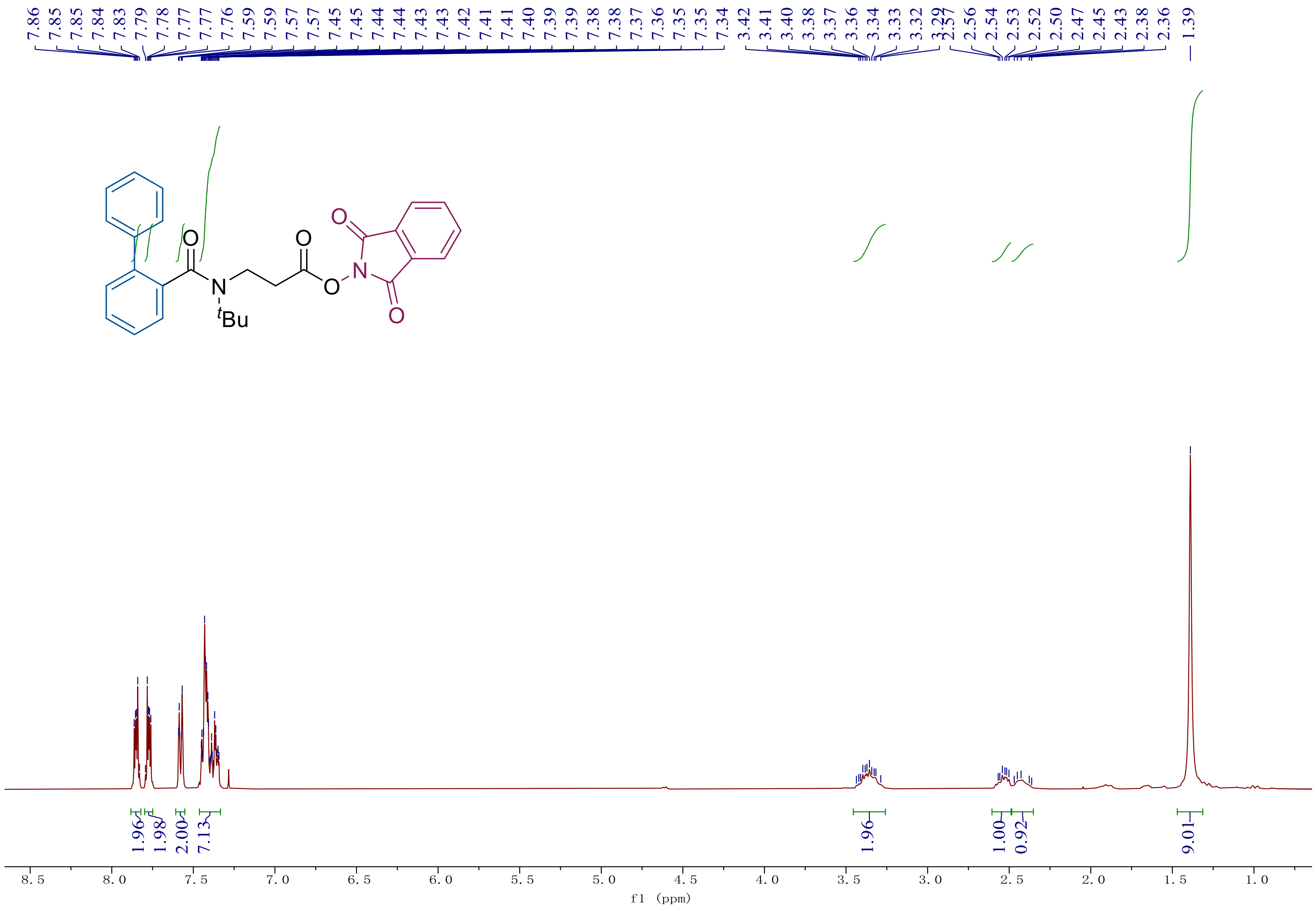

^{13}C NMR (101 MHz, $CDCl_3$) of **1p**

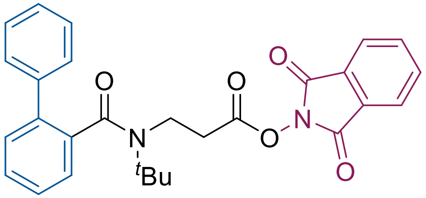

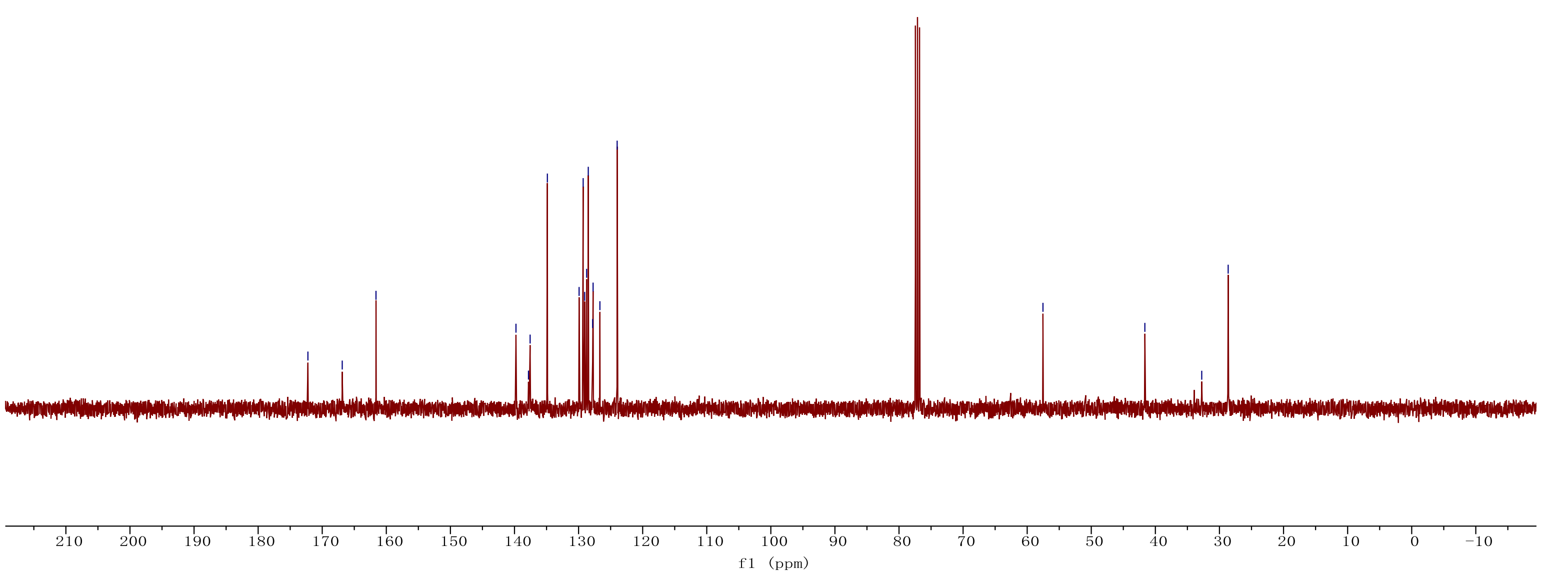

^{1}H NMR (400 MHz, $CDCl_3$) of **1q**

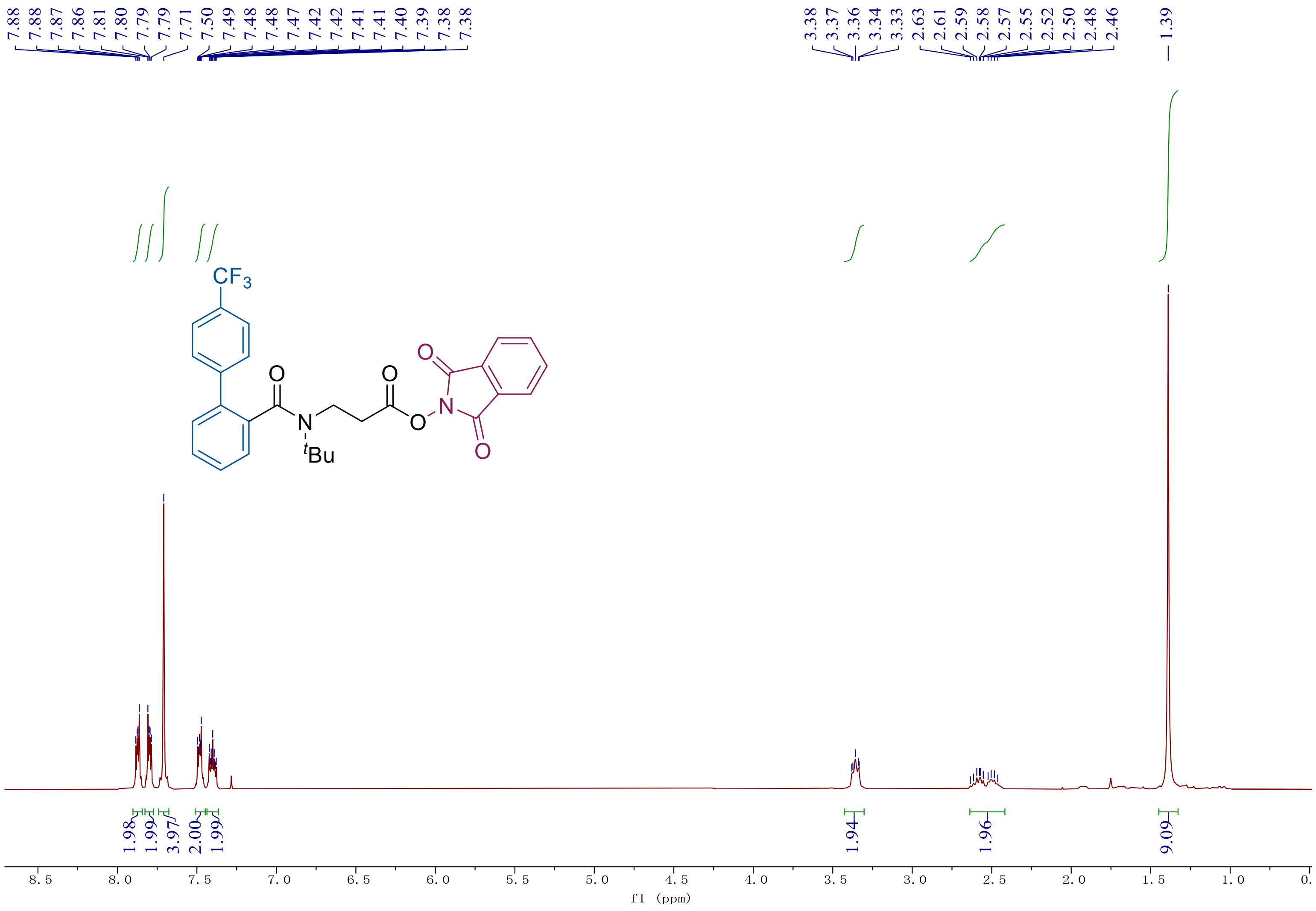

^{13}C NMR (126 MHz, CDCl$_3$) of **1q**

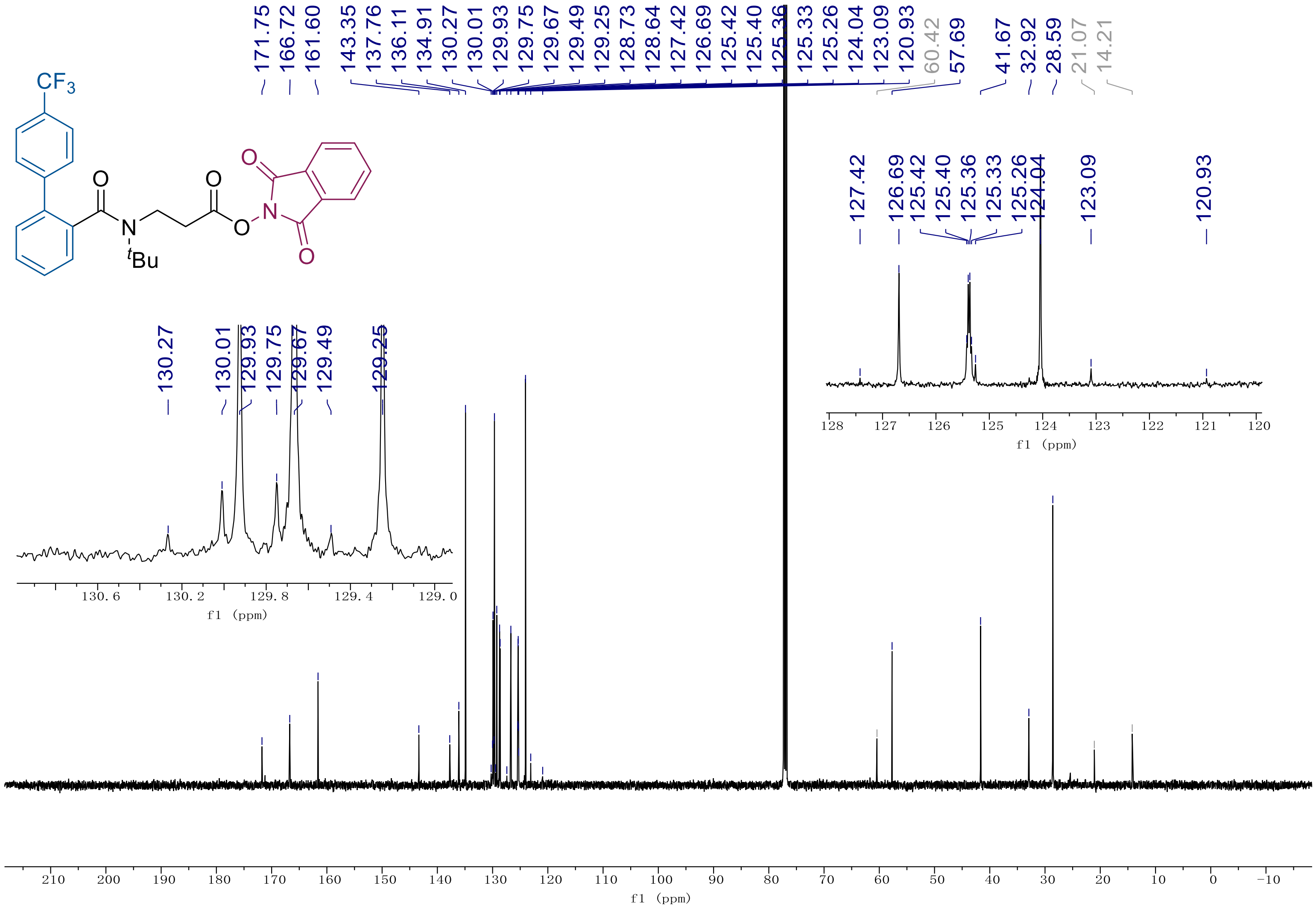

^{19}F NMR (376 MHz, $CDCl_3$) of **1q**

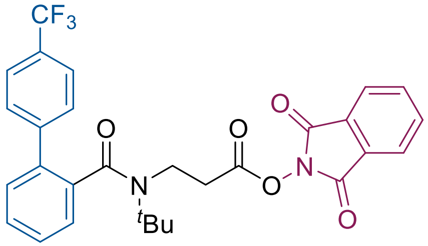

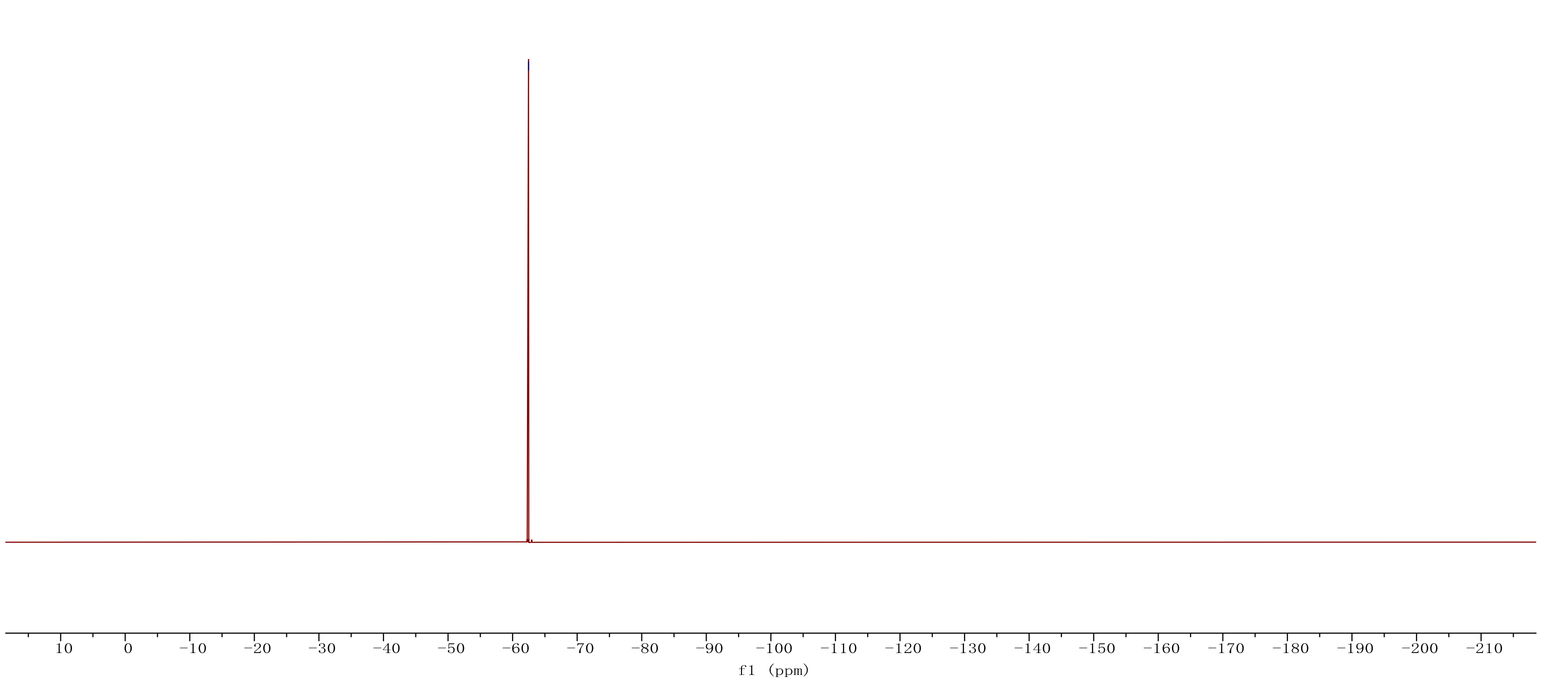

^{1}H NMR (400 MHz, $CDCl_3$) of **1r**

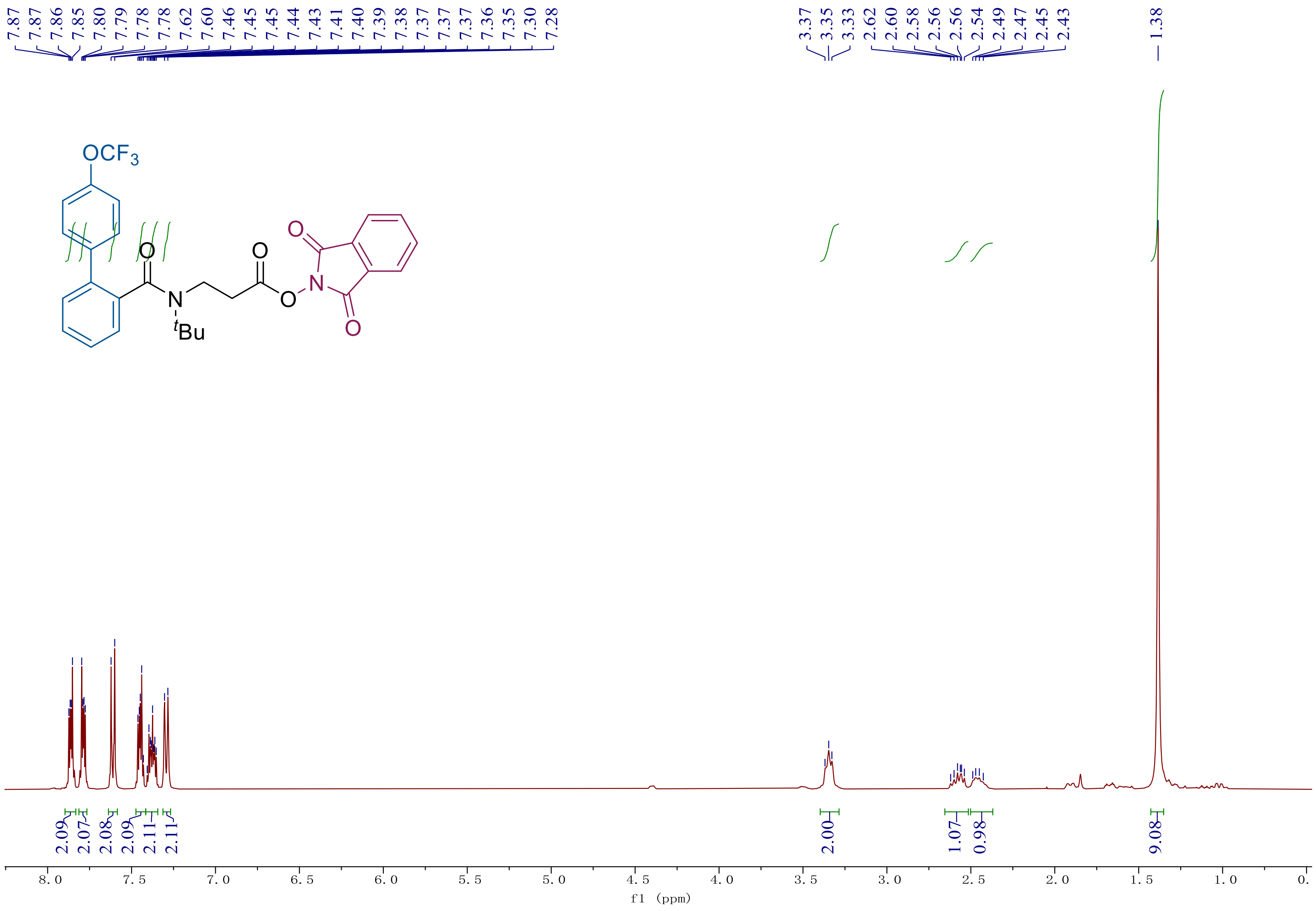

^{13}C NMR (101 MHz, $CDCl_3$) of **1r**

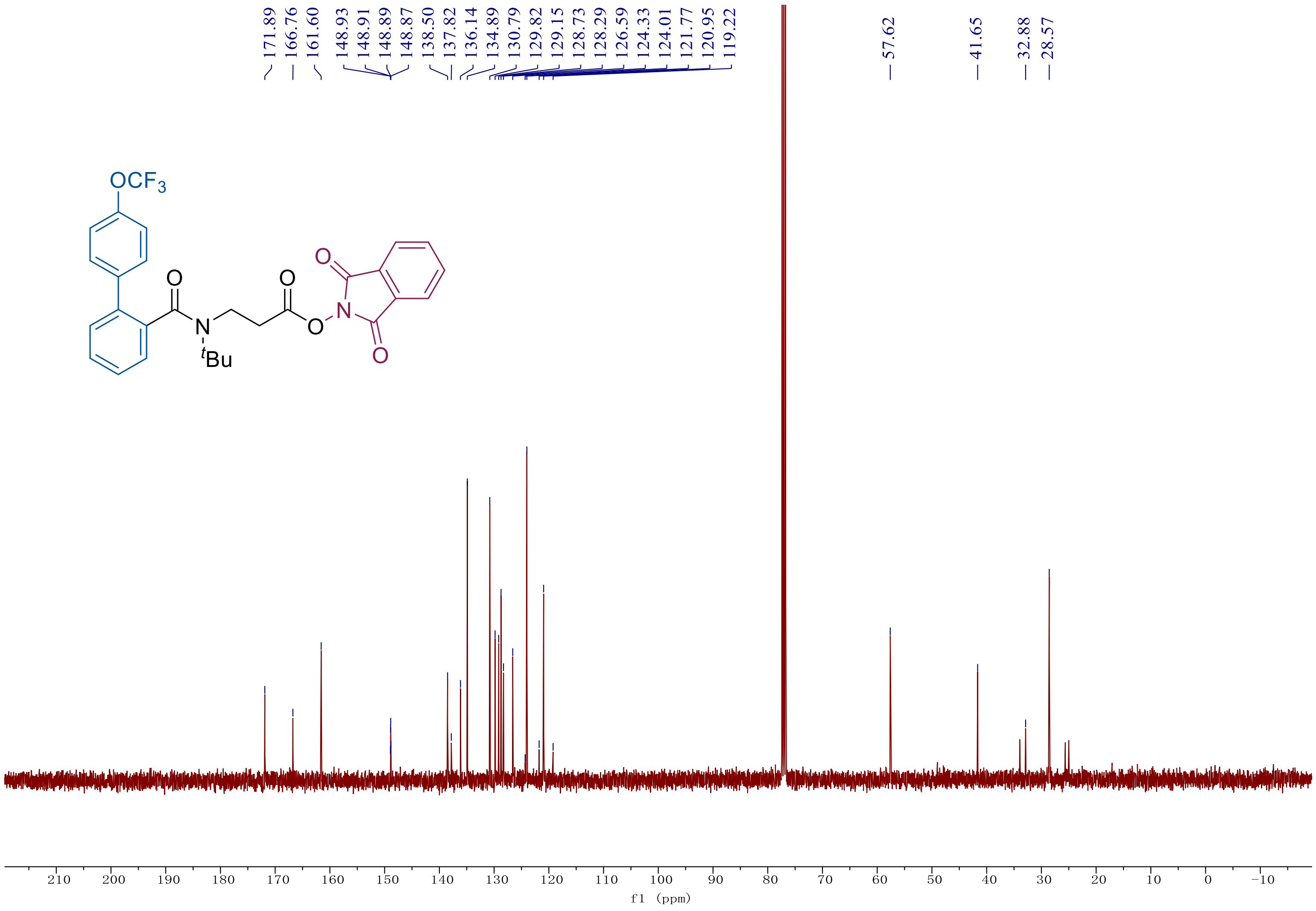

^{19}F NMR (376 MHz, $CDCl_3$) of **1r**

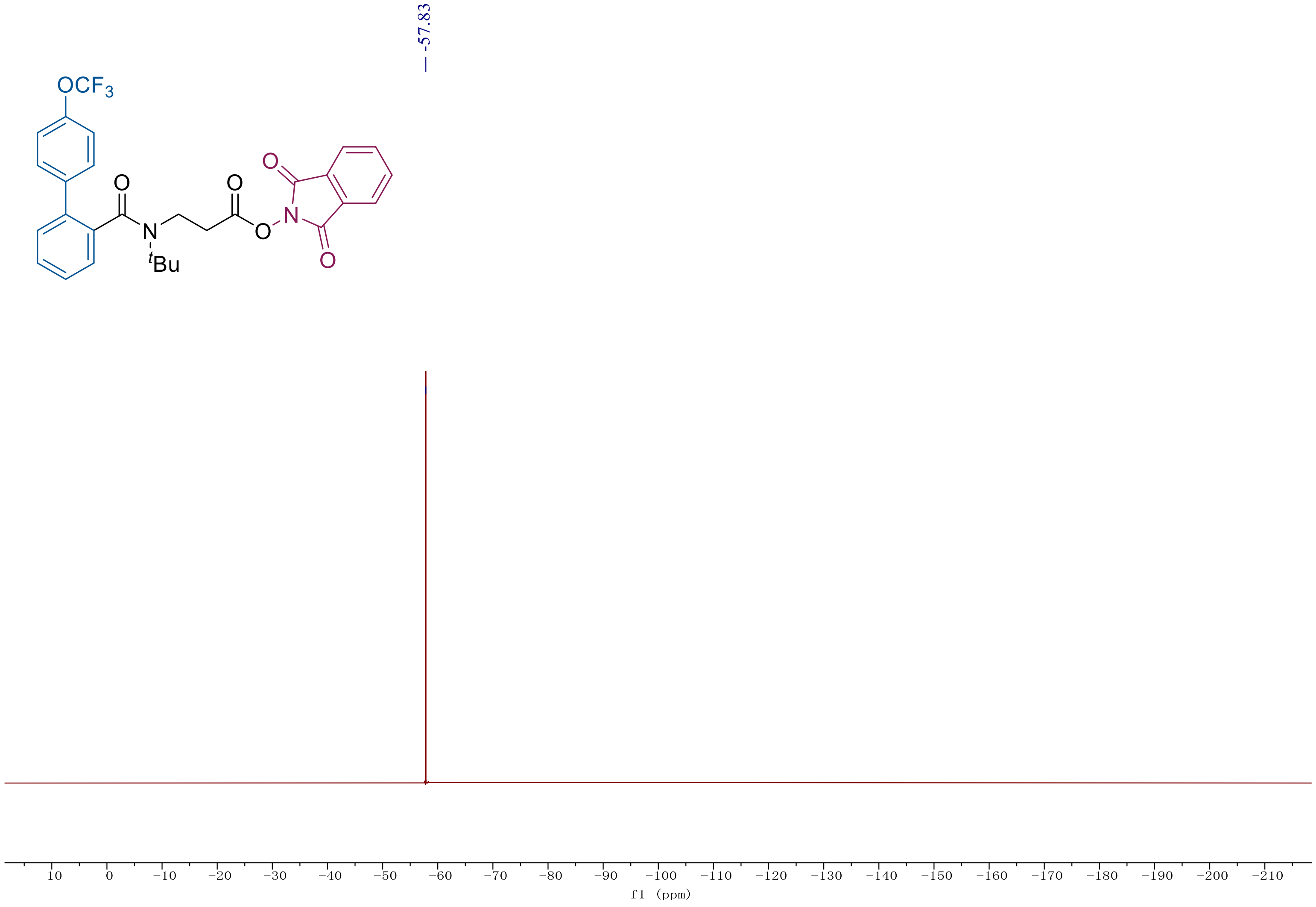

^{1}H NMR (400 MHz, $CDCl_3$) of **1s**

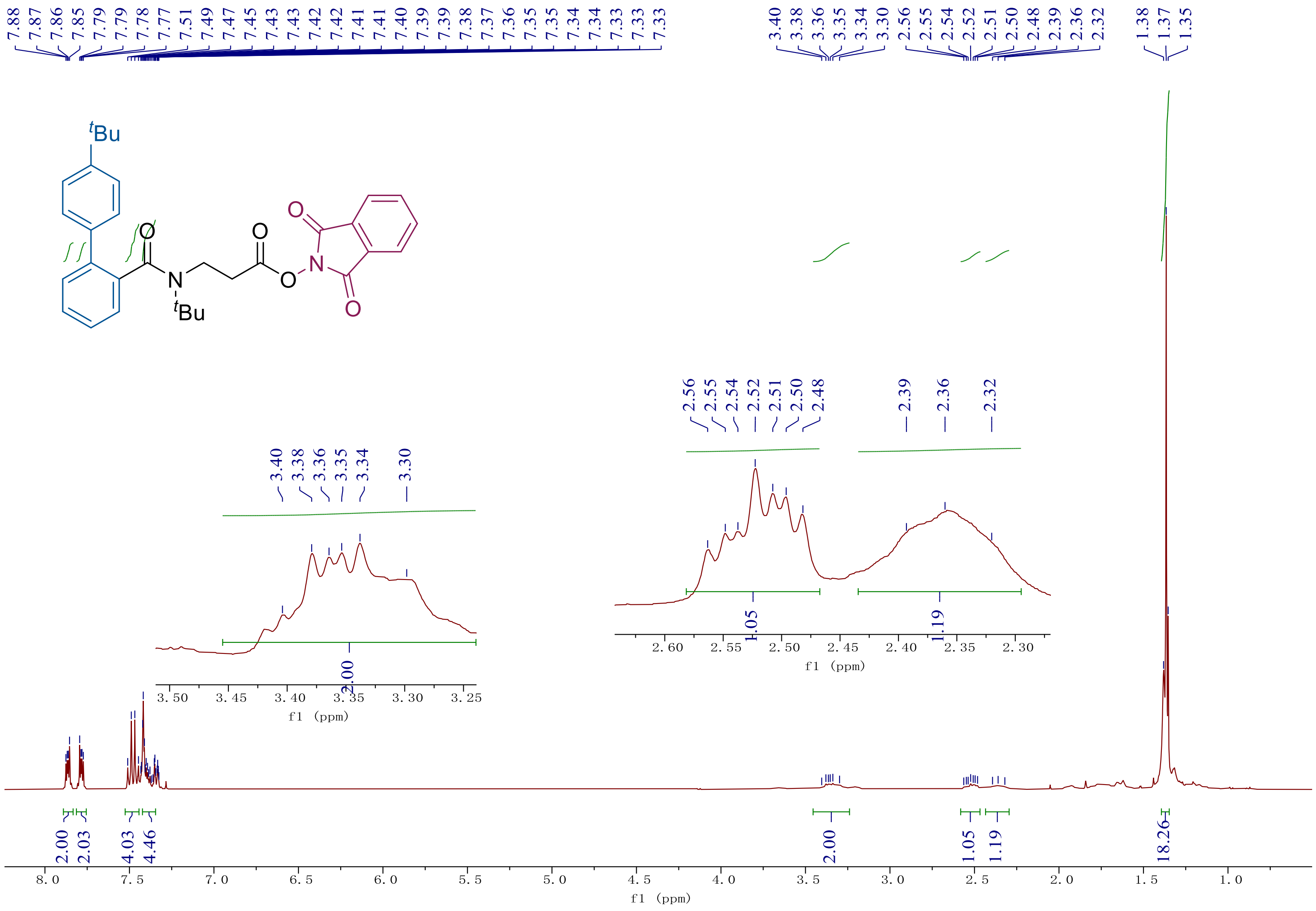

^{13}C NMR (126 MHz, $CDCl_3$) of **1s**

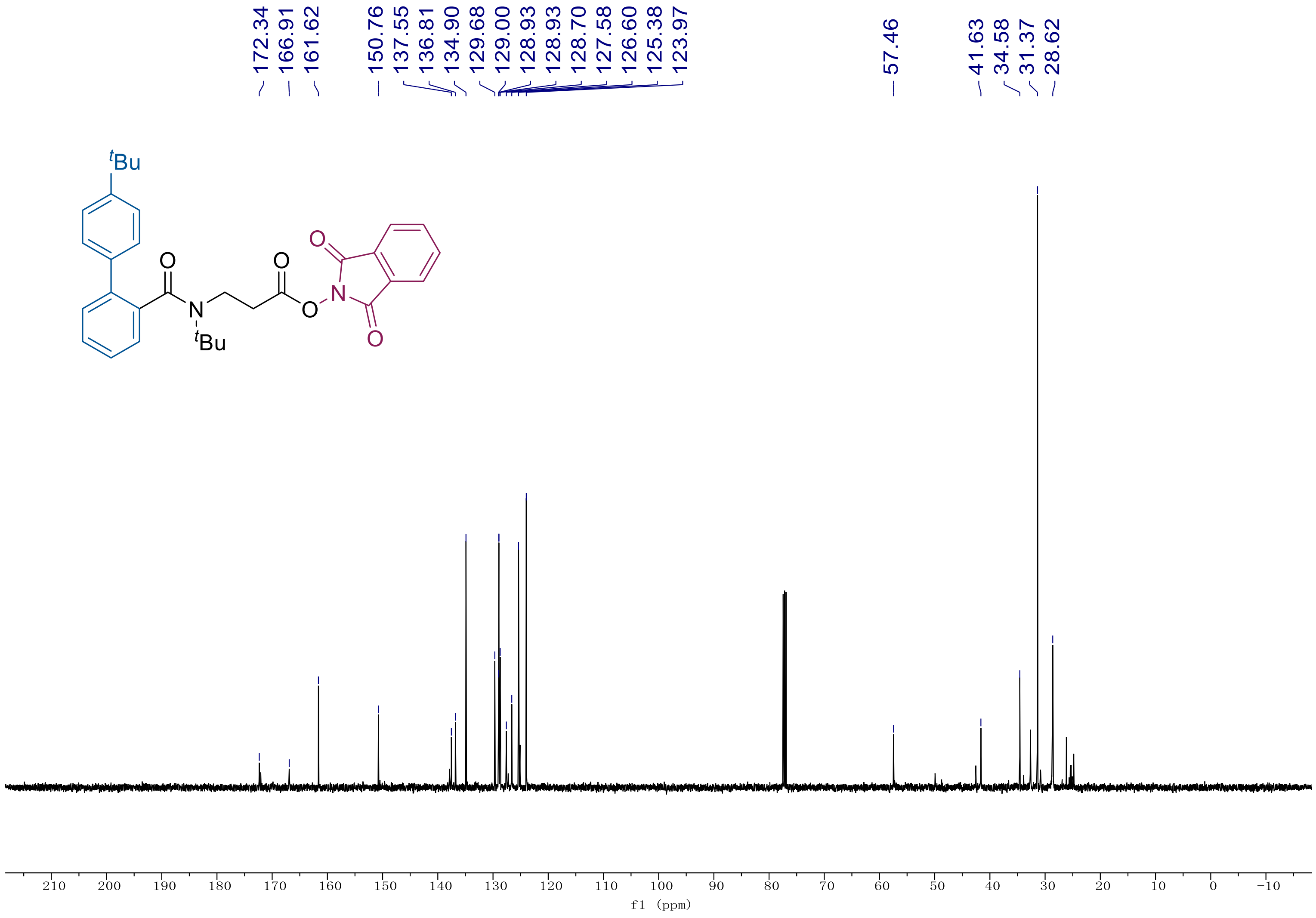

^{1}H NMR (400 MHz, $CDCl_3$) of **1t**

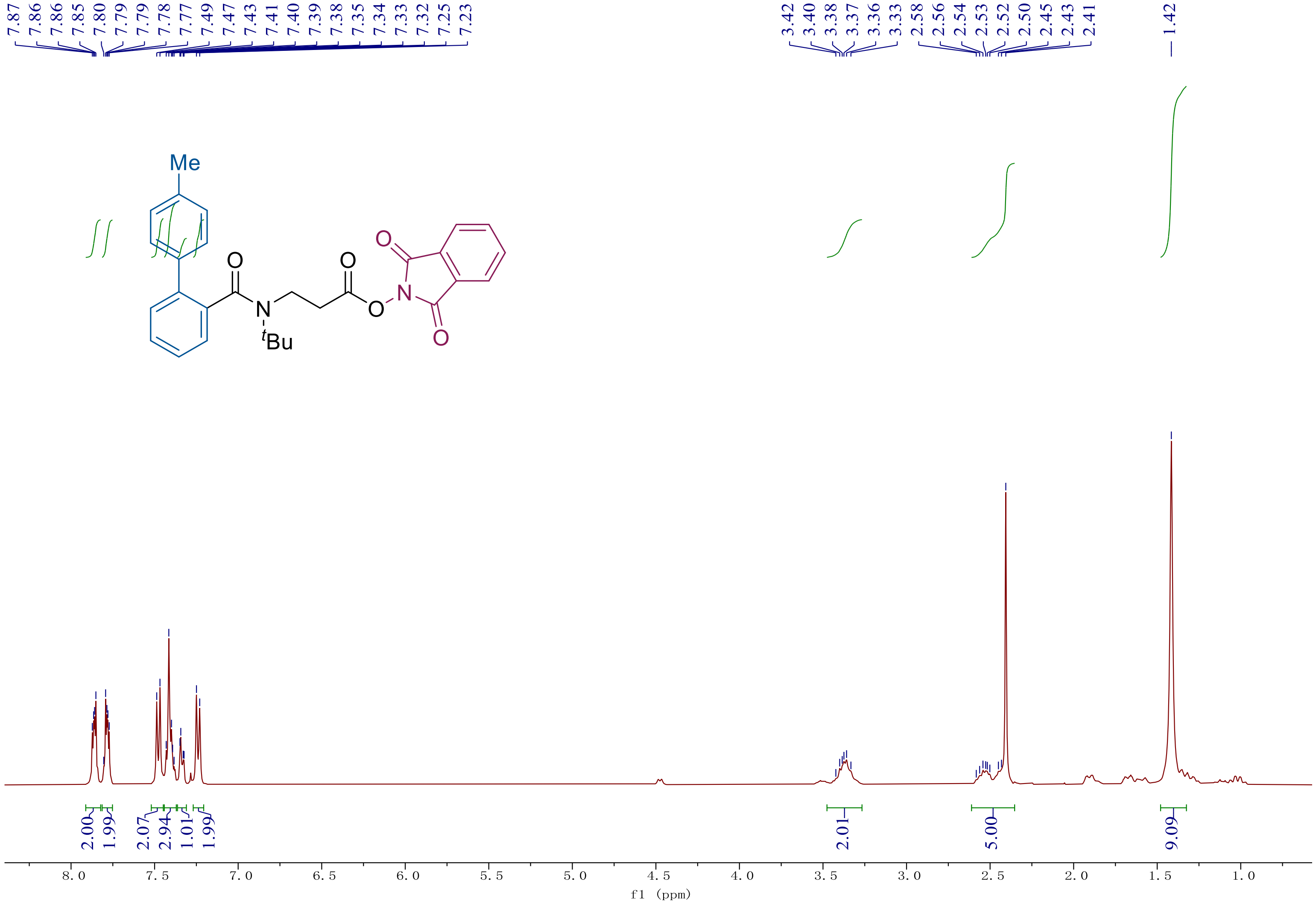

^{13}C NMR (101 MHz, $CDCl_3$) of **1t**

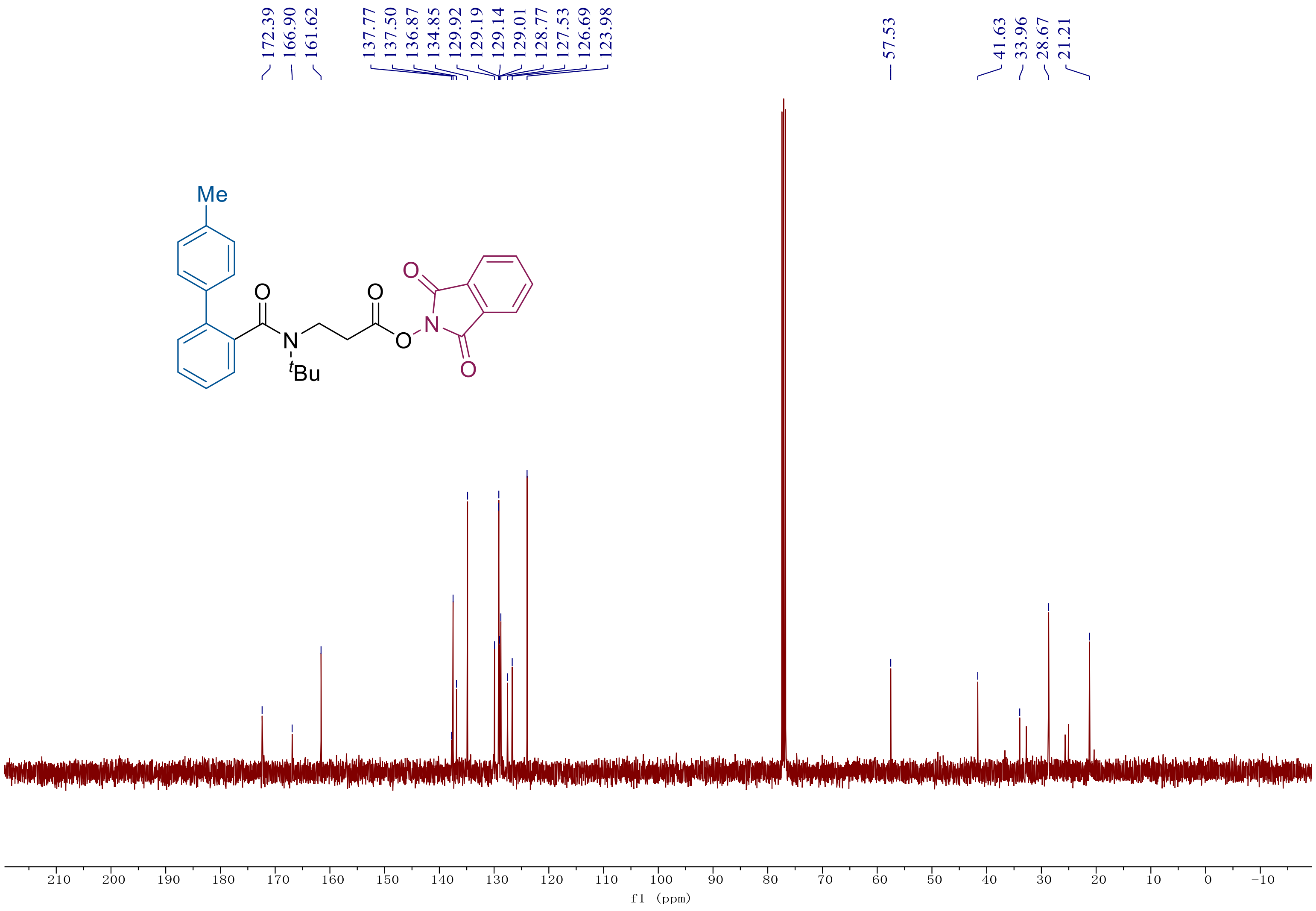

^{1}H NMR (400 MHz, $CDCl_3$) of **1u**

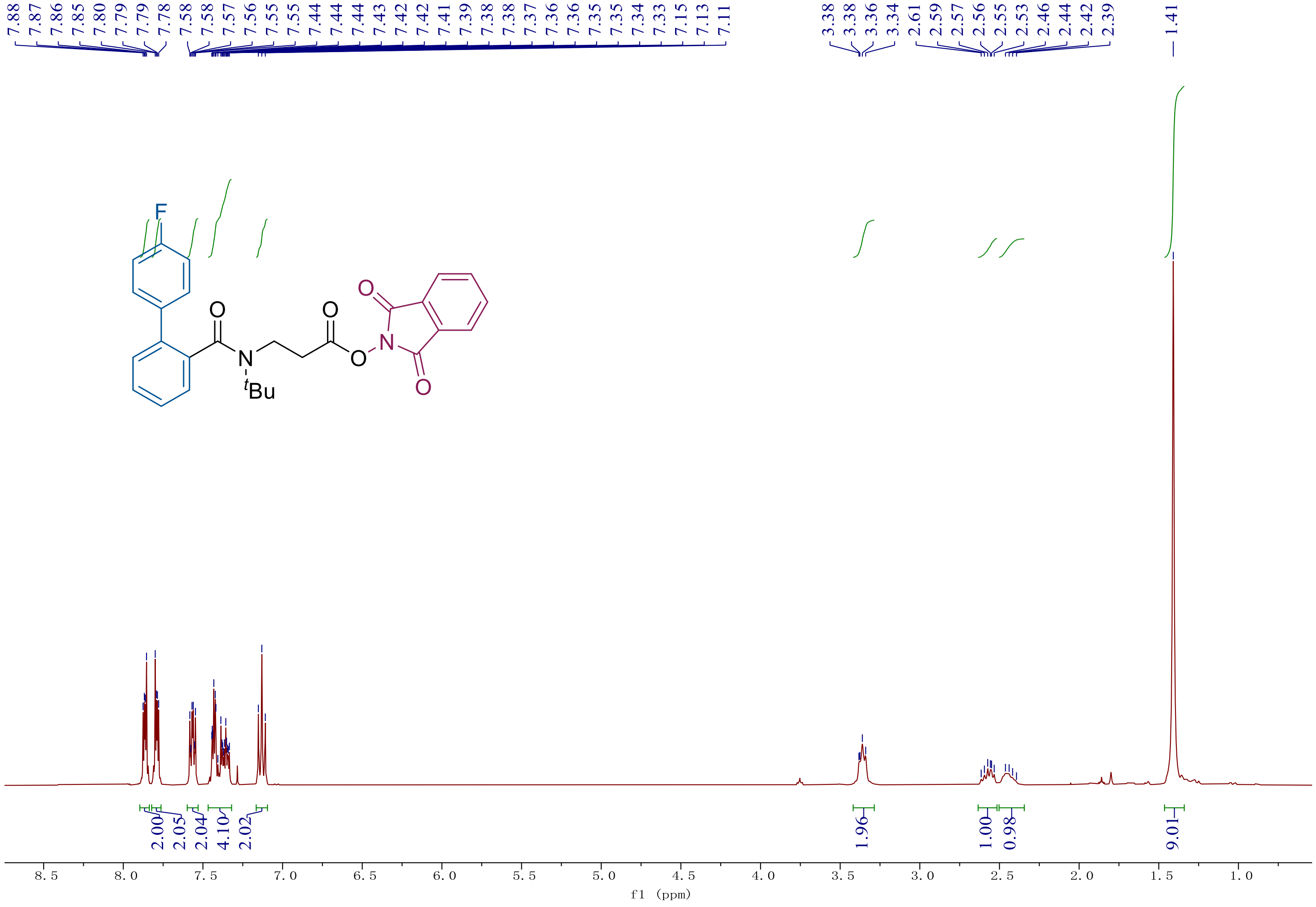

^{13}C NMR (101 MHz, $CDCl_3$) of **1u**

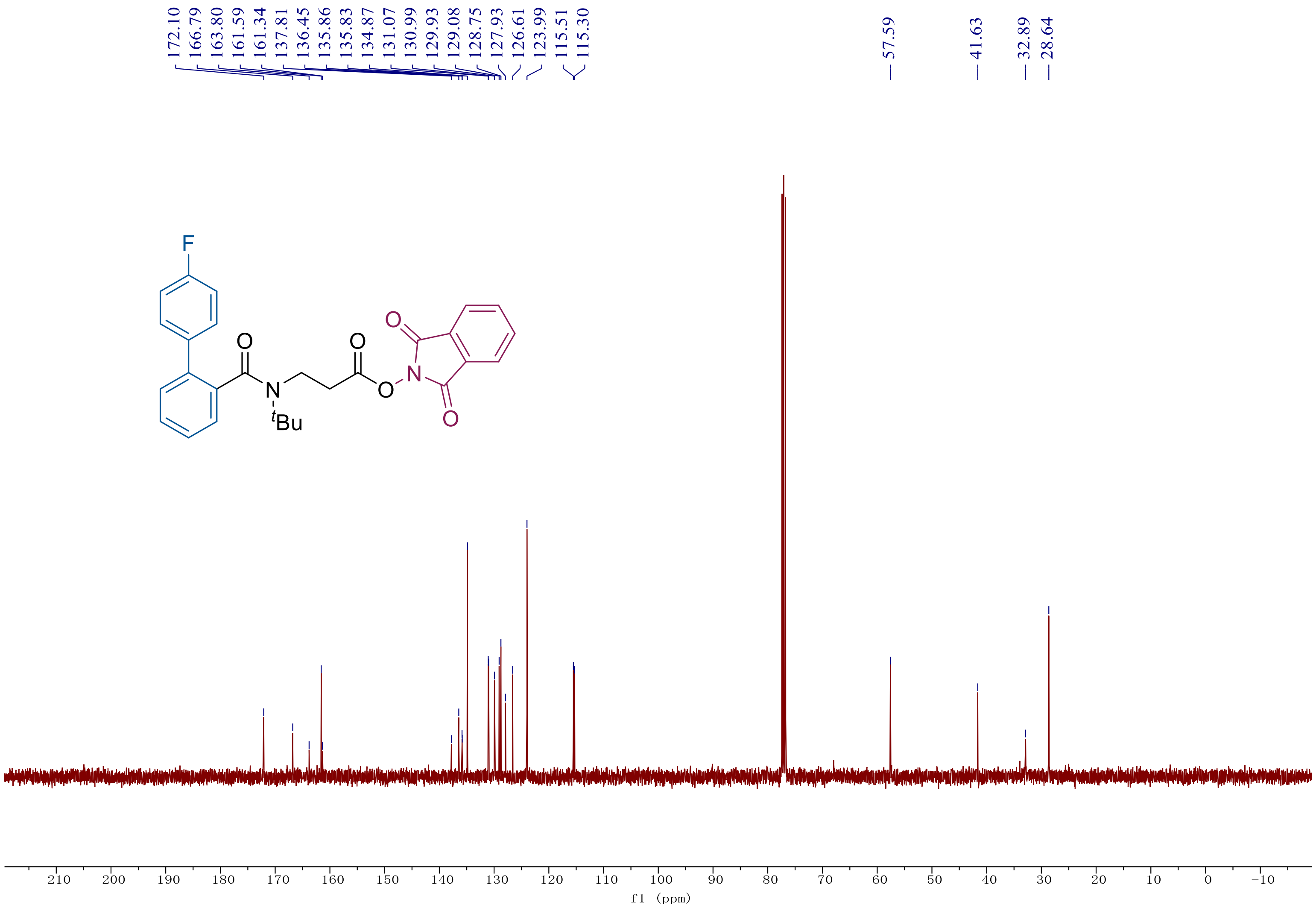

^{19}F NMR (376 MHz, $CDCl_3$) of **1u**

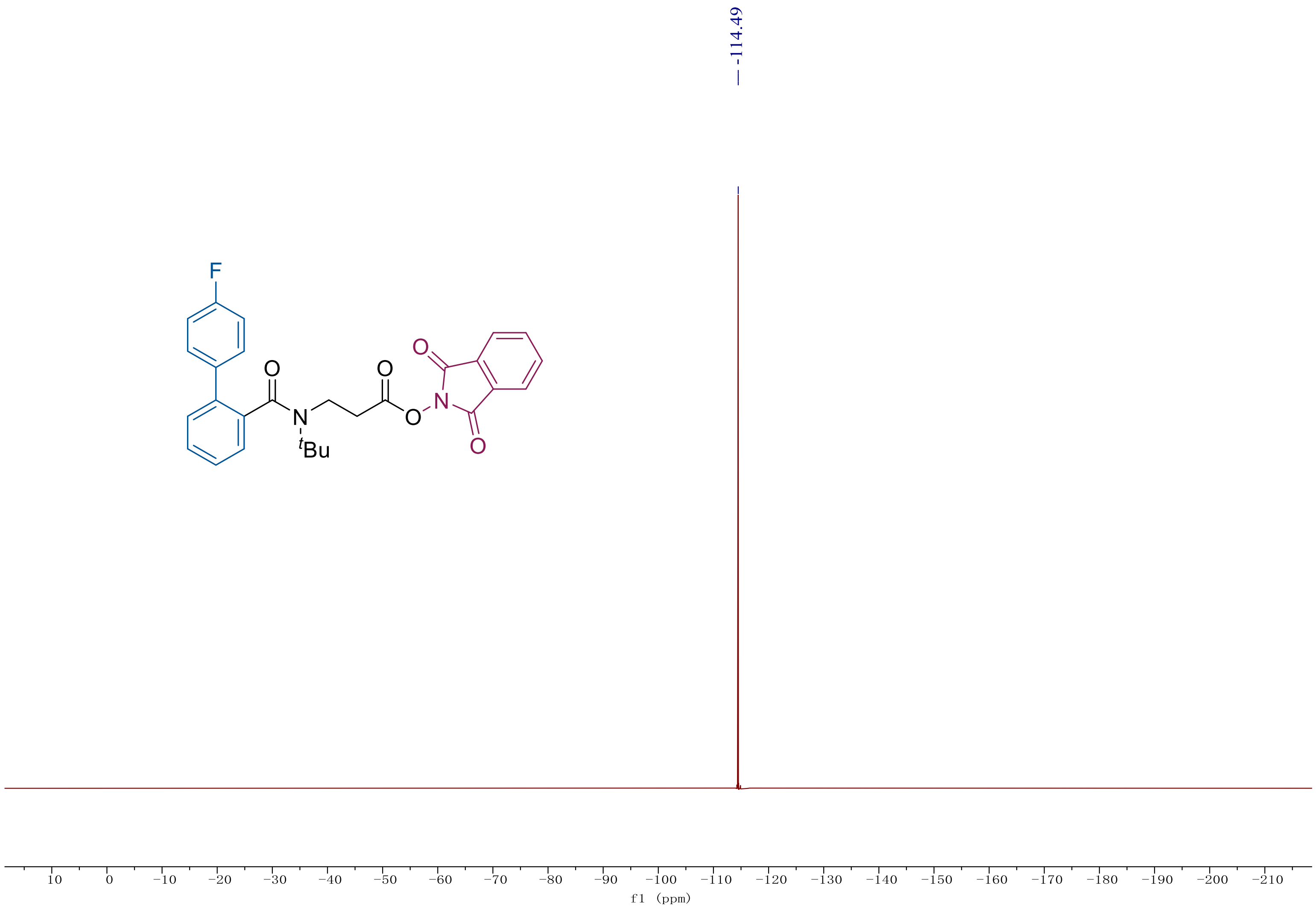

^{1}H NMR (400 MHz, $CDCl_3$) of **1v**

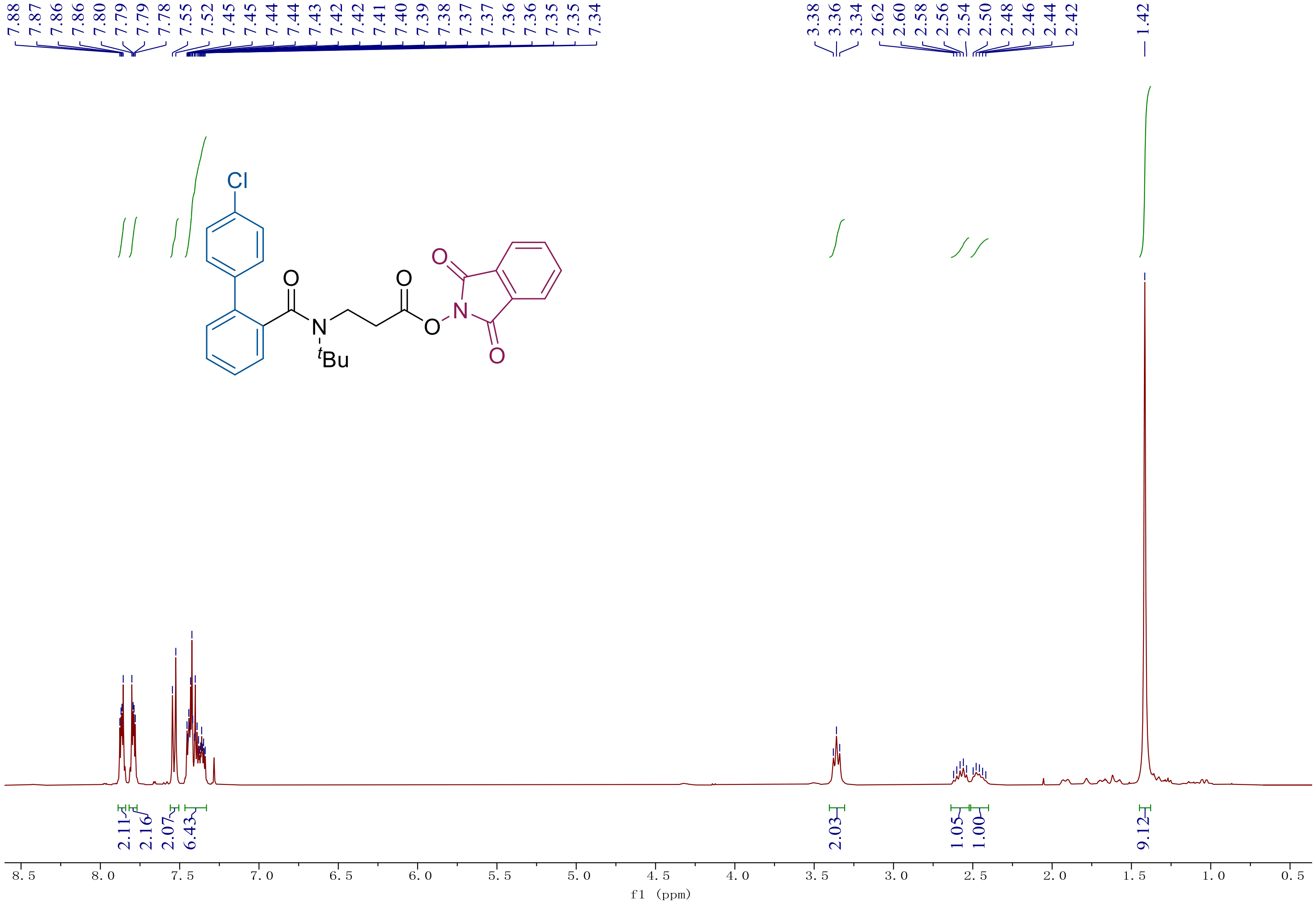

^{13}C NMR (101 MHz, $CDCl_3$) of **1v**

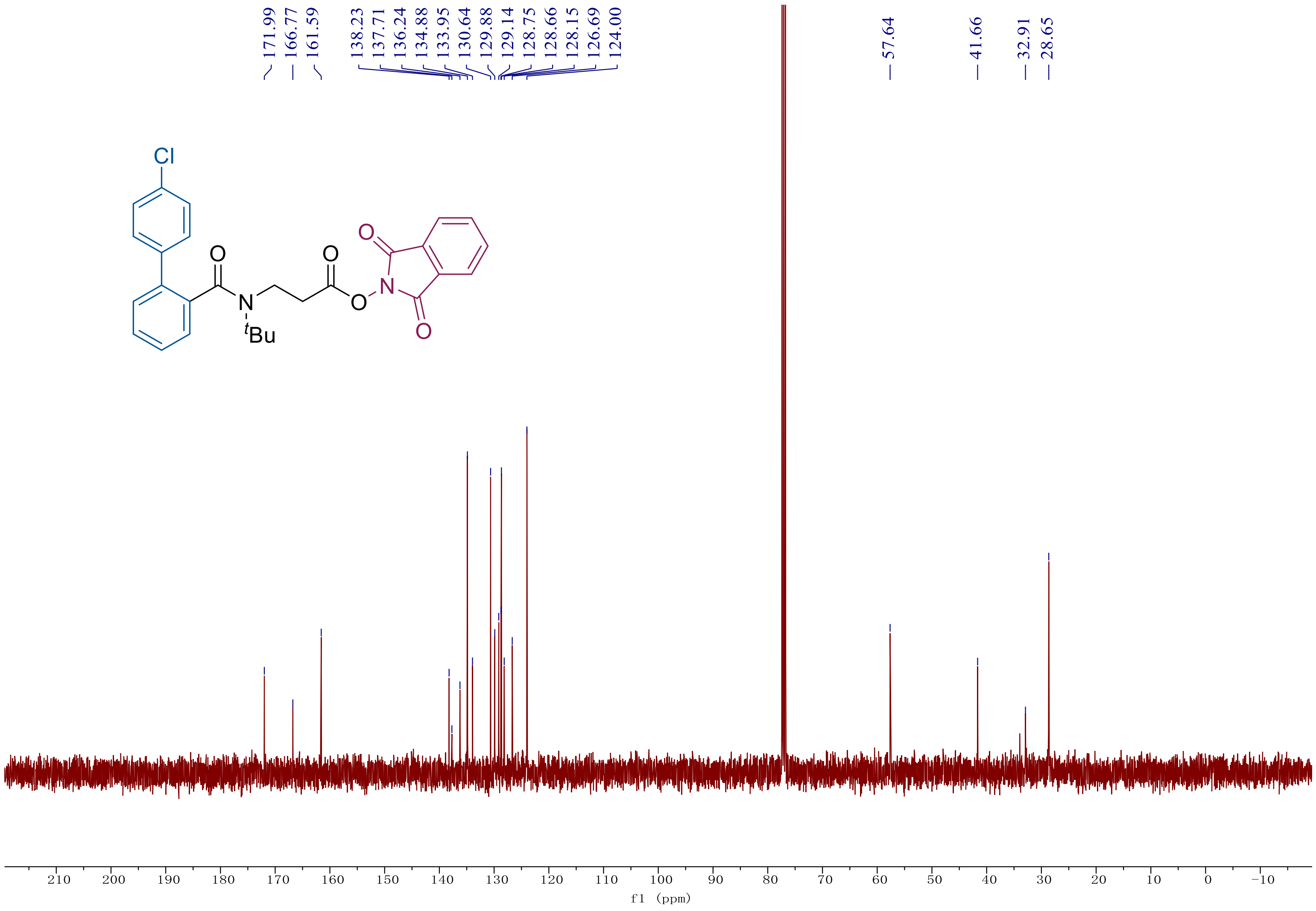

^{1}H NMR (400 MHz, $CDCl_3$) of **1w**

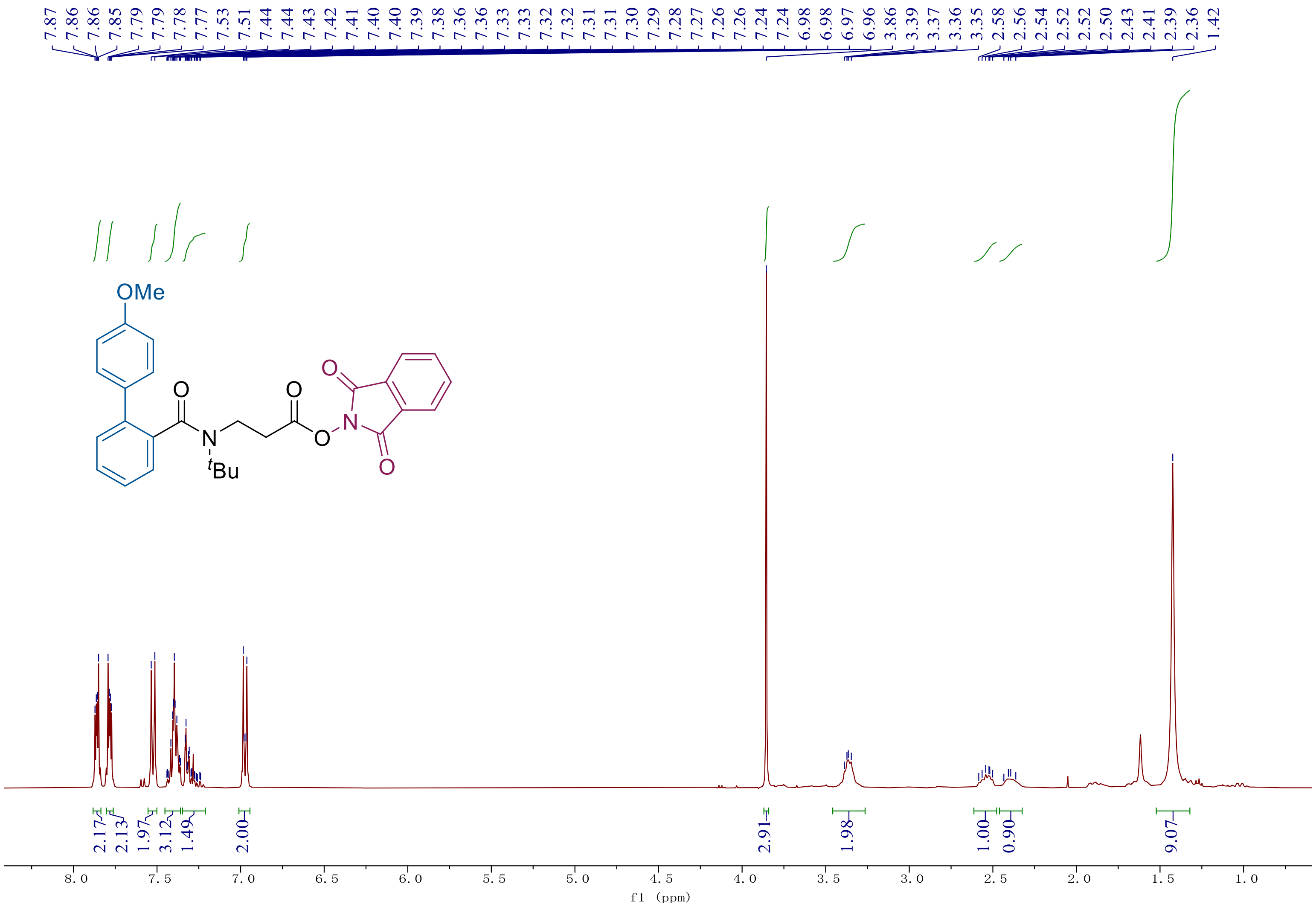

^{13}C NMR (101 MHz, CDCl$_3$) of **1w**

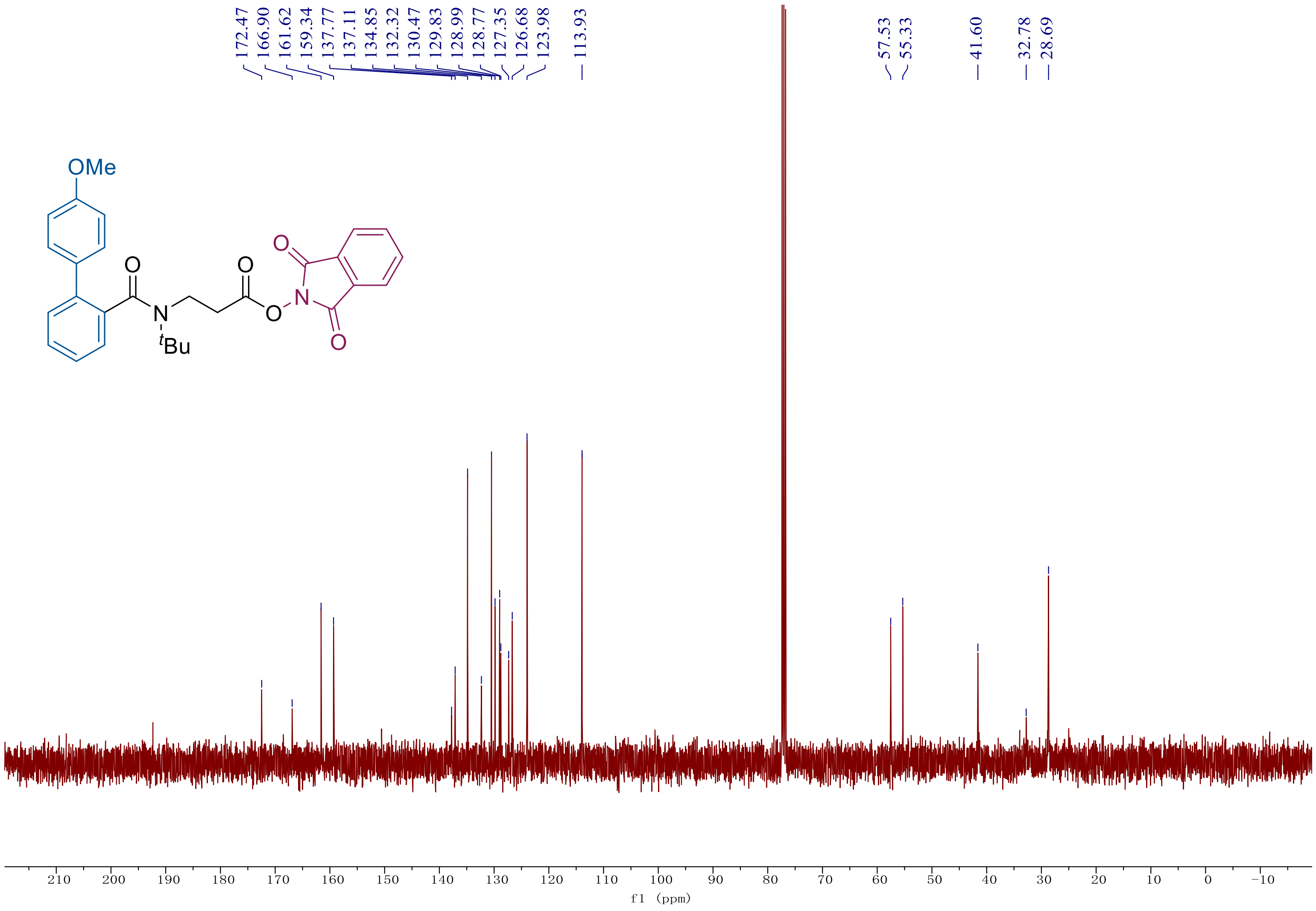

^{1}H NMR (400 MHz, $CDCl_3$) of **1x**

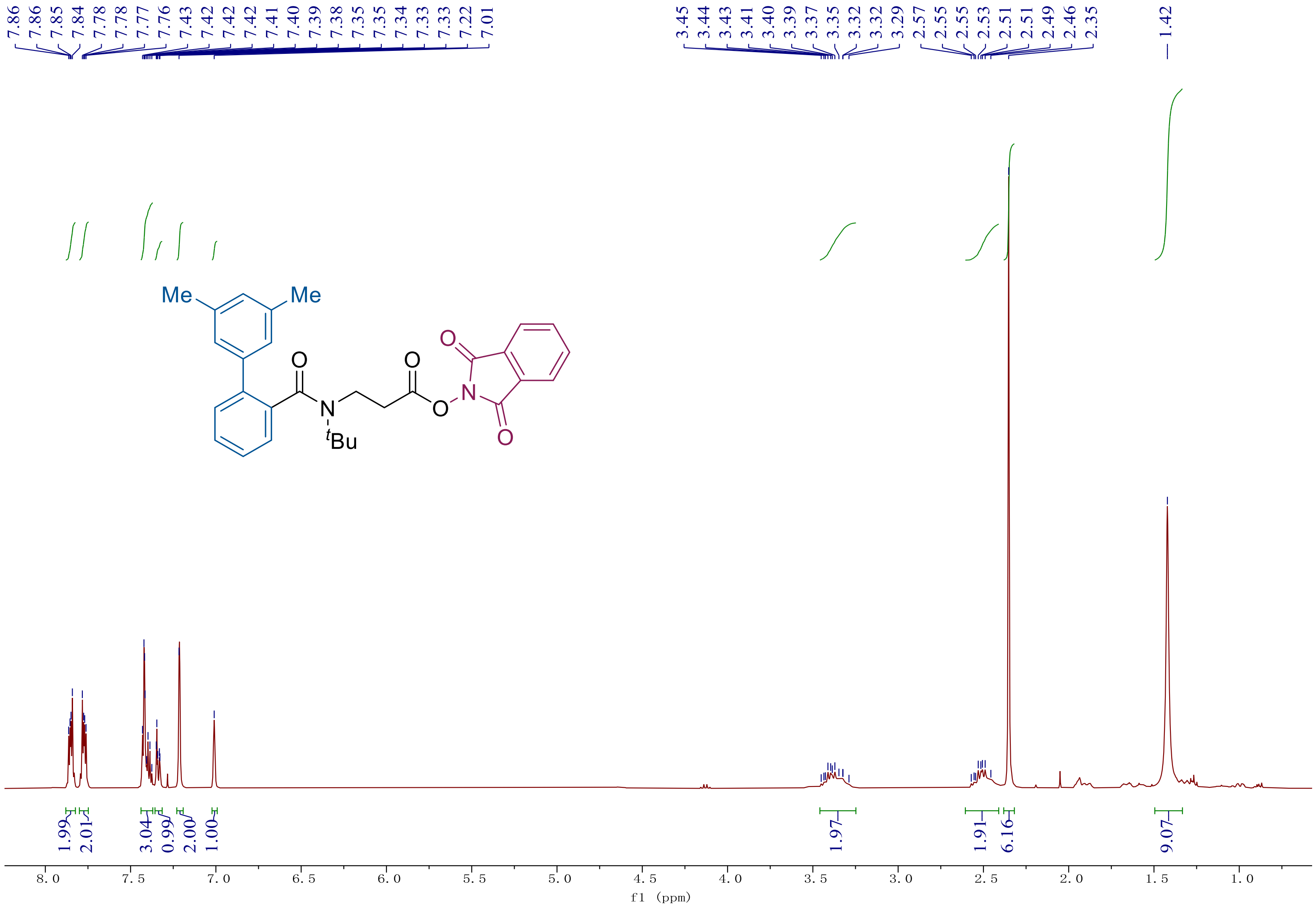

^{13}C NMR (101 MHz, $CDCl_3$) of **1x**

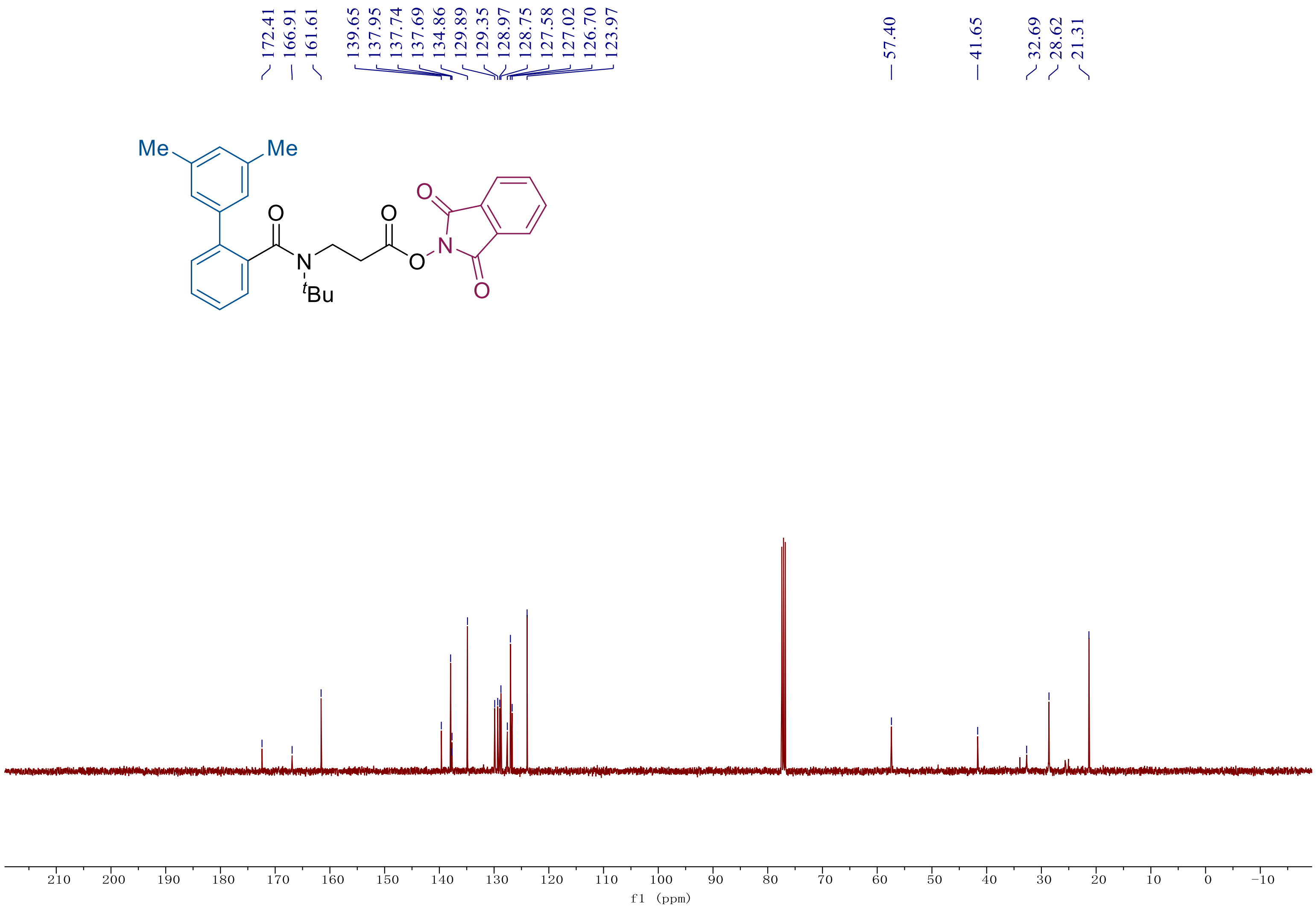

^{1}H NMR (400 MHz, $CDCl_3$) of **1y**

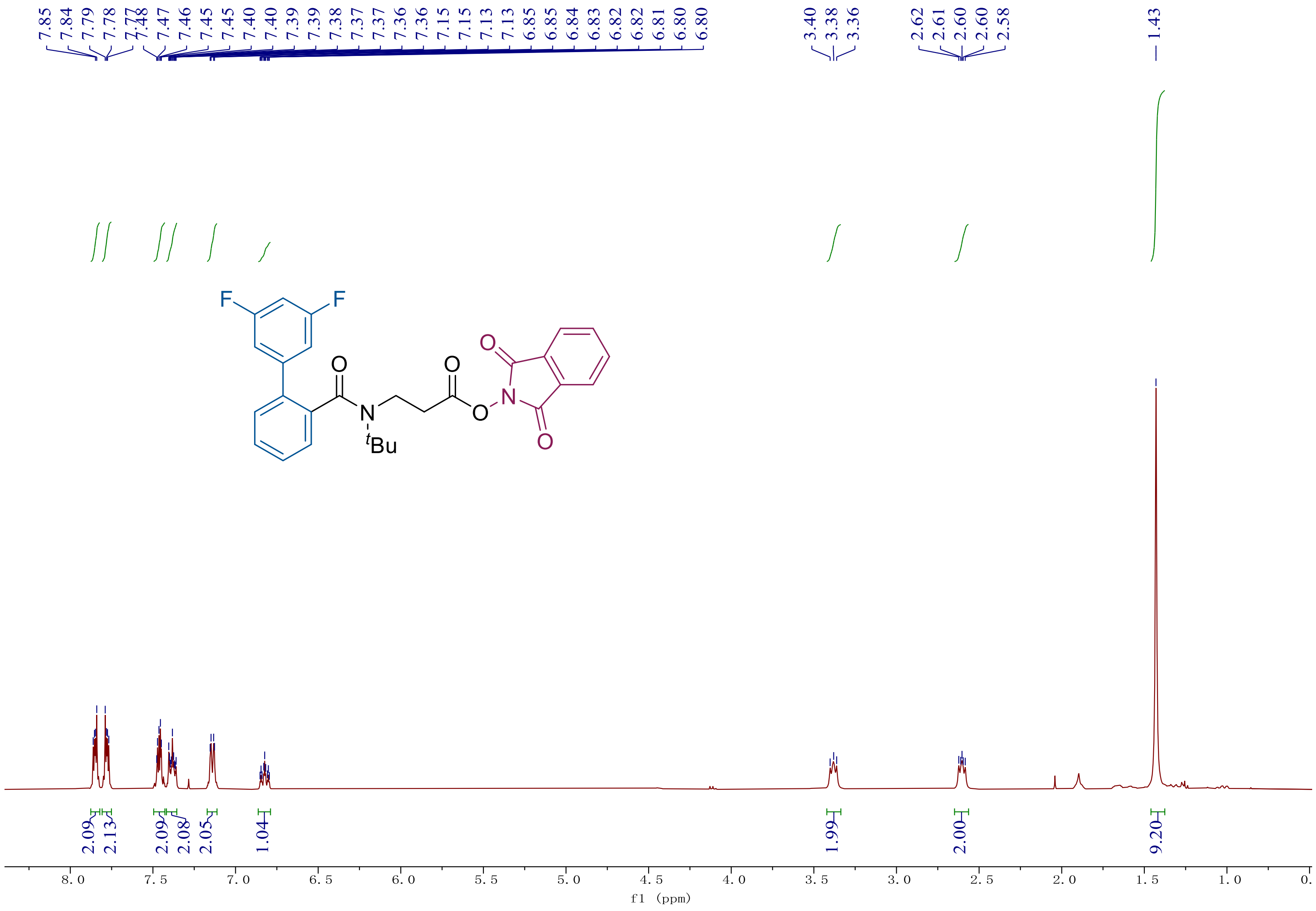

^{13}C NMR (101 MHz, $CDCl_3$) of **1y**

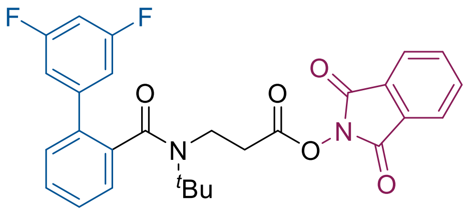

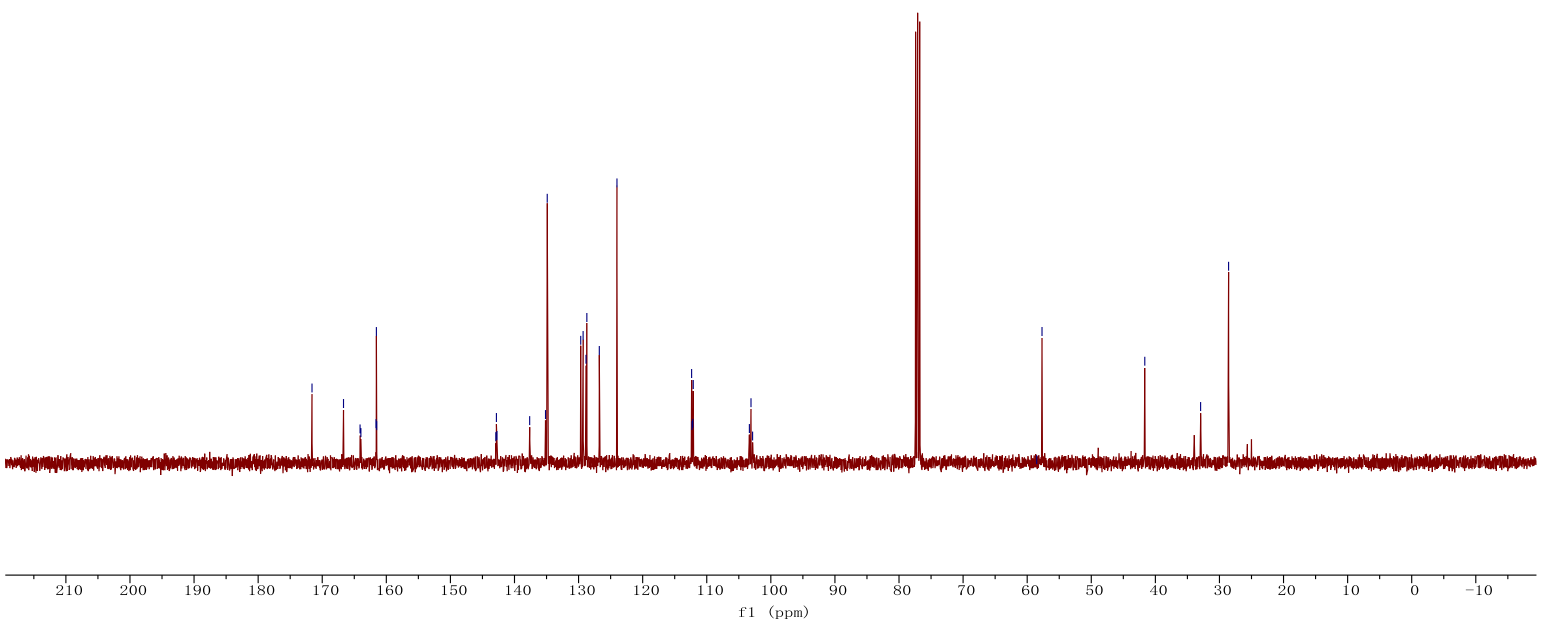

^{19}F NMR (376 MHz, $CDCl_3$) of **1y**

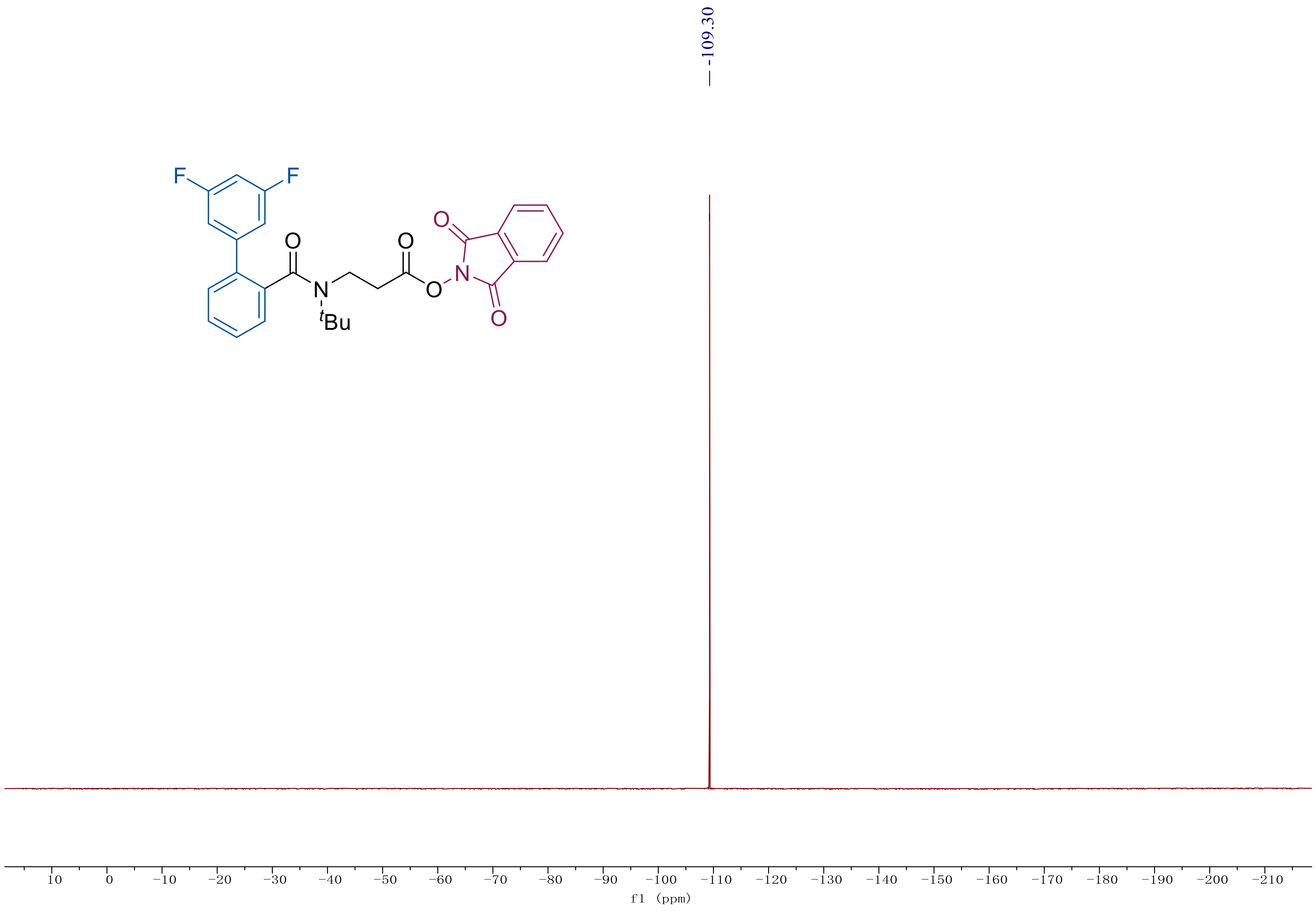

^{1}H NMR (400 MHz, $CDCl_3$) of **1z**

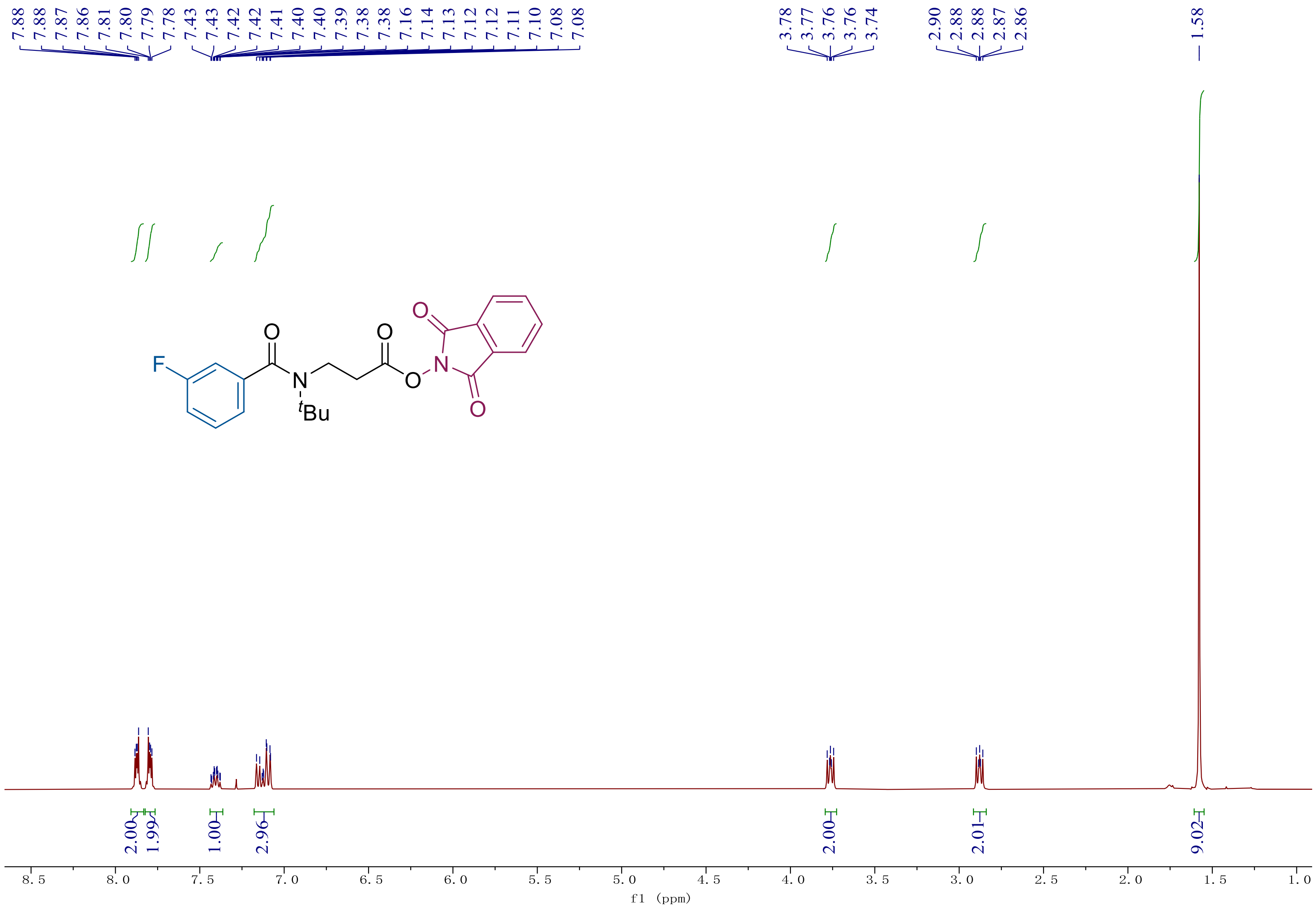

^{13}C NMR (101 MHz, CDCl$_3$) of **1z**

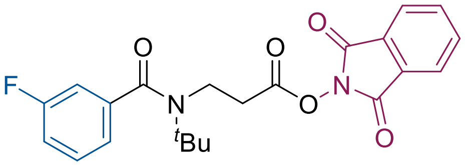

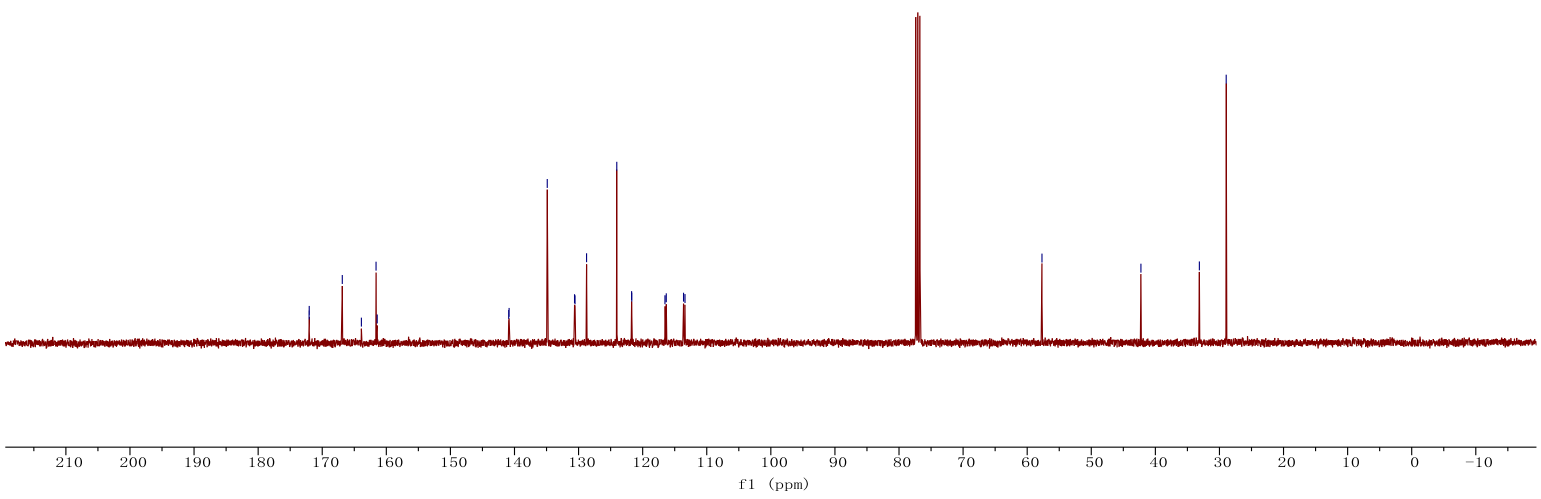

^{19}F NMR (376 MHz, $CDCl_3$) of **1z**

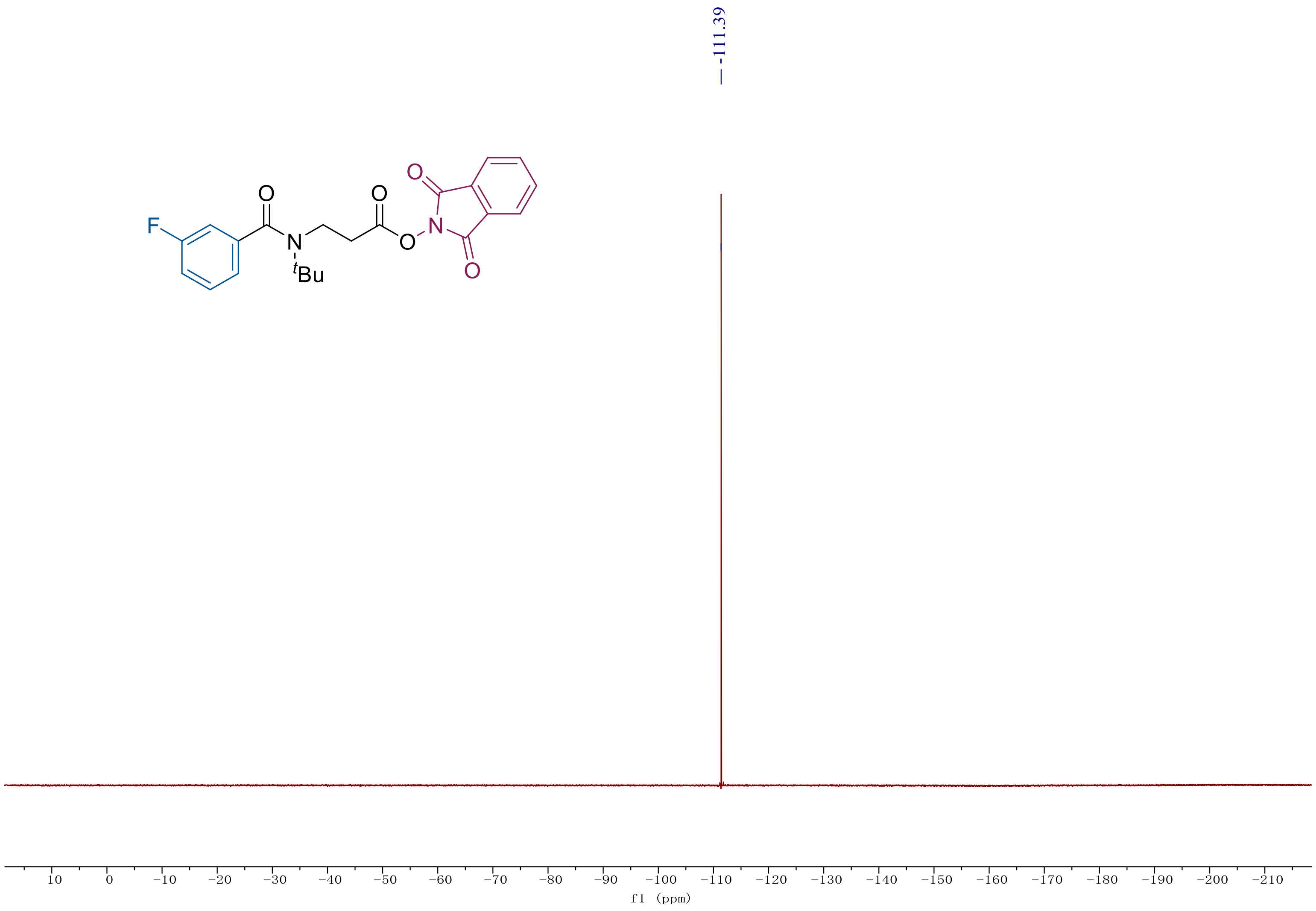

^{1}H NMR (400 MHz, $CDCl_3$) of **1aa**

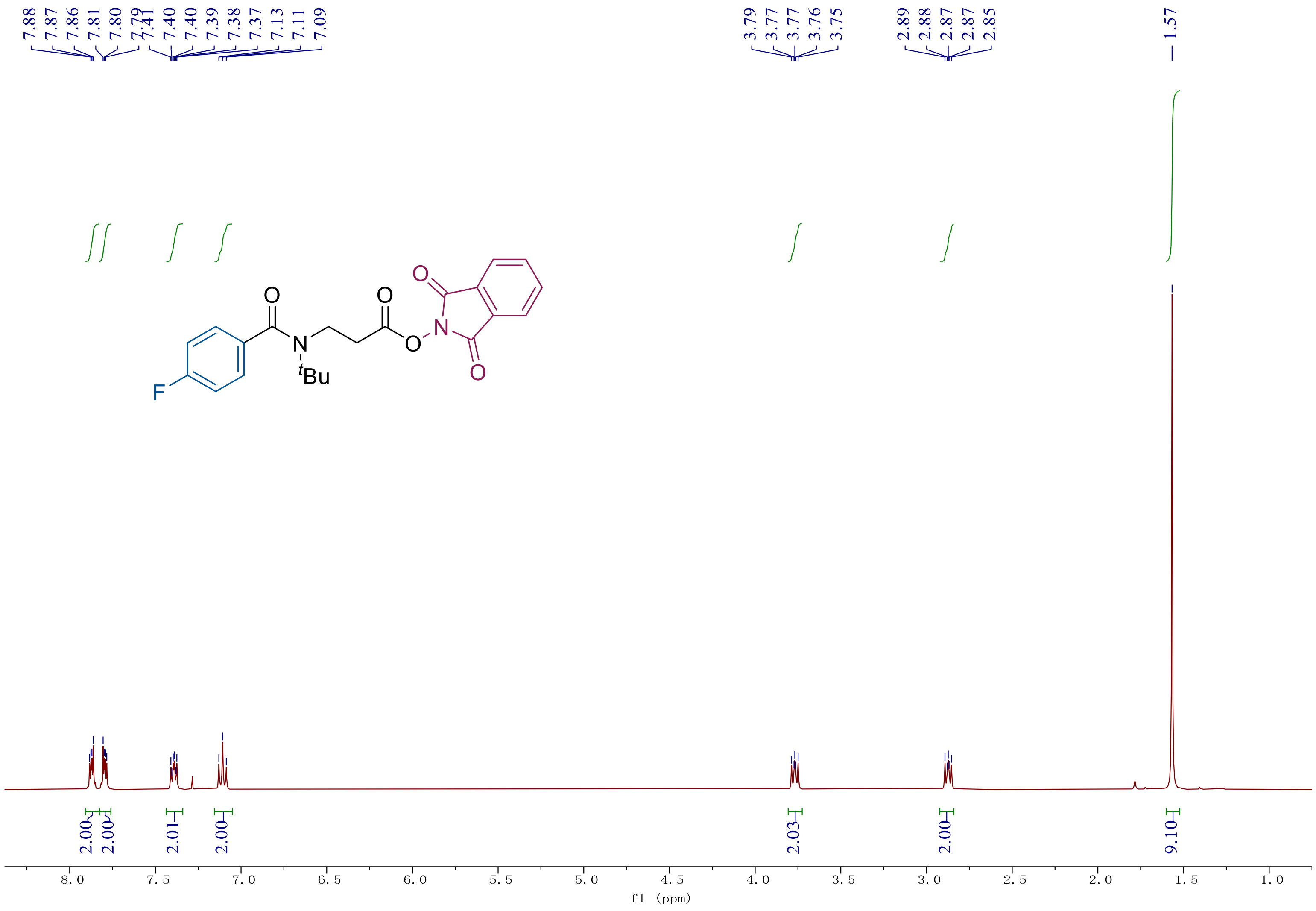

^{13}C NMR (101 MHz, $CDCl_3$) of **1aa**

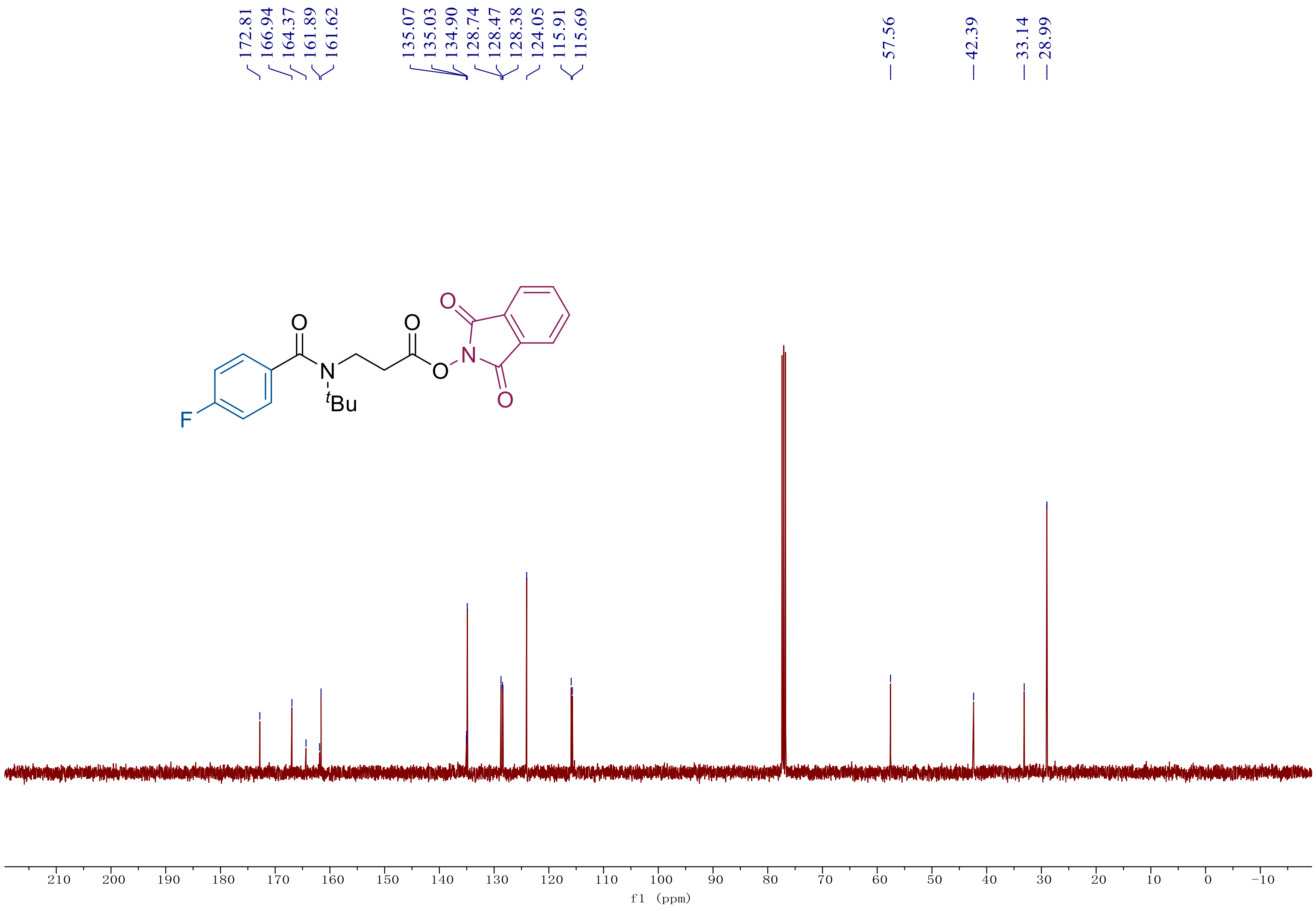

^{19}F NMR (376 MHz, CDCl$_3$) of **1aa**

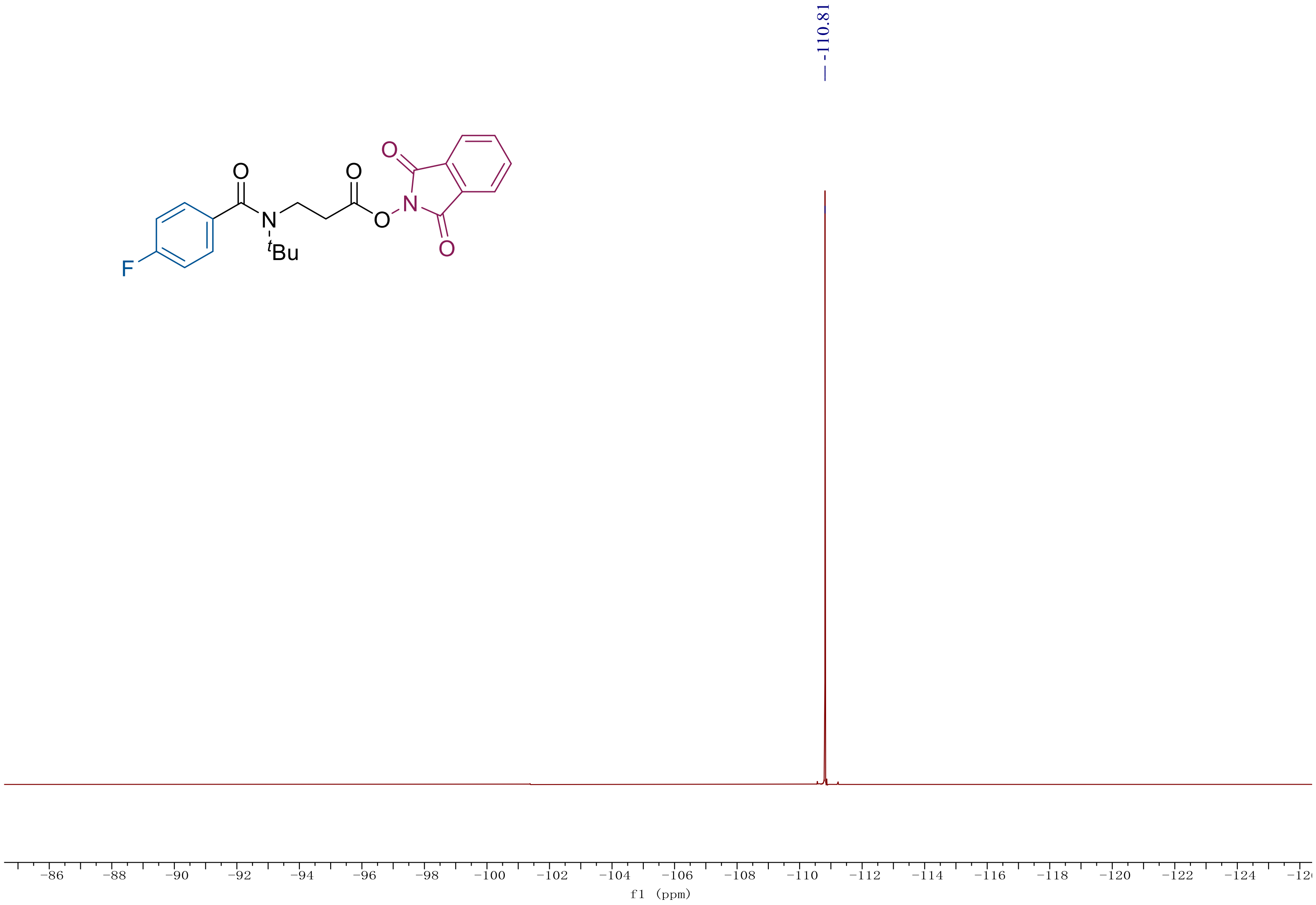

^{1}H NMR (400 MHz, CDCl$_3$) of **1ac**

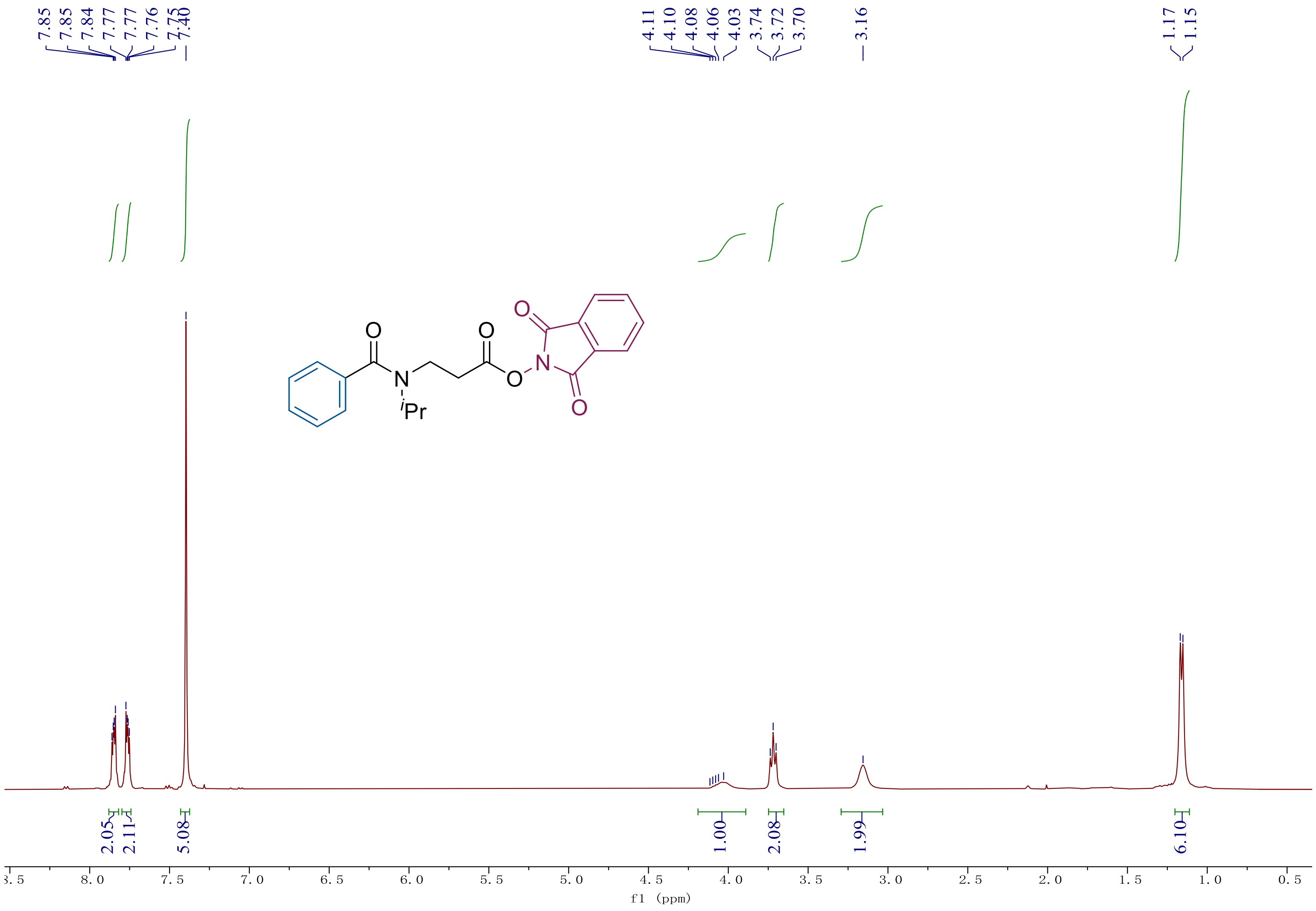

^{13}C NMR (101 MHz, $CDCl_3$) of **1ac**

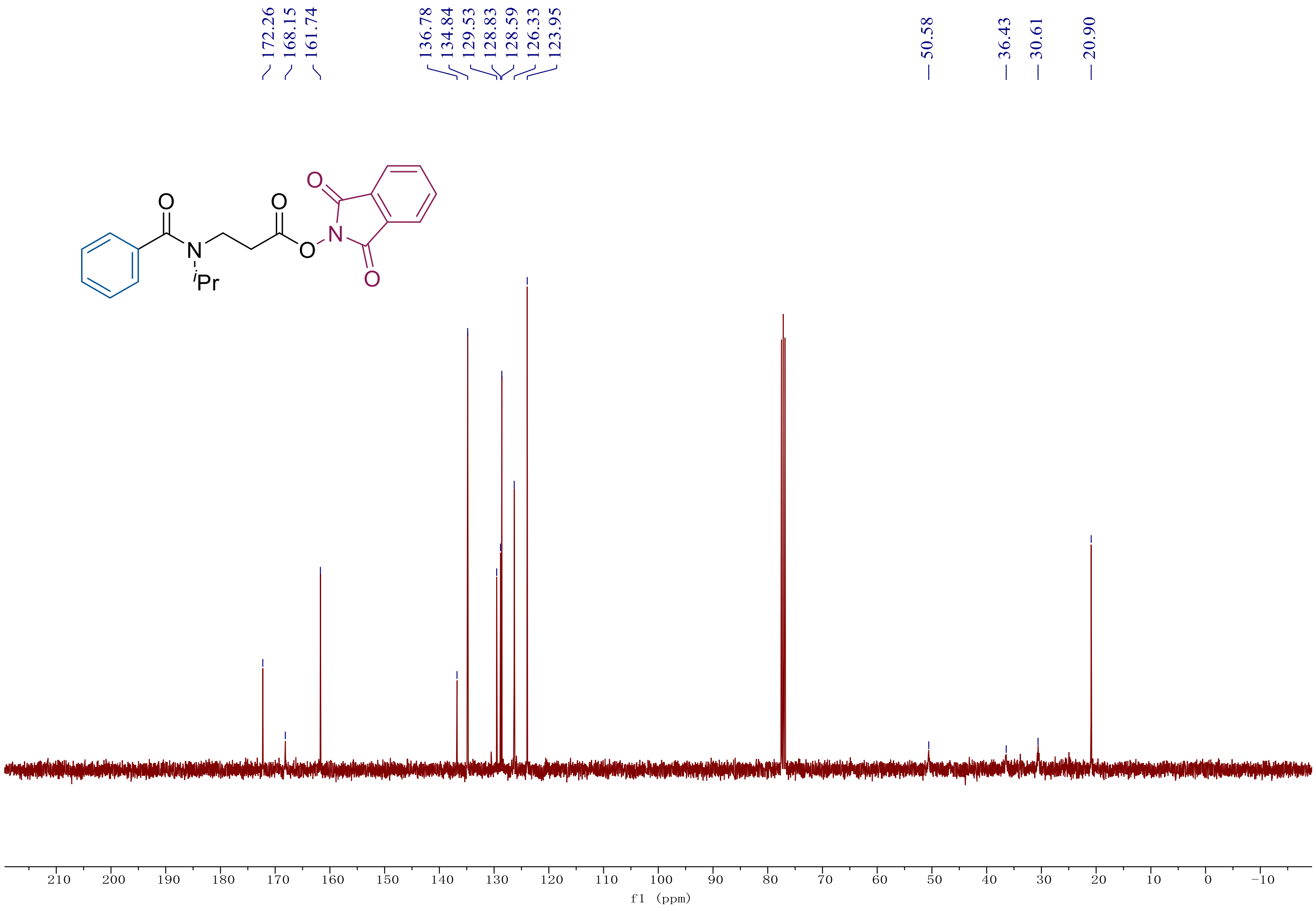

^{1}H NMR (500 MHz, $CDCl_3$) of **1ad**

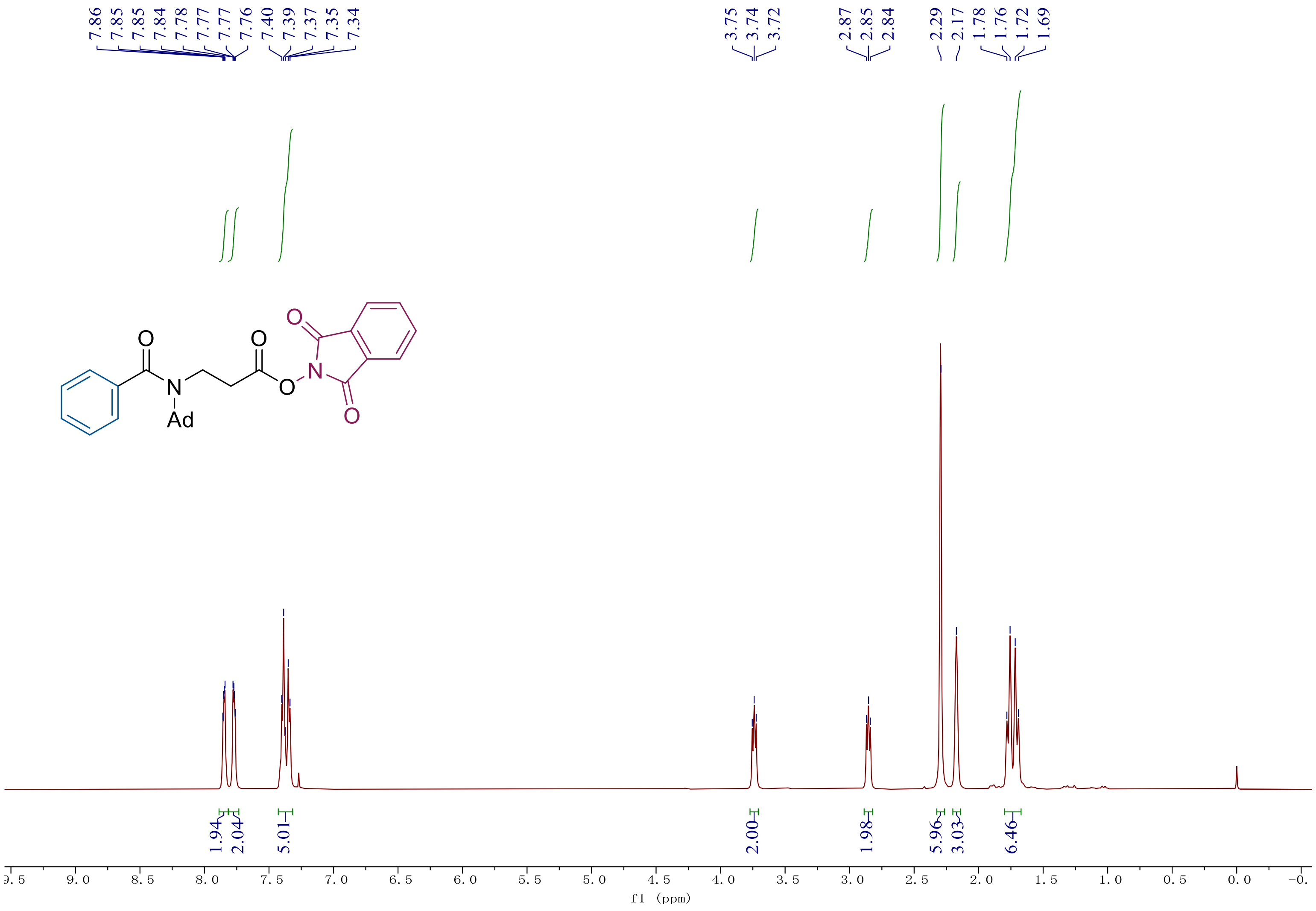

^{13}C NMR (126 MHz, $CDCl_3$) of **1ad**

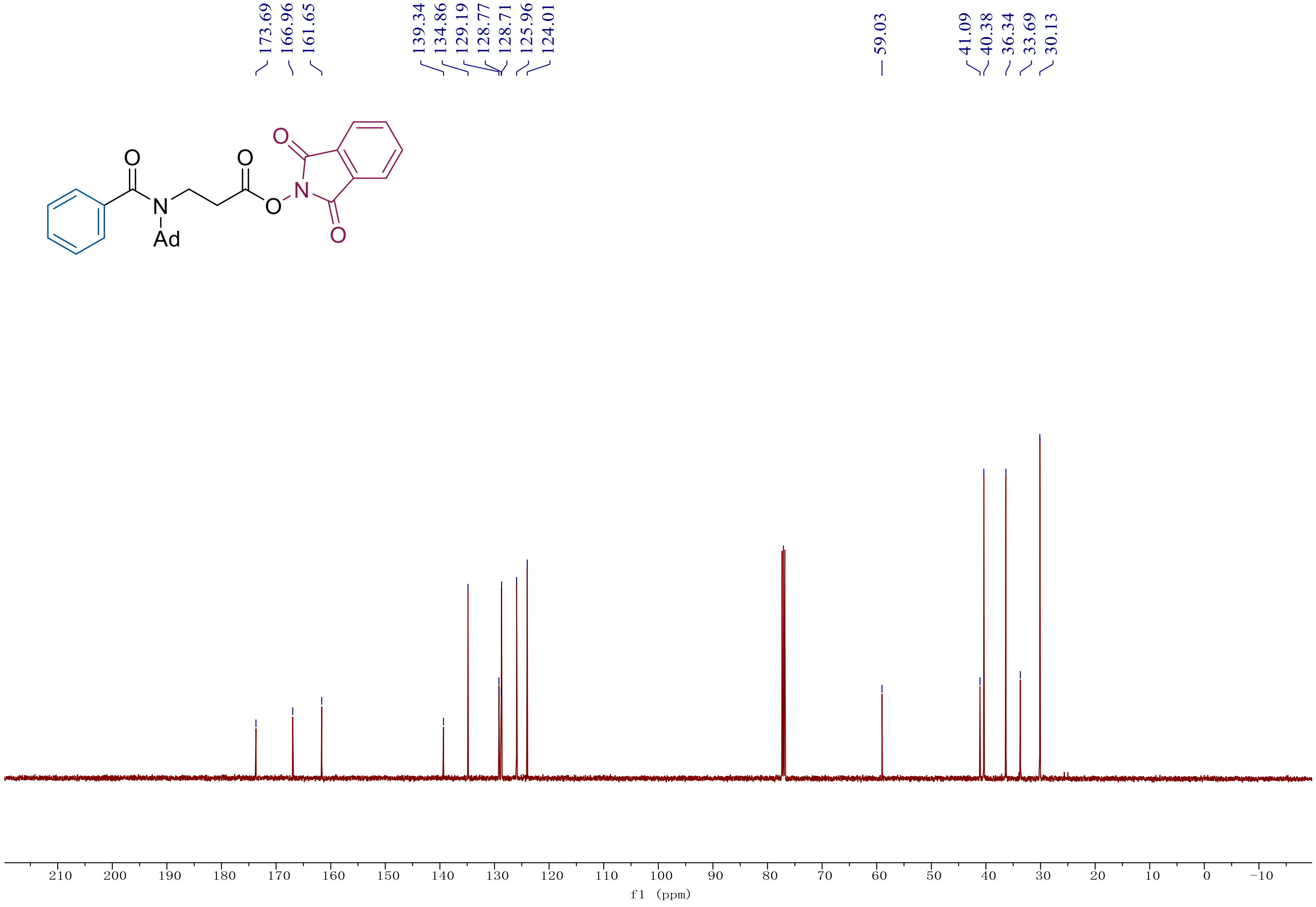

^{1}H NMR (400 MHz, $CDCl_3$) of **1ae**

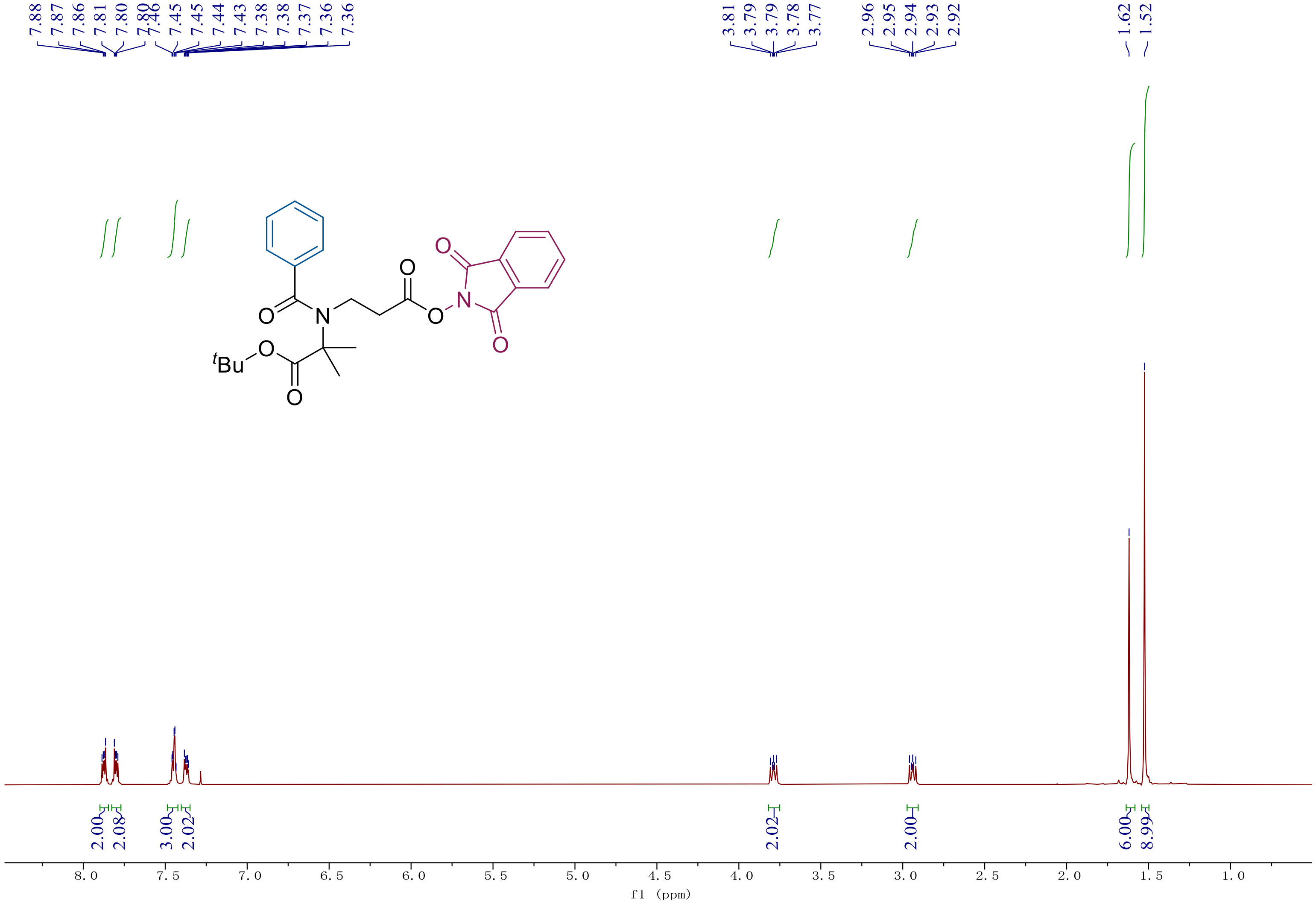

^{13}C NMR (101 MHz, $CDCl_3$) of **1ae**

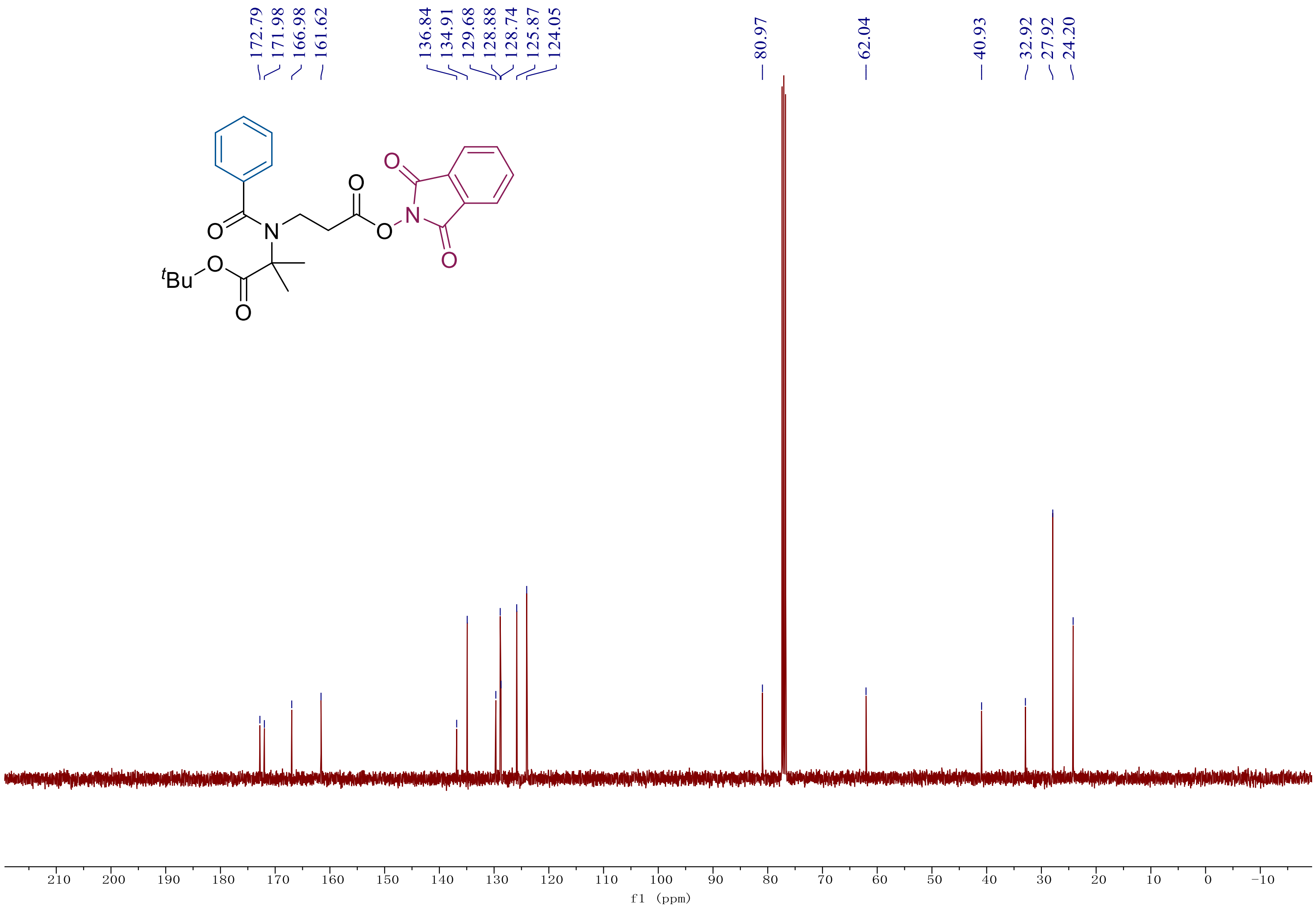

^{1}H NMR (400 MHz, CDCl$_3$) of **1af**

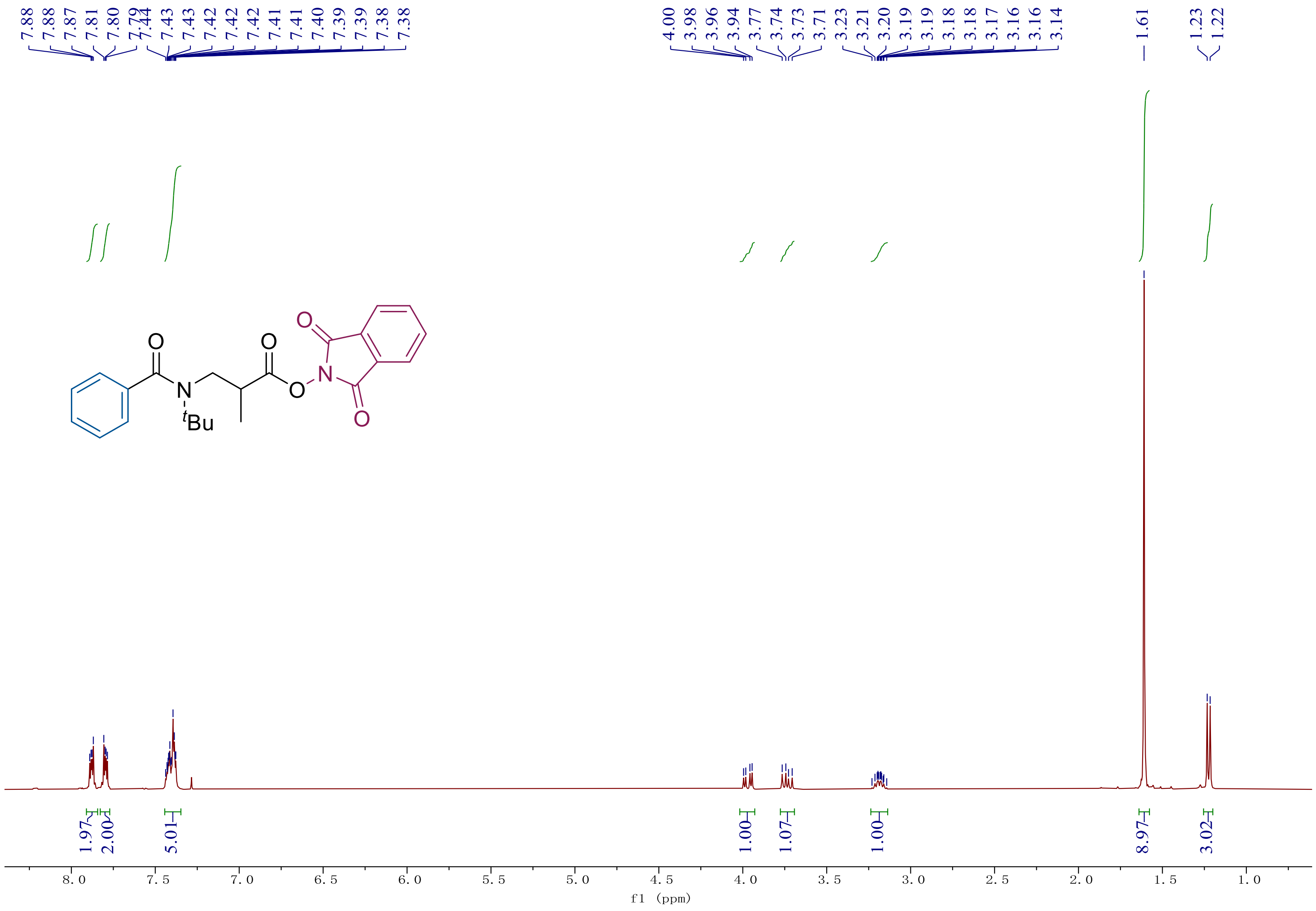

^{13}C NMR (101 MHz, CDCl$_3$) of **1af**

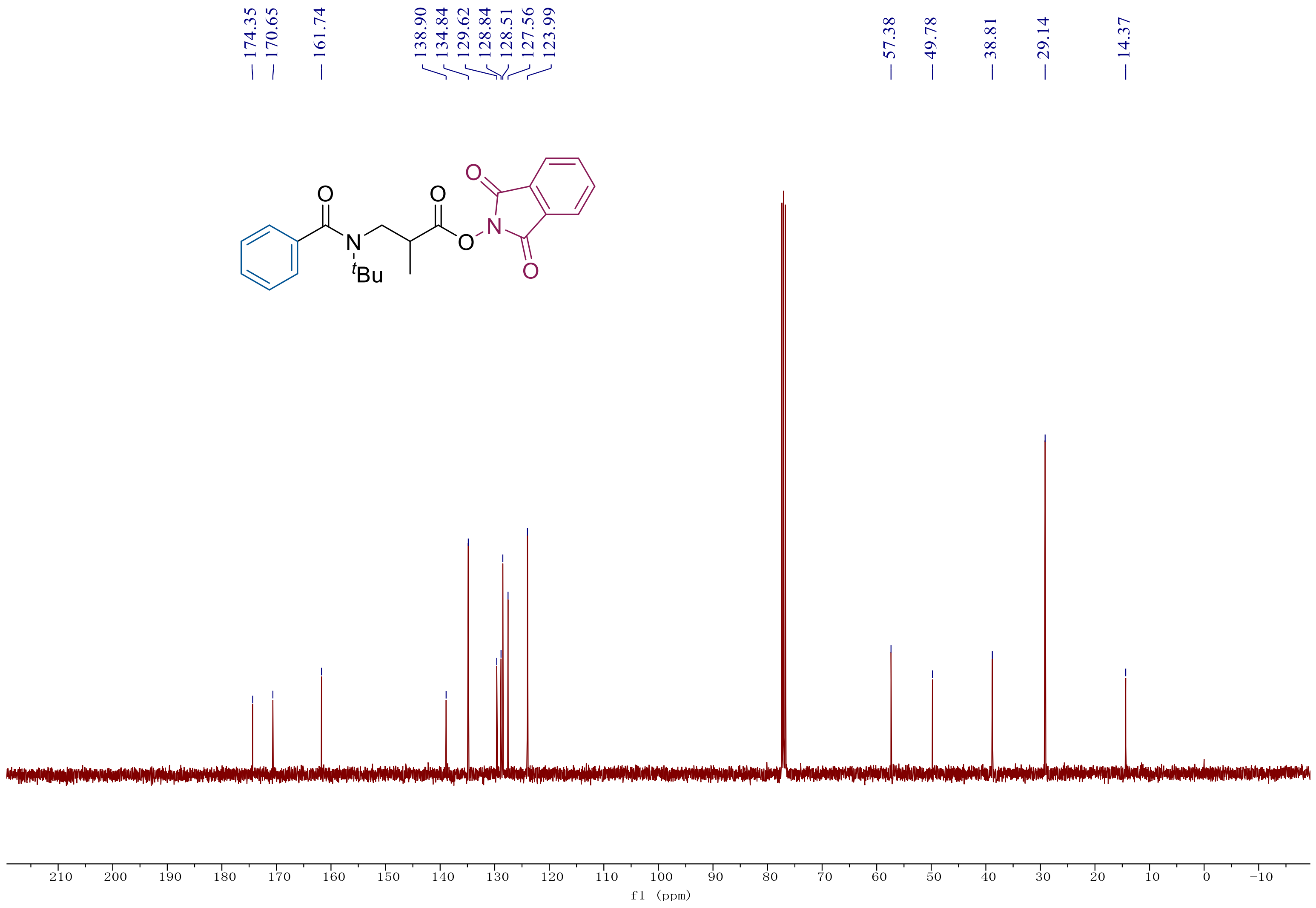

^{1}H NMR (400 MHz, $CDCl_3$) of **1ag**

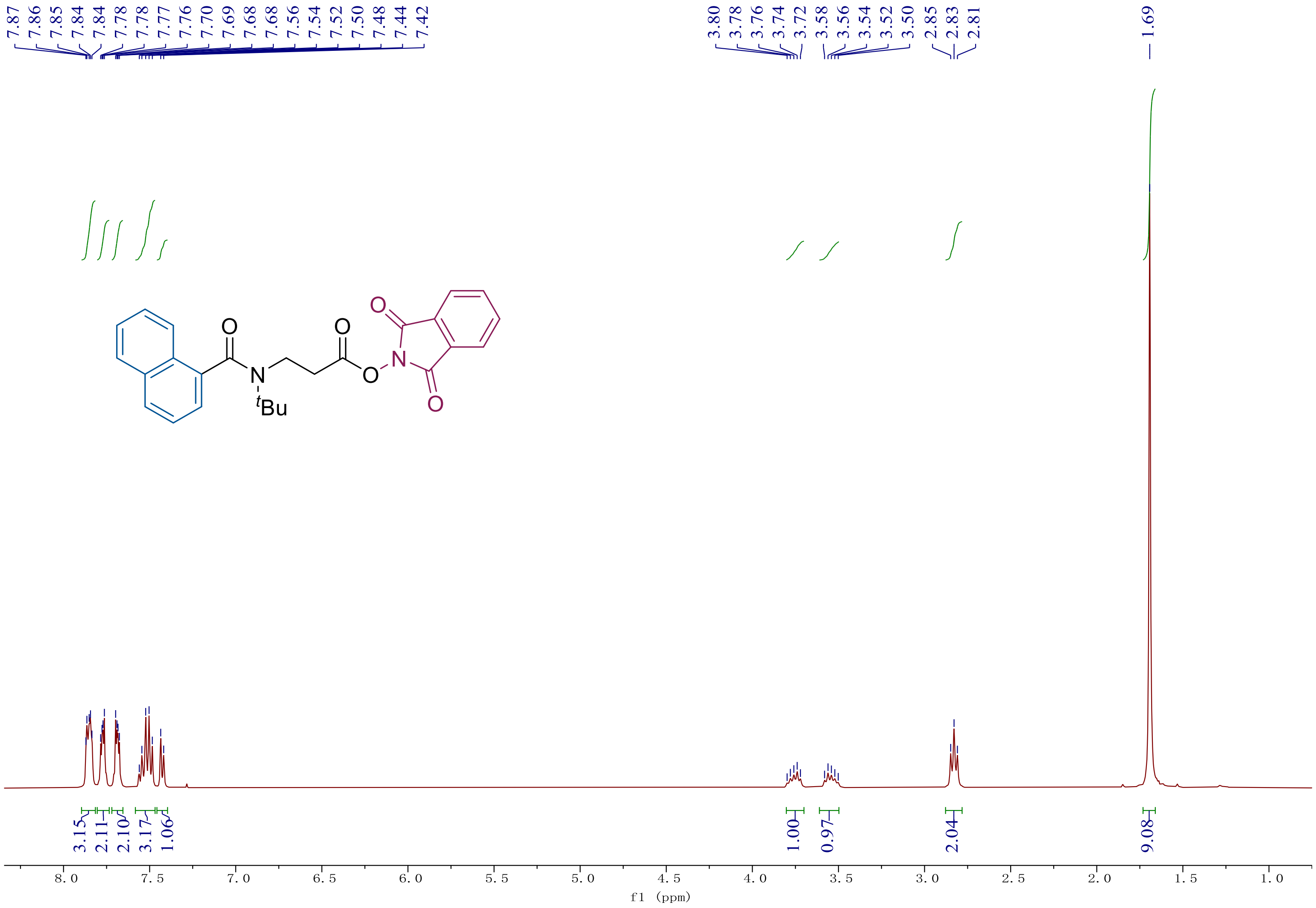

^{13}C NMR (101 MHz, $CDCl_3$) of **1ag**

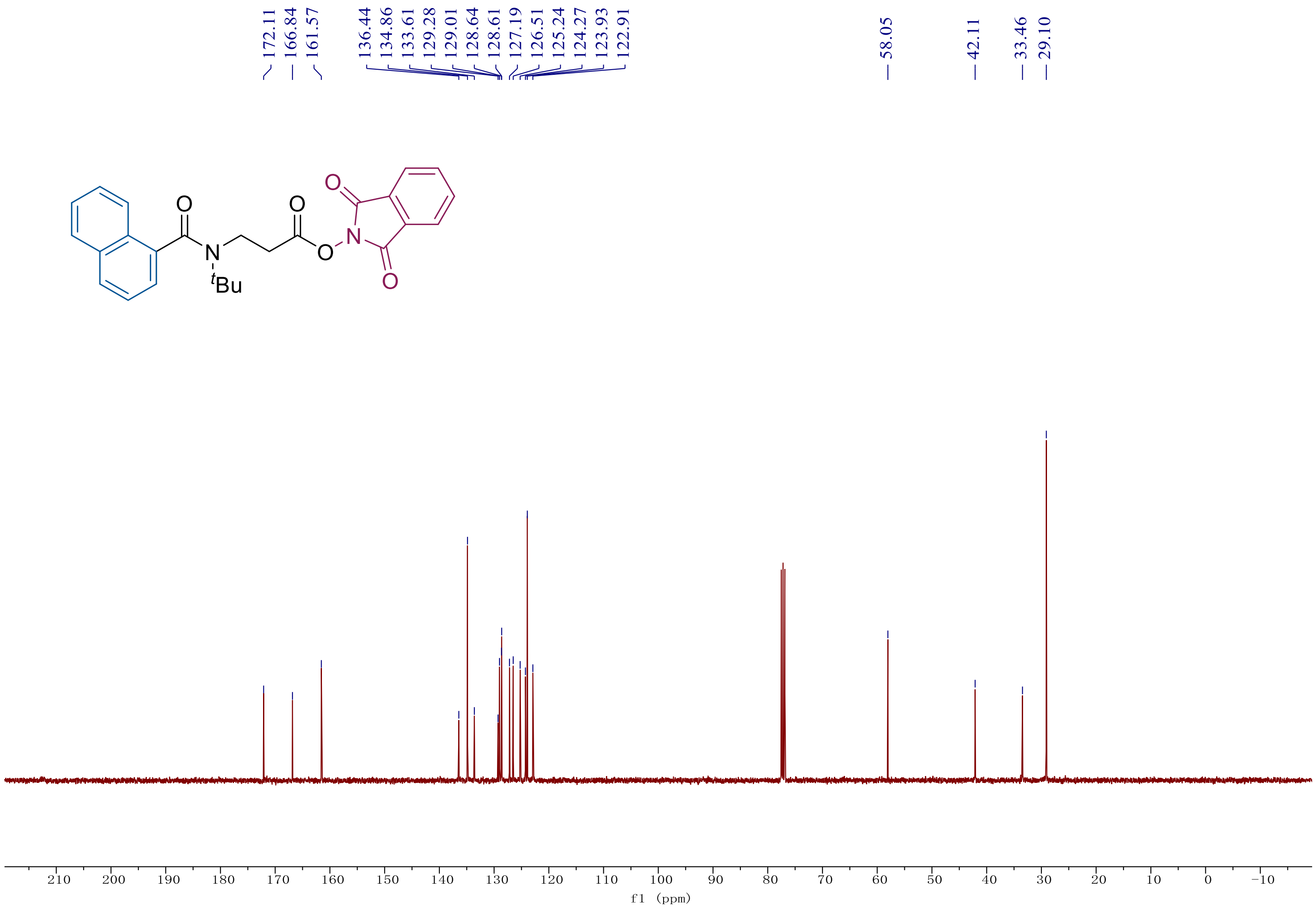

^{1}H NMR (400 MHz, $CDCl_3$) of **1ah**

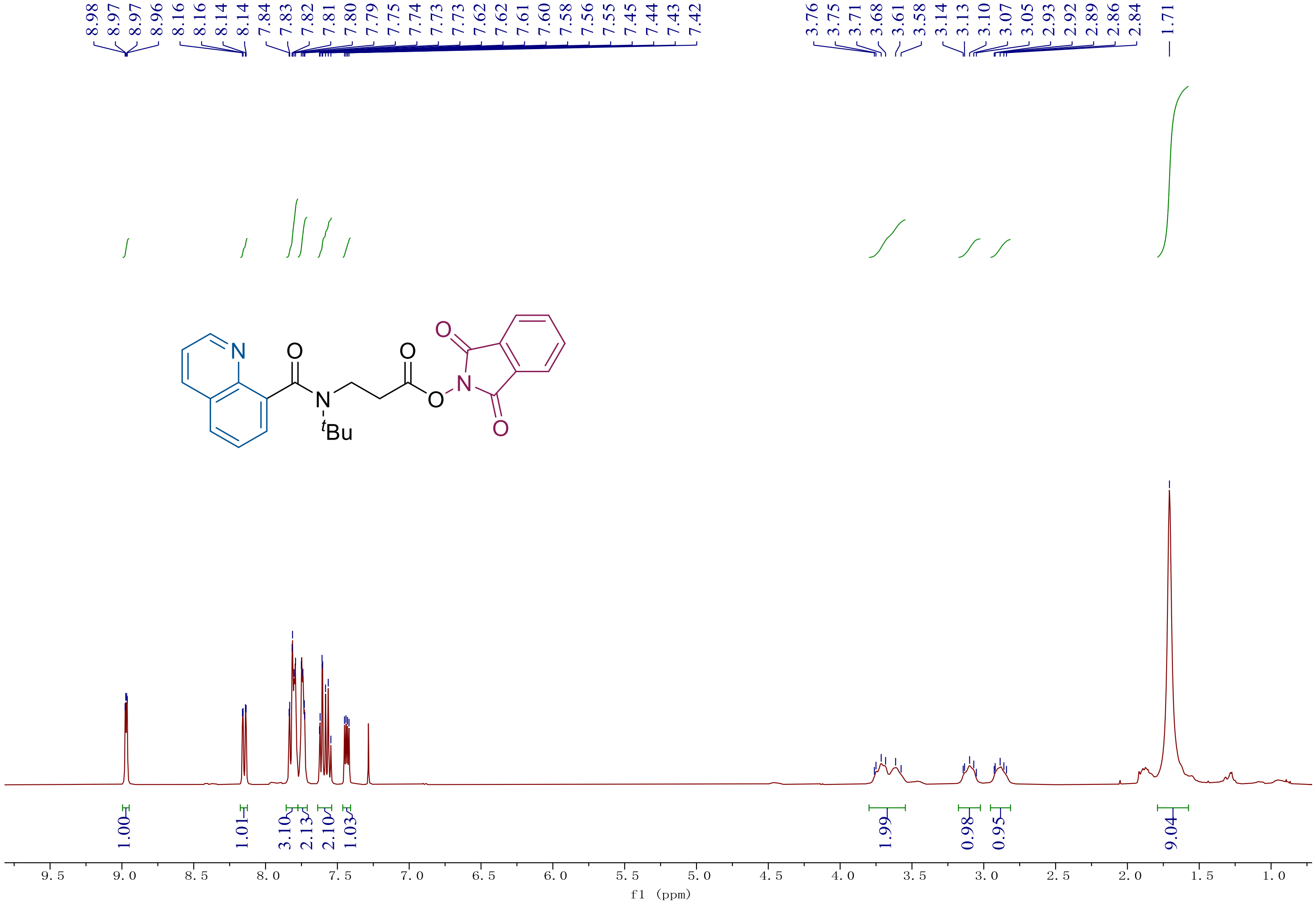

^{13}C NMR (101 MHz, $CDCl_3$) of **1ah**

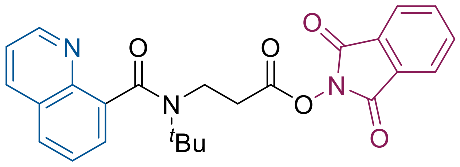

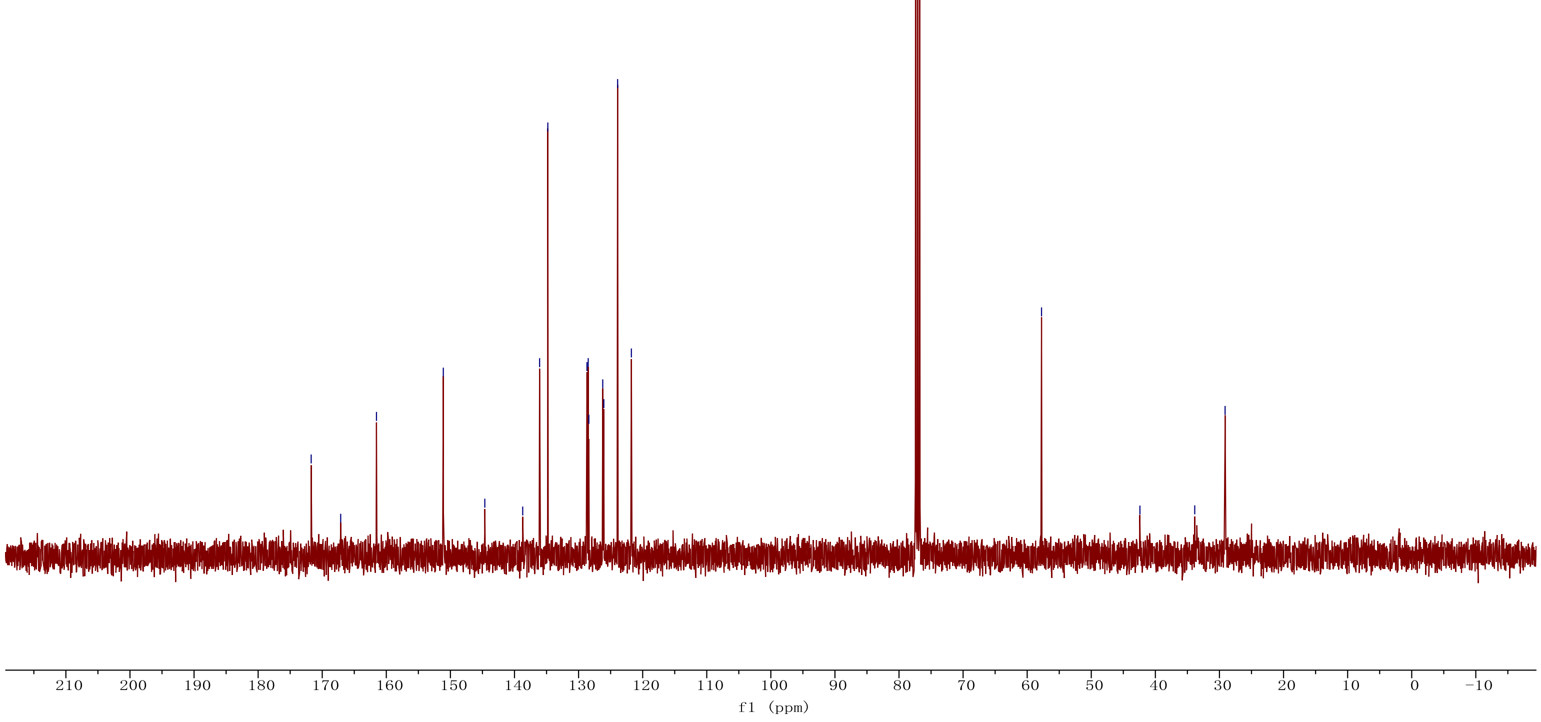

^{1}H NMR (500 MHz, $CDCl_3$) of **1ai**

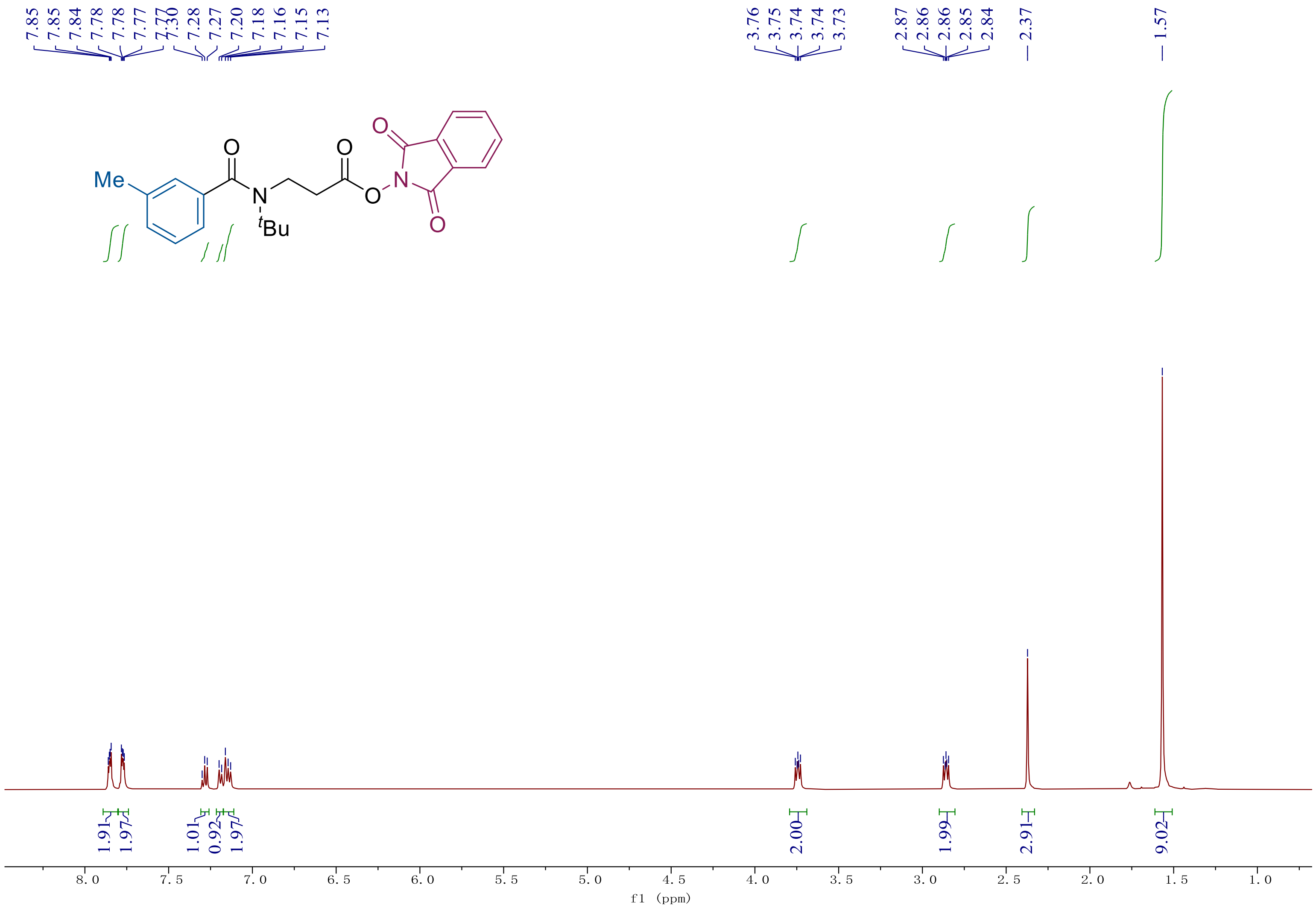

^{13}C NMR (126 MHz, CDCl$_3$) of **1ai**

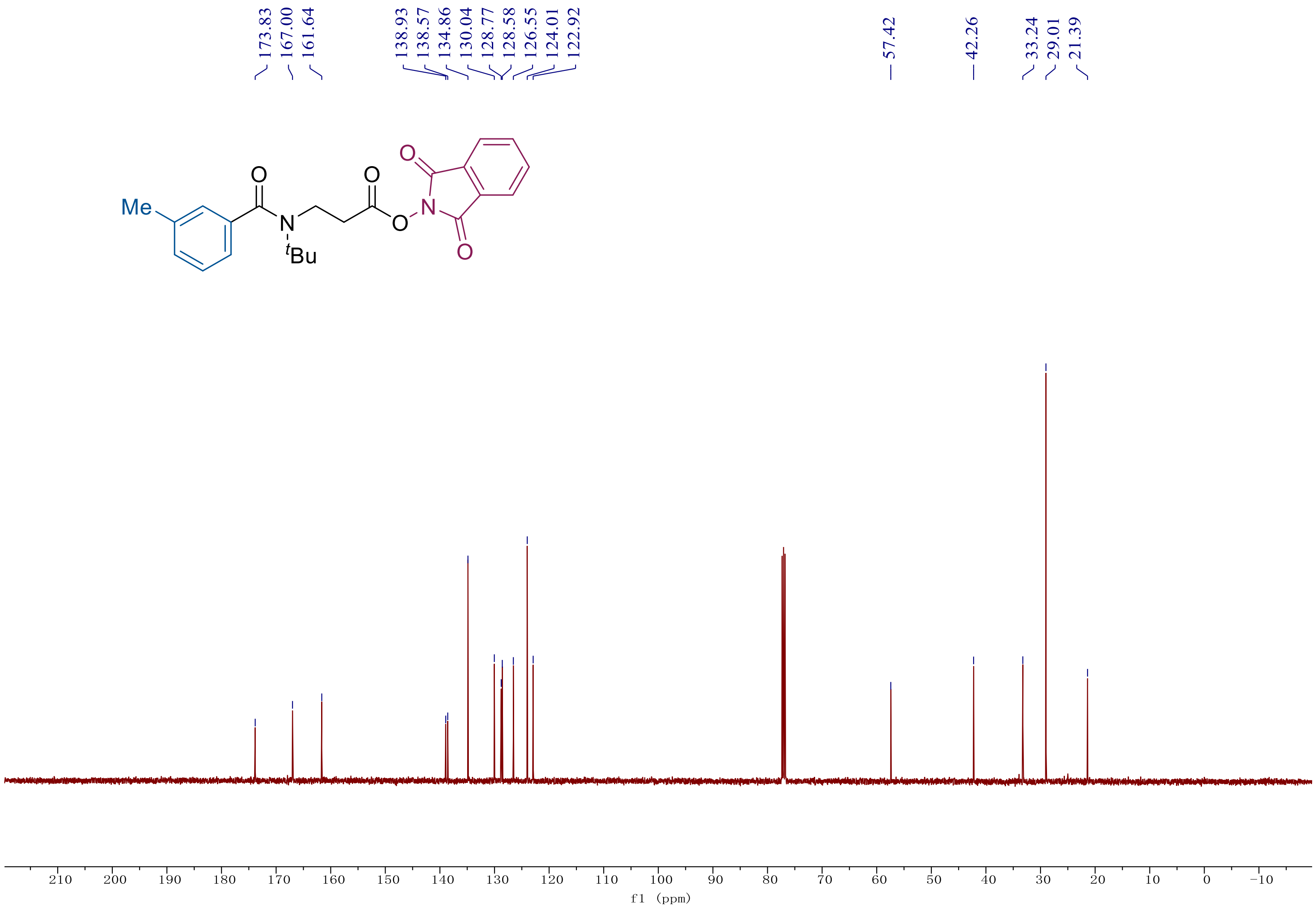

^{1}H NMR (400 MHz, $CDCl_3$) of **1aj**

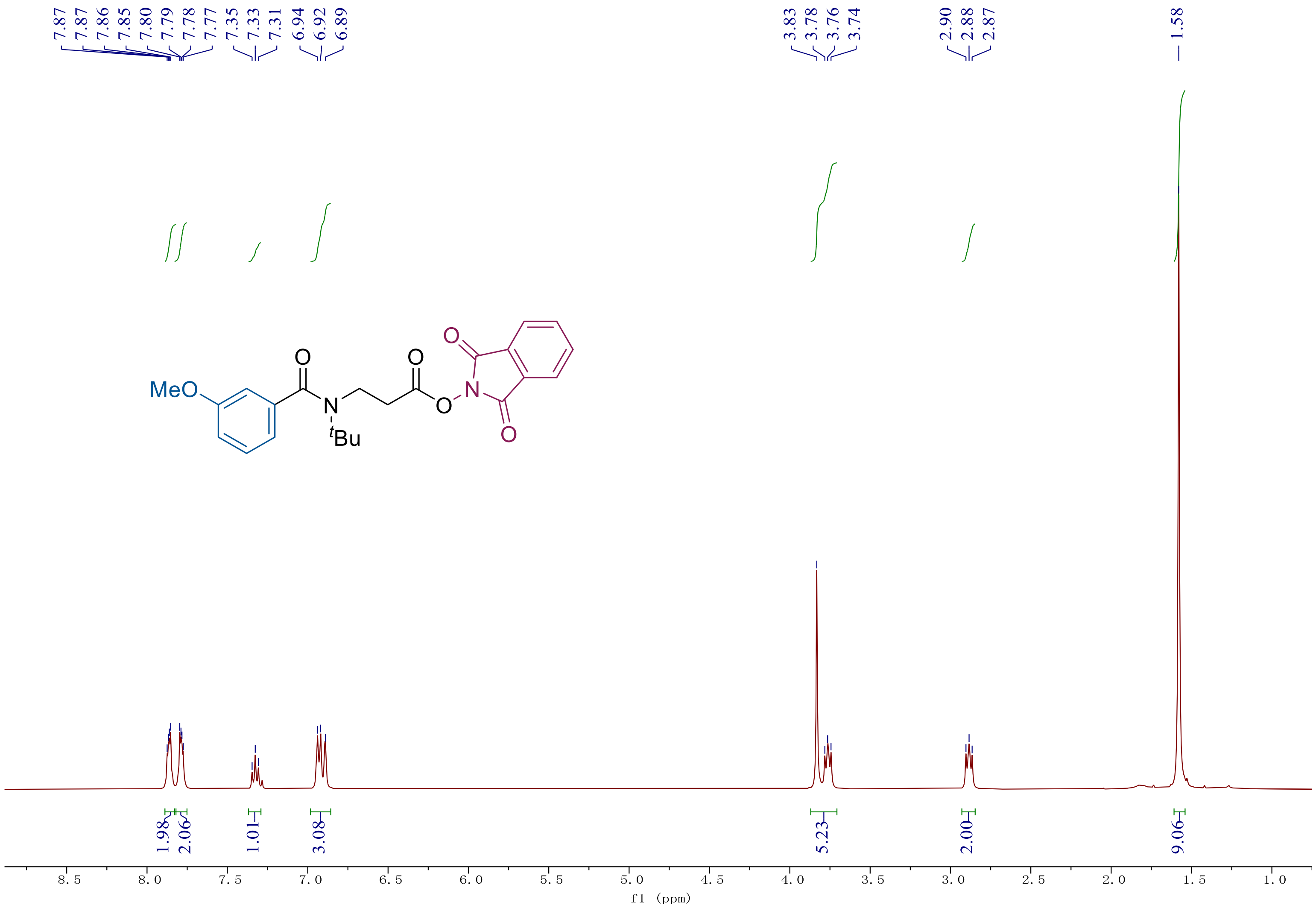

^{13}C NMR (101 MHz, $CDCl_3$) of **1aj**

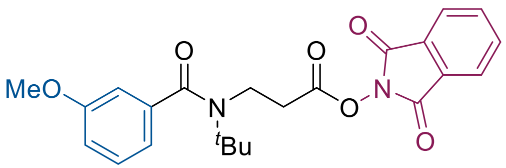

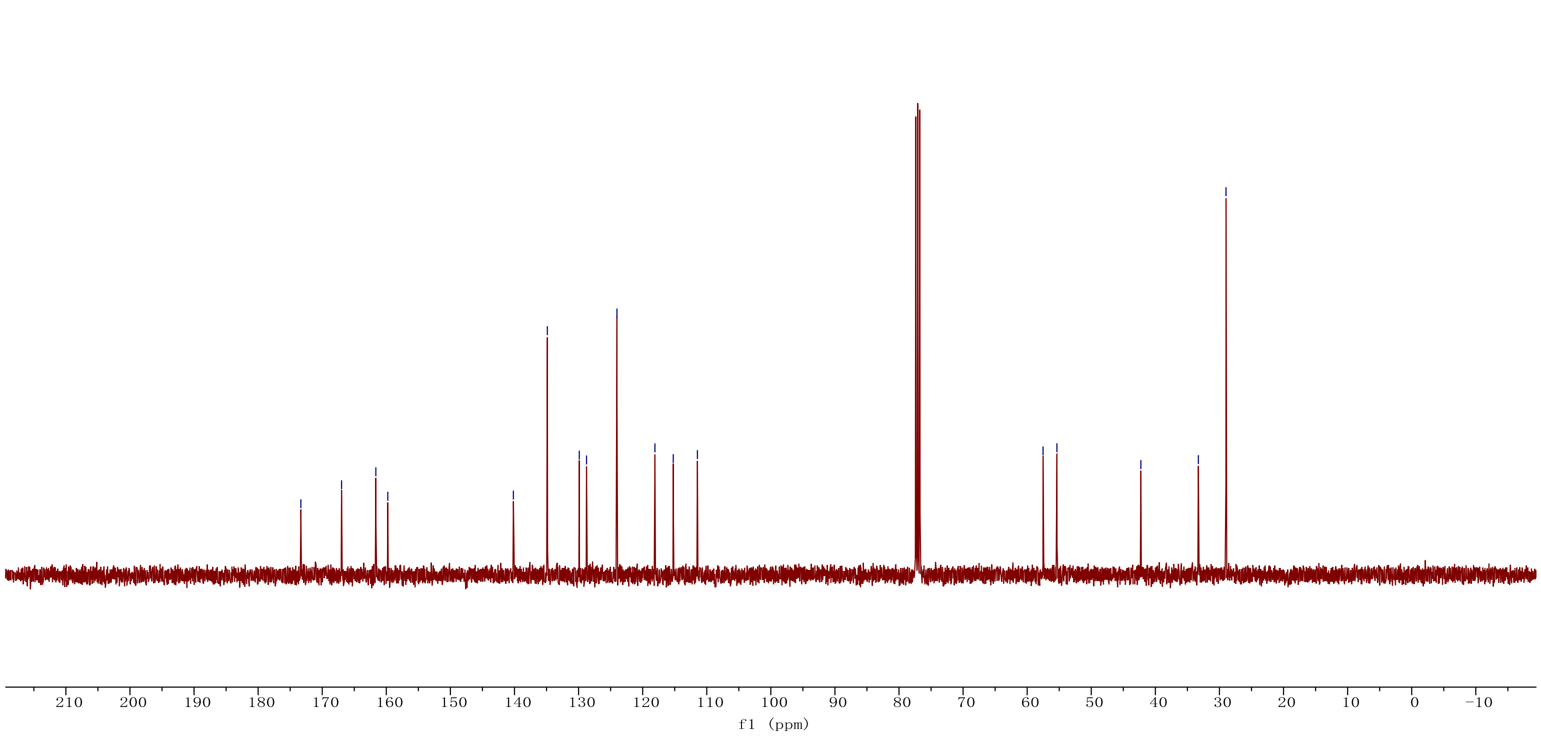

^{1}H NMR (400 MHz, $CDCl_3$) of **1ak**

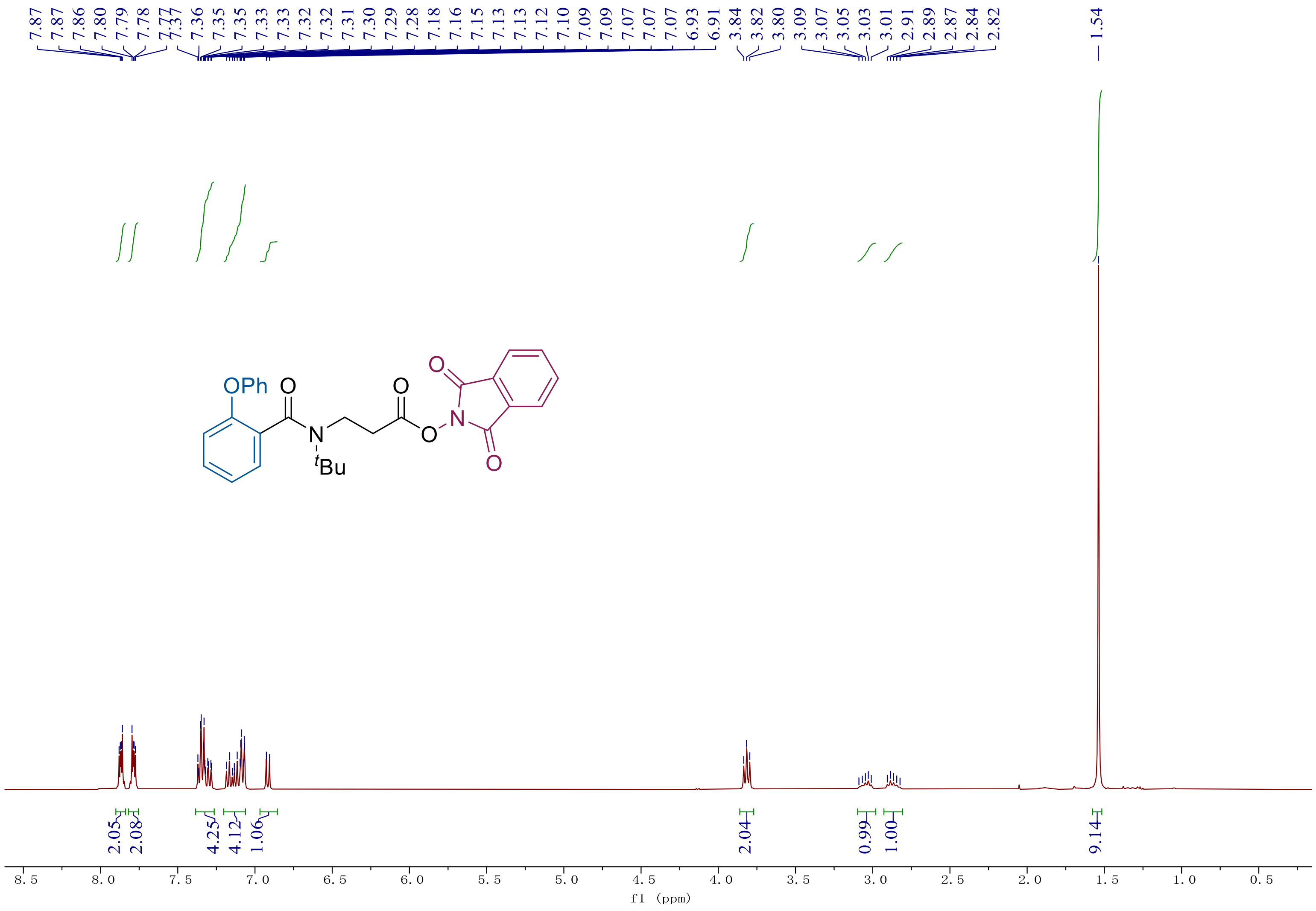

^{13}C NMR (101 MHz, $CDCl_3$) of **1ak**

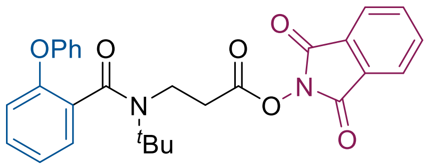

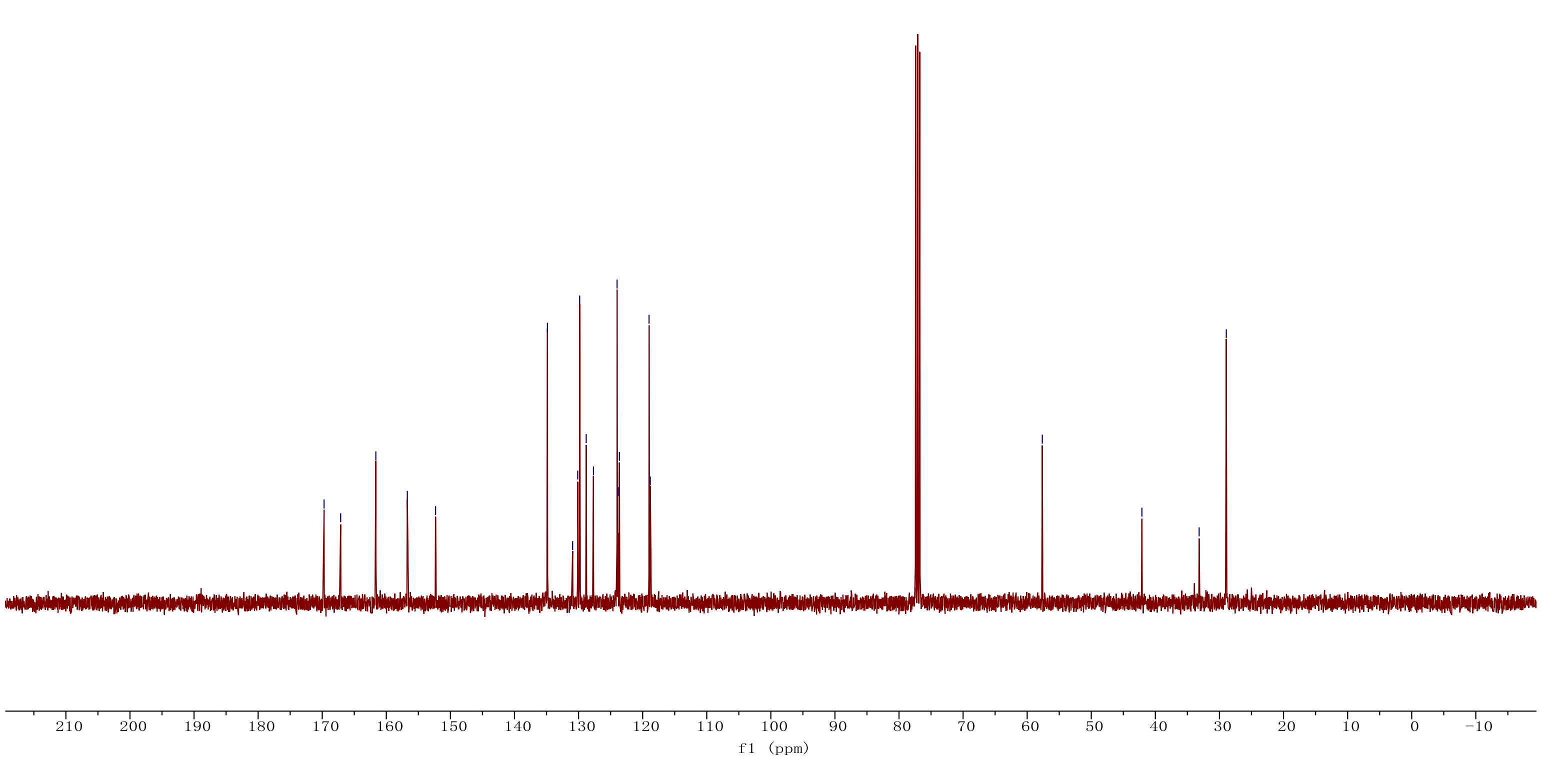

^{1}H NMR (400 MHz, $CDCl_3$) of **1al**

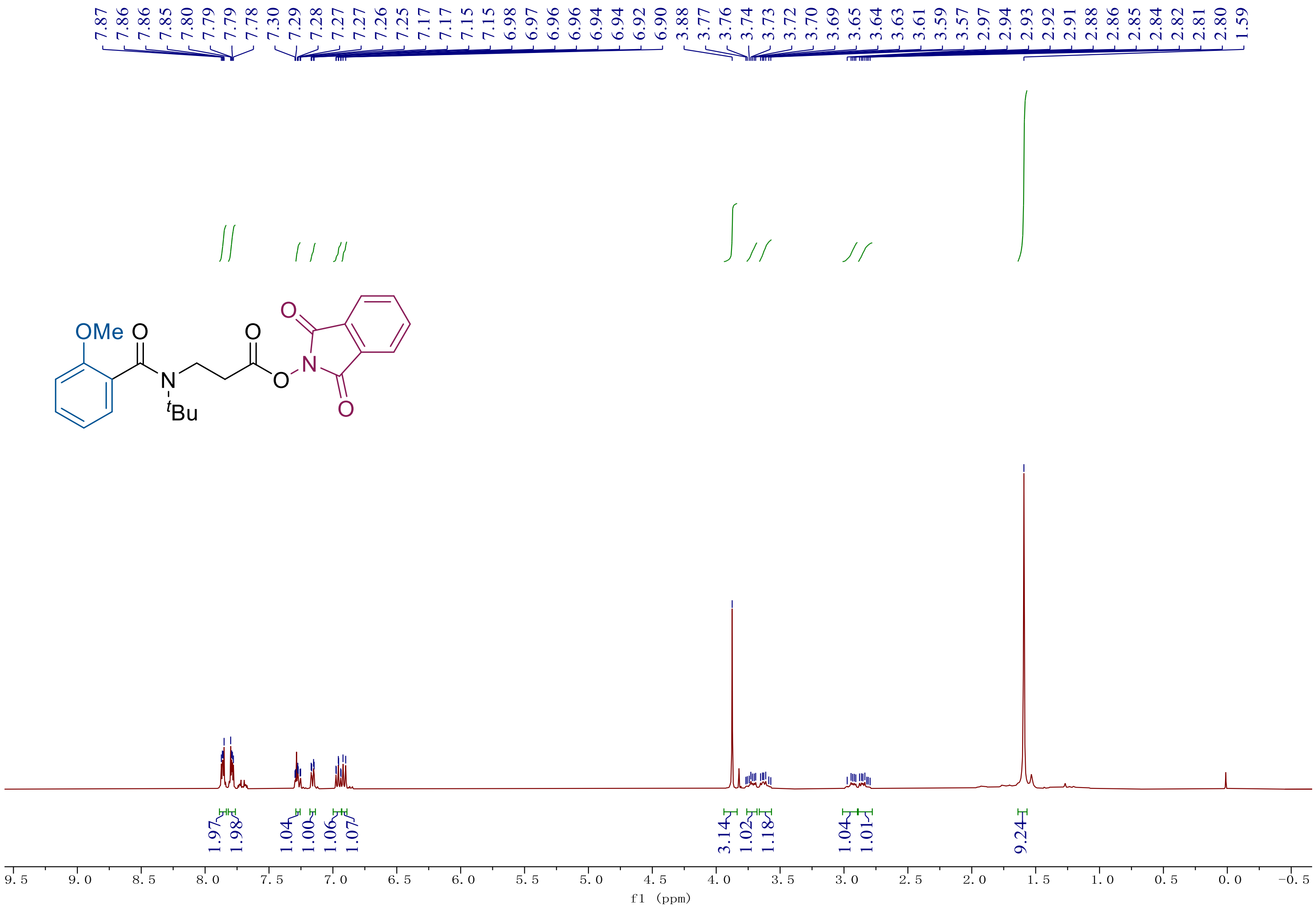

^{13}C NMR (101 MHz, $CDCl_3$) of **1al**

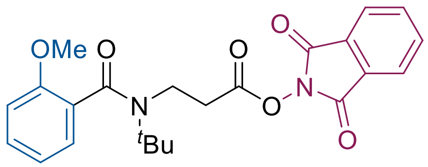

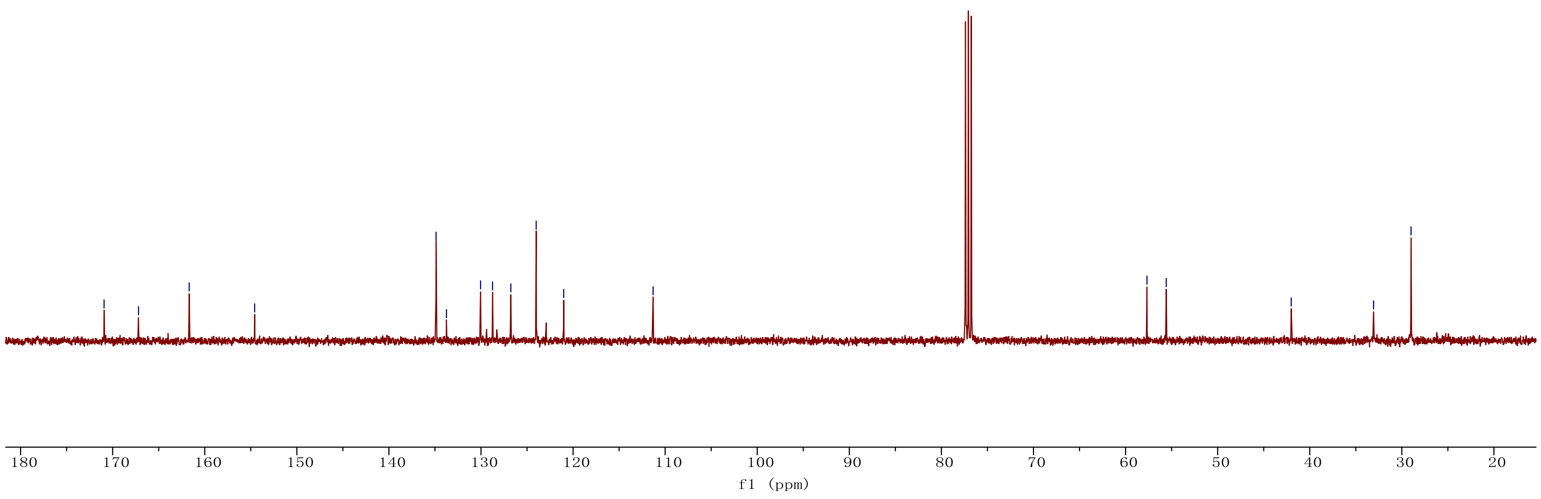

^{1}H NMR (400 MHz, $CDCl_3$) of **1am**

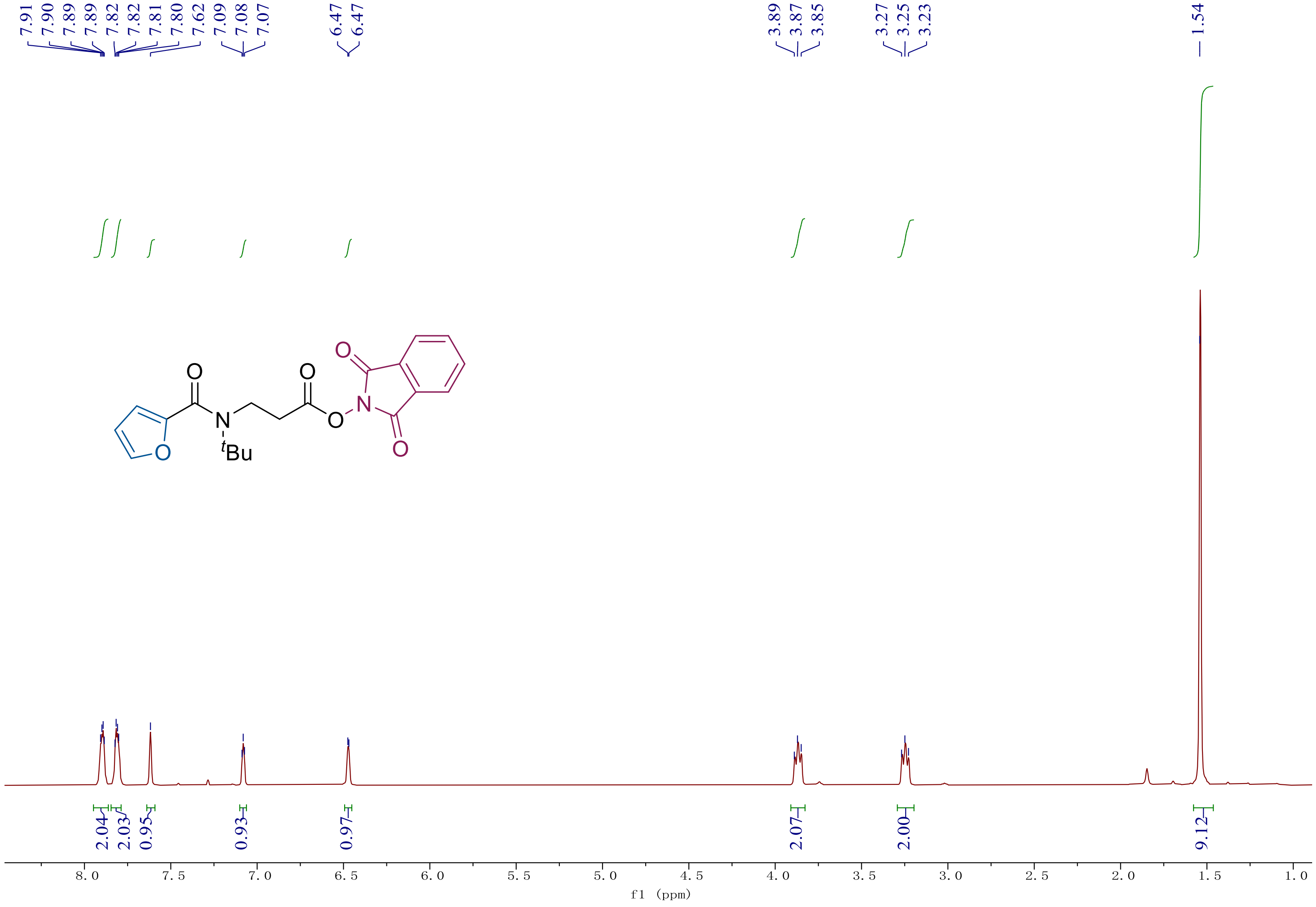

^{13}C NMR (101 MHz, $CDCl_3$) of **1am**

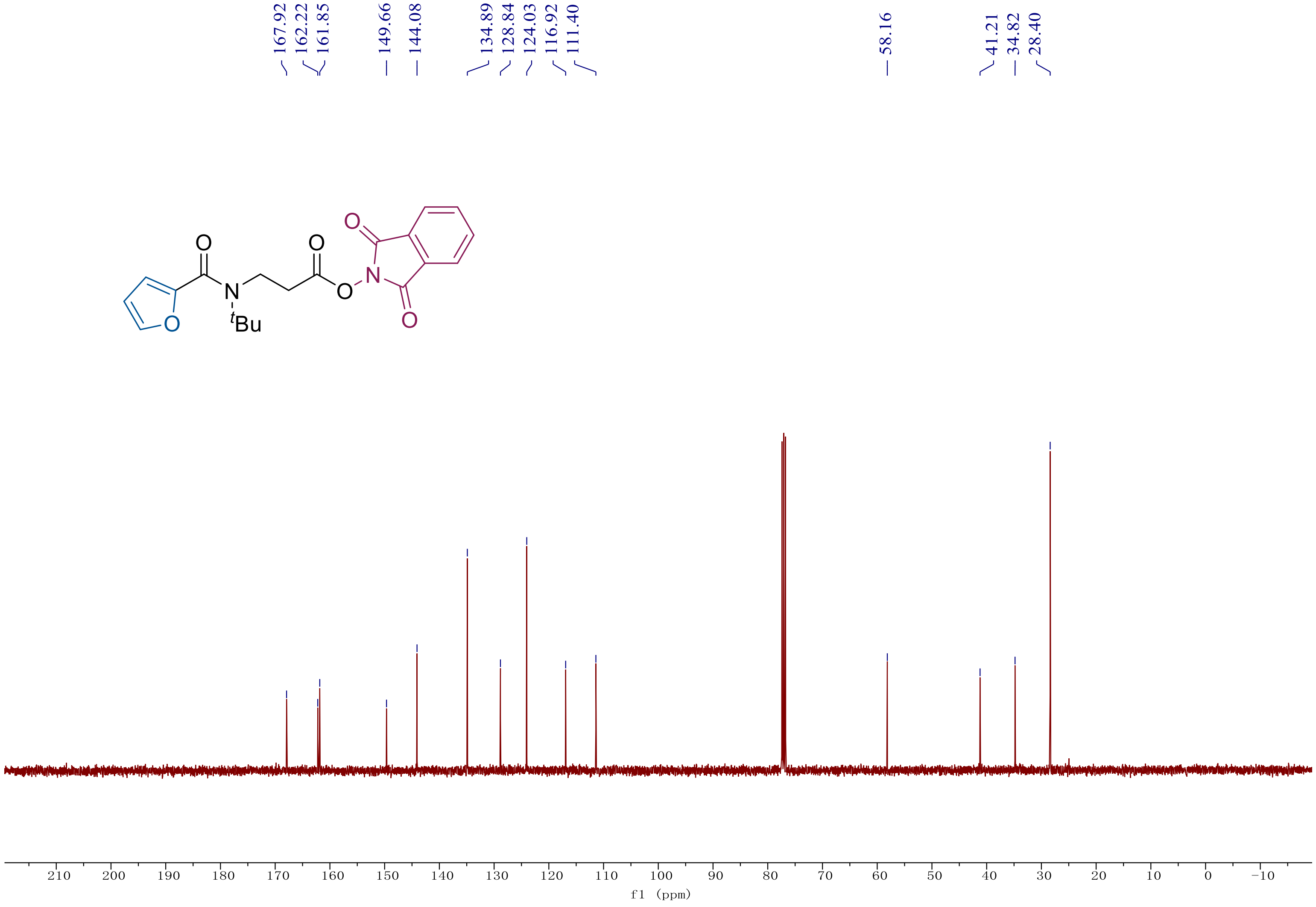

^{1}H NMR (400 MHz, $CDCl_3$) of **1an**

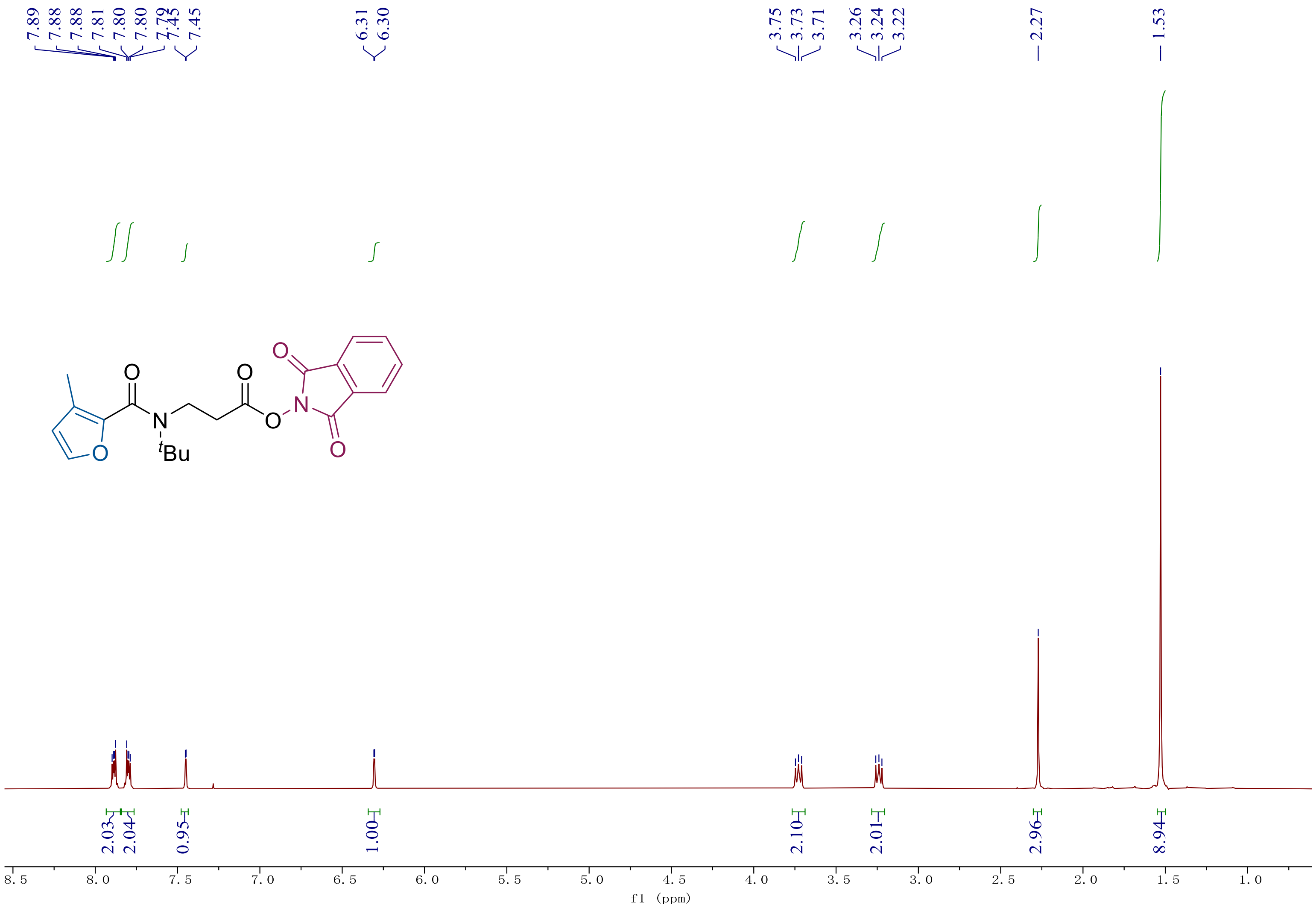

^{13}C NMR (101 MHz, $CDCl_3$) of **1an**

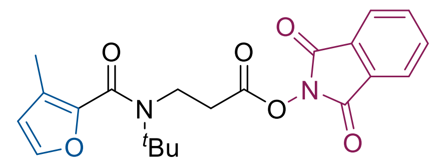

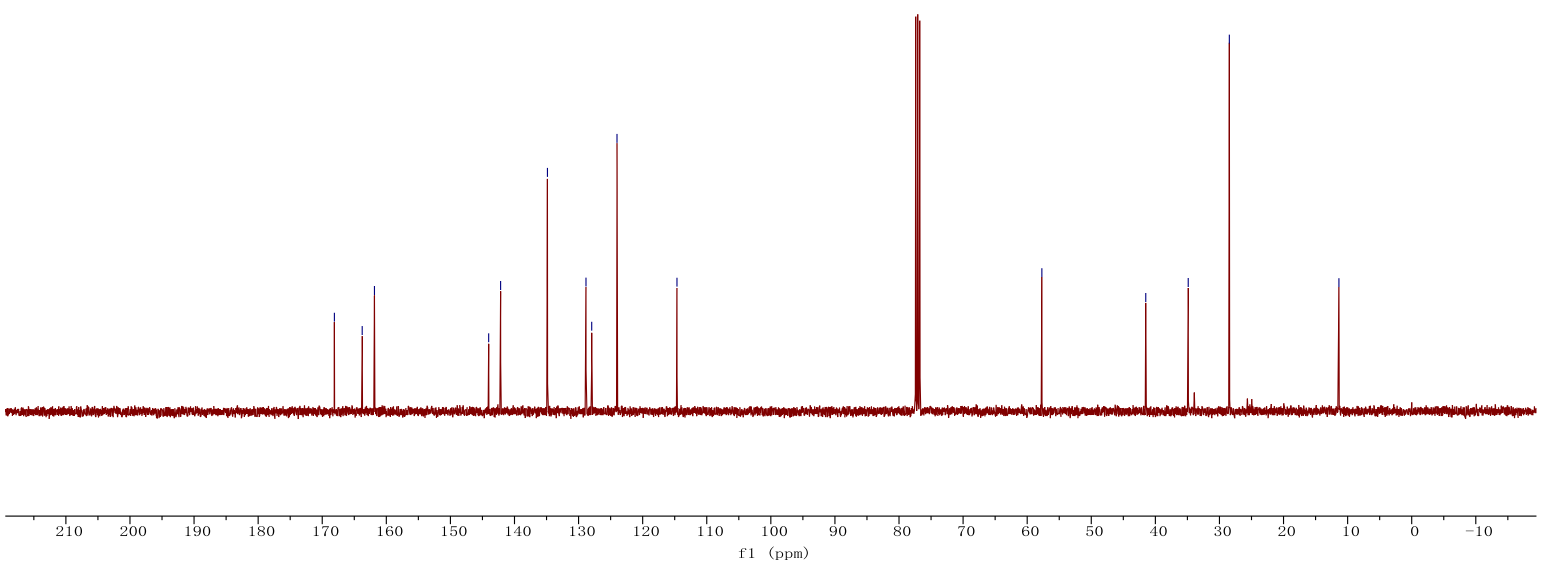

^{1}H NMR (500 MHz, $CDCl_3$) of **1ao**

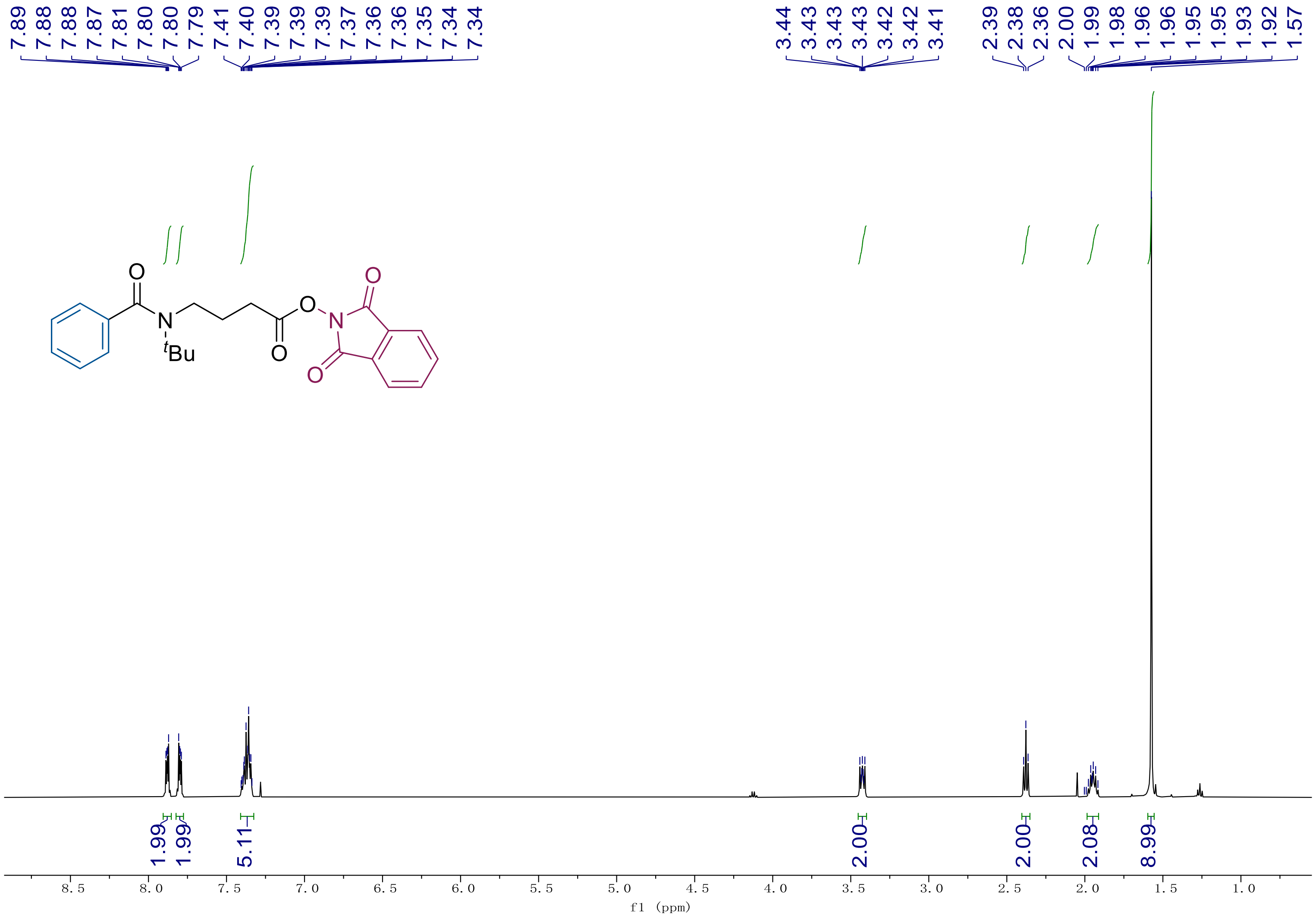

^{13}C NMR (126 MHz, $CDCl_3$) of **1ao**

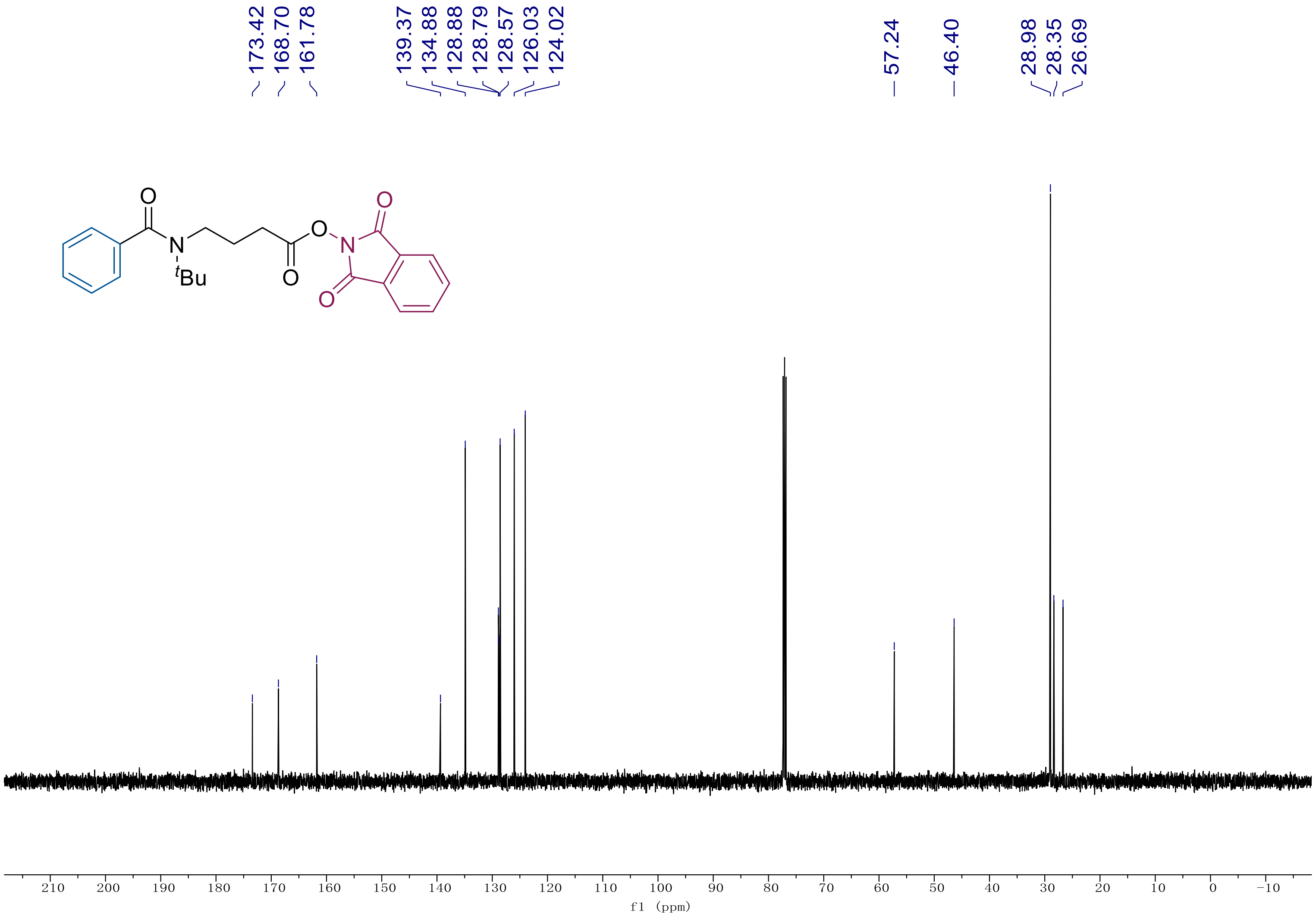

^1H NMR (400 MHz, $CDCl_3$) of **1ap**

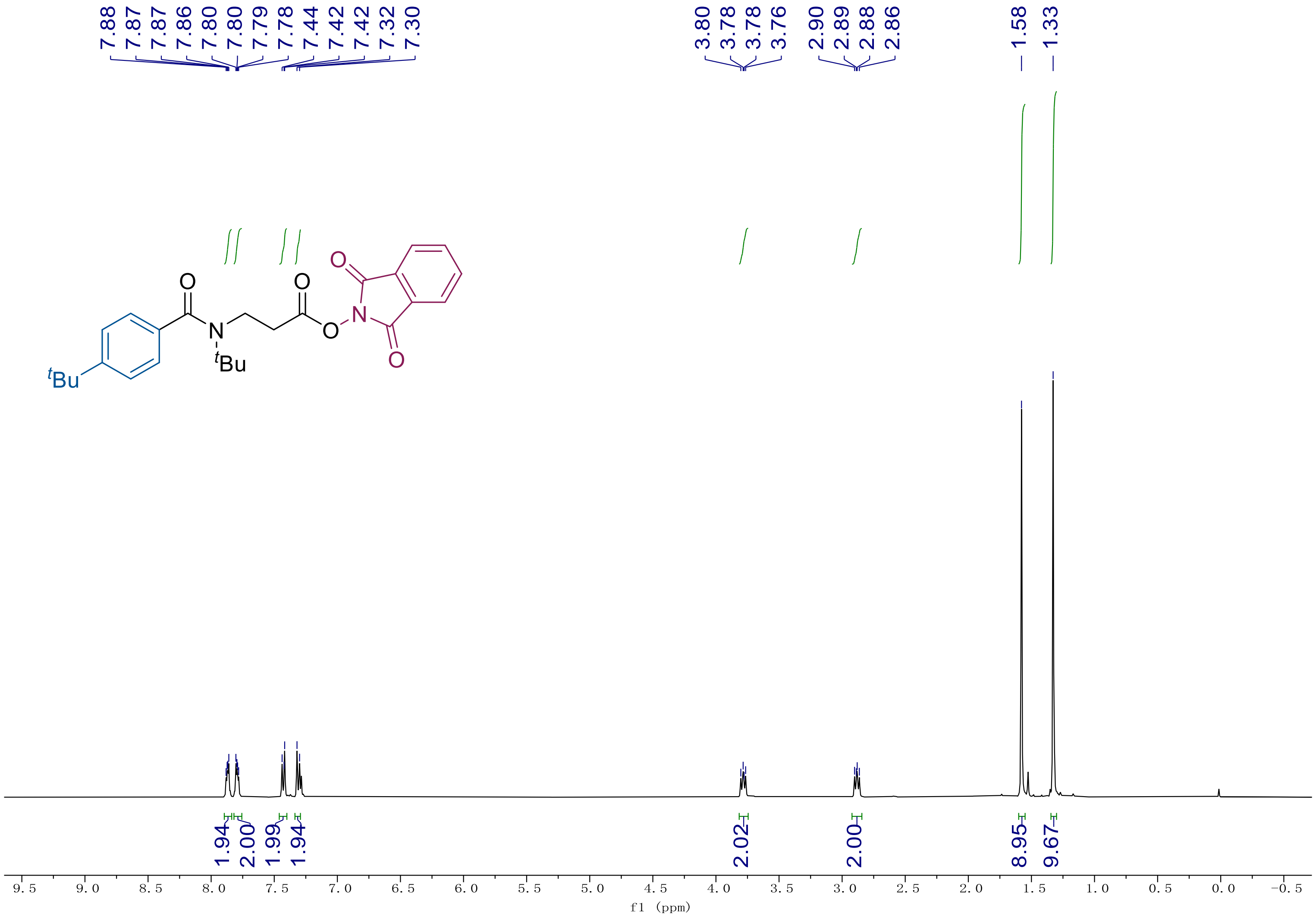

^{13}C NMR (101 MHz, $CDCl_3$) of **1ap**

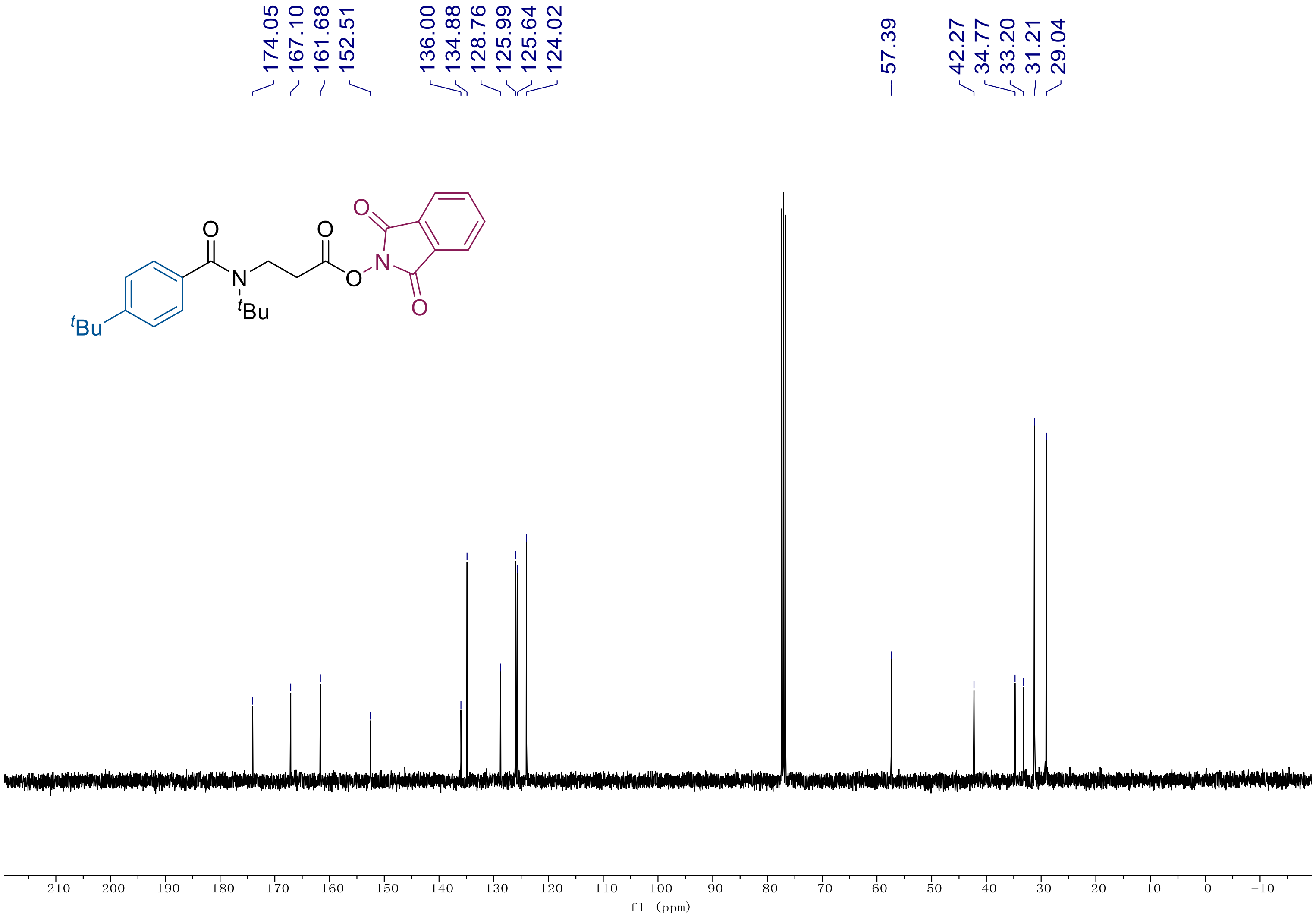

^{1}H NMR (500 MHz, $CDCl_3$) of **1aq**

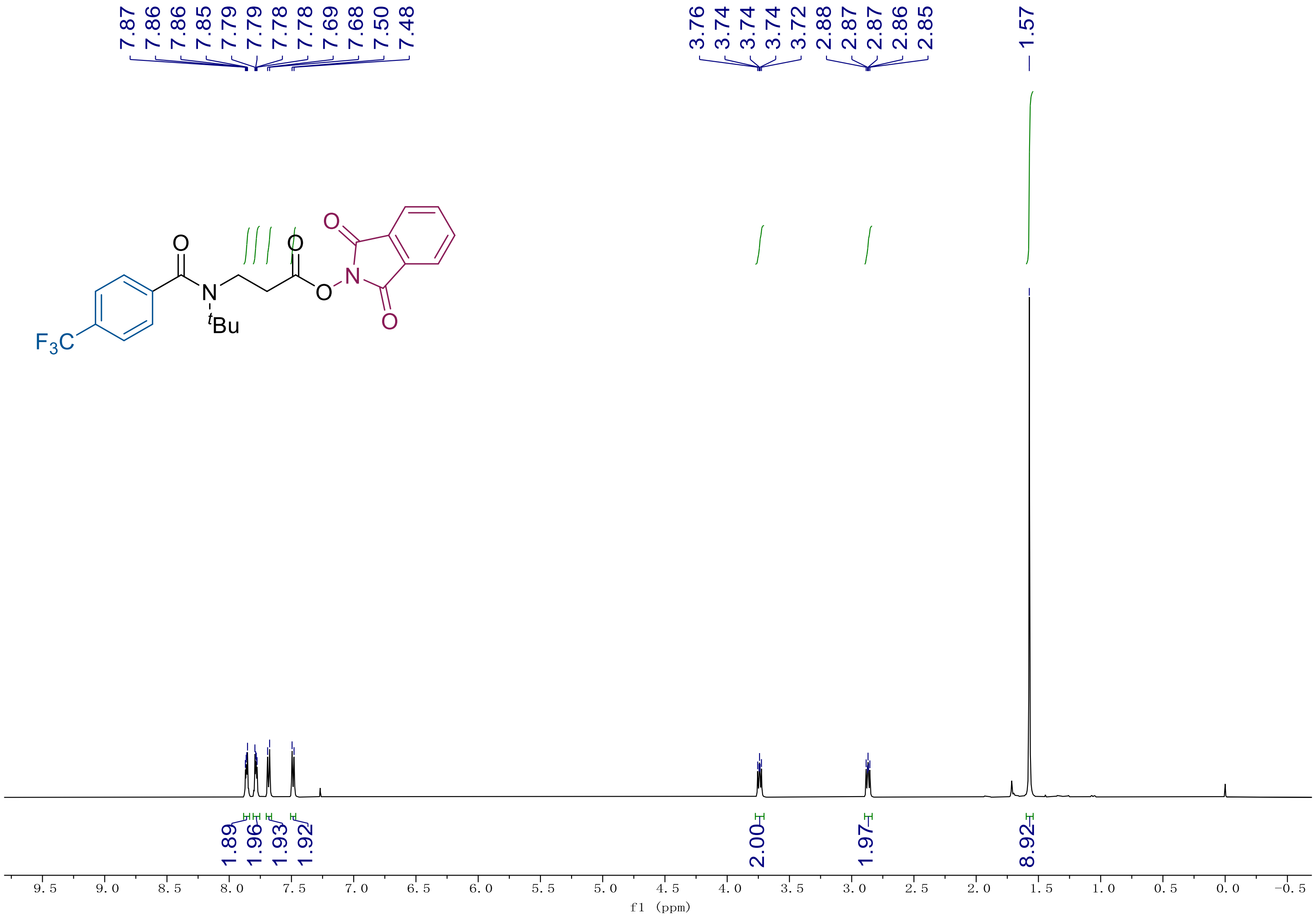

^{13}C NMR (126 MHz, $CDCl_3$) of **1aq**

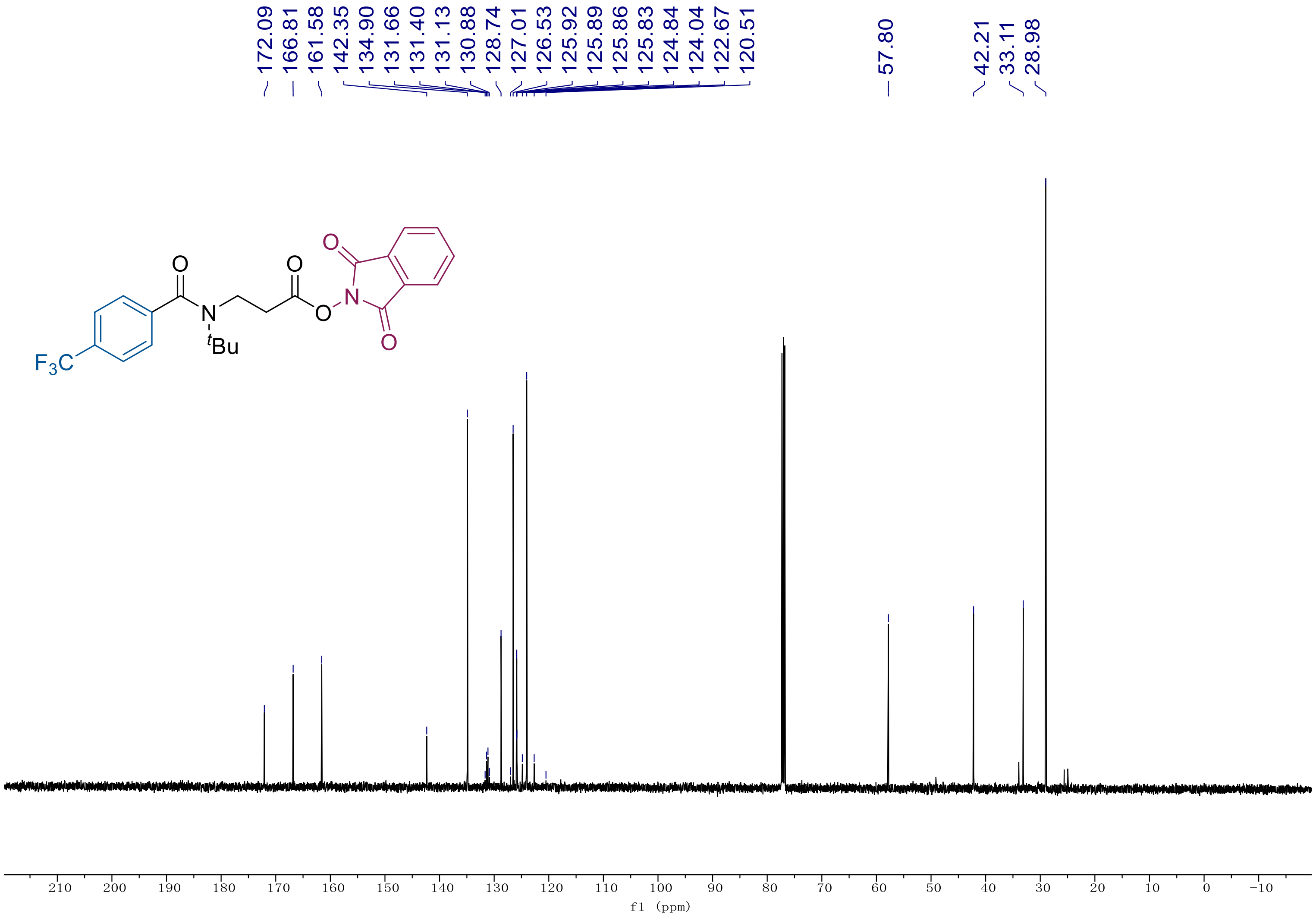

^{19}F NMR (471 MHz, $CDCl_3$) of **1aq**

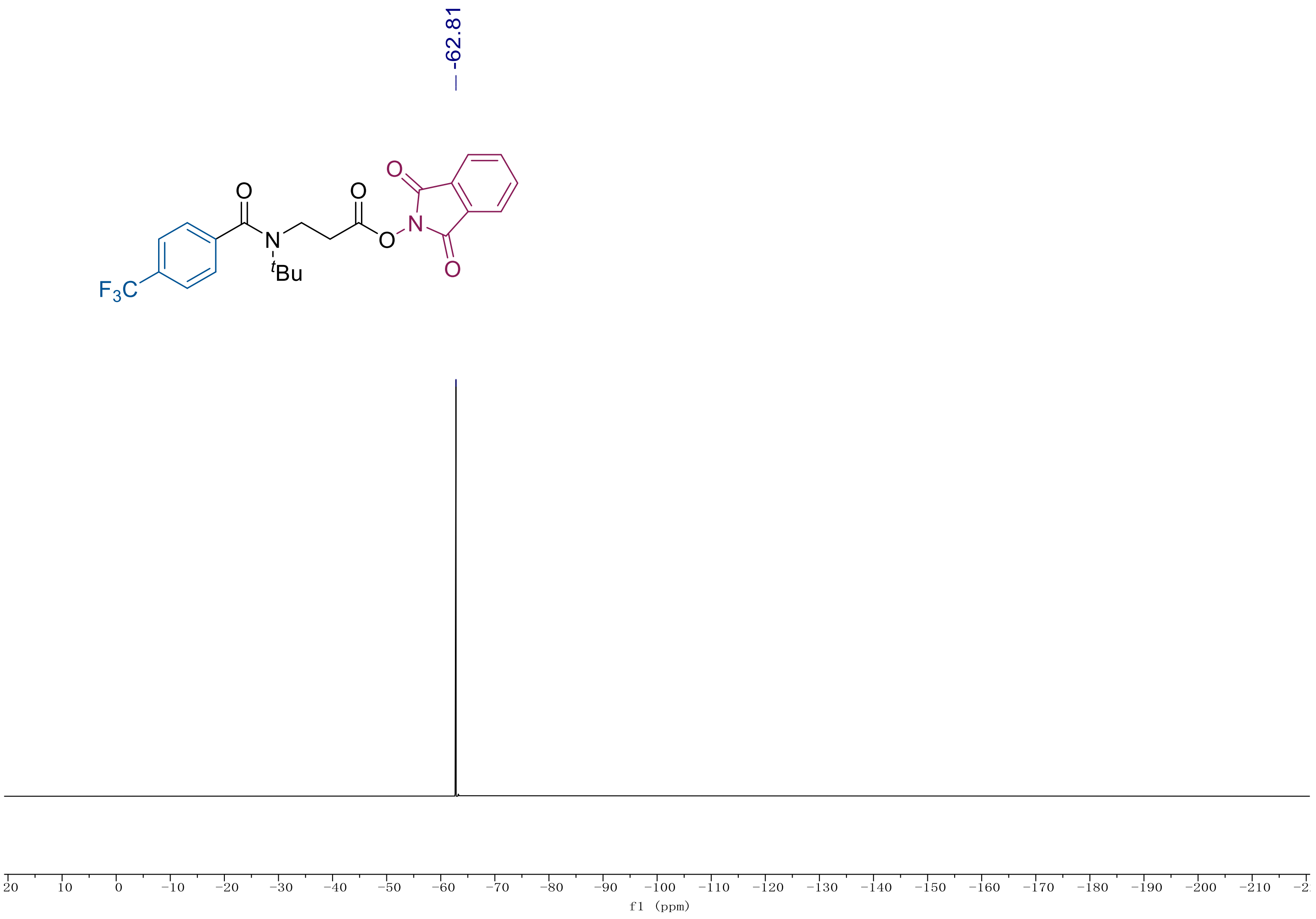

^{1}H NMR (400 MHz, $CDCl_3$) of **1ar**

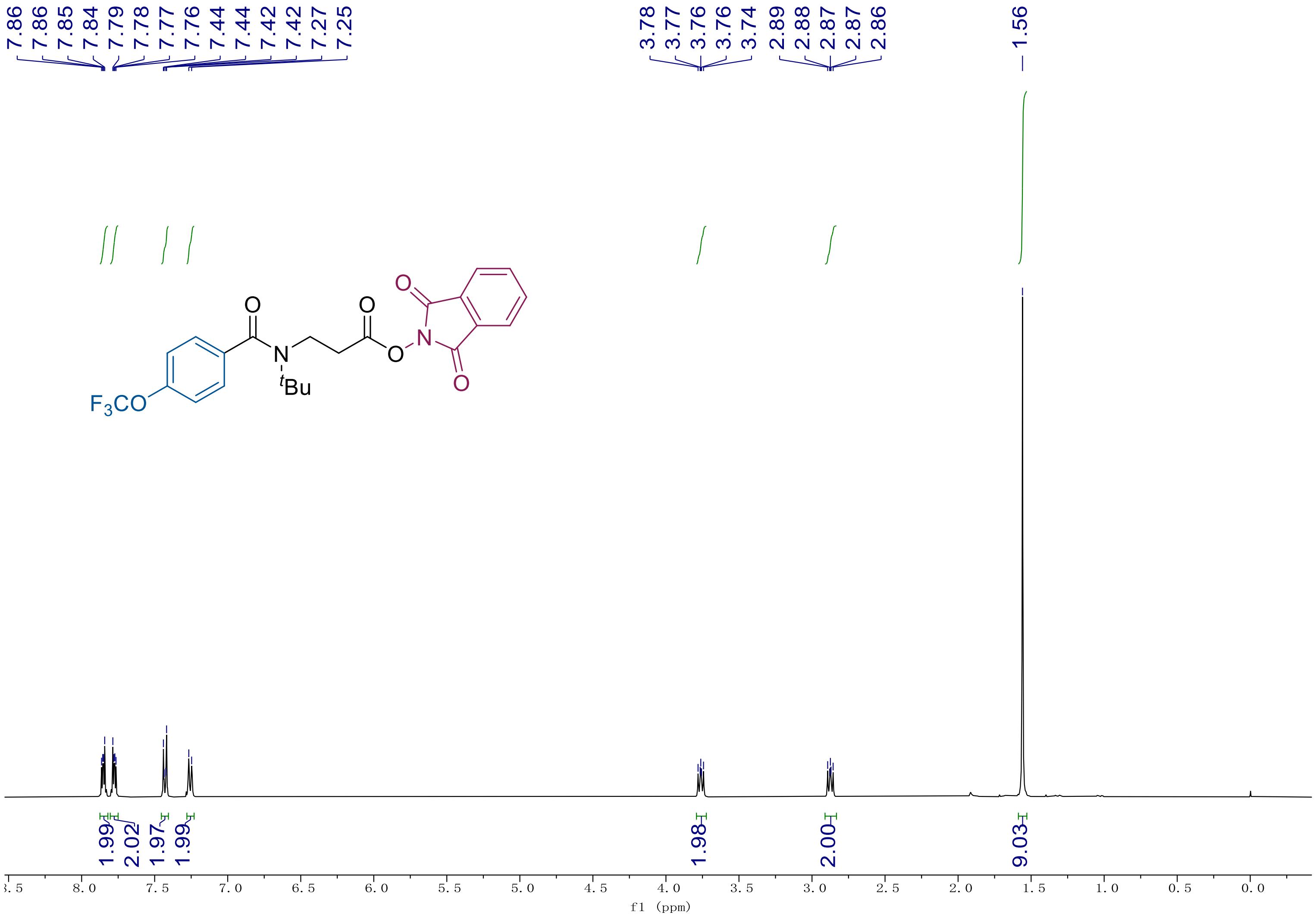

^{13}C NMR (101 MHz, $CDCl_3$) of **1ar**

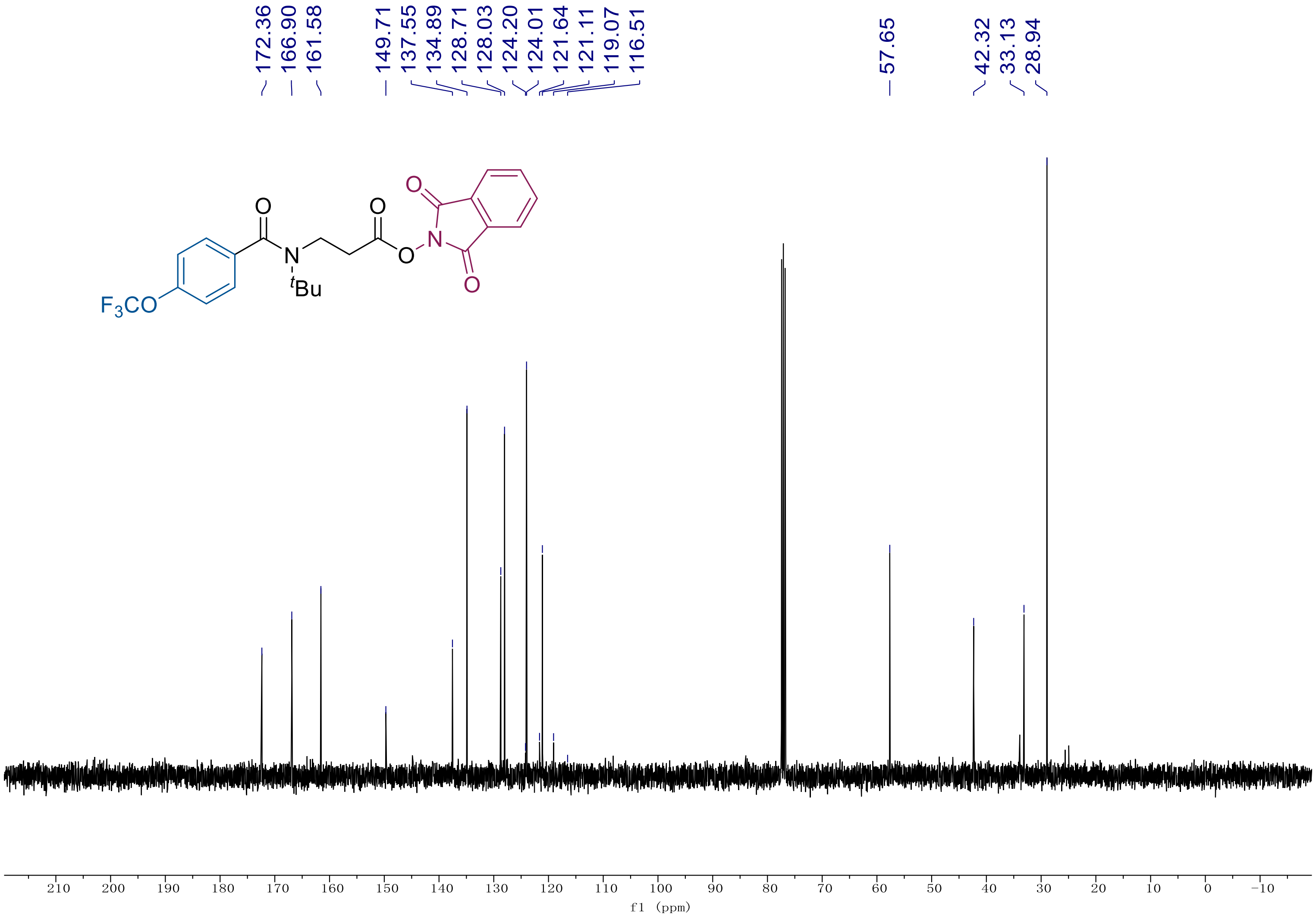

^{19}F NMR (376 MHz, $CDCl_3$) of **1ar**

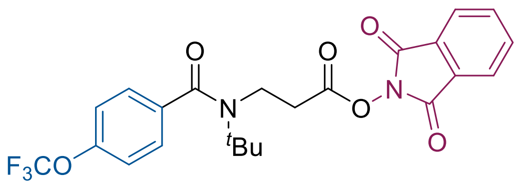

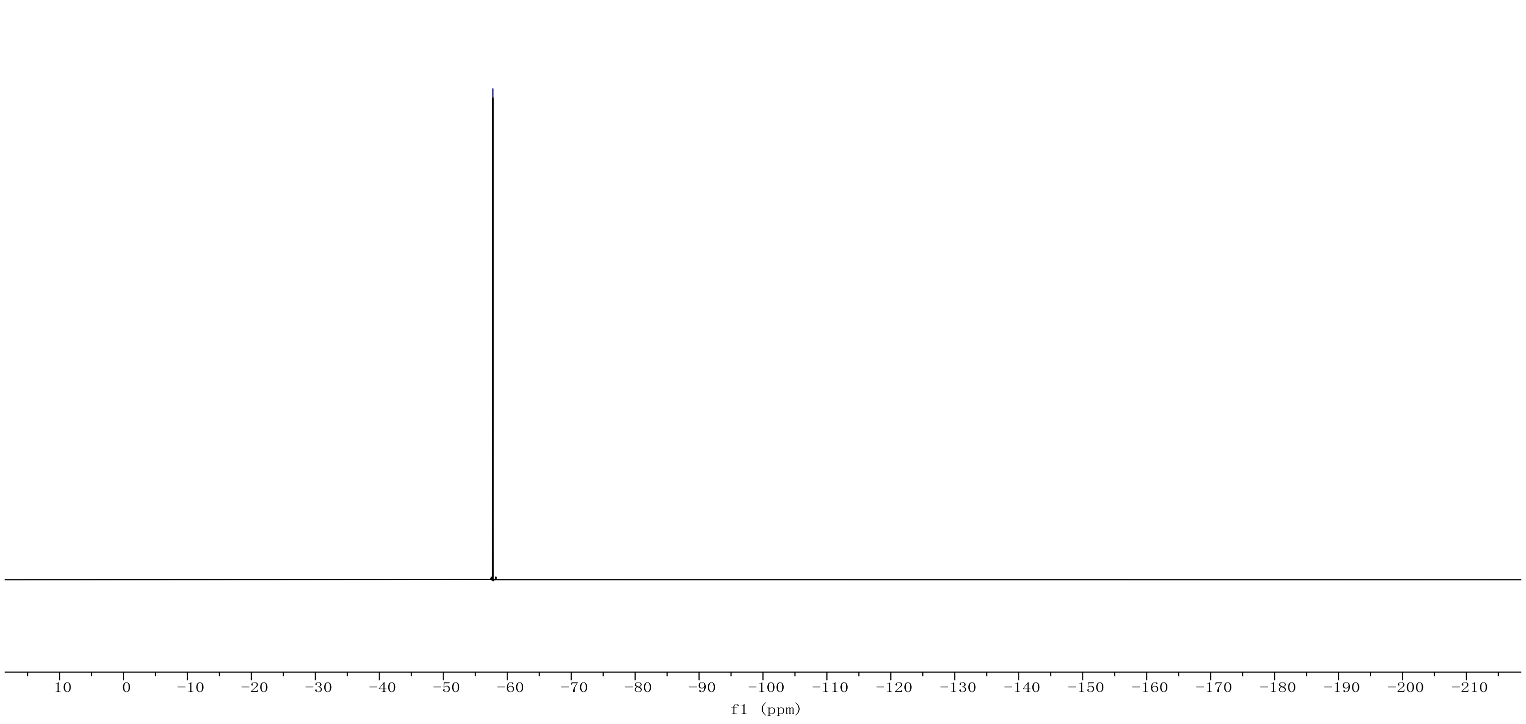

^1H NMR (400 MHz, $CDCl_3$) of **3a**

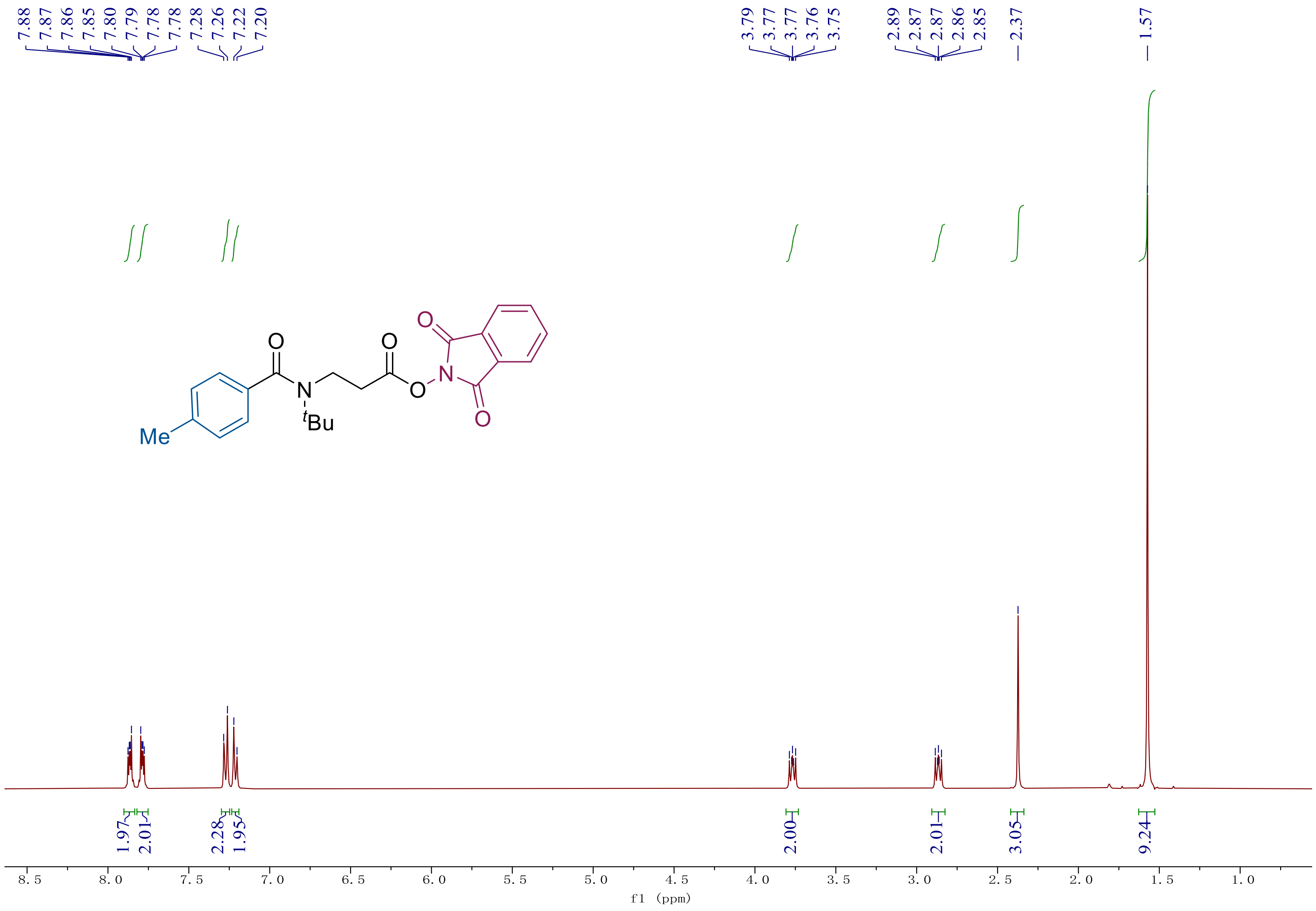

^{13}C NMR (101 MHz, $CDCl_3$) of **3a**

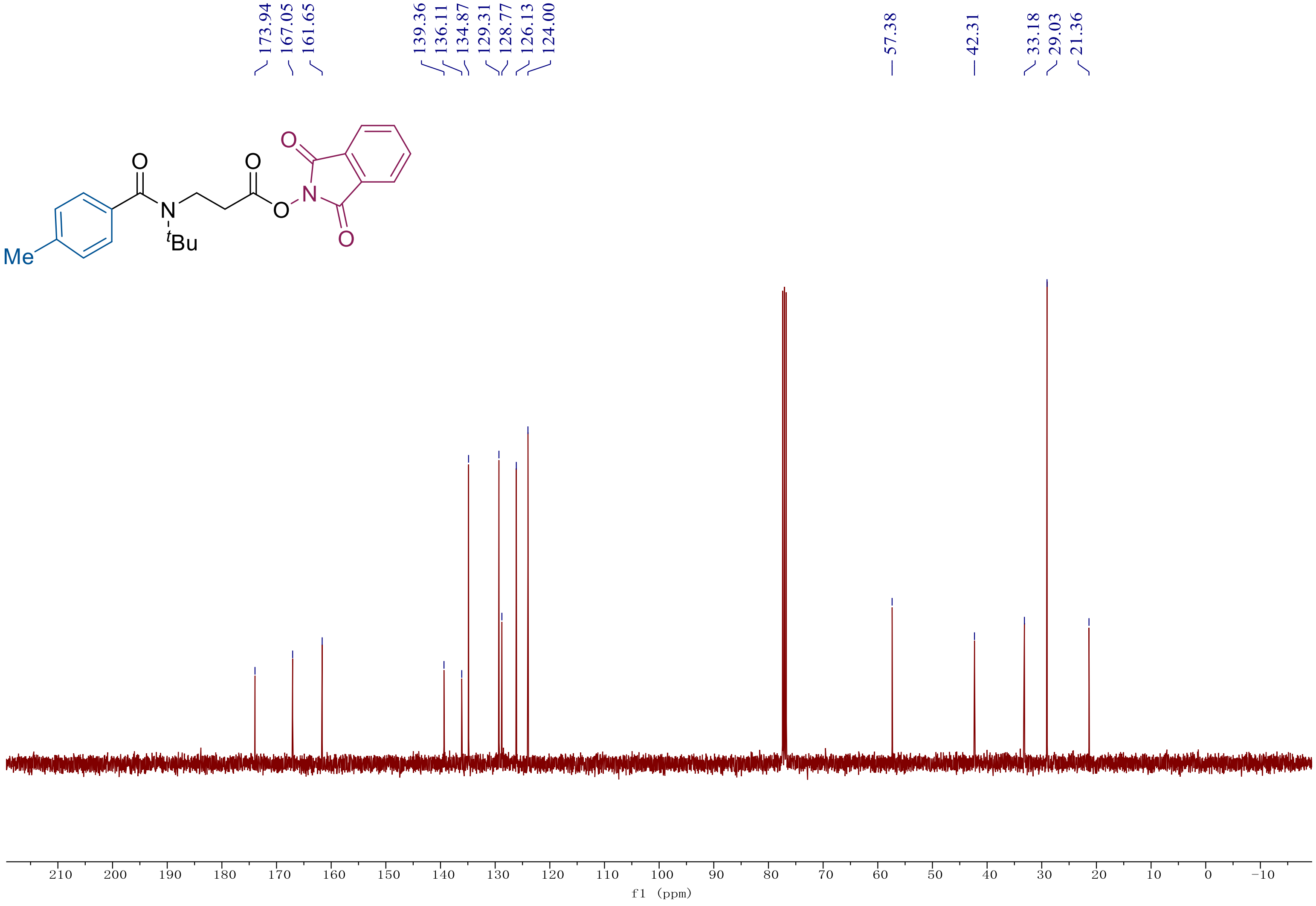

^{1}H NMR (400 MHz, $CDCl_3$) of **3b**

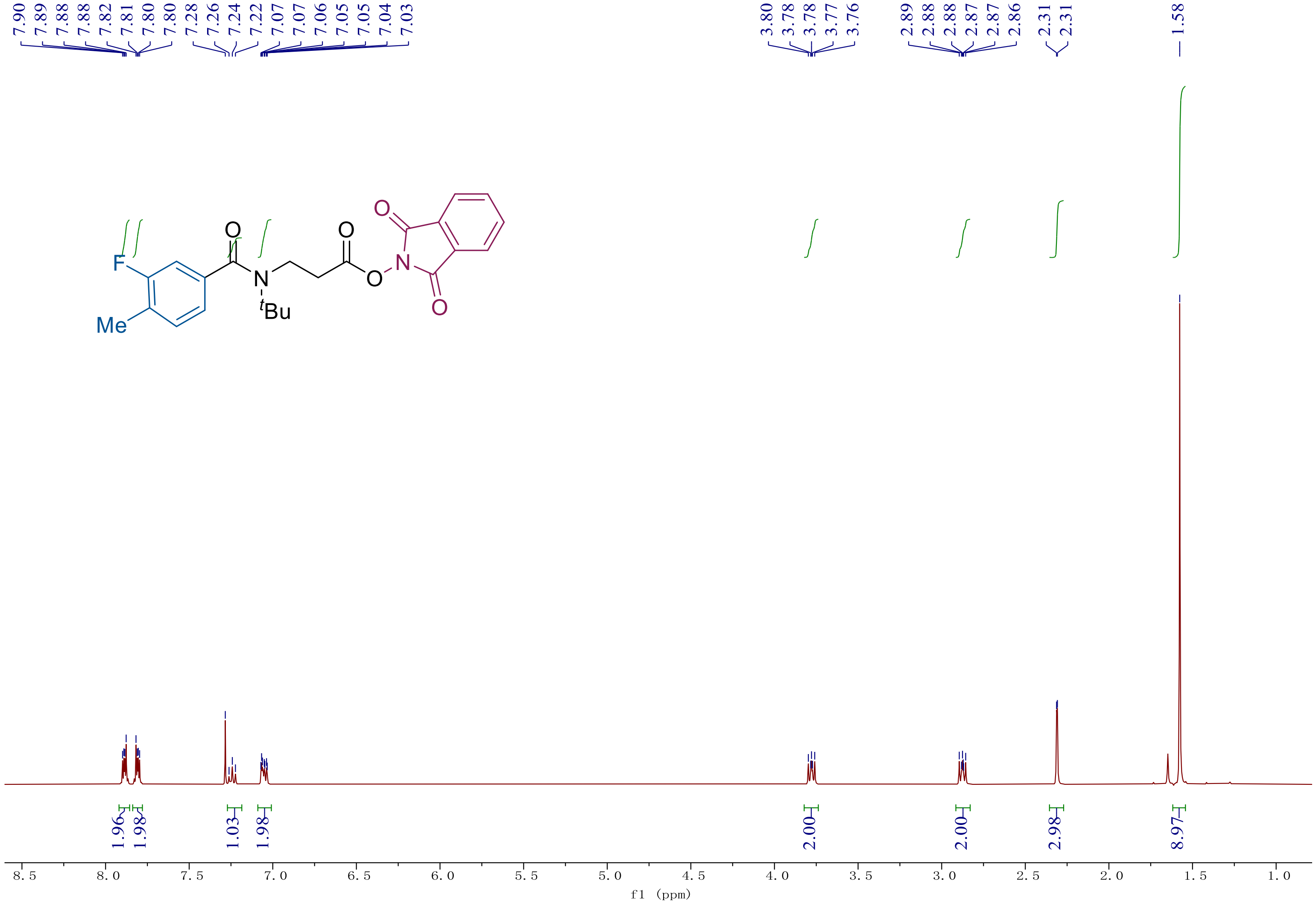

^{13}C NMR (101 MHz, $CDCl_3$) of **3b**

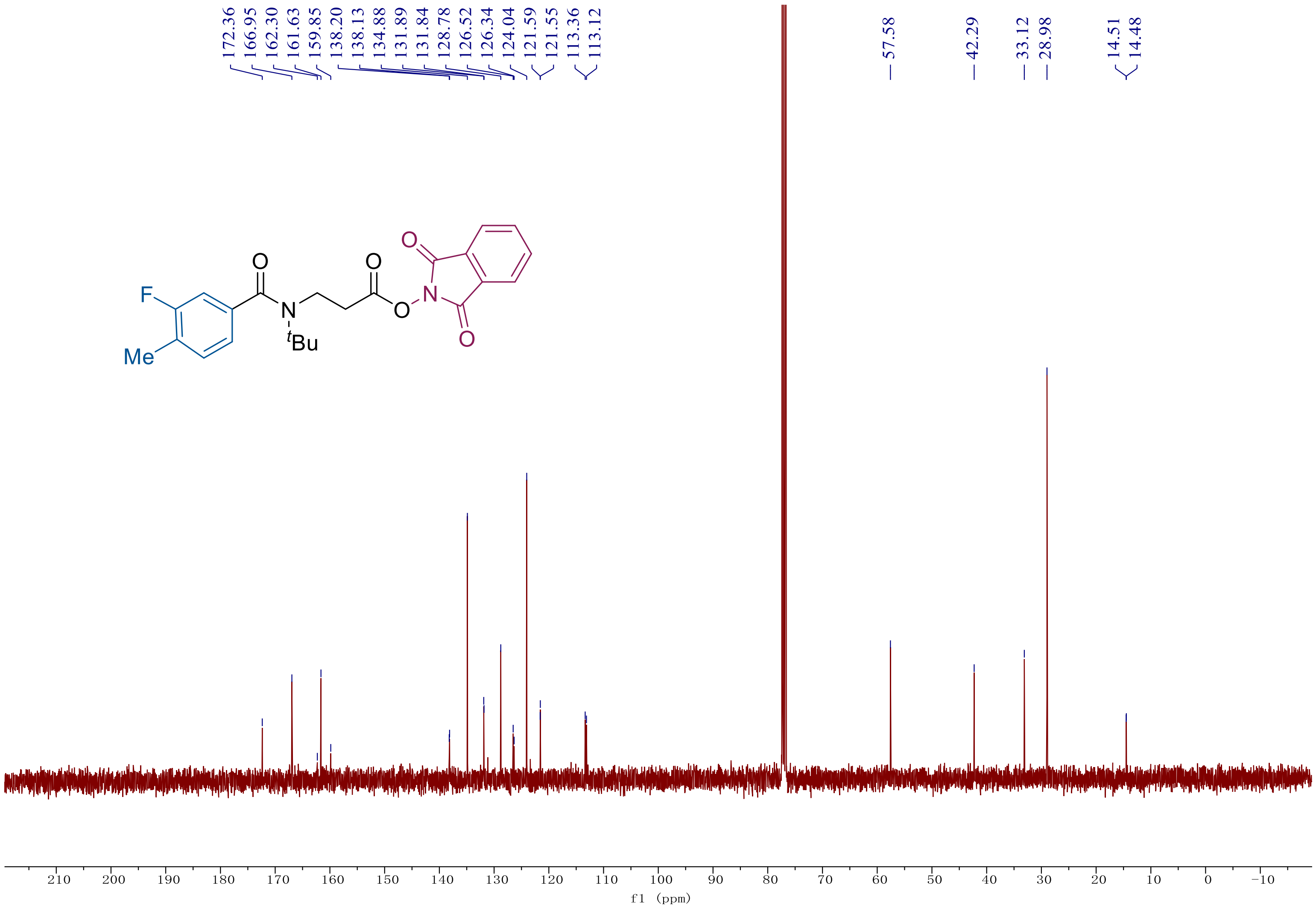

^{19}F NMR (376 MHz, $CDCl_3$) of **3b**

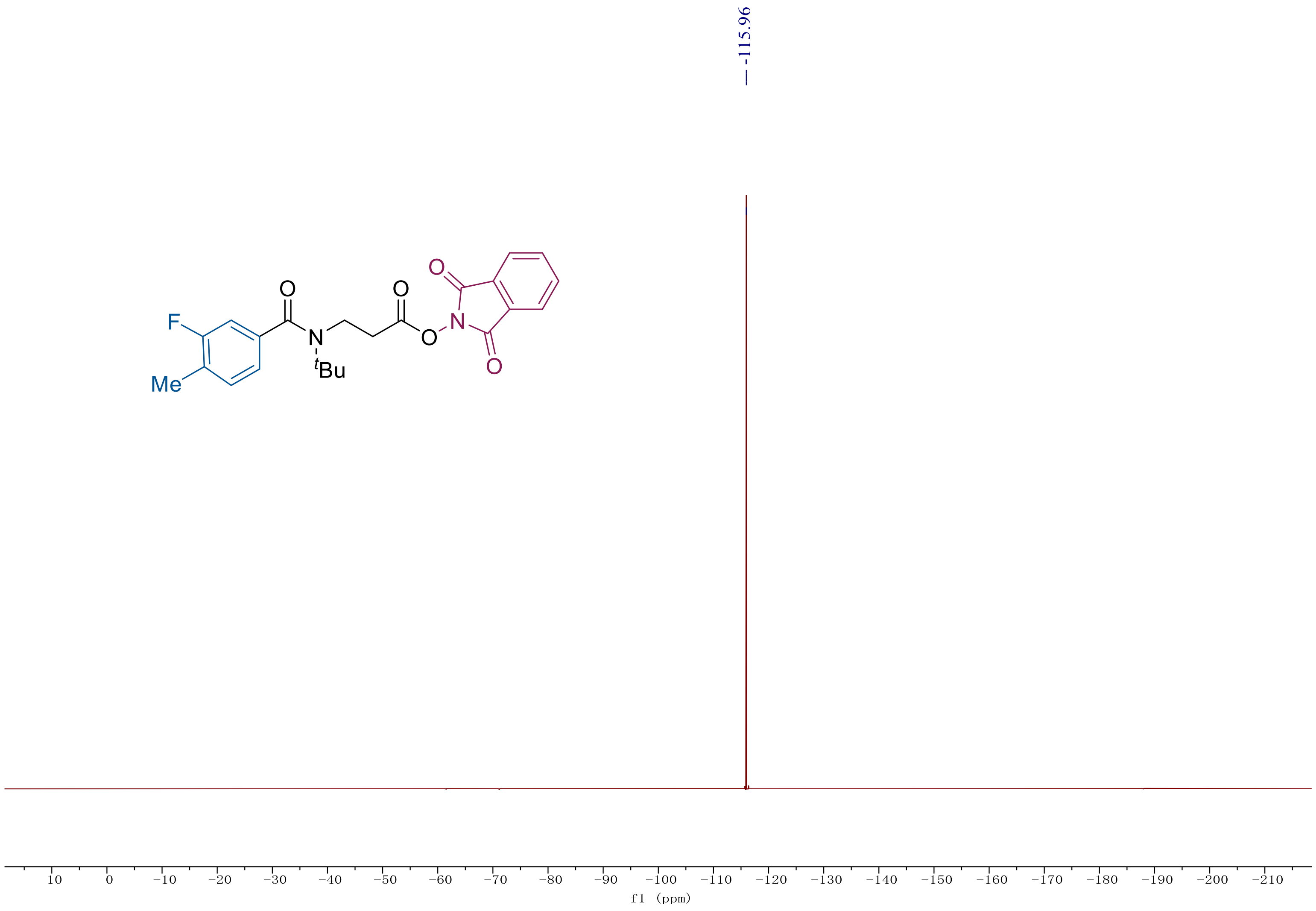

^1H NMR (400 MHz, $CDCl_3$) of **3c**

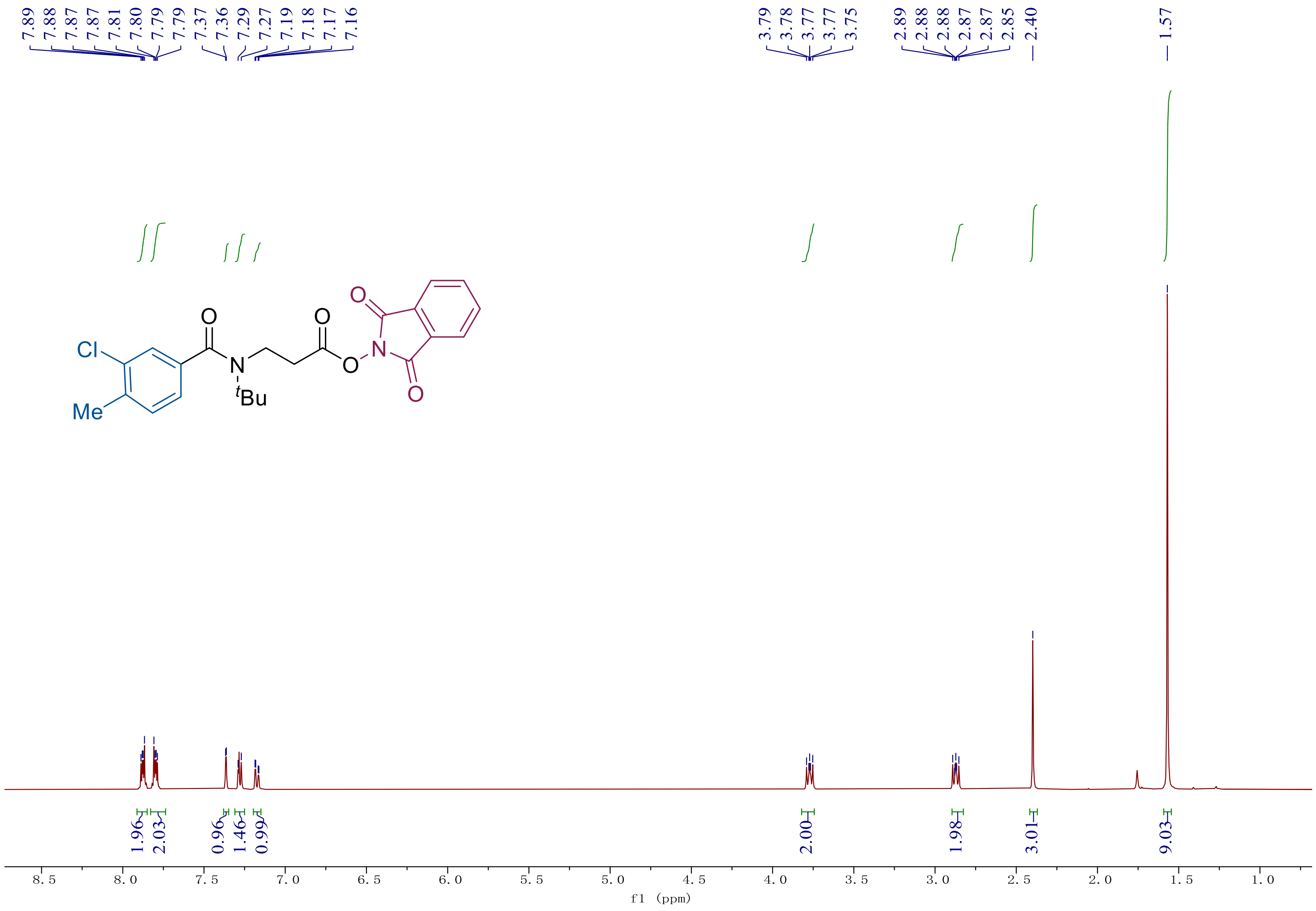

^{13}C NMR (101 MHz, $CDCl_3$) of **3c**

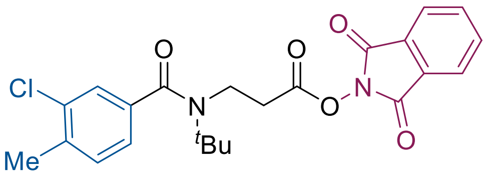

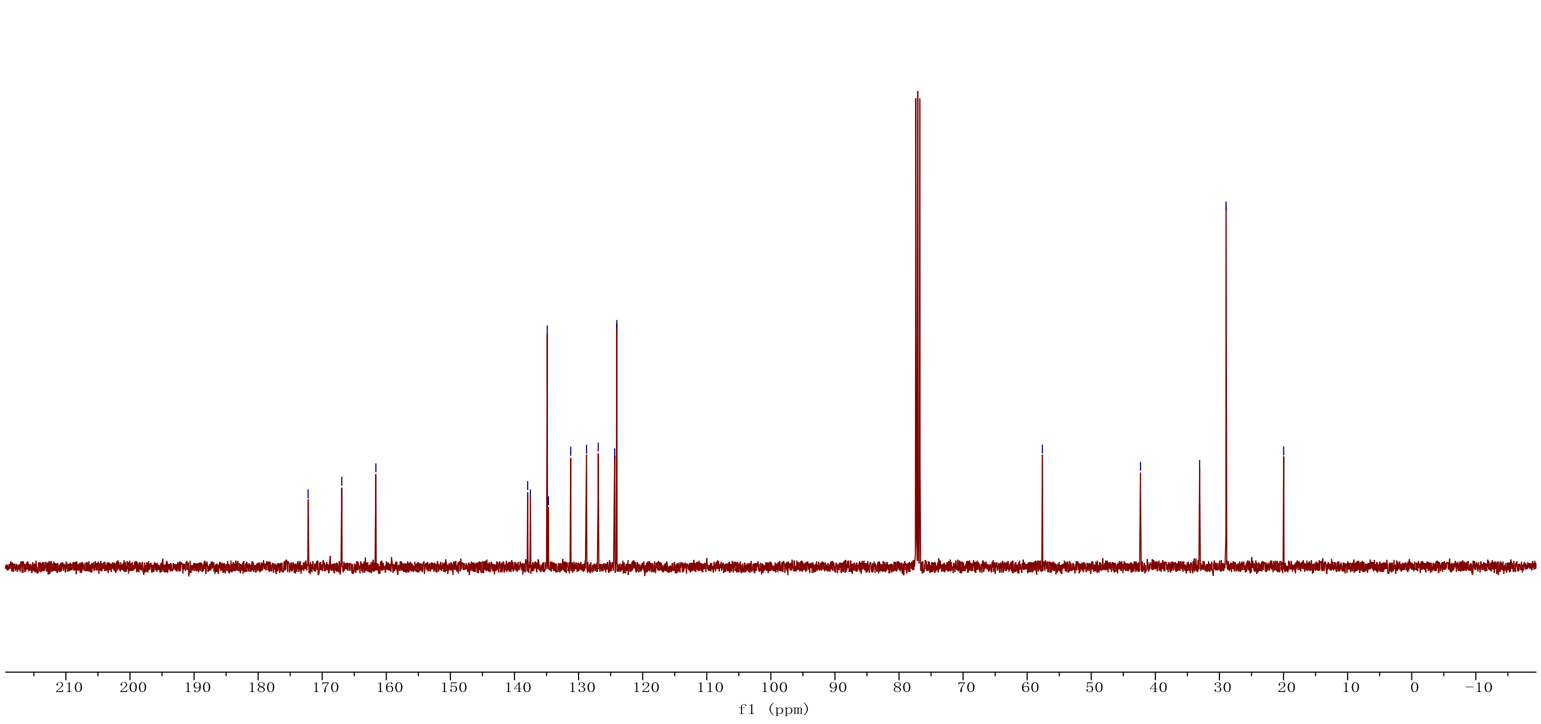

^1H NMR (400 MHz, $CDCl_3$) of **3d**

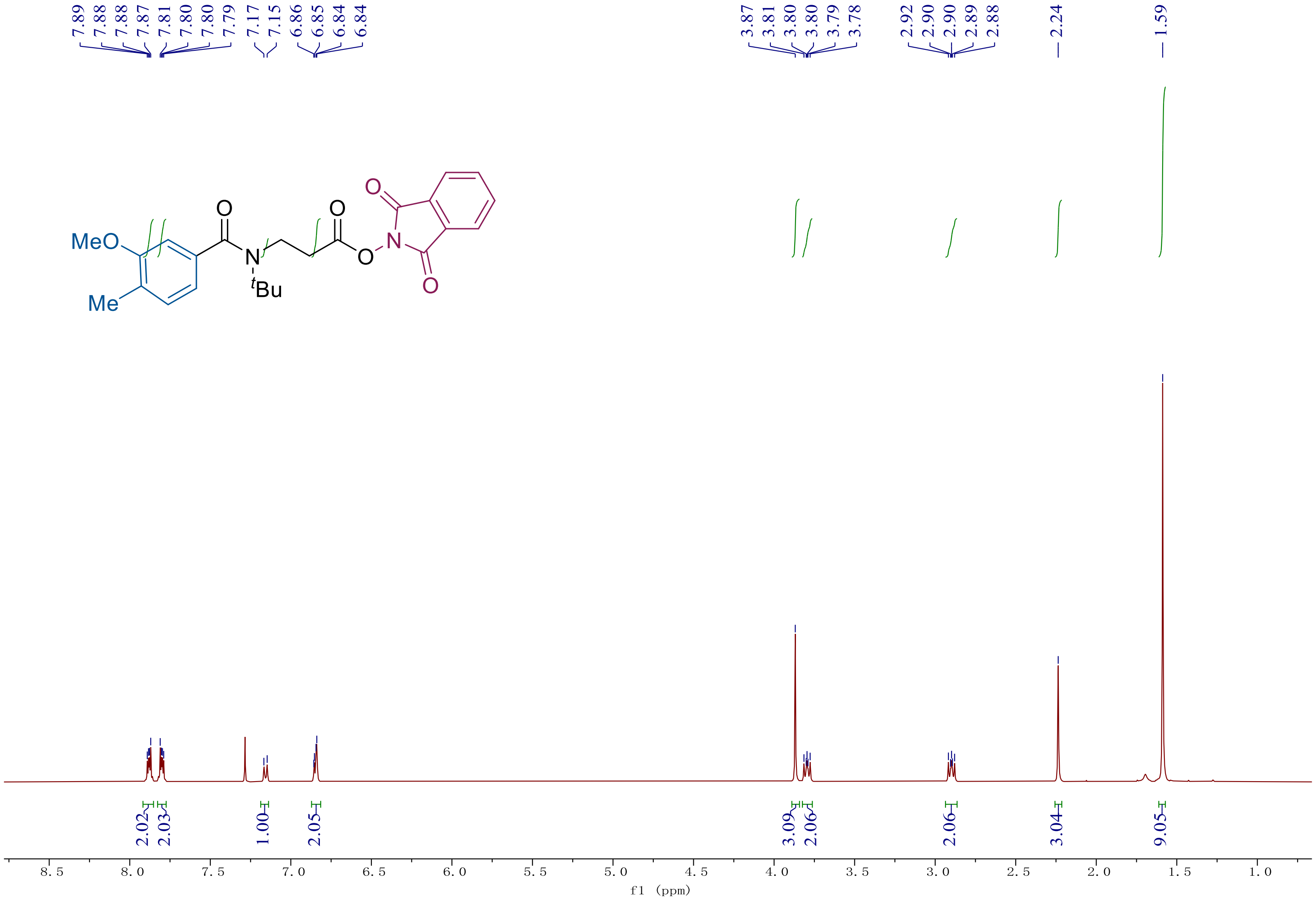

^{13}C NMR (101 MHz, CDCl$_3$) of **3d**

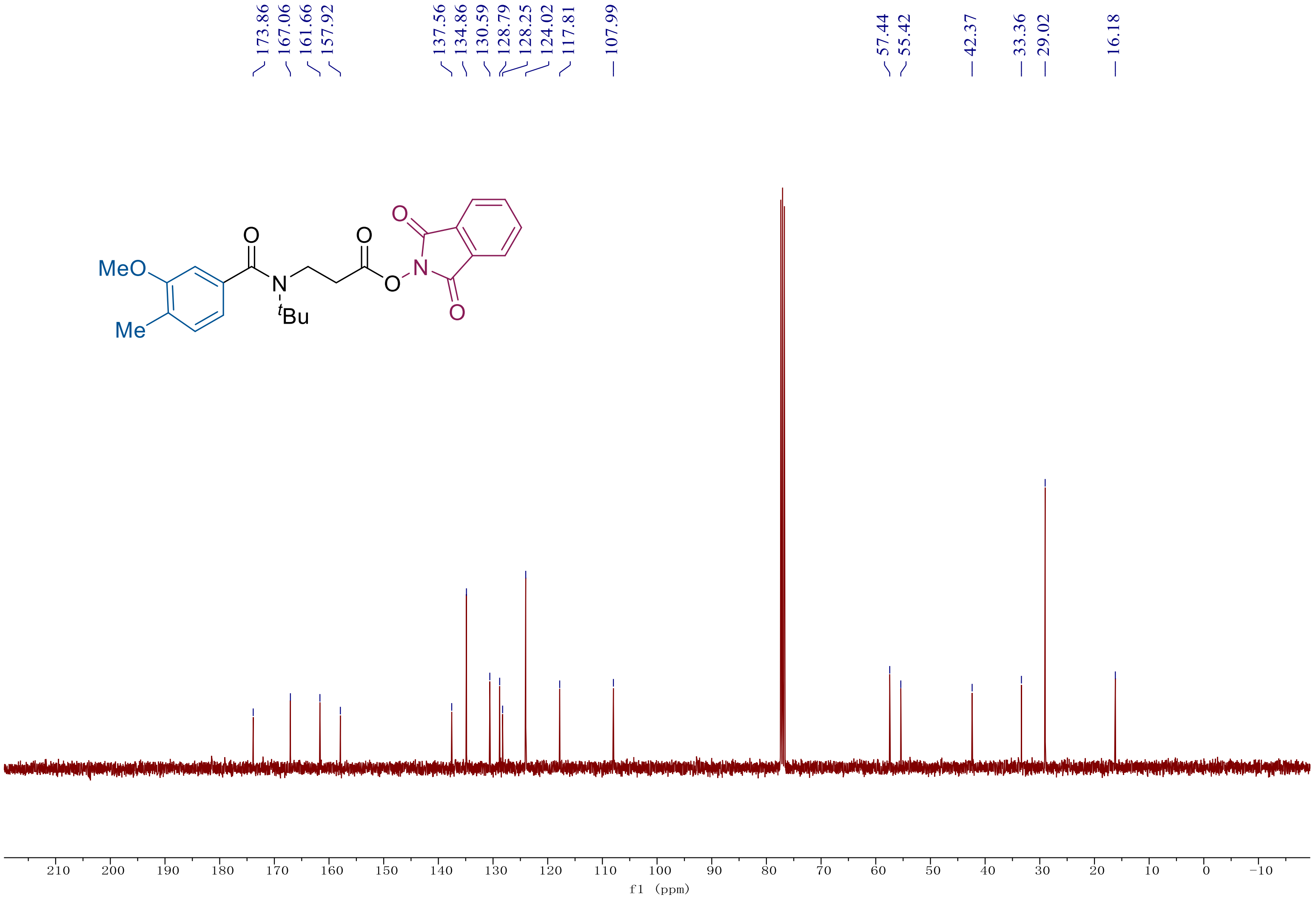

^{1}H NMR (400 MHz, $CDCl_3$) of **3e**

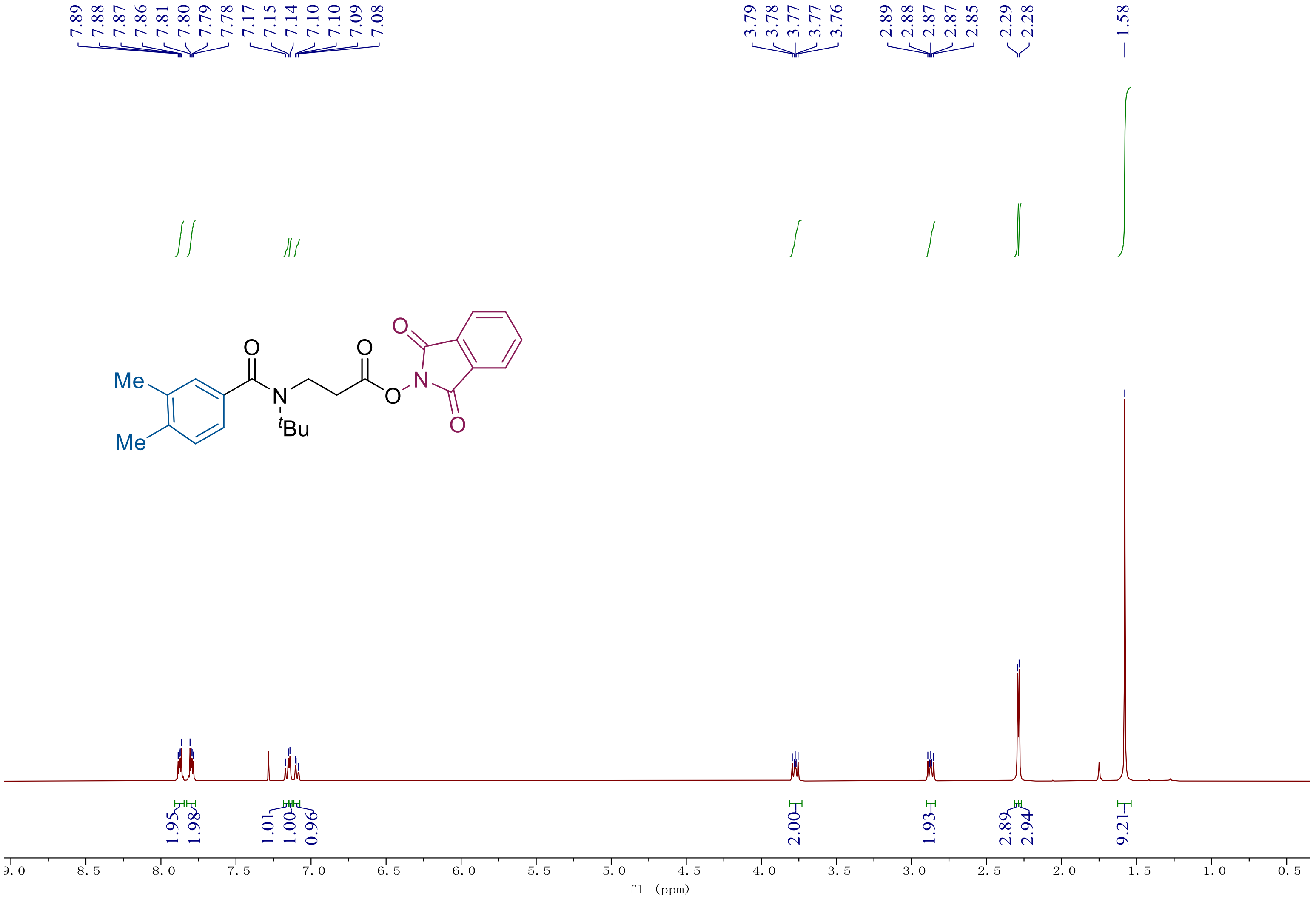

^{13}C NMR (101 MHz, $CDCl_3$) of **3e**

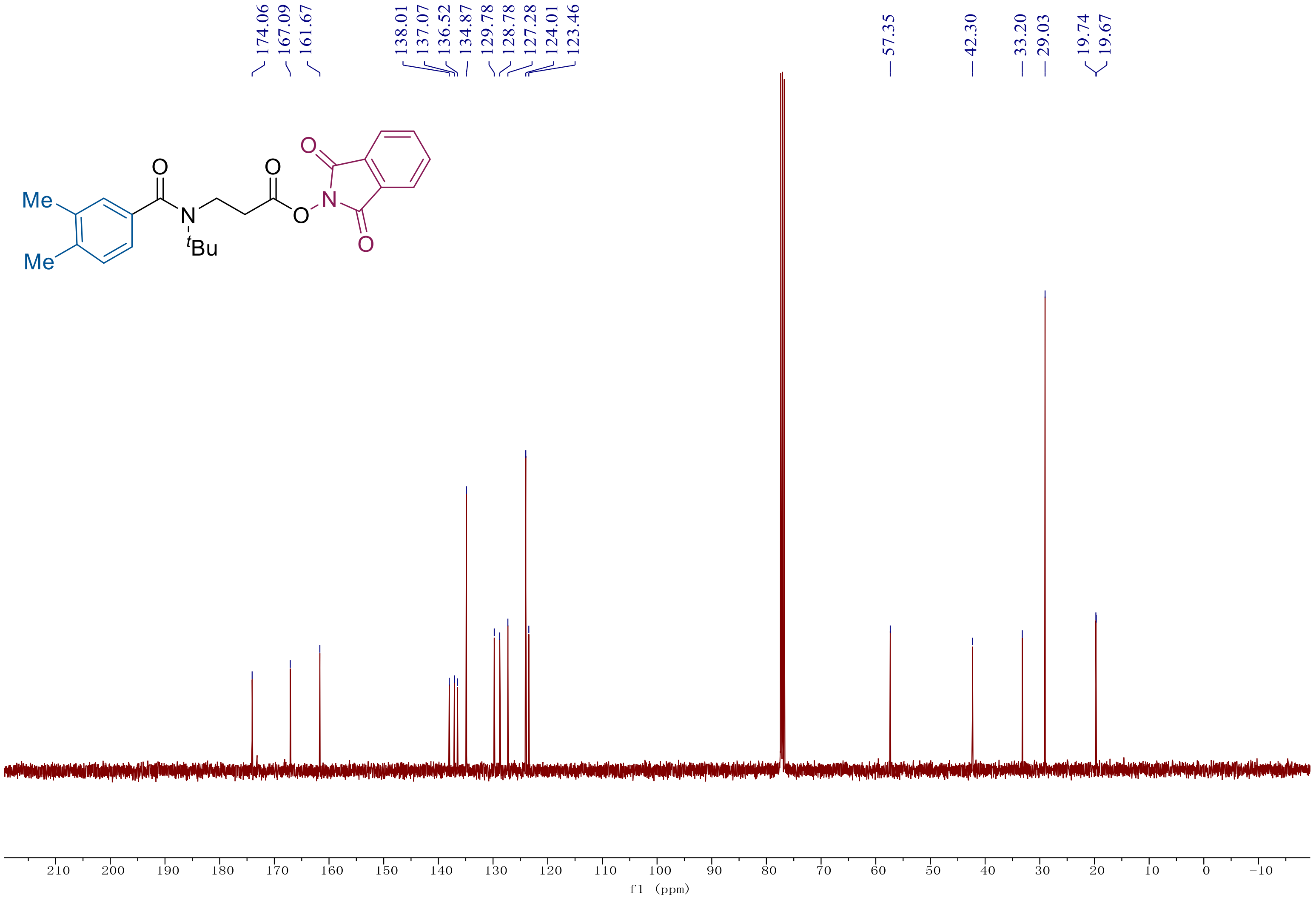

^{1}H NMR (400 MHz, $CDCl_3$) of **3f**

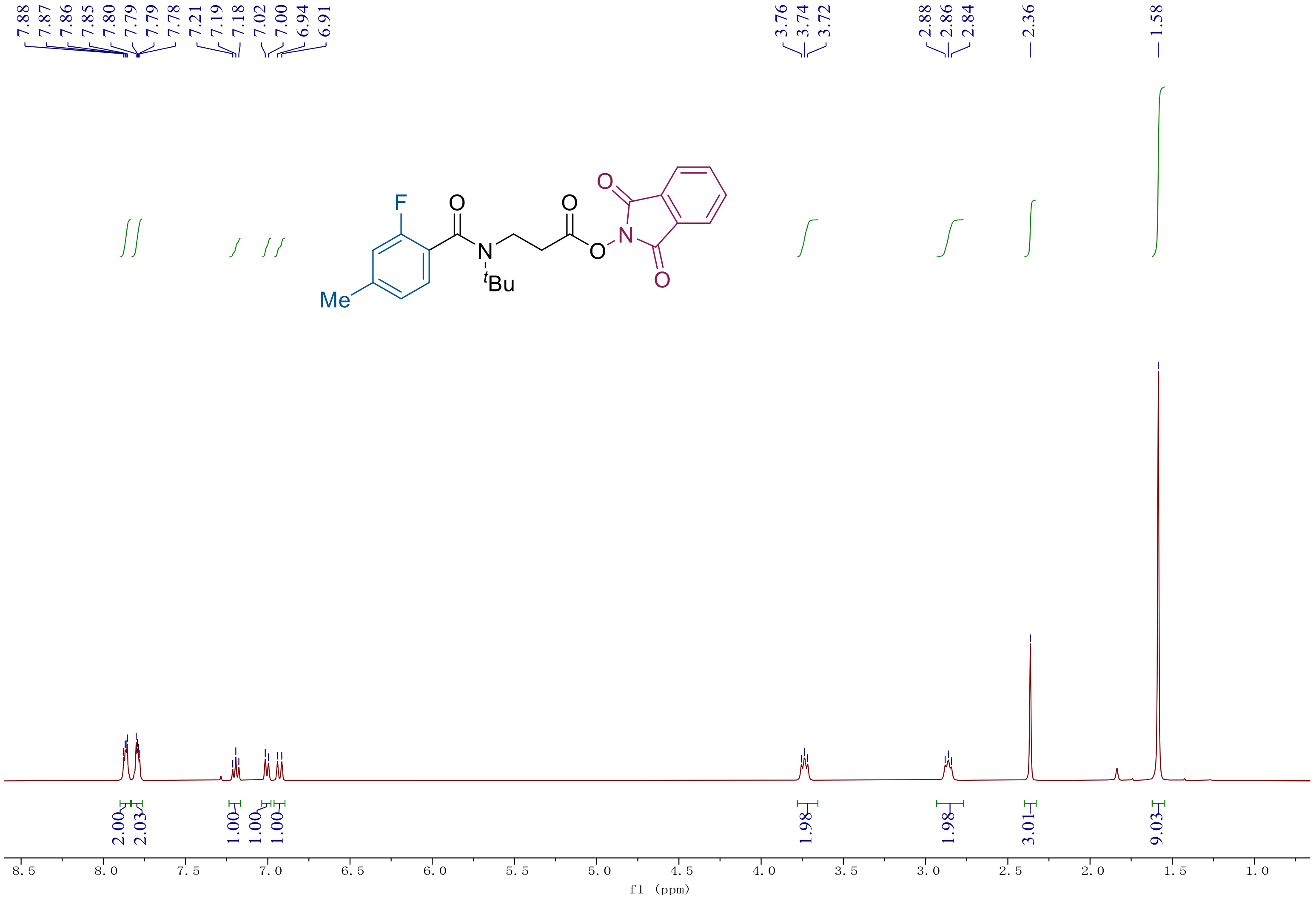

^{13}C NMR (101 MHz, CDCl$_3$) of **3f**

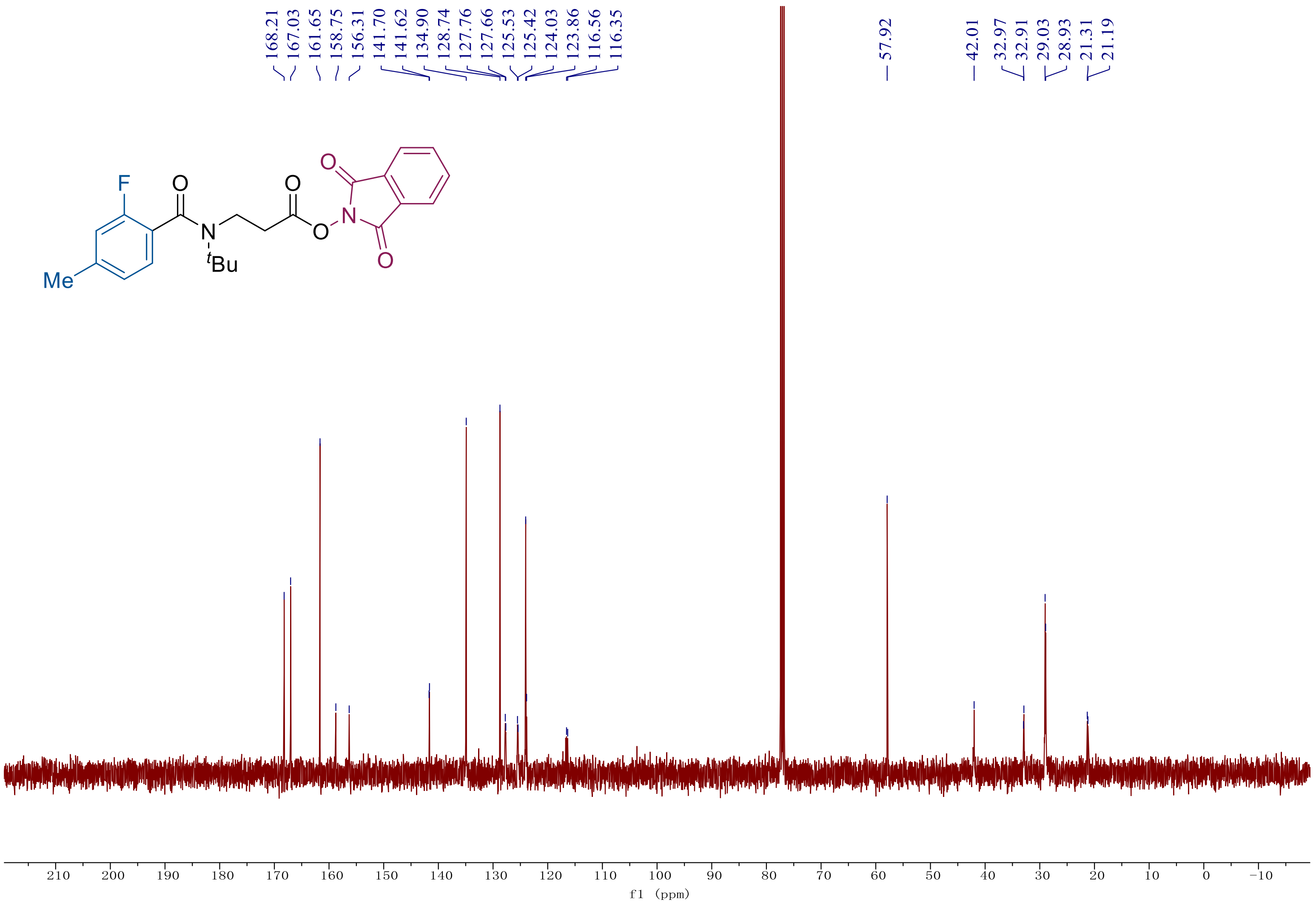

^{19}F NMR (376 MHz, $CDCl_3$) of **3f**

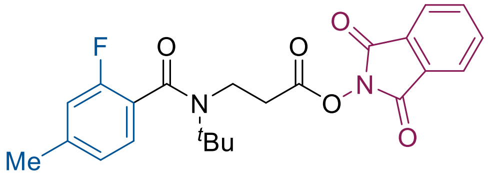

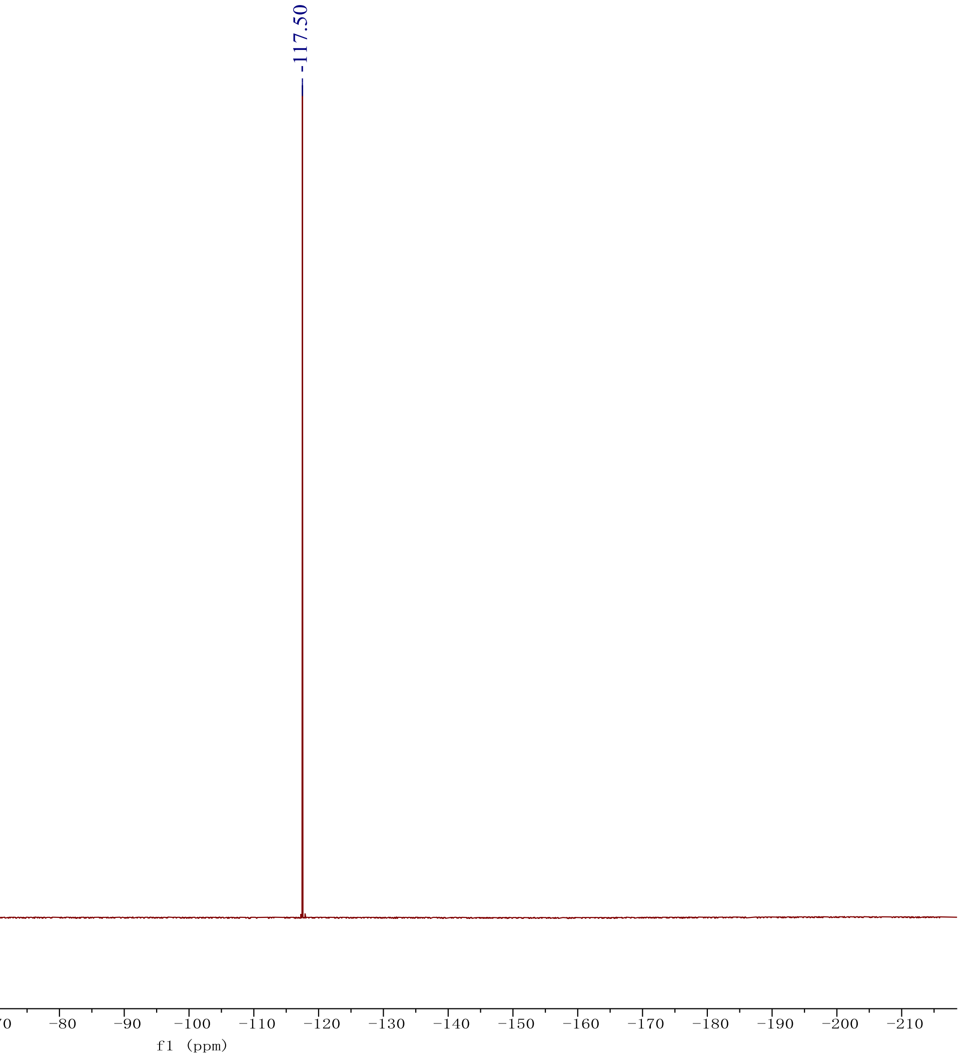

^{1}H NMR (400 MHz, $CDCl_3$) of **3g**

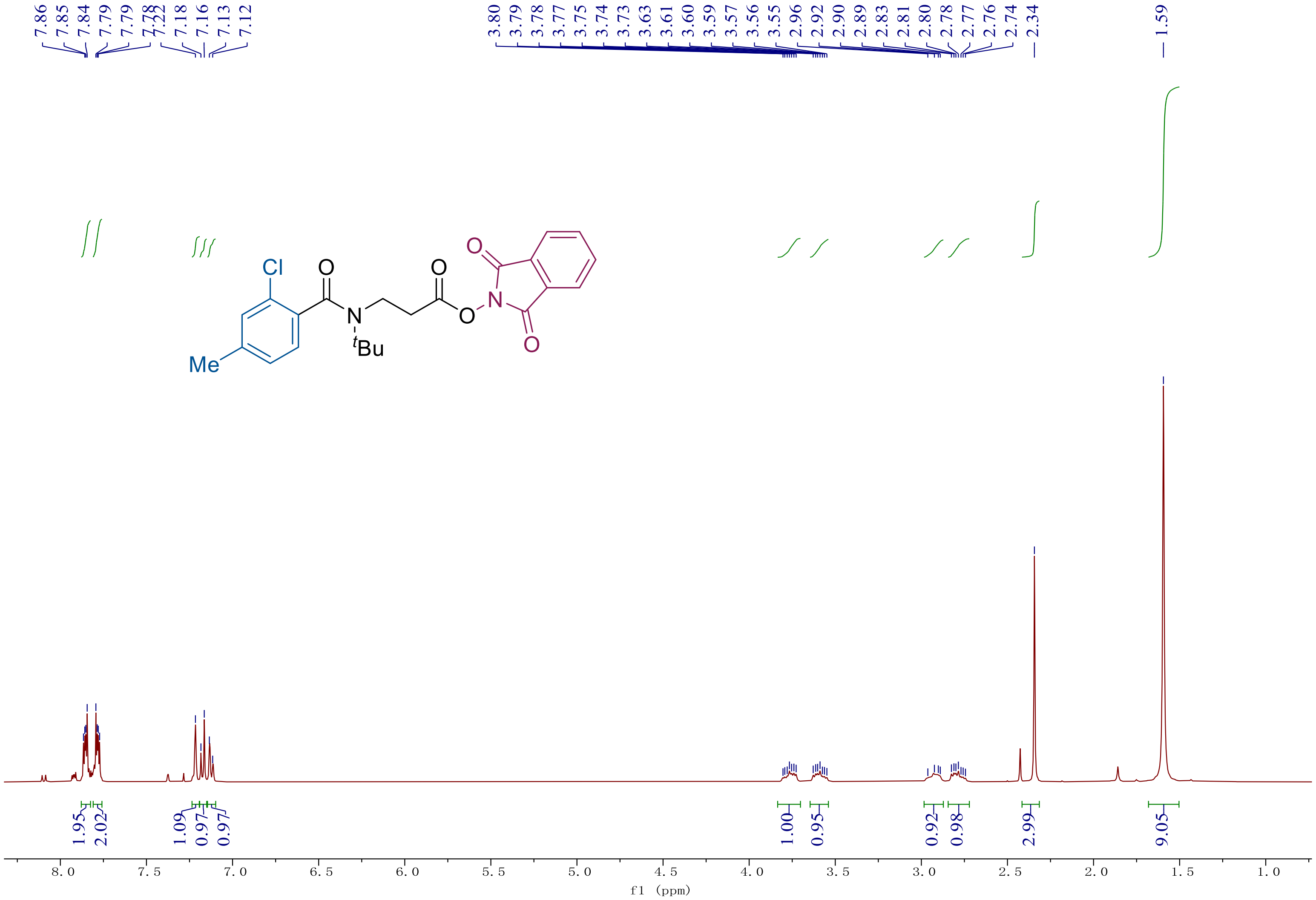

^{13}C NMR (101 MHz, CDCl$_3$) of **3g**

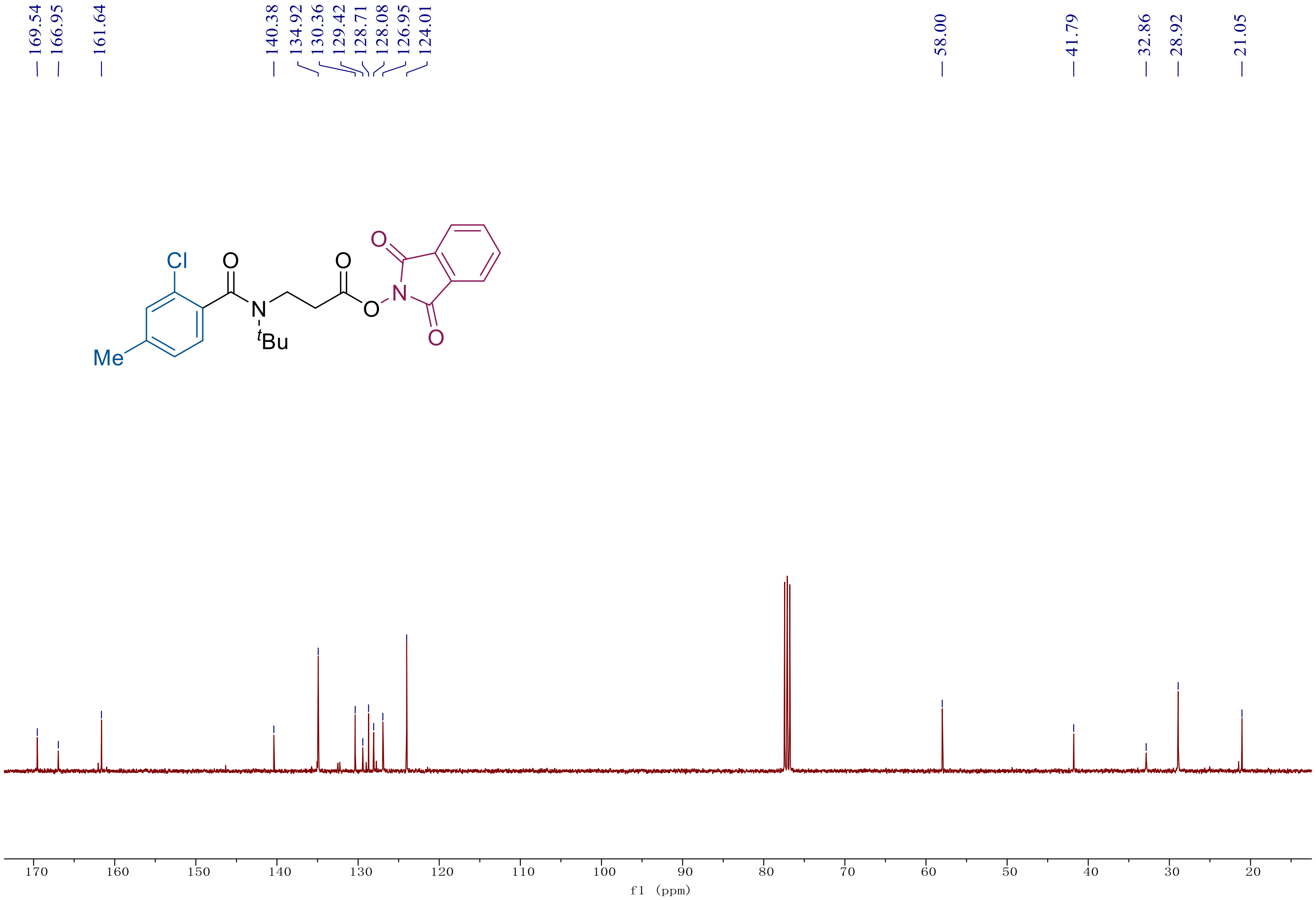

^{1}H NMR (400 MHz, $CDCl_3$) of **3h**

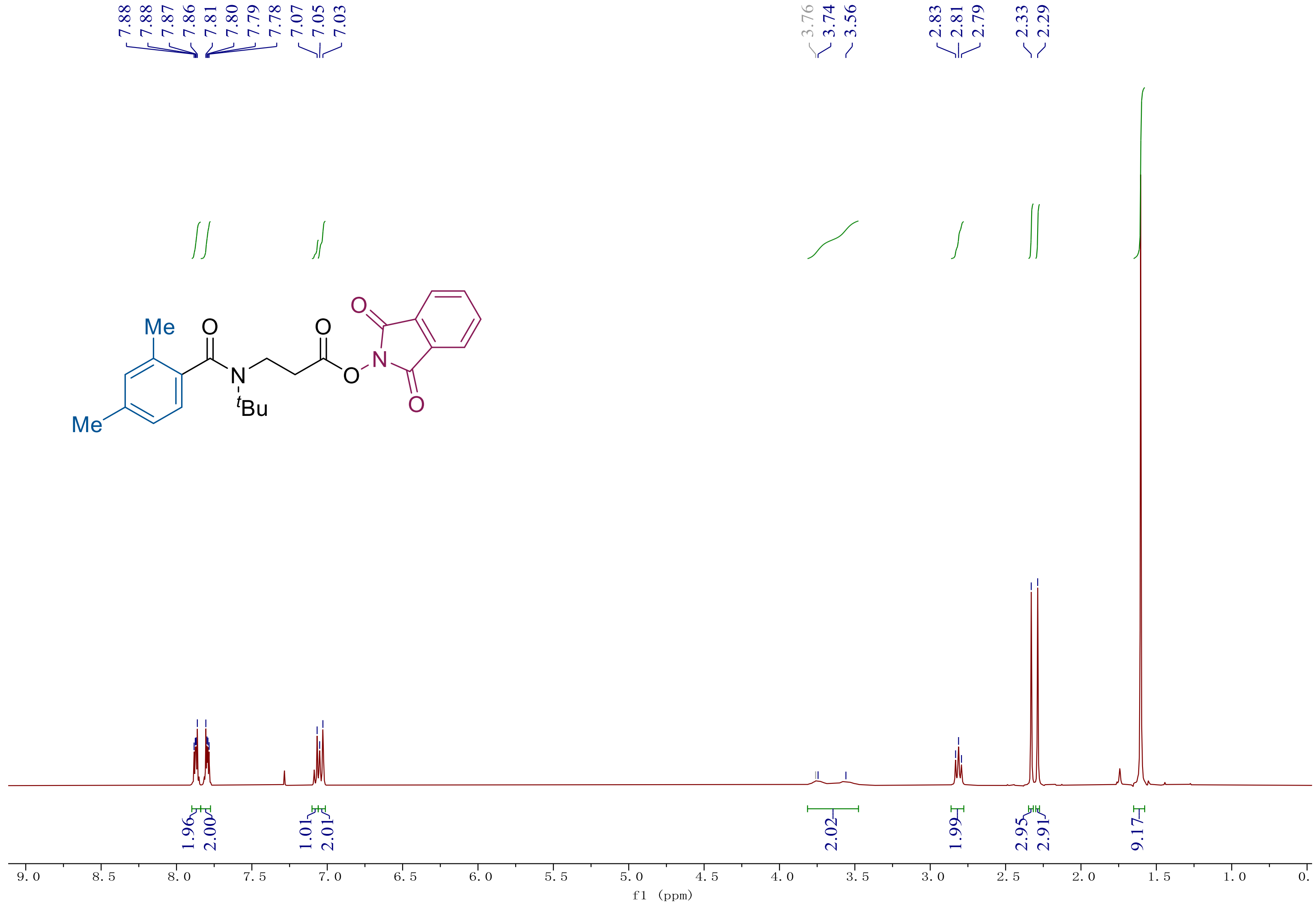

^{13}C NMR (101 MHz, CDCl$_3$) of **3h**

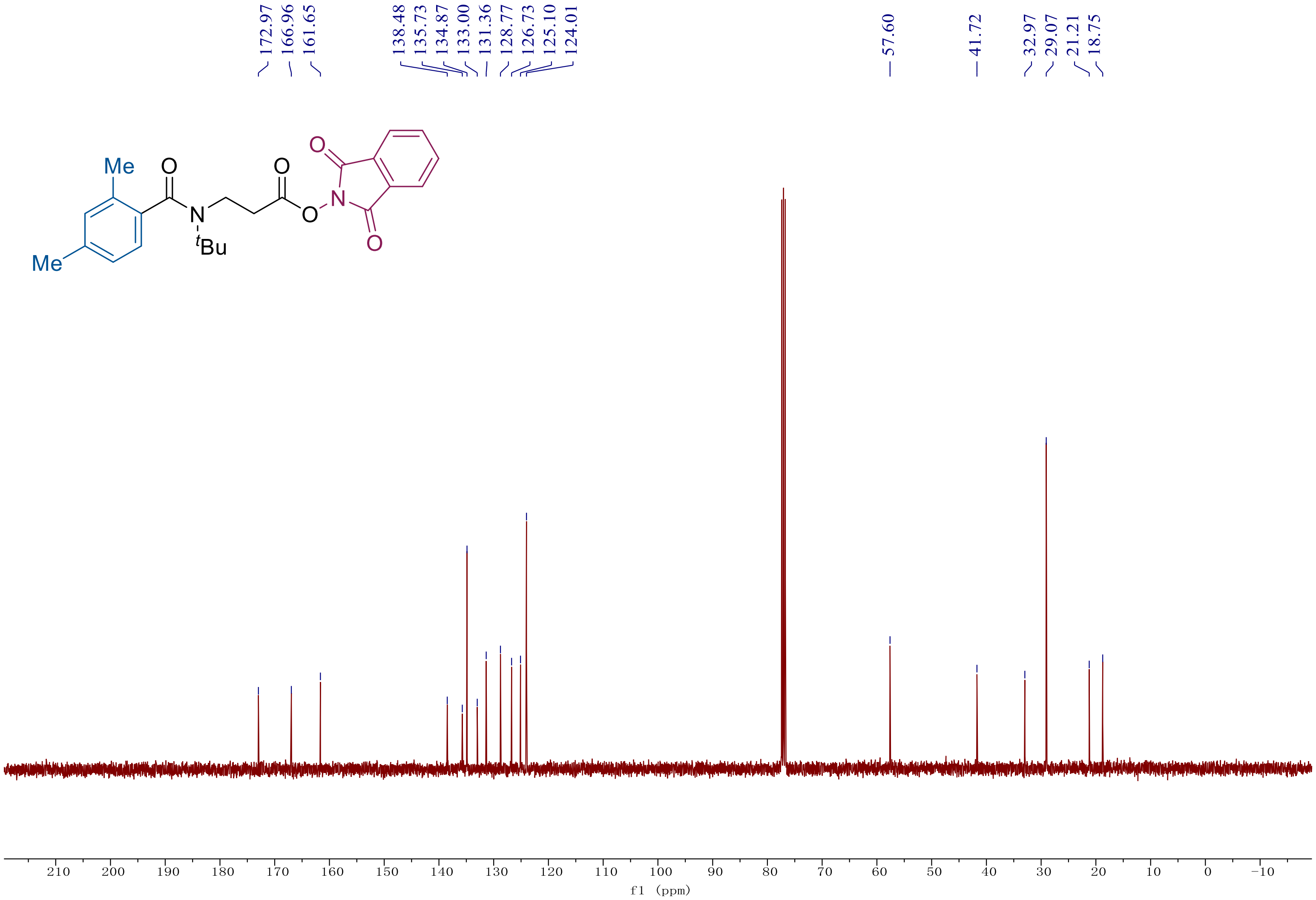

^{1}H NMR (500 MHz, CDCl$_3$) of **3i**

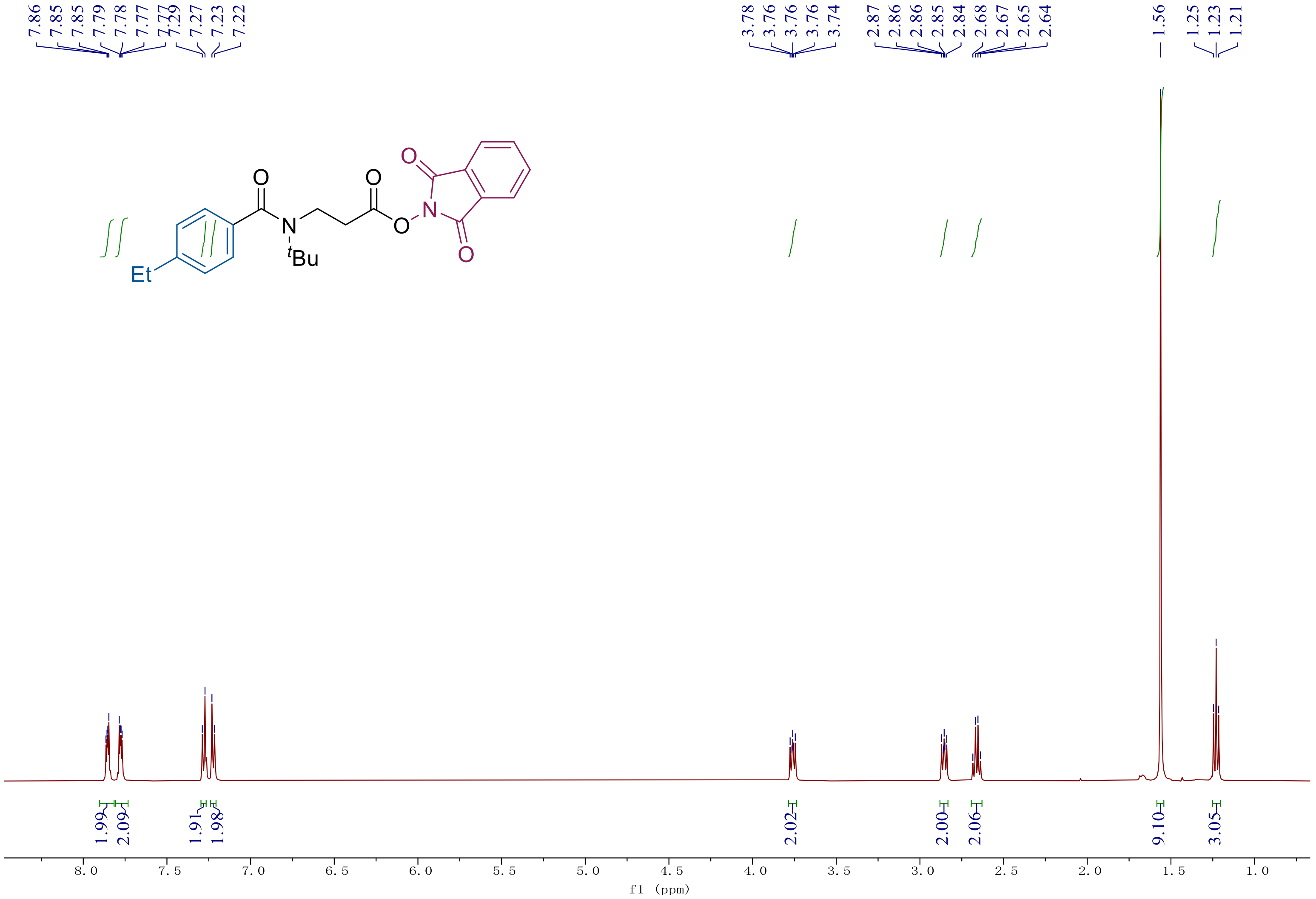

^{13}C NMR (126 MHz, $CDCl_3$) of **3i**

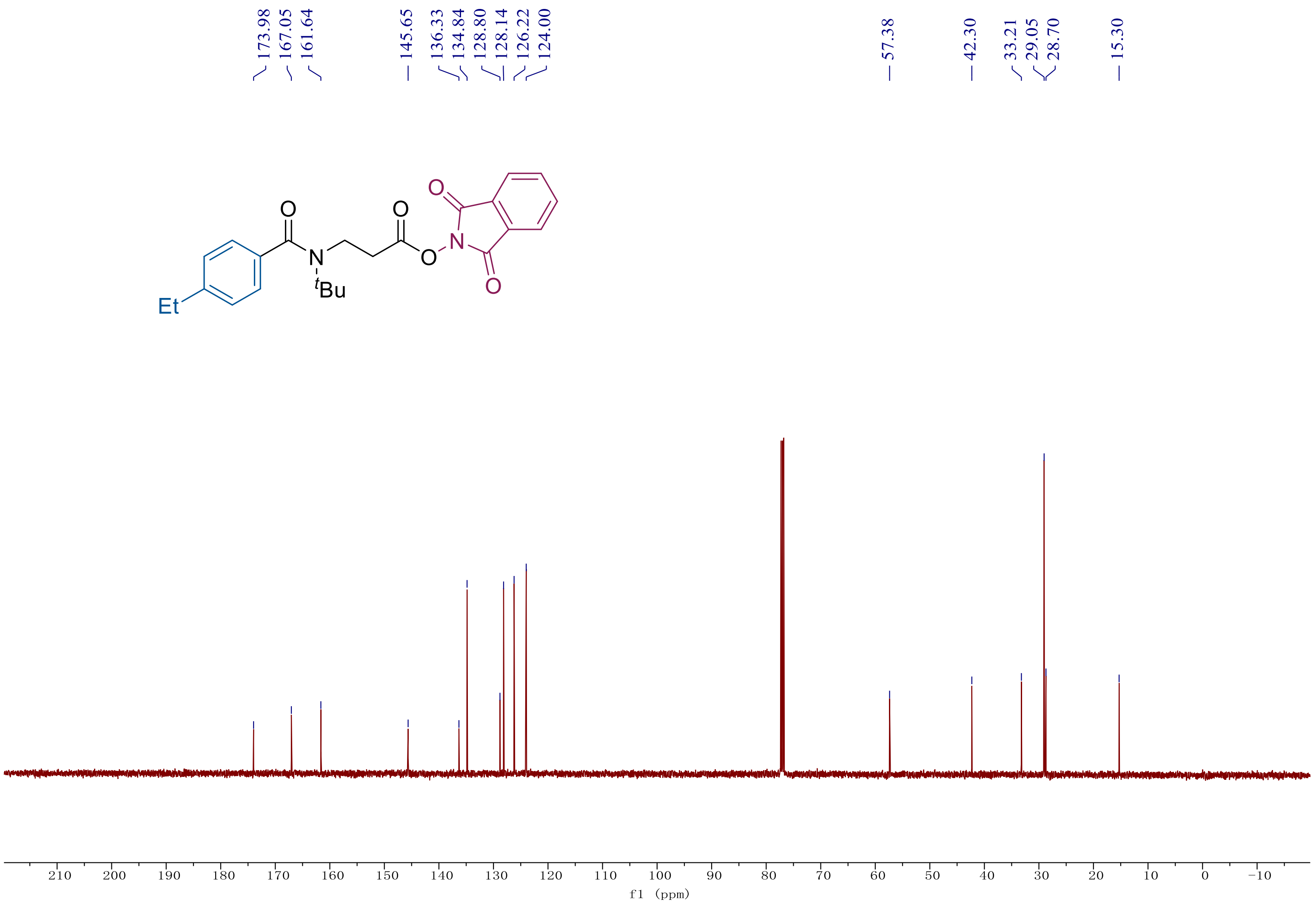

^{1}H NMR (500 MHz, $CDCl_3$) of **3j**

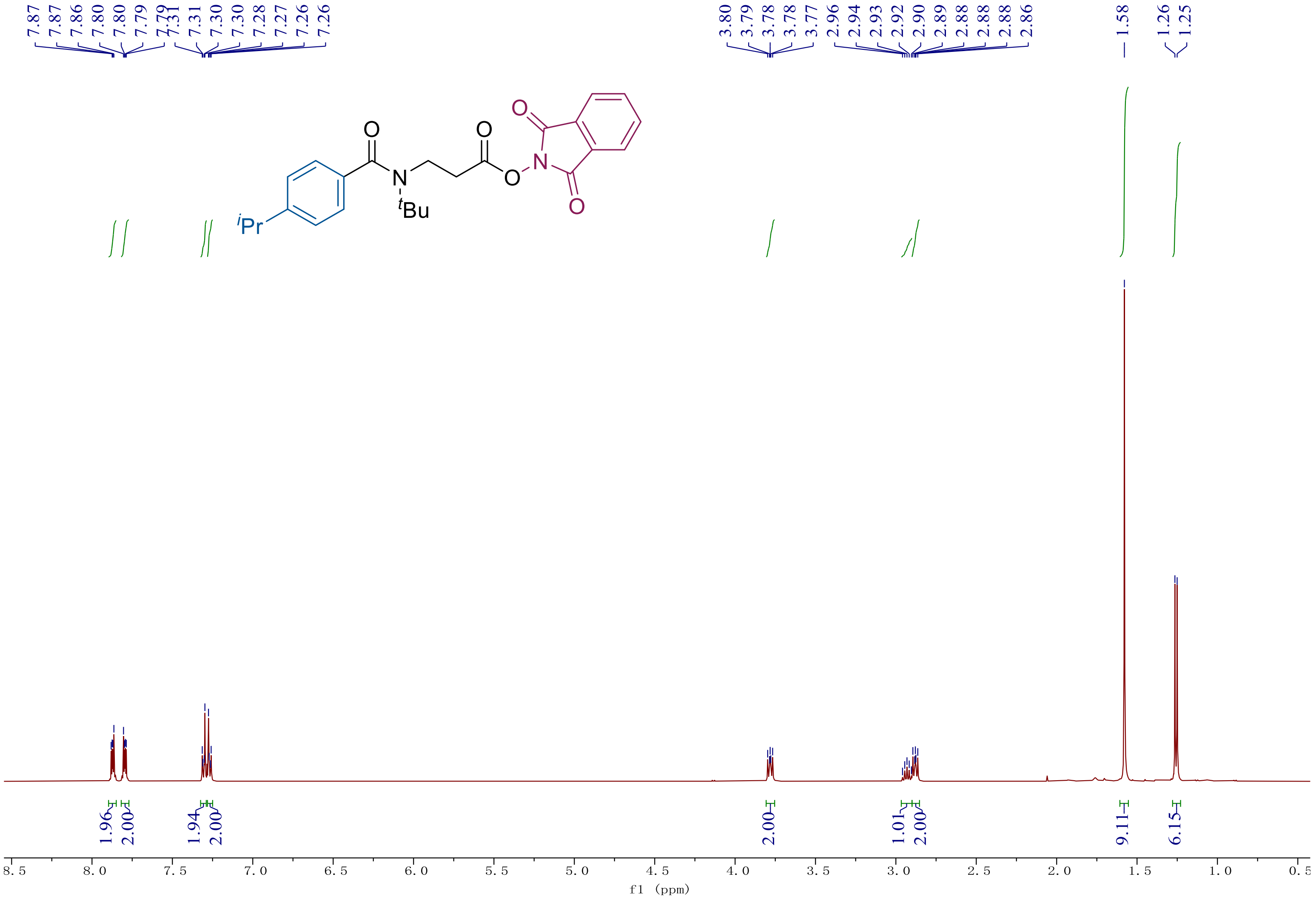

^{13}C NMR (126 MHz, $CDCl_3$) of **3j**

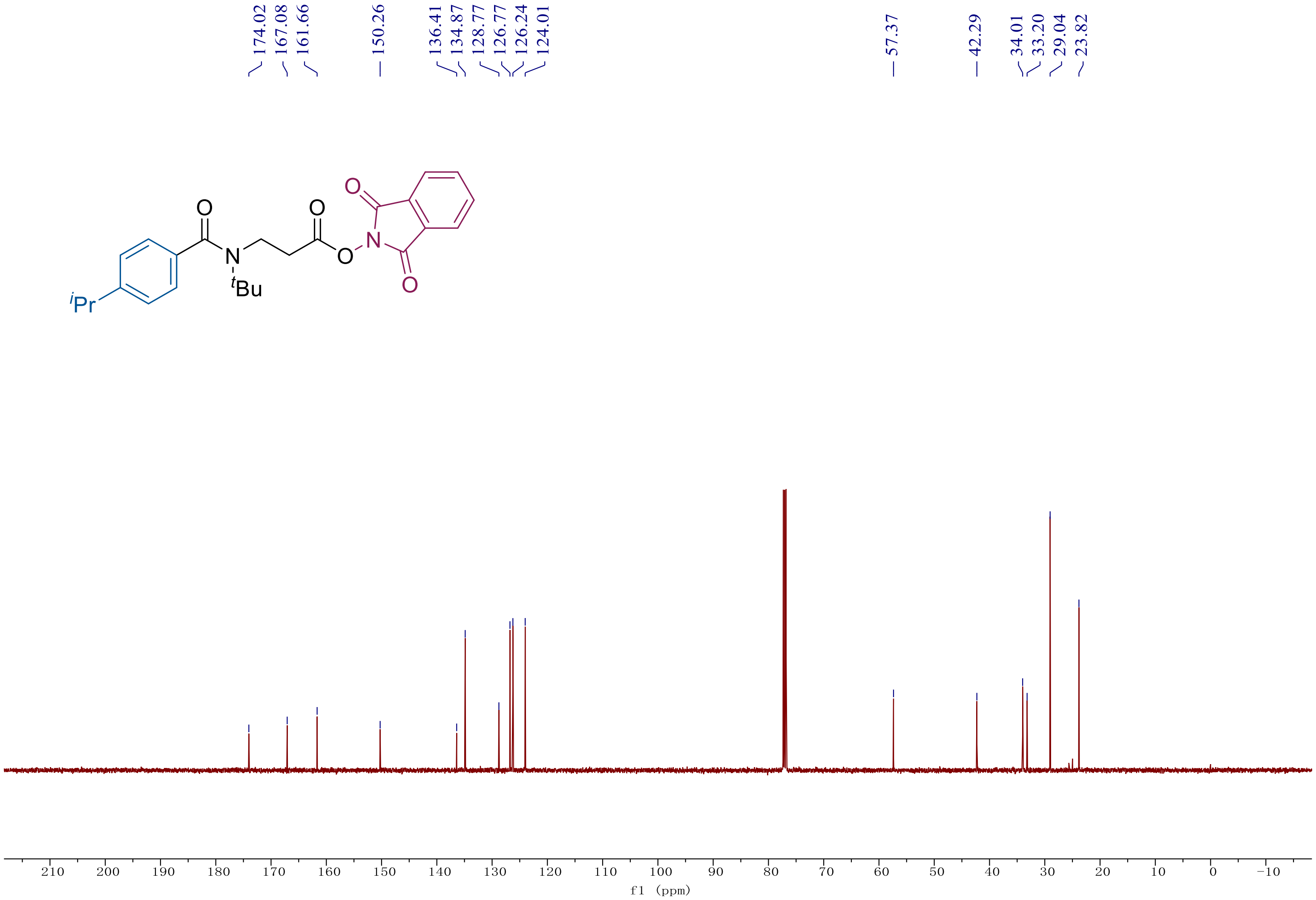

^{1}H NMR (500 MHz, $CDCl_3$) of **3k**

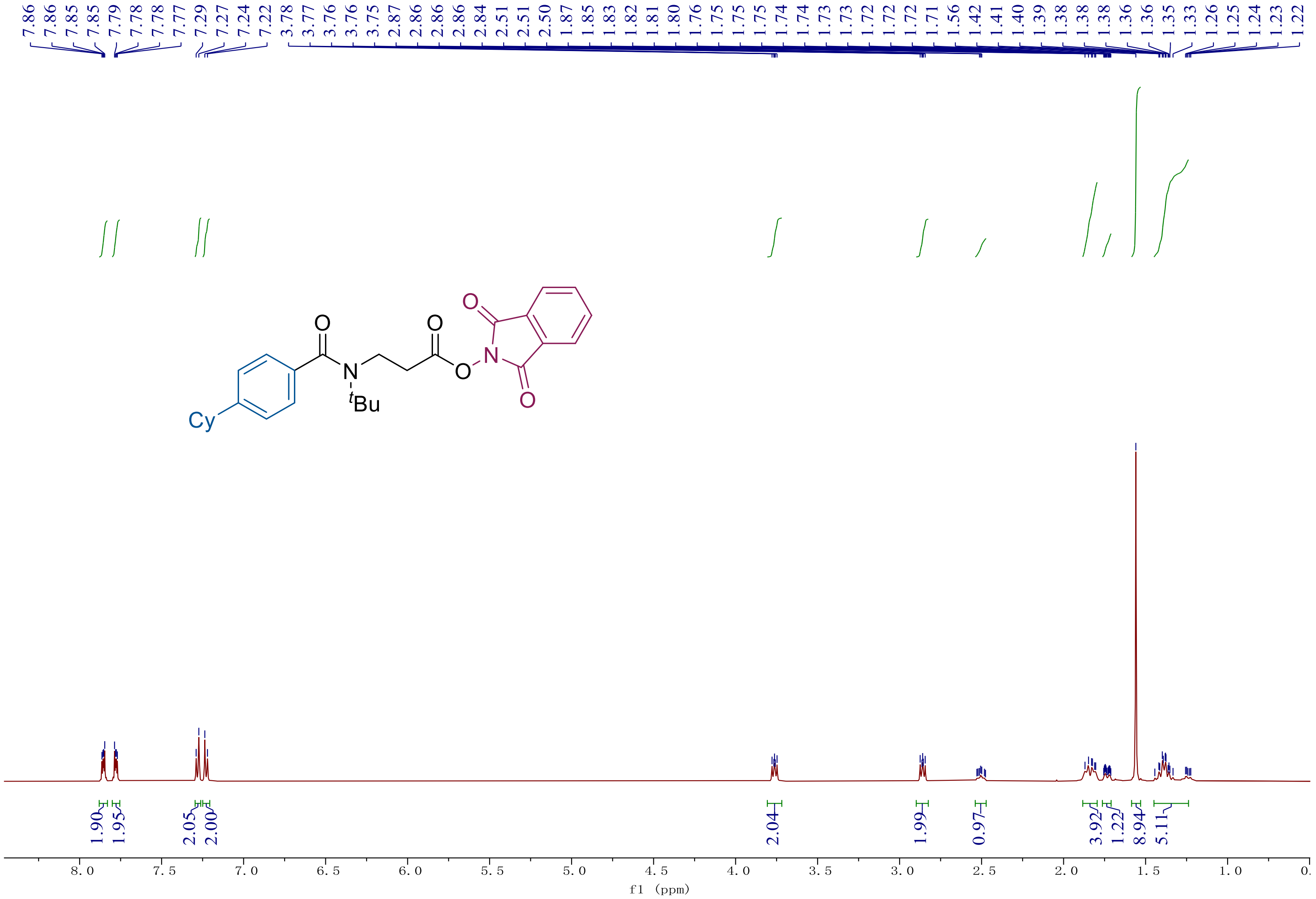

^{13}C NMR (126 MHz, CDCl$_3$) of **3k**

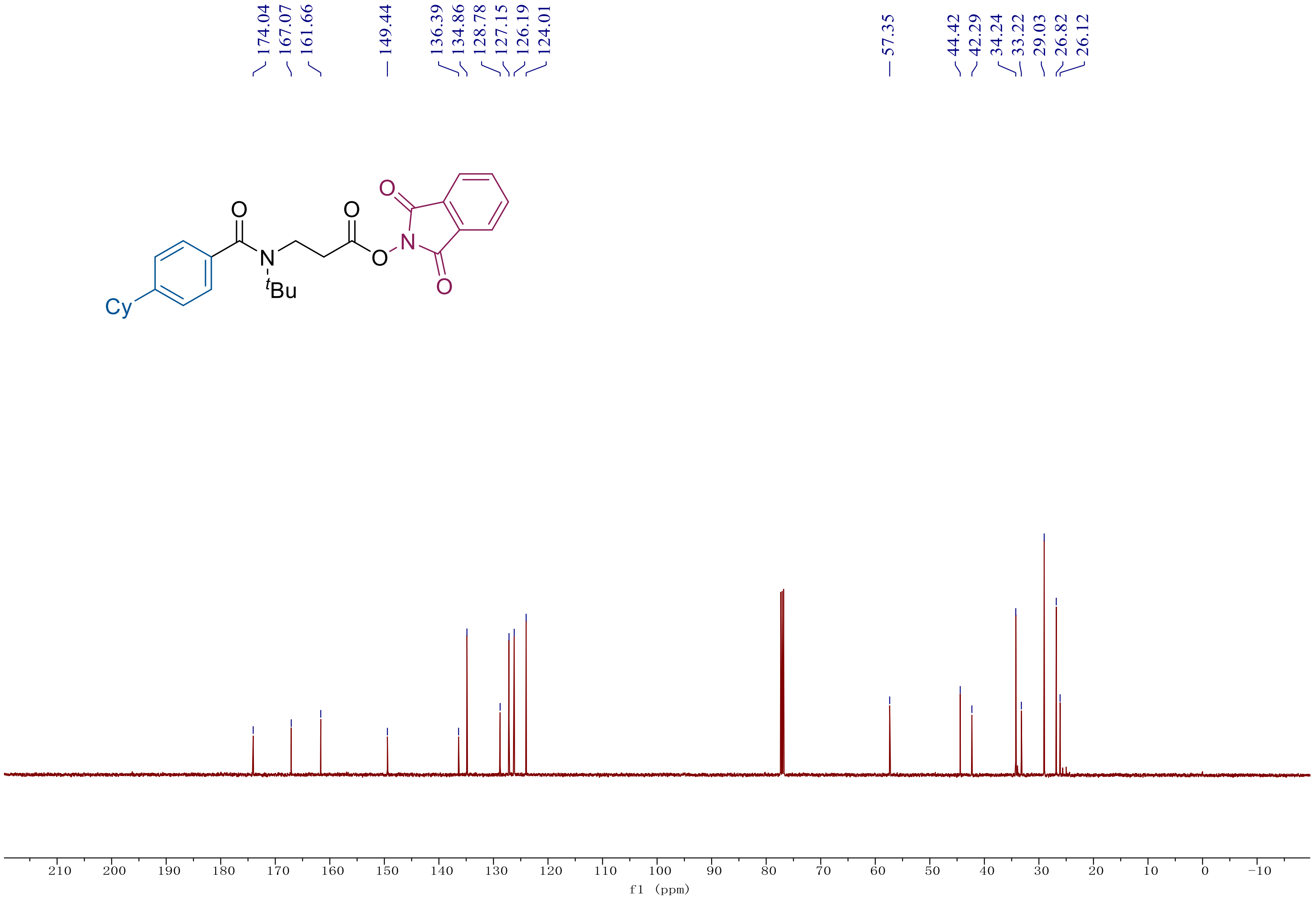

^{1}H NMR (400 MHz, $CDCl_3$) of **3l**

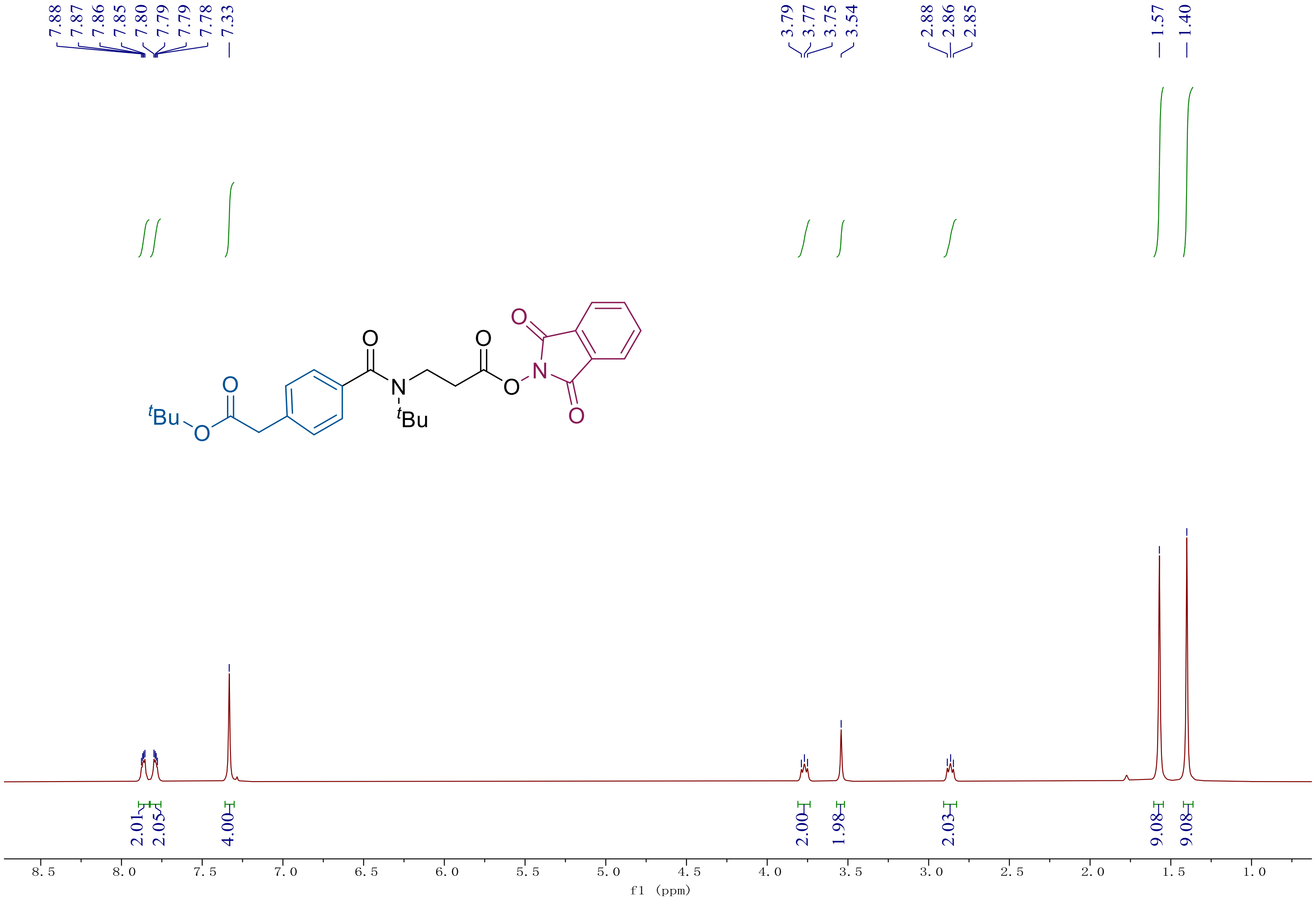

^{13}C NMR (101 MHz, $CDCl_3$) of **3l**

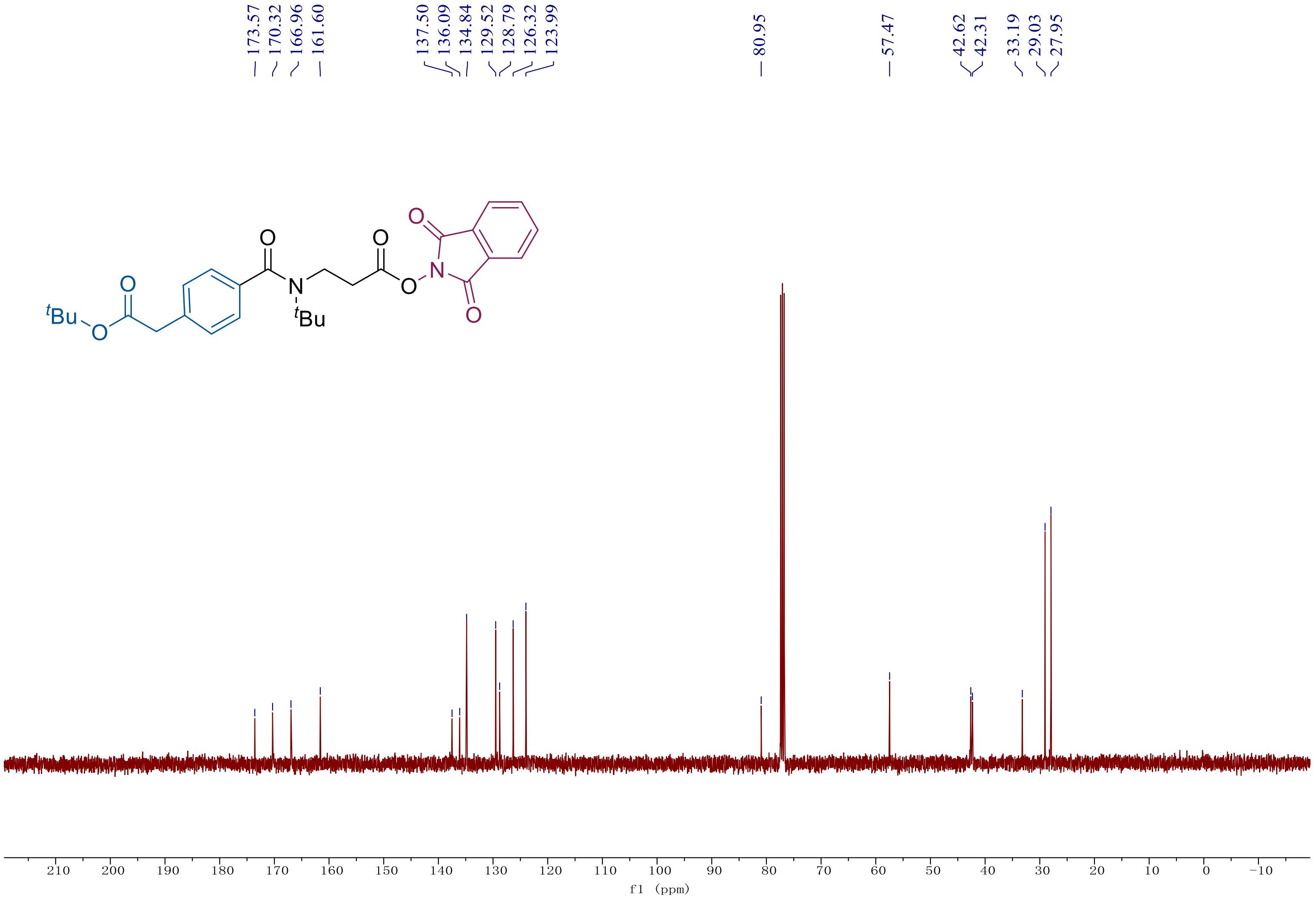

^{1}H NMR (400 MHz, $CDCl_3$) of **3m**

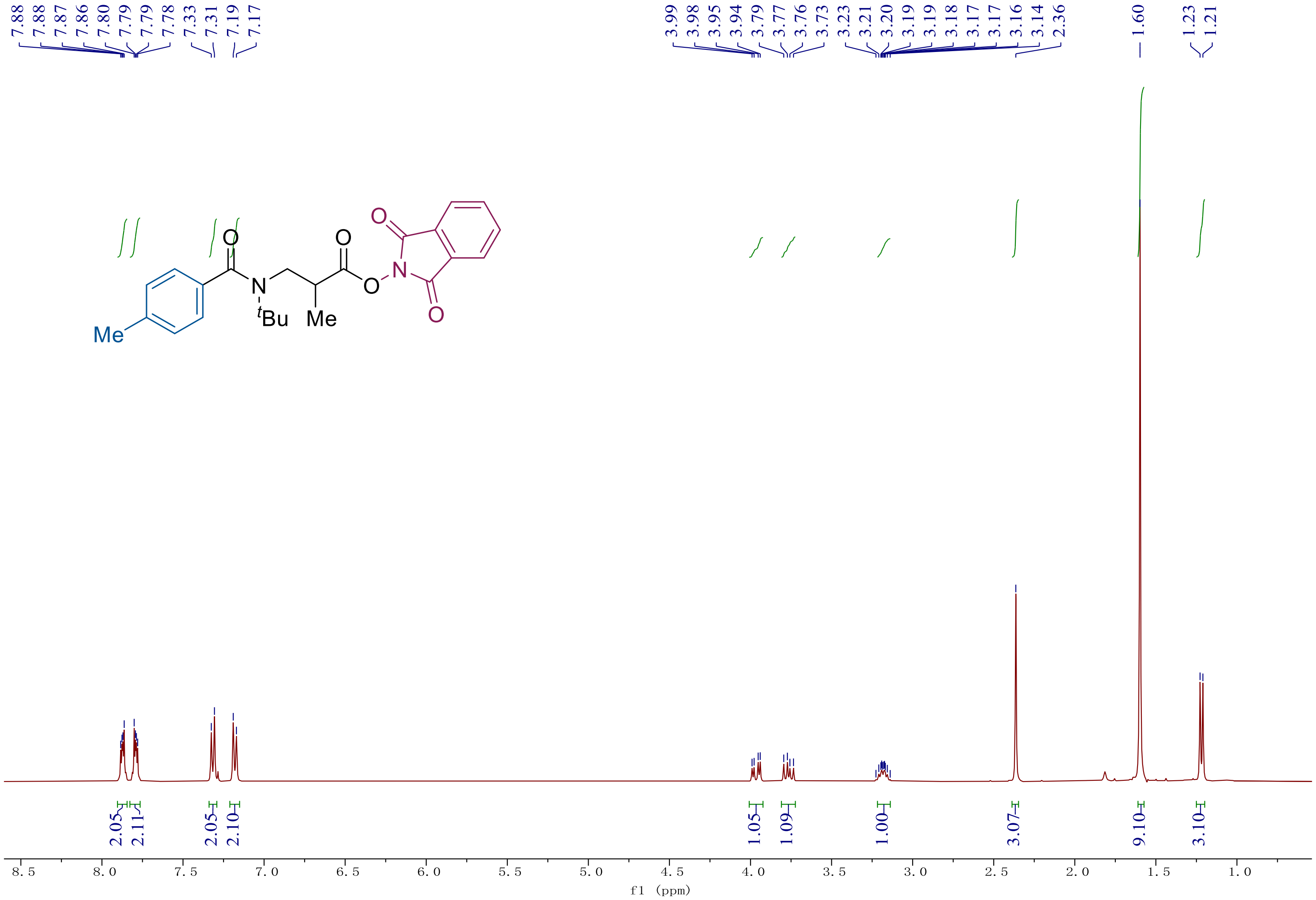

^{13}C NMR (101 MHz, $CDCl_3$) of **3m**

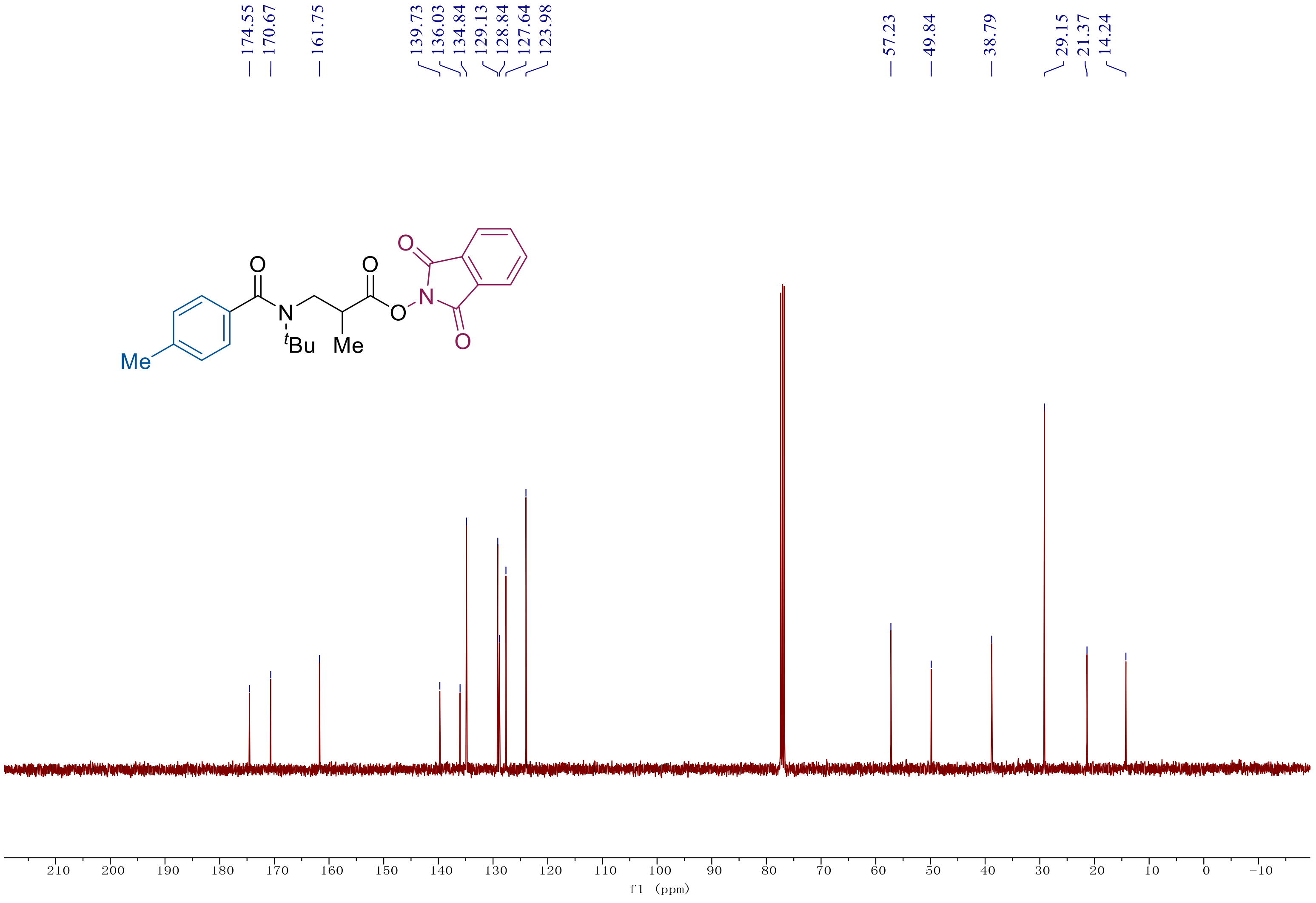

^{1}H NMR (400 MHz, $CDCl_3$) of **3n**

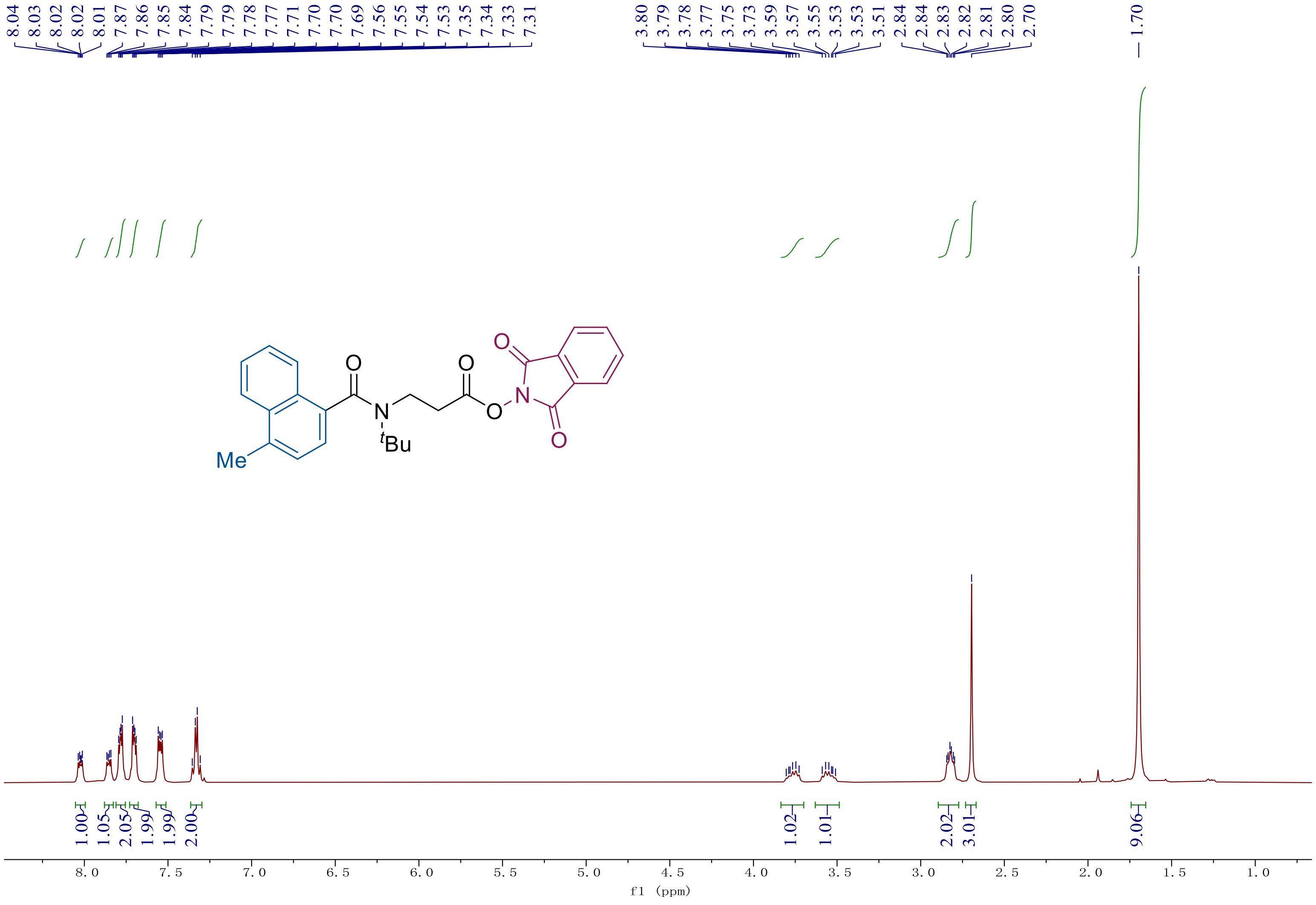

^{13}C NMR (101 MHz, CDCl$_3$) of **3n**

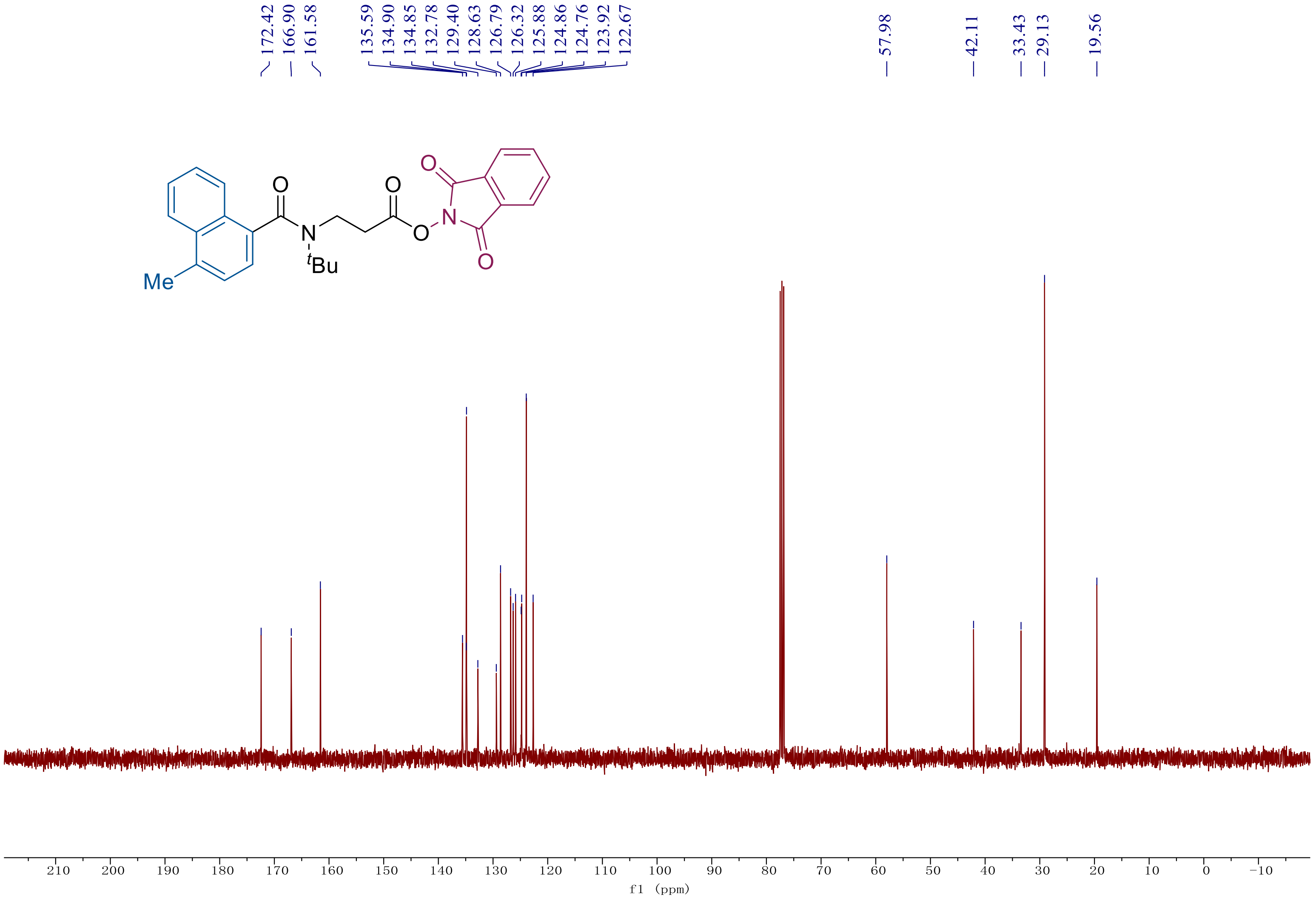

^{1}H NMR (400 MHz, $CDCl_3$) of **3o**

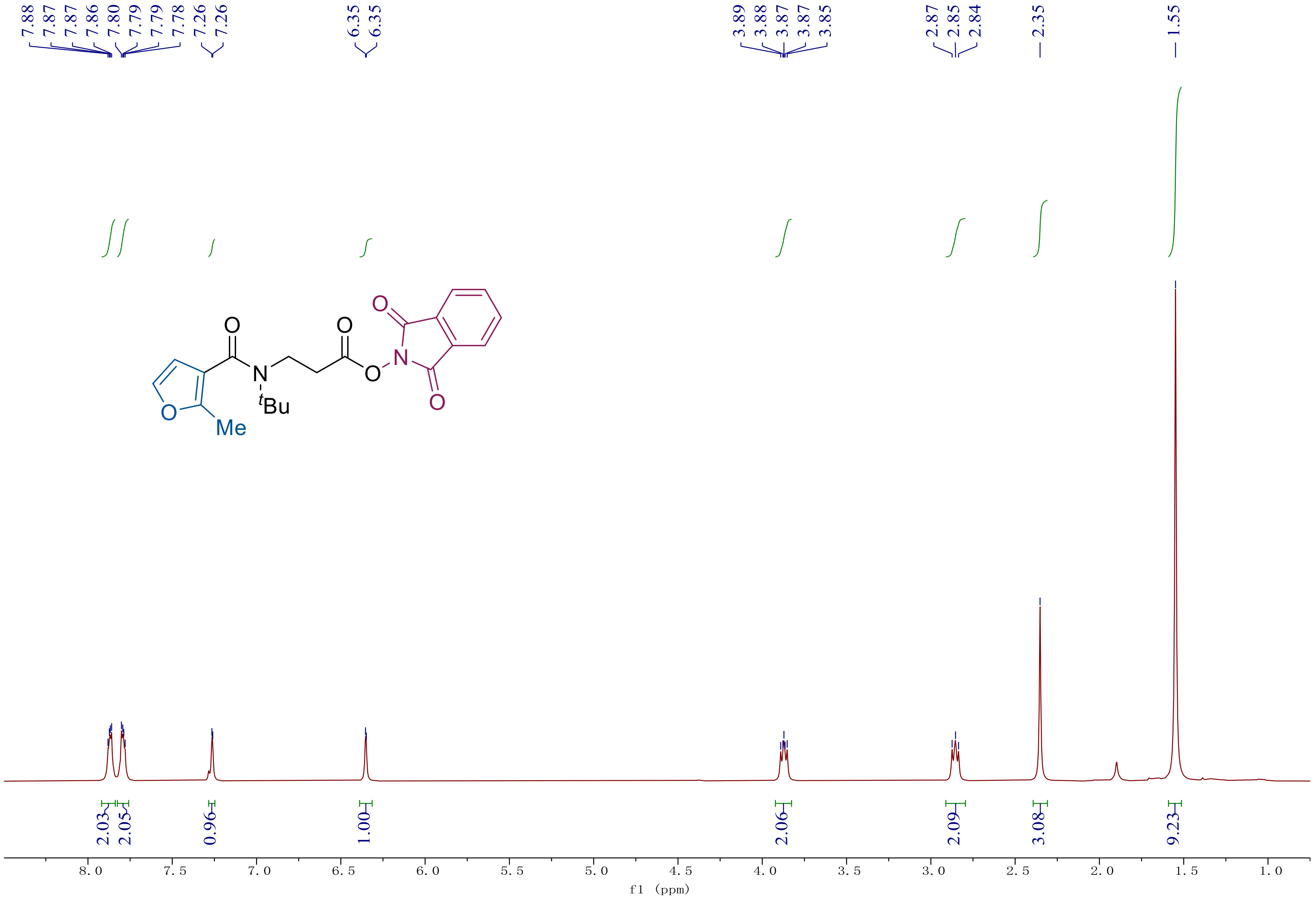

^{13}C NMR (101 MHz, $CDCl_3$) of **3o**

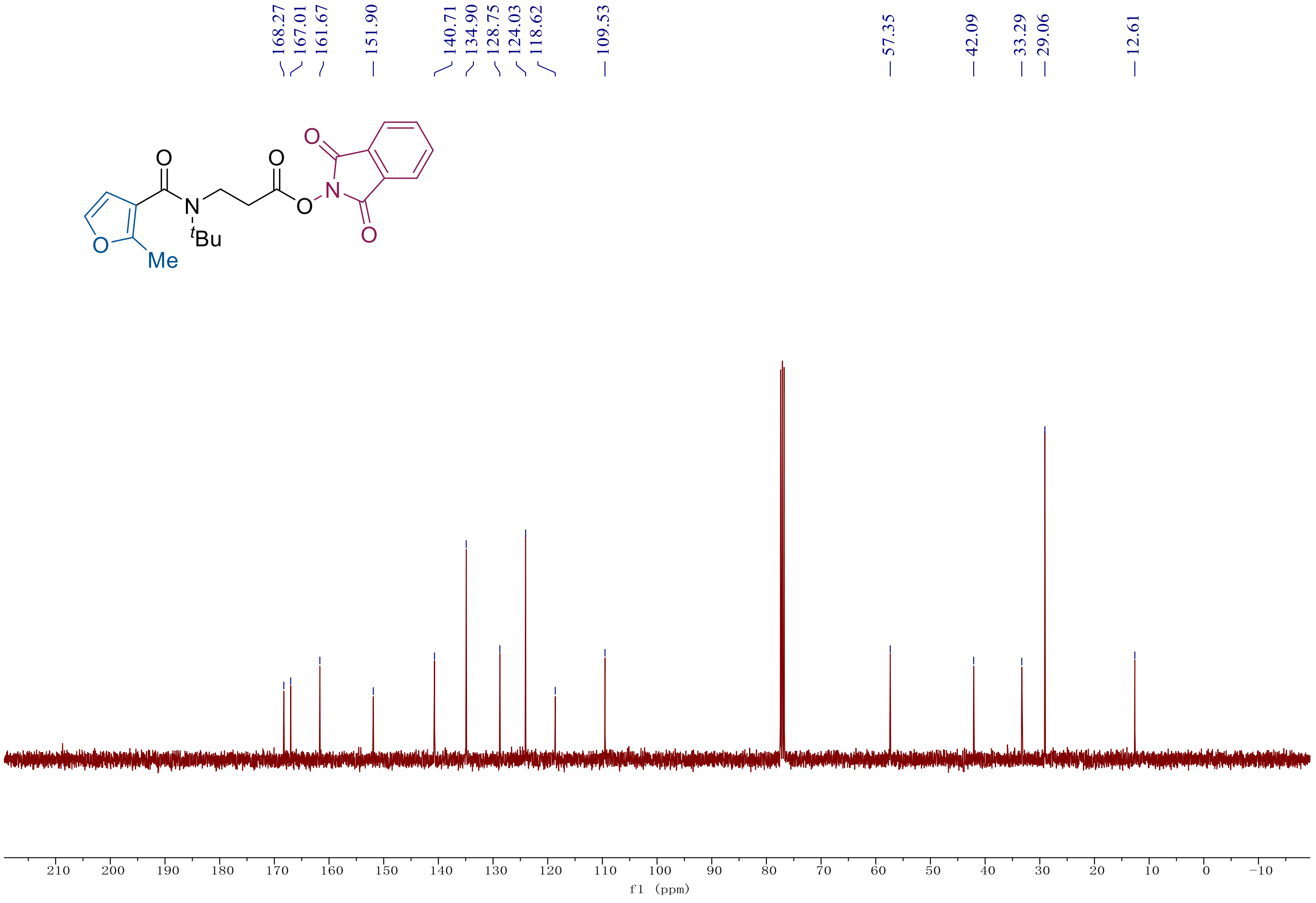

^{1}H NMR (400 MHz, $CDCl_3$) of **3p**

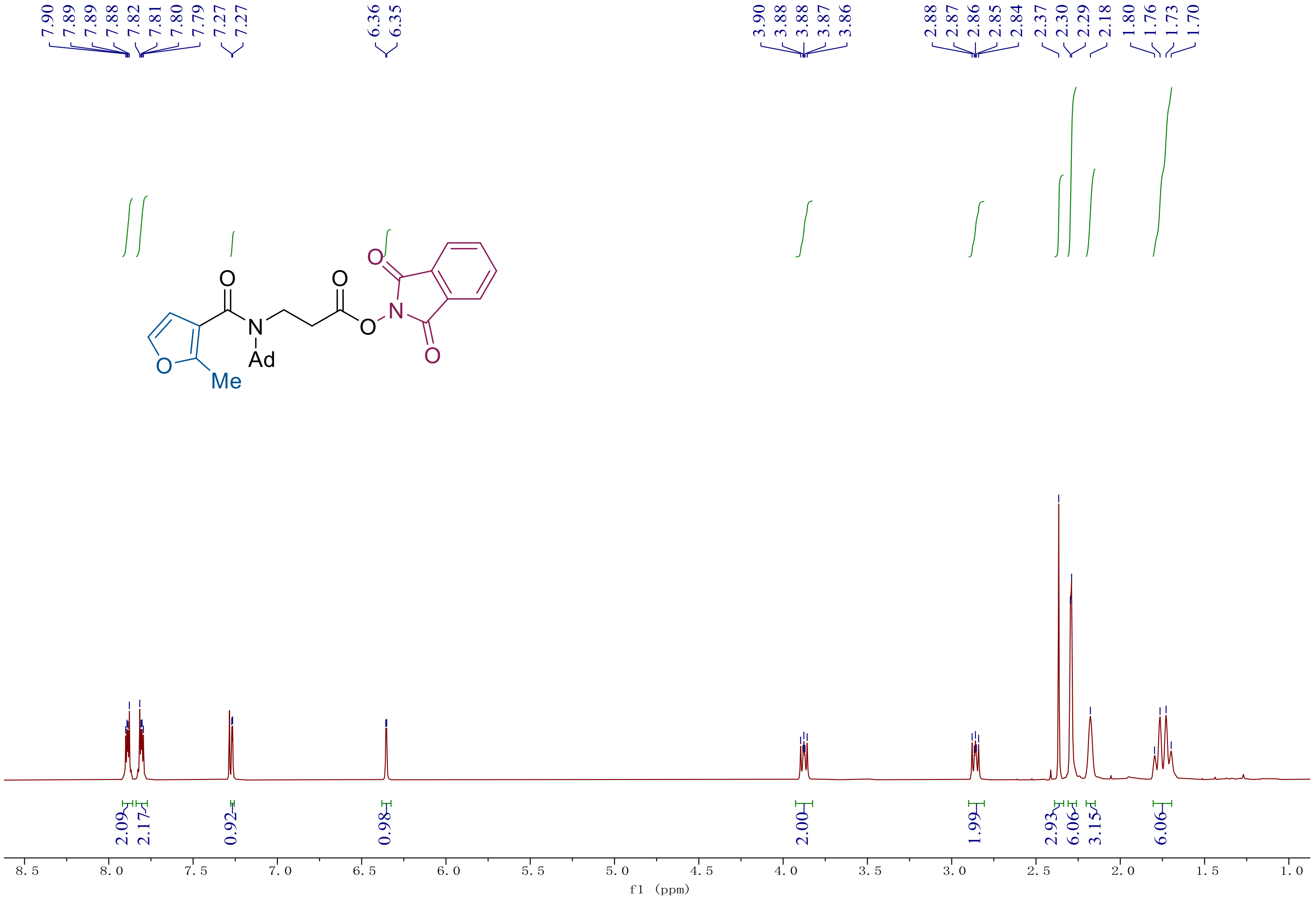

^{13}C NMR (101 MHz, $CDCl_3$) of **3p**

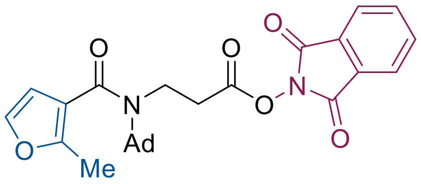

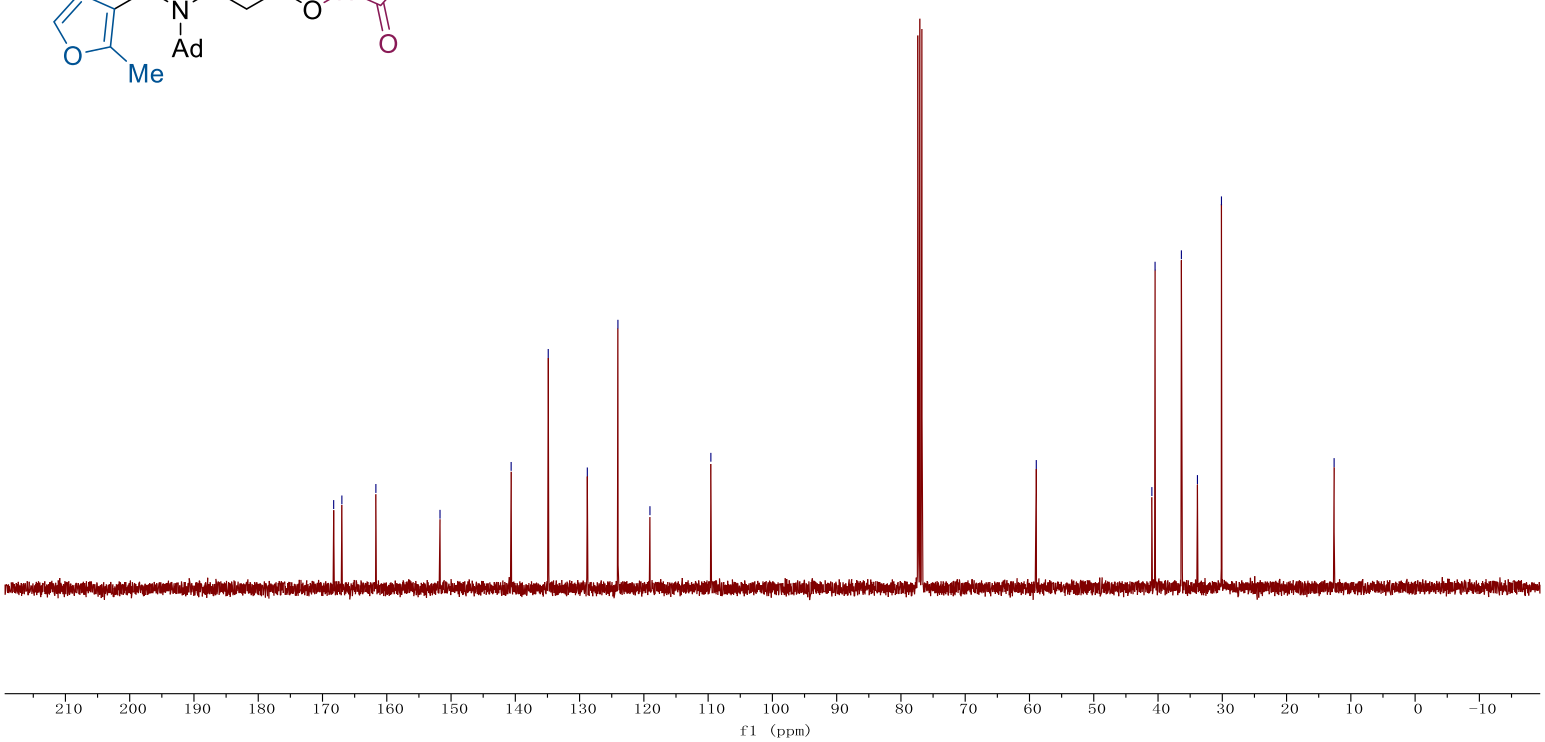

^{1}H NMR (400 MHz, CDCl$_3$) of **3q**

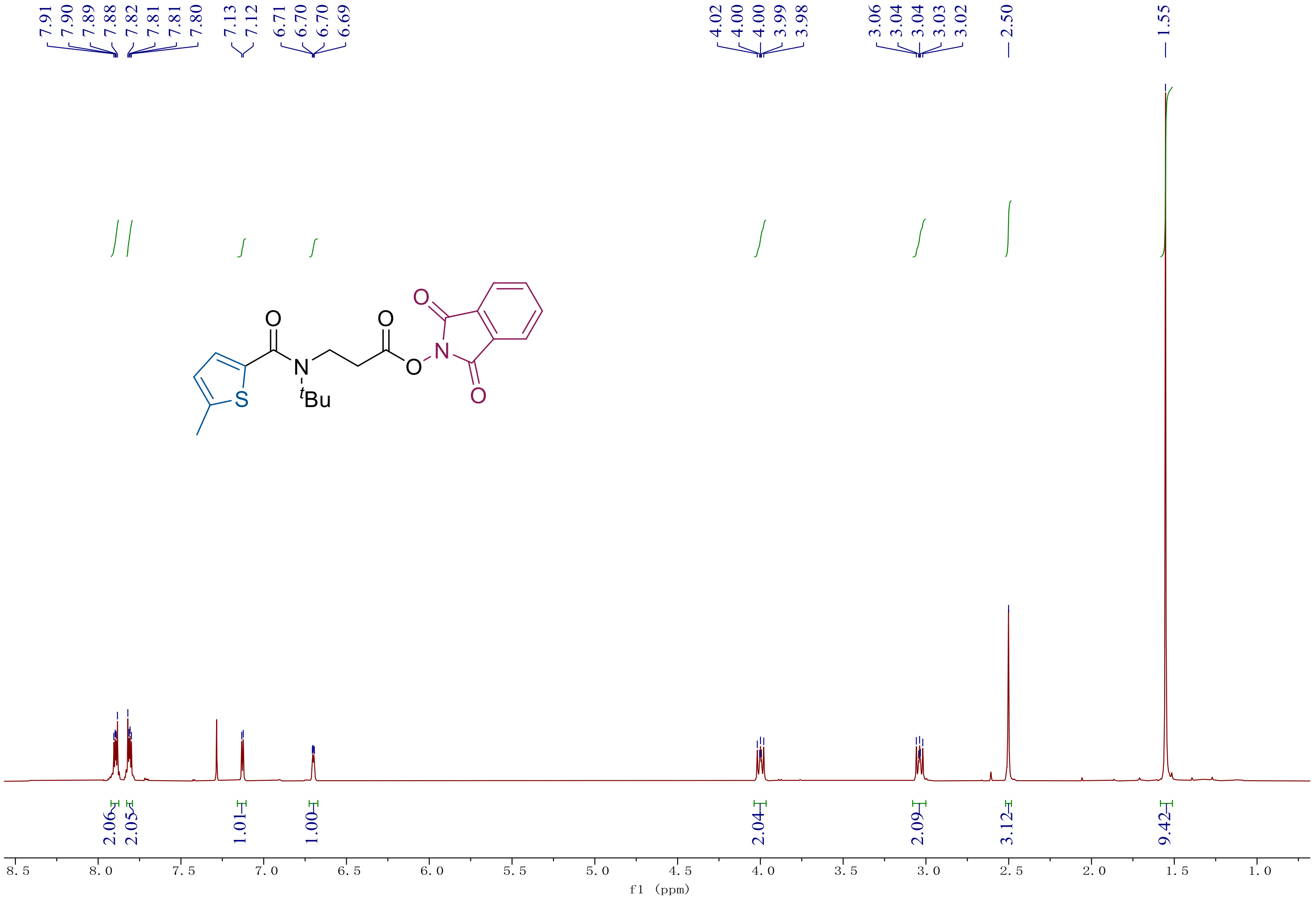

^{13}C NMR (101 MHz, $CDCl_3$) of **3q**

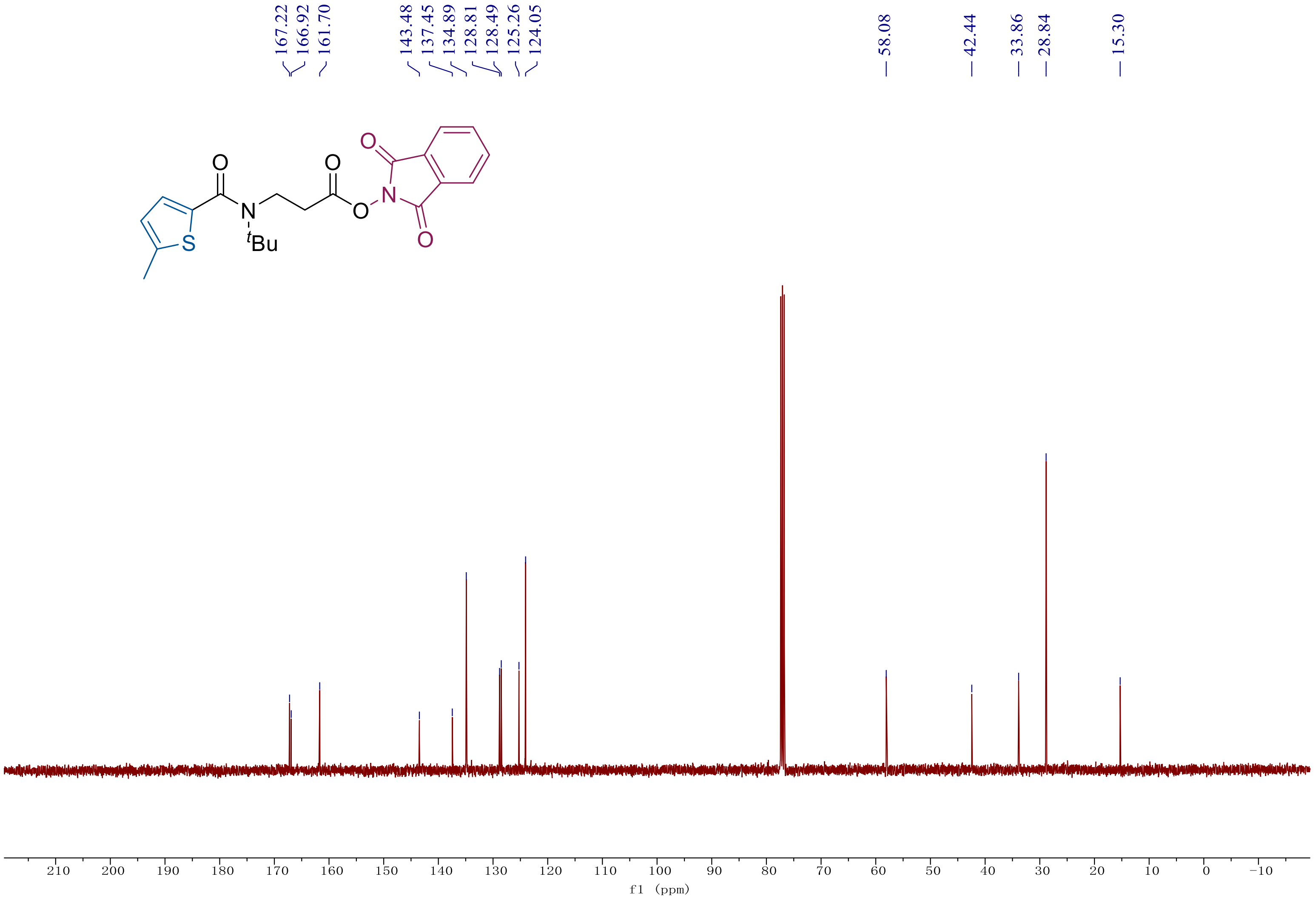

^{1}H NMR (400 MHz, $CDCl_3$) of **3r**

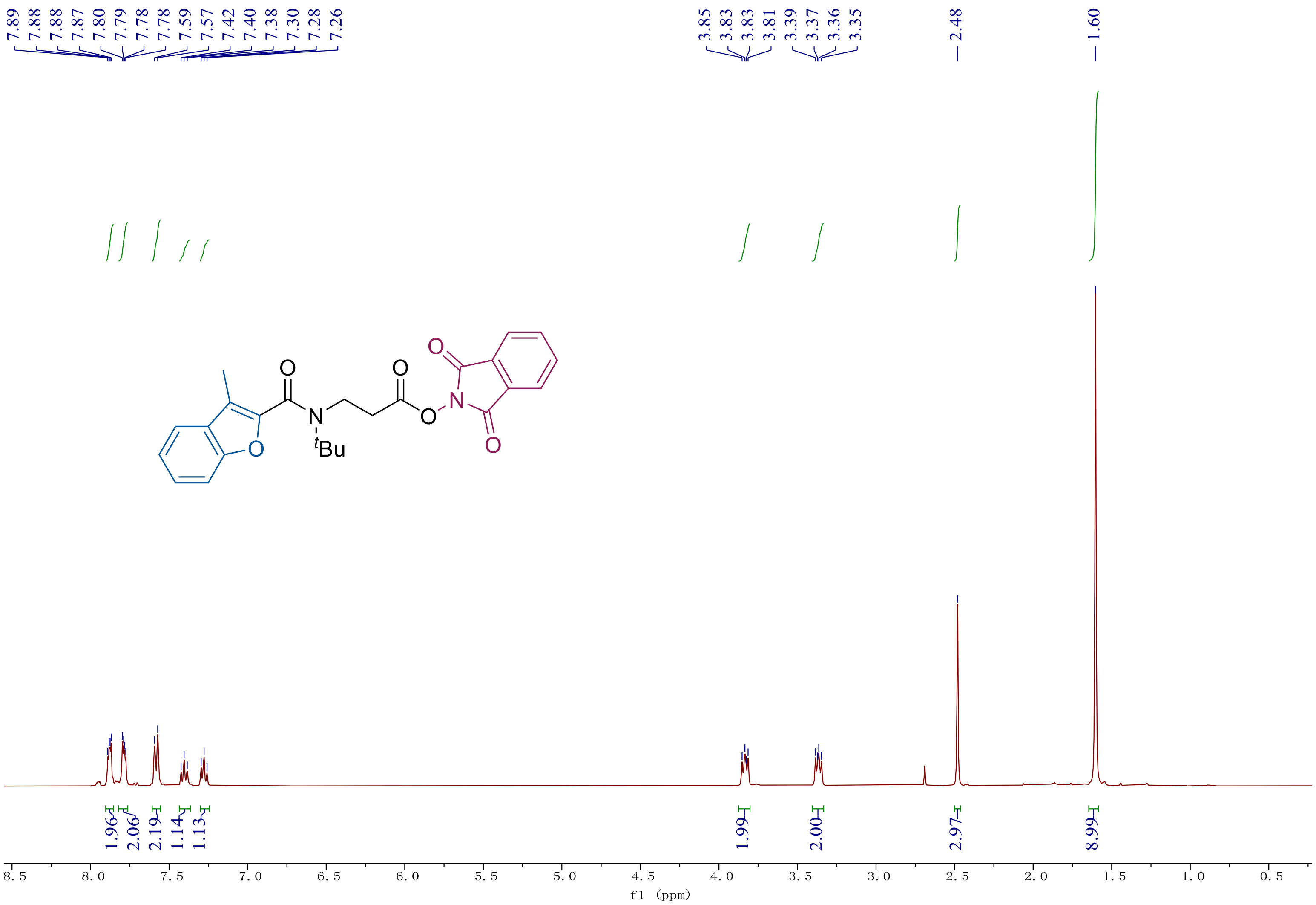

^{13}C NMR (101 MHz, $CDCl_3$) of **3r**

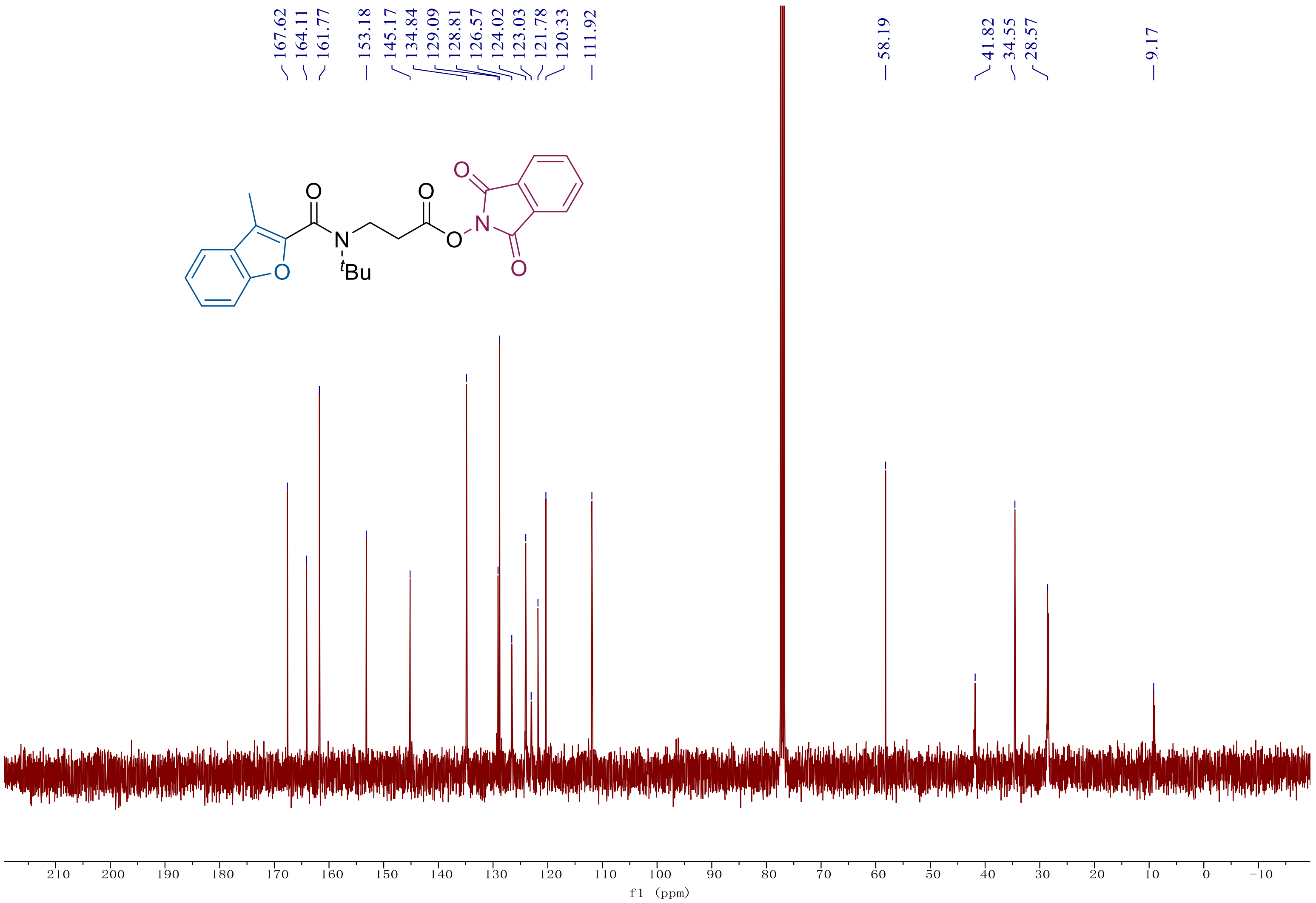

^{1}H NMR (500 MHz, $CDCl_3$) of **2a**

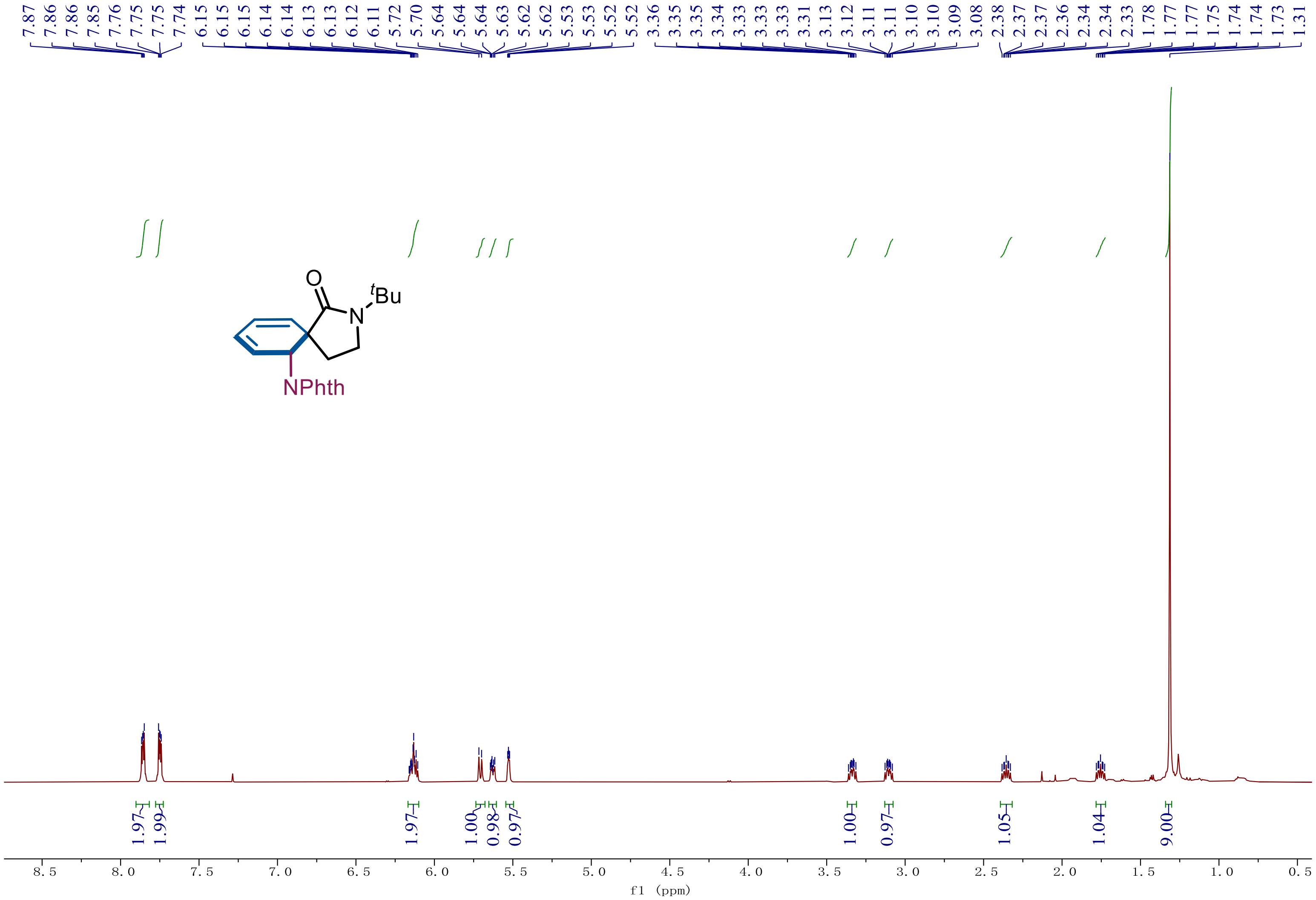

^{13}C NMR (126 MHz, $CDCl_3$) of **2a**

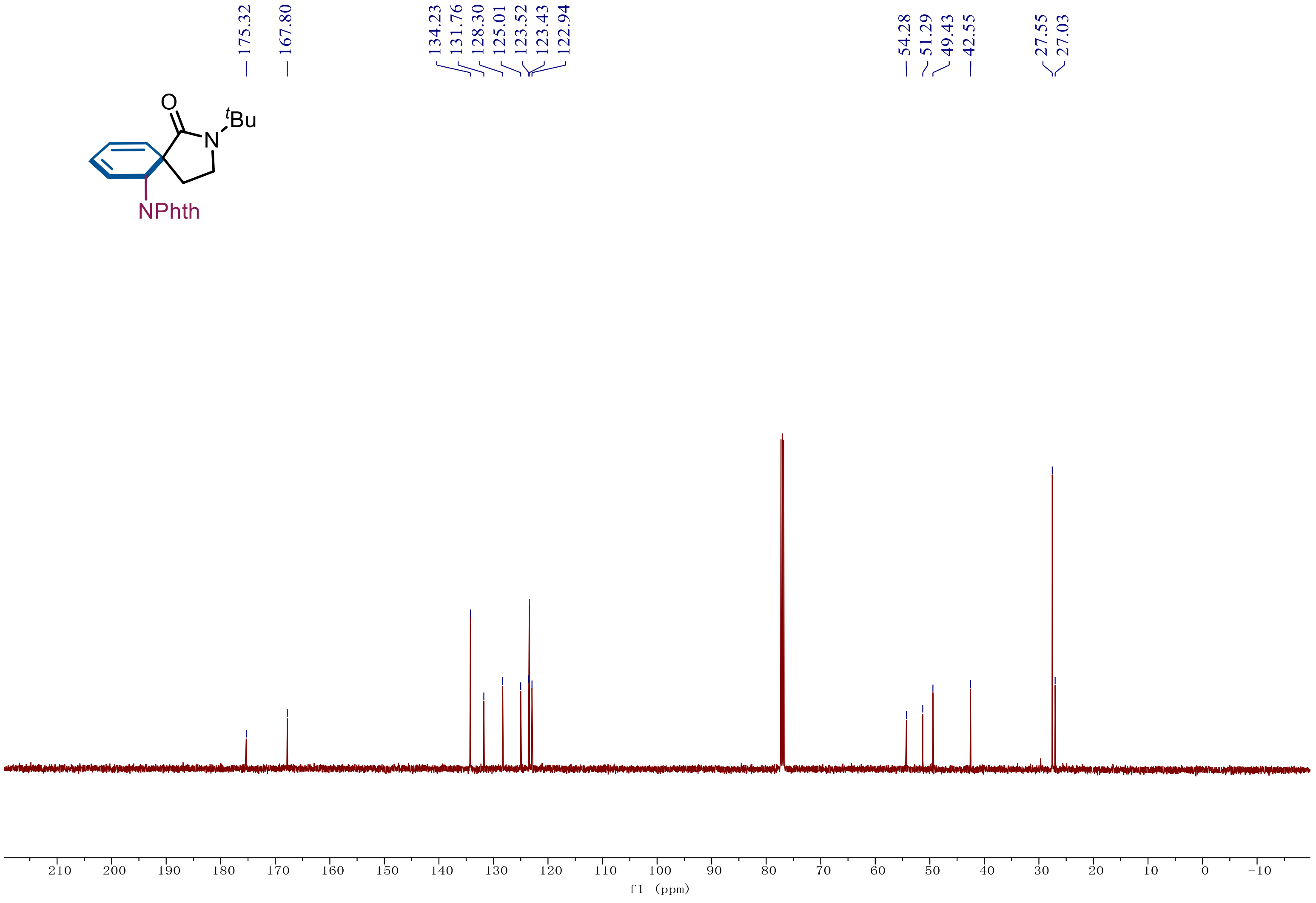

^{1}H NMR (400 MHz, $CDCl_3$) of **2b**

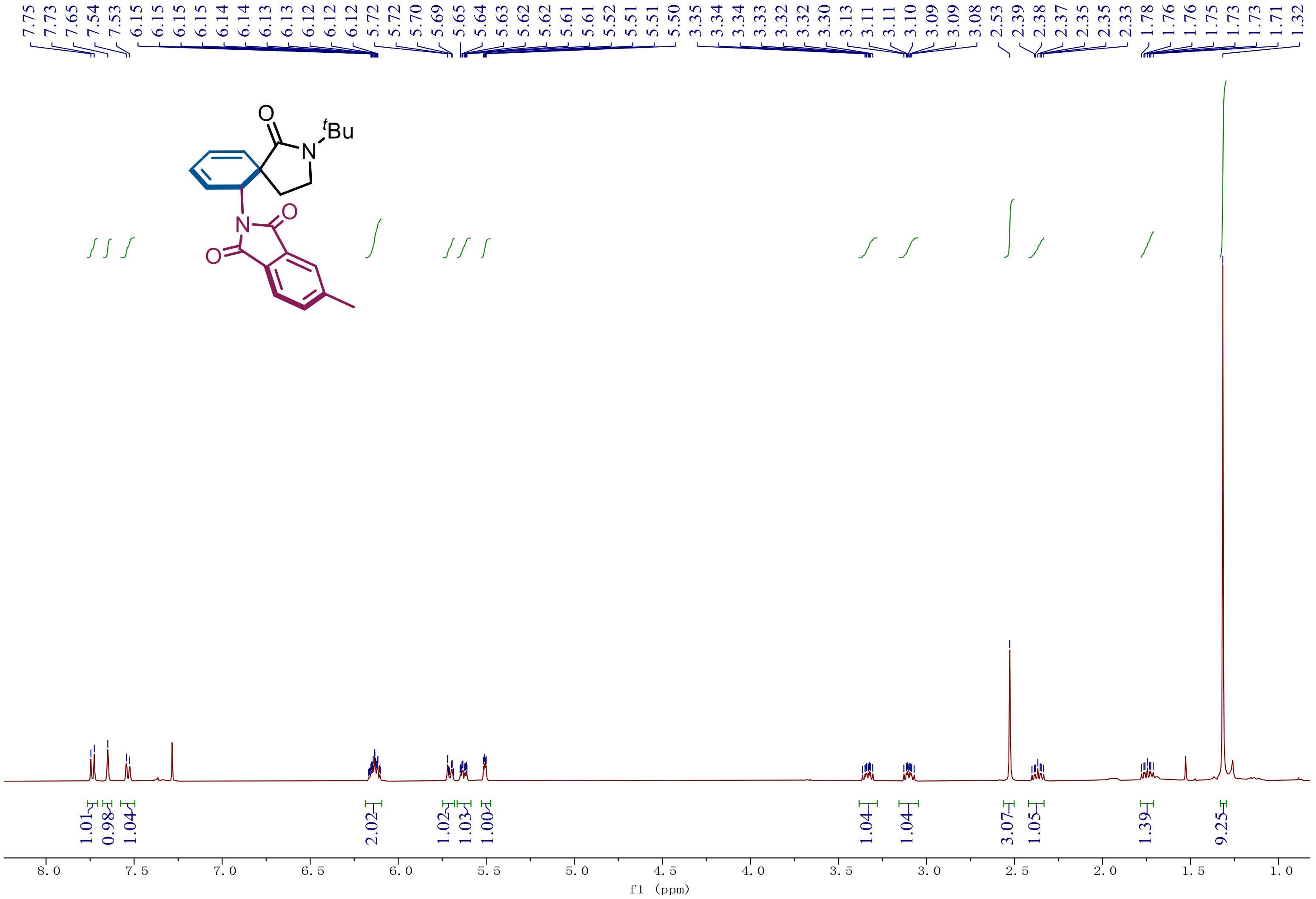

^{13}C NMR (101 MHz, $CDCl_3$) of **2b**

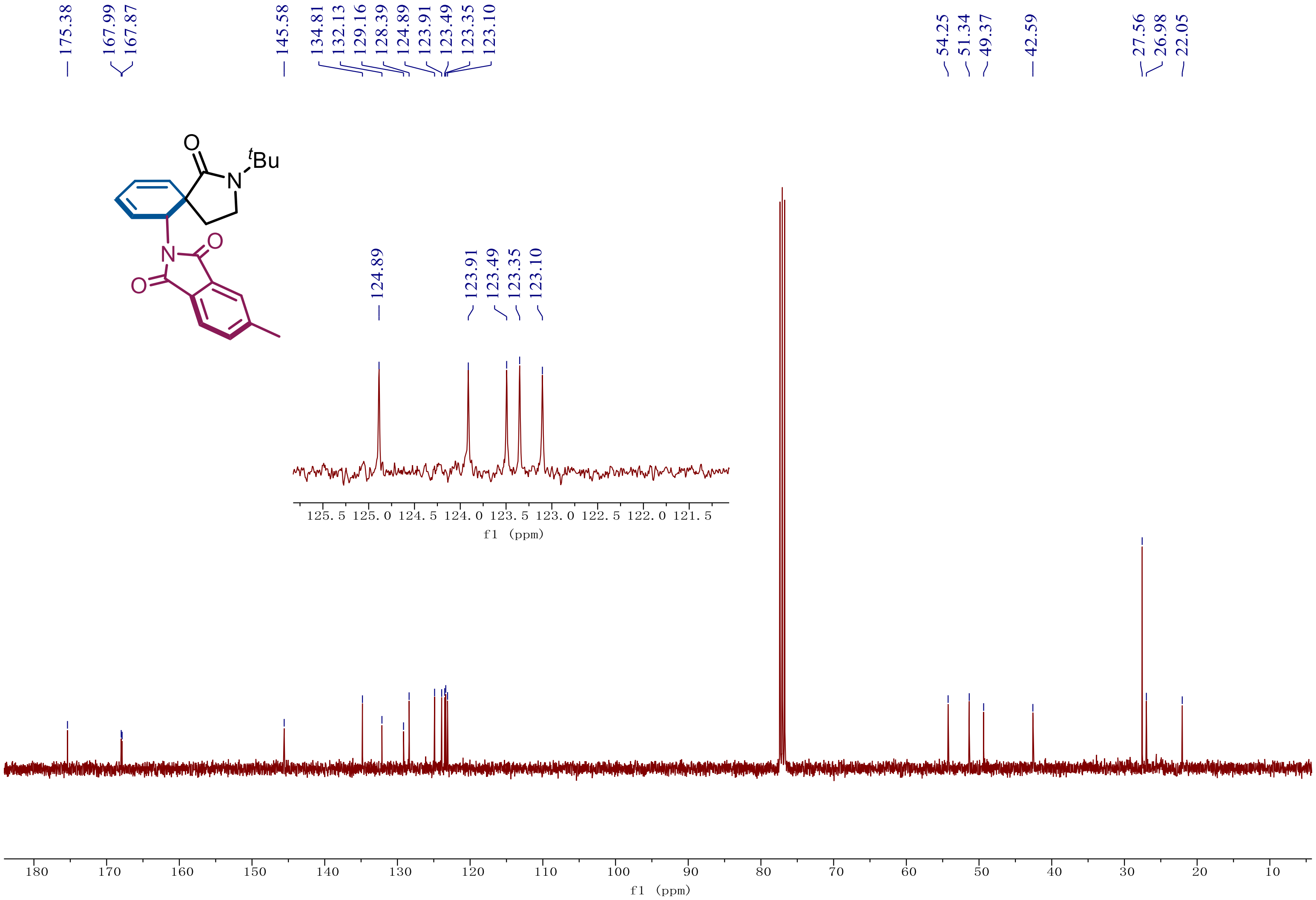

^{1}H NMR (400 MHz, $CDCl_3$) of **2c**

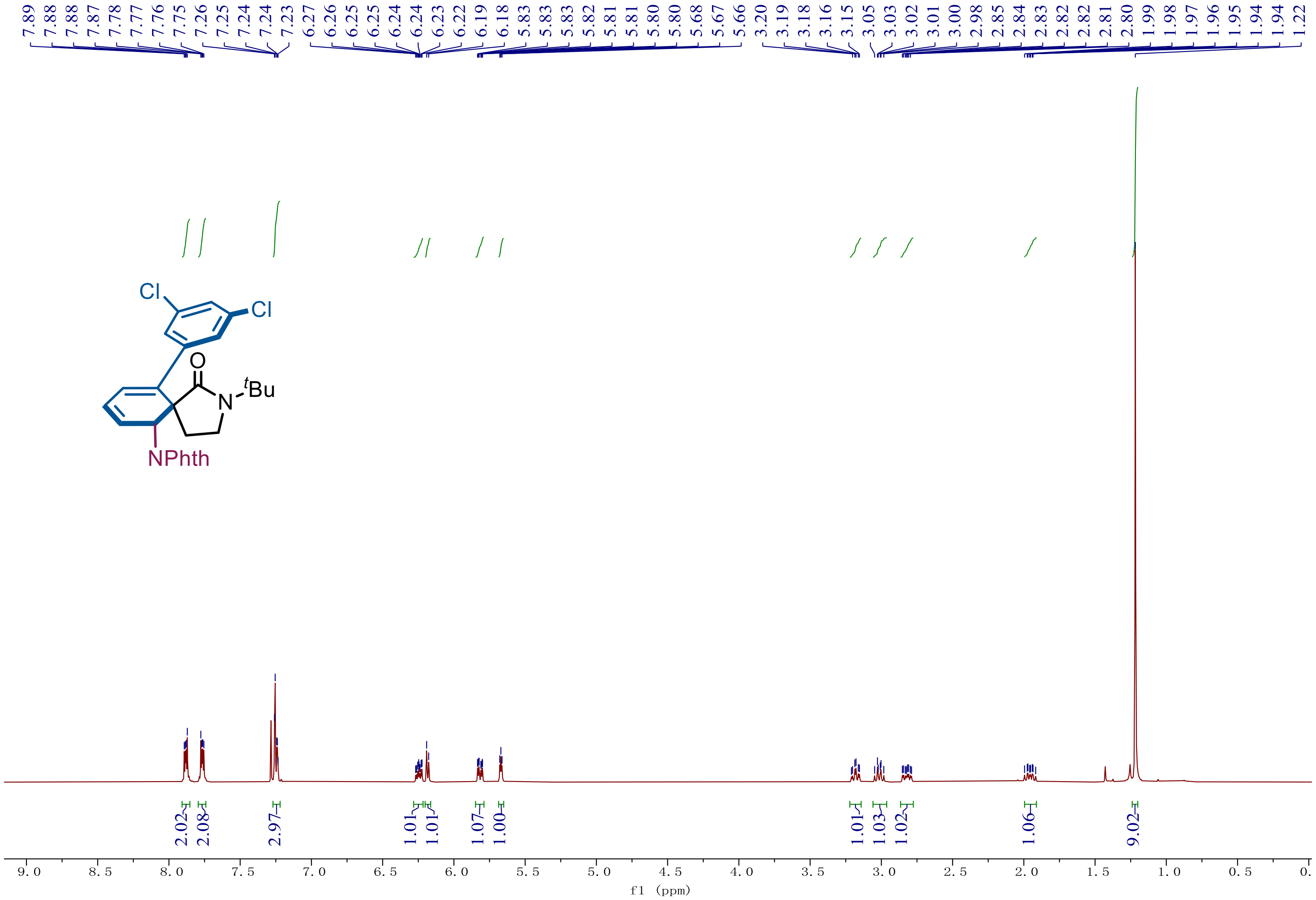

^{13}C NMR (101 MHz, CDCl$_3$) of **2c**

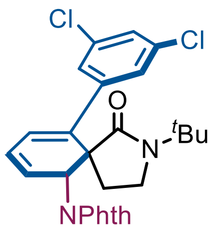

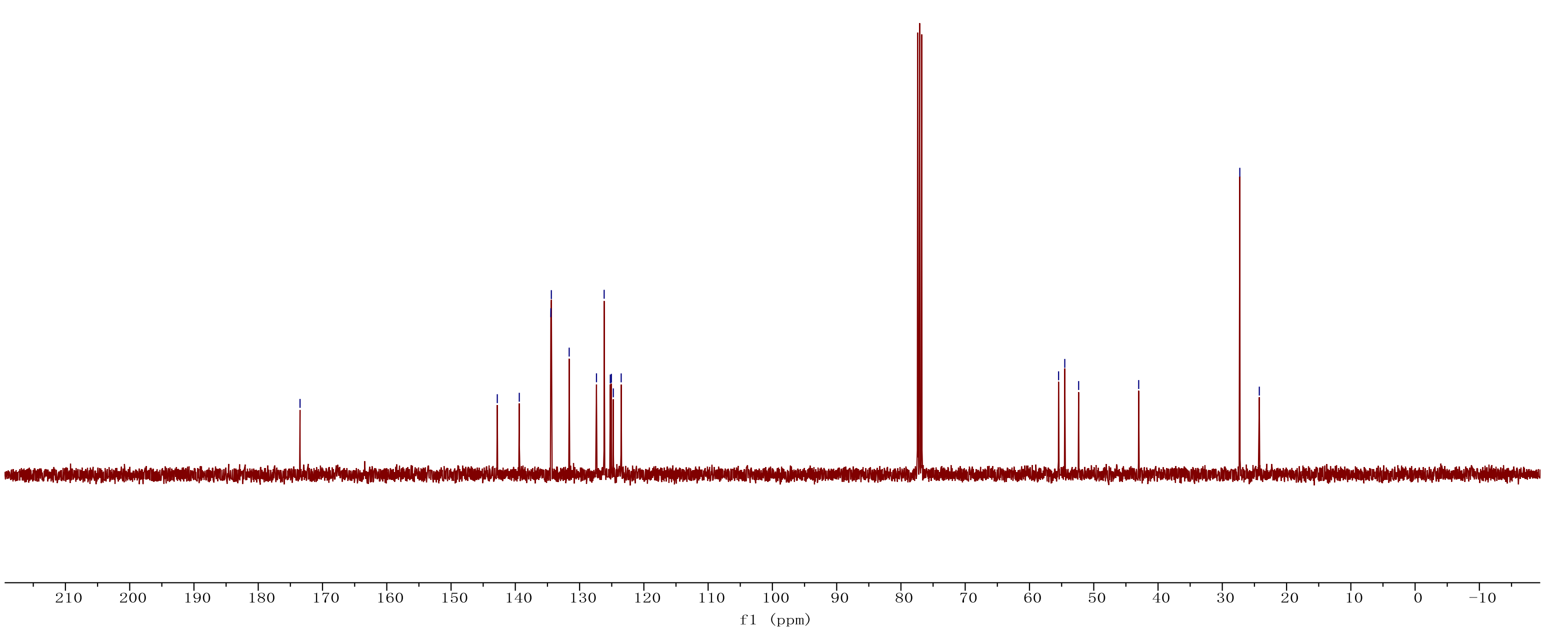

^{1}H NMR (400 MHz, $CDCl_3$) of **2d**

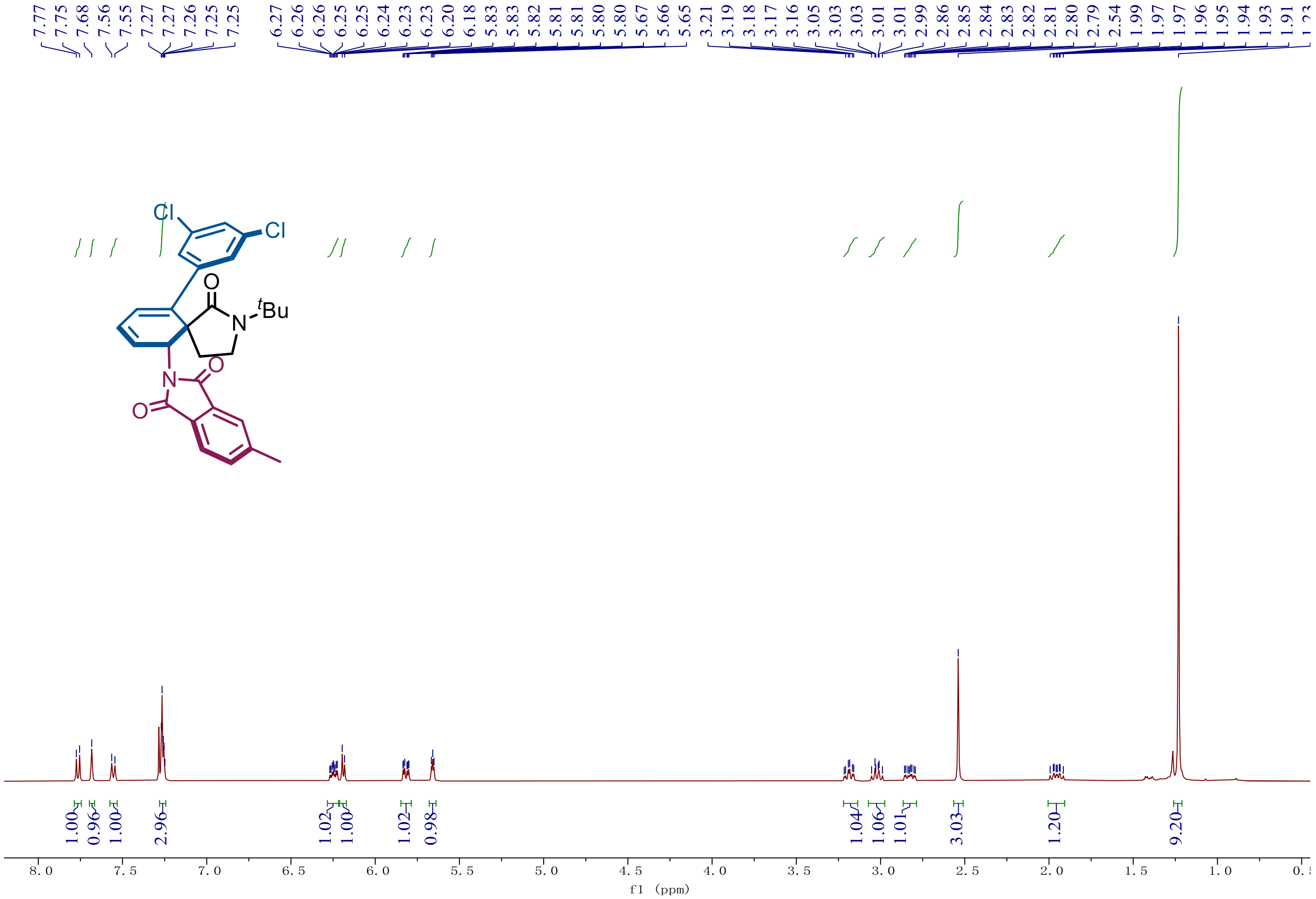

^{13}C NMR (101 MHz, $CDCl_3$) of **2d**

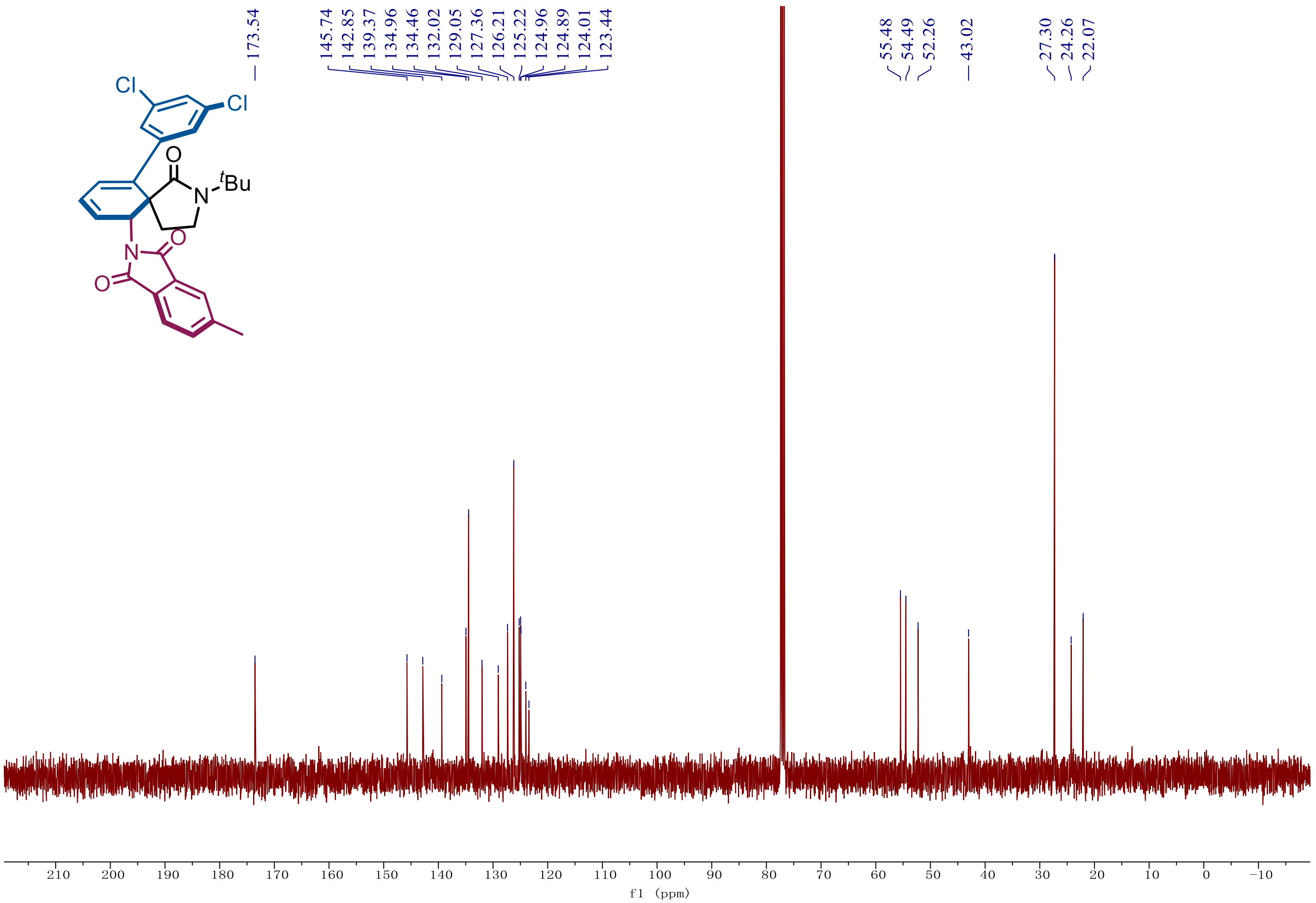

^{1}H NMR (400 MHz, CDCl$_3$) of **2e**

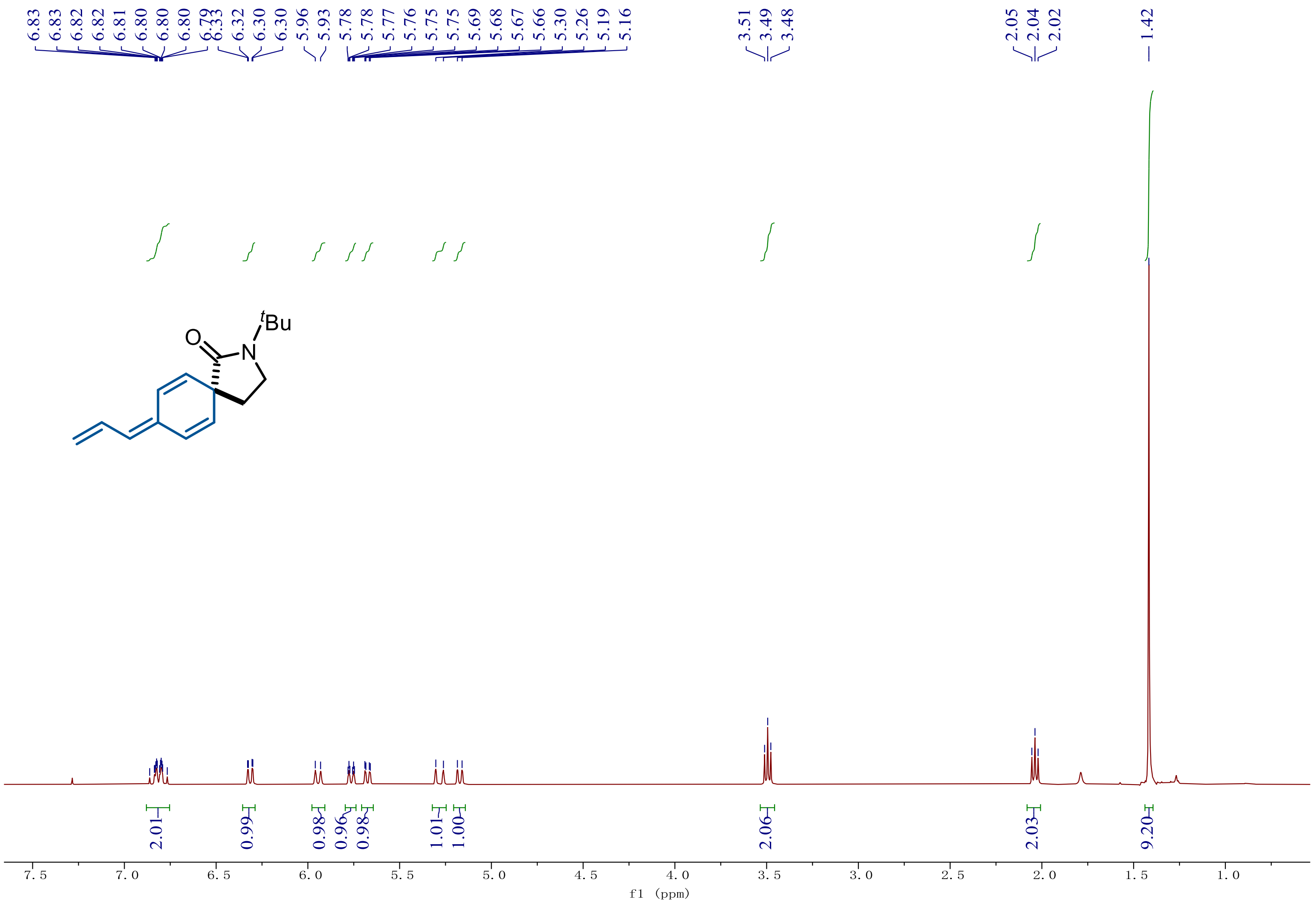

^{13}C NMR (101 MHz, $CDCl_3$) of **2e**

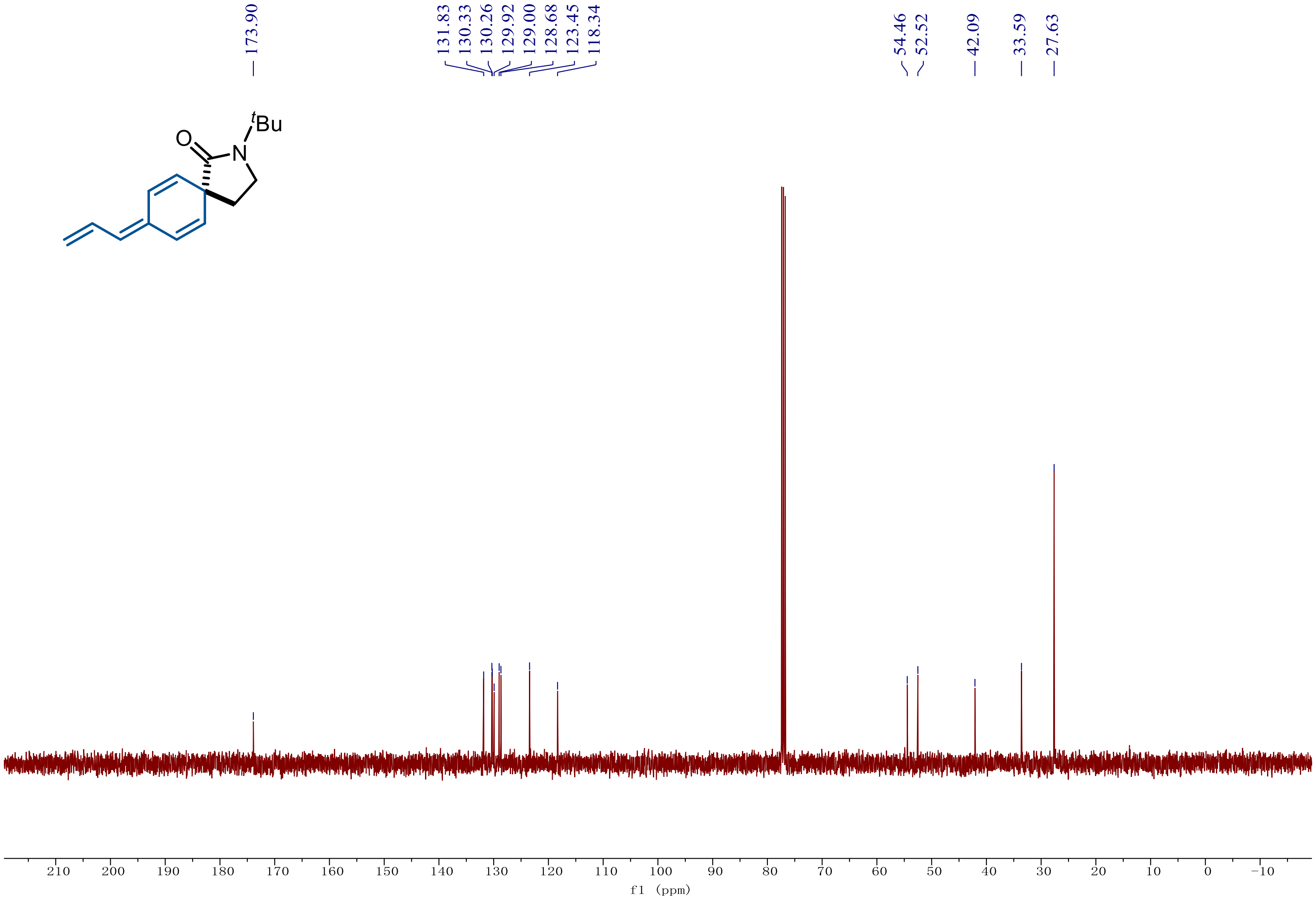

^{1}H NMR (400 MHz, $CDCl_3$) of **2f**

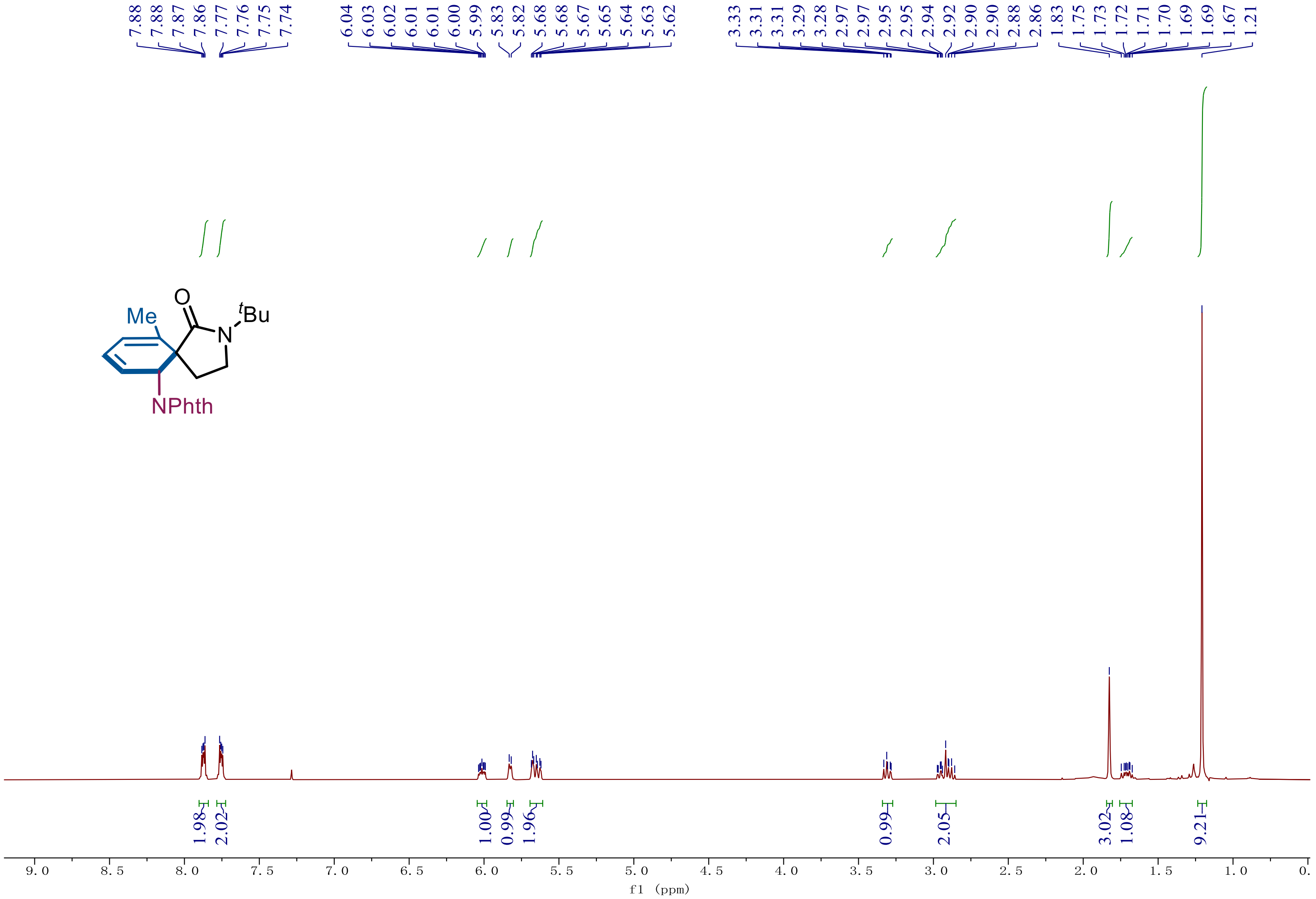

^{13}C NMR (101 MHz, $CDCl_3$) of **2f**

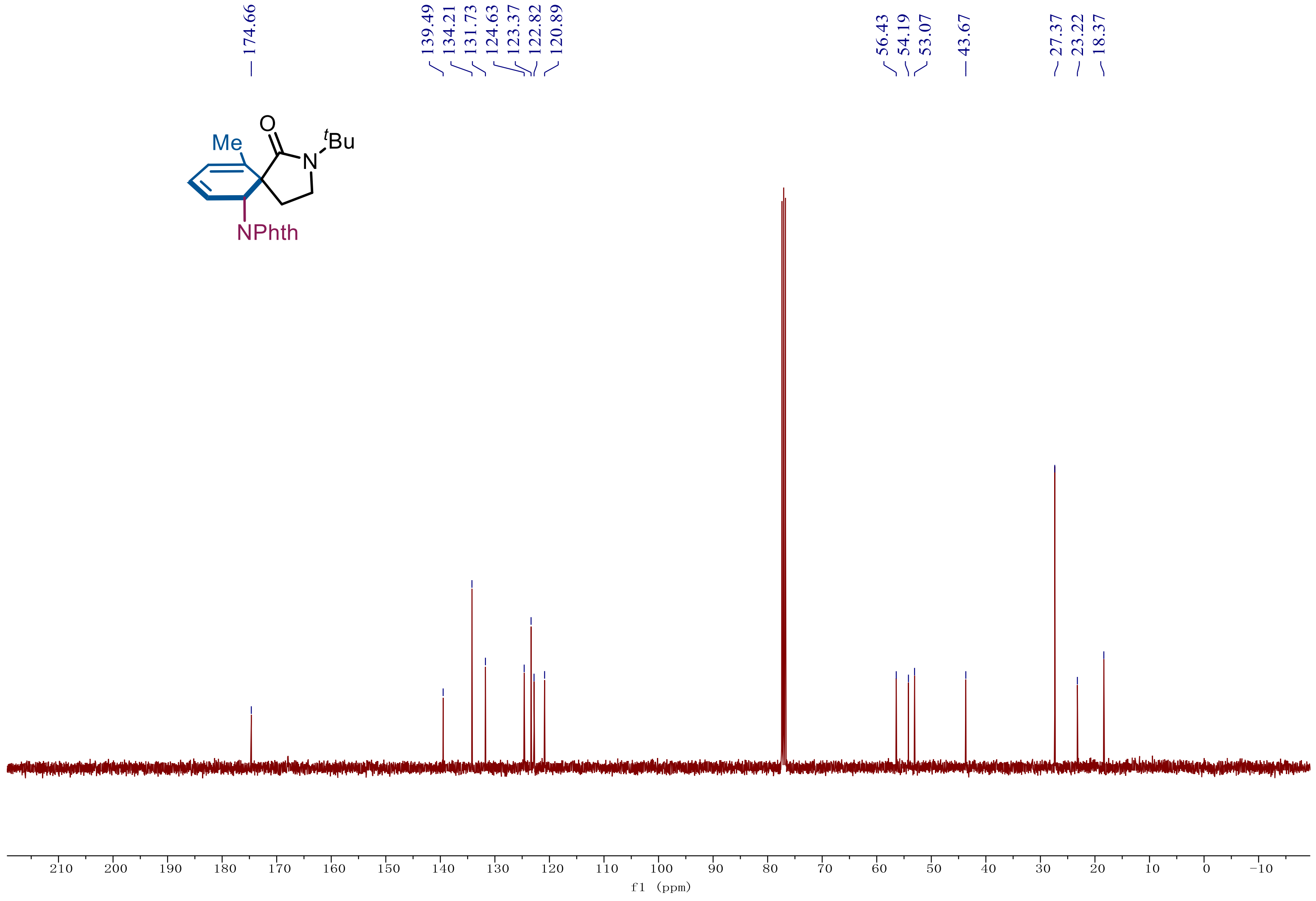

^{1}H NMR (400 MHz, $CDCl_3$) of **2g**

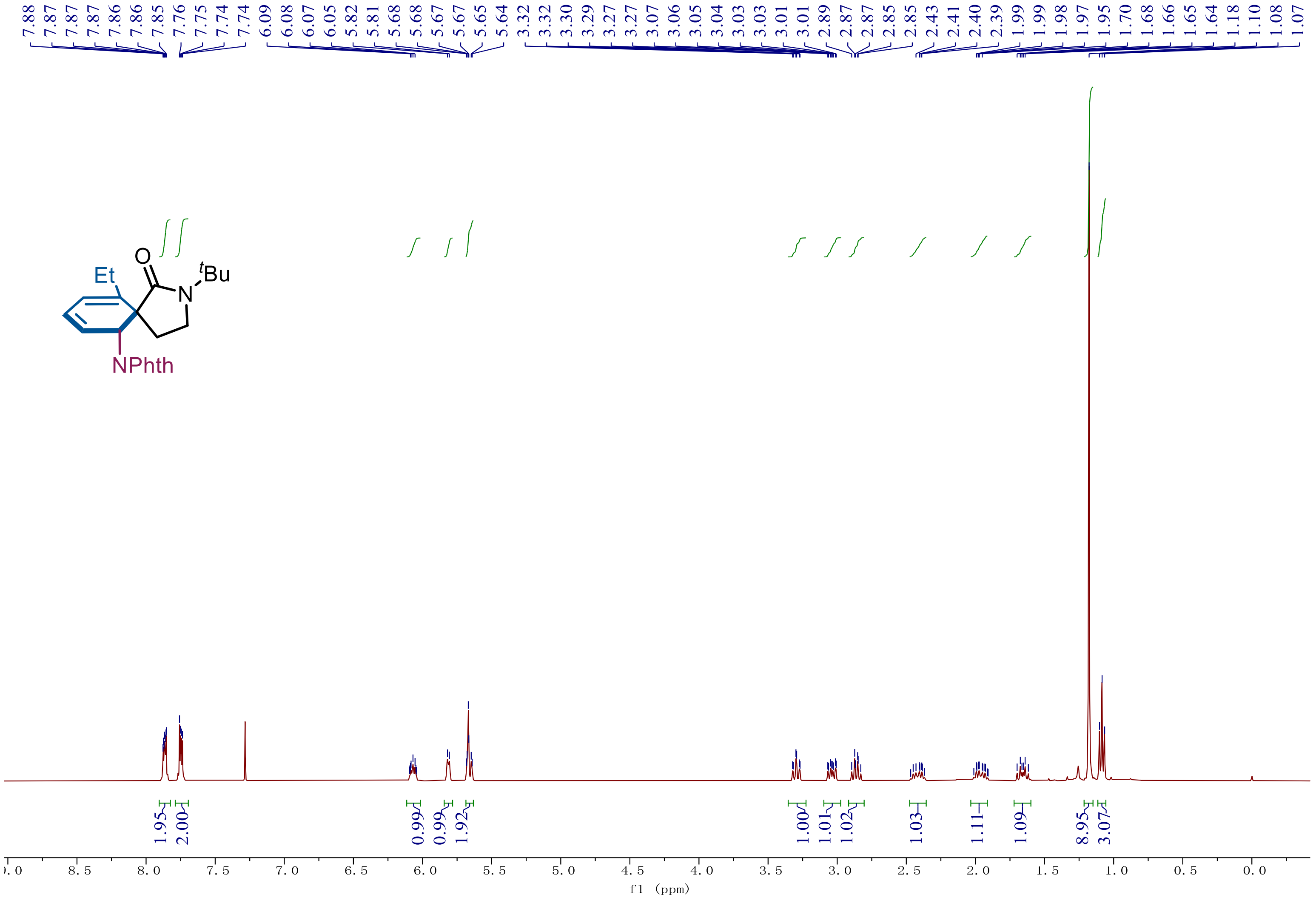

^{13}C NMR (101 MHz, $CDCl_3$) of **2g**

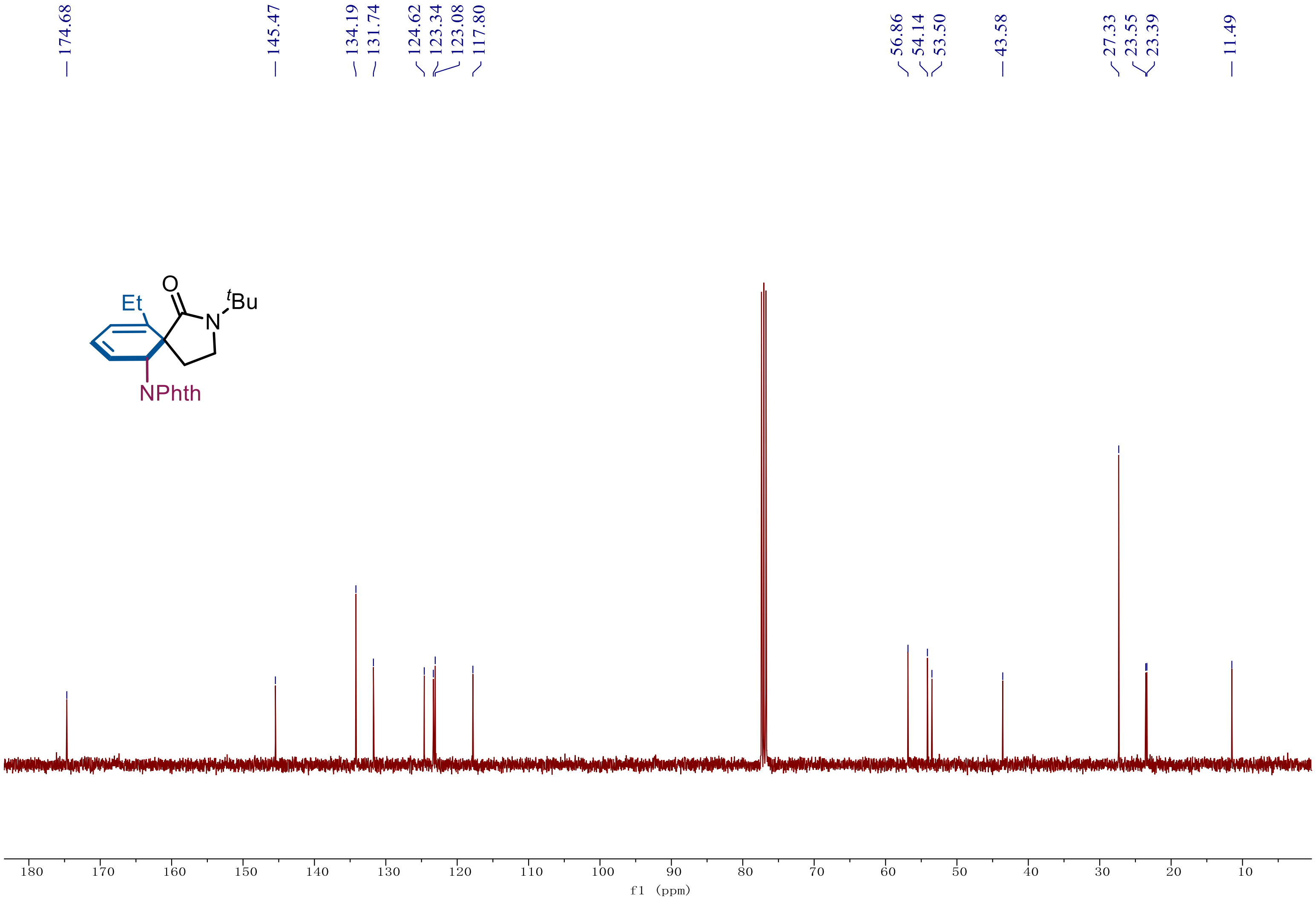

^{1}H NMR (400 MHz, $CDCl_3$) of **2h**

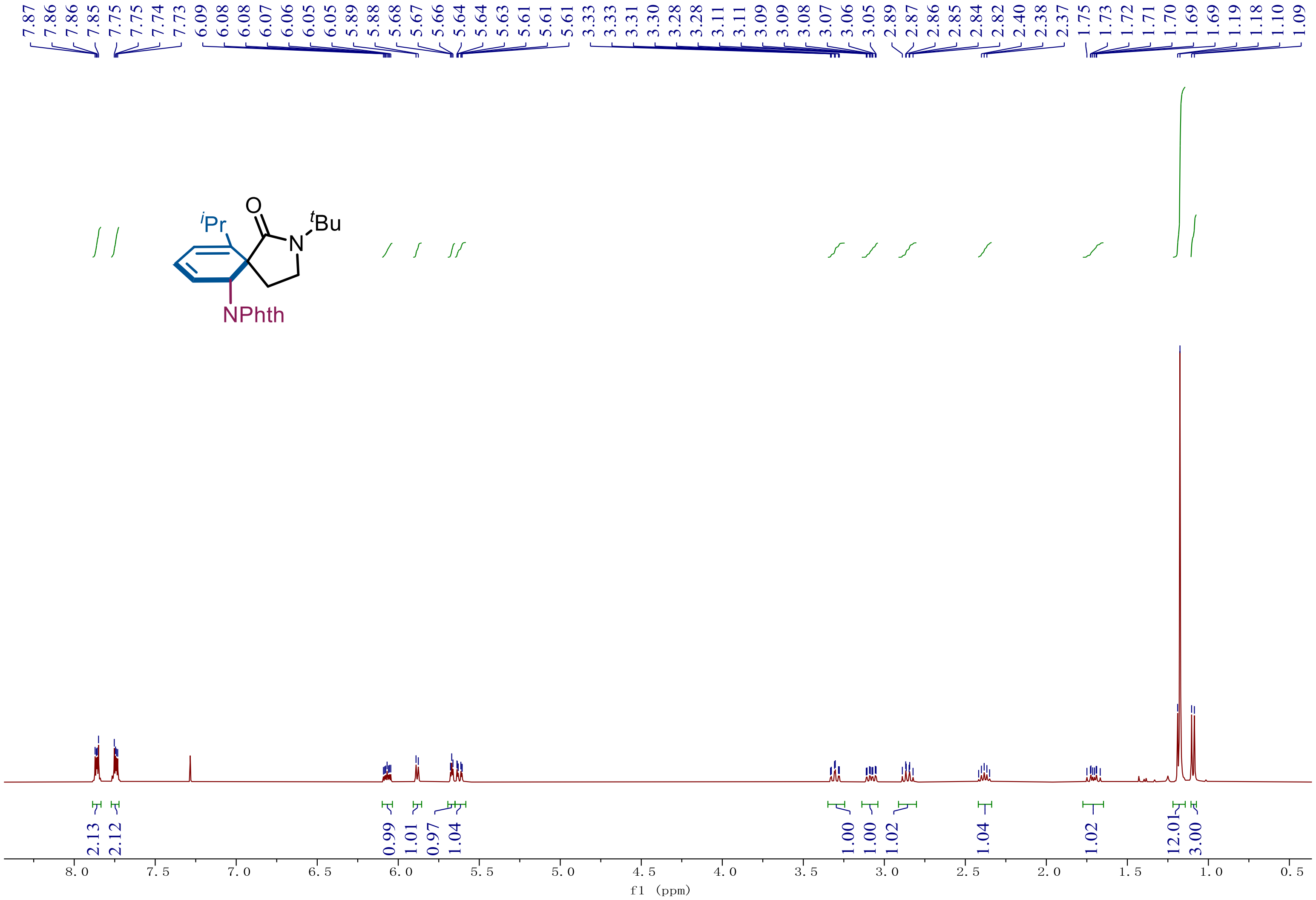

^{13}C NMR (101 MHz, $CDCl_3$) of **2h**

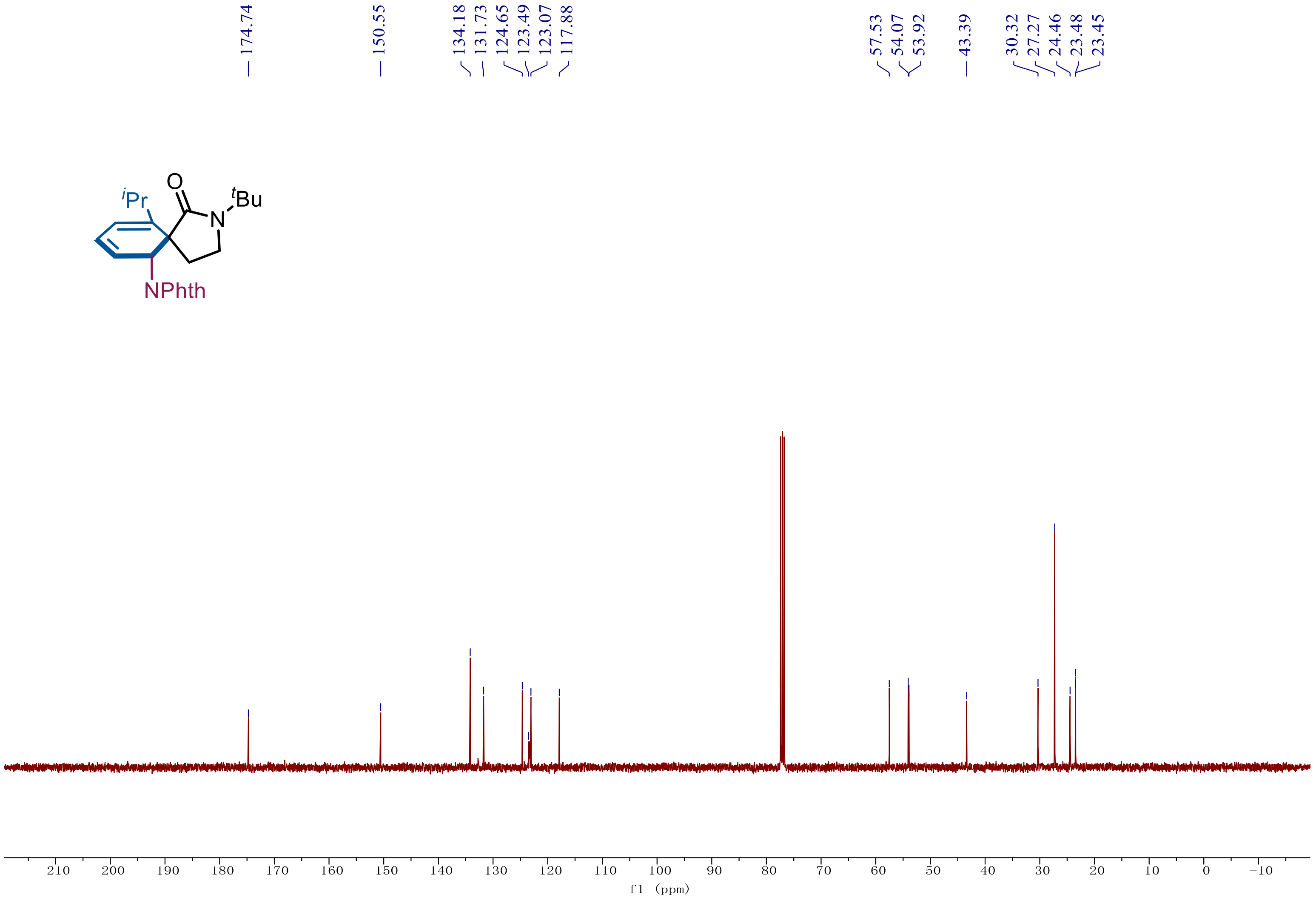

^{1}H NMR (400 MHz, $CDCl_3$) of **2i**

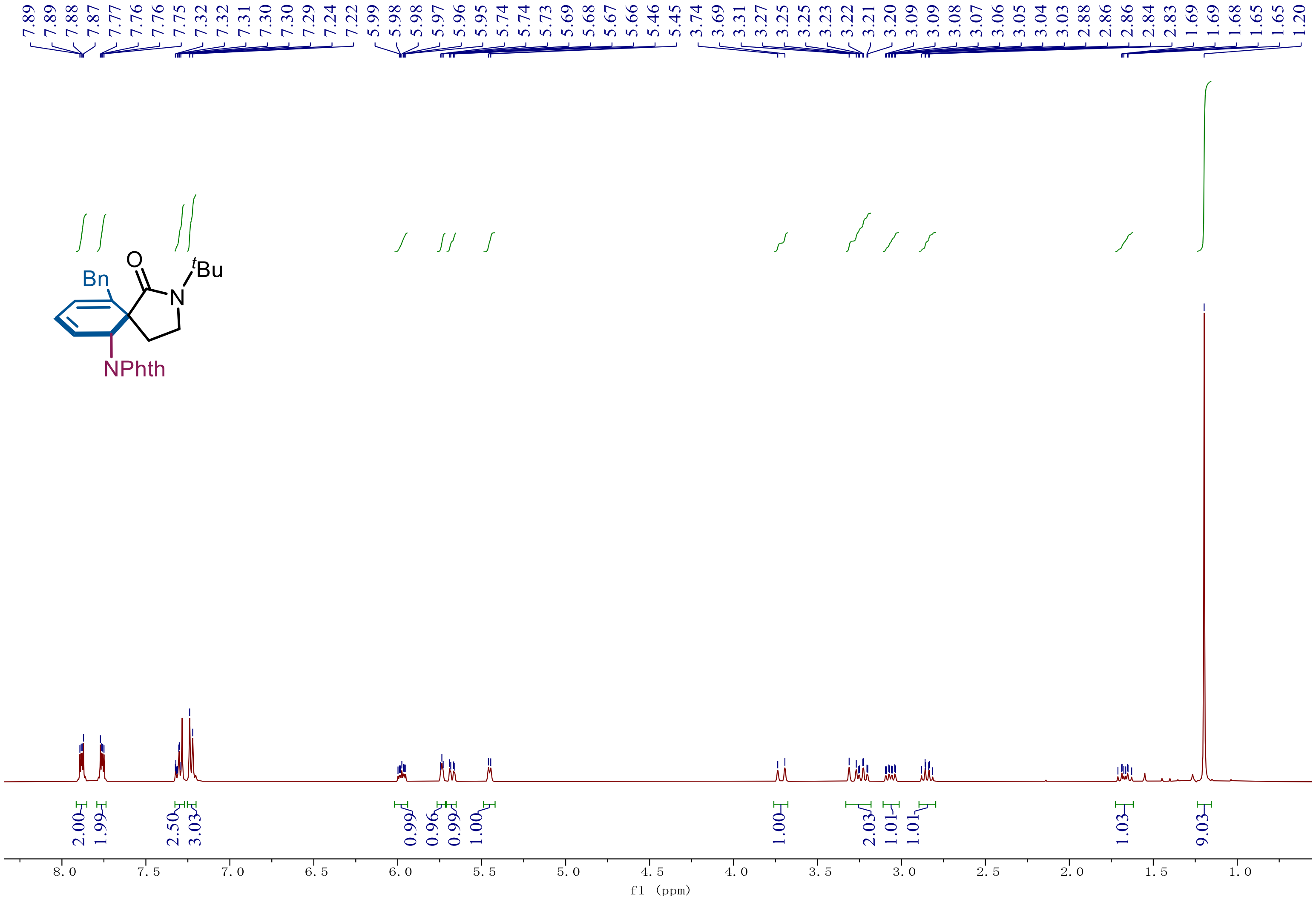

^{13}C NMR (101 MHz, $CDCl_3$) of **2i**

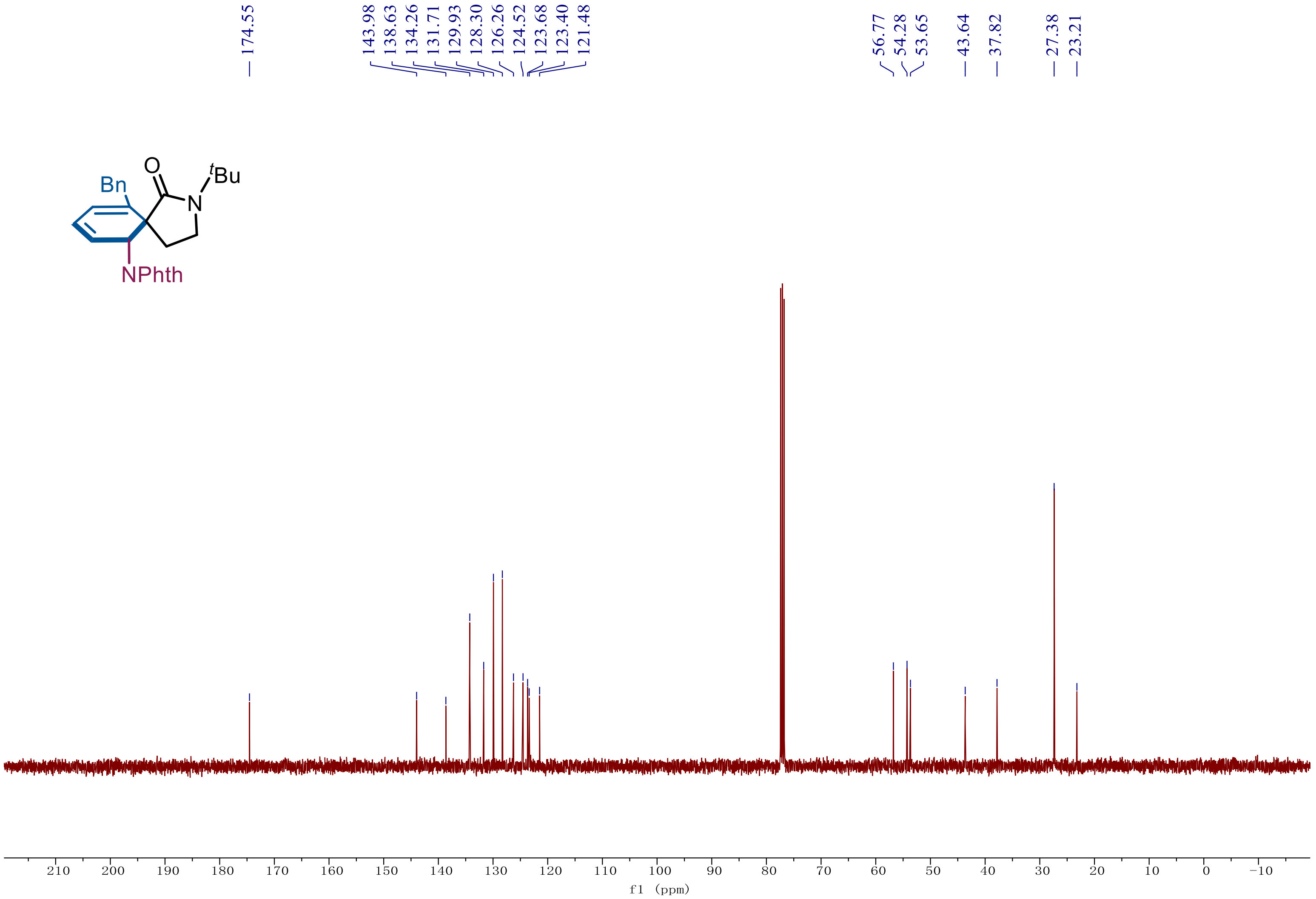

^{1}H NMR (500 MHz, $CDCl_3$) of **2j**

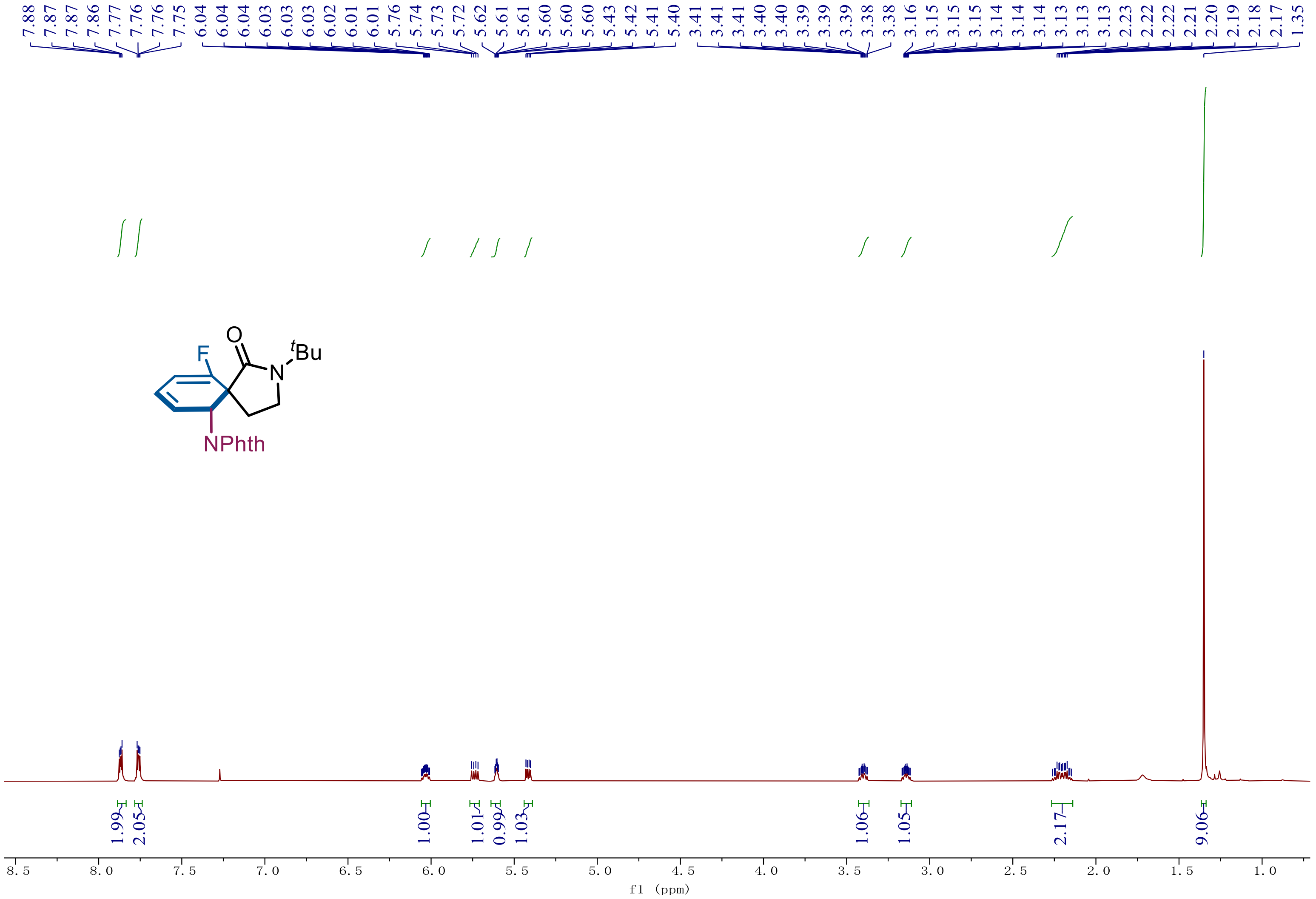

^{13}C NMR (126 MHz, $CDCl_3$) of **2j**

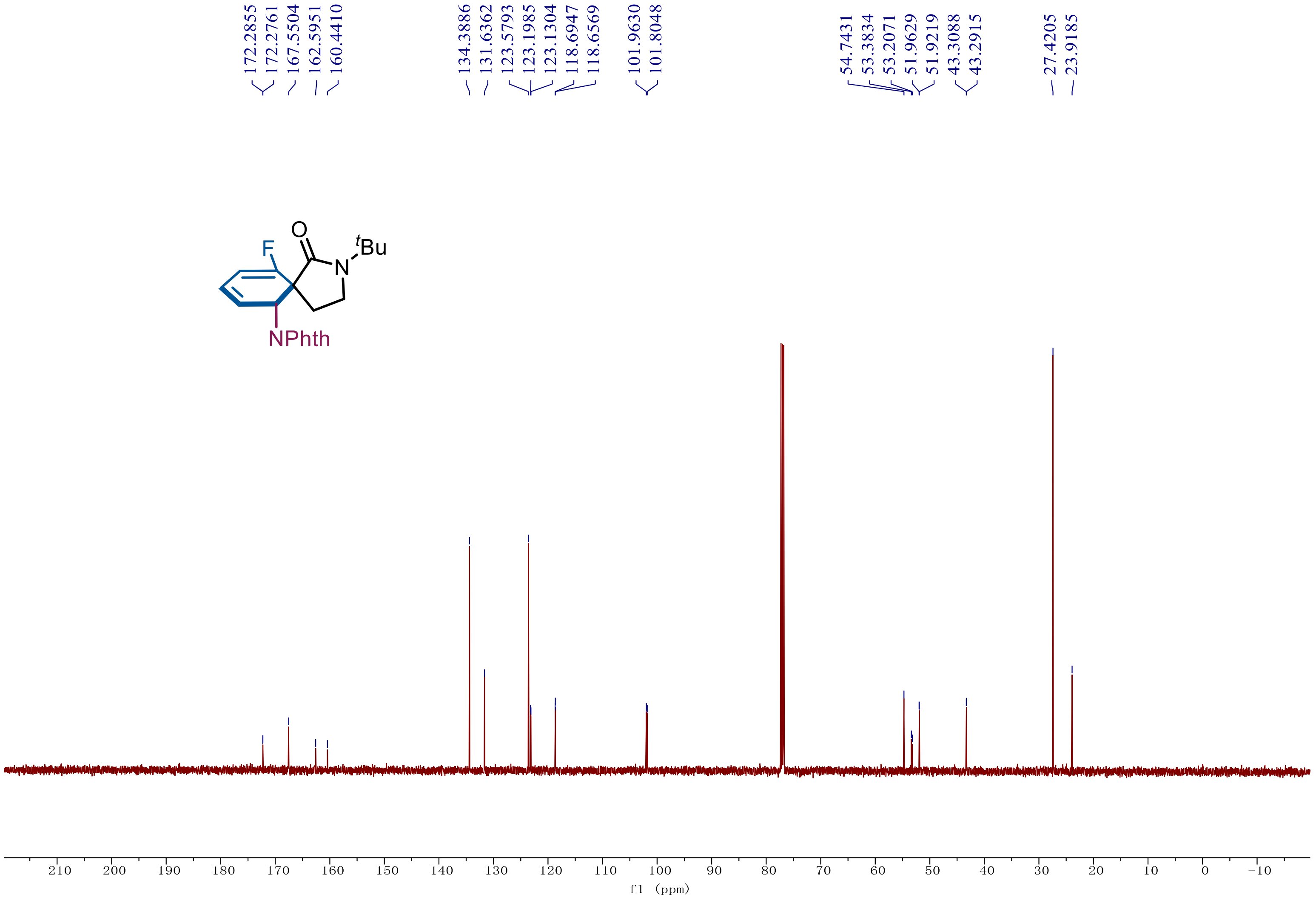

^{19}F NMR (471 MHz, $CDCl_3$) of **2j**

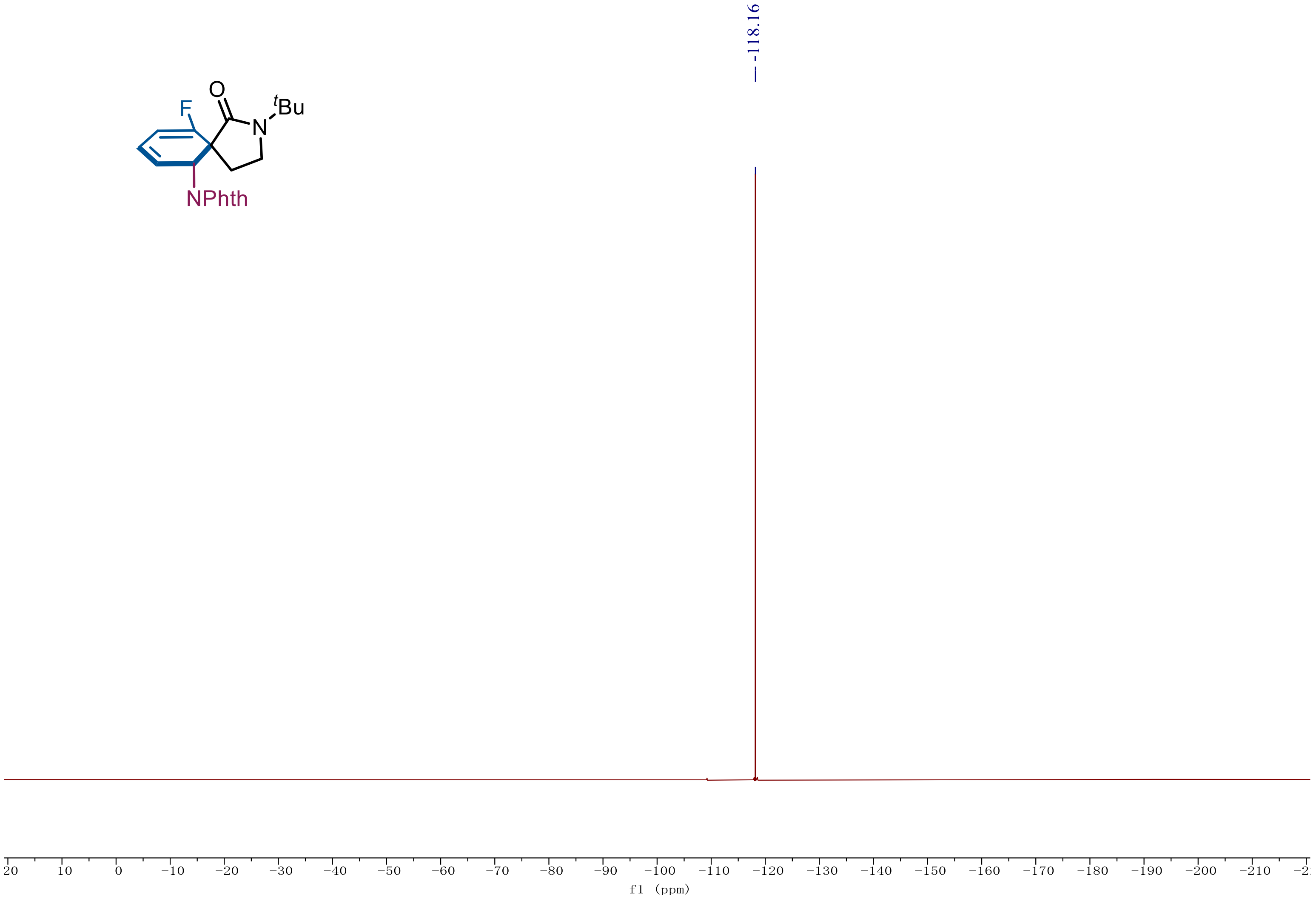

^{1}H NMR (500 MHz, $CDCl_3$) of **2k**

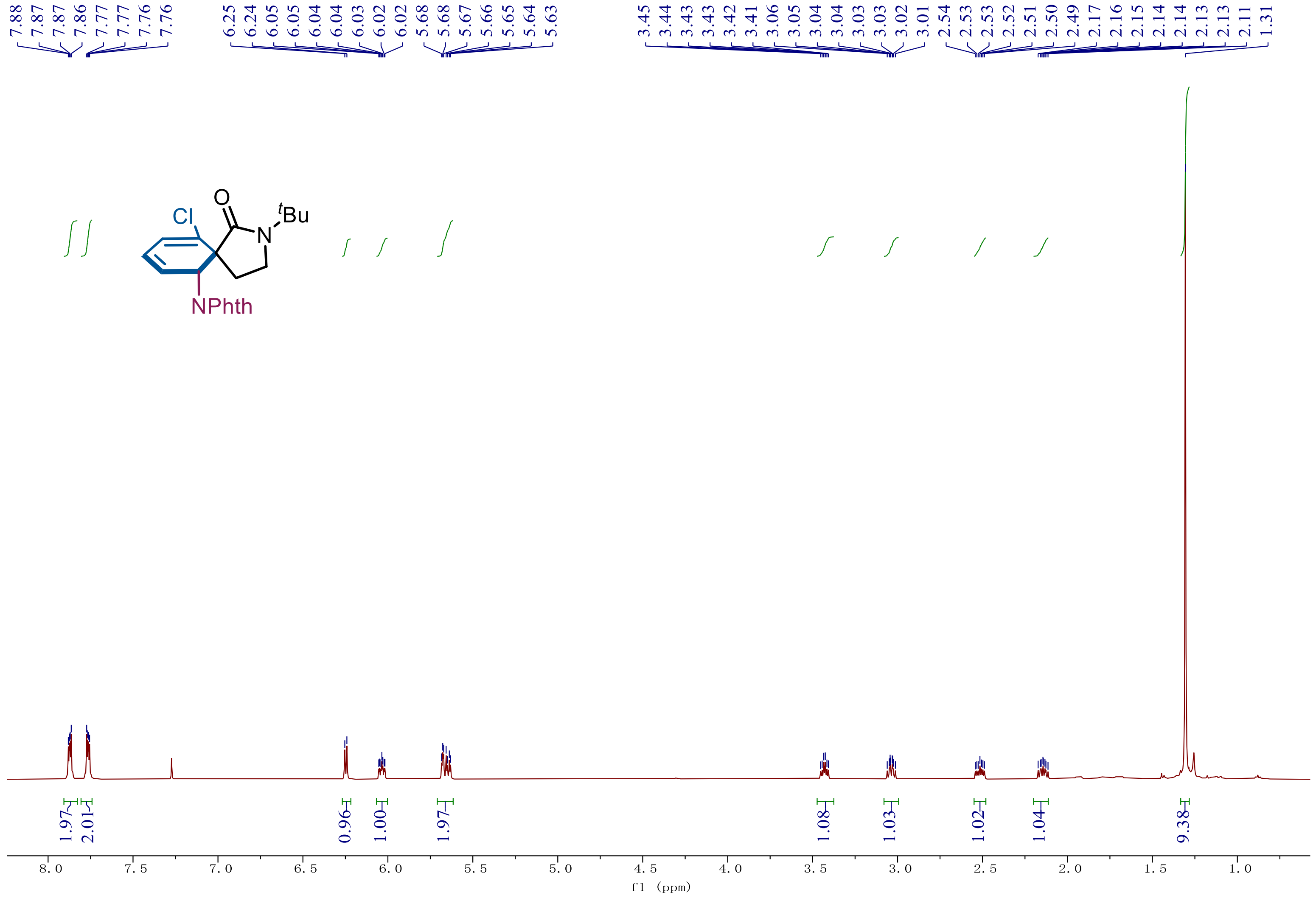

^{13}C NMR (126 MHz, $CDCl_3$) of **2k**

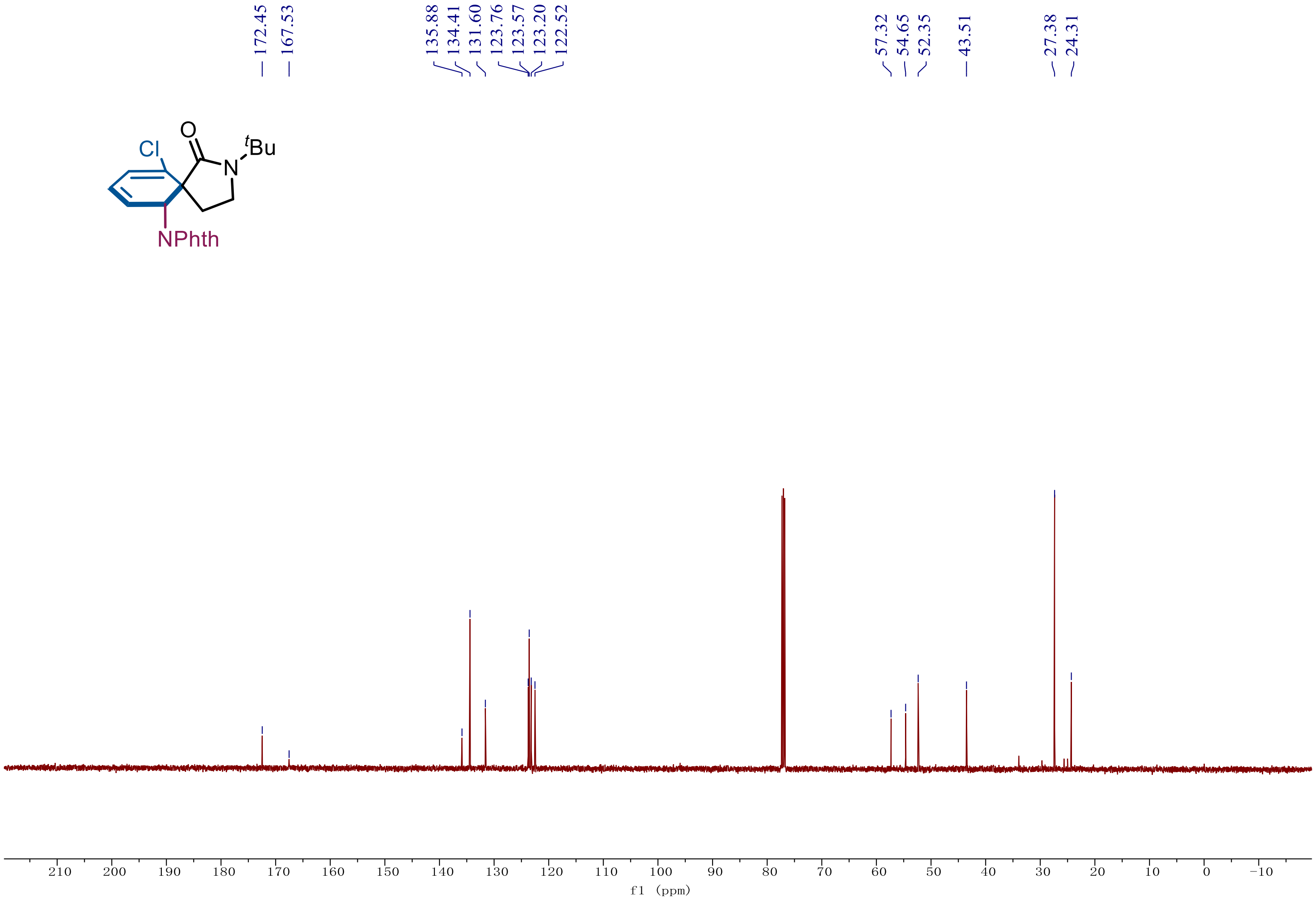

^{1}H NMR (400 MHz, $CDCl_3$) of **2l**

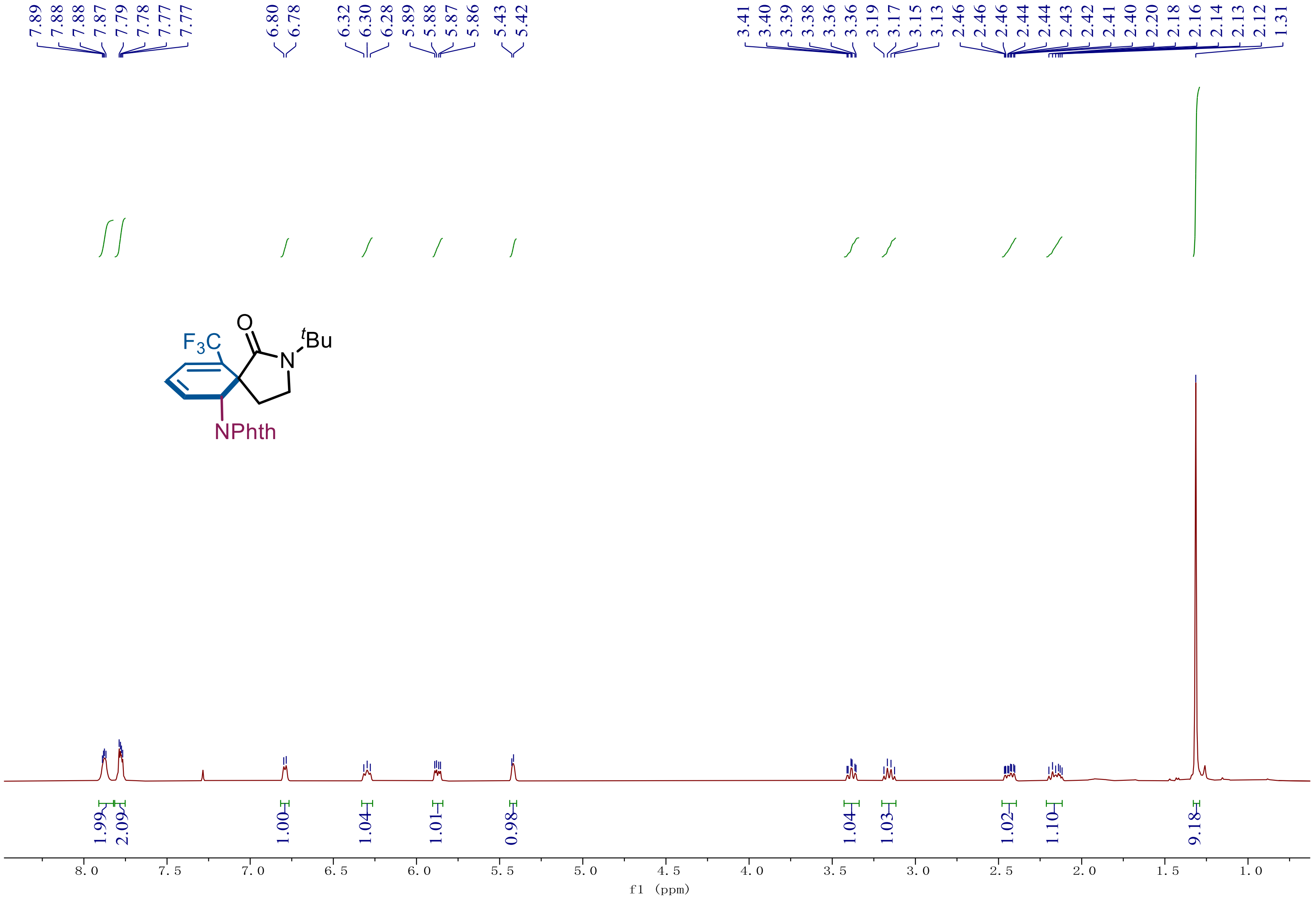

^{13}C NMR (126 MHz, CDCl3) of **2l**

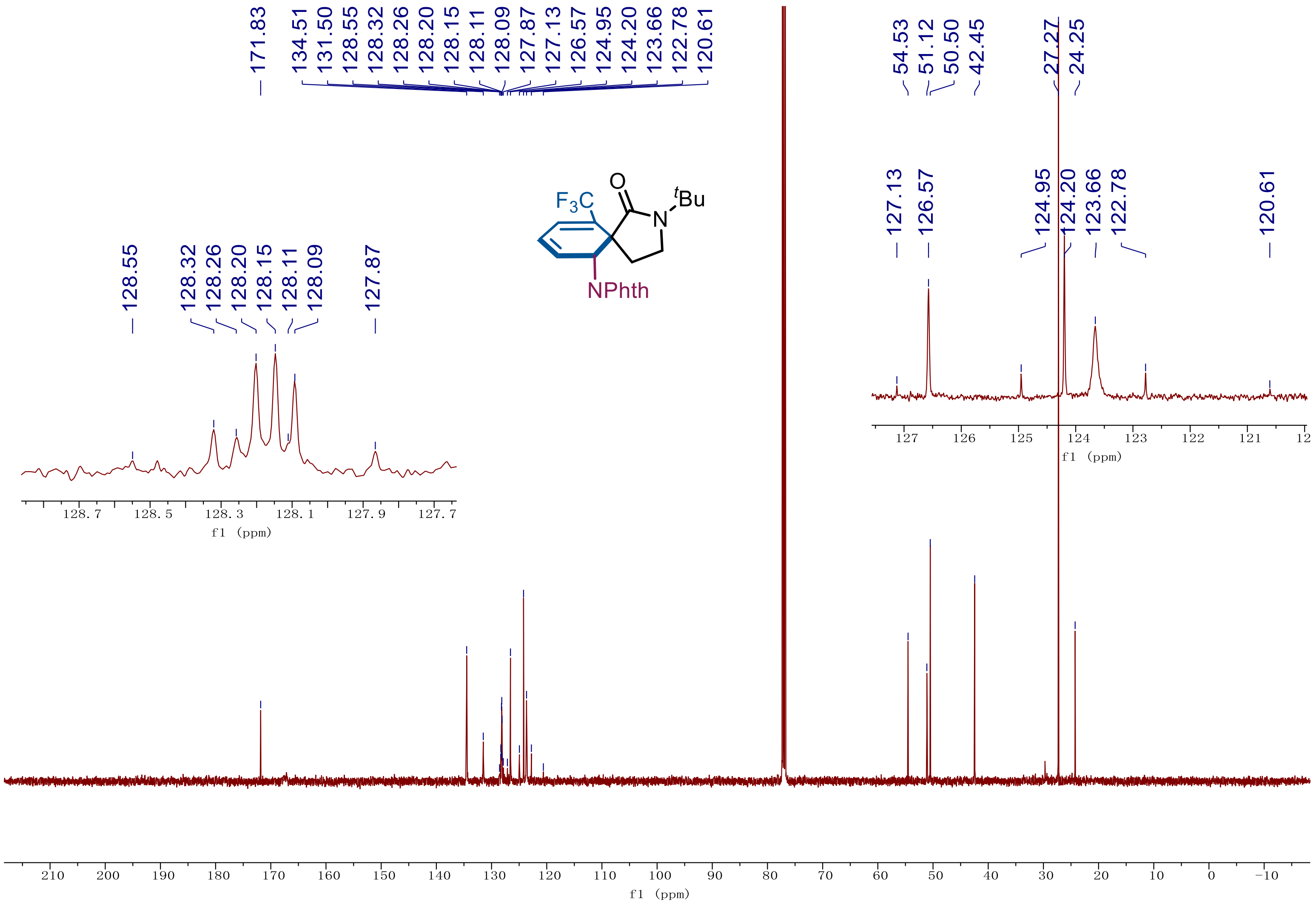

^{19}F NMR (471 MHz, $CDCl_3$) of **2l**

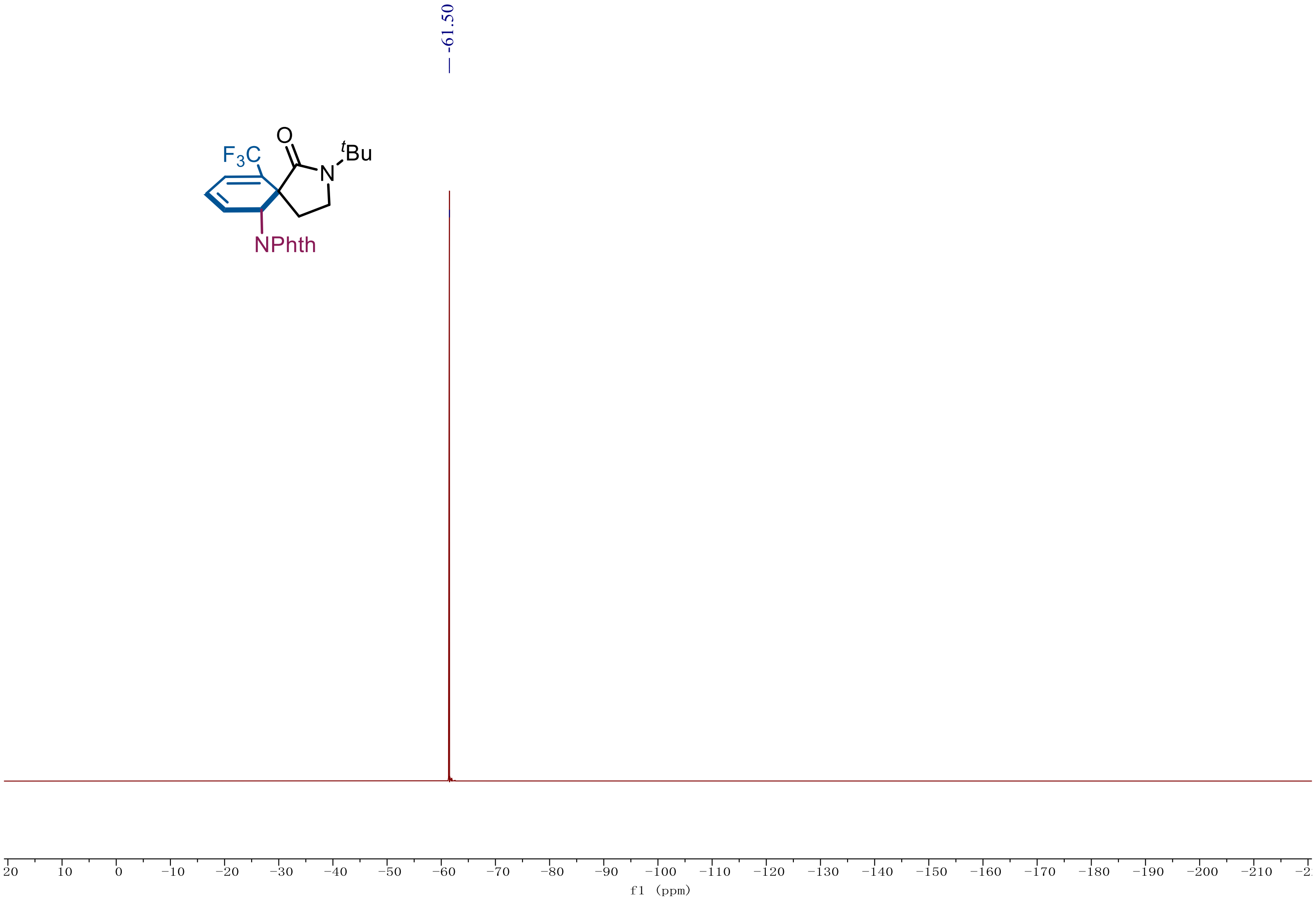

^{1}H NMR (400 MHz, $CDCl_3$) of **2m**

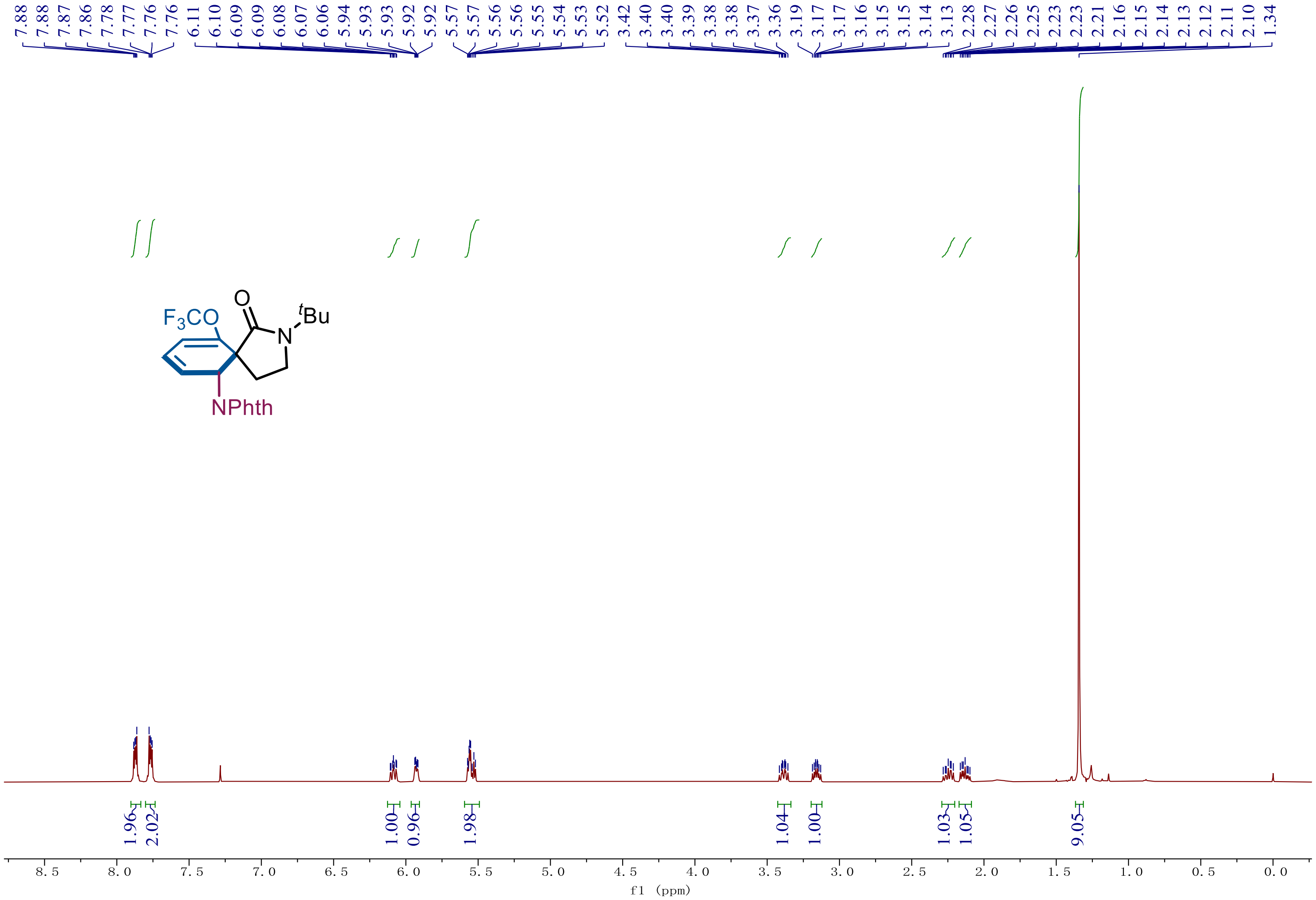

^{13}C NMR (126 MHz, $CDCl_3$) of **2m**

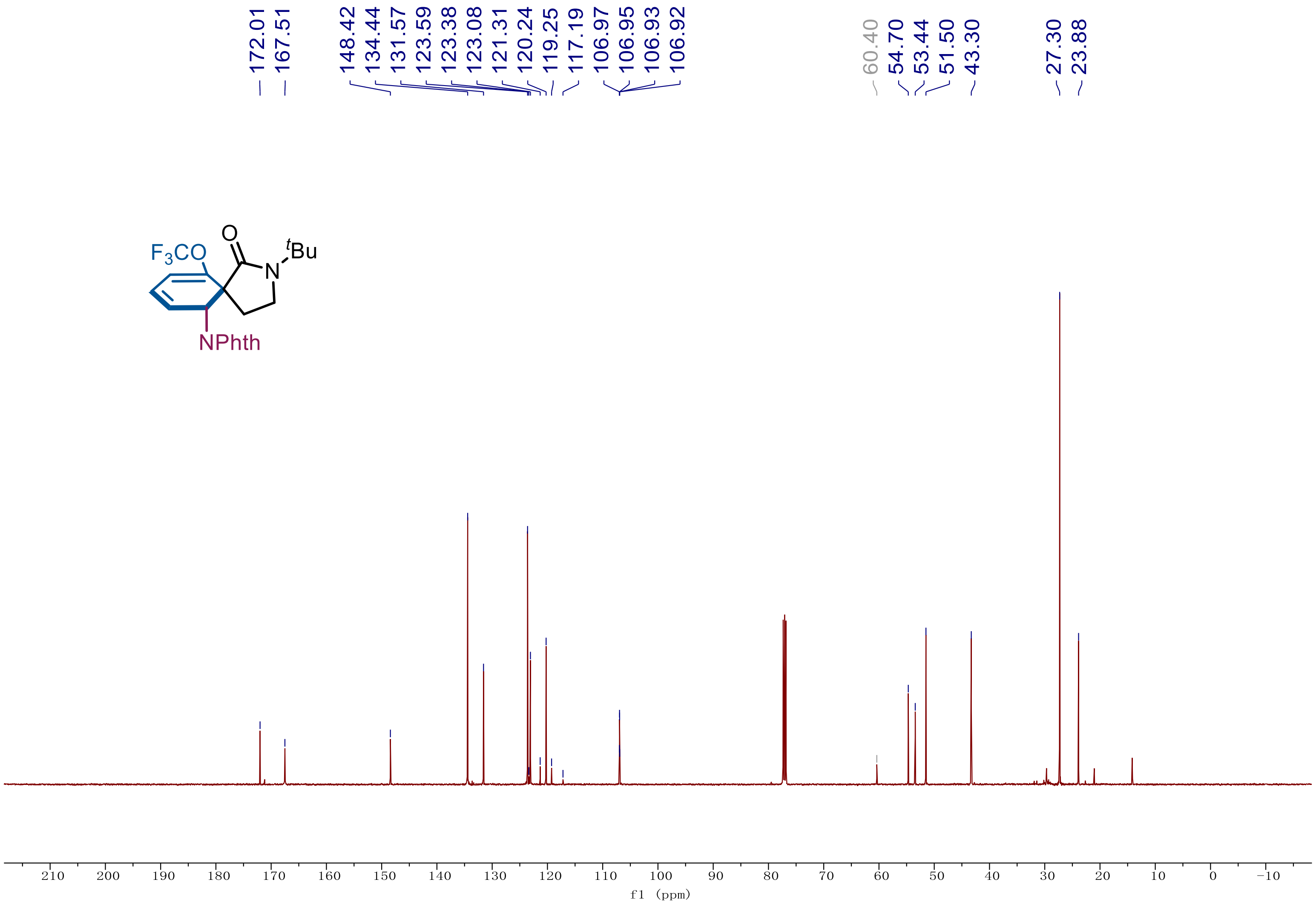

^{19}F NMR (376 MHz, $CDCl_3$) of **2m**

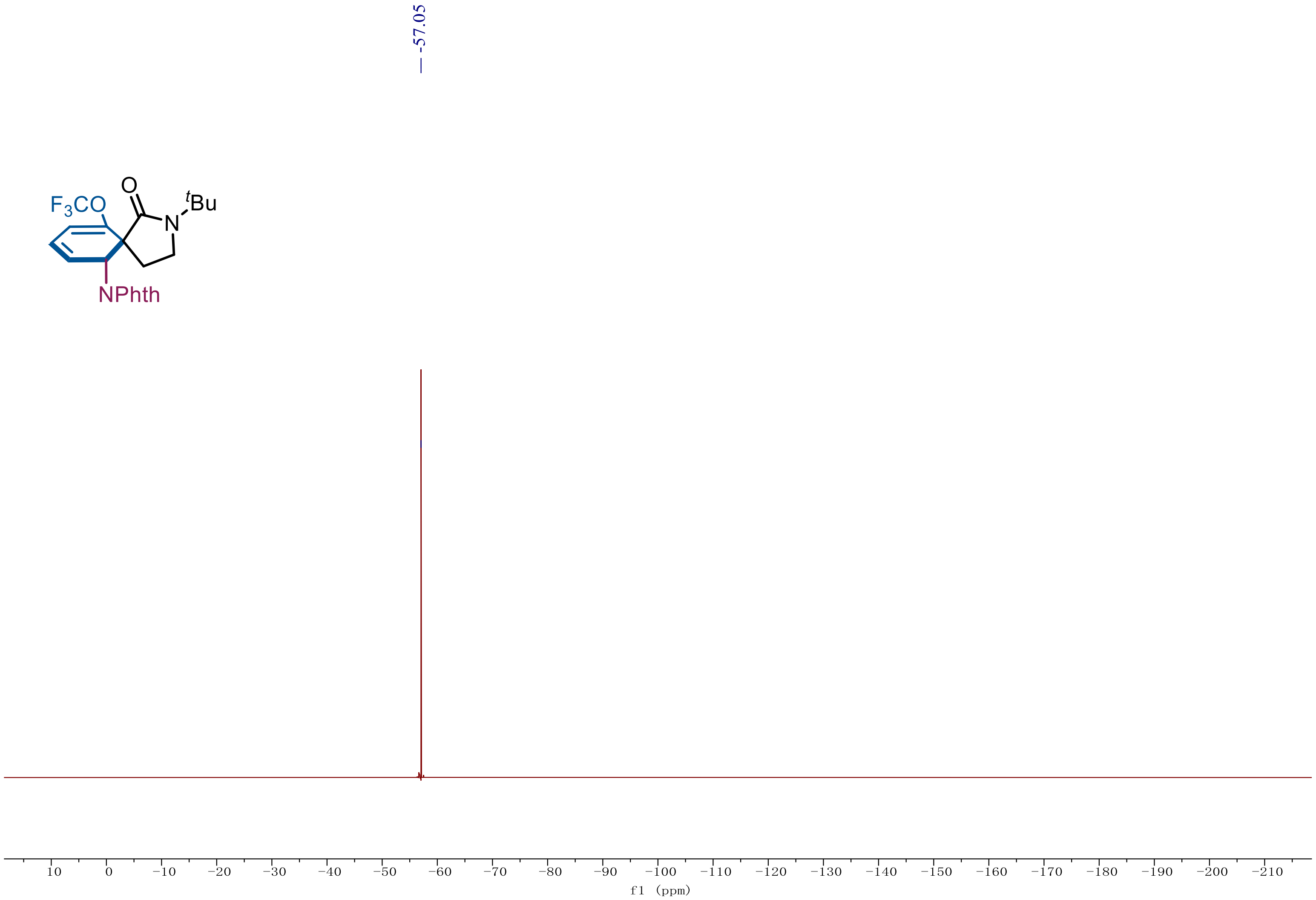

^{1}H NMR (400 MHz, CDCl$_3$) of **2n**

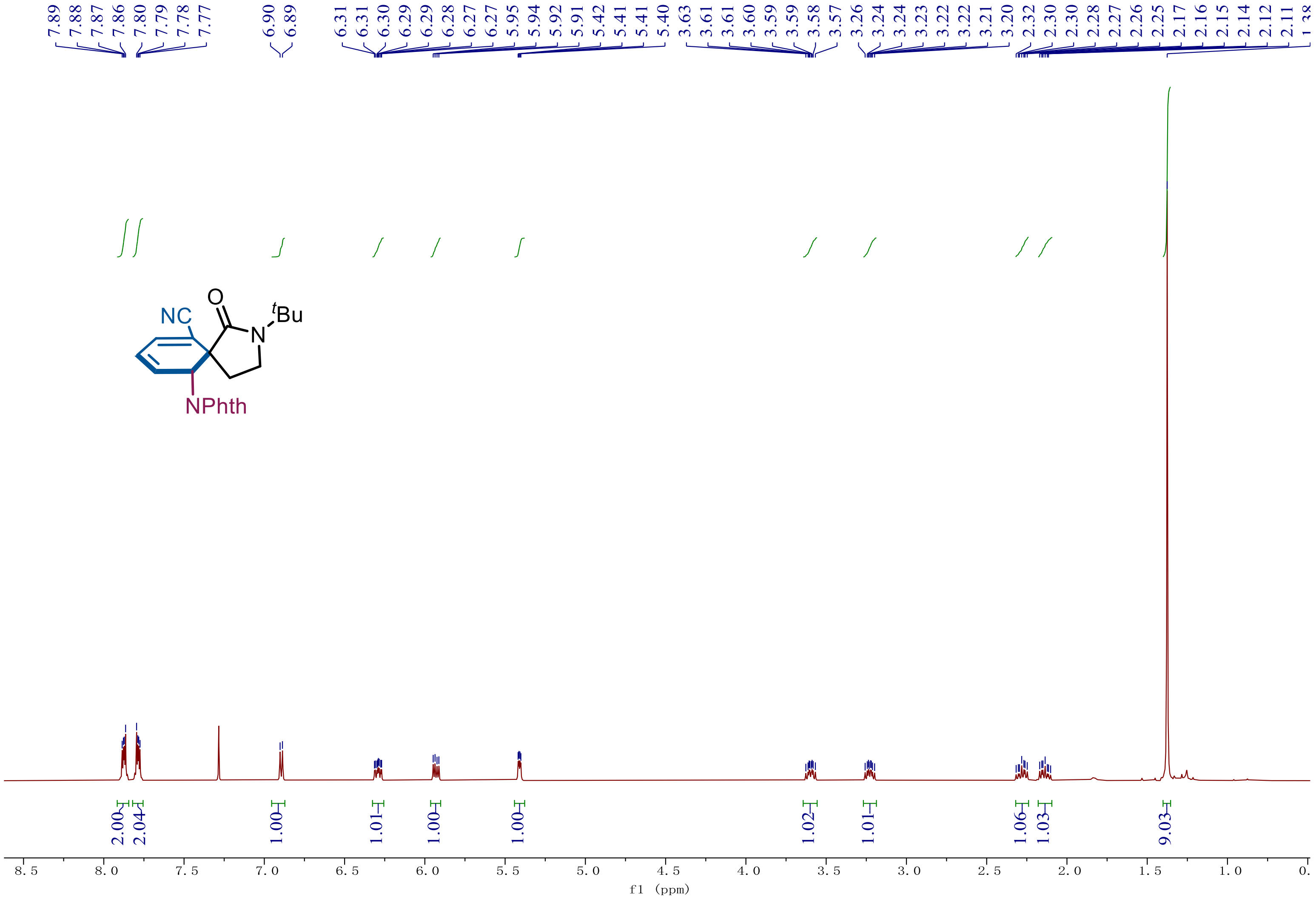

^{13}C NMR (101 MHz, CDCl$_3$) of **2n**

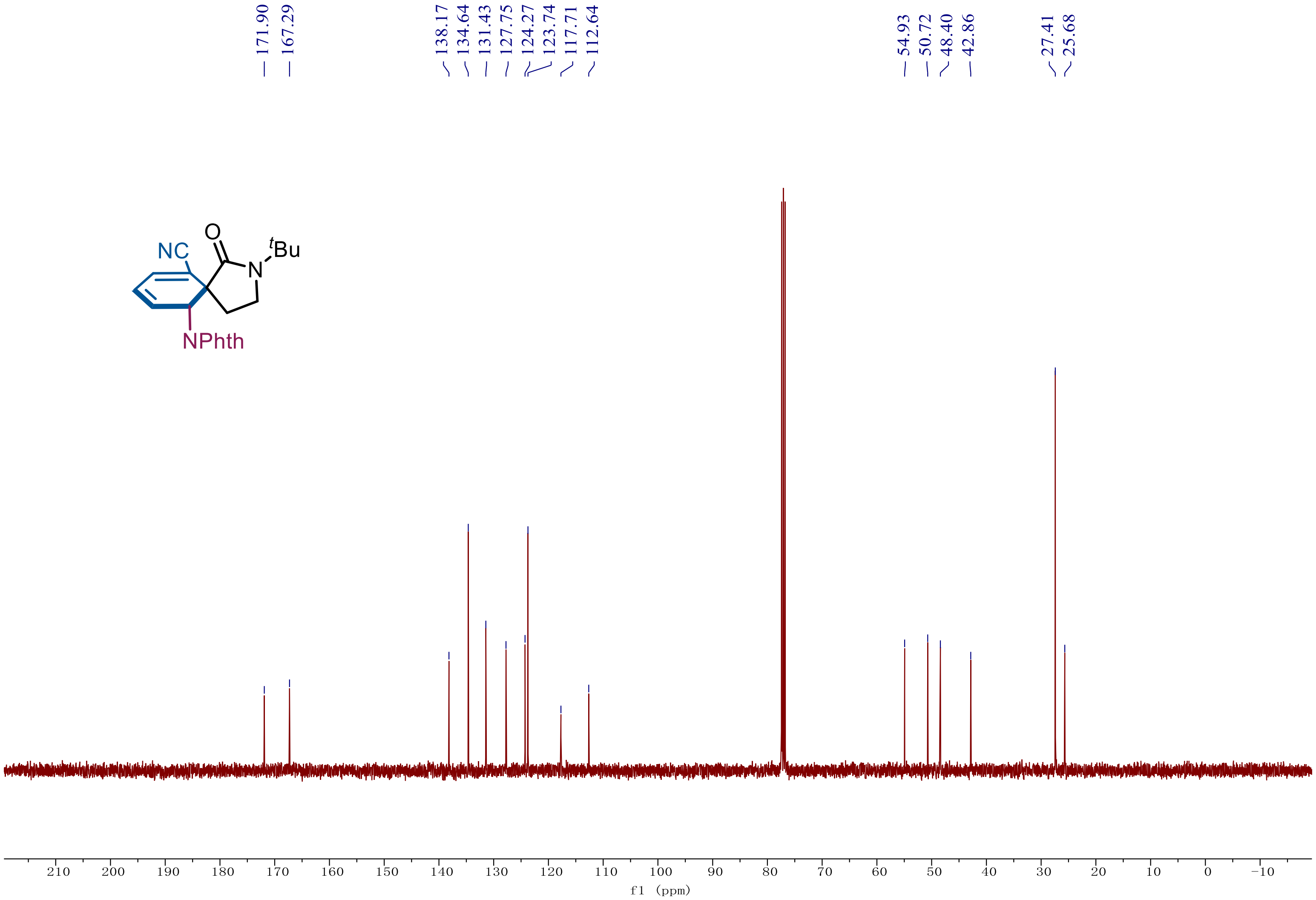

^{1}H NMR (400 MHz, $CDCl_3$) of **2o**

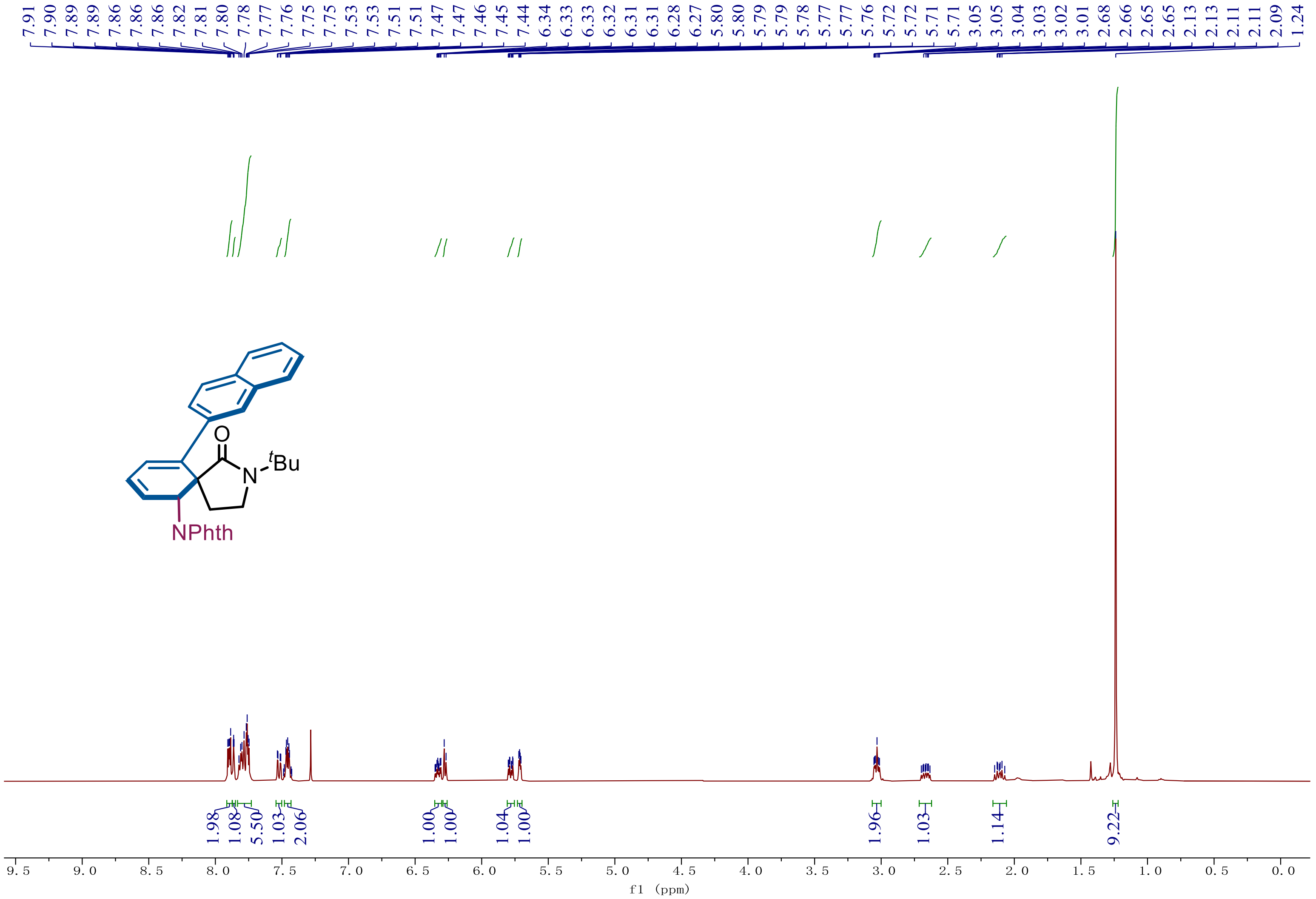

^{13}C NMR (101 MHz, $CDCl_3$) of **2o**

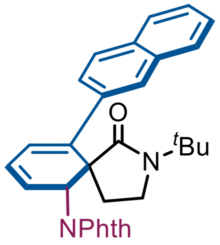

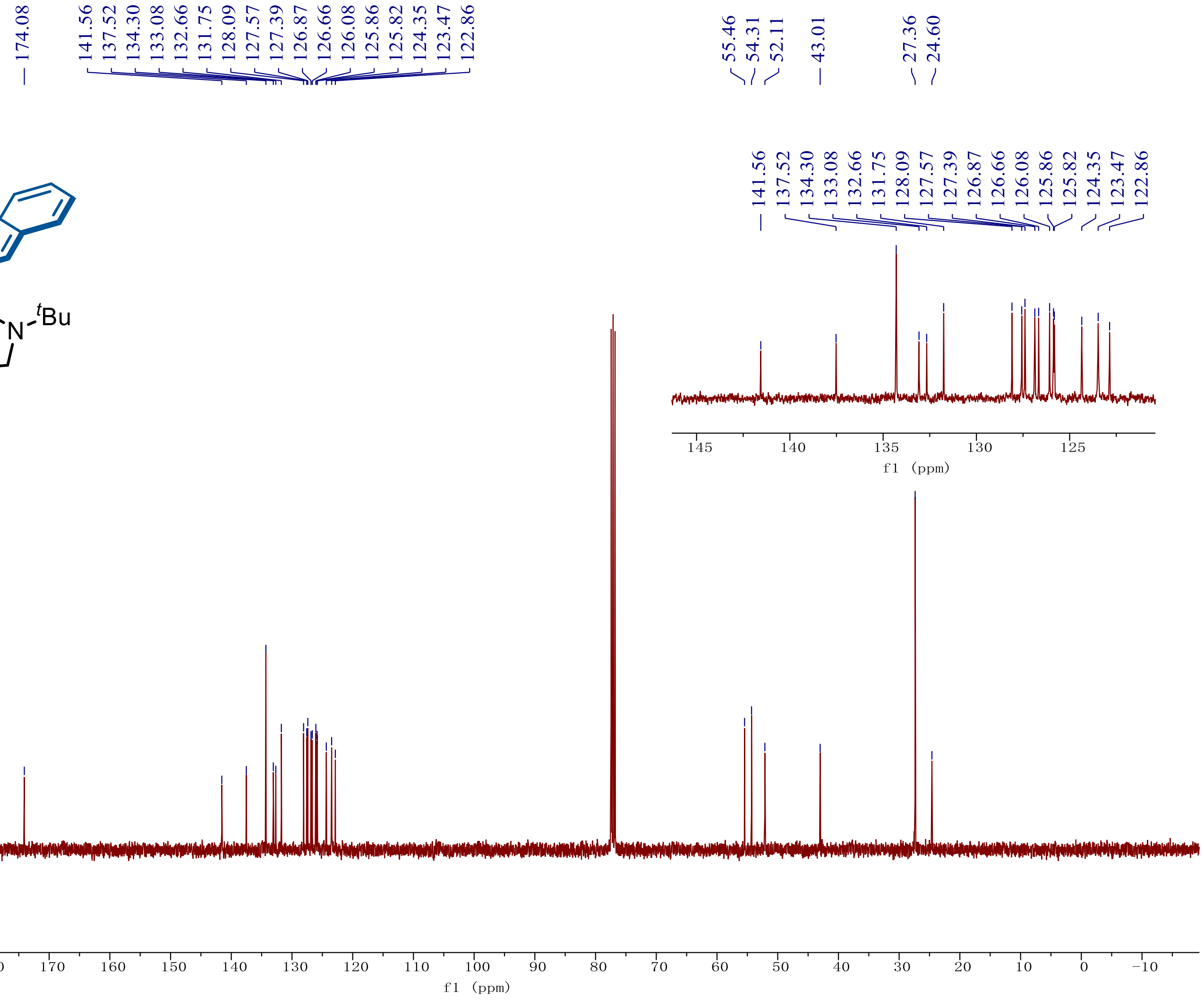

^{1}H NMR (400 MHz, $CDCl_3$) of **2p**

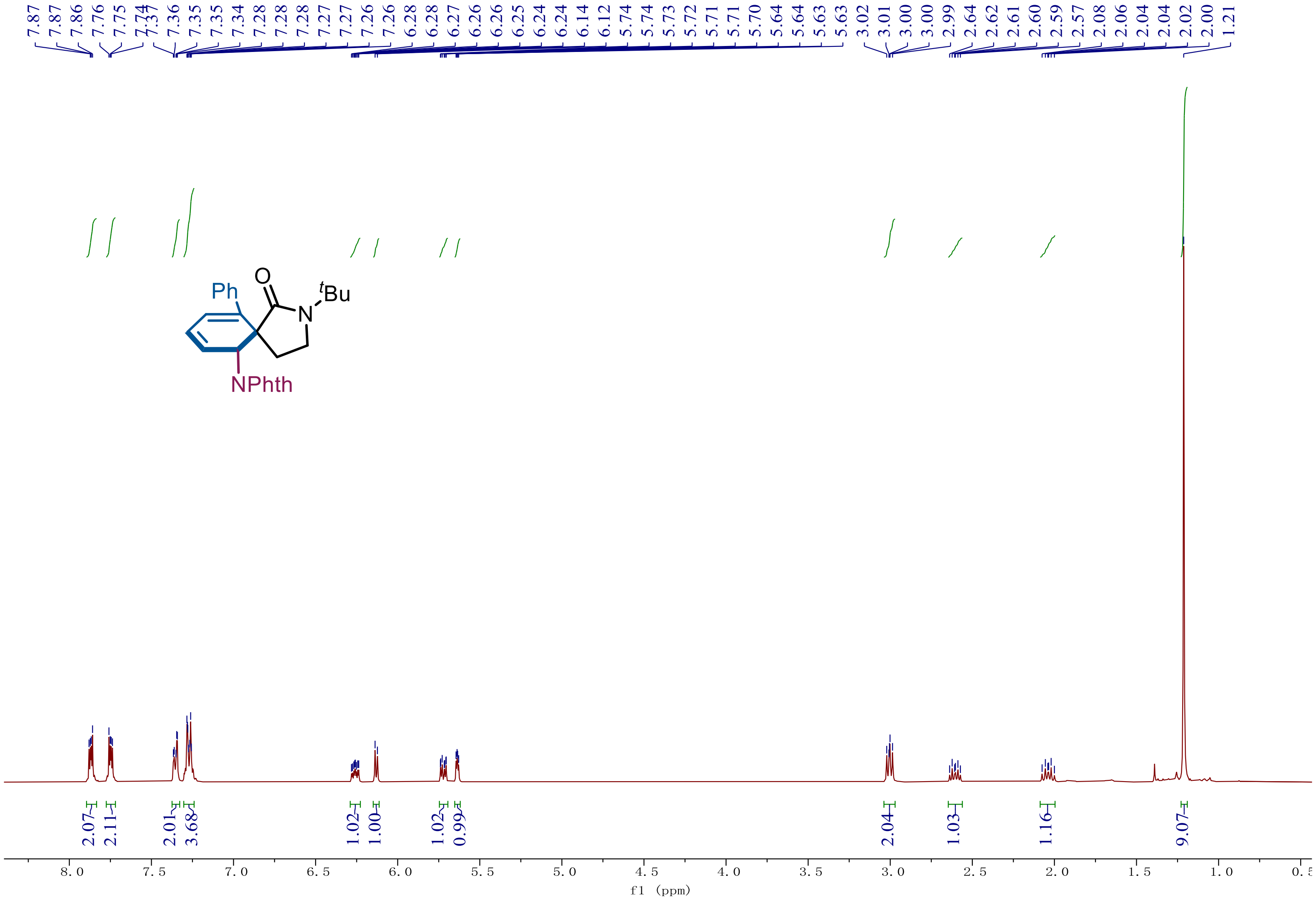

^{13}C NMR (101 MHz, CDCl$_3$) of **2p**

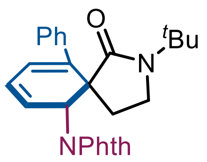

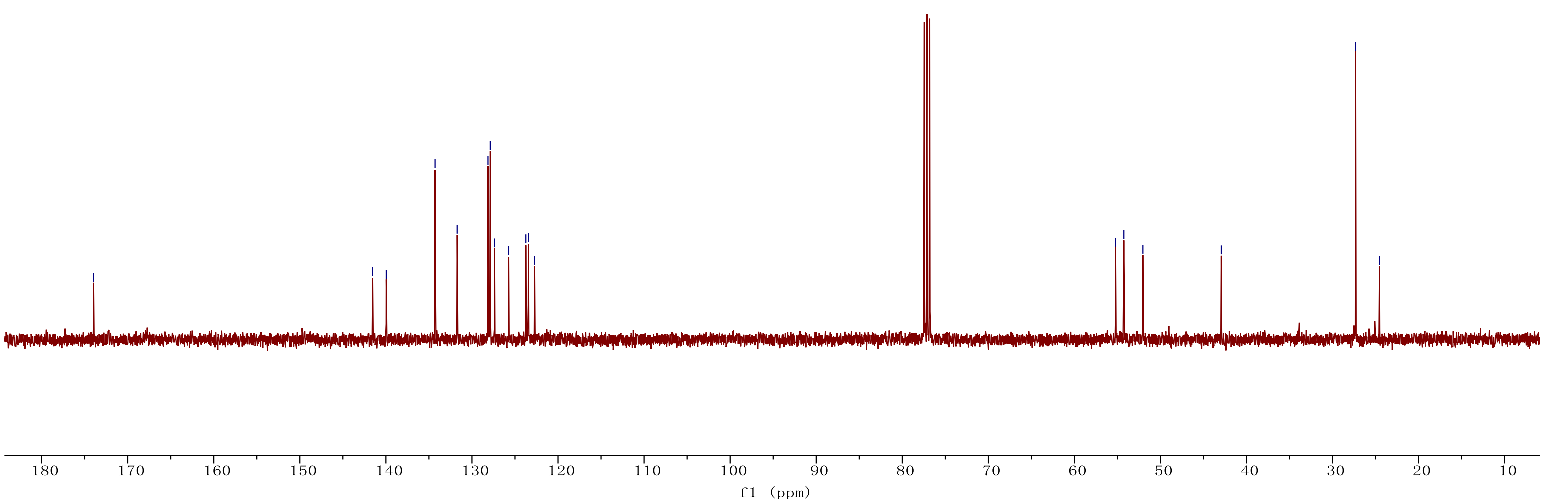

^{1}H NMR (500 MHz, $CDCl_3$) of **2q**

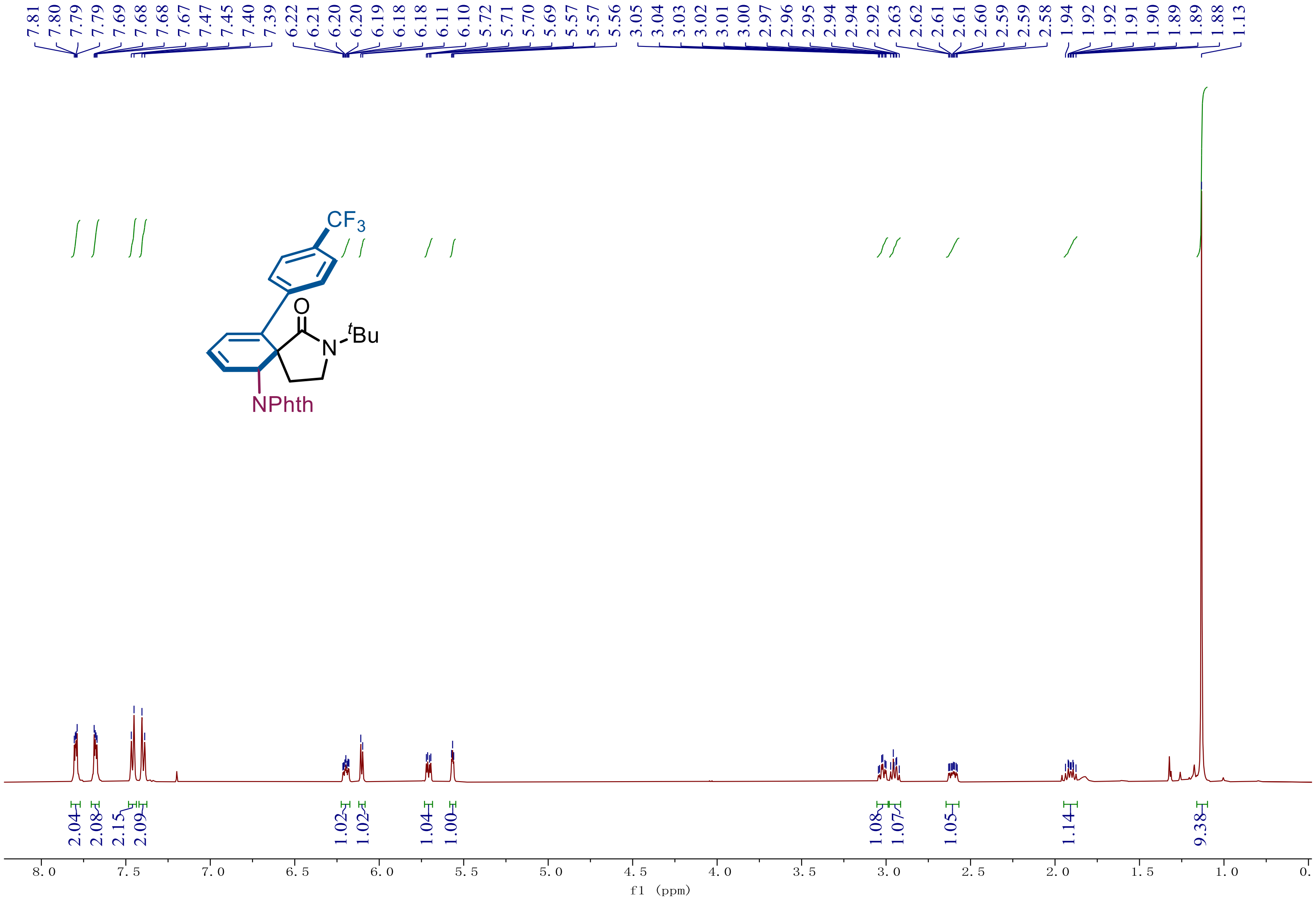

^{13}C NMR (126 MHz, $CDCl_3$) of **2q**

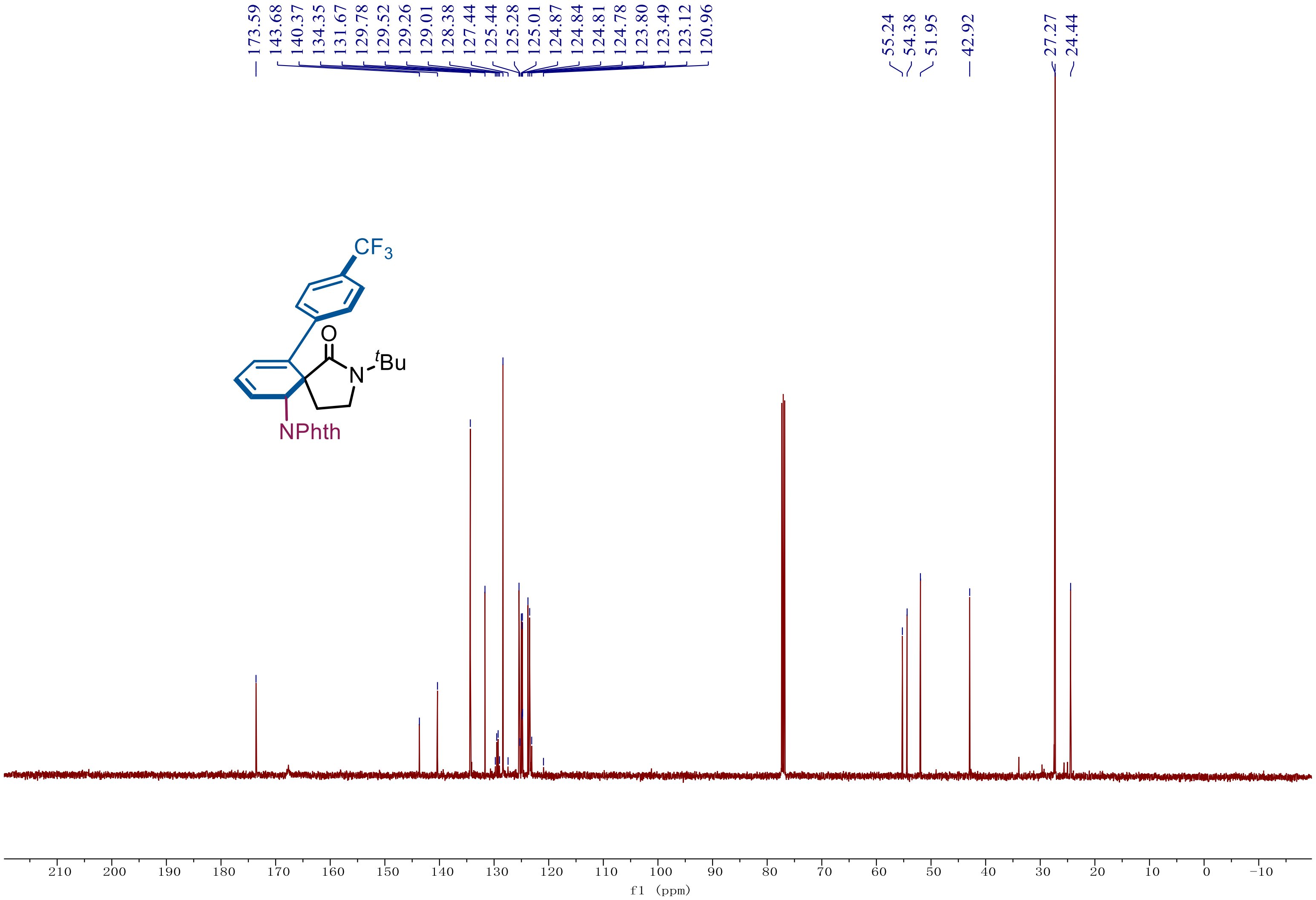

^{19}F NMR (471 MHz, $CDCl_3$) of **2q**

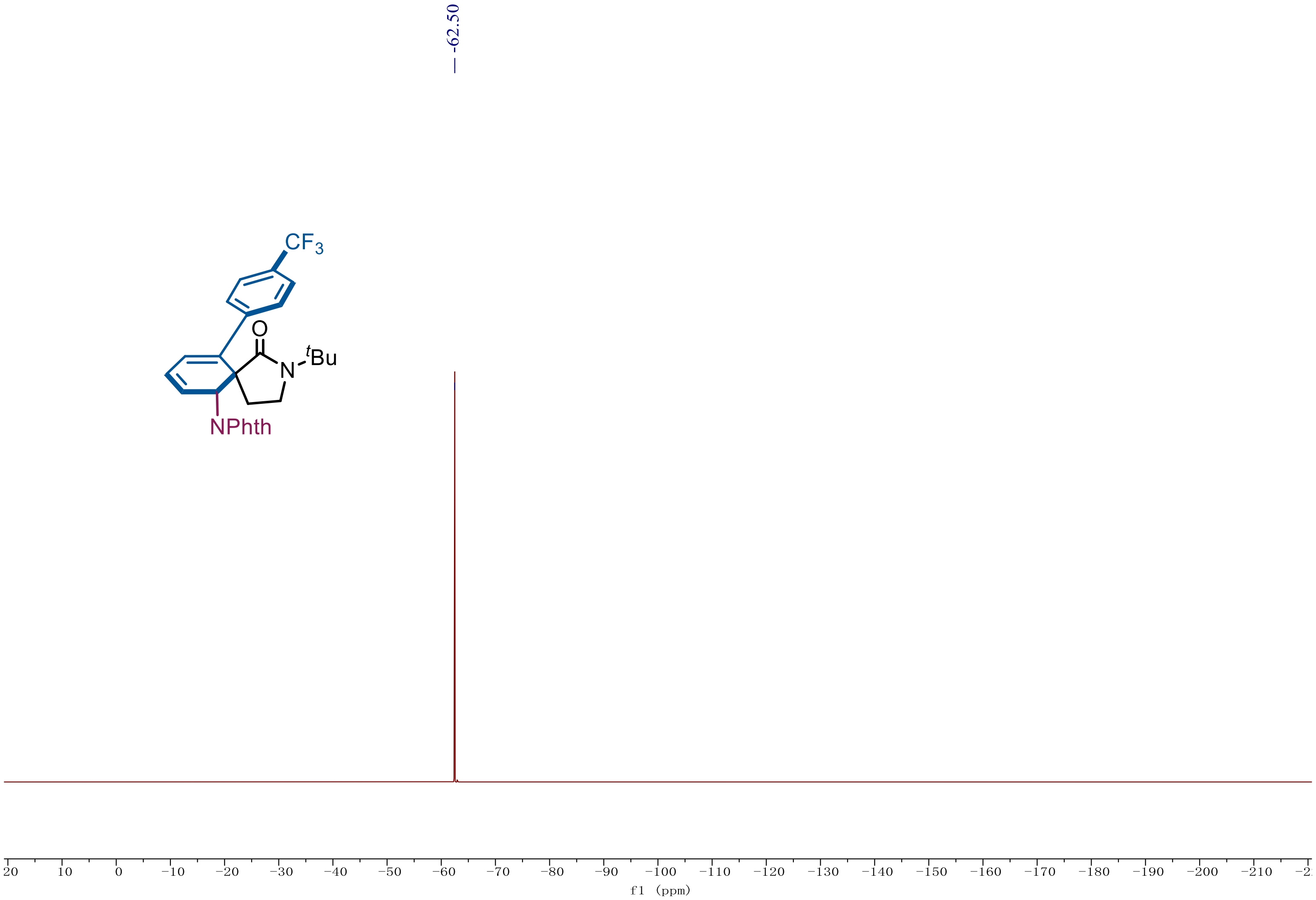

^{1}H NMR (400 MHz, $CDCl_3$) of **2r**

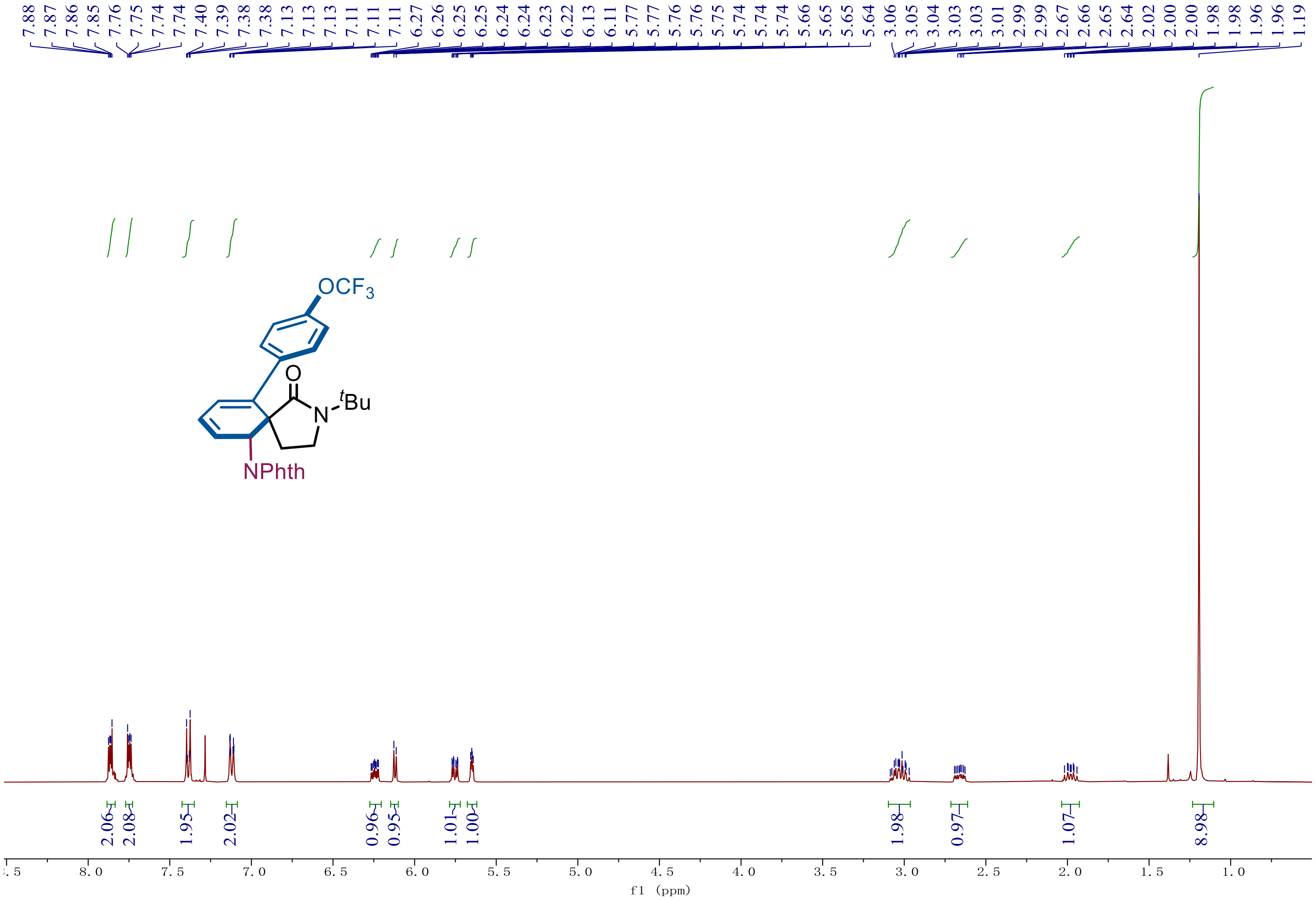

^{13}C NMR (126 MHz, CDCl$_3$) of **2r**

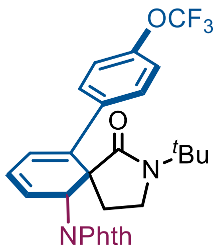

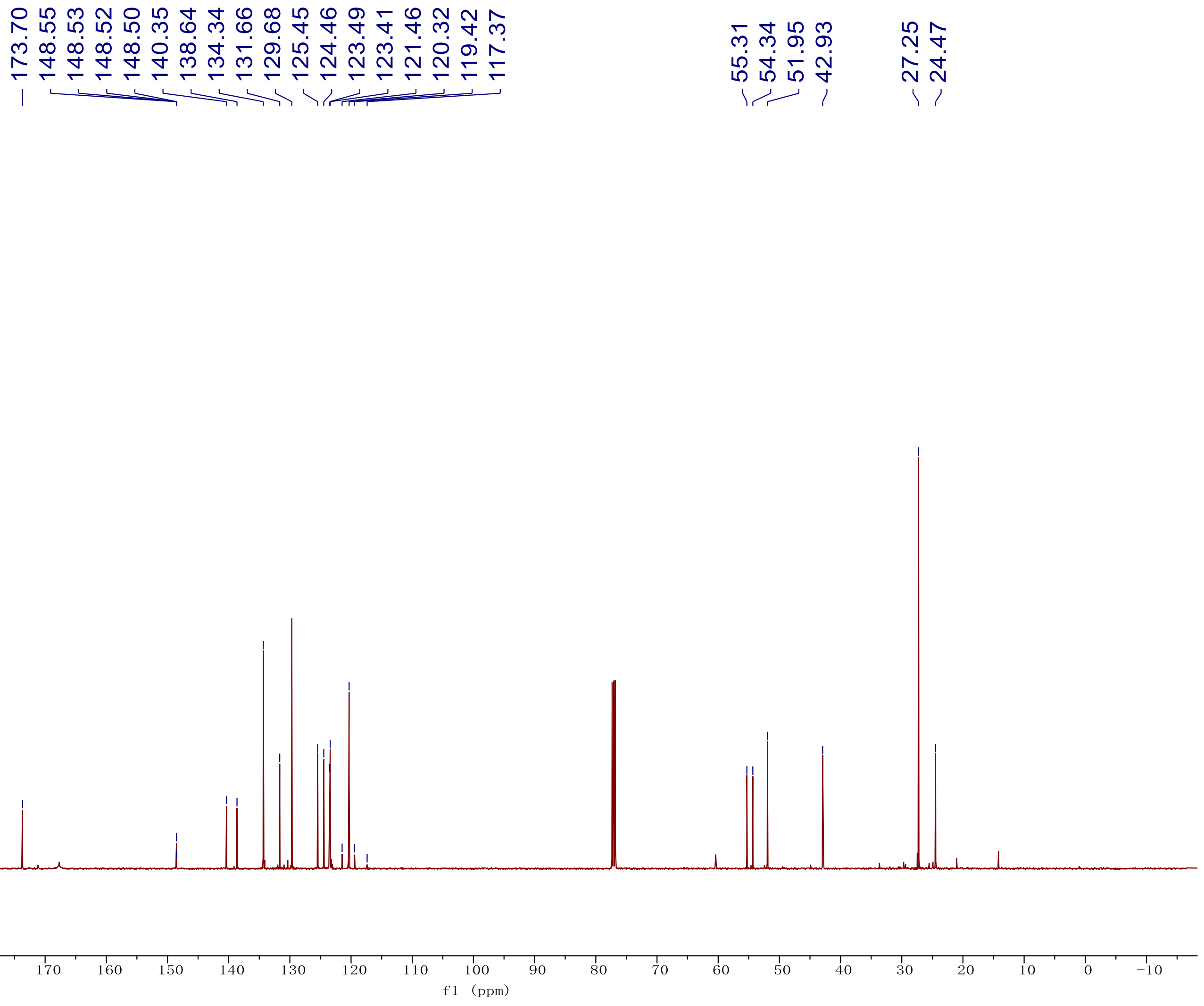

^{19}F NMR (376 MHz, $CDCl_3$) of **2r**

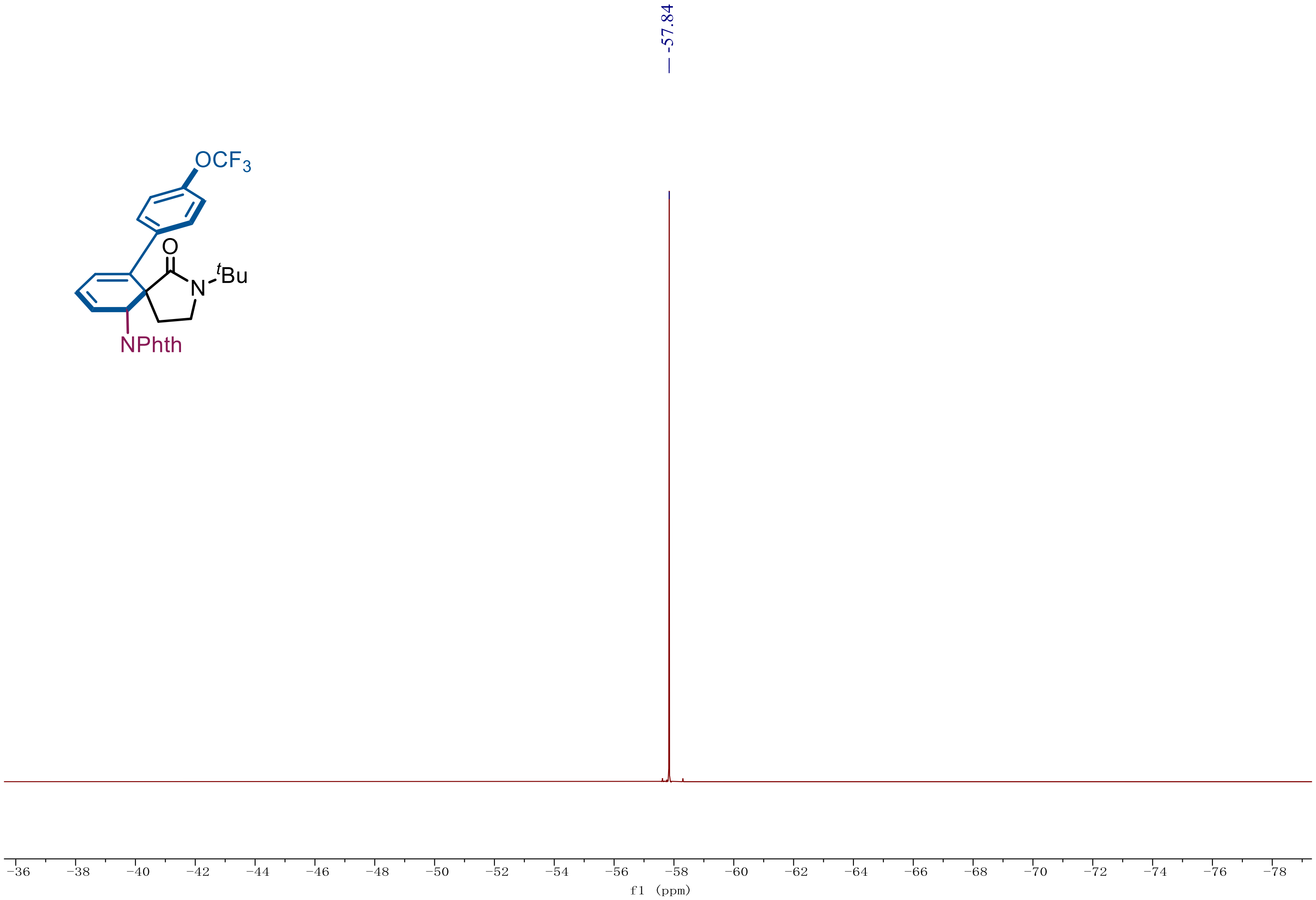

^{1}H NMR (400 MHz, CDCl$_3$) of **2s**

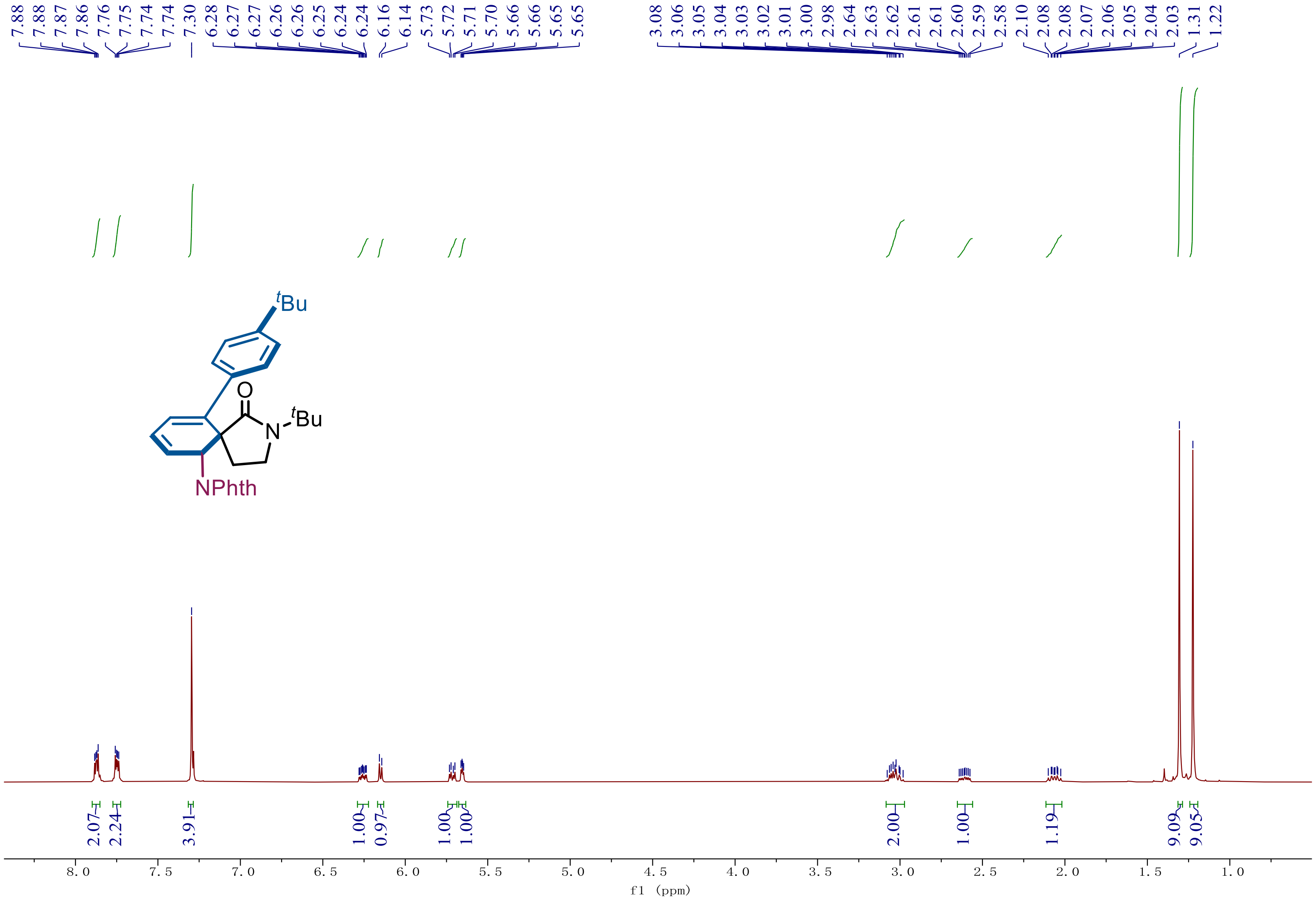

^{13}C NMR (101 MHz, $CDCl_3$) of **2s**

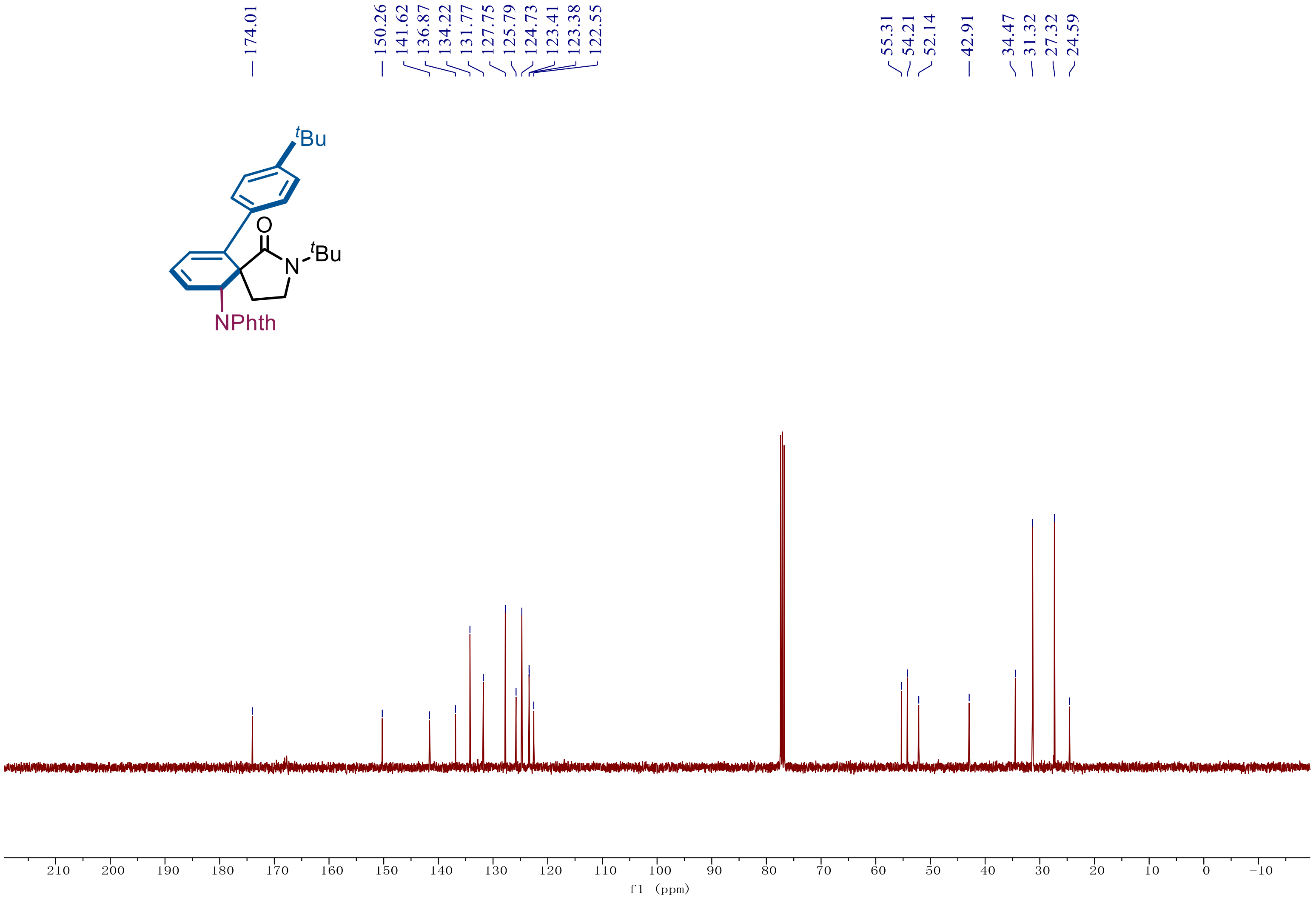

^{1}H NMR (400 MHz, $CDCl_3$) of **2t**

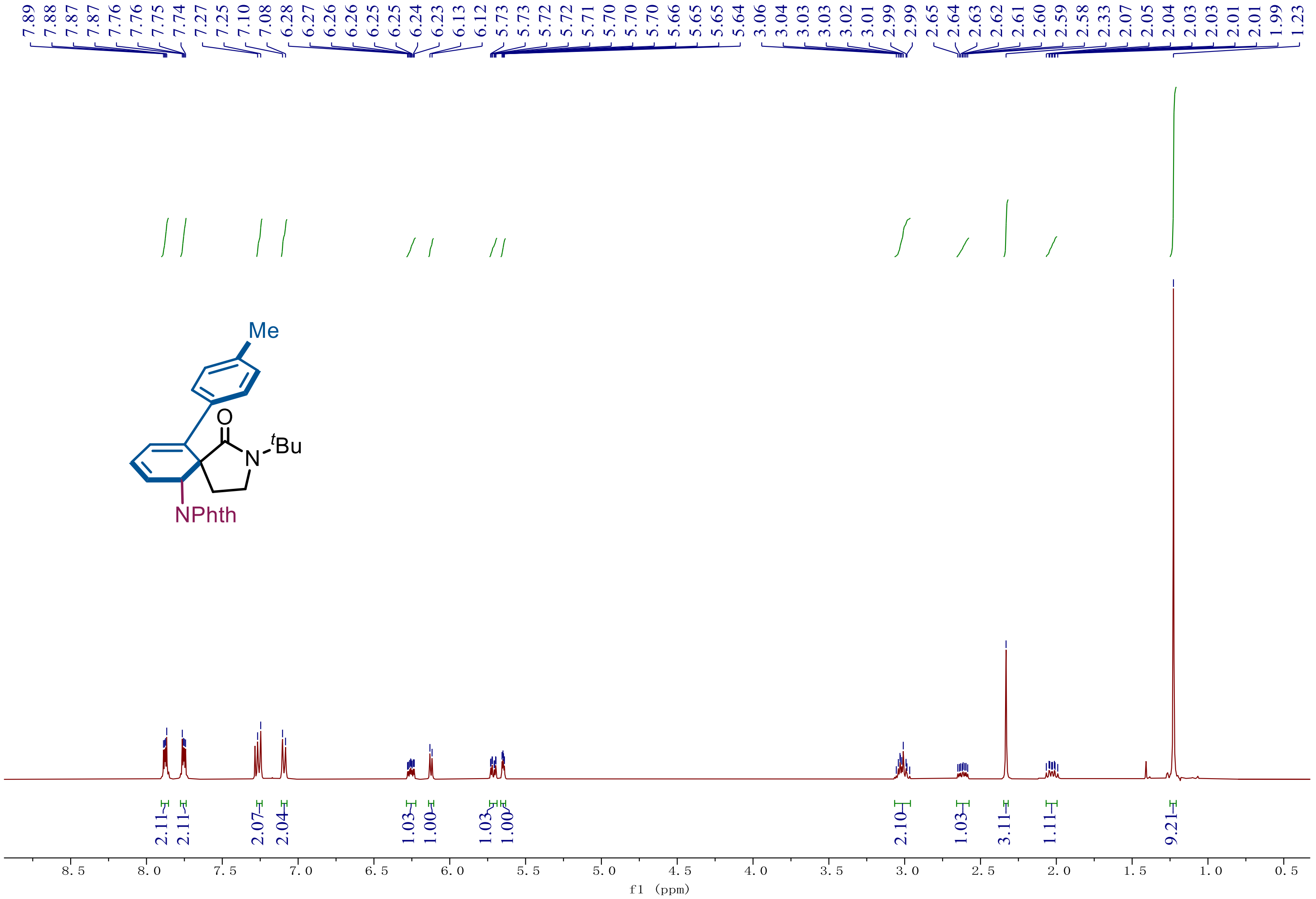

^{13}C NMR (101 MHz, CDCl$_3$) of **2t**

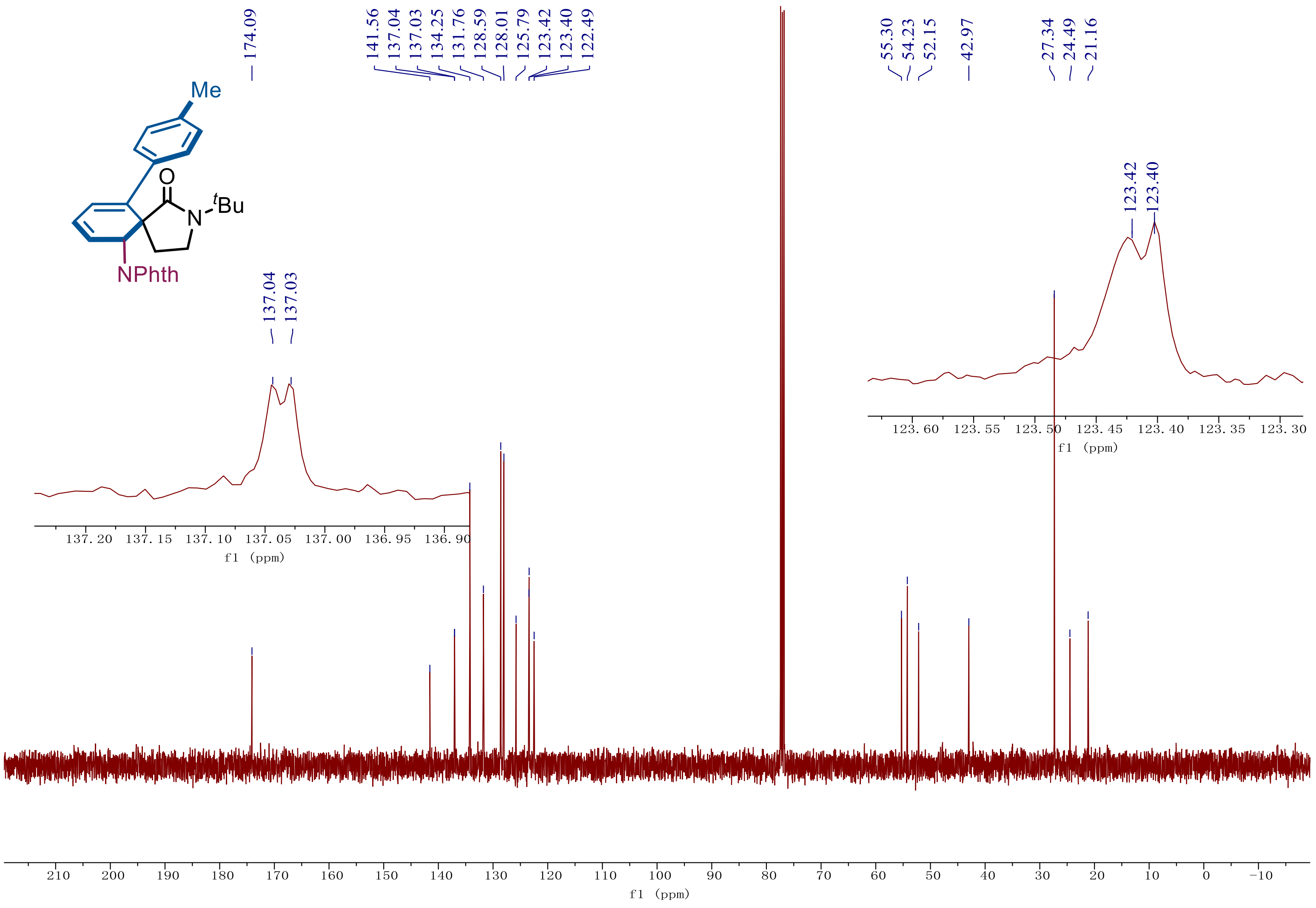

^{1}H NMR (400 MHz, $CDCl_3$) of **2u**

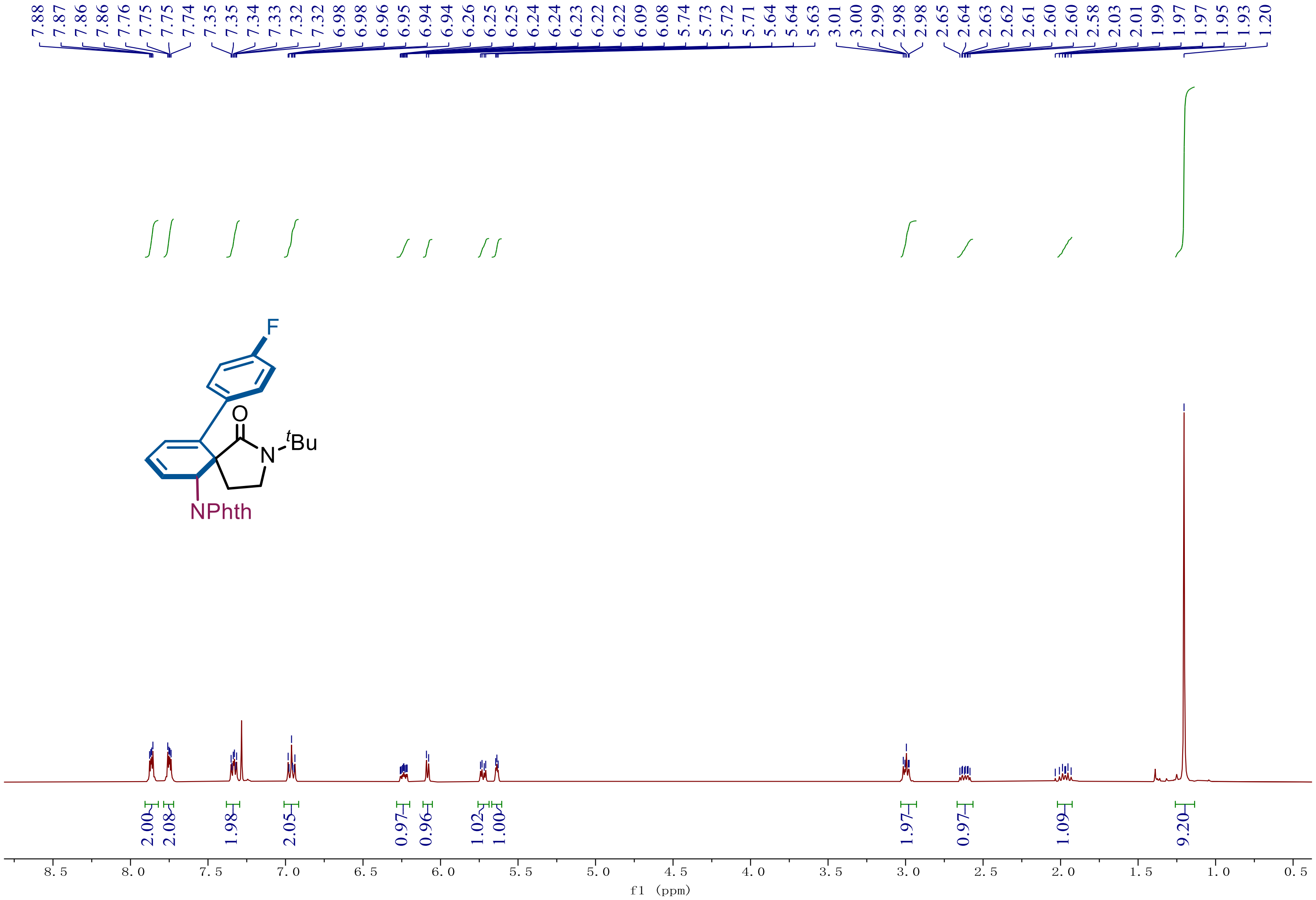

^{13}C NMR (101 MHz, $CDCl_3$) of **2u**

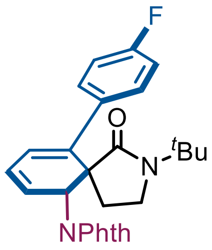

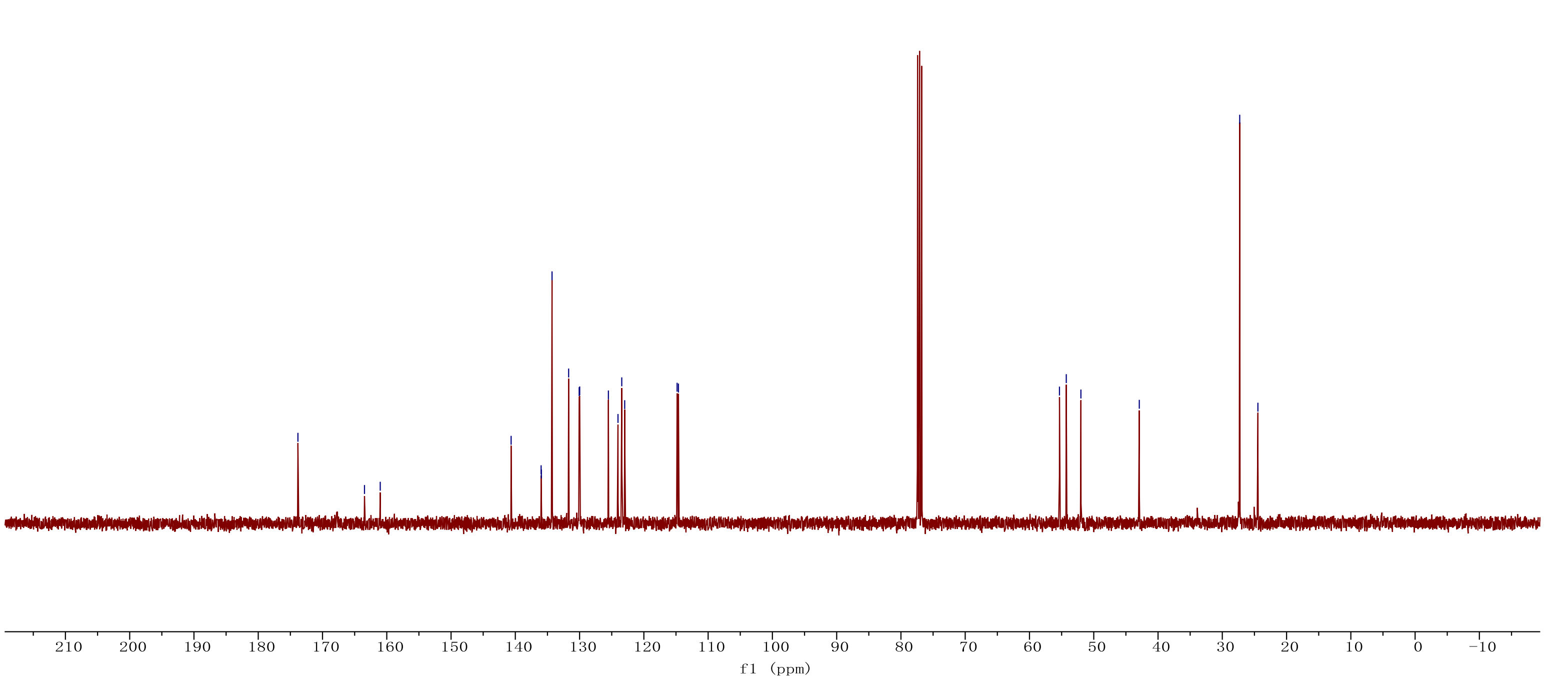

^{19}F NMR (376 MHz, $CDCl_3$) of **2u**

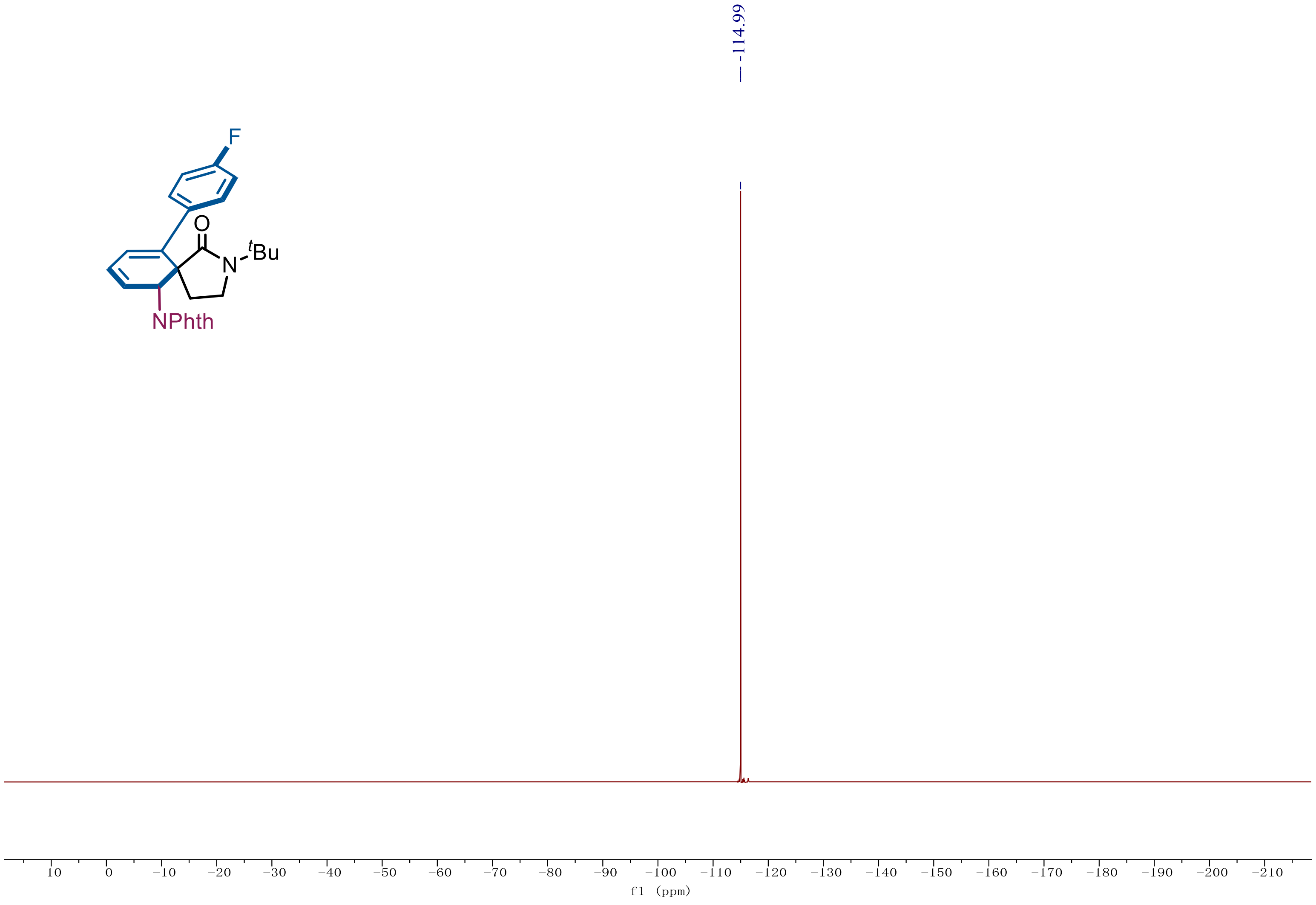

^{1}H NMR (400 MHz, $CDCl_3$) of **2v**

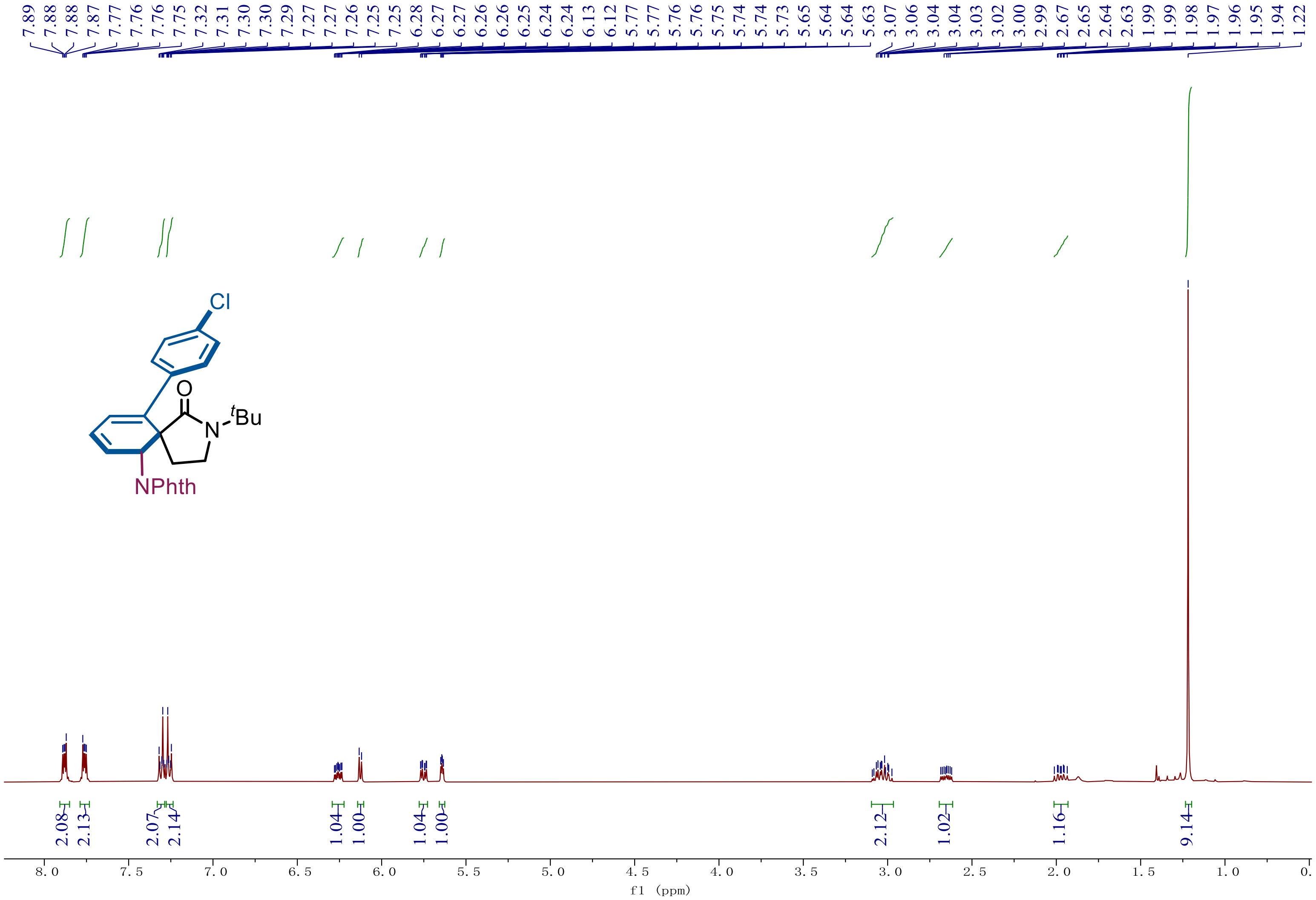

^{13}C NMR (101 MHz, CDCl$_3$) of **2v**

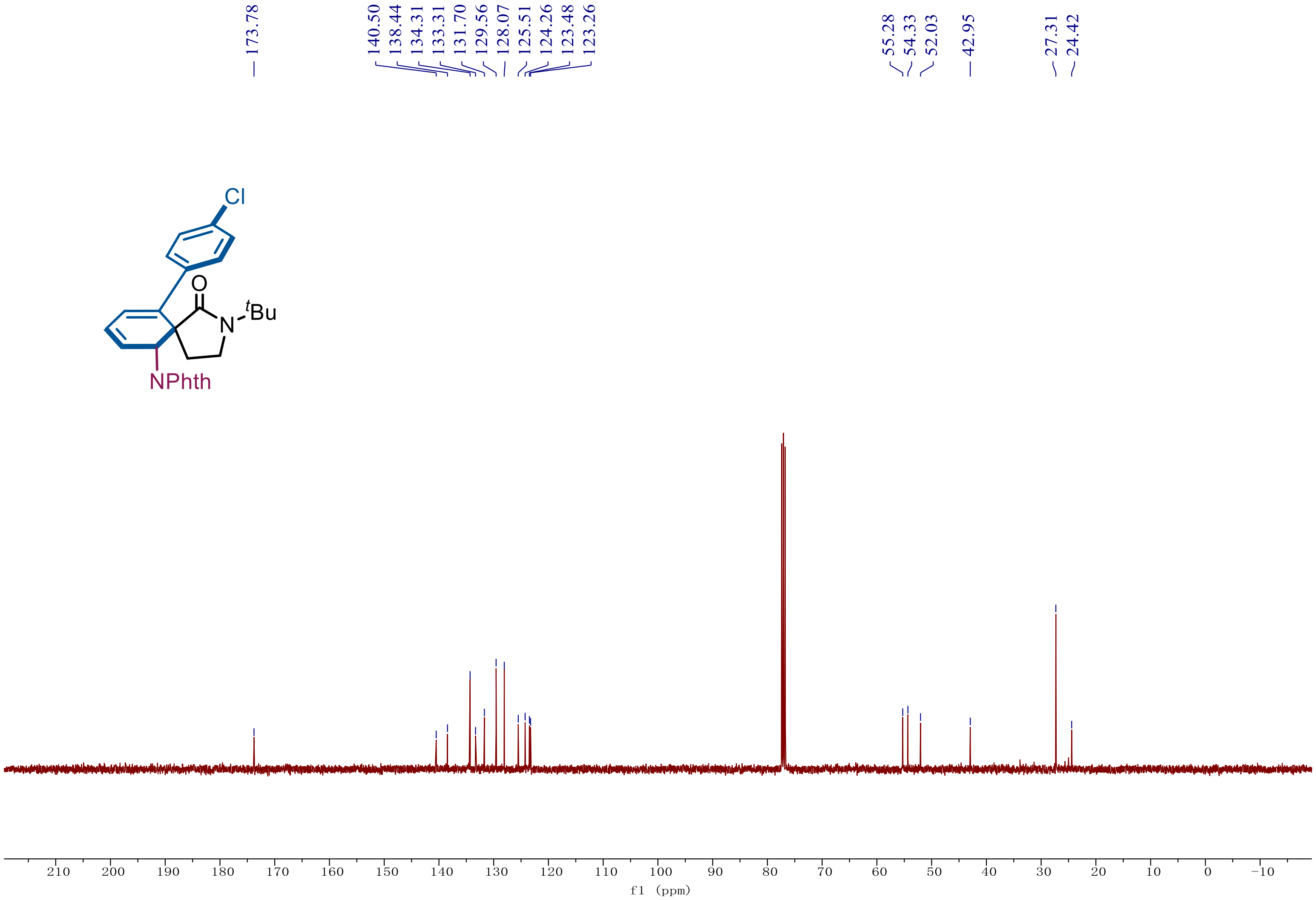

^{1}H NMR (400 MHz, $CDCl_3$) of **2w**

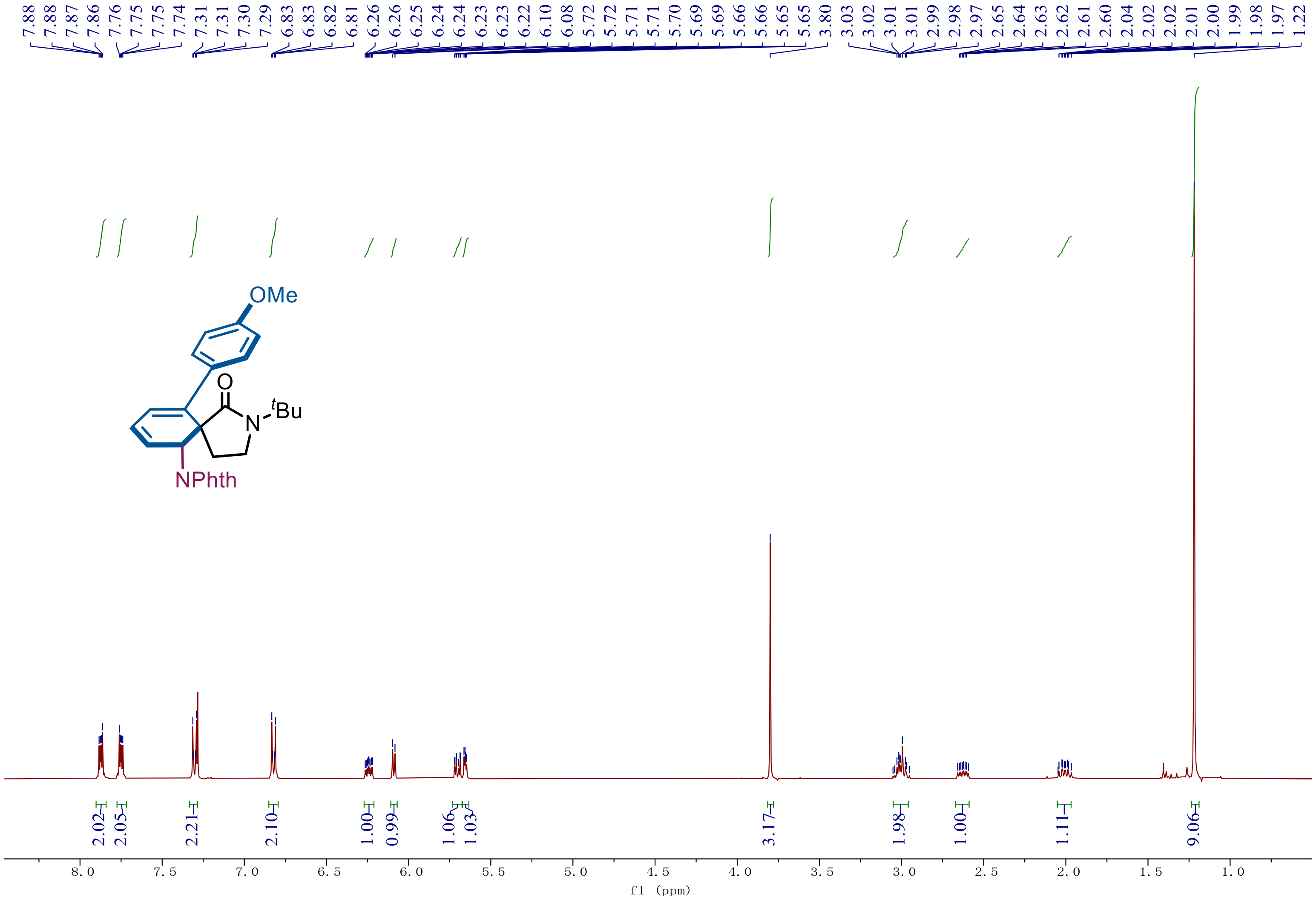

^{13}C NMR (101 MHz, CDCl$_3$) of **2w**

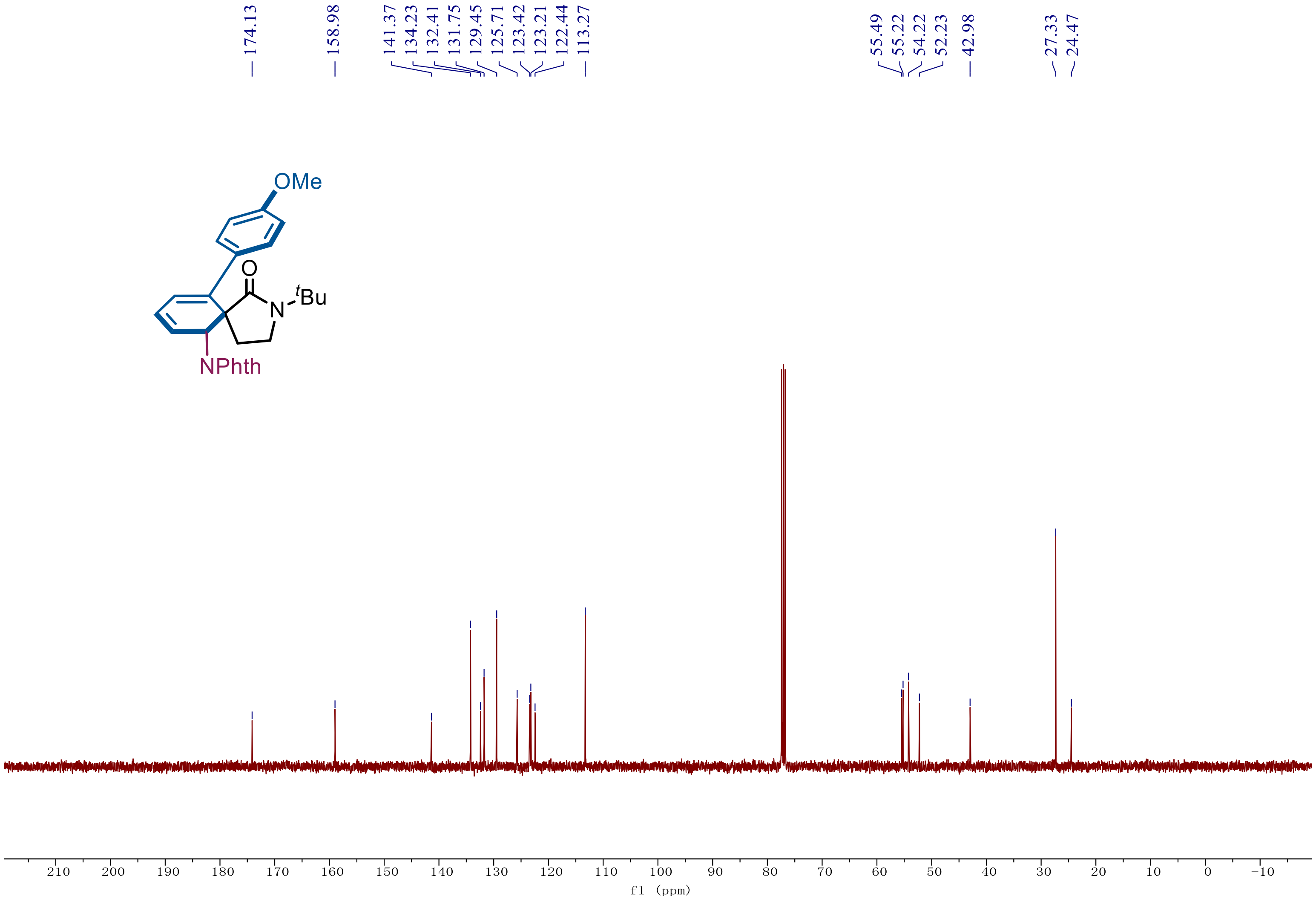

^{1}H NMR (400 MHz, $CDCl_3$) of **2x**

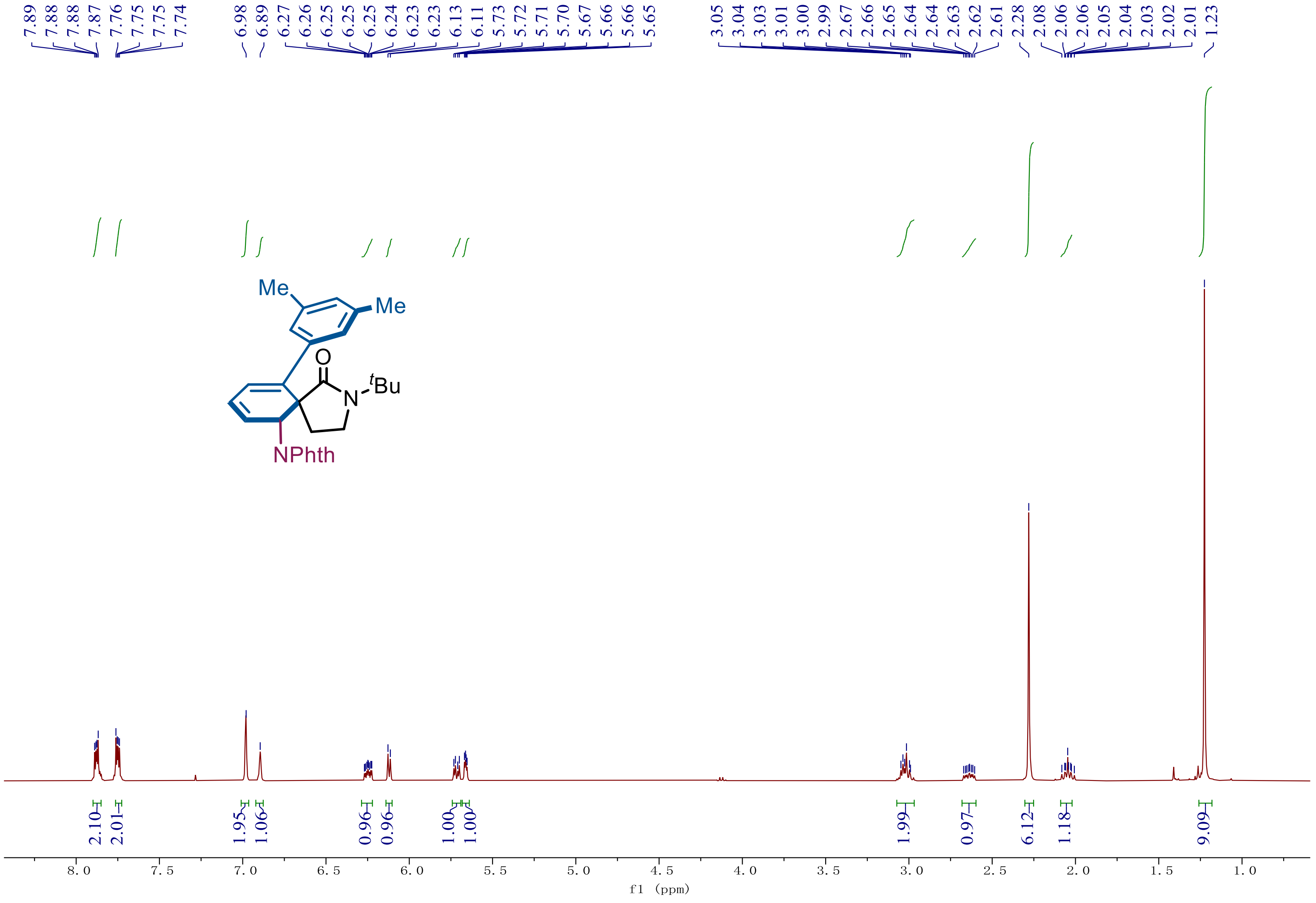

^{13}C NMR (101 MHz, $CDCl_3$) of **2x**

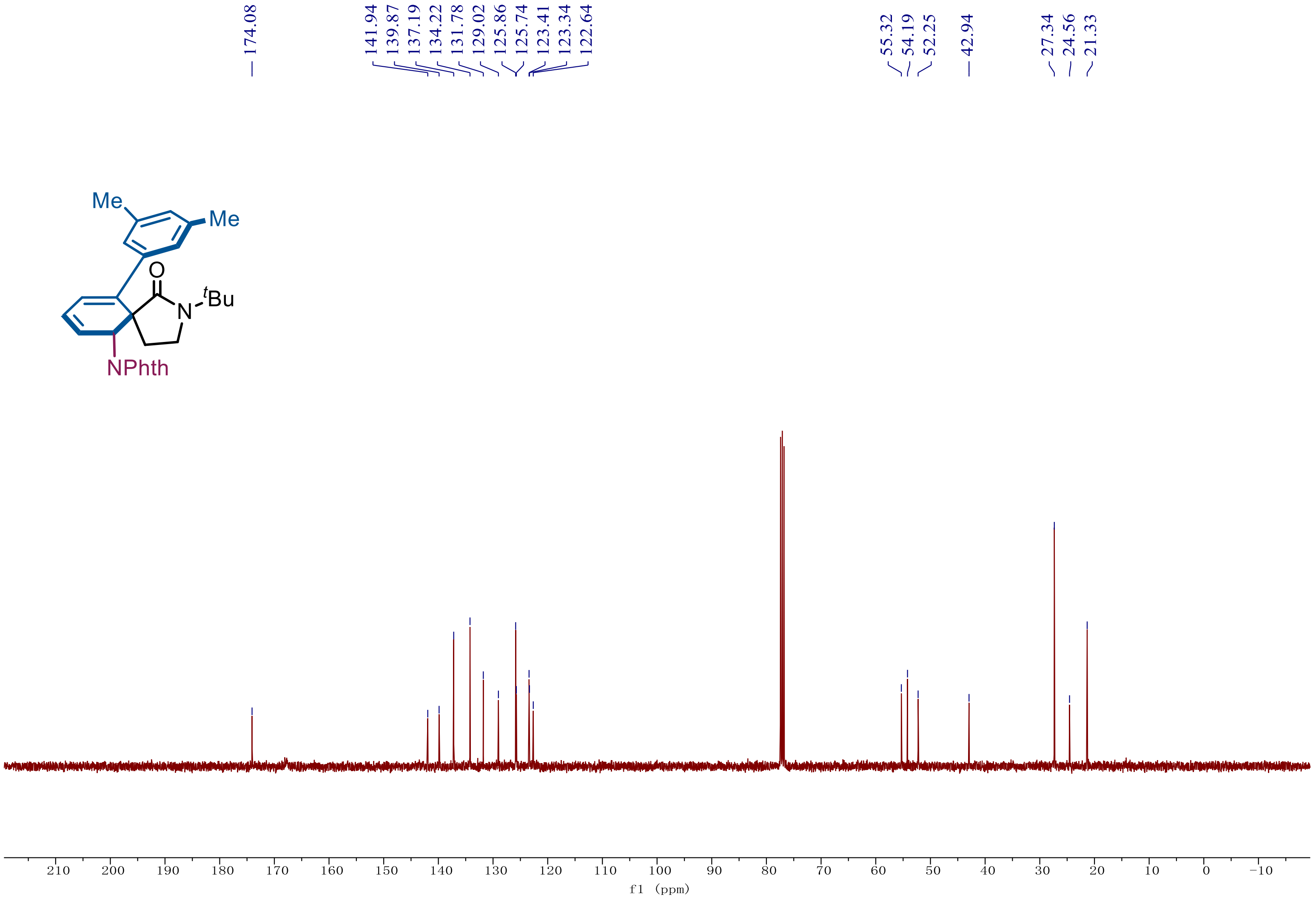

^{1}H NMR (400 MHz, $CDCl_3$) of **2y**

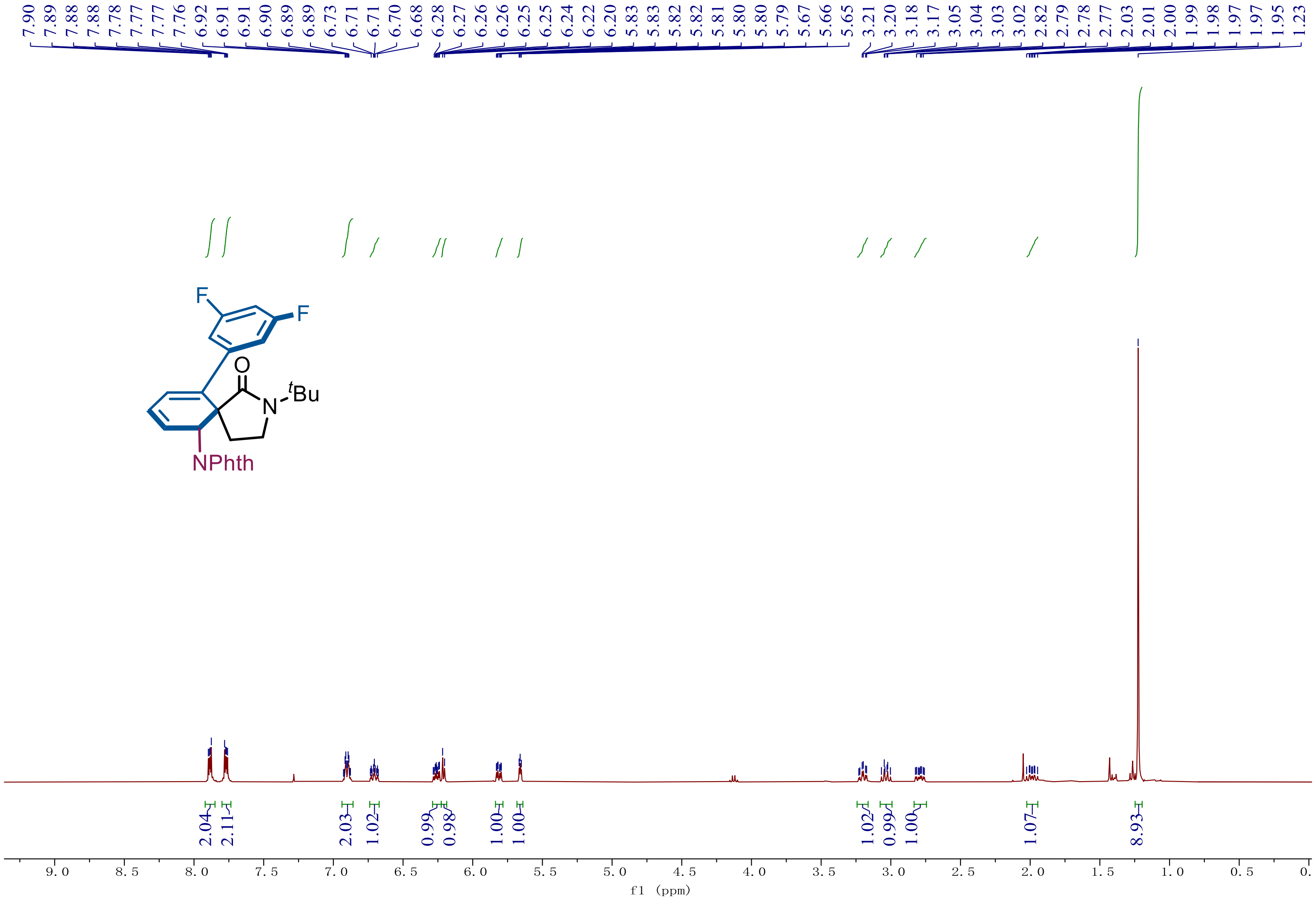

^{13}C NMR (101 MHz, $CDCl_3$) of **2y**

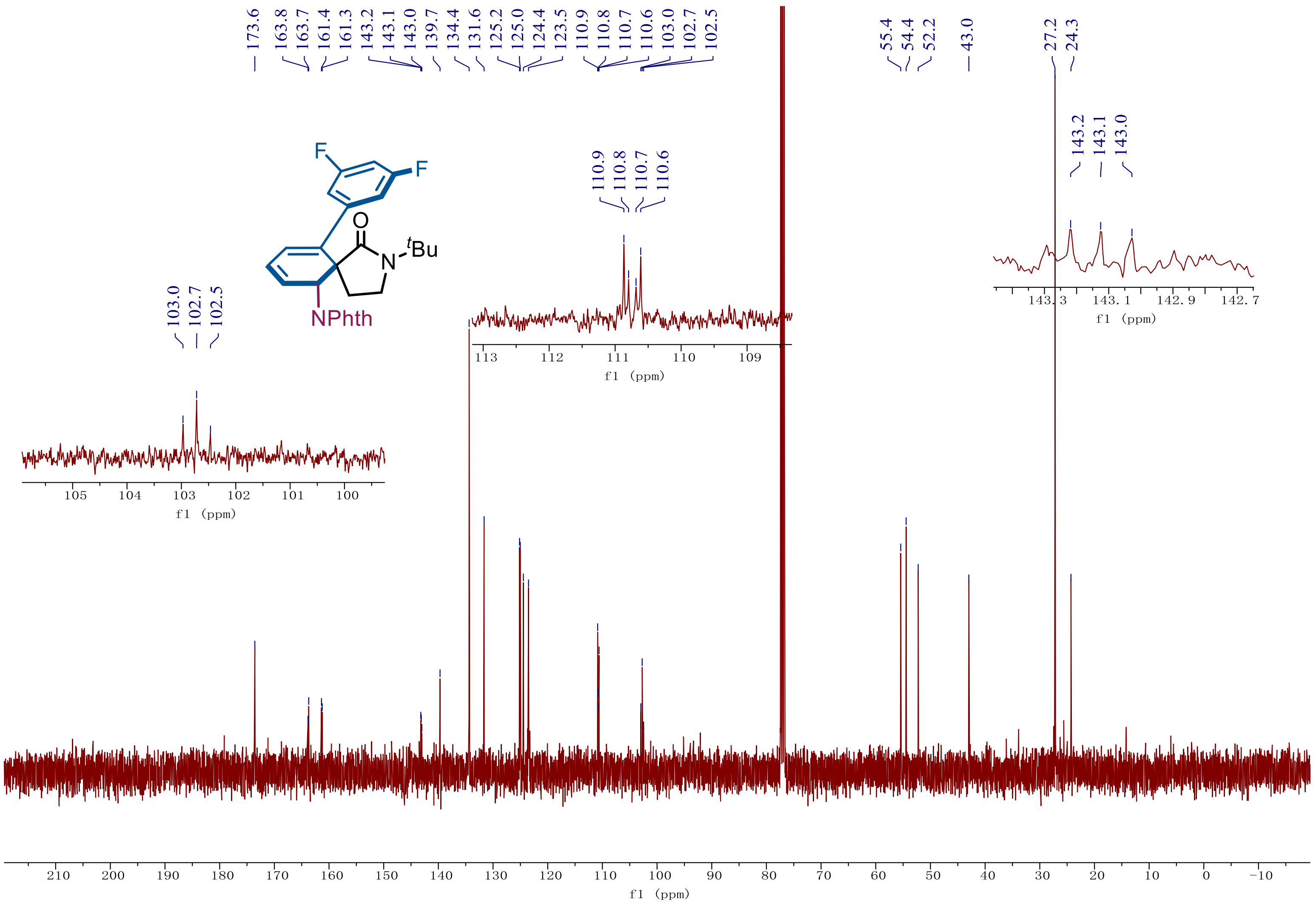

^{19}F NMR (376 MHz, $CDCl_3$) of **2y**

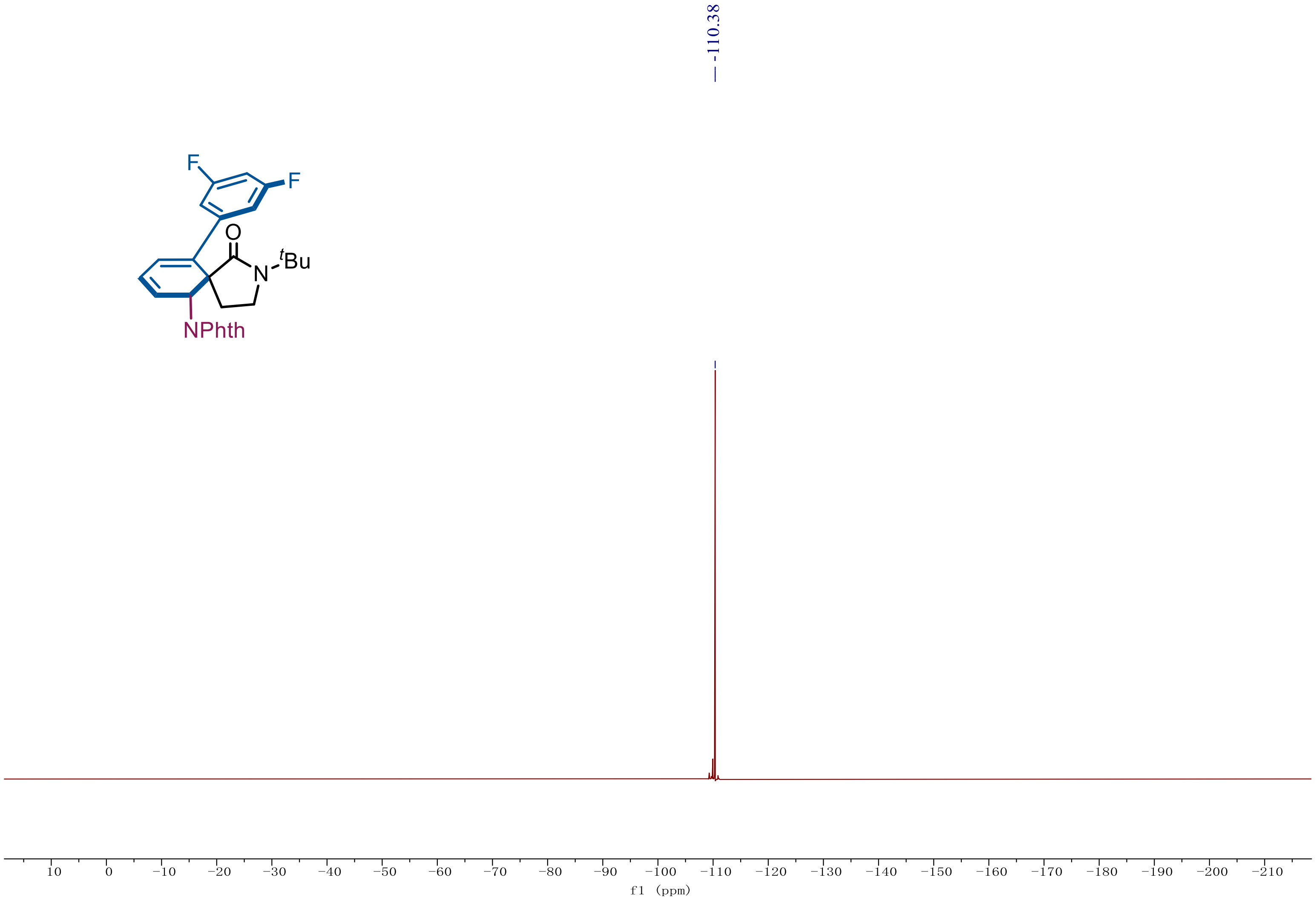

^{1}H NMR (500 MHz, $CDCl_3$) of **2z**

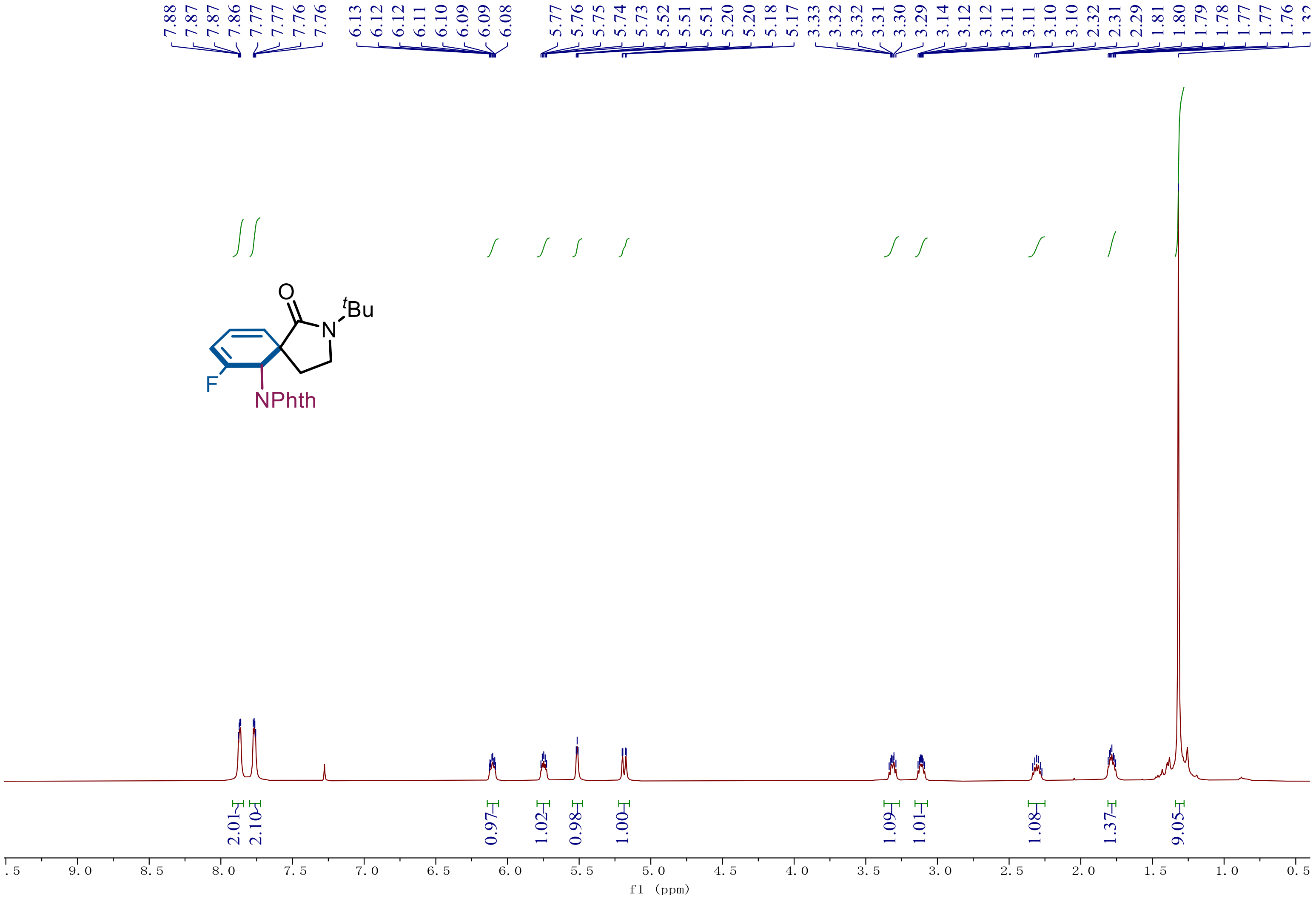

^{13}C NMR (126 MHz, $CDCl_3$) of **2z**

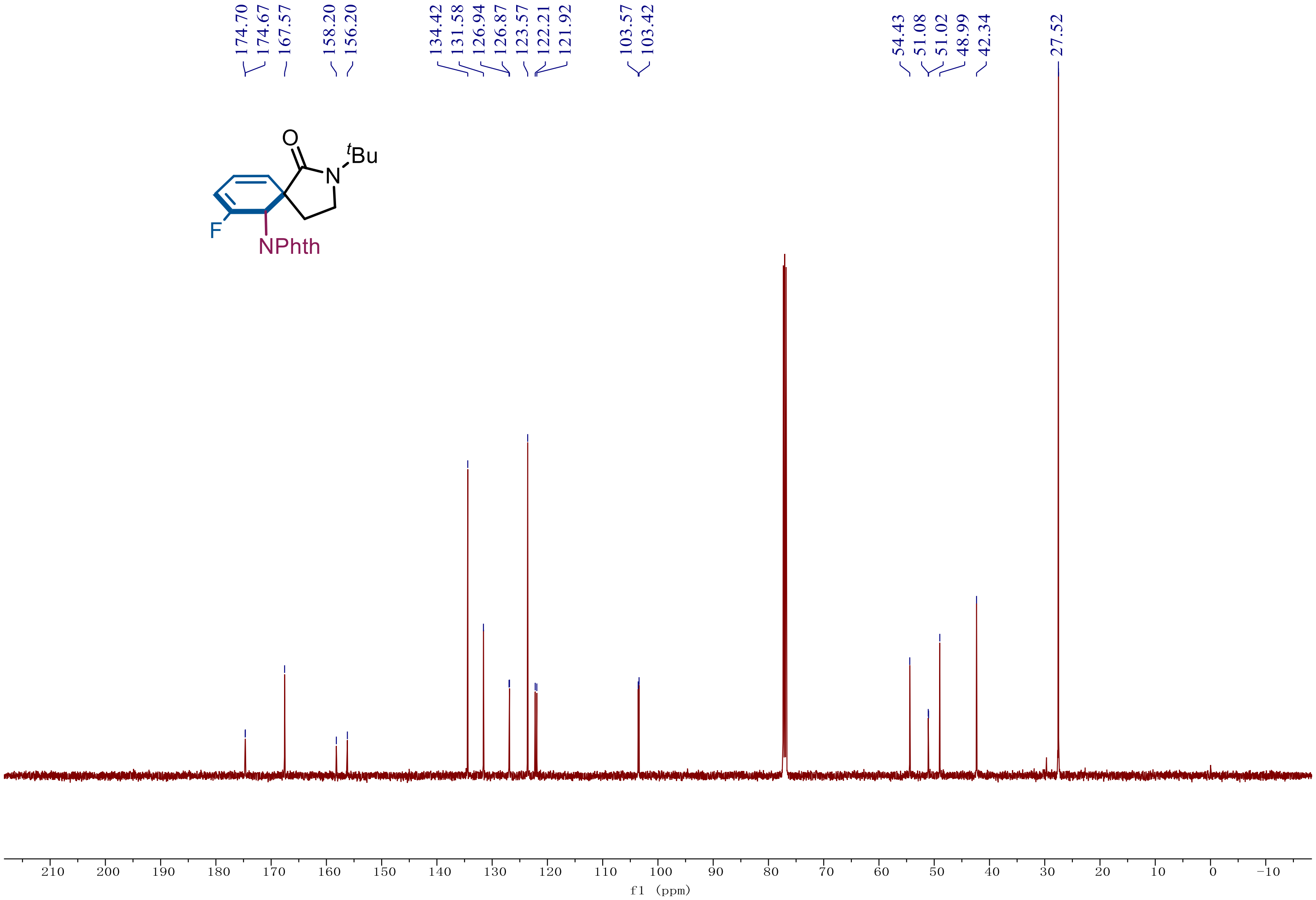

^{19}F NMR (471 MHz, $CDCl_3$) of **2z**

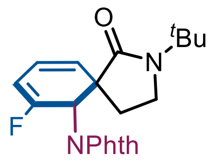

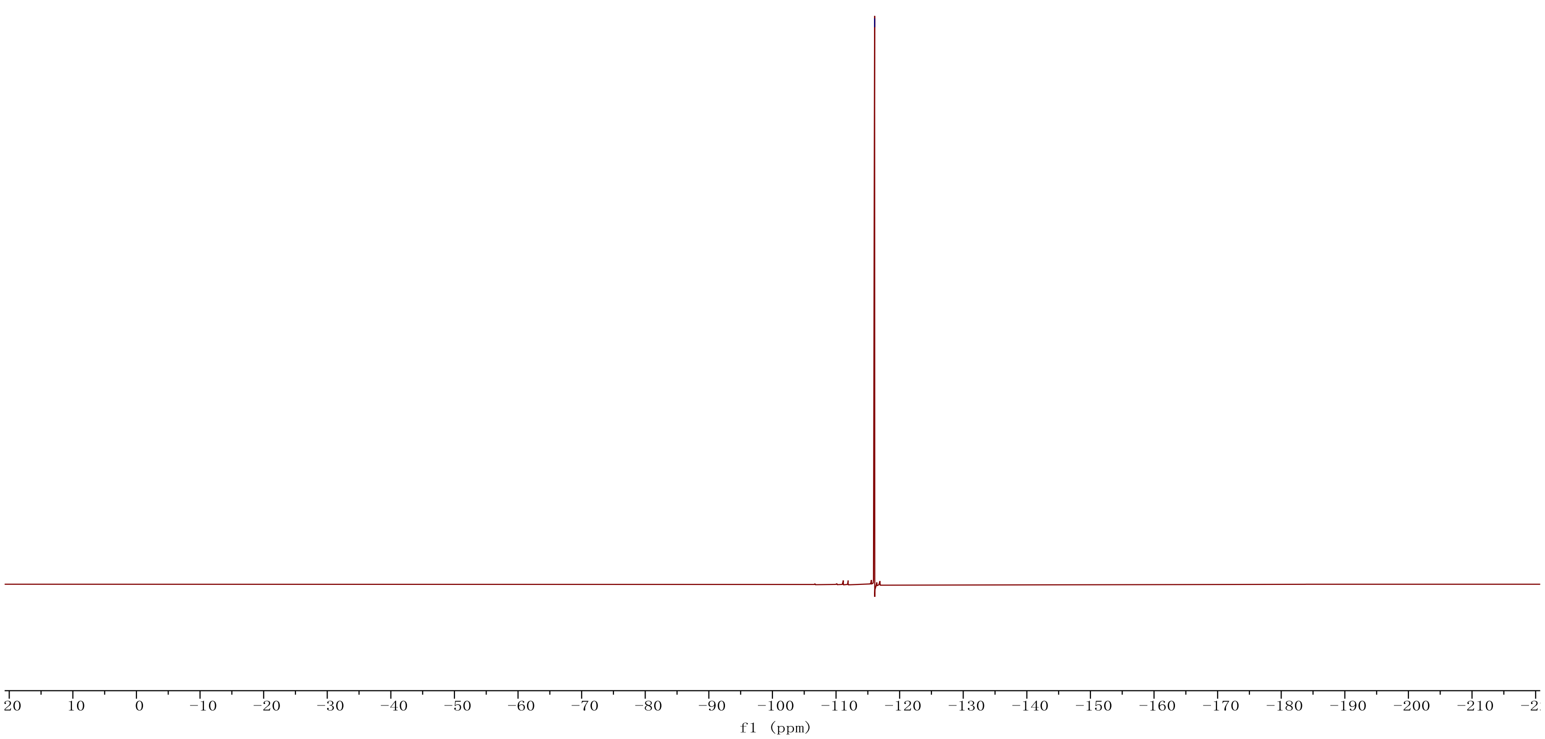

COSY of **2z**

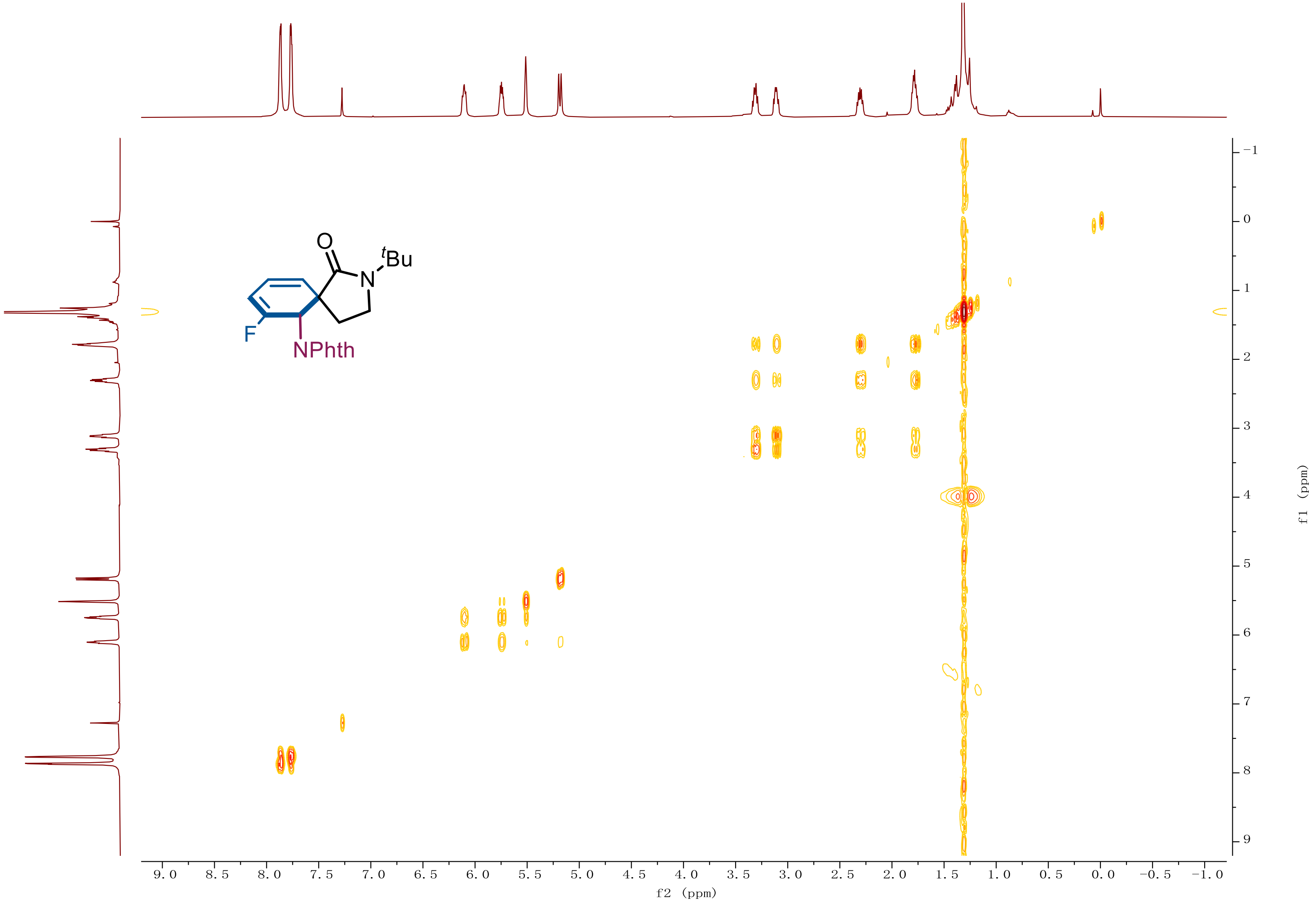

HMBC of **2z**

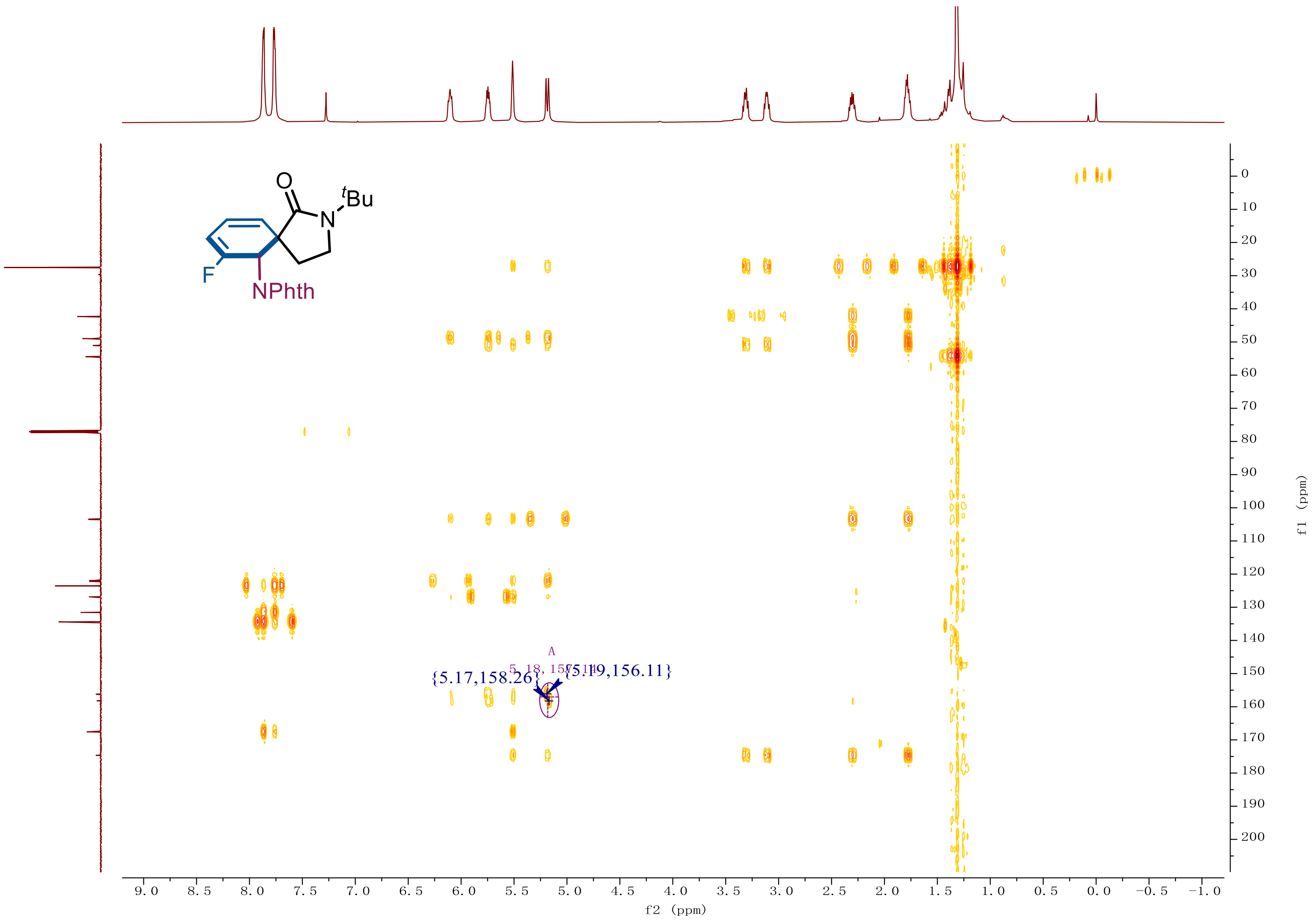

^{1}H NMR (400 MHz, $CDCl_3$) of **2aa**

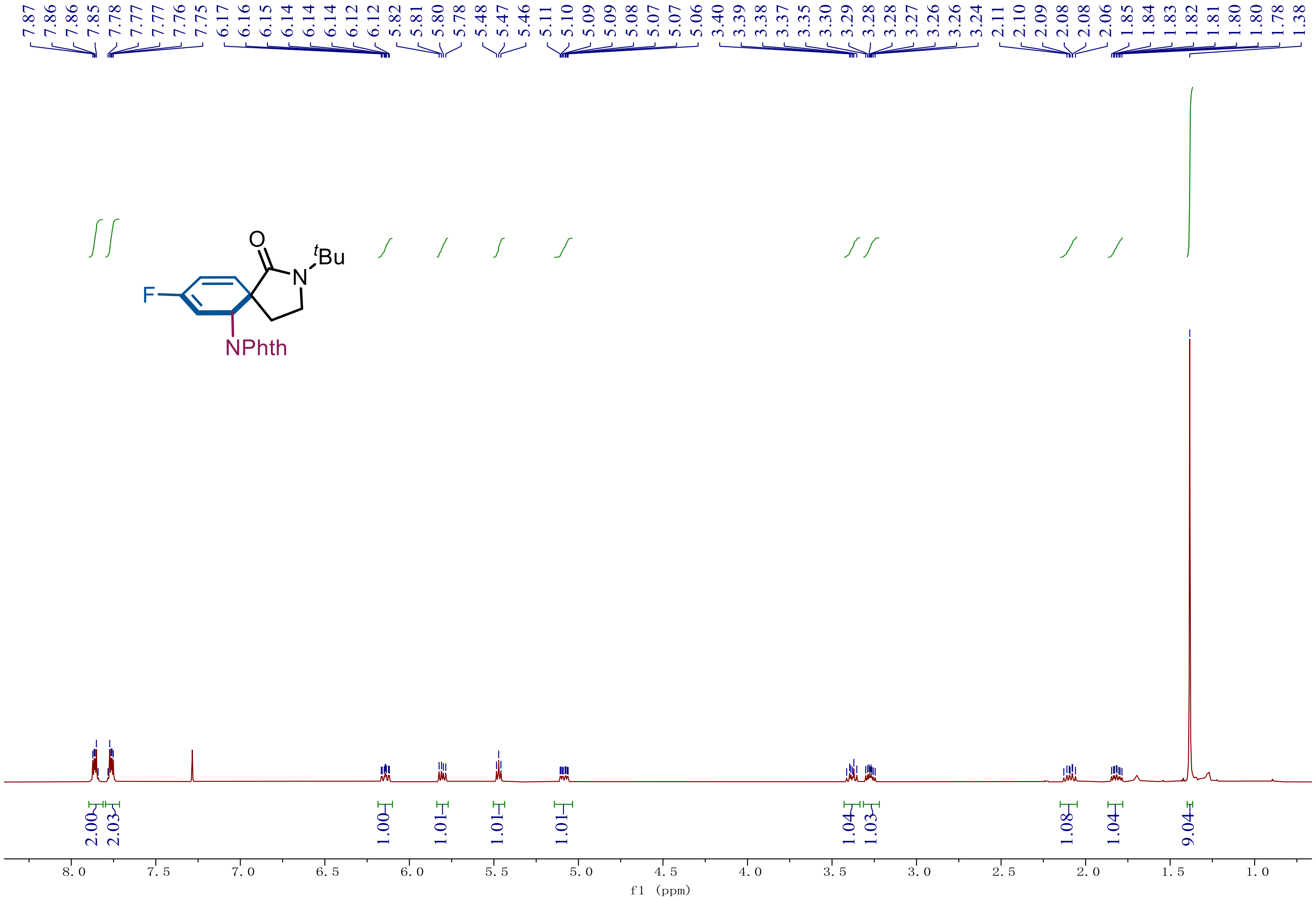

^{13}C NMR (101 MHz, CDCl$_3$) of **2aa**

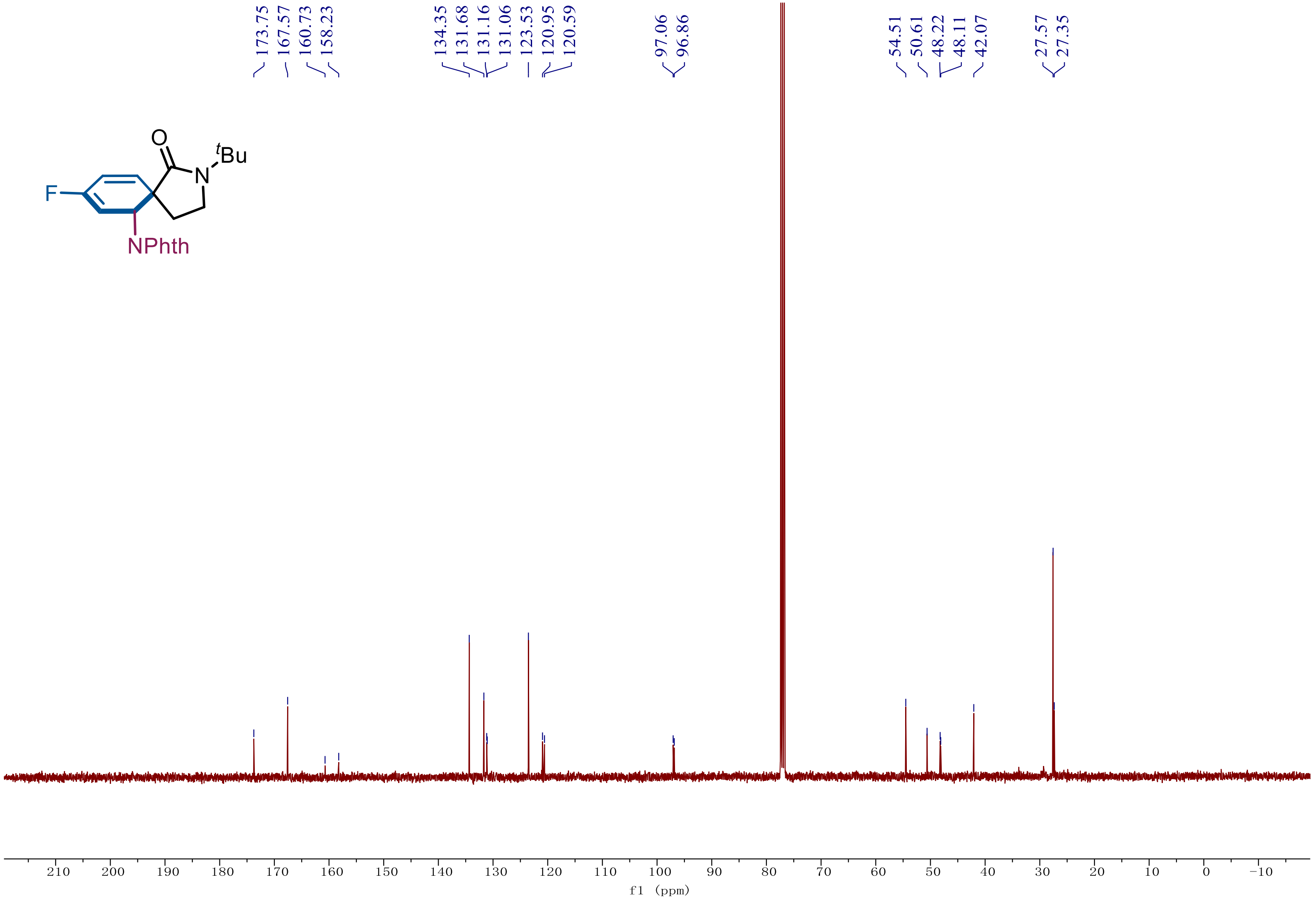

^{19}F NMR (376 MHz, $CDCl_3$) of **2aa**

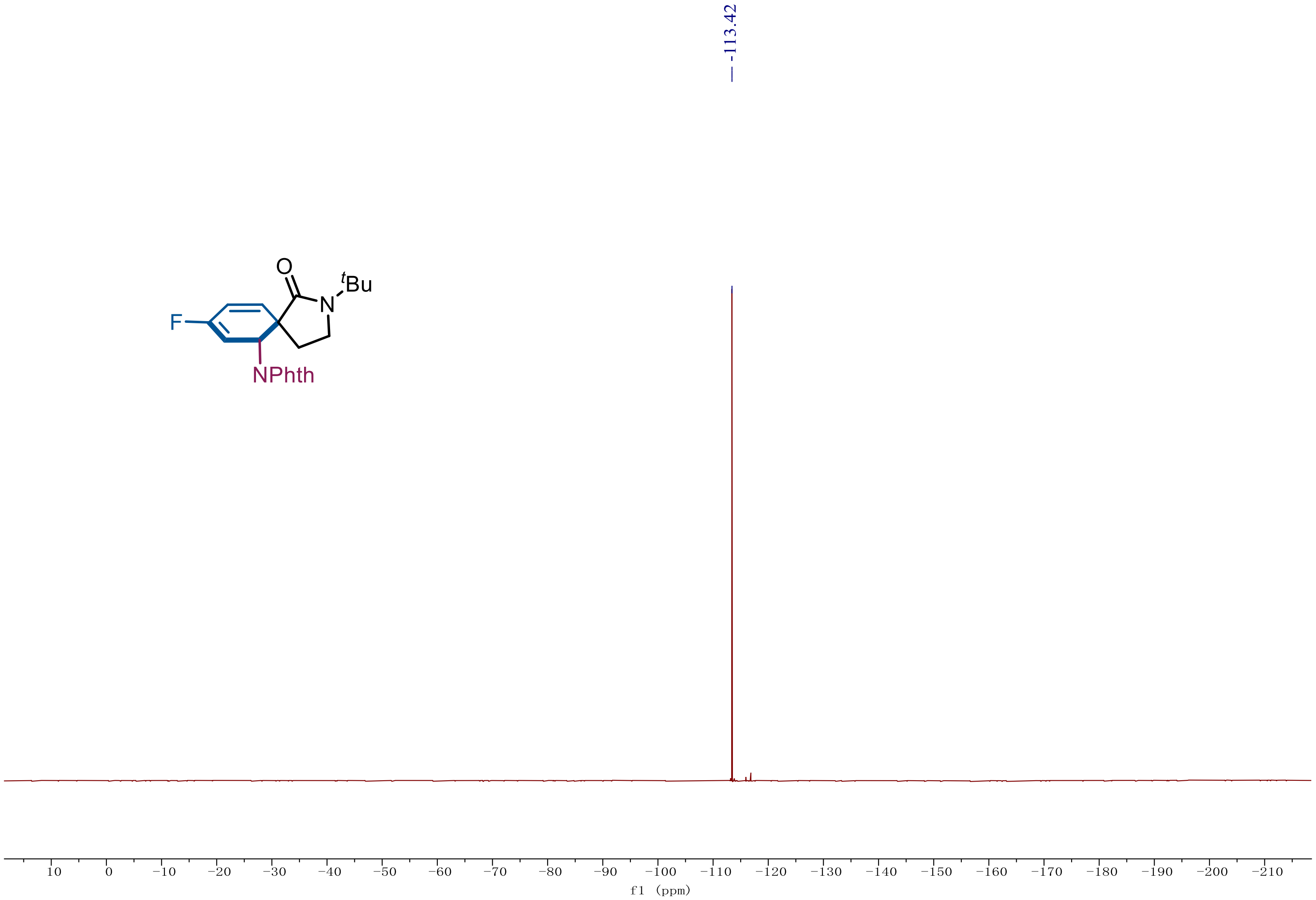

^{1}H NMR (400 MHz, $CDCl_3$) of **2ab**

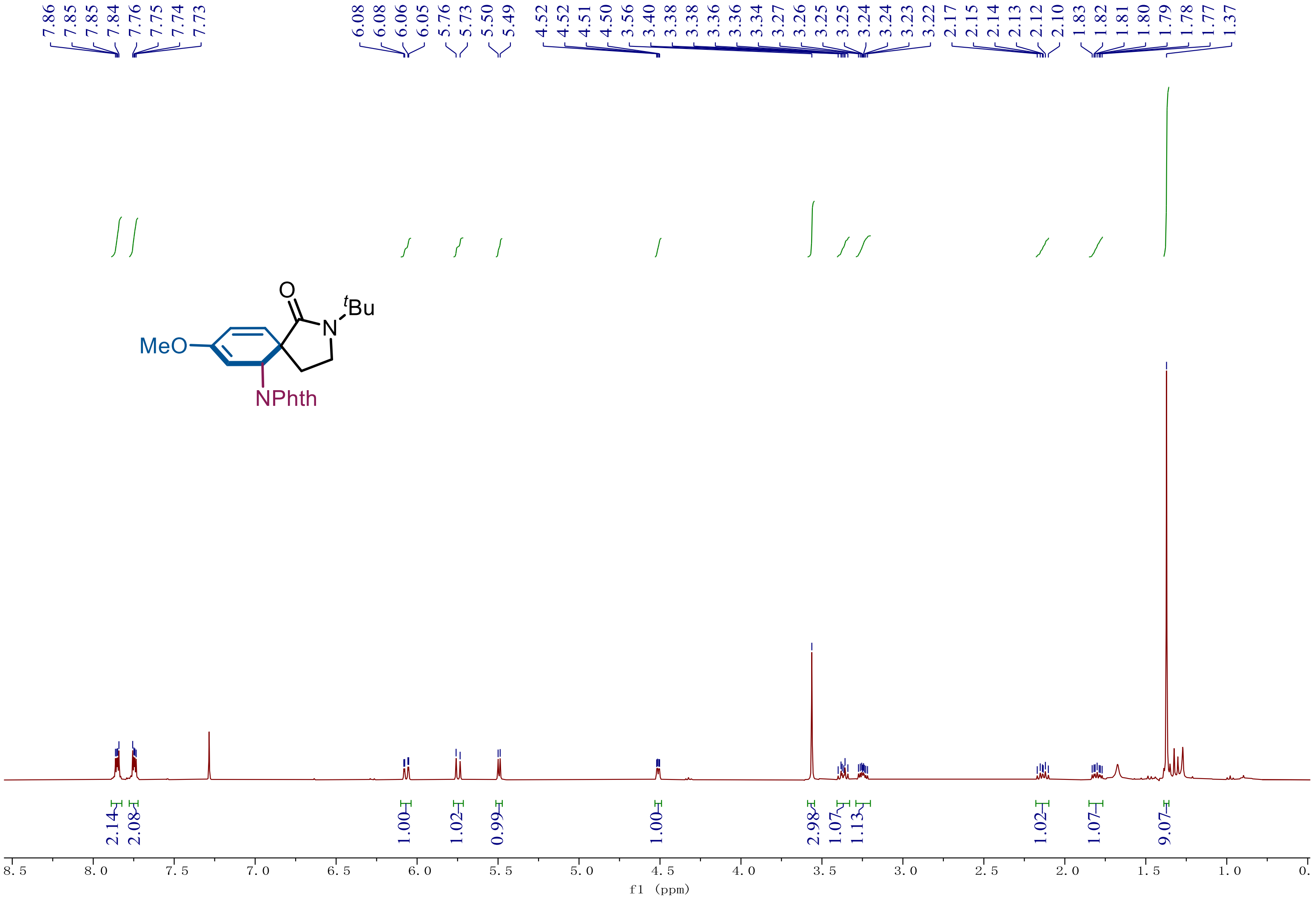

^{13}C NMR (101 MHz, $CDCl_3$) of **2ab**

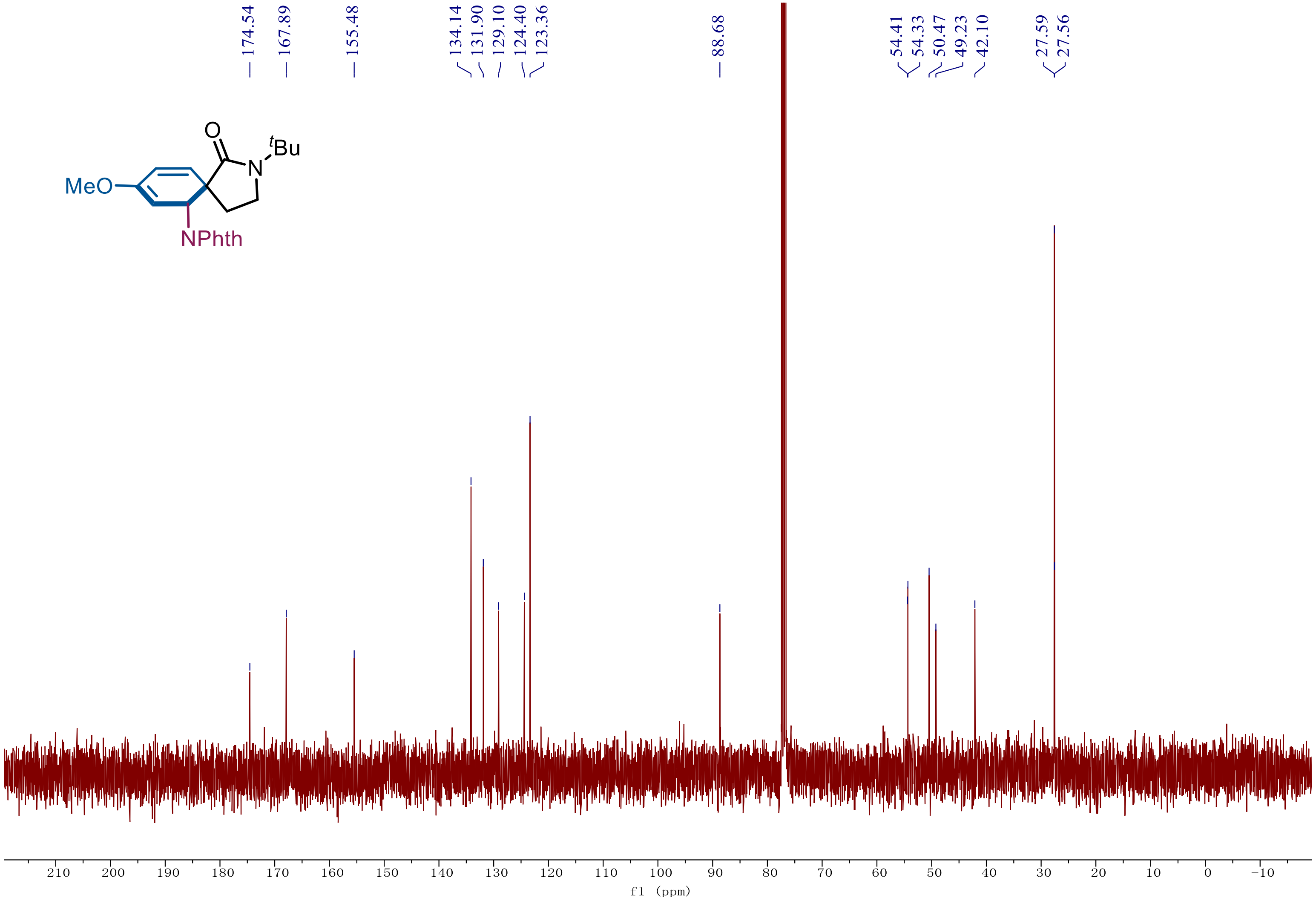

^{1}H NMR (400 MHz, $CDCl_3$) of **2ac**

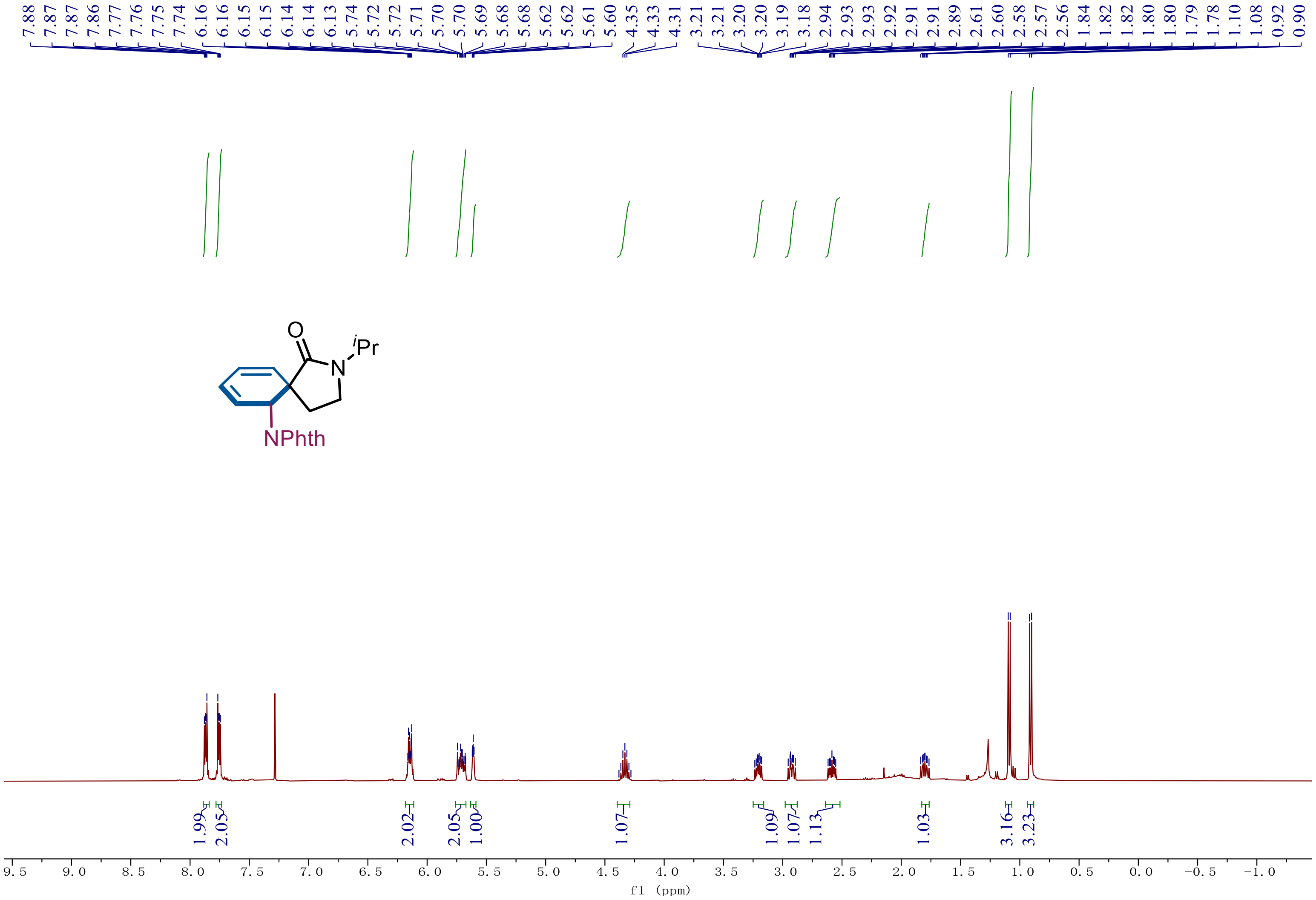

^{13}C NMR (101 MHz, CDCl$_3$) of **2ac**

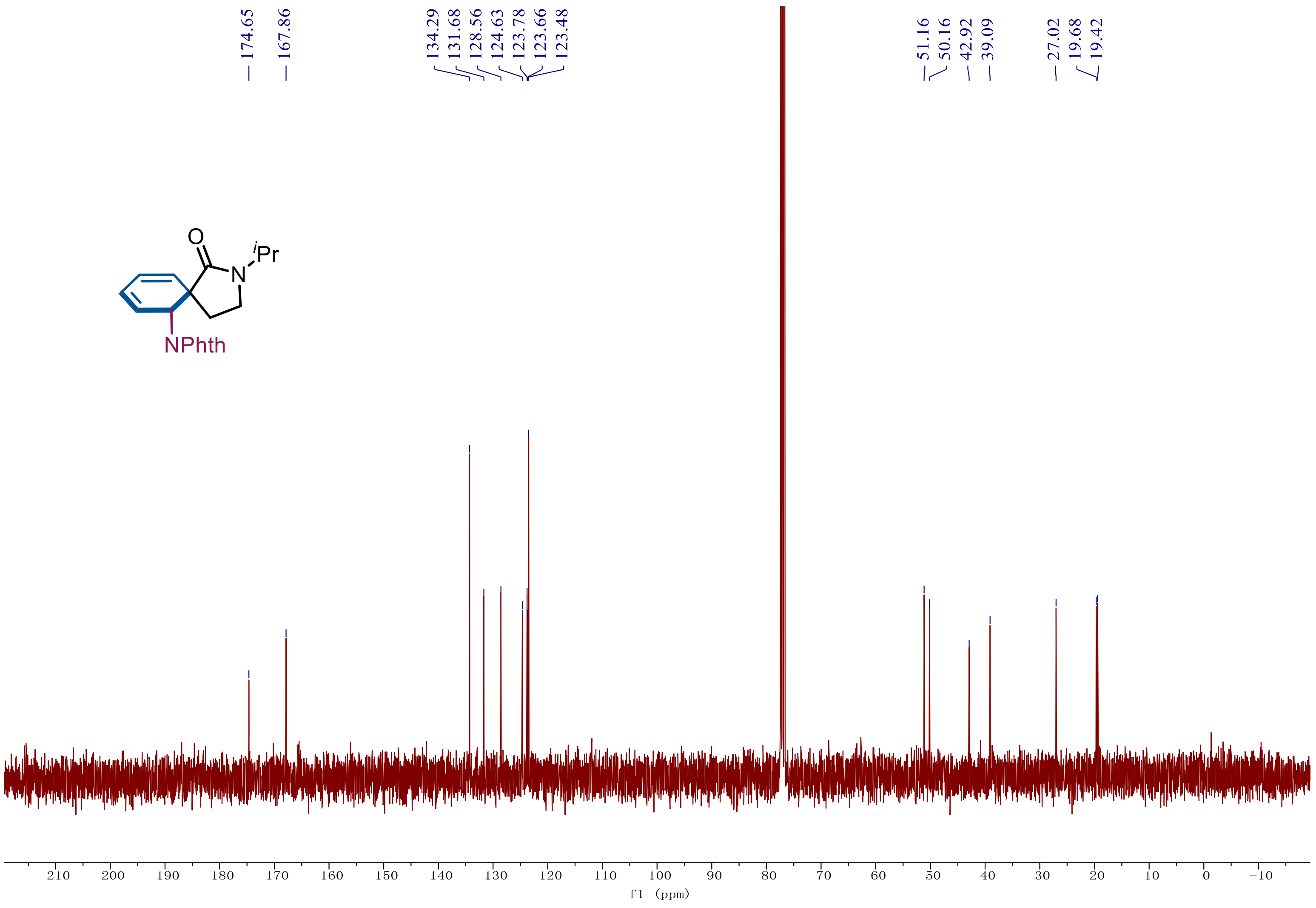

^{1}H NMR (400 MHz, $CDCl_3$) of **2ad**

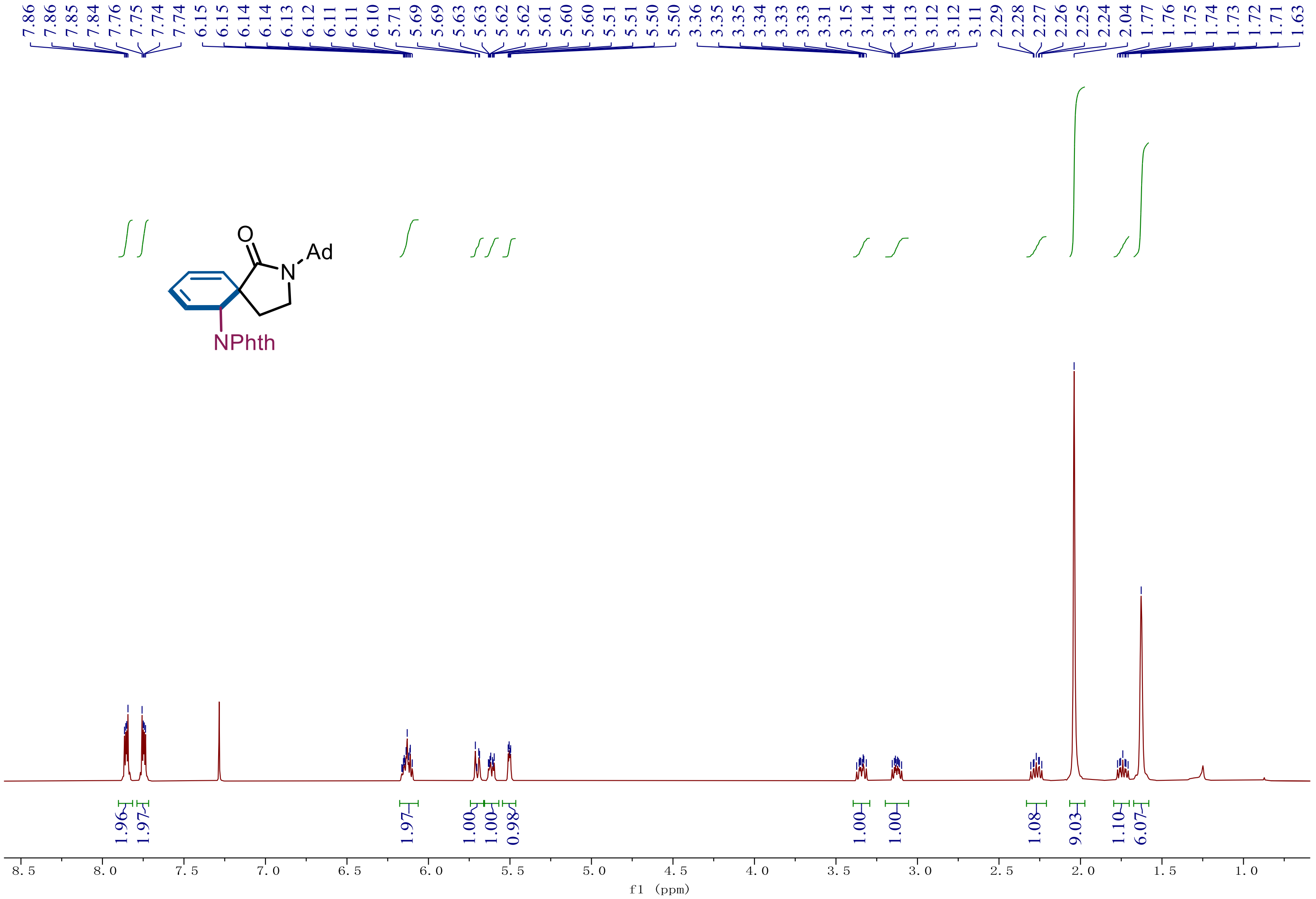

^{13}C NMR (101 MHz, $CDCl_3$) of **2ad**

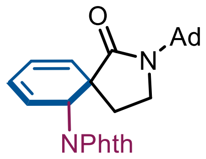

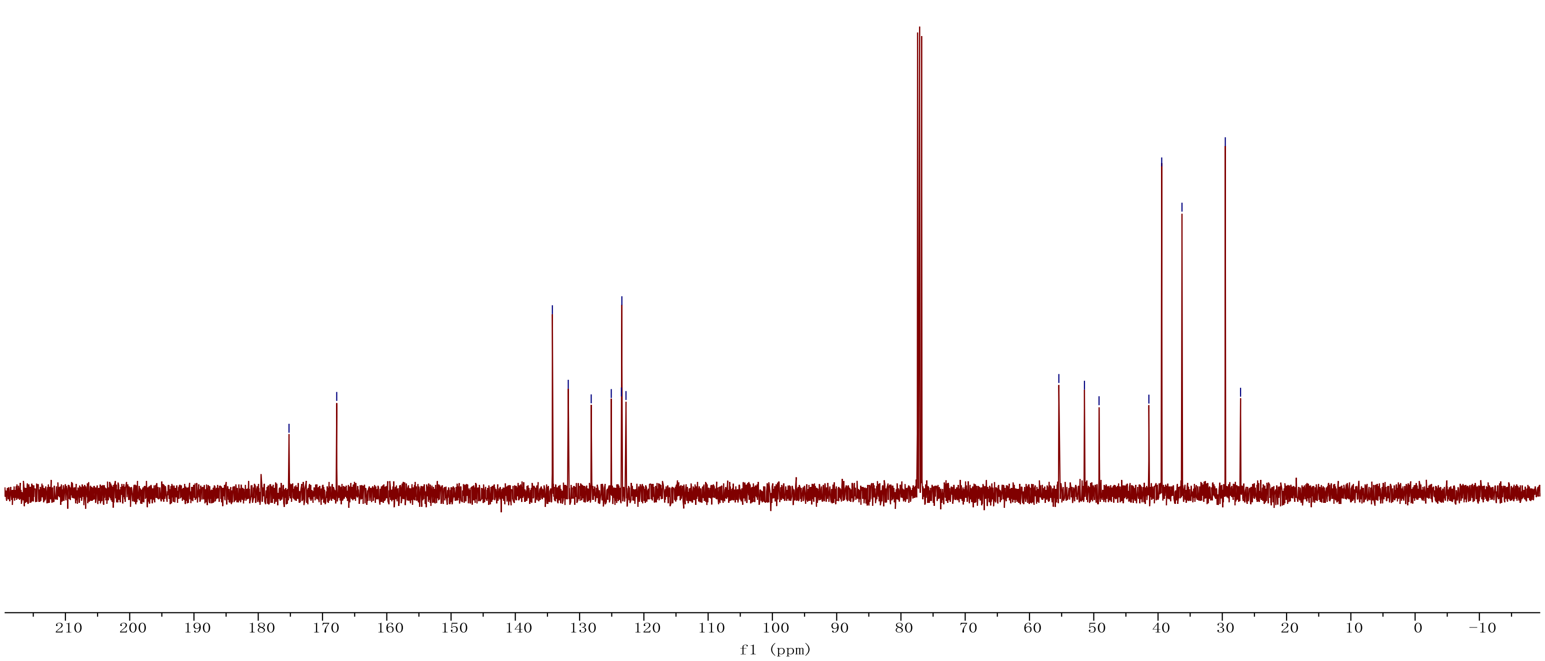

^{1}H NMR (400 MHz, $CDCl_3$) of **2ae**

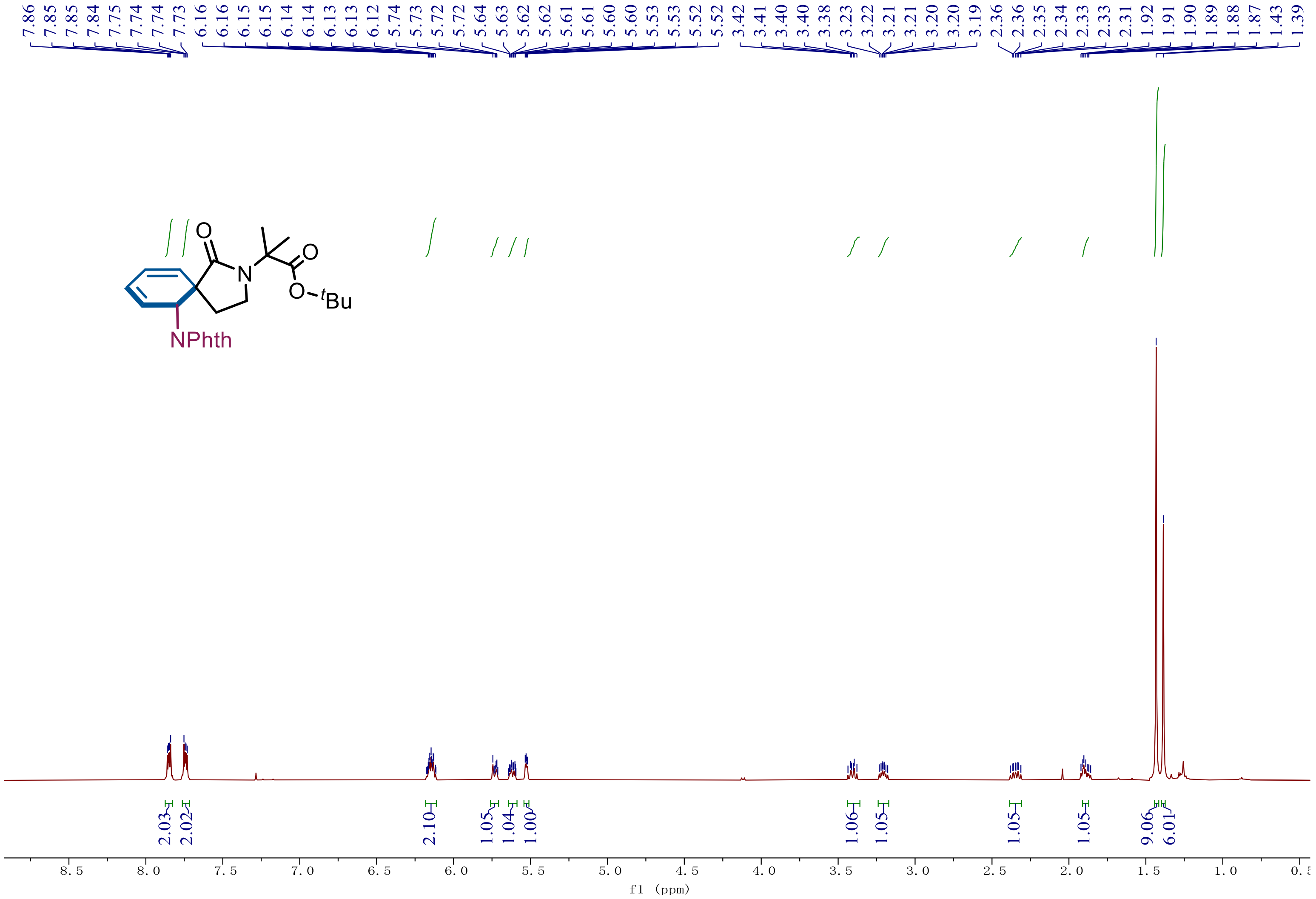

^{13}C NMR (101 MHz, $CDCl_3$) of **2ae**

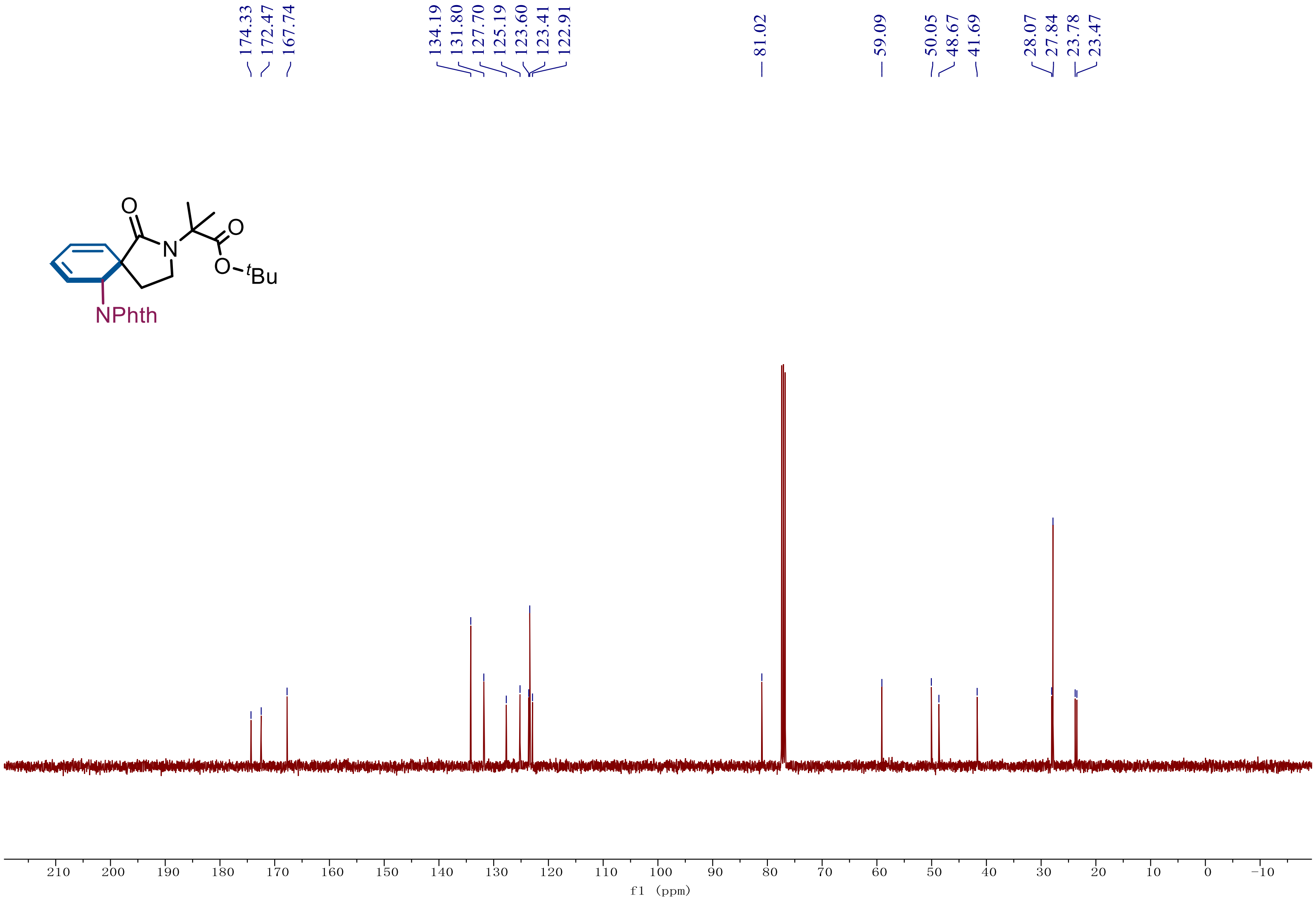
174.33
172.47
167.74
134.19
131.80
127.70
125.19
123.60
123.41
122.91
81.02
59.09
50.05
48.67
41.69
28.07
27.84
23.78
23.47
O
N
O
O
tBu
NPhth
210 200 190 180 170 160 150 140 130 120 110 100 90 80 70 60 50 40 30 20 10 0 -10
f1 (ppm)

^{1}H NMR (400 MHz, $CDCl_3$) of **2af-1**

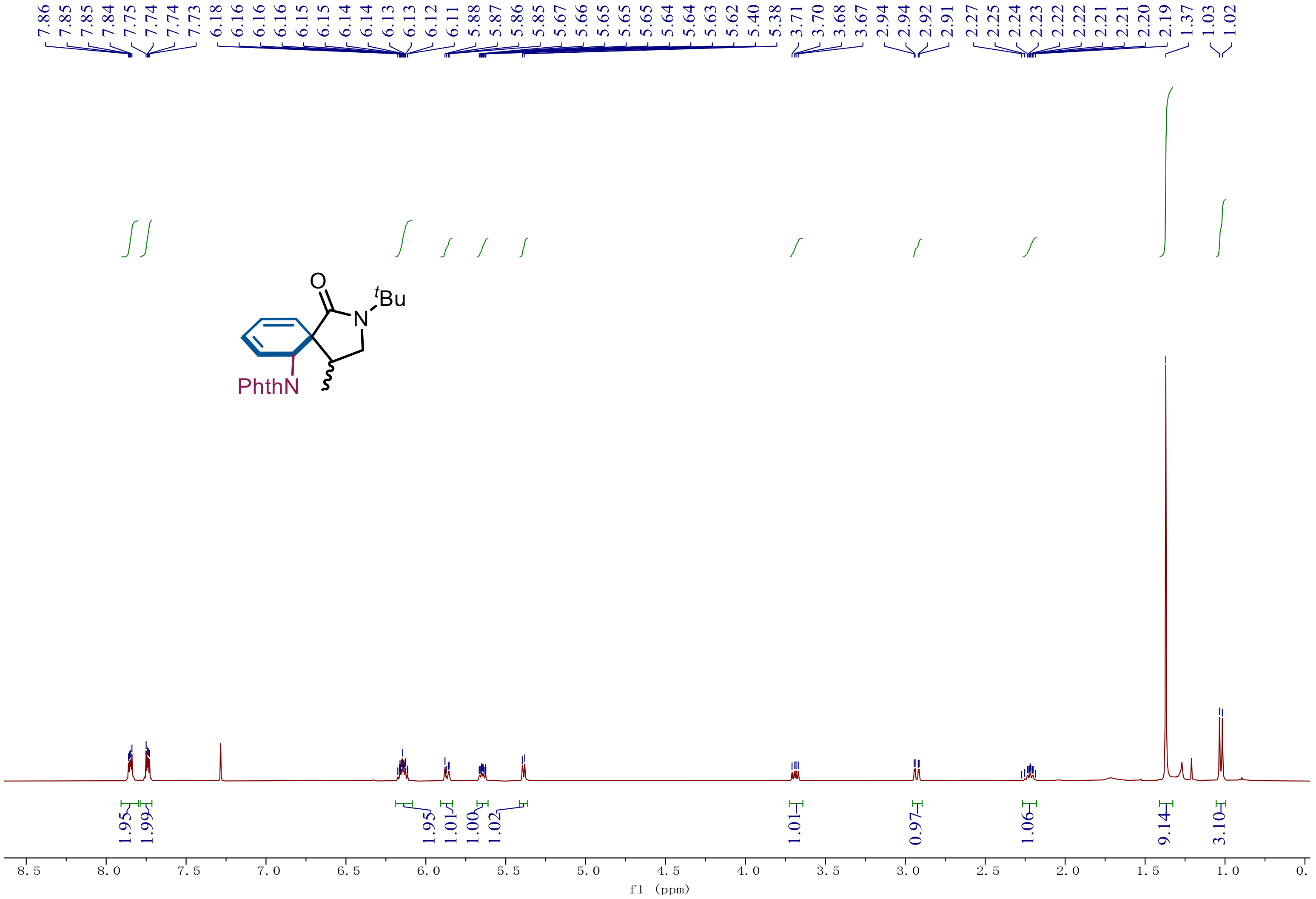

^{13}C NMR (101 MHz, $CDCl_3$) of **2af-1**

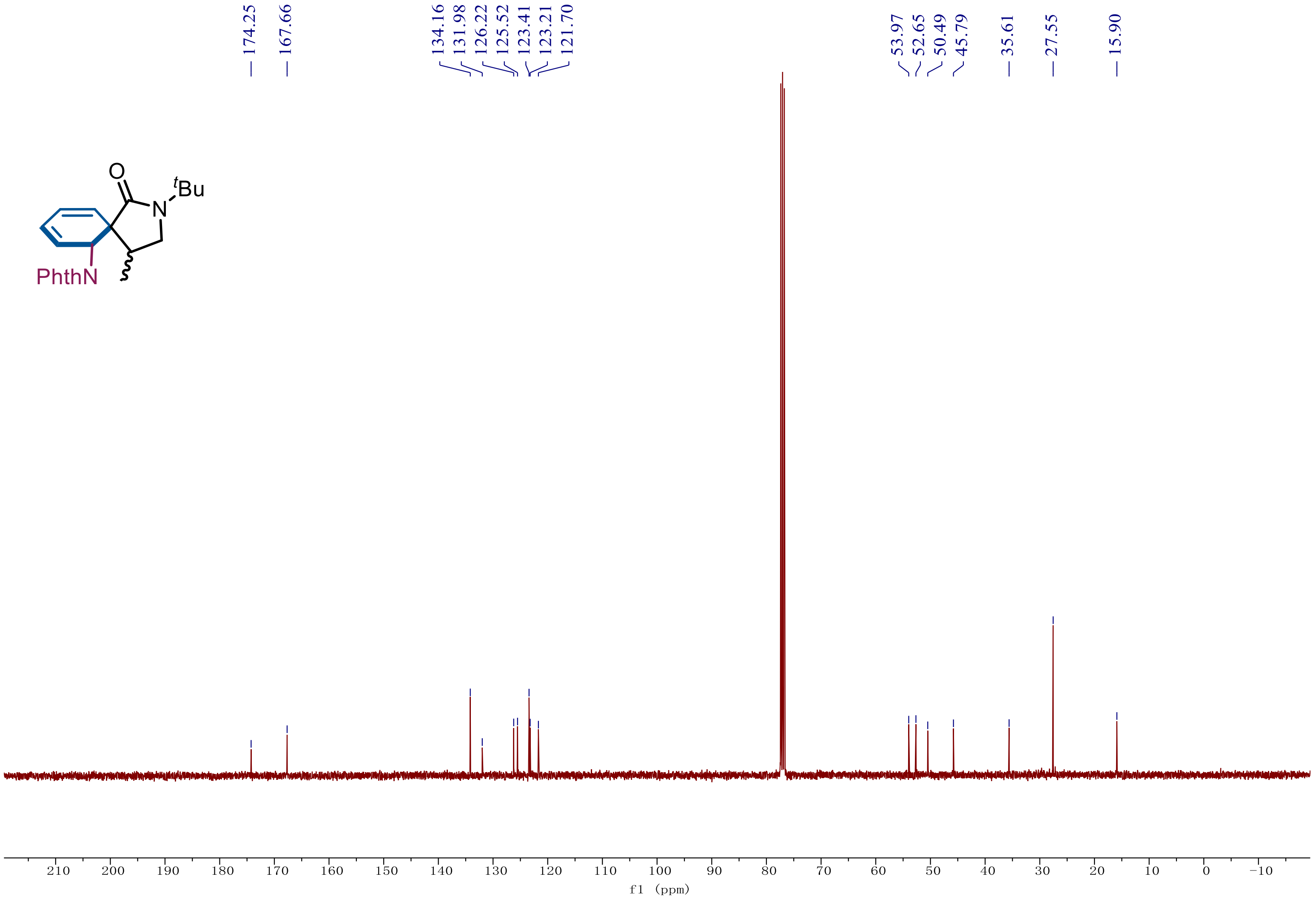

NOE of **2af-1**

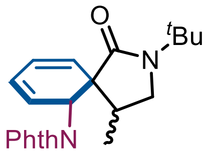
O
tBu
N
PhthN

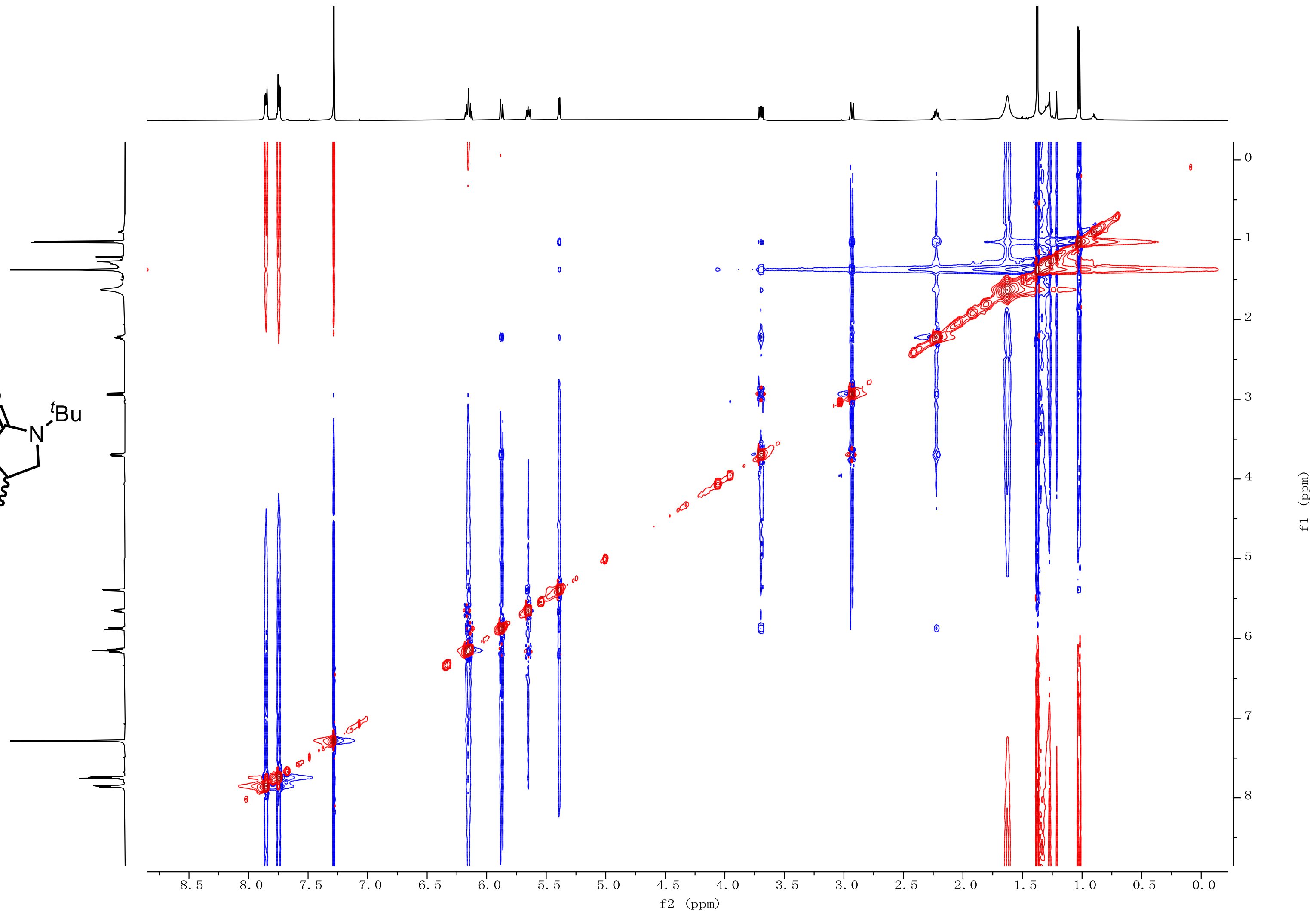
8.5
8.0
7.5
7.0
6.5
6.0
5.5
5.0
4.5
4.0
3.5
3.0
2.5
2.0
1.5
1.0
0.5
0.0
f2 (ppm)
0
1
2
3
4
5
6
7
8
f1 (ppm)

^{1}H NMR (400 MHz, $CDCl_3$) of **2af-2**

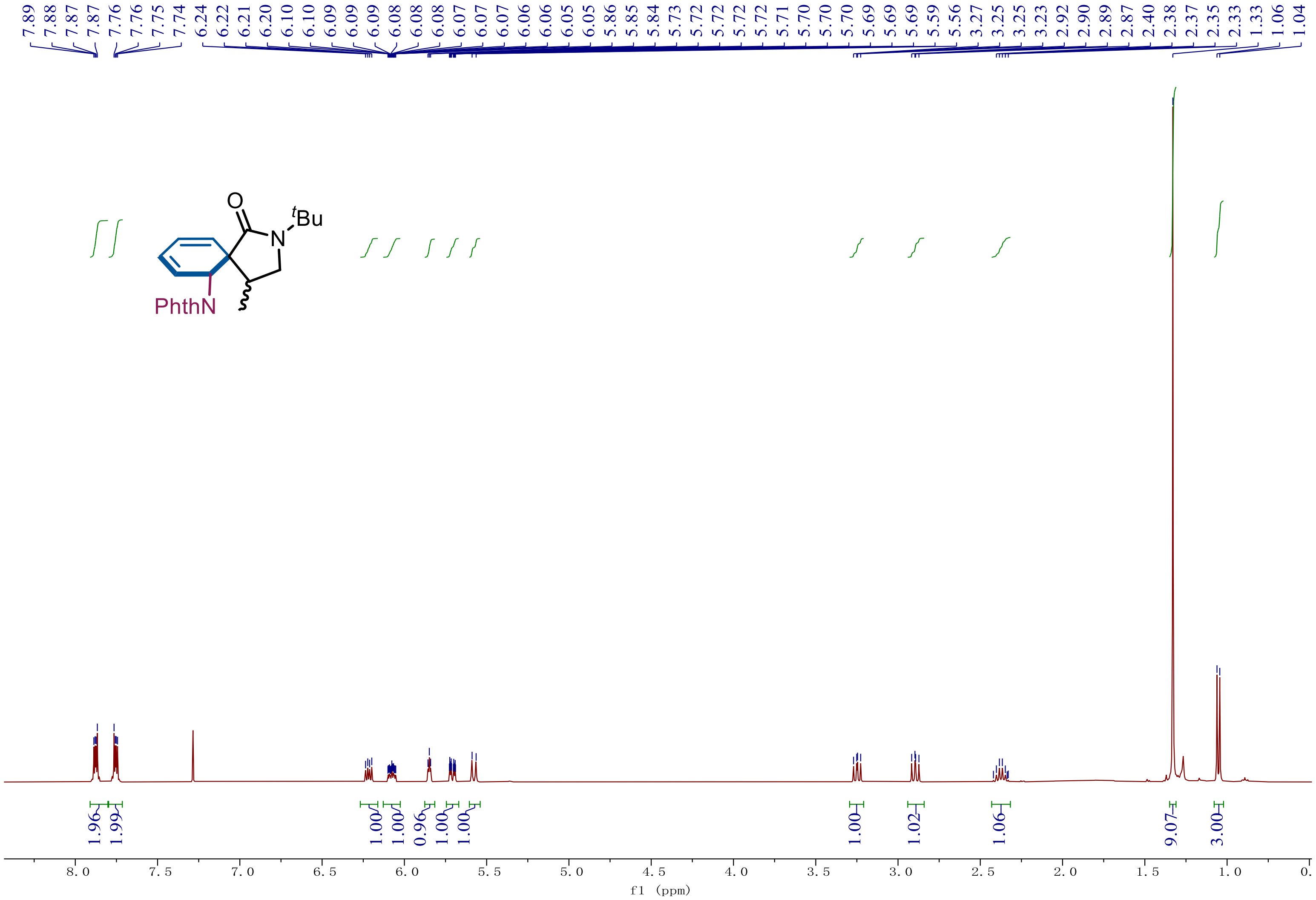

^{13}C NMR (101 MHz, $CDCl_3$) of **2af-2**

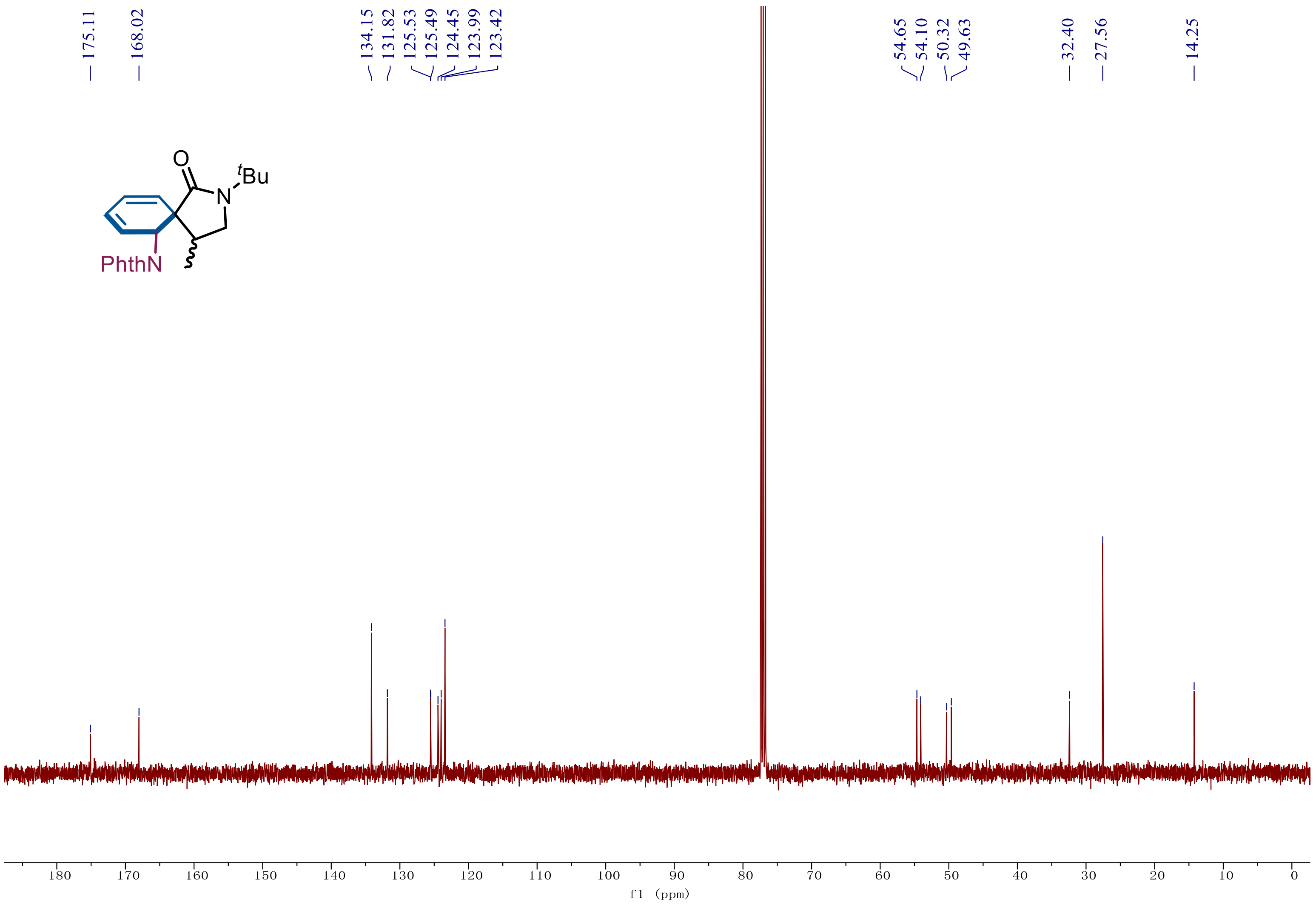

NOE of **2af-2**

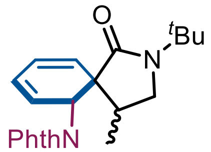

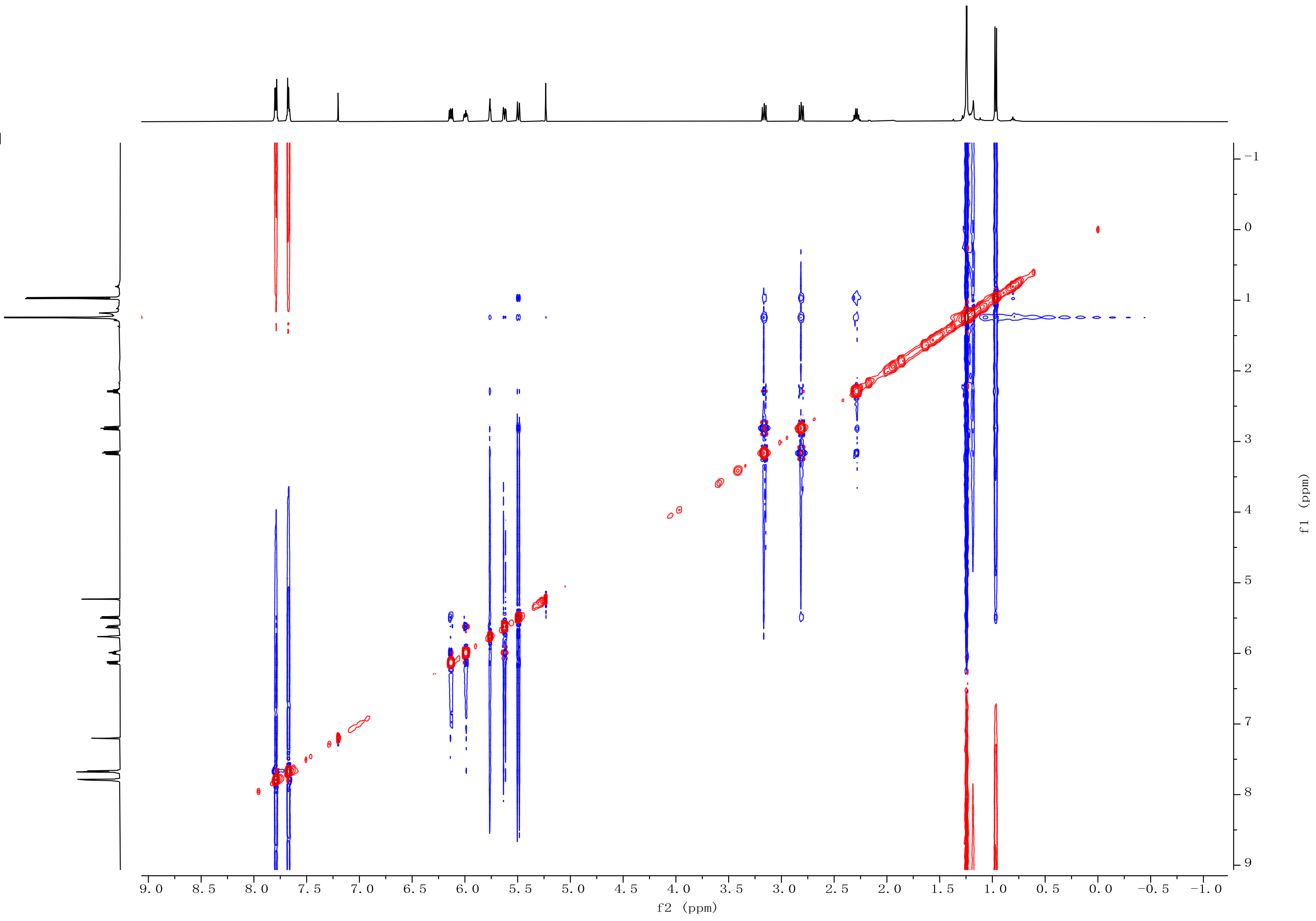

^{1}H NMR (400 MHz, $CDCl_3$) of **2ag**

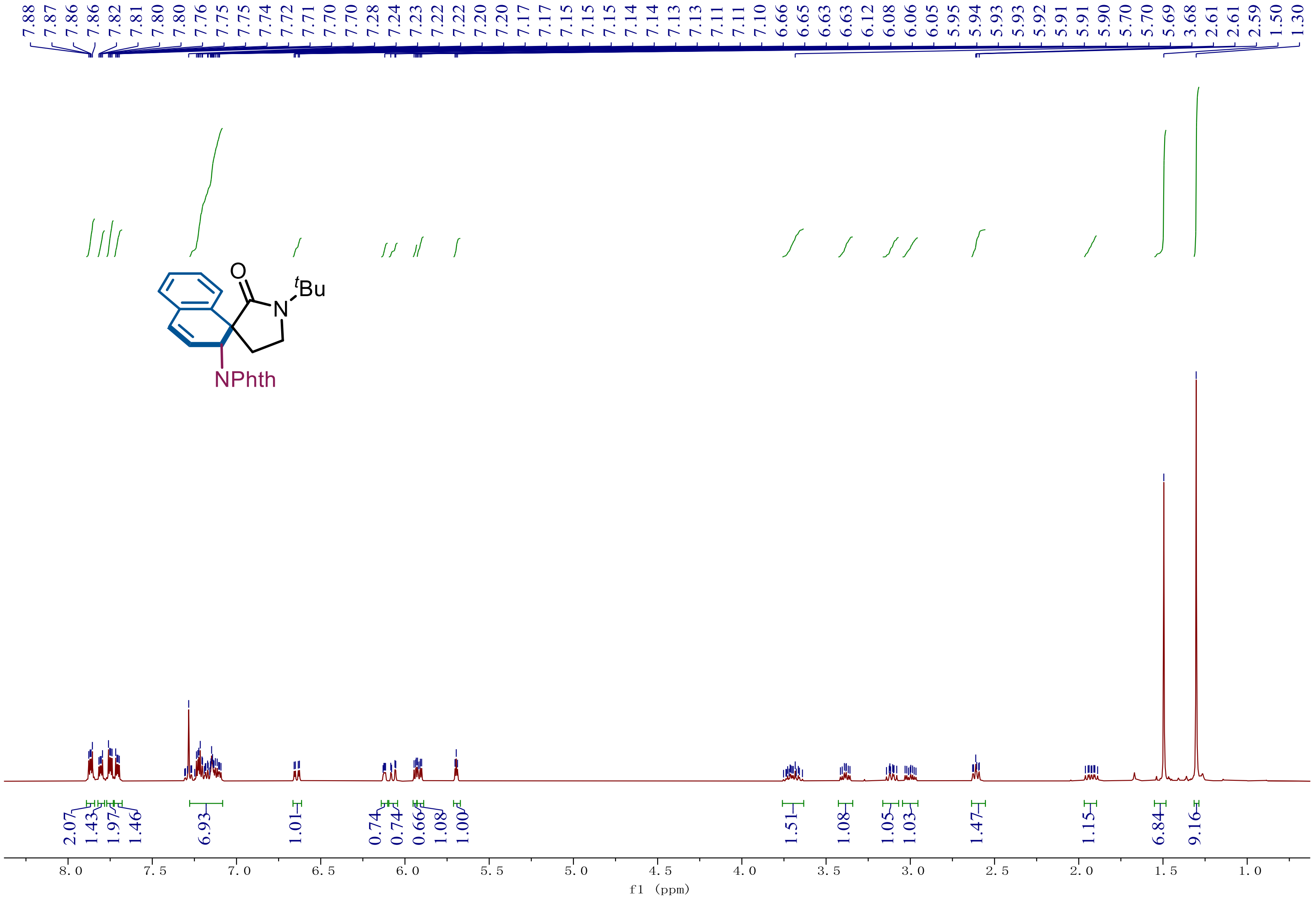

^{13}C NMR (101 MHz, $CDCl_3$) of **2ag**

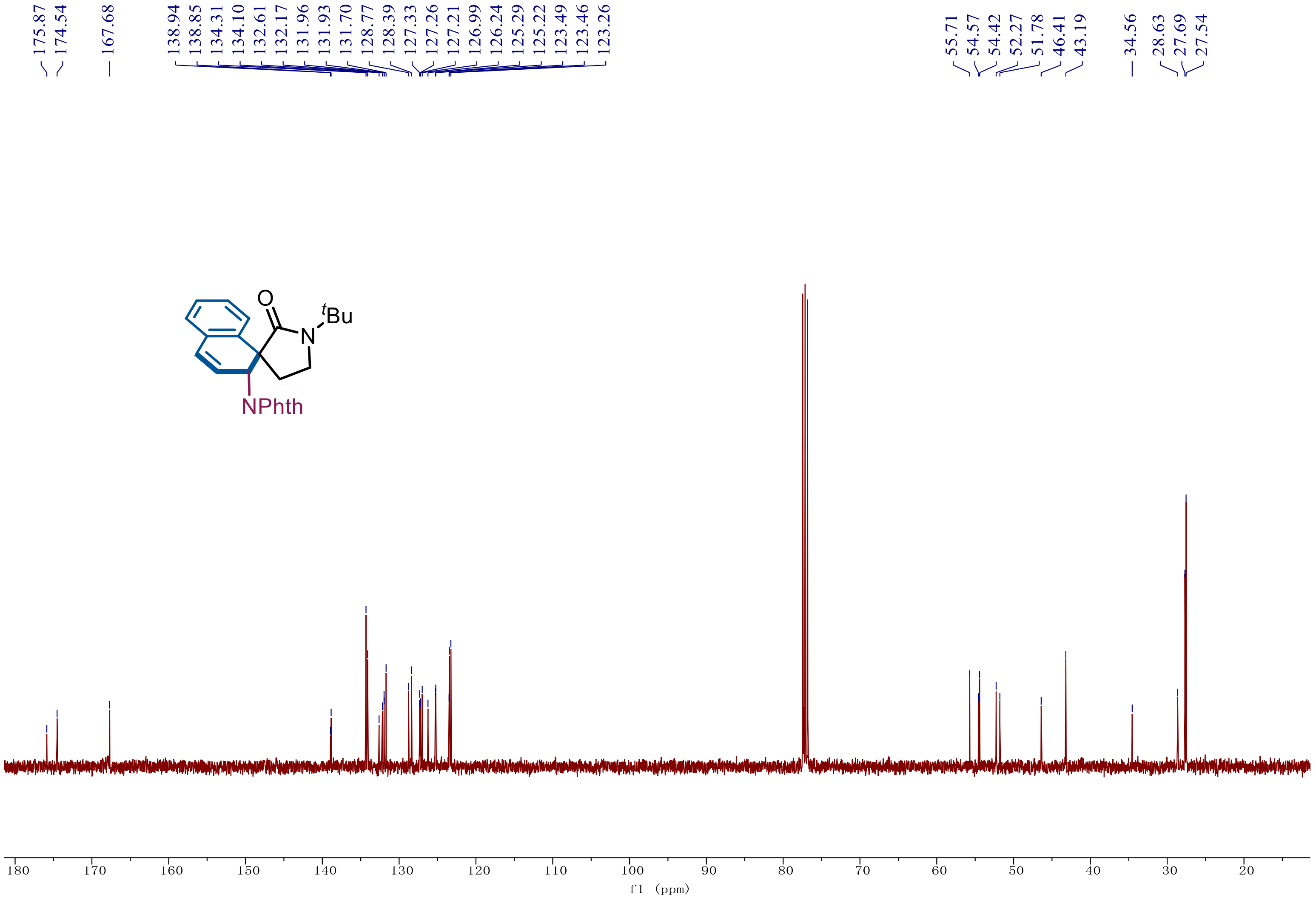

^{1}H NMR (400 MHz, $CDCl_3$) of **2ah**

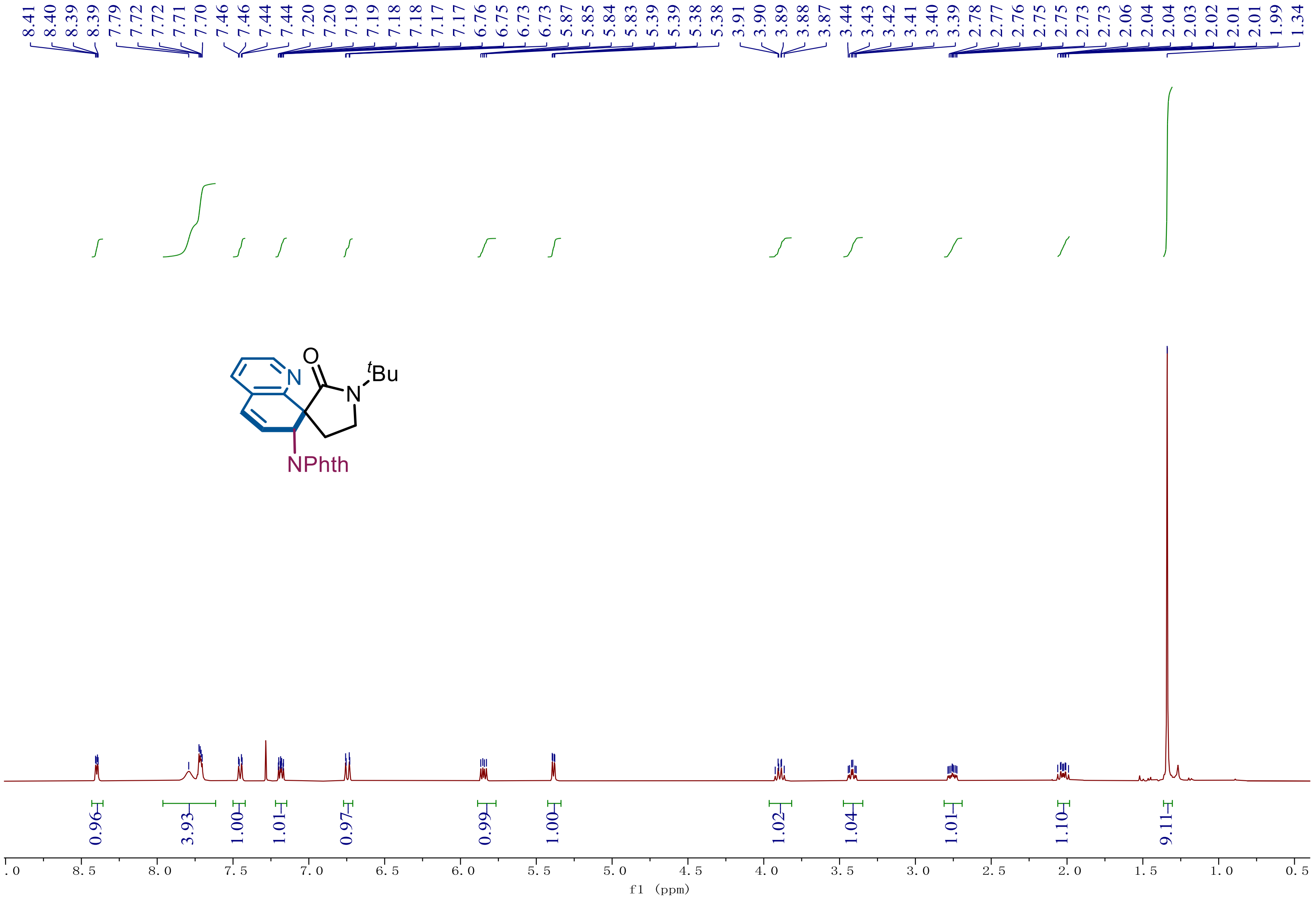

^{13}C NMR (101 MHz, $CDCl_3$) of **2ah**

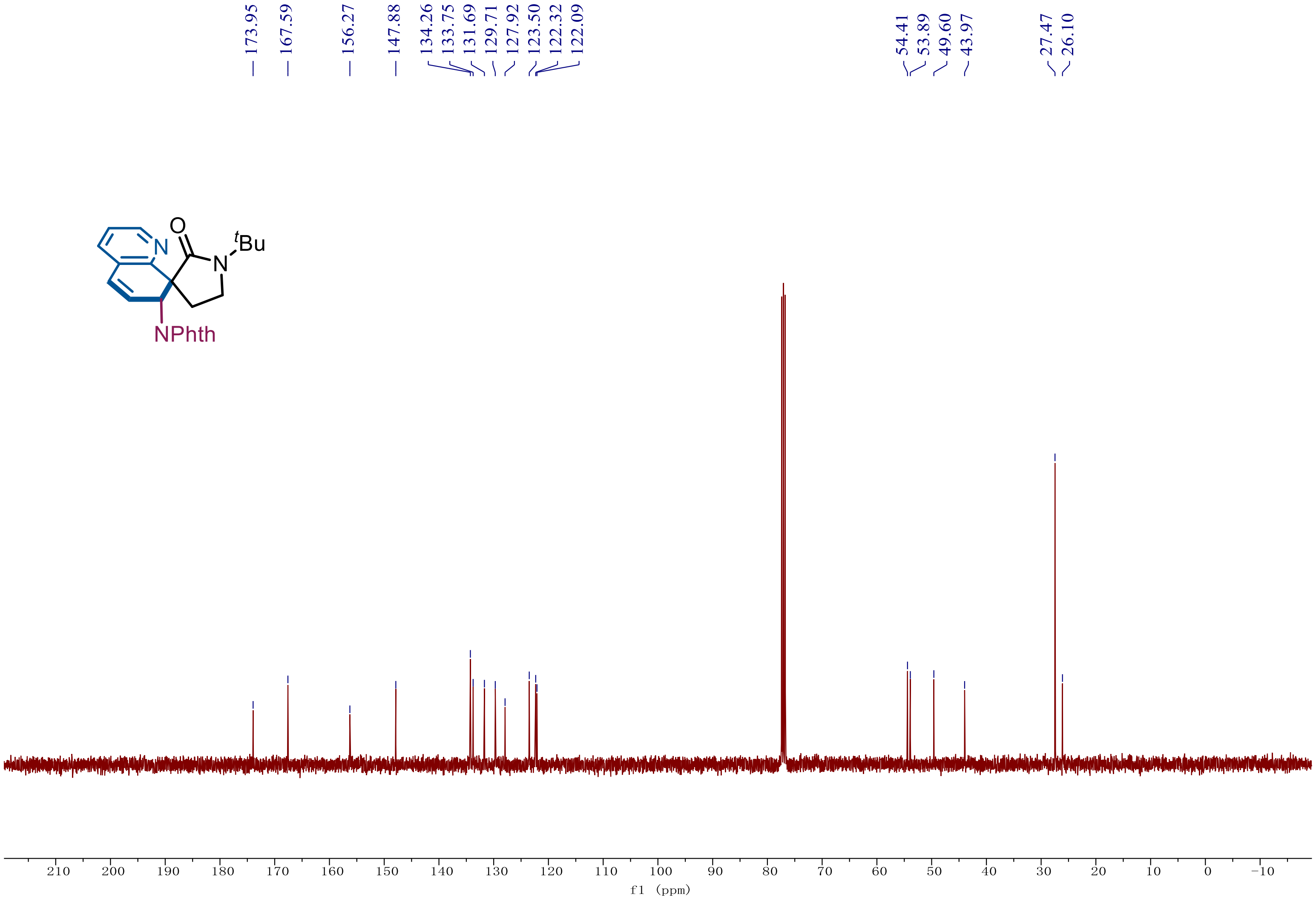

^1H NMR (400 MHz, $CDCl_3$) of **2ai**

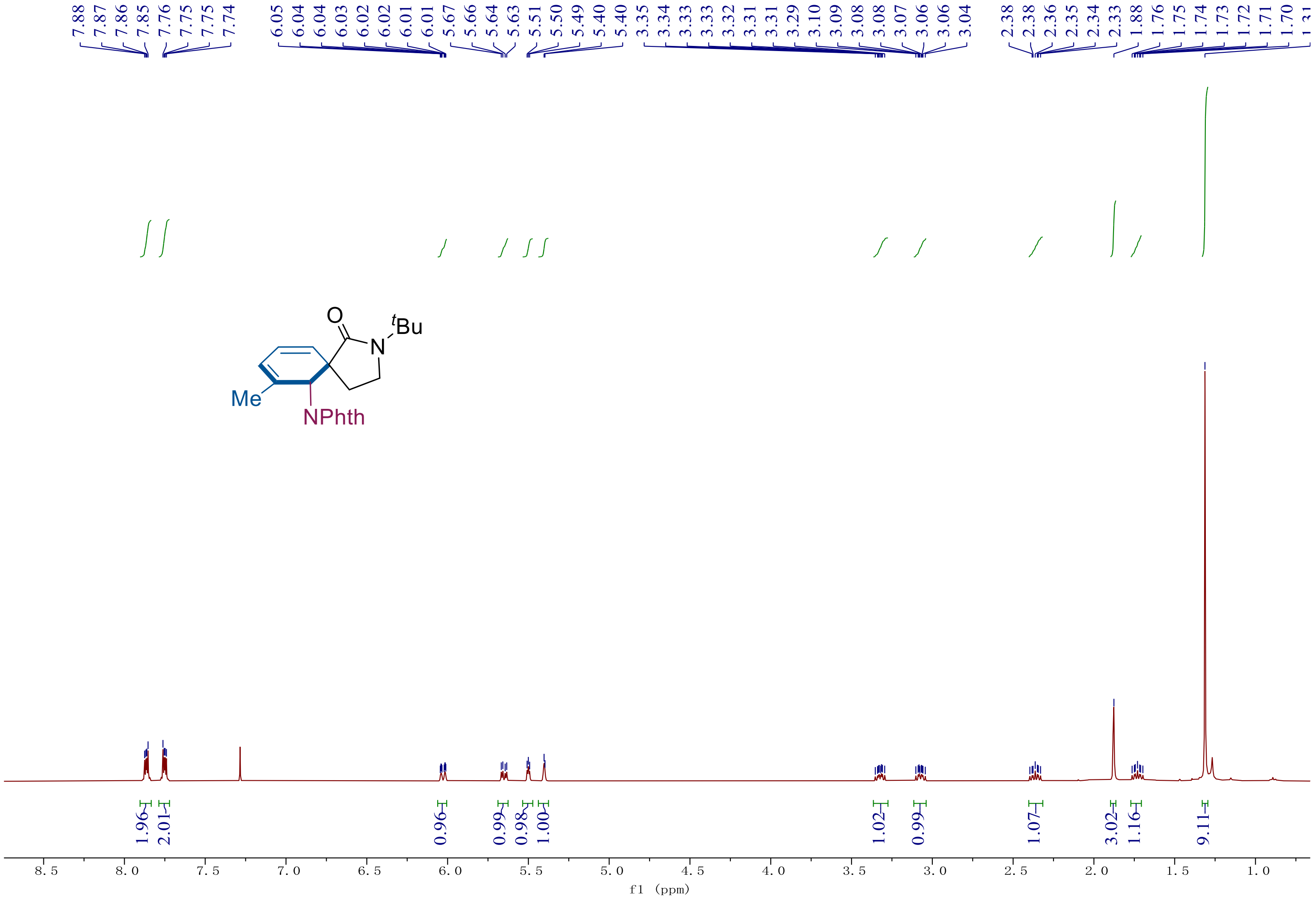

^{13}C NMR (101 MHz, $CDCl_3$) of **2ai**

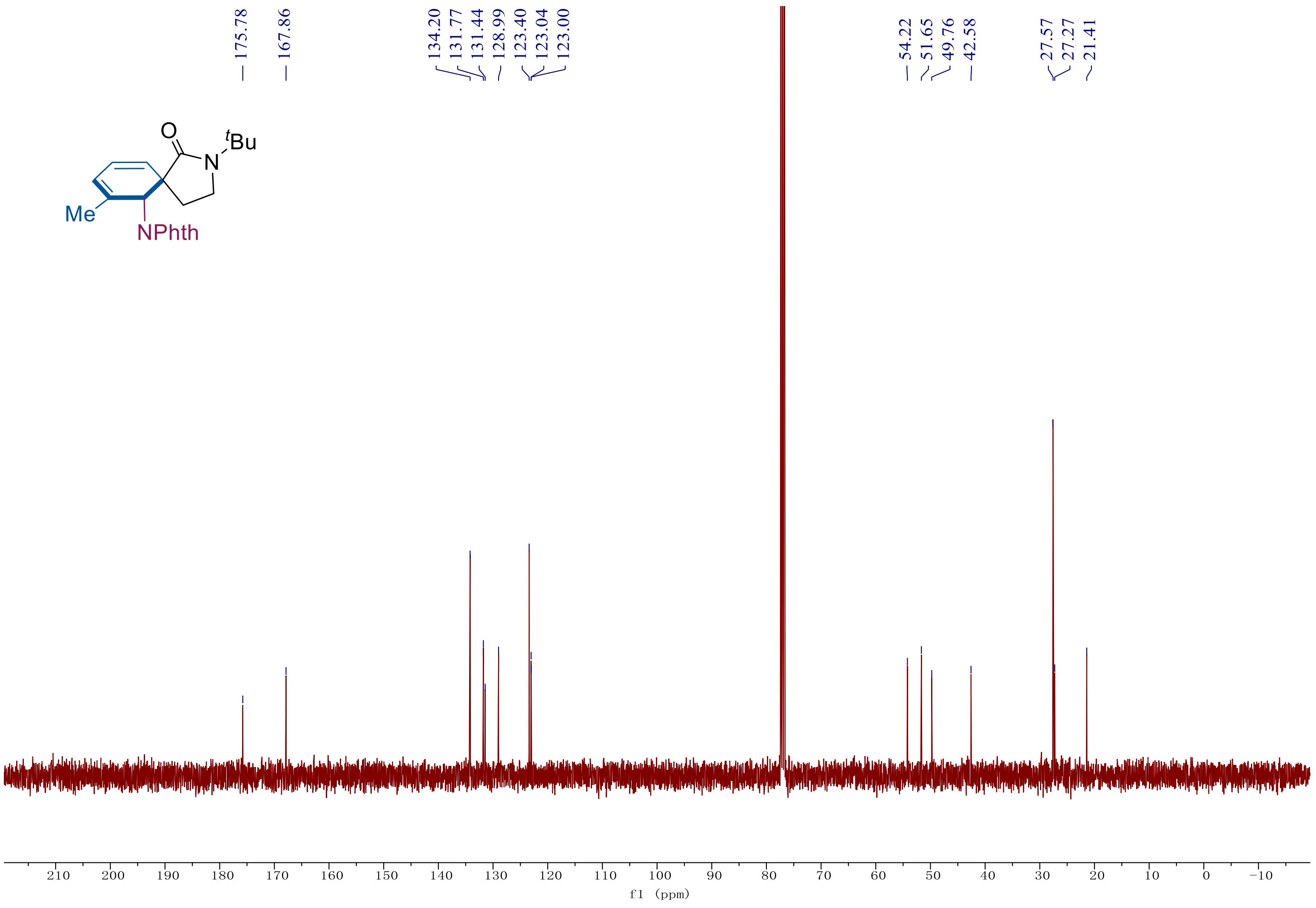

HMBC NMR (^{1}H NMR 400 MHz, ^{13}C 101 MHz, $CDCl_3$) of **2ai**

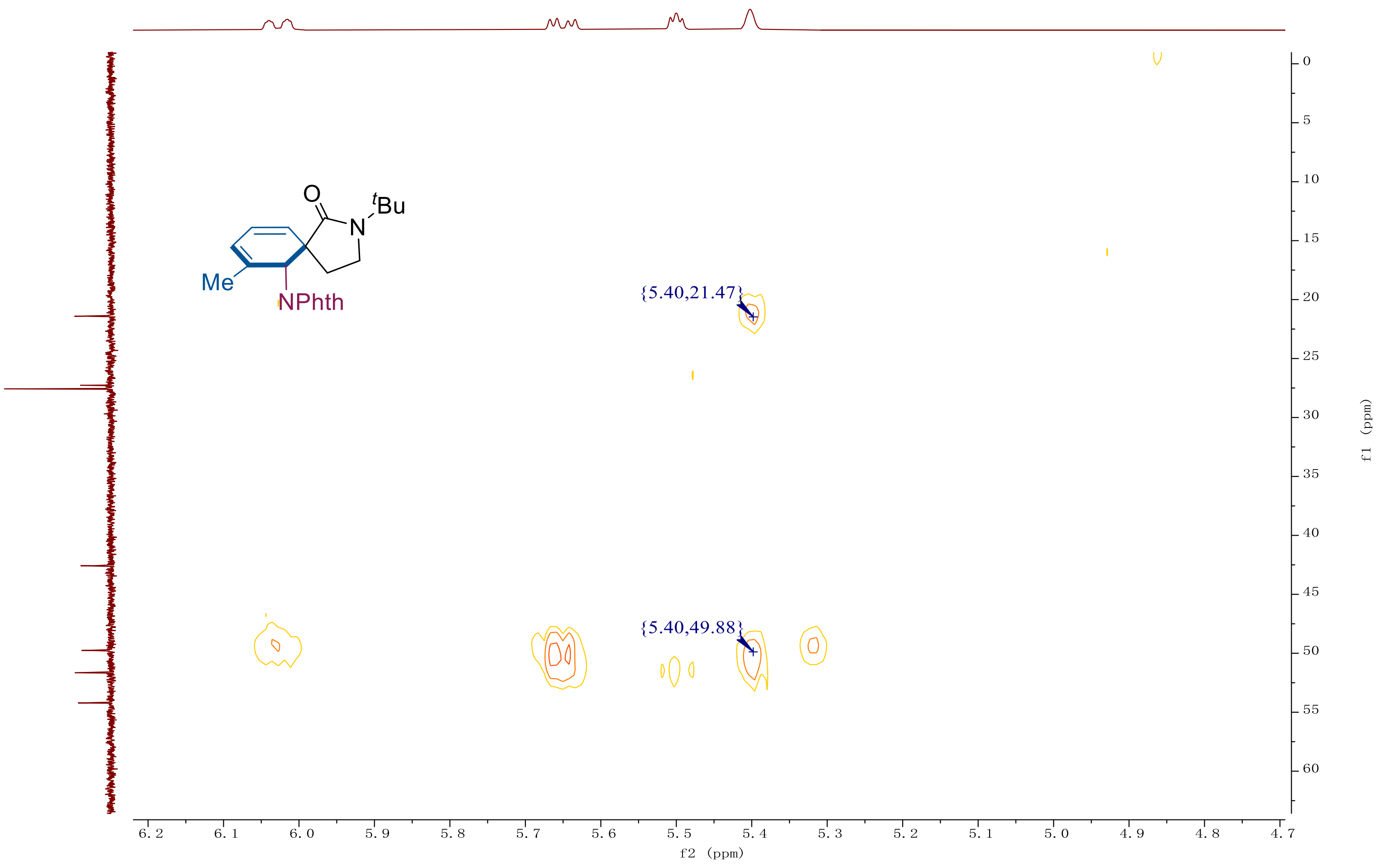

^{1}H NMR (400 MHz, $CDCl_3$) of **2ai’**

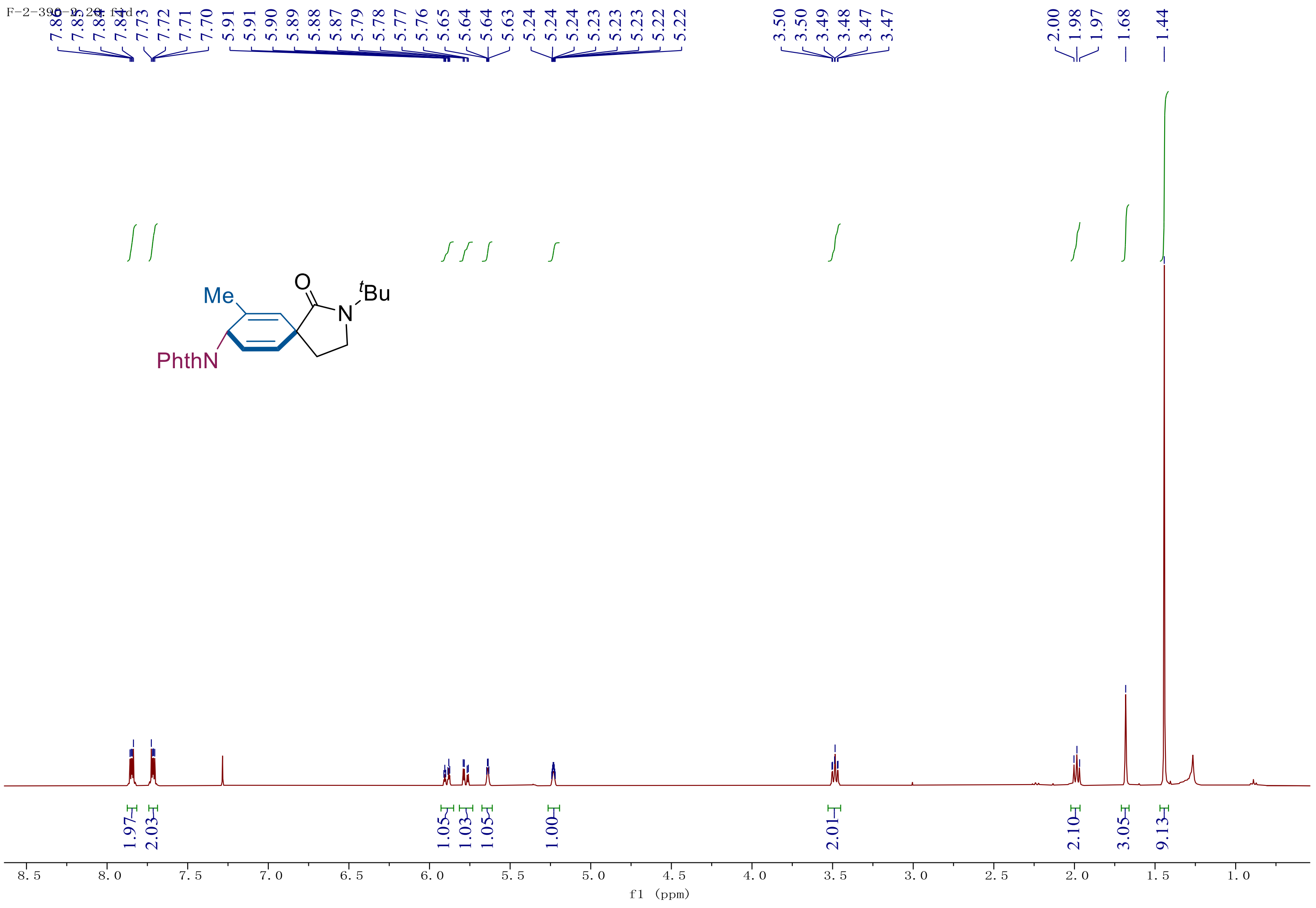

^{13}C NMR (101 MHz, CDCl$_3$) of **2ai'**

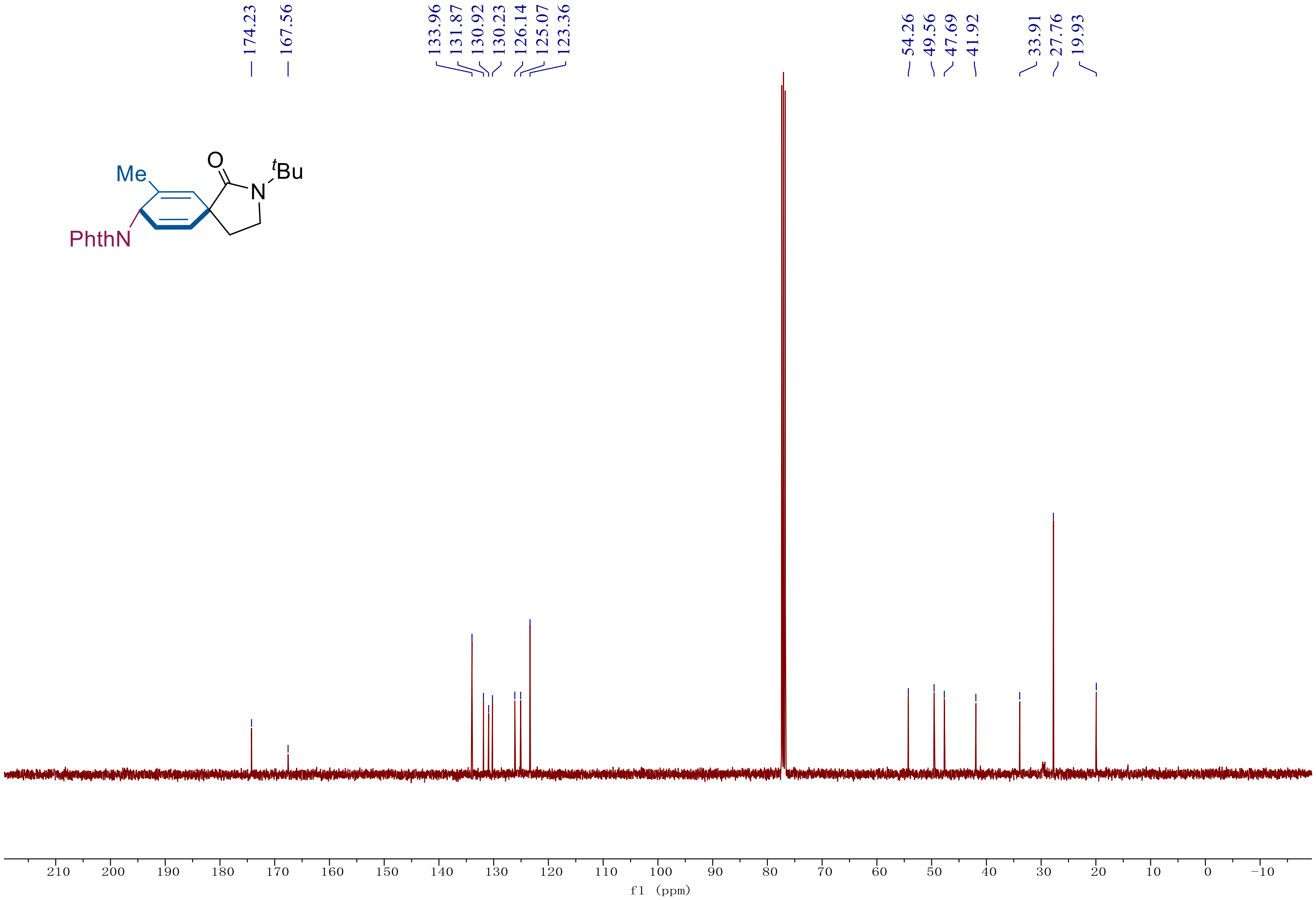

HMBC NMR (^{1}H NMR 400 MHz, ^{13}C 101 MHz, $CDCl_3$) of **2ai’**

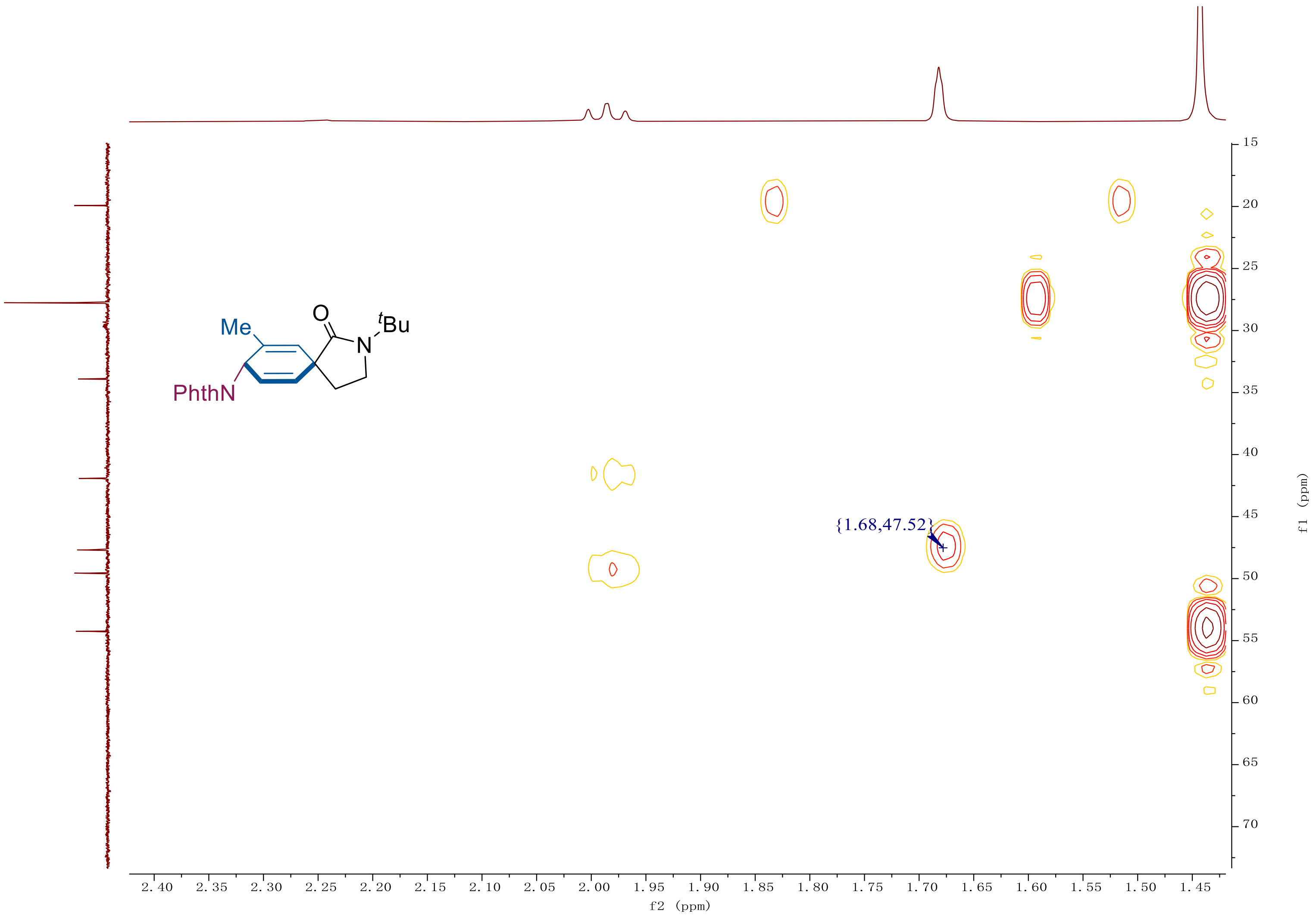

^{1}H NMR (400 MHz, $CDCl_3$) of **2aj**

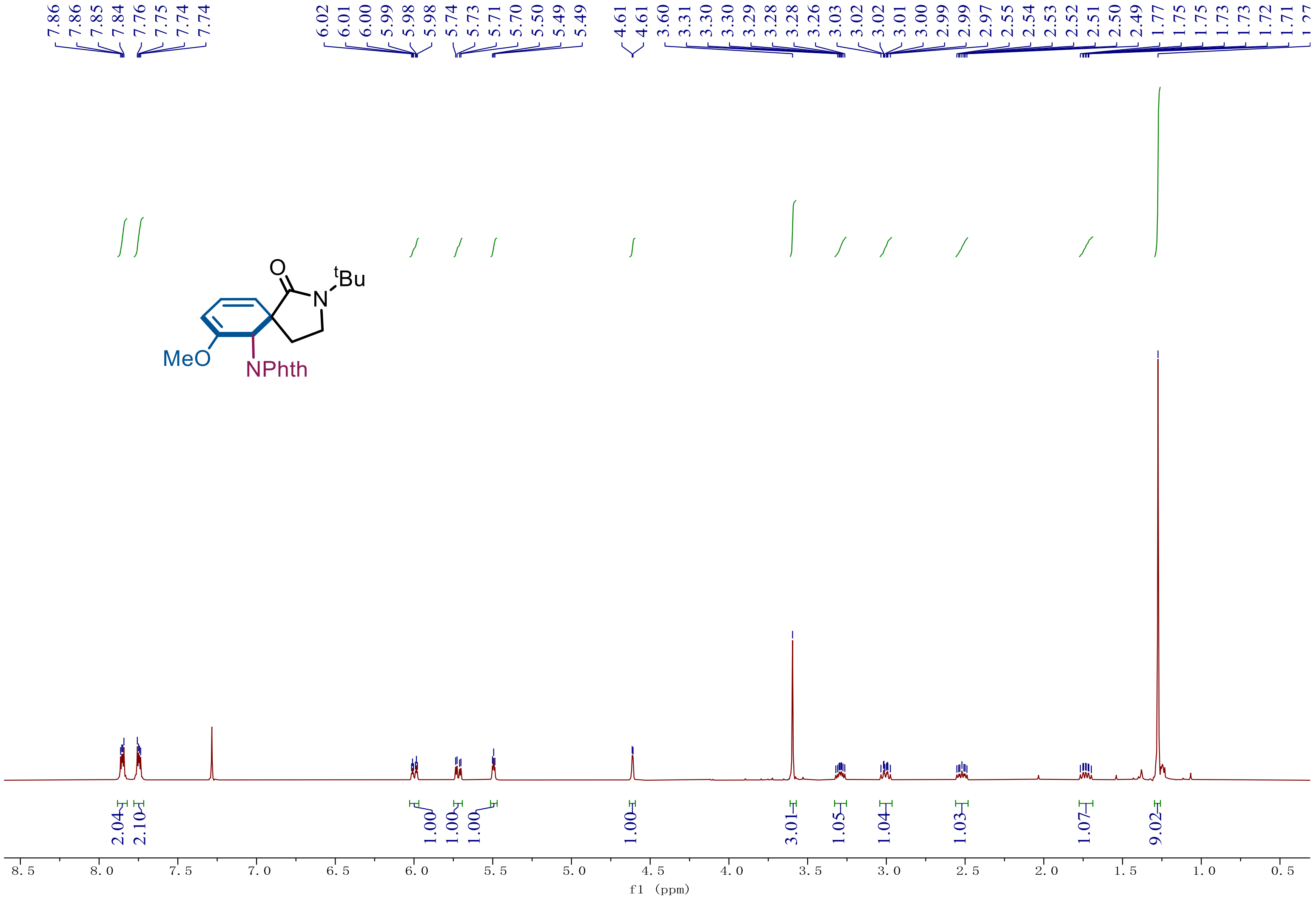

^{13}C NMR (101 MHz, CDCl$_3$) of **2aj**

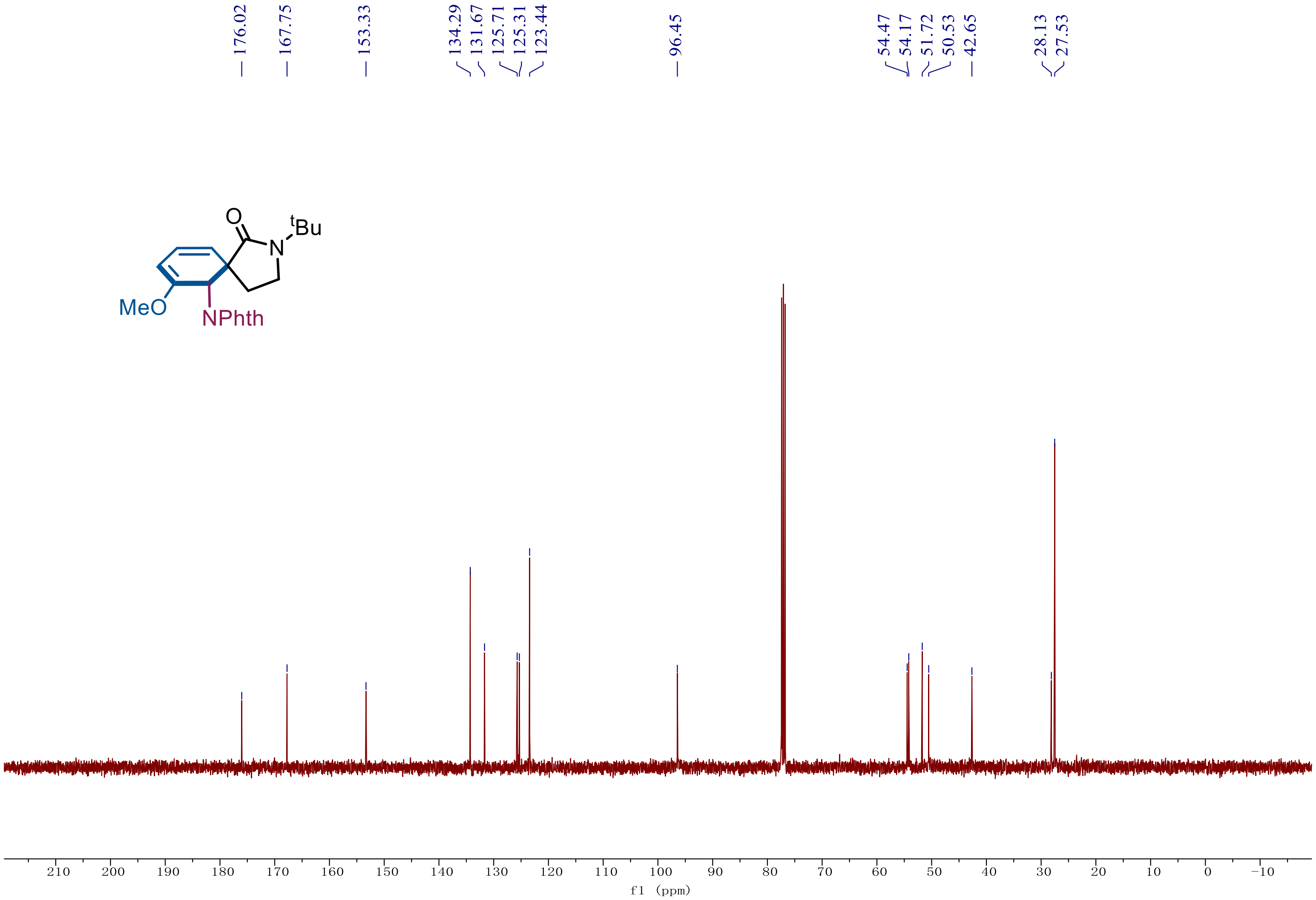

^{1}H NMR (400 MHz, CDCl3) of **2aj’**

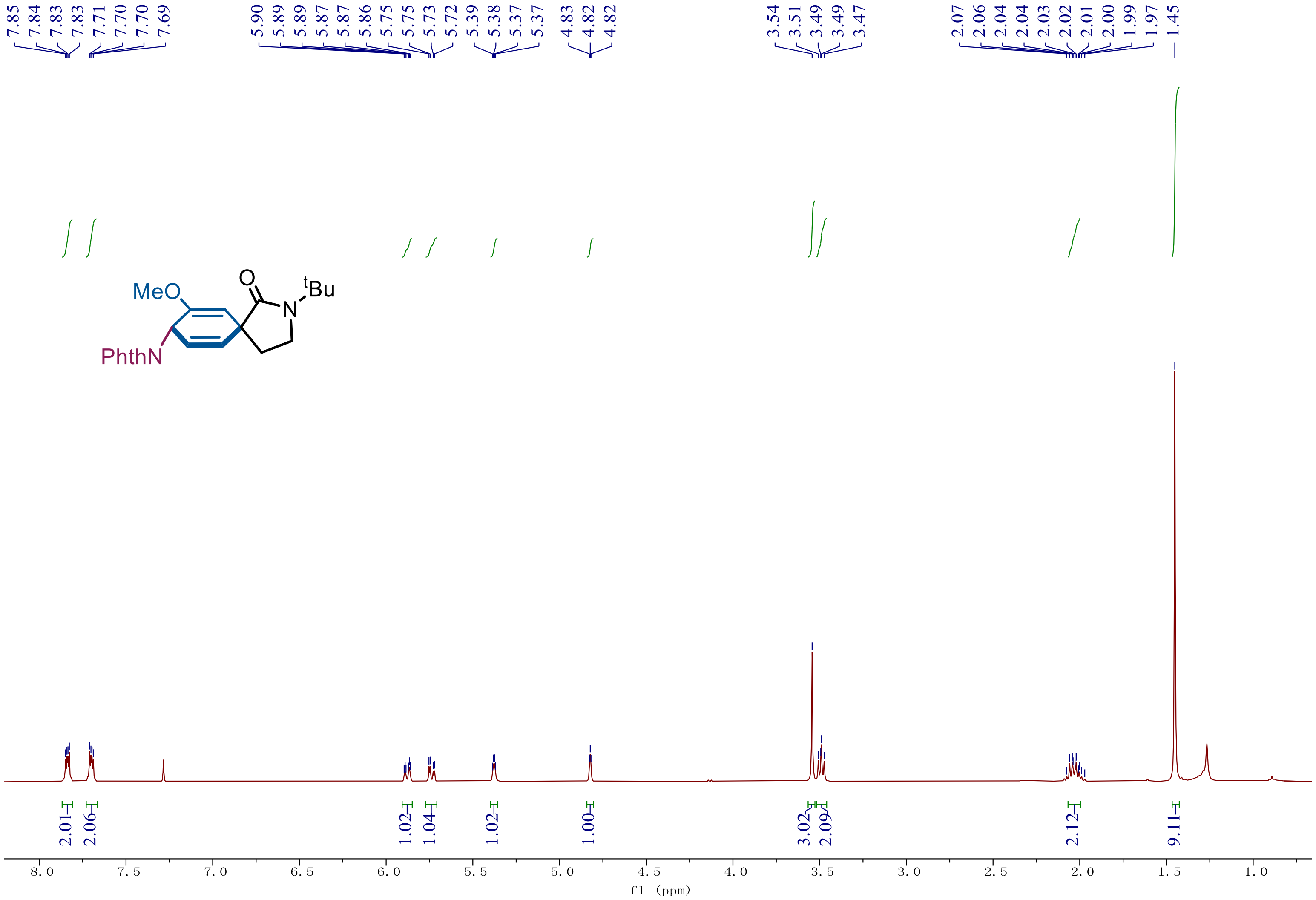

^{13}C NMR (101 MHz, $CDCl_3$) of **2aj’**

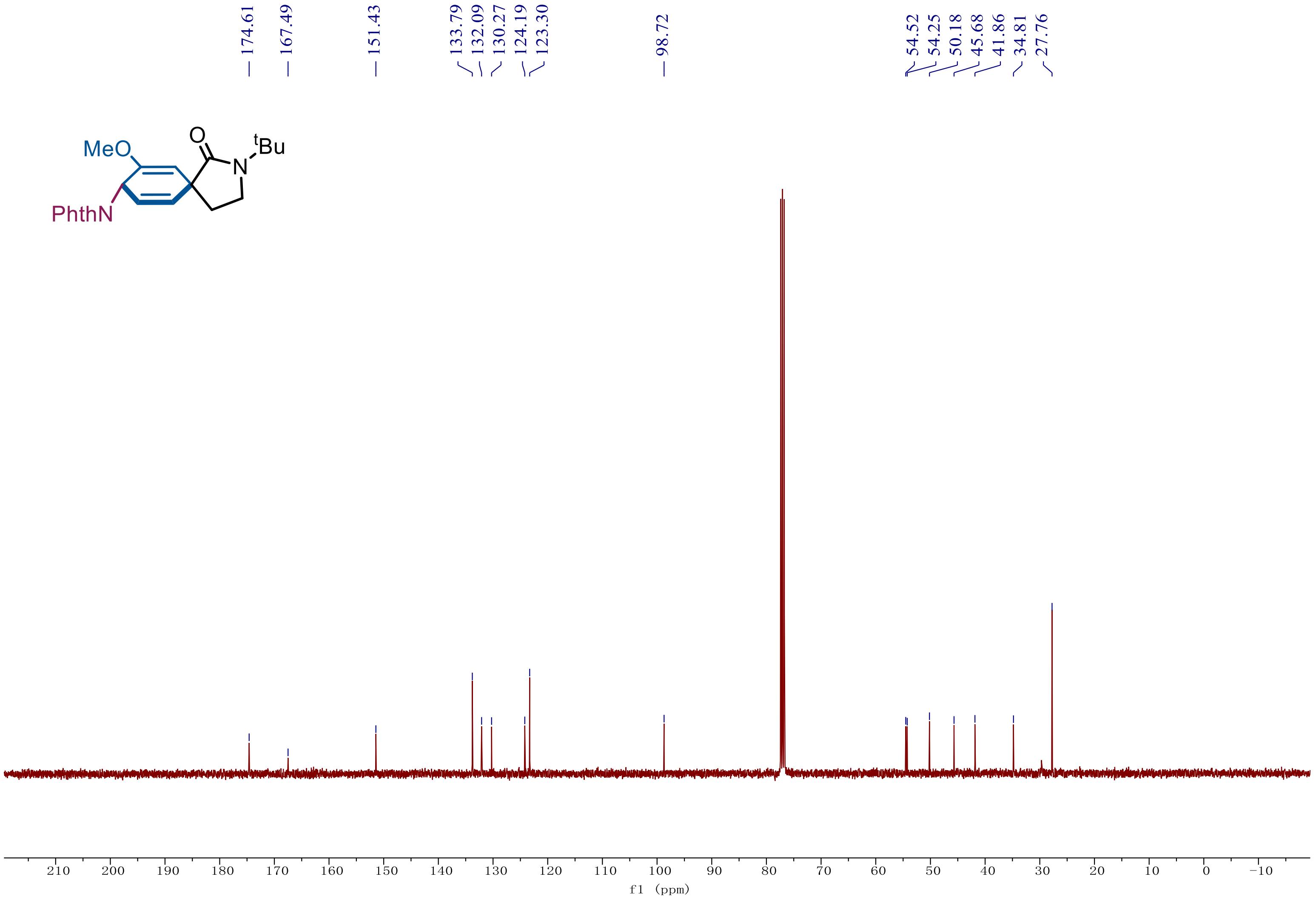

^{1}H NMR (400 MHz, $CDCl_3$) of **2ak**

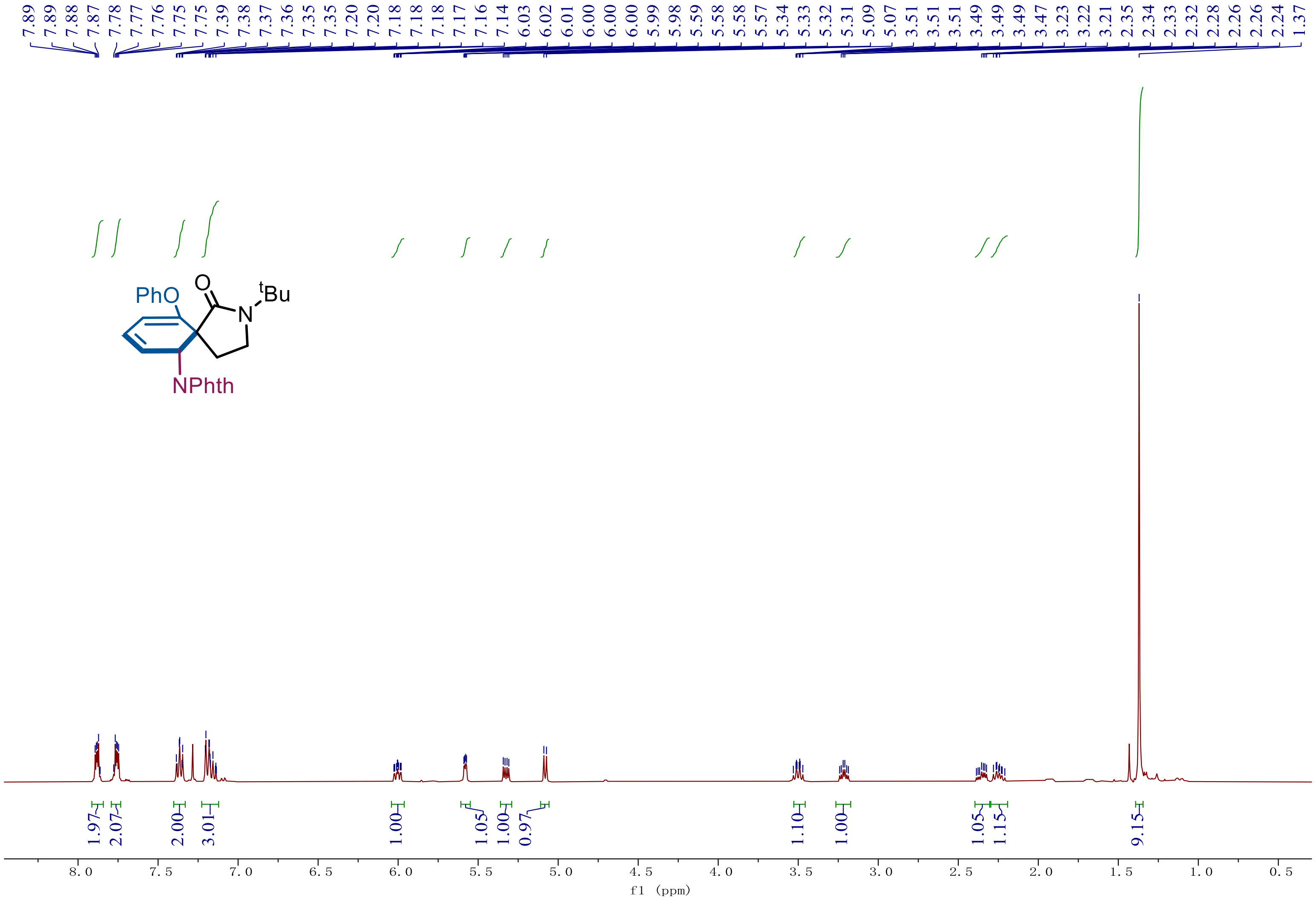

^{13}C NMR (101 MHz, $CDCl_3$) of **2ak**

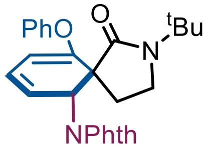

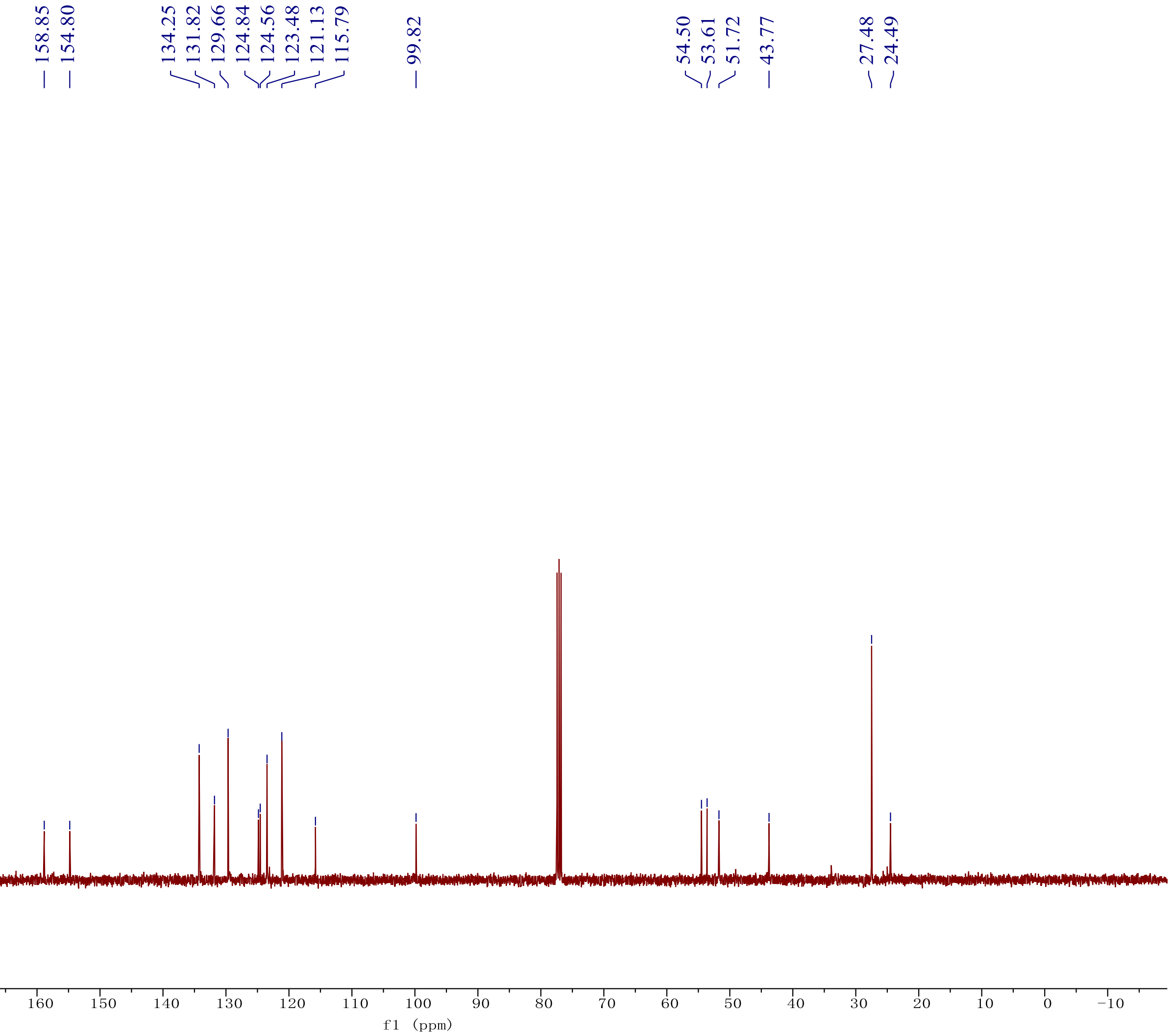

^{1}H NMR (400 MHz, $CDCl_3$) of **2ak’**

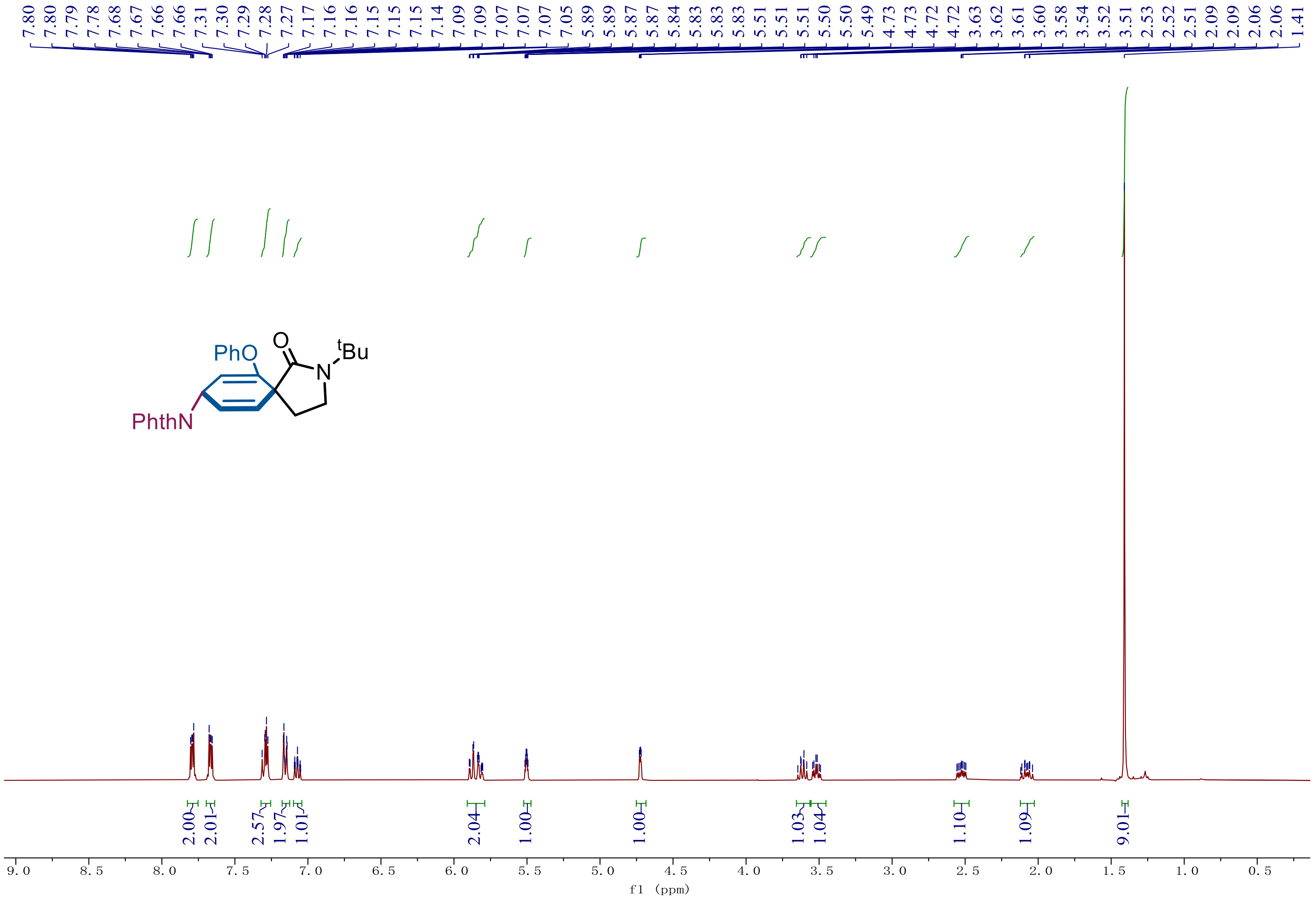

^{13}C NMR (101 MHz, CDCl$_3$) of **2ak'**

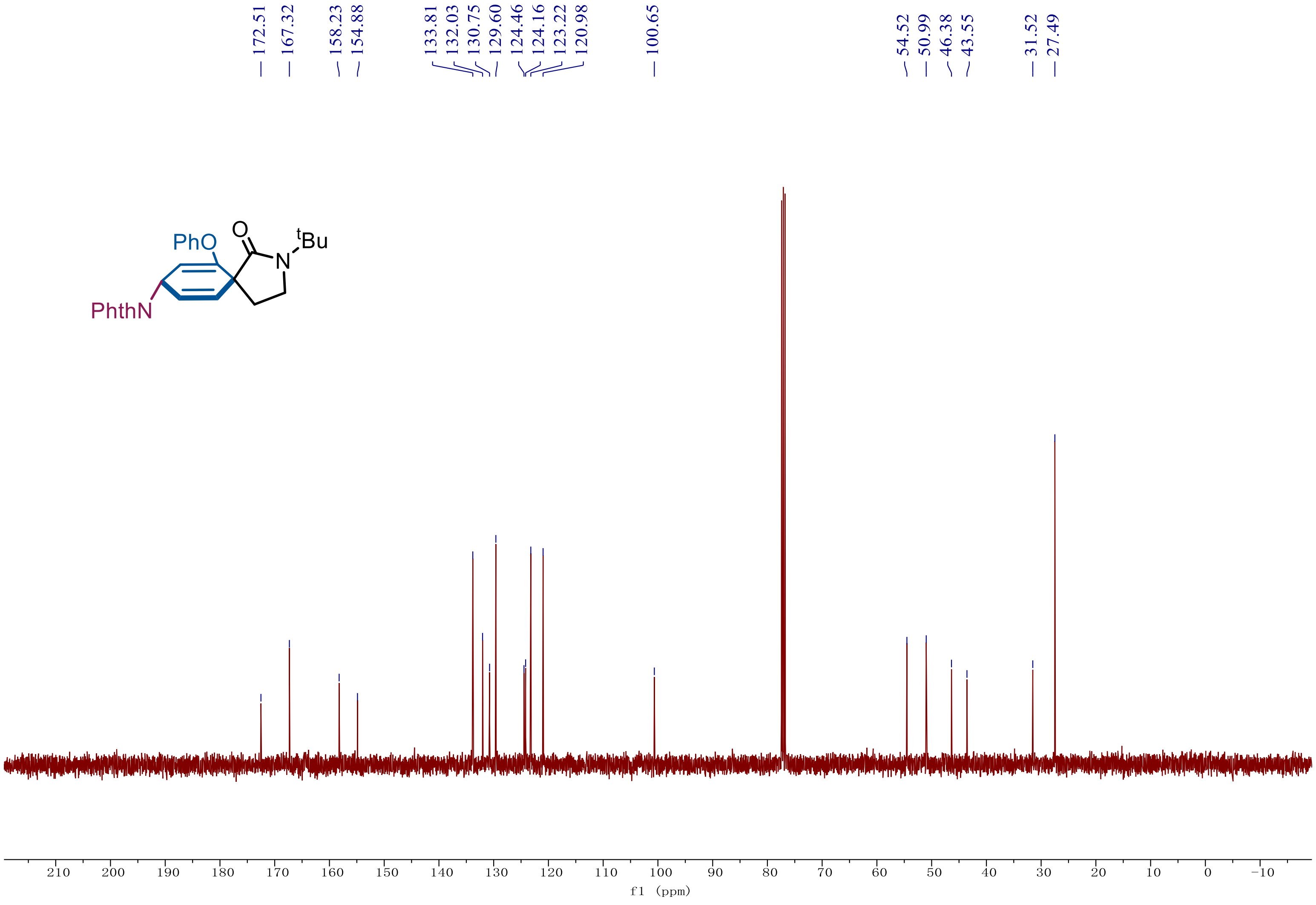

^{1}H NMR (500 MHz, $CDCl_3$) of **2al**

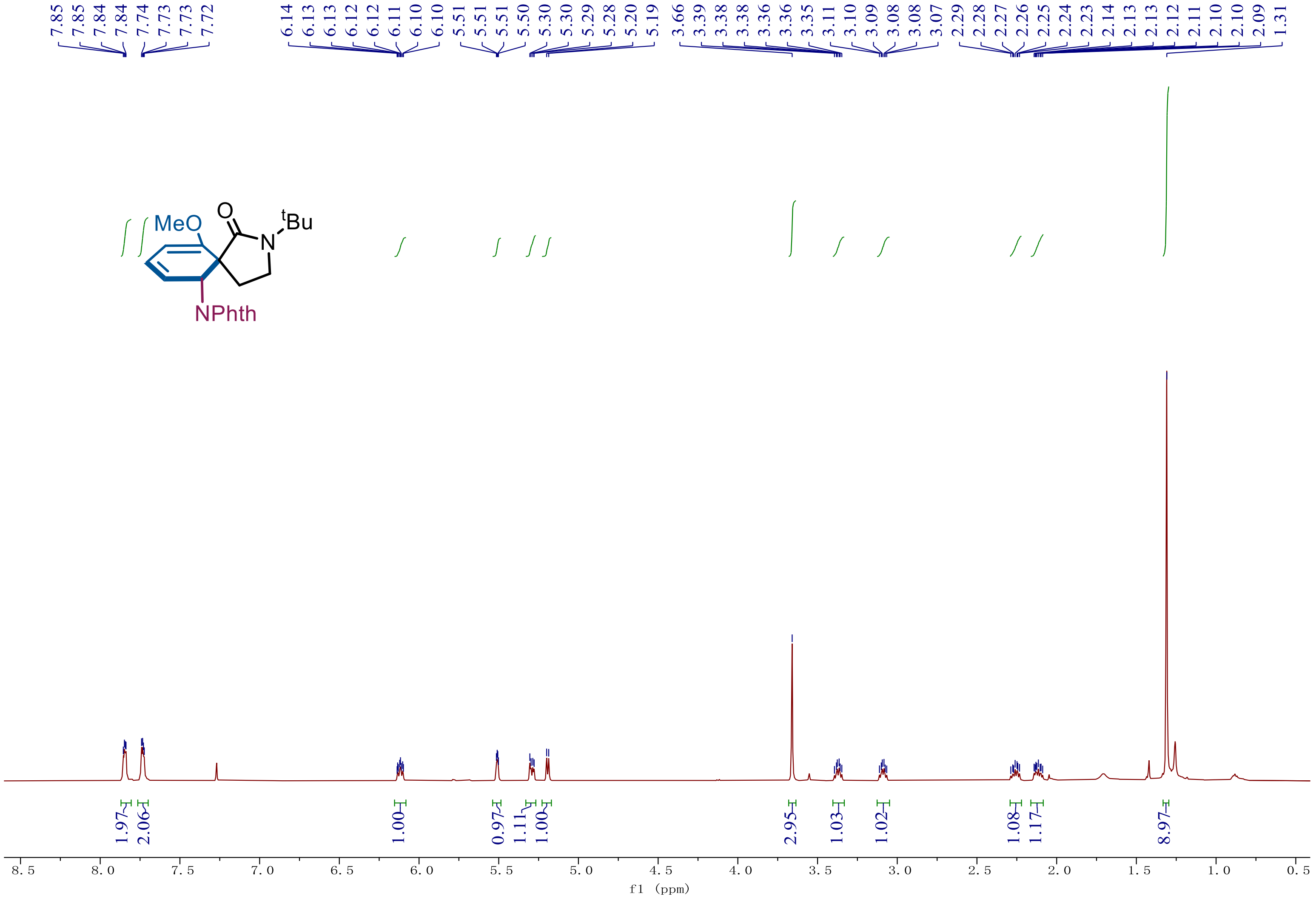

^{13}C NMR (126 MHz, $CDCl_3$) of **2al**

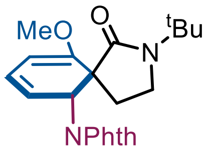

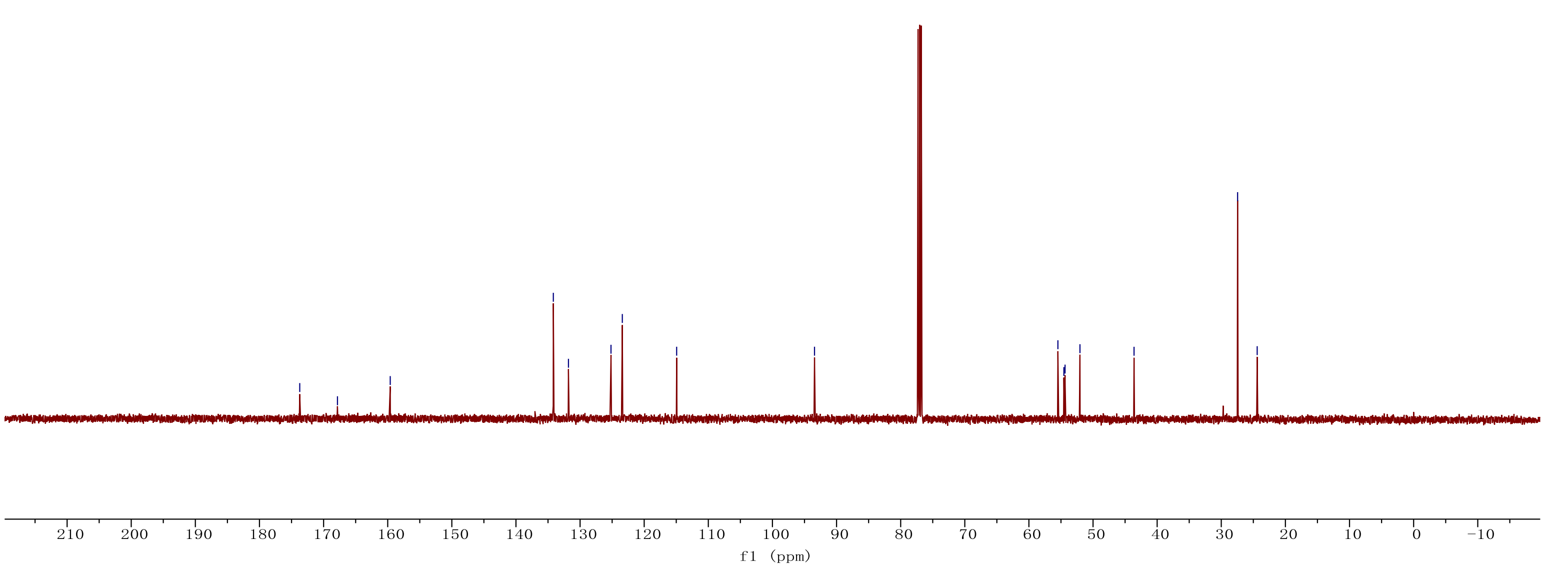

^{1}H NMR (500 MHz, $CDCl_3$) of **2al’**

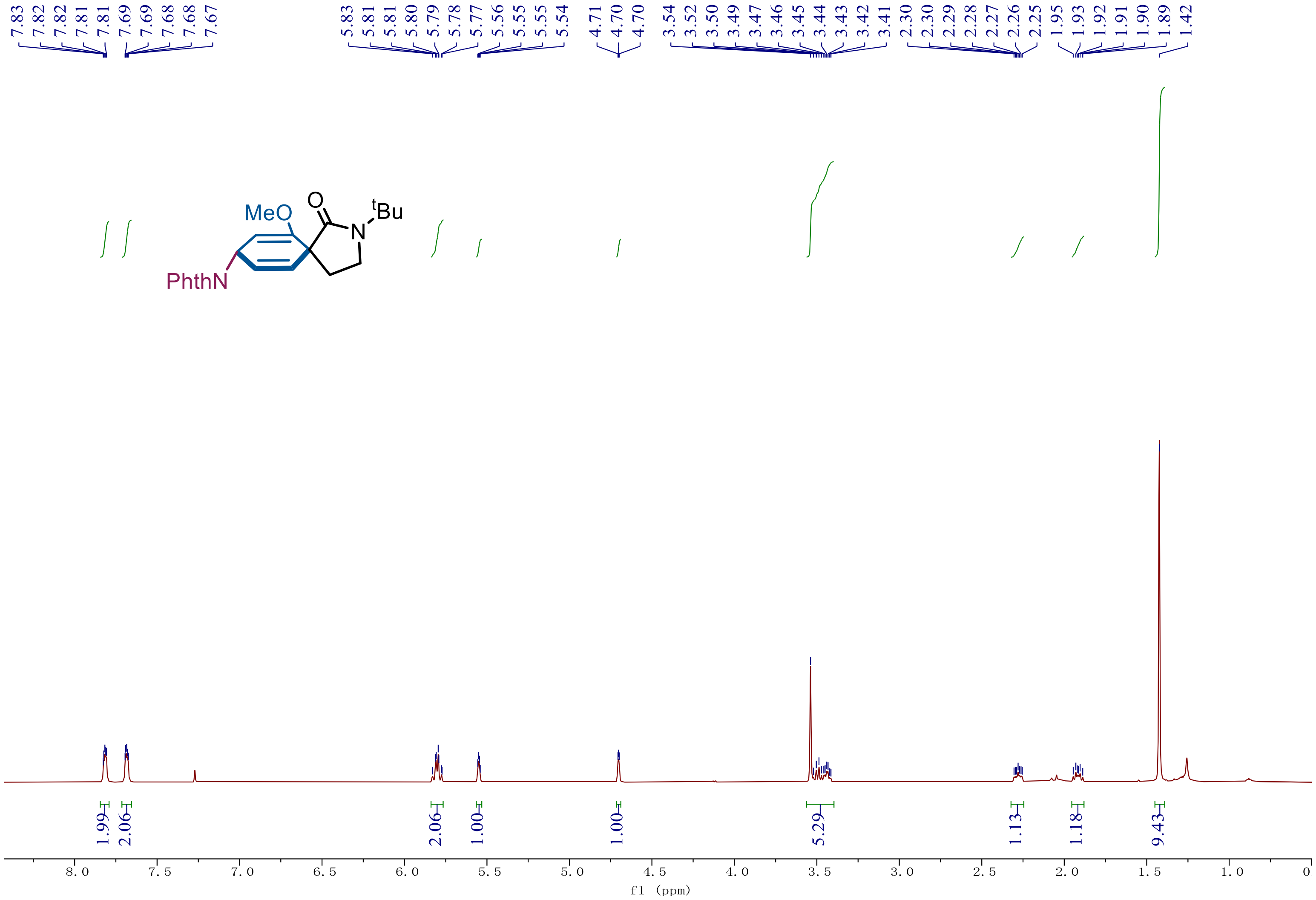

^{13}C NMR (126 MHz, $CDCl_3$) of **2al'**

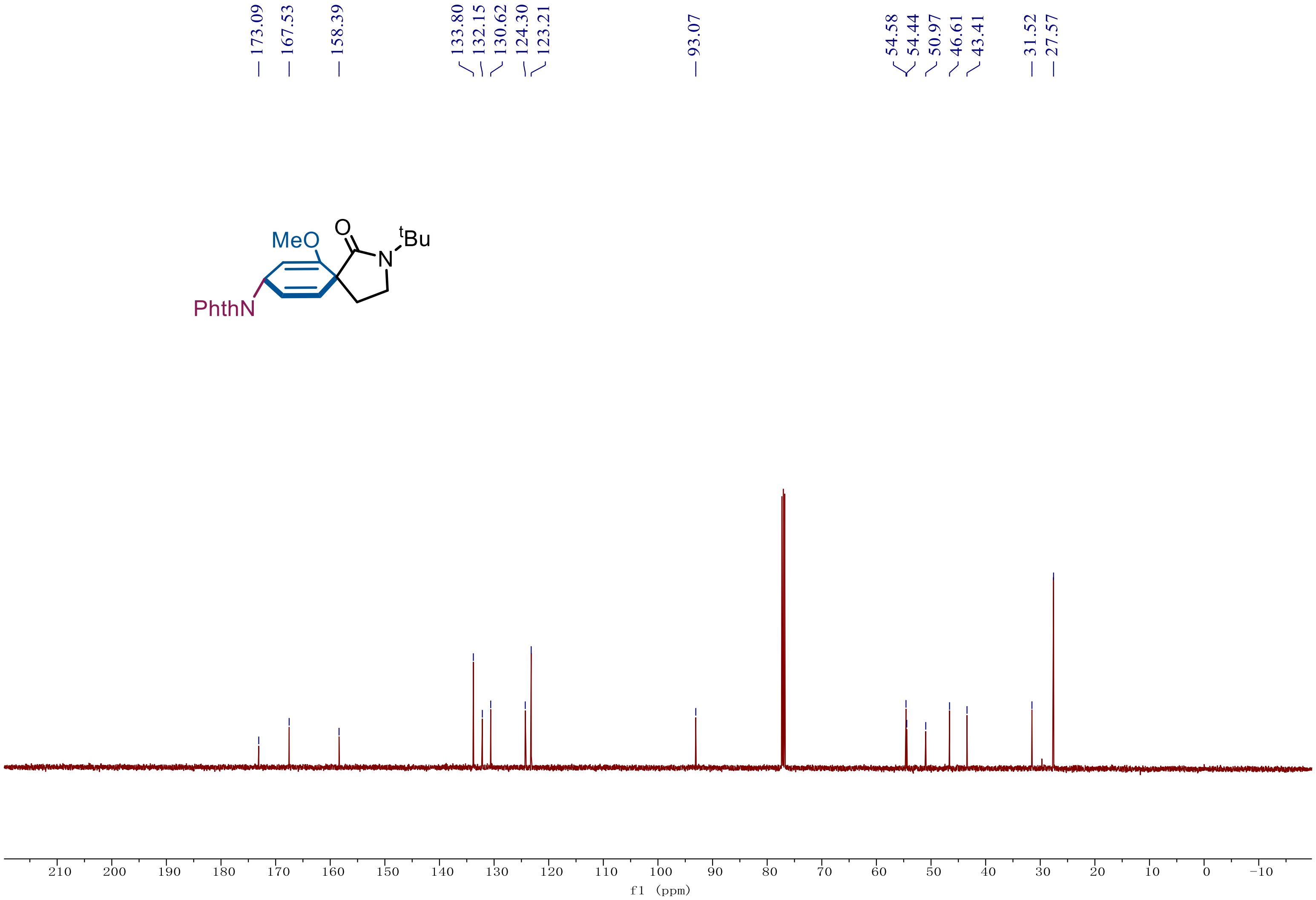

^{1}H NMR (400 MHz, $CDCl_3$) of **2am'**

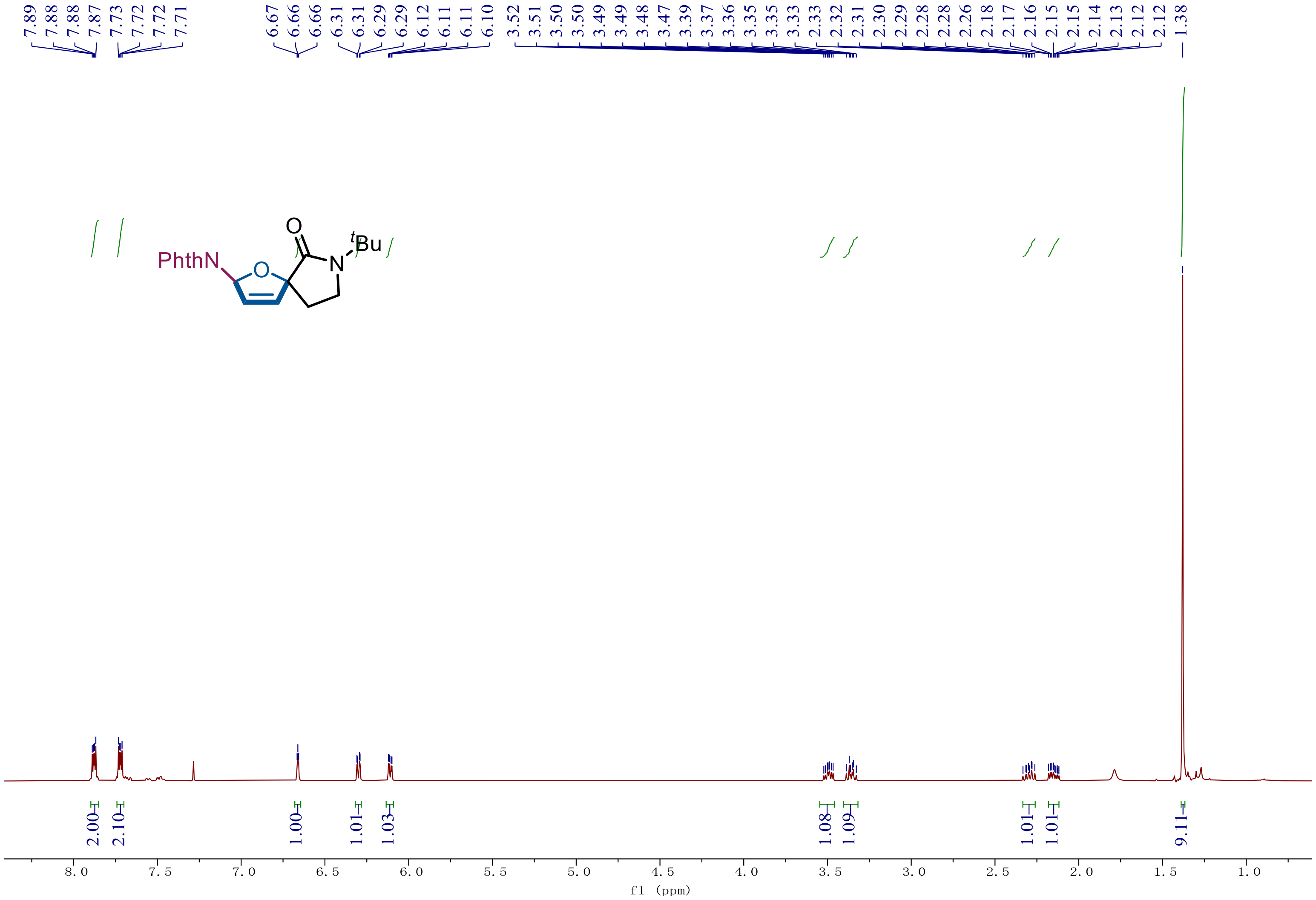

^{13}C NMR (101 MHz, $CDCl_3$) of **2am'**

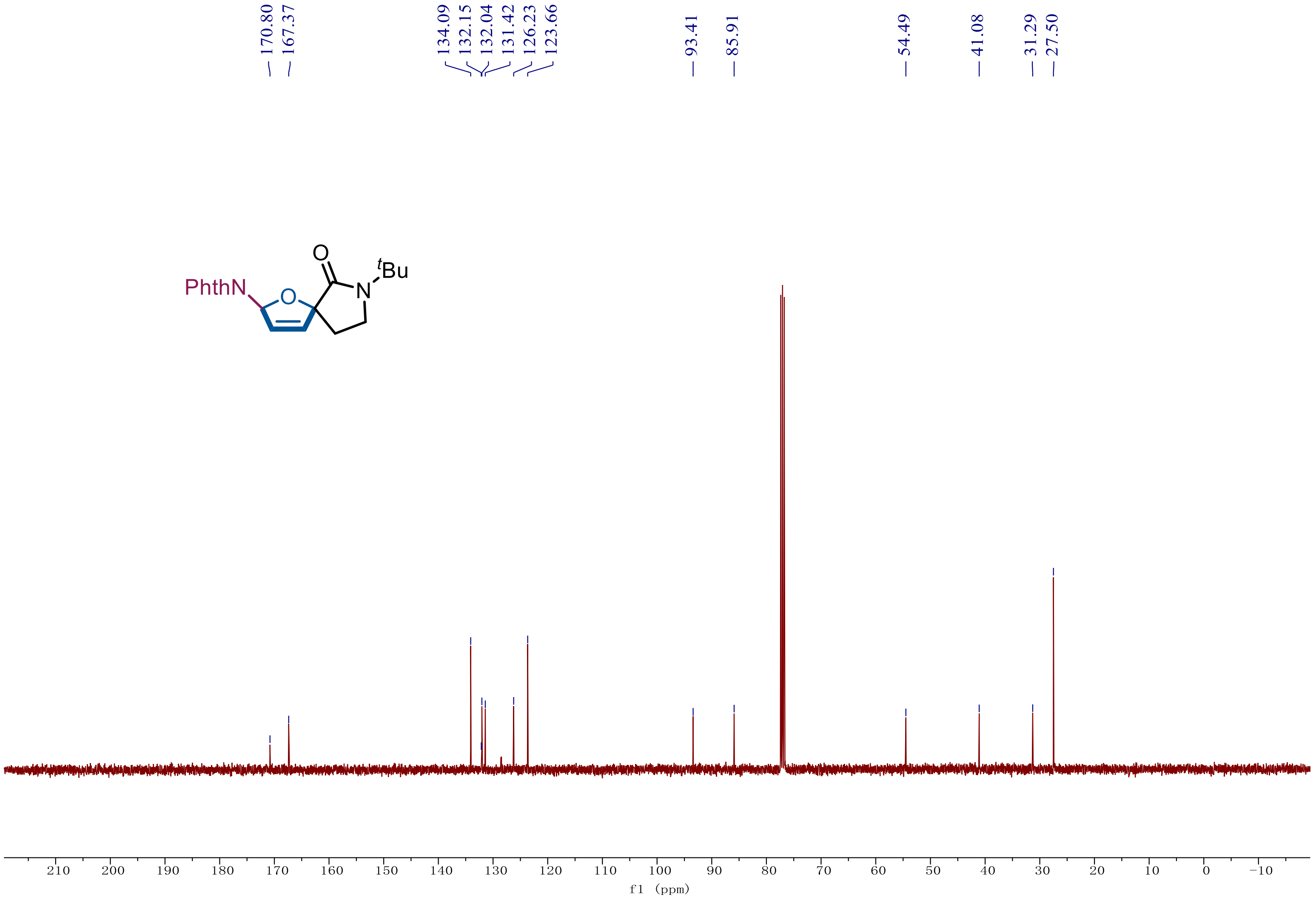

^{1}H NMR (400 MHz, CDCl3) of **2an'**

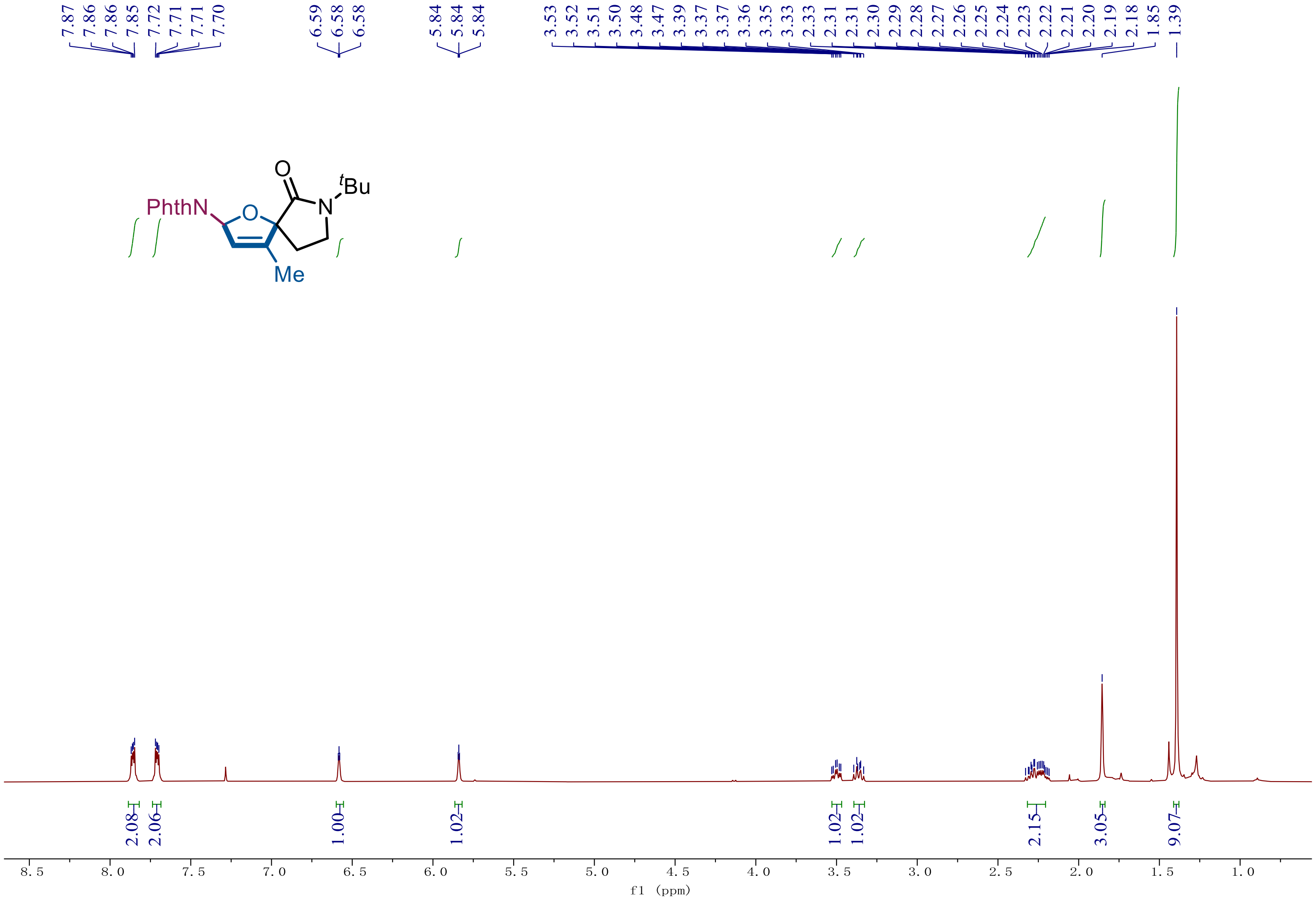

^{13}C NMR (101 MHz, $CDCl_3$) of **2an'**

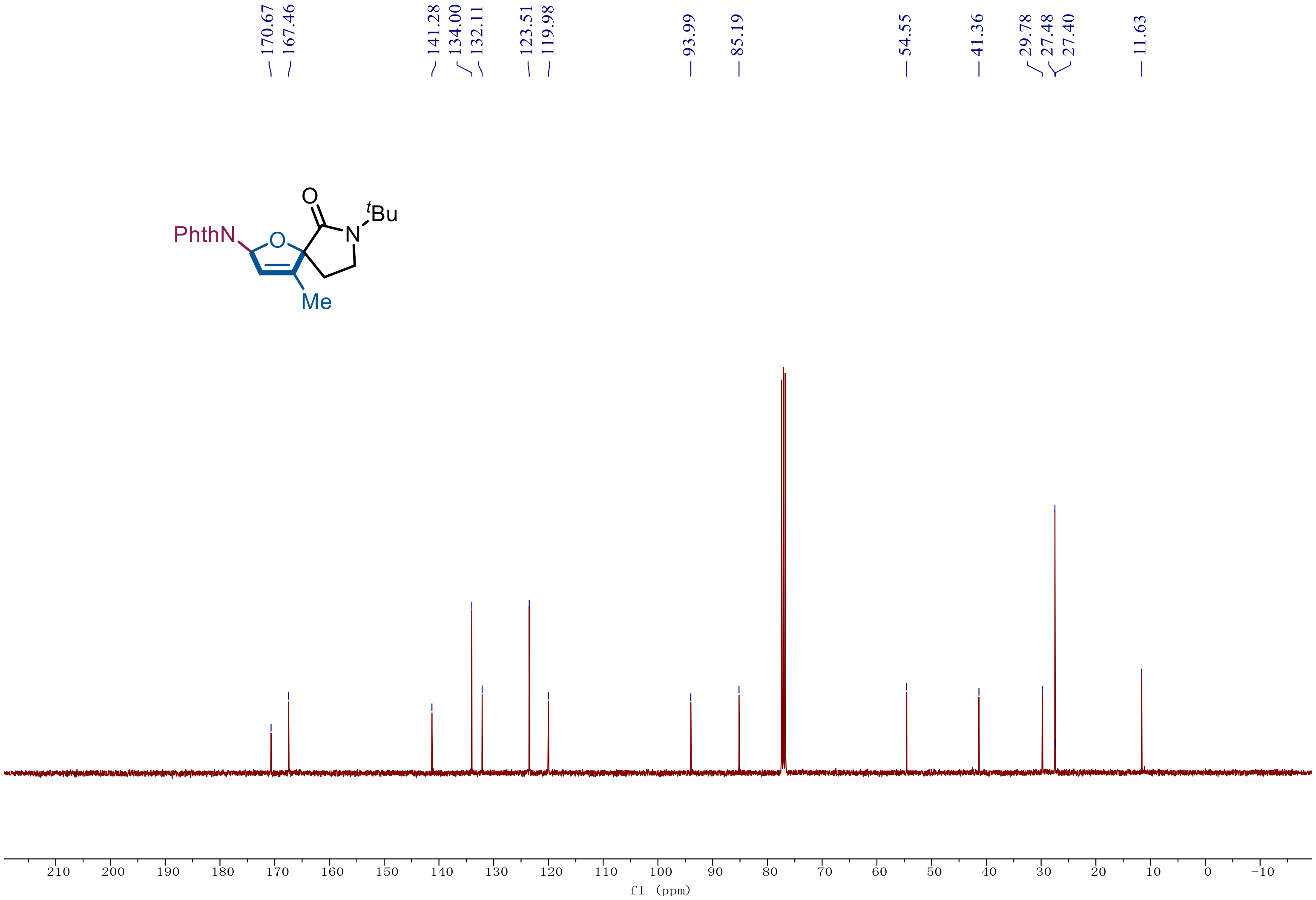

^{1}H NMR (400 MHz, $CDCl_3$) of **4a**

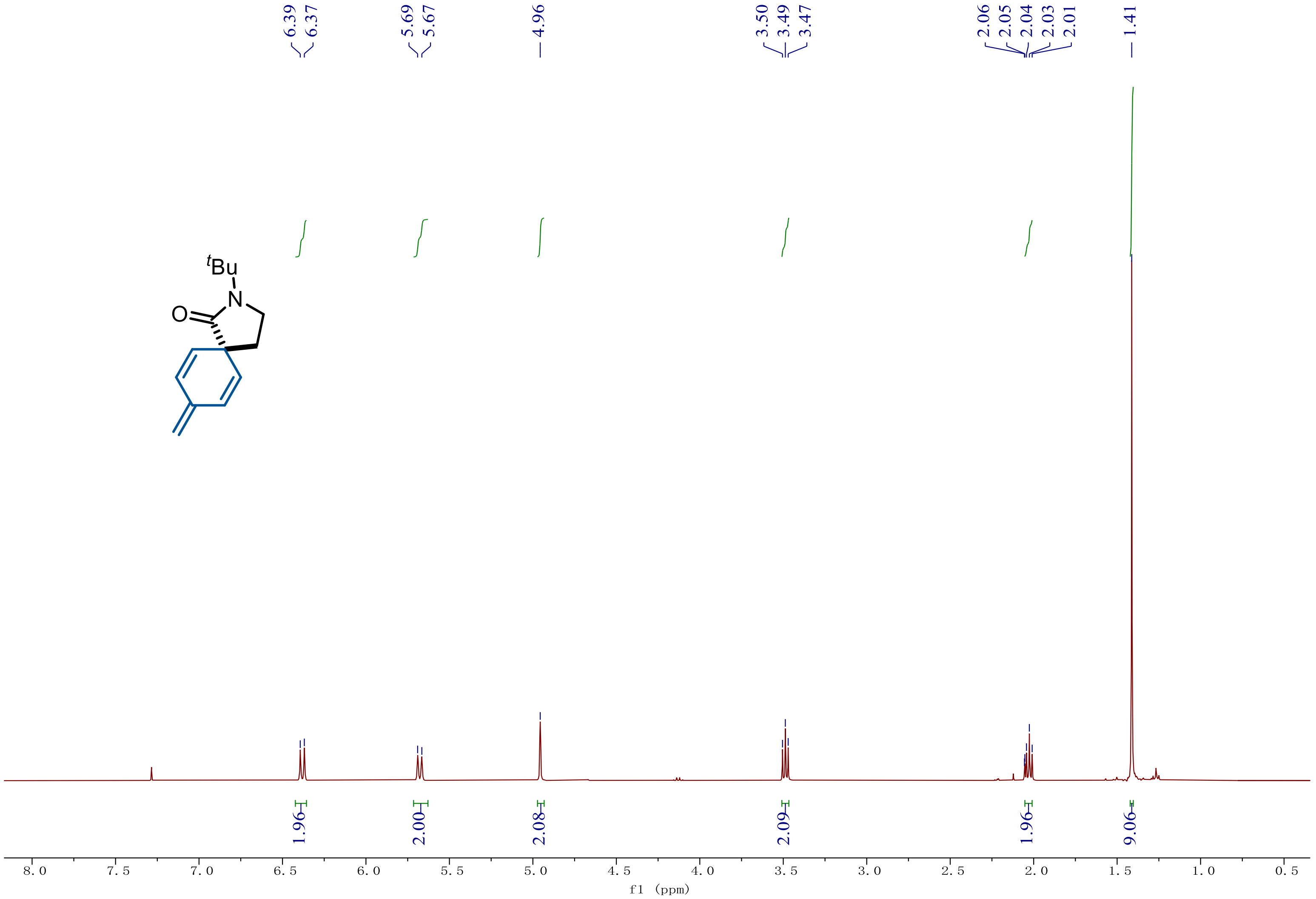

^{13}C NMR (101 MHz, $CDCl_3$) of **4a**

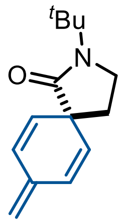

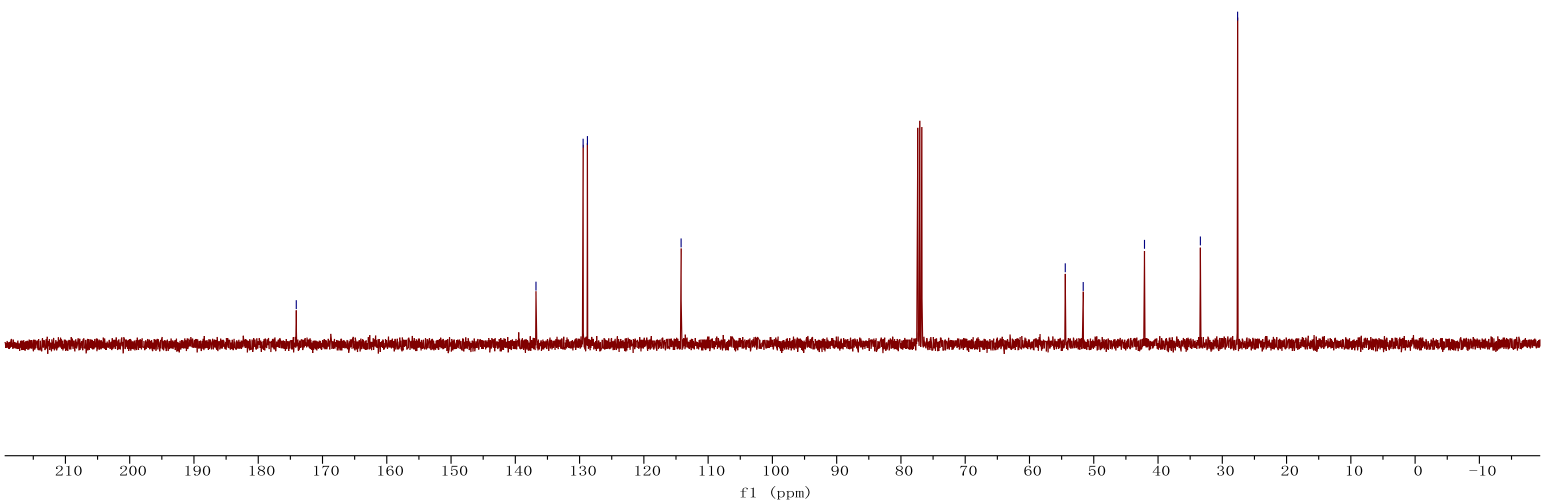

^{1}H NMR (400 MHz, $CDCl_3$) of **4b**

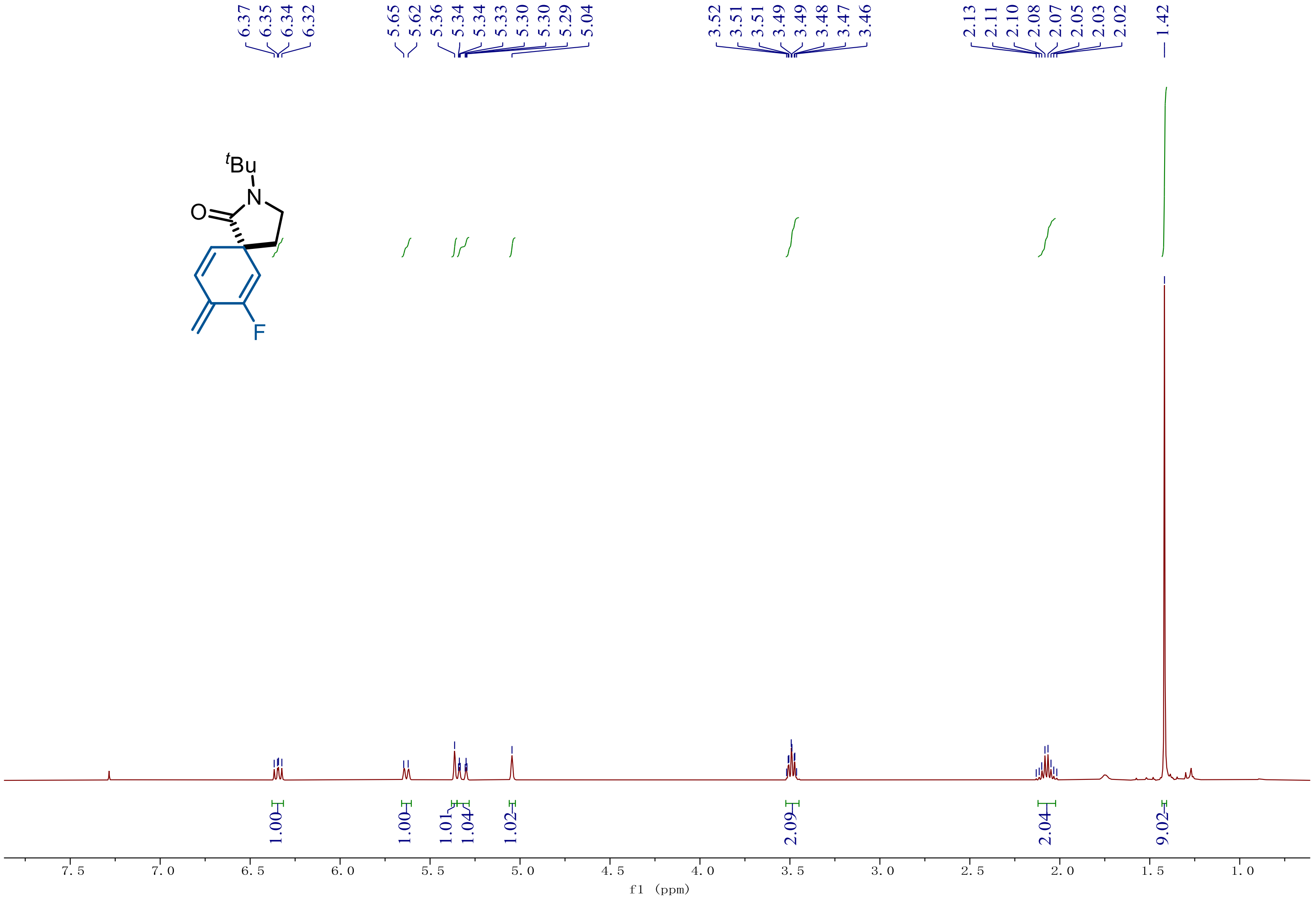

^{13}C NMR (101 MHz, $CDCl_3$) of **4b**

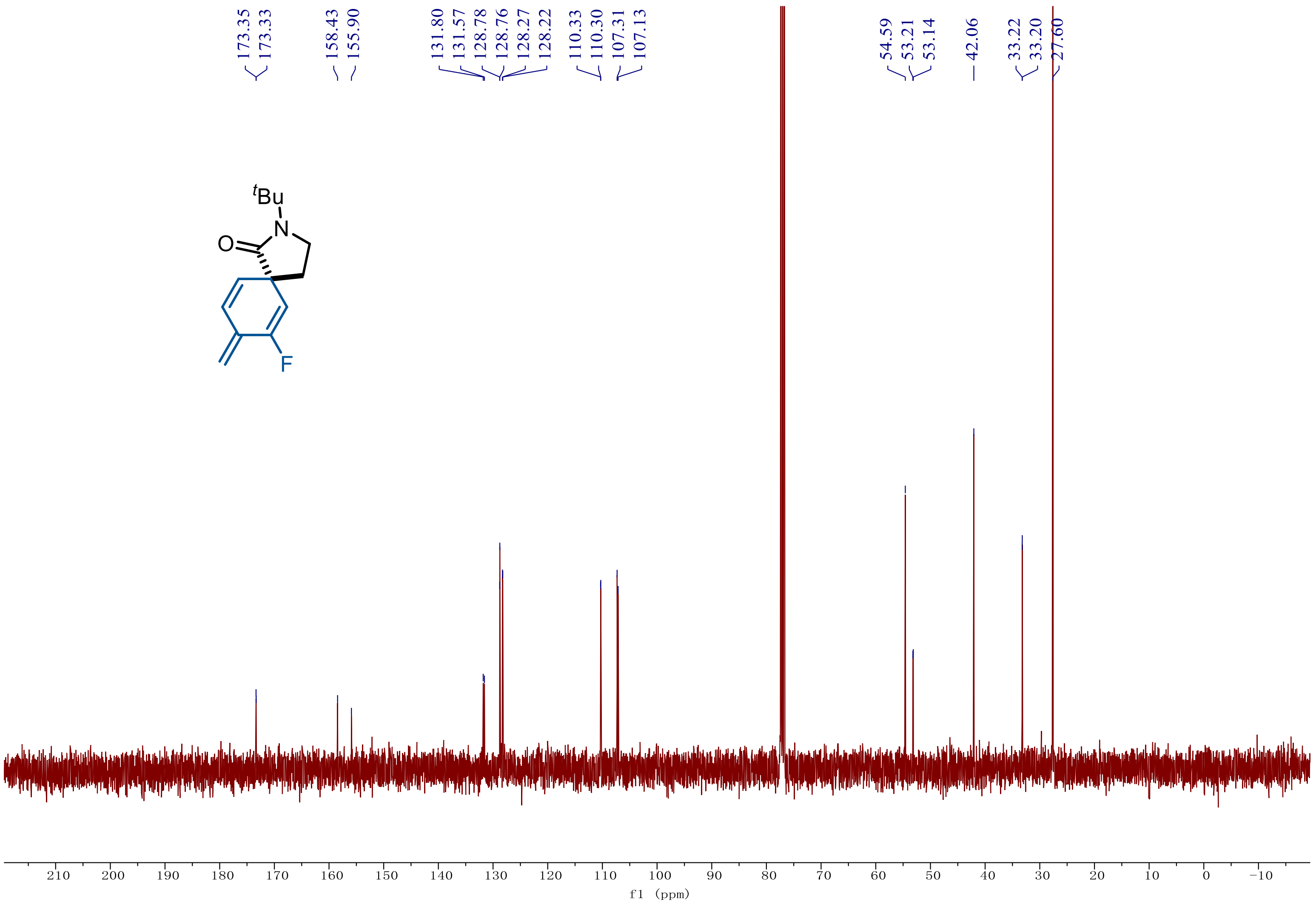

^{19}F NMR (376 MHz, $CDCl_3$) of **4b**

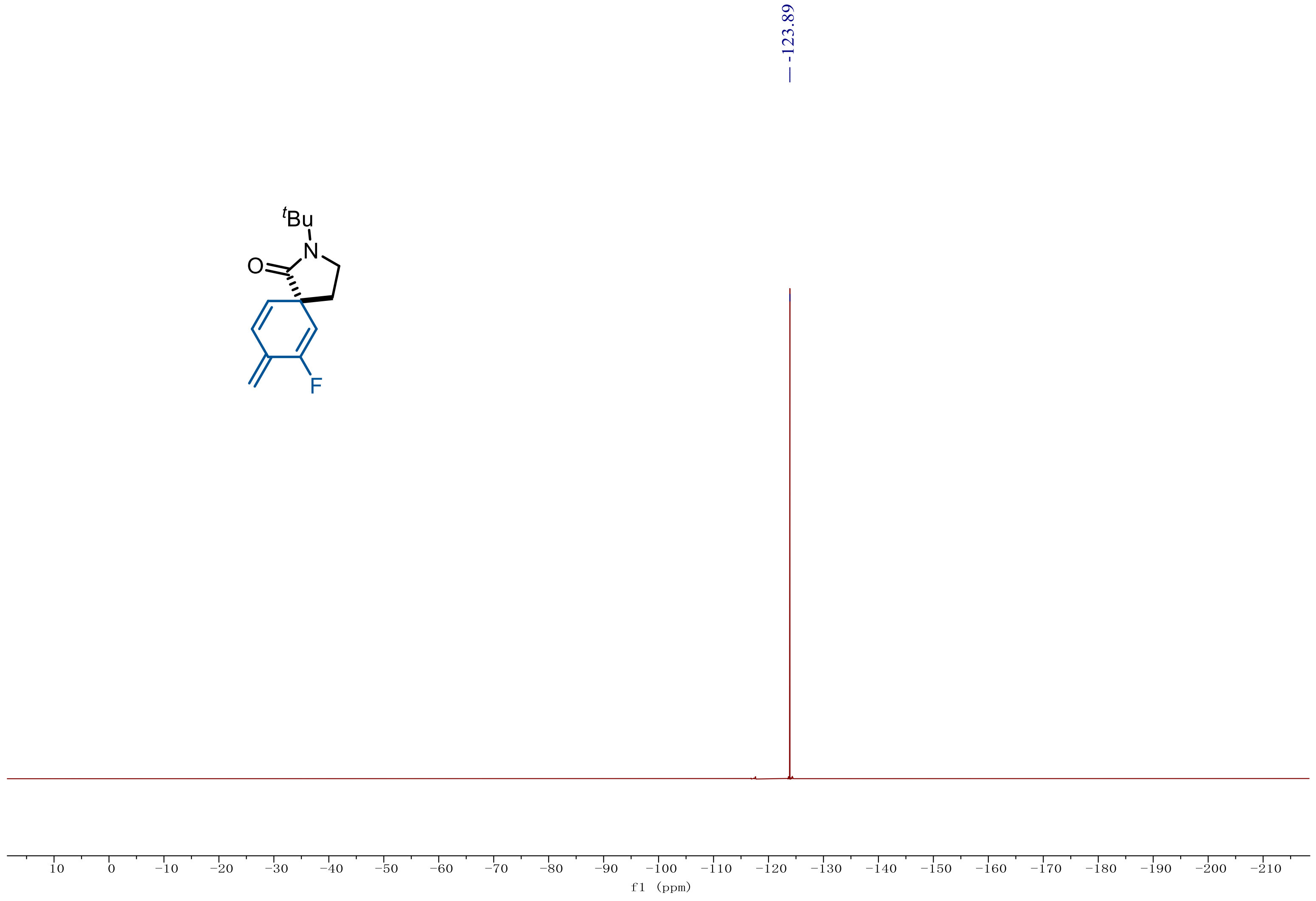

^{1}H NMR (400 MHz, $CDCl_3$) of **4c**

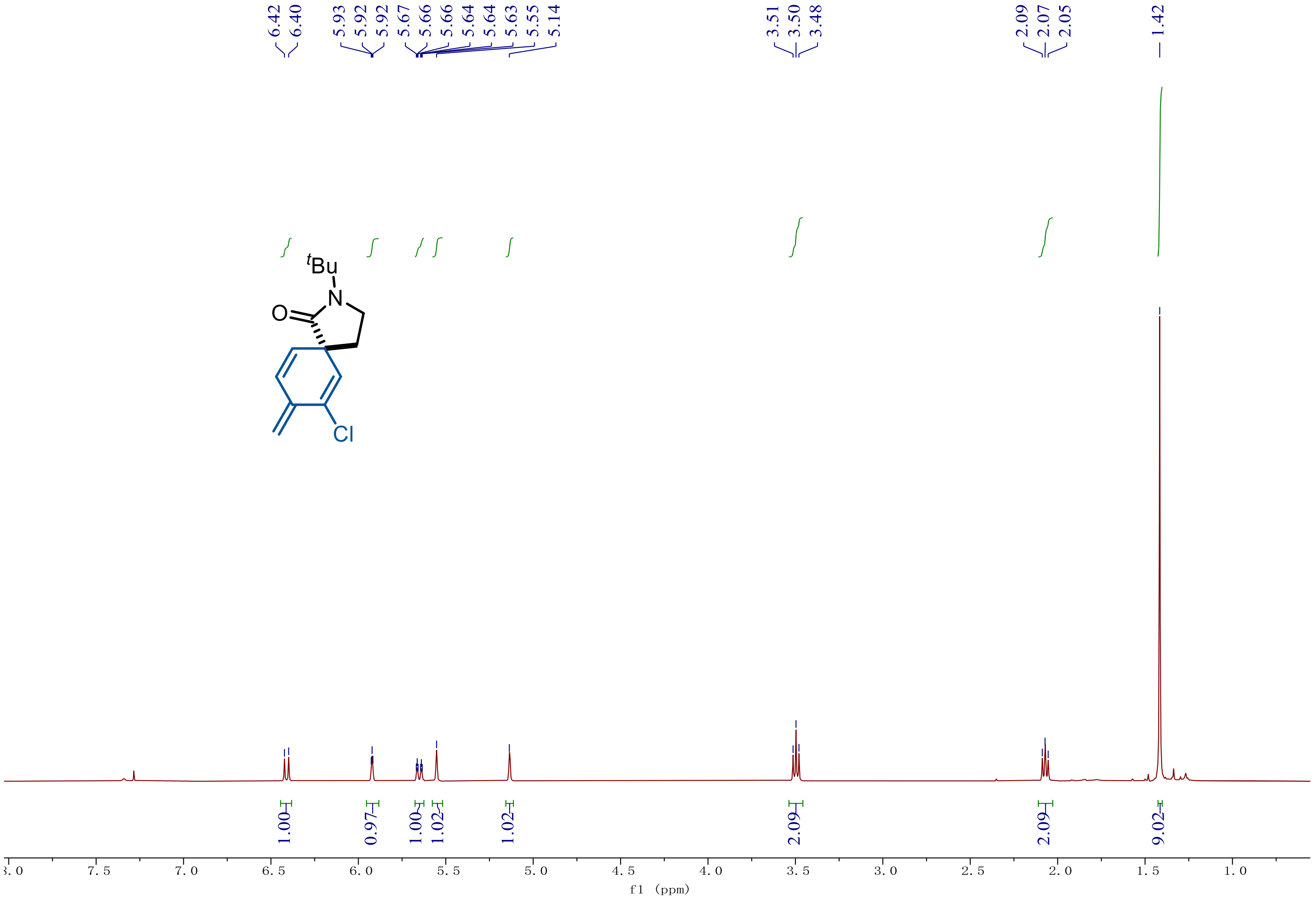

^{13}C NMR (101 MHz, $CDCl_3$) of **4c**

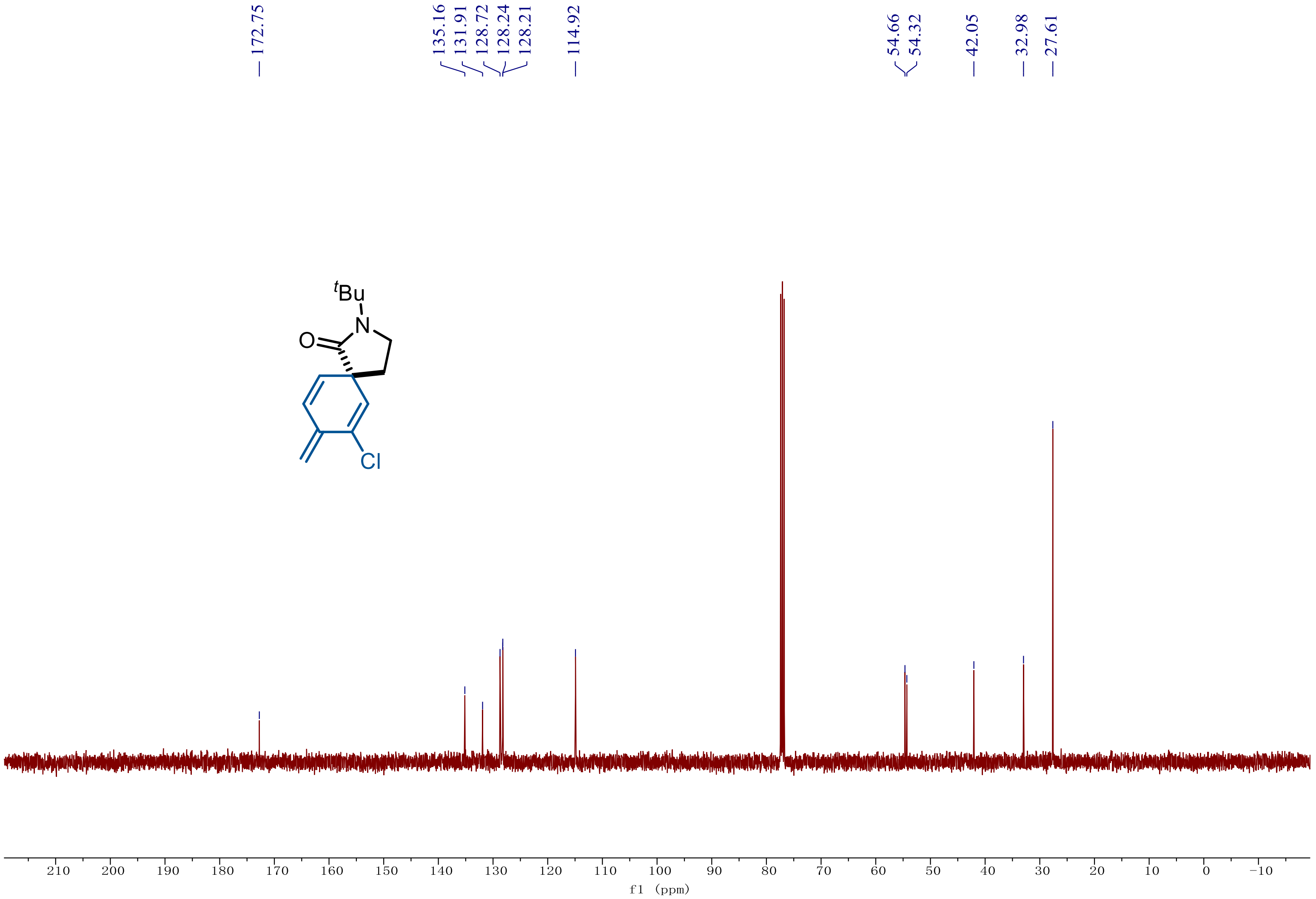

^{1}H NMR (400 MHz, $CDCl_3$) of **4d**

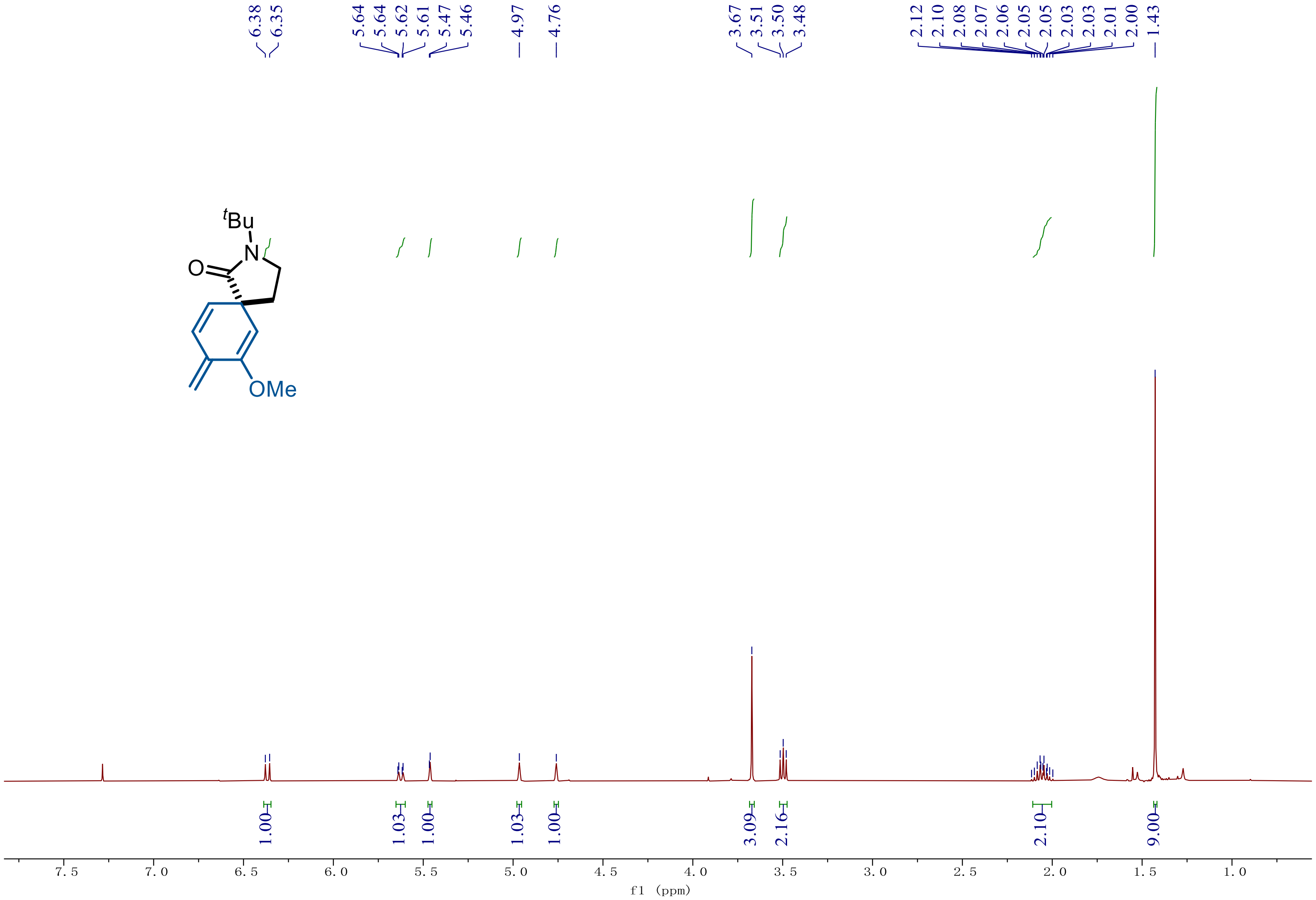

^{13}C NMR (101 MHz, $CDCl_3$) of **4d**

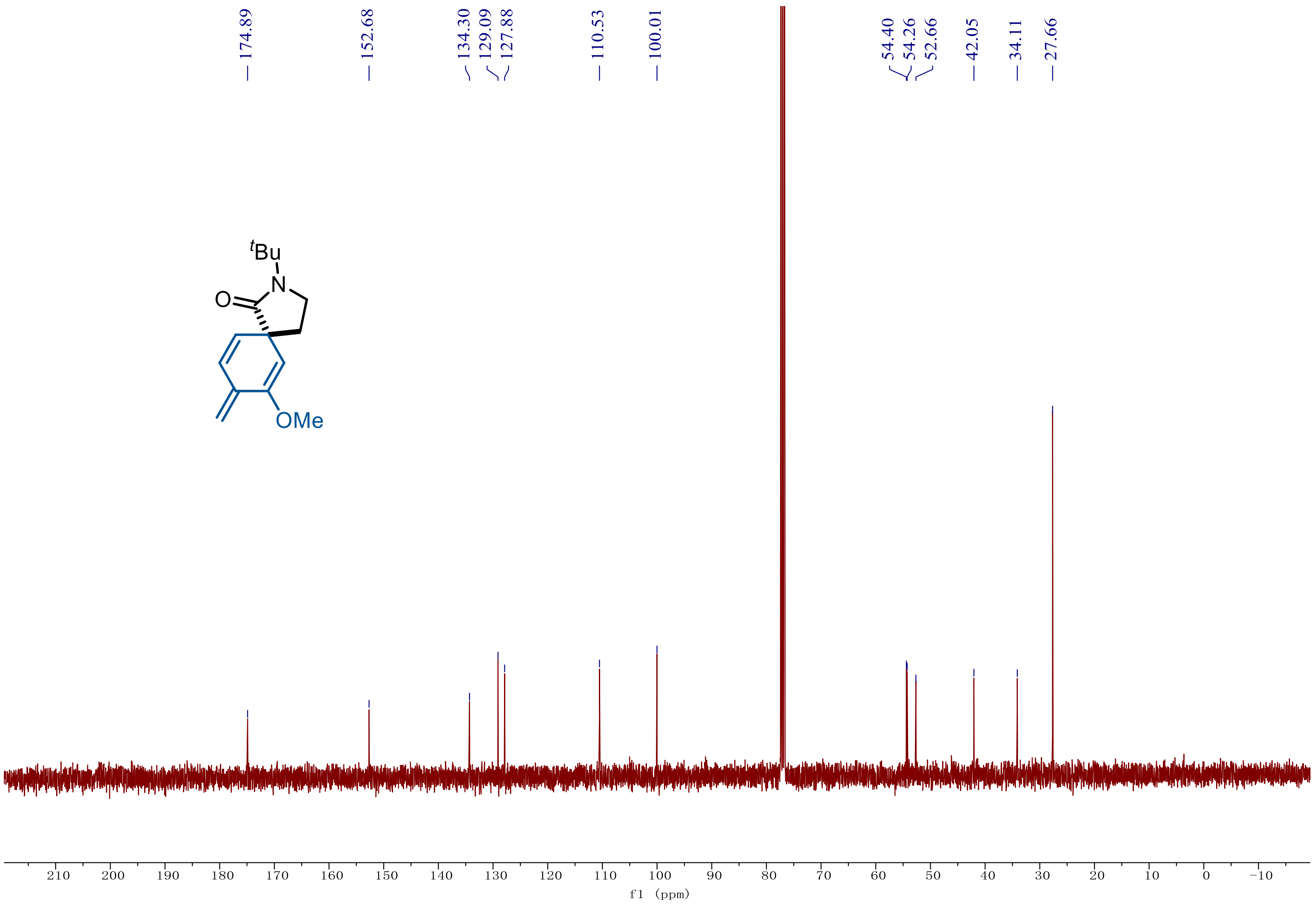

^{1}H NMR (400 MHz, $CDCl_3$) of **4e**

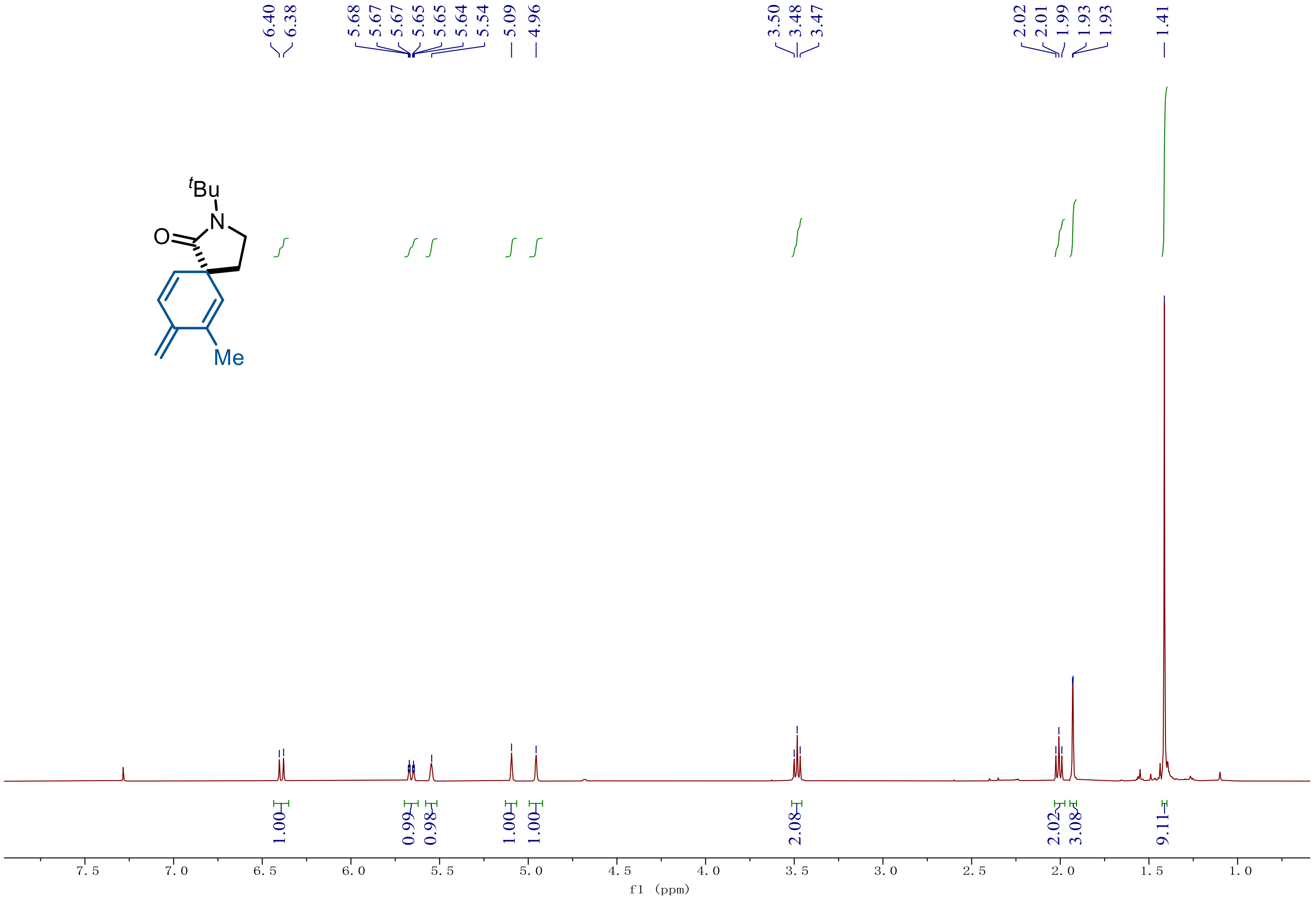

^{13}C NMR (101 MHz, CDCl$_3$) of **4e**

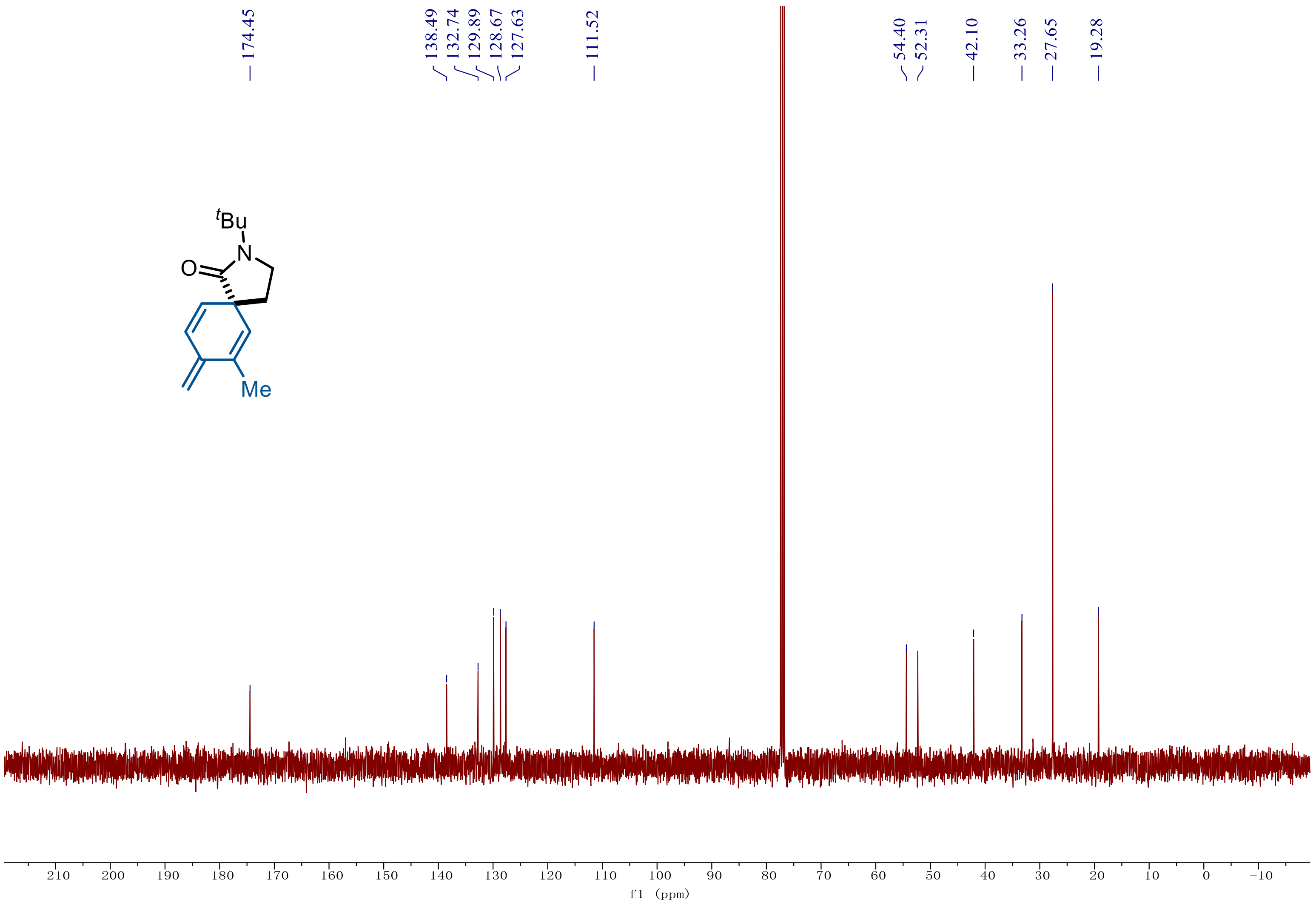

^{1}H NMR (500 MHz, $CDCl_3$) of **4f**

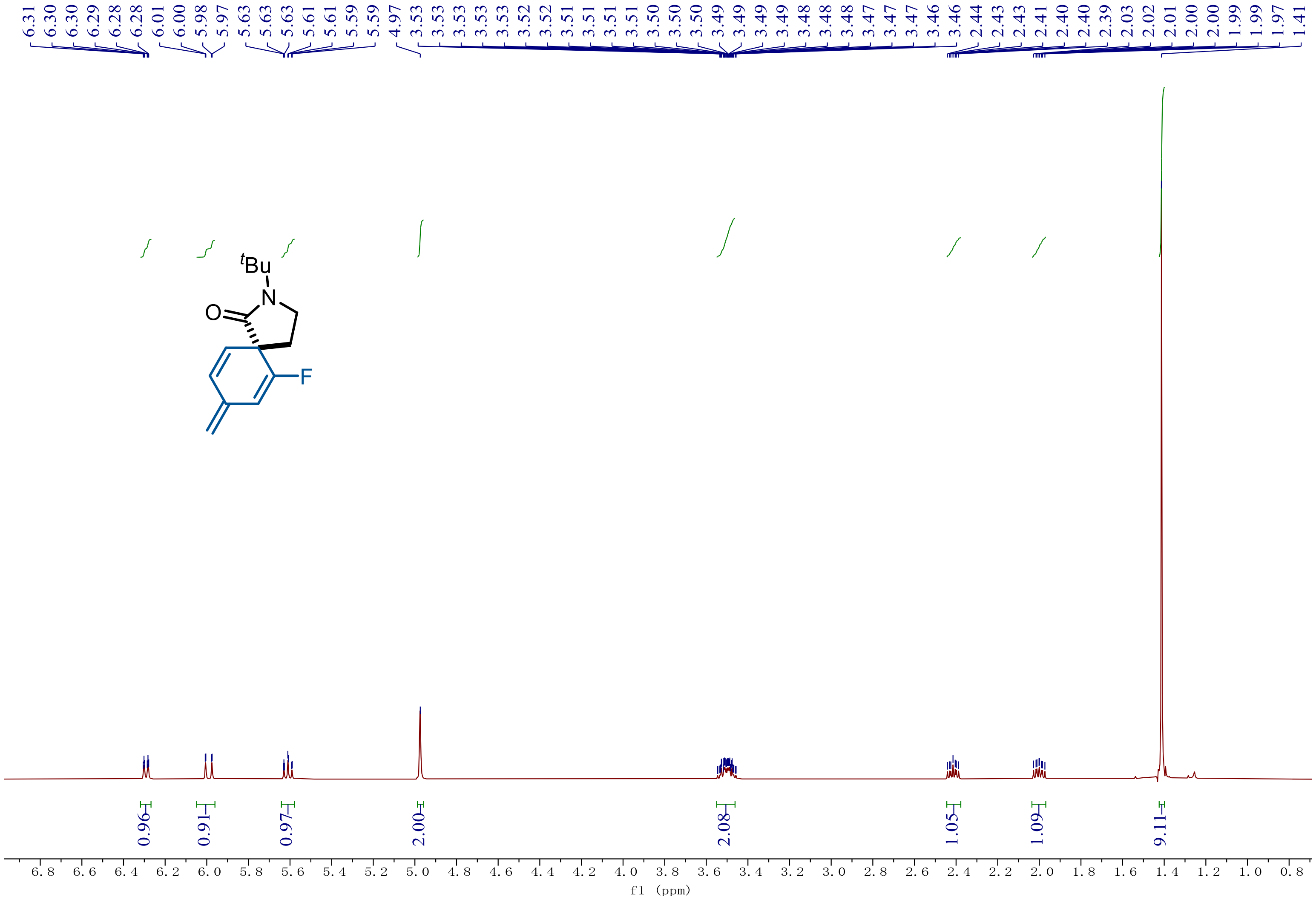

^{13}C NMR (126 MHz, $CDCl_3$) of **4f**

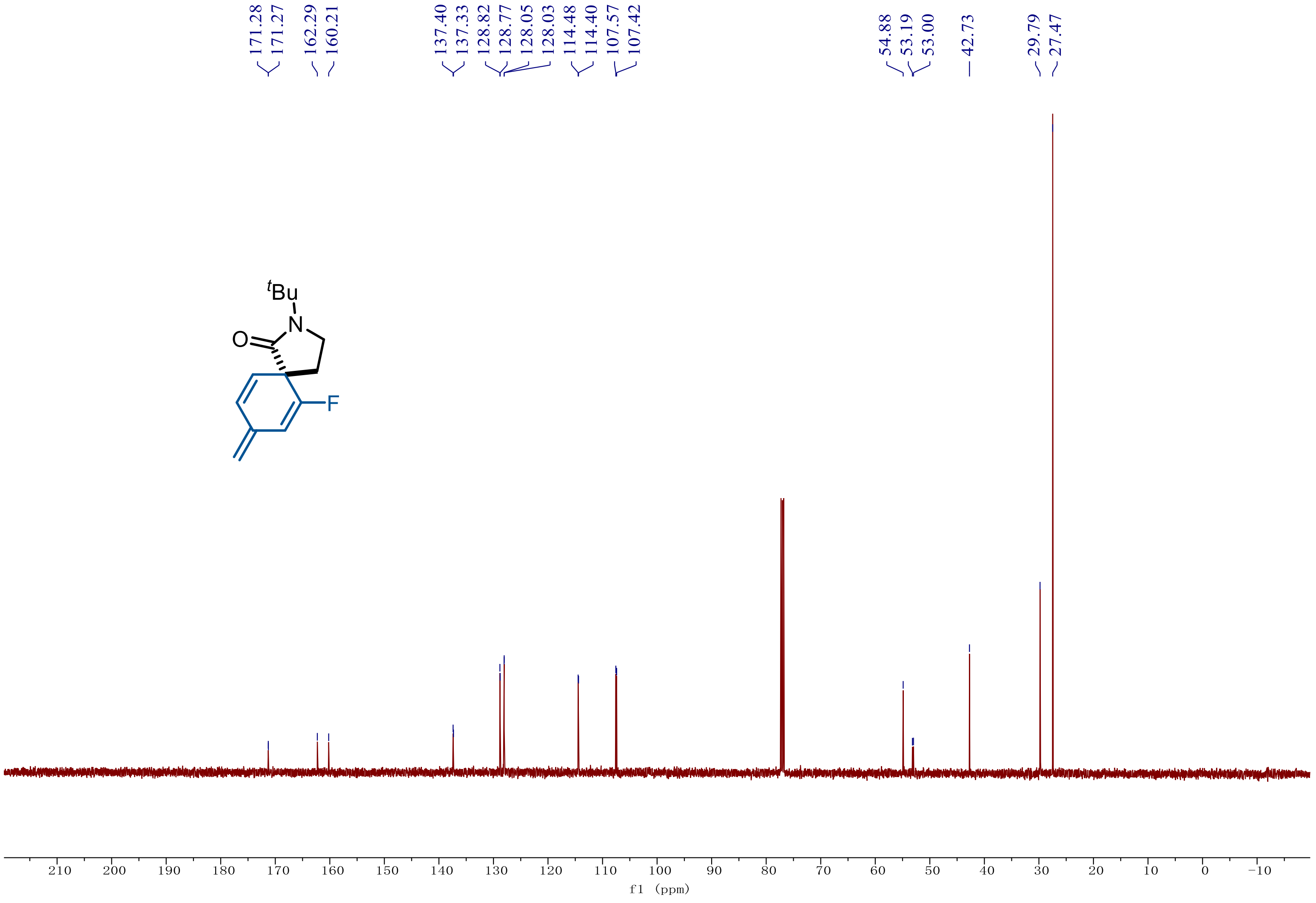

^{19}F NMR (471 MHz, CDCl3) of **4f**

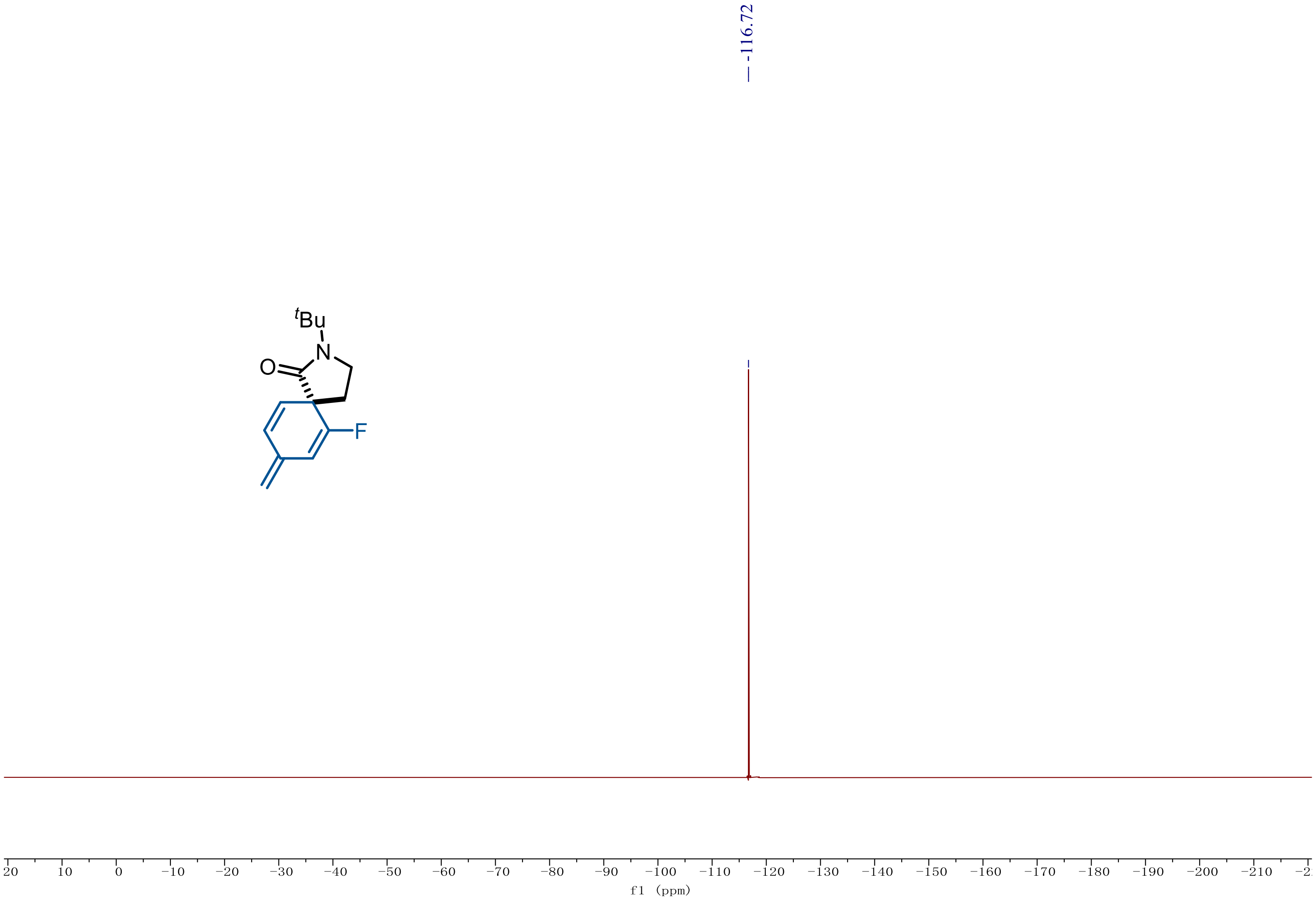

^{1}H NMR (400 MHz, $CDCl_3$) of **4g**

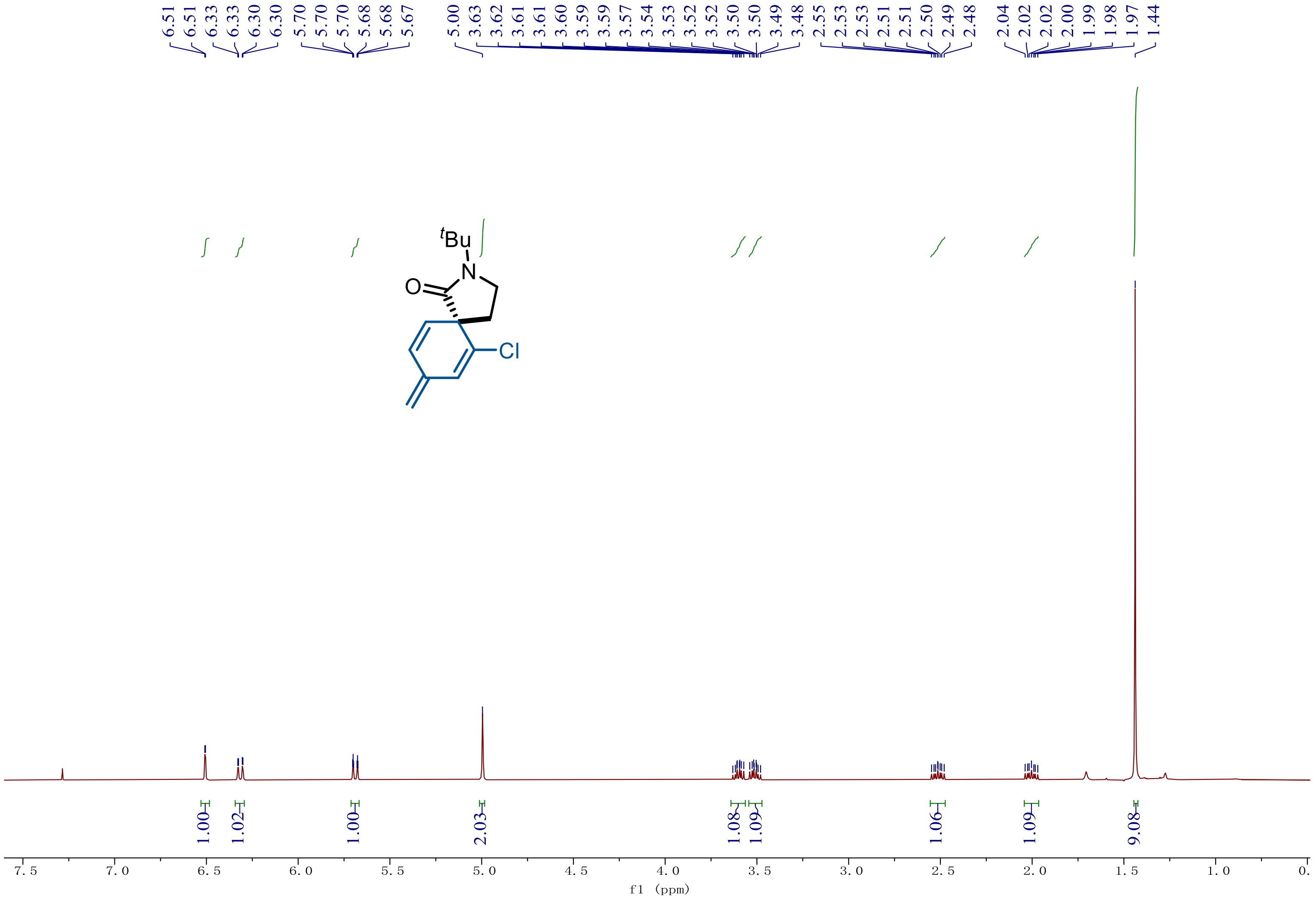

^{13}C NMR (101 MHz, $CDCl_3$) of **4g**

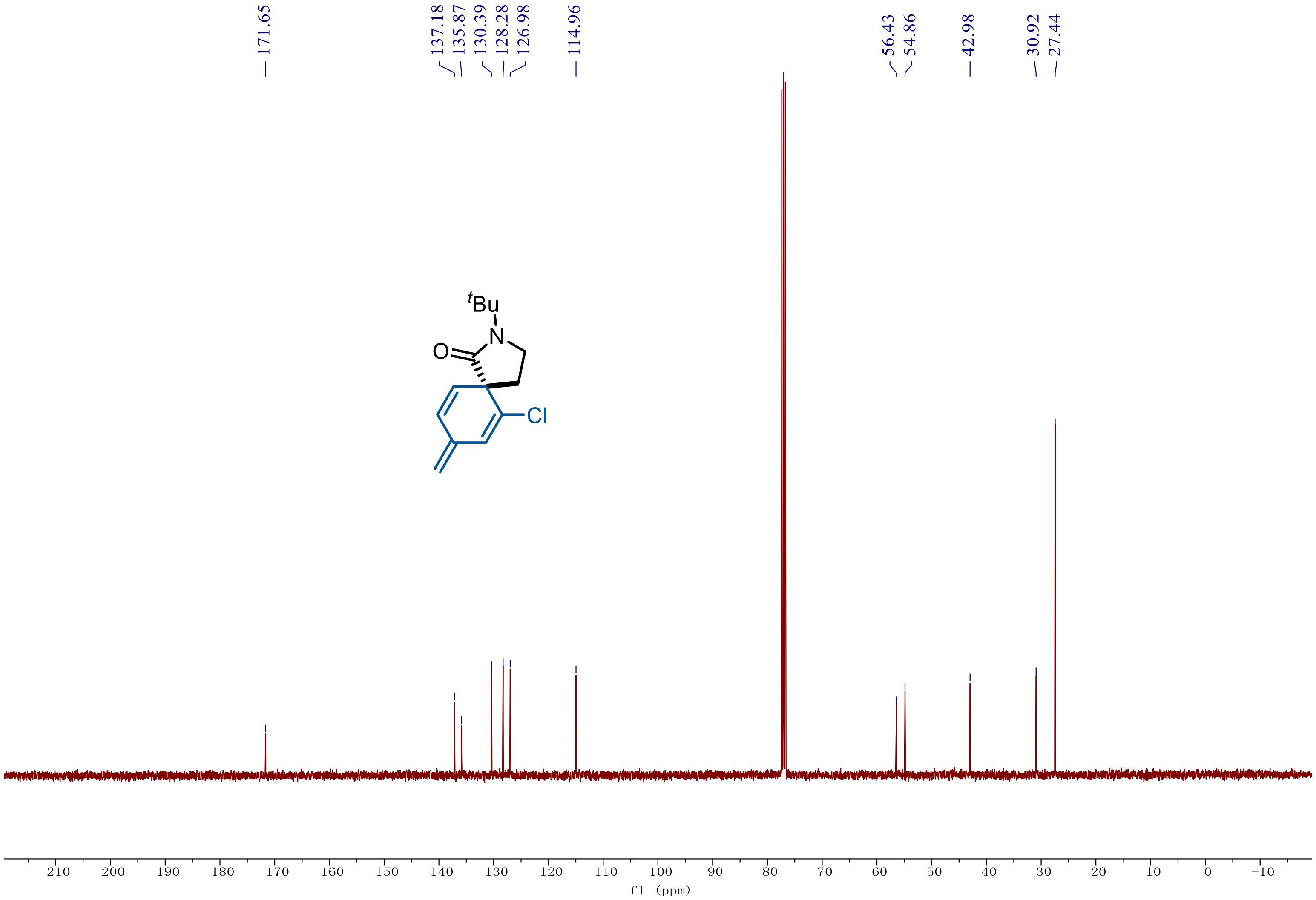

^1H NMR (400 MHz, $CDCl_3$) of **4h**

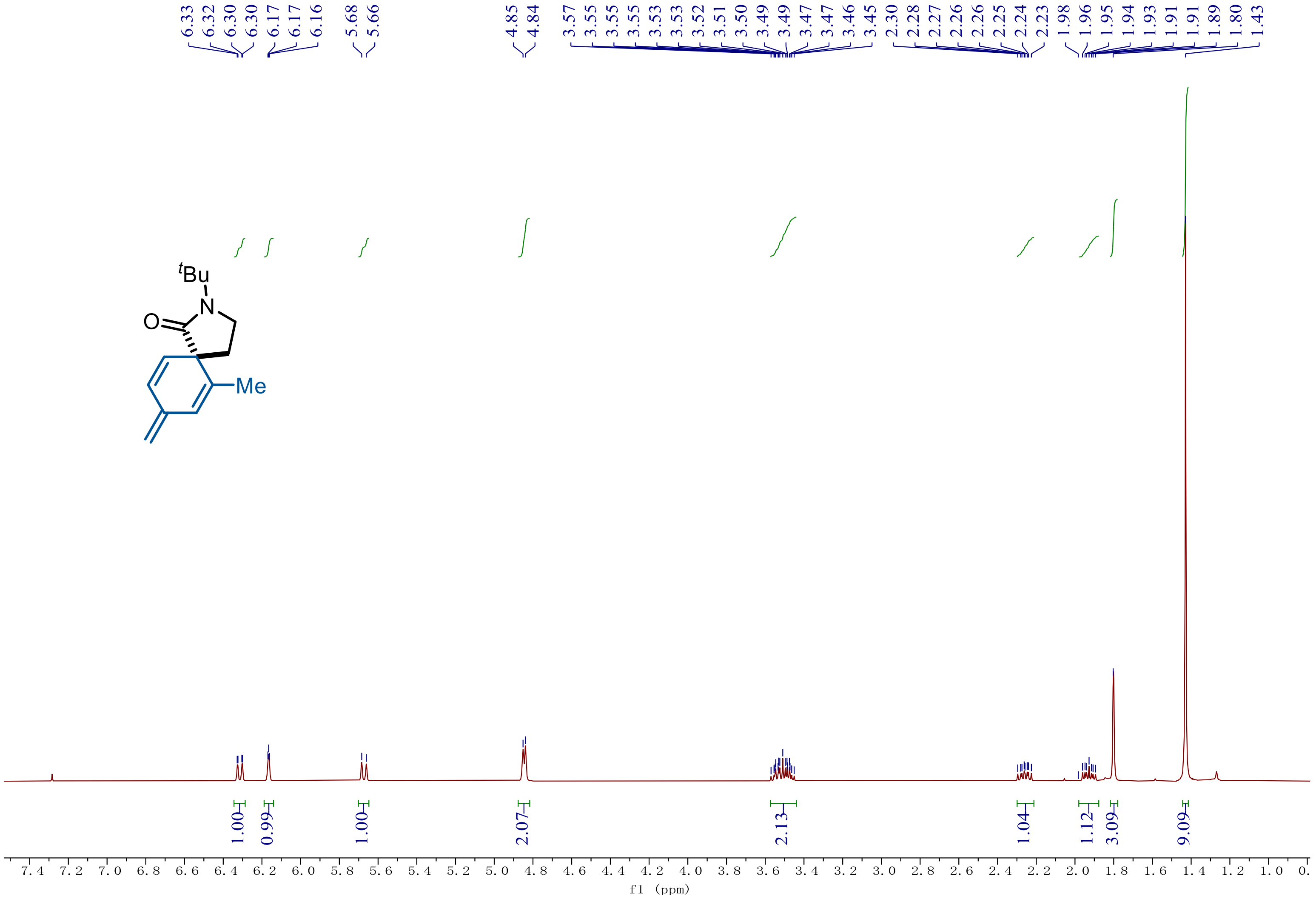

^{13}C NMR (101 MHz, $CDCl_3$) of **4h**

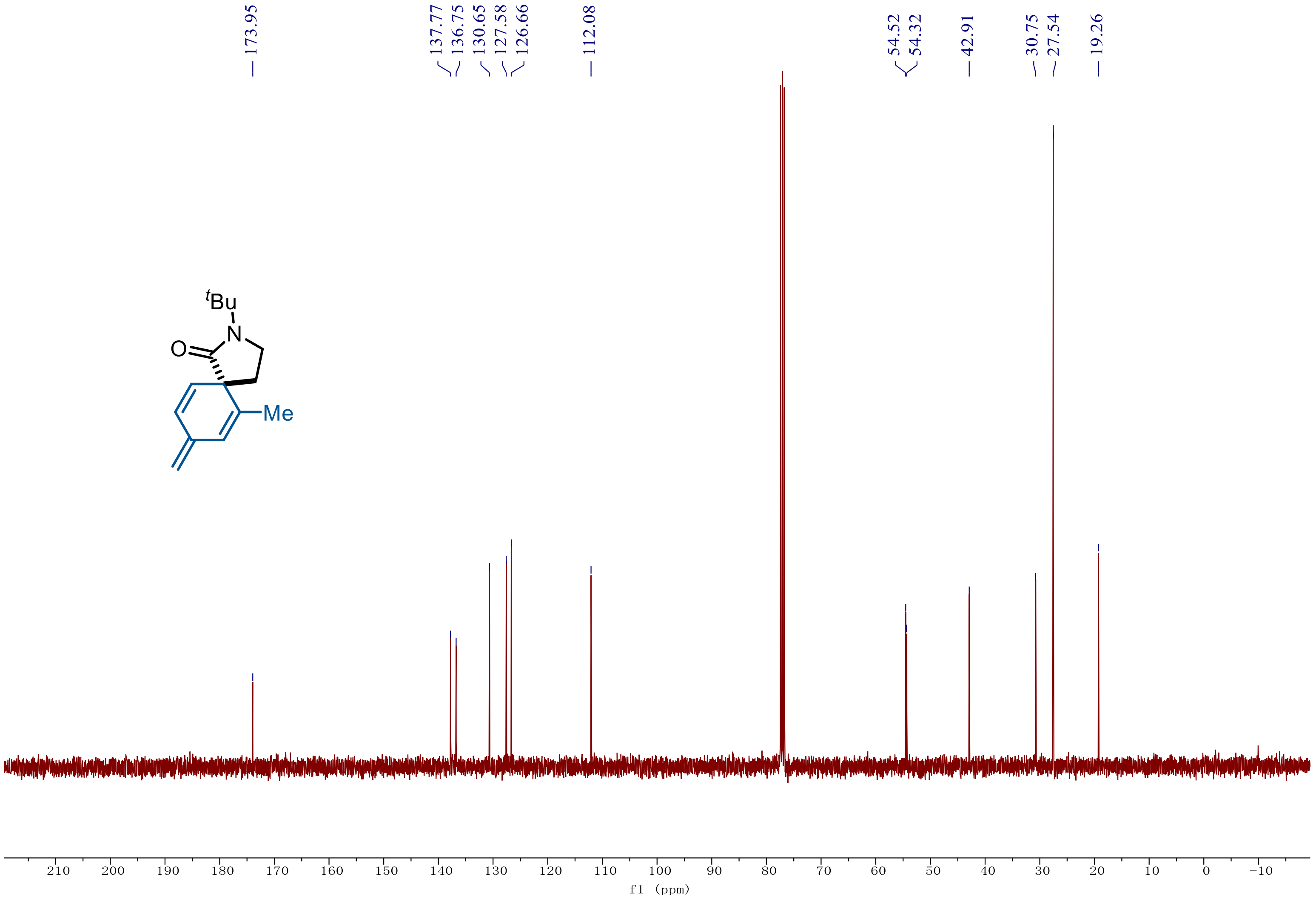

^{1}H NMR (500 MHz, $CDCl_3$) of **4i**

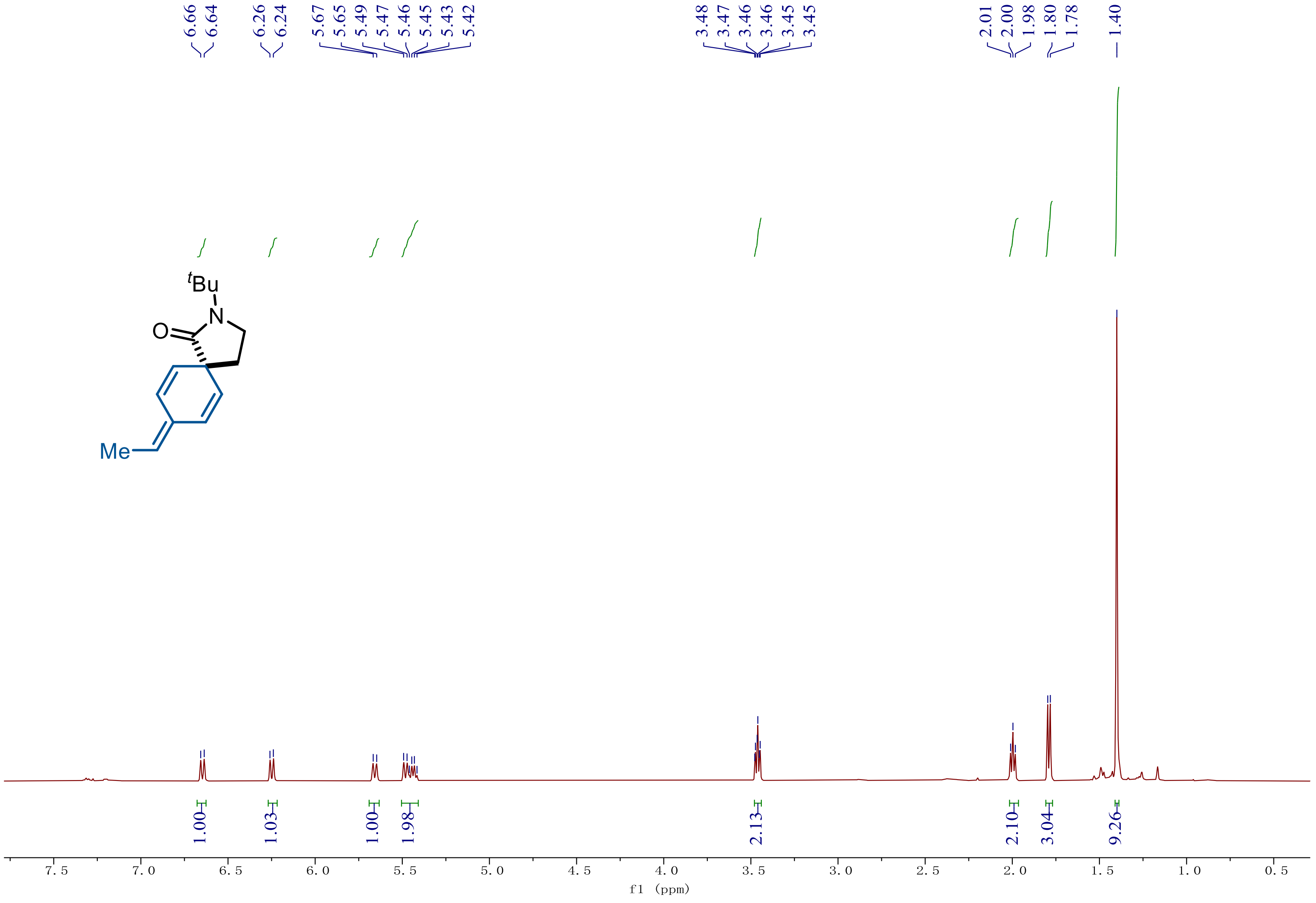

^{13}C NMR (126 MHz, $CDCl_3$) of **4i**

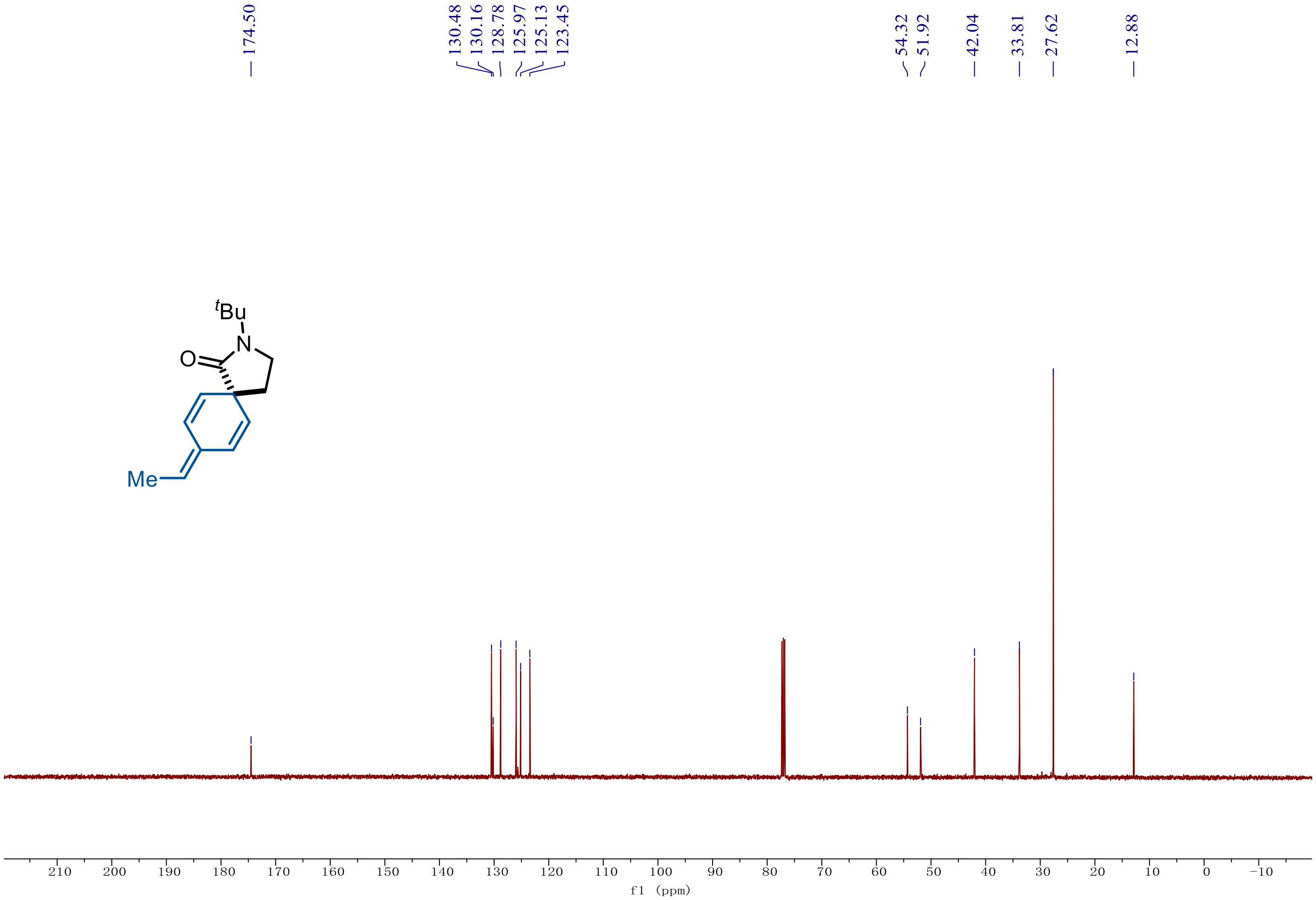

^1H NMR (500 MHz, $CDCl_3$) of **4j**

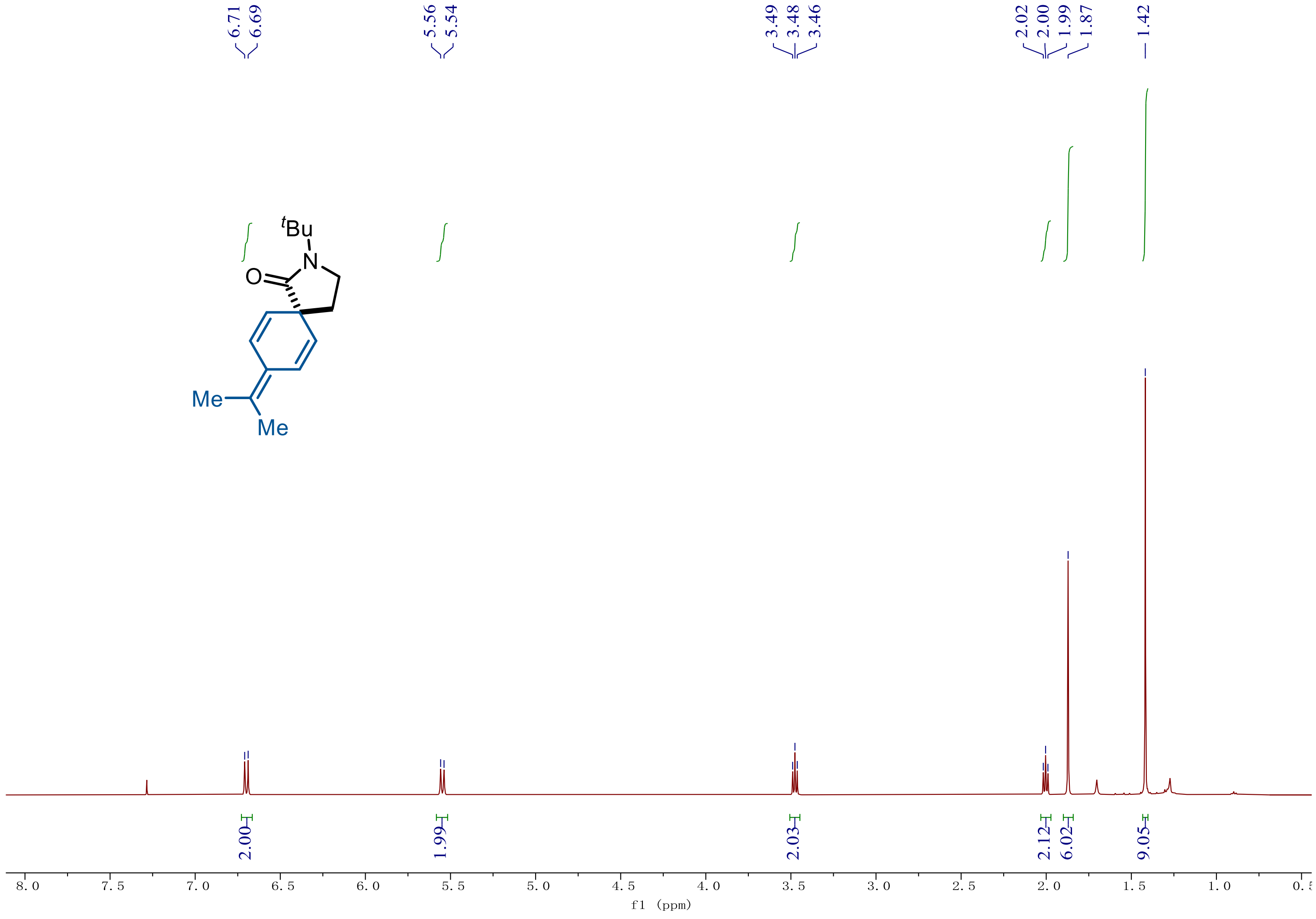

^{13}C NMR (126 MHz, $CDCl_3$) of **4j**

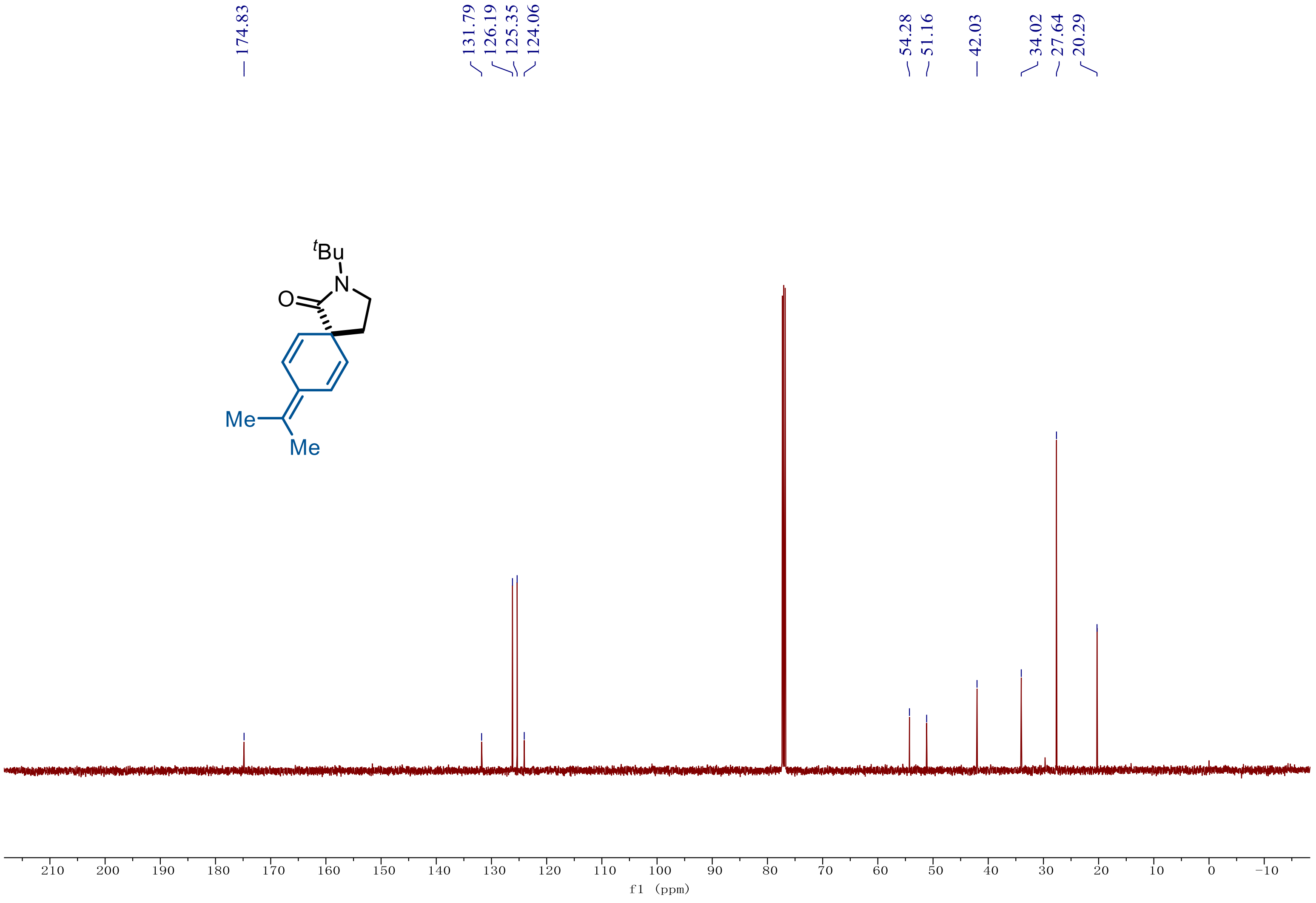

^{1}H NMR (500 MHz, $CDCl_3$) of **4k**

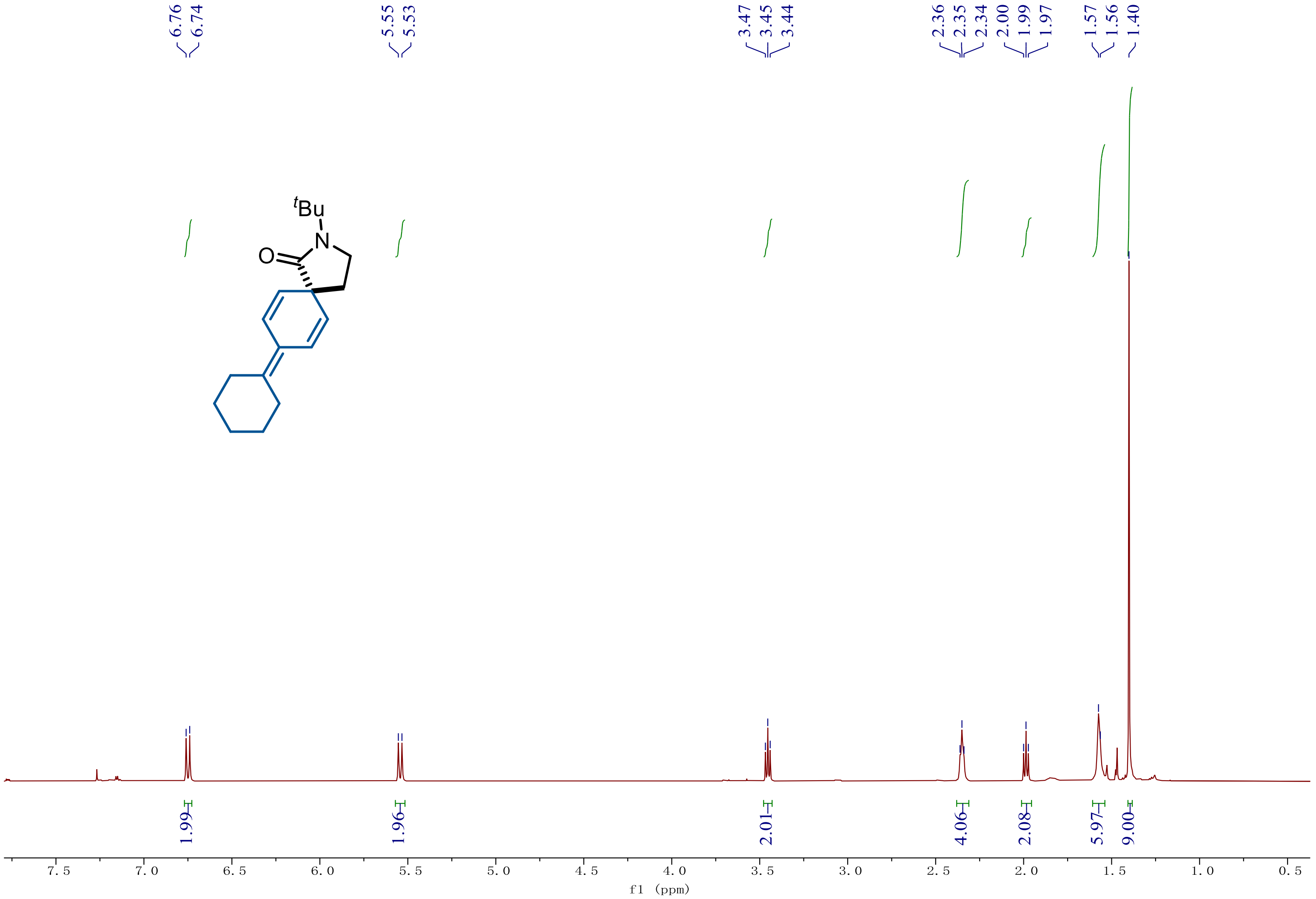

^{13}C NMR (126 MHz, $CDCl_3$) of **4k**

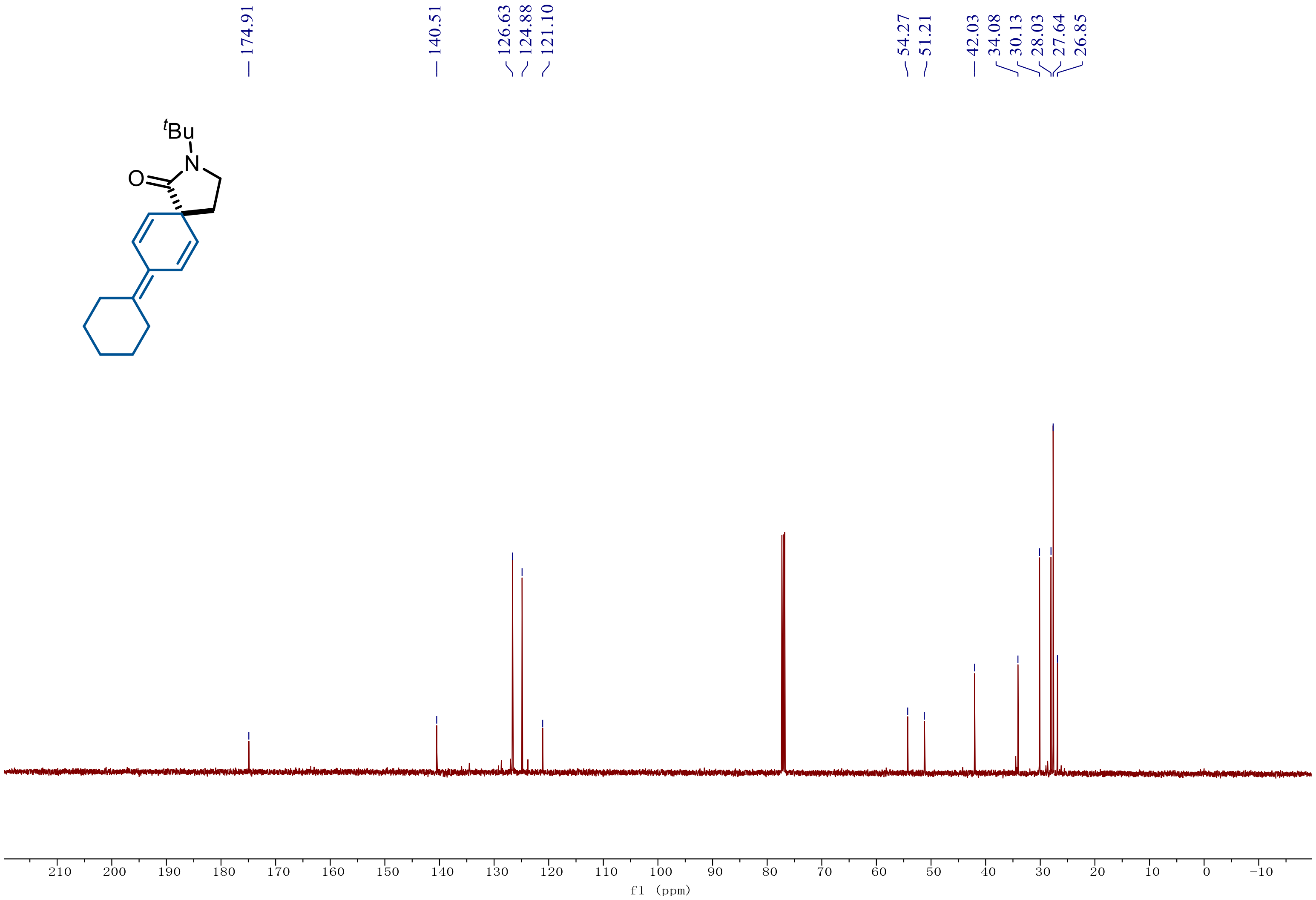

^{1}H NMR (500 MHz, $CDCl_3$) of **4l**

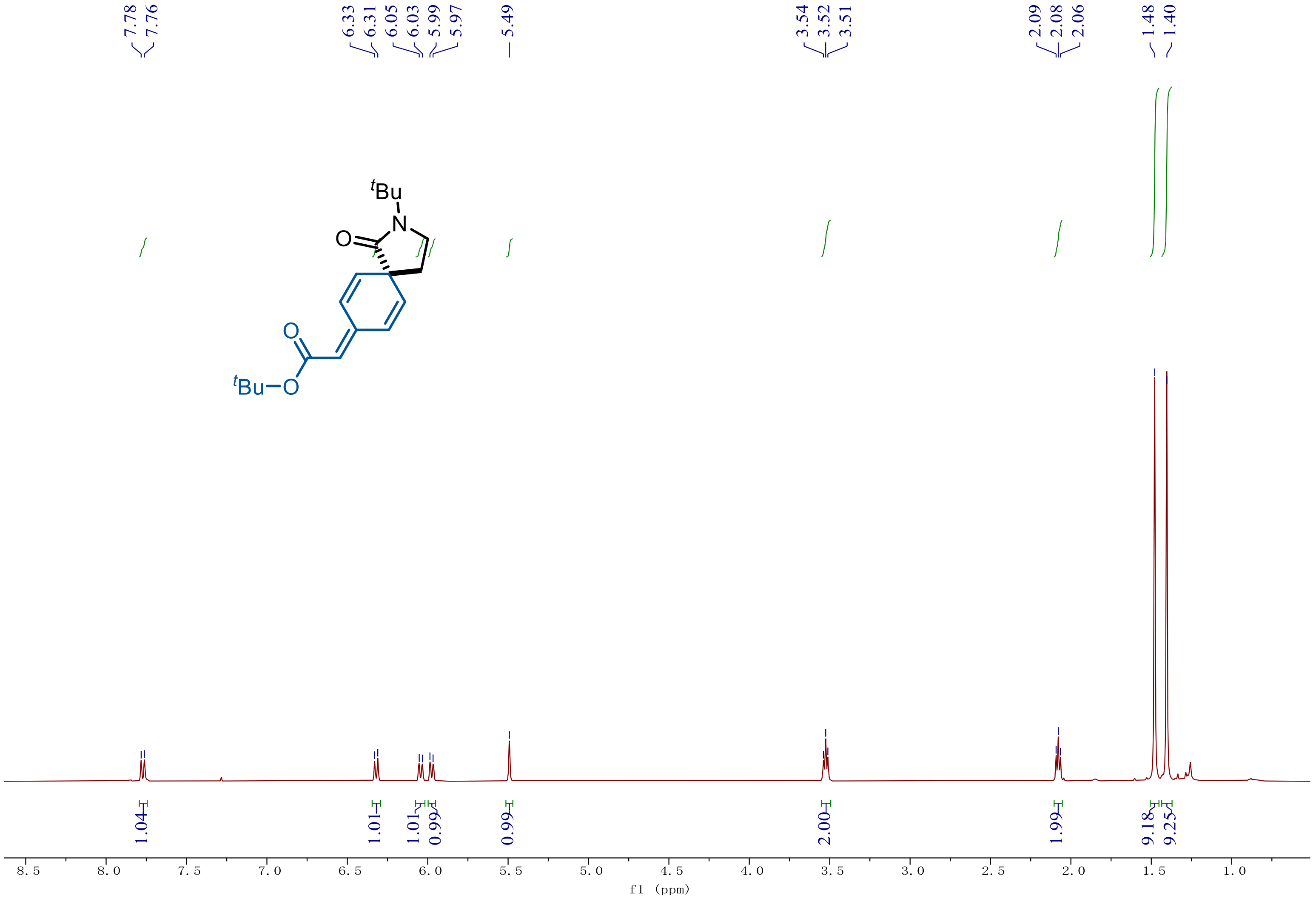

^{13}C NMR (126 MHz, $CDCl_3$) of **4l**

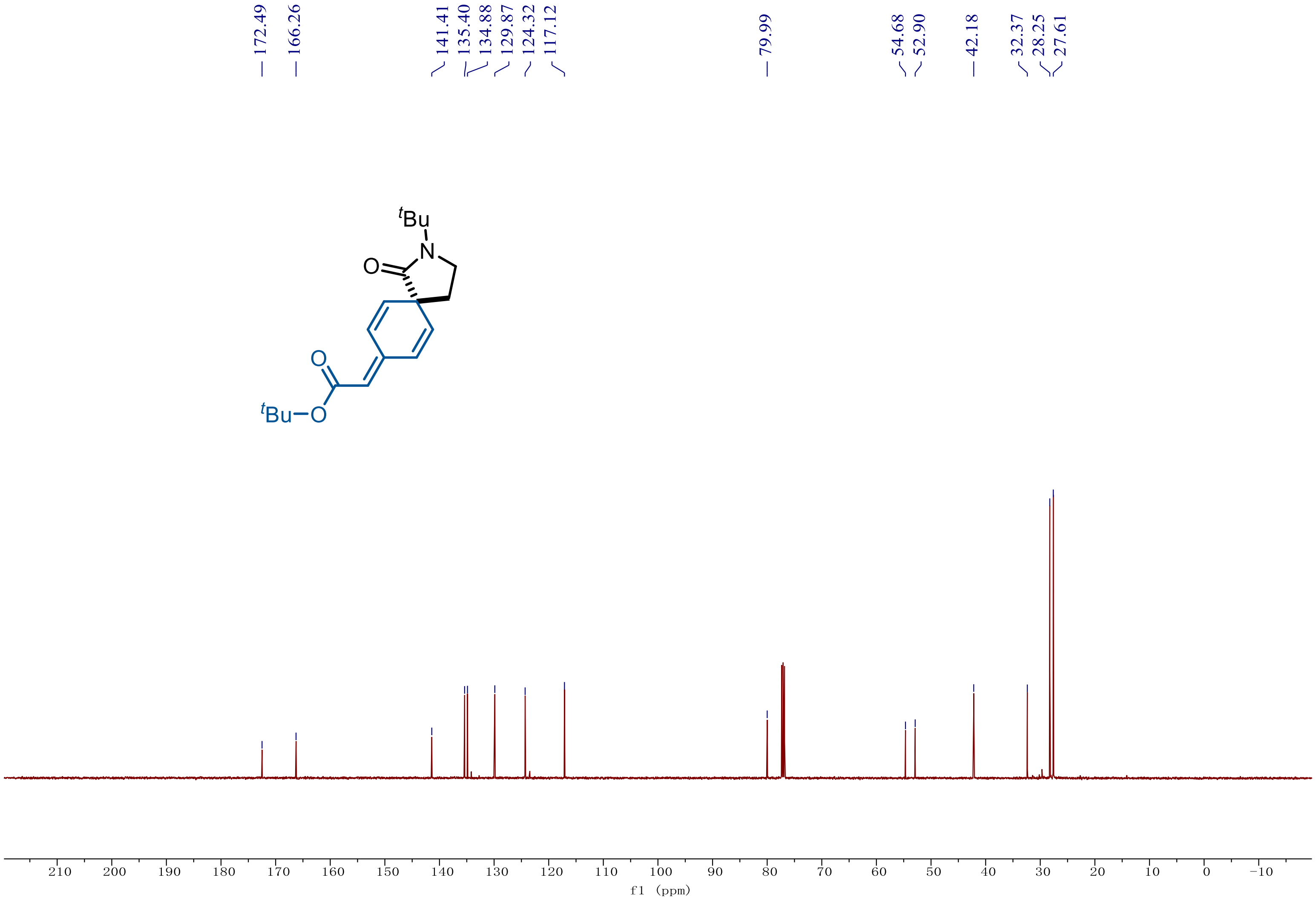

^{1}H NMR (400 MHz, CDCl$_3$) of **4m**

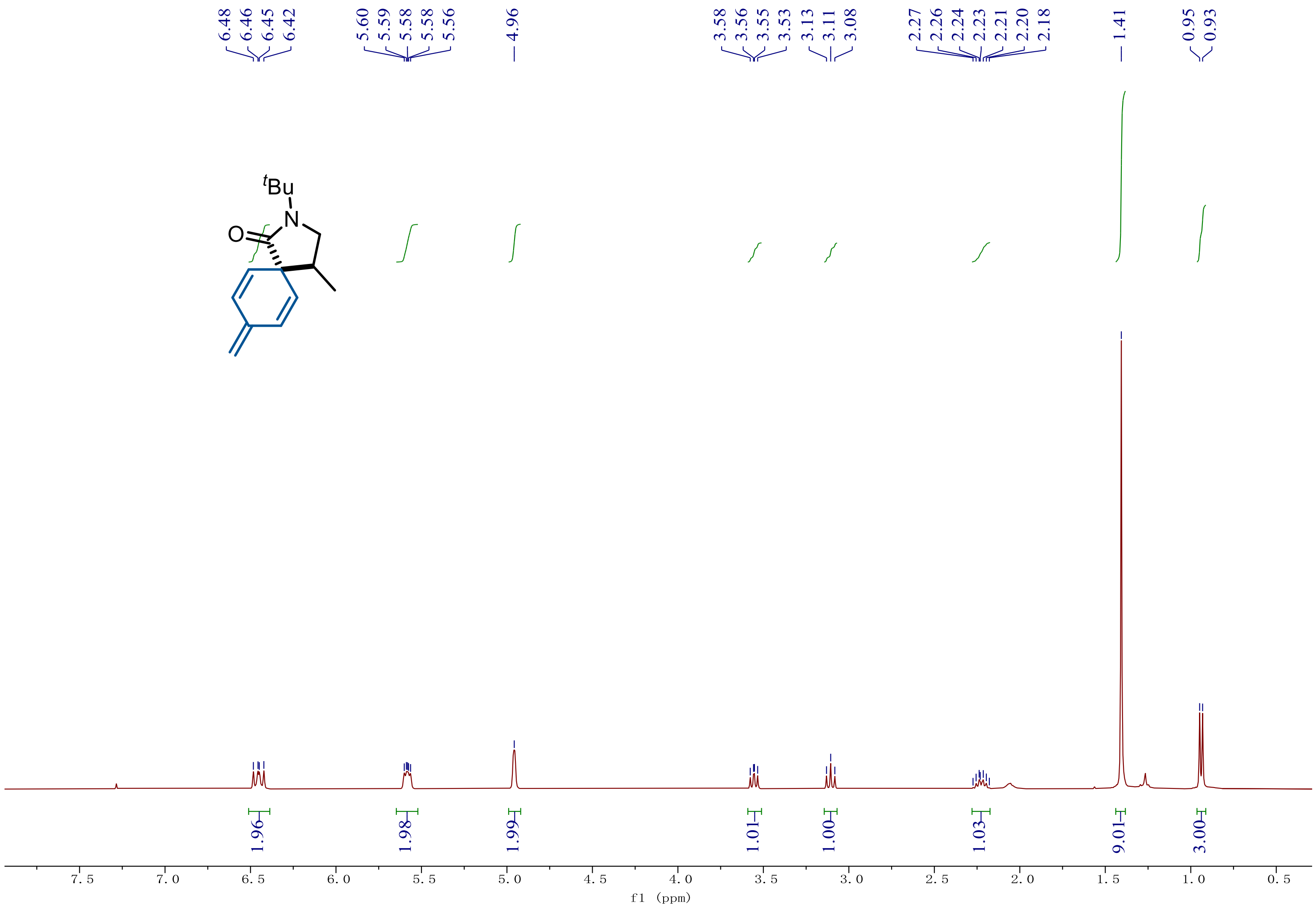

^{13}C NMR (101 MHz, $CDCl_3$) of **4m**

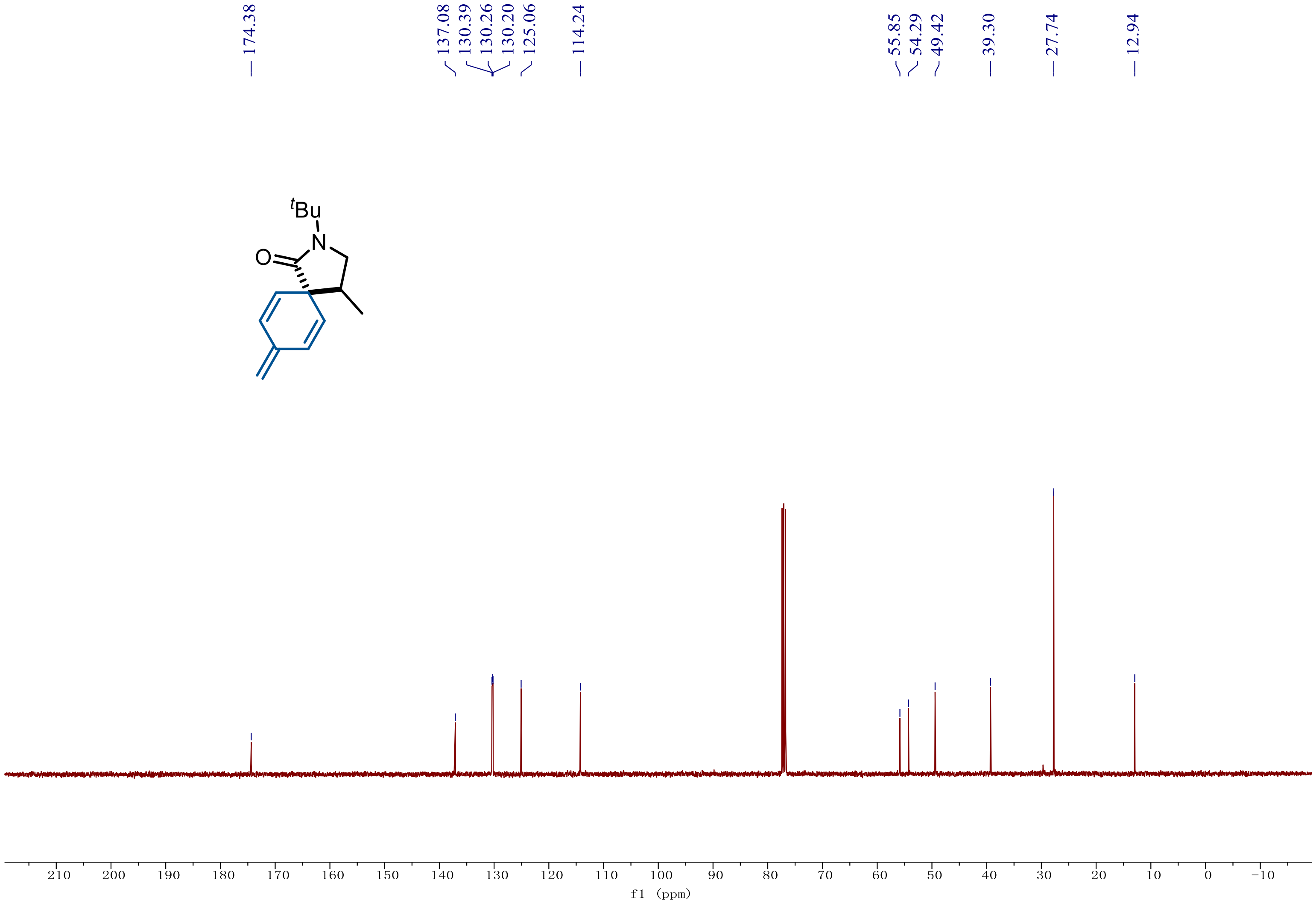

^{1}H NMR (400 MHz, $CDCl_3$) of **4n**

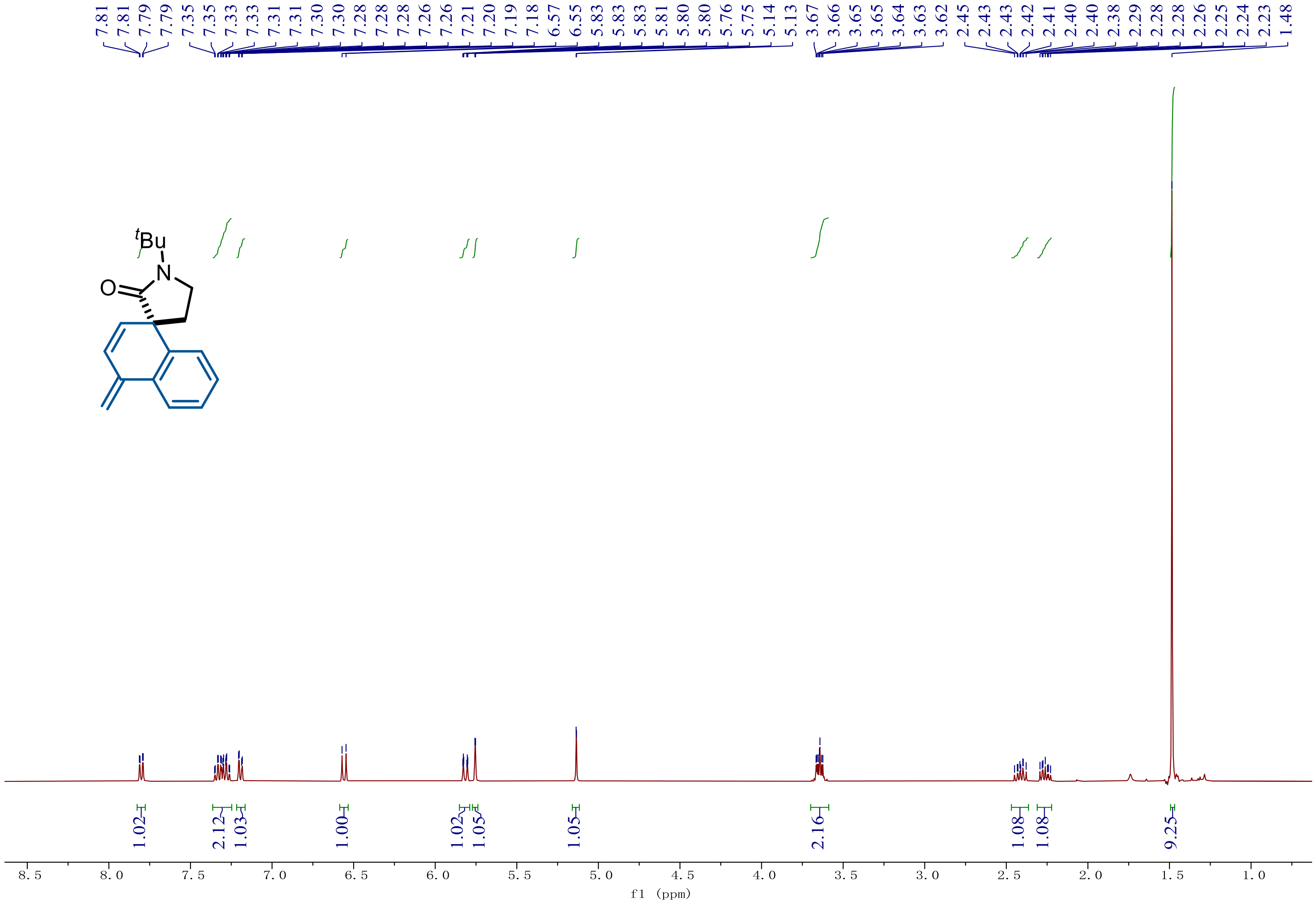

^{13}C NMR (101 MHz, $CDCl_3$) of **4n**

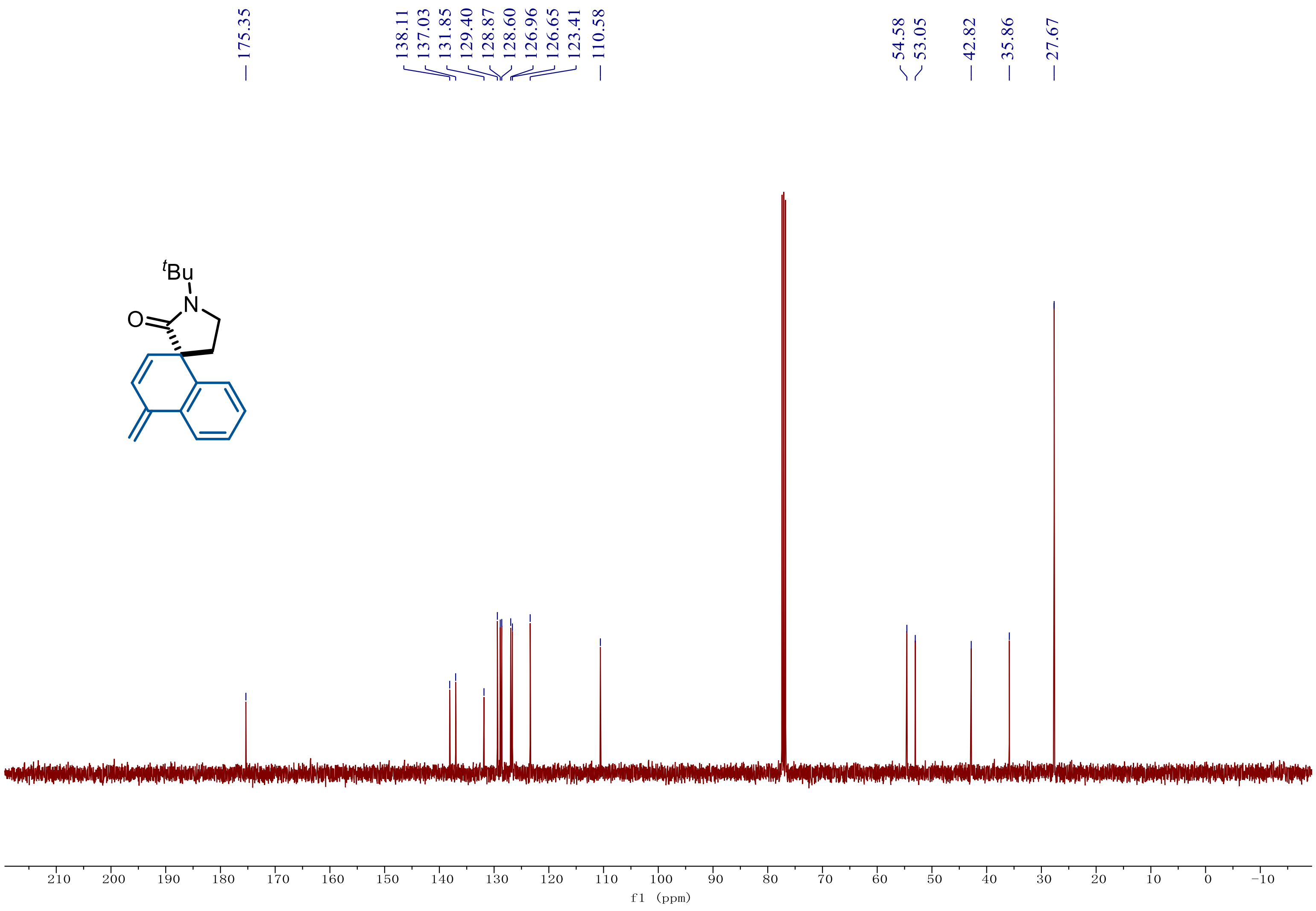

^{1}H NMR (500 MHz, CDCl$_3$) of **4o**

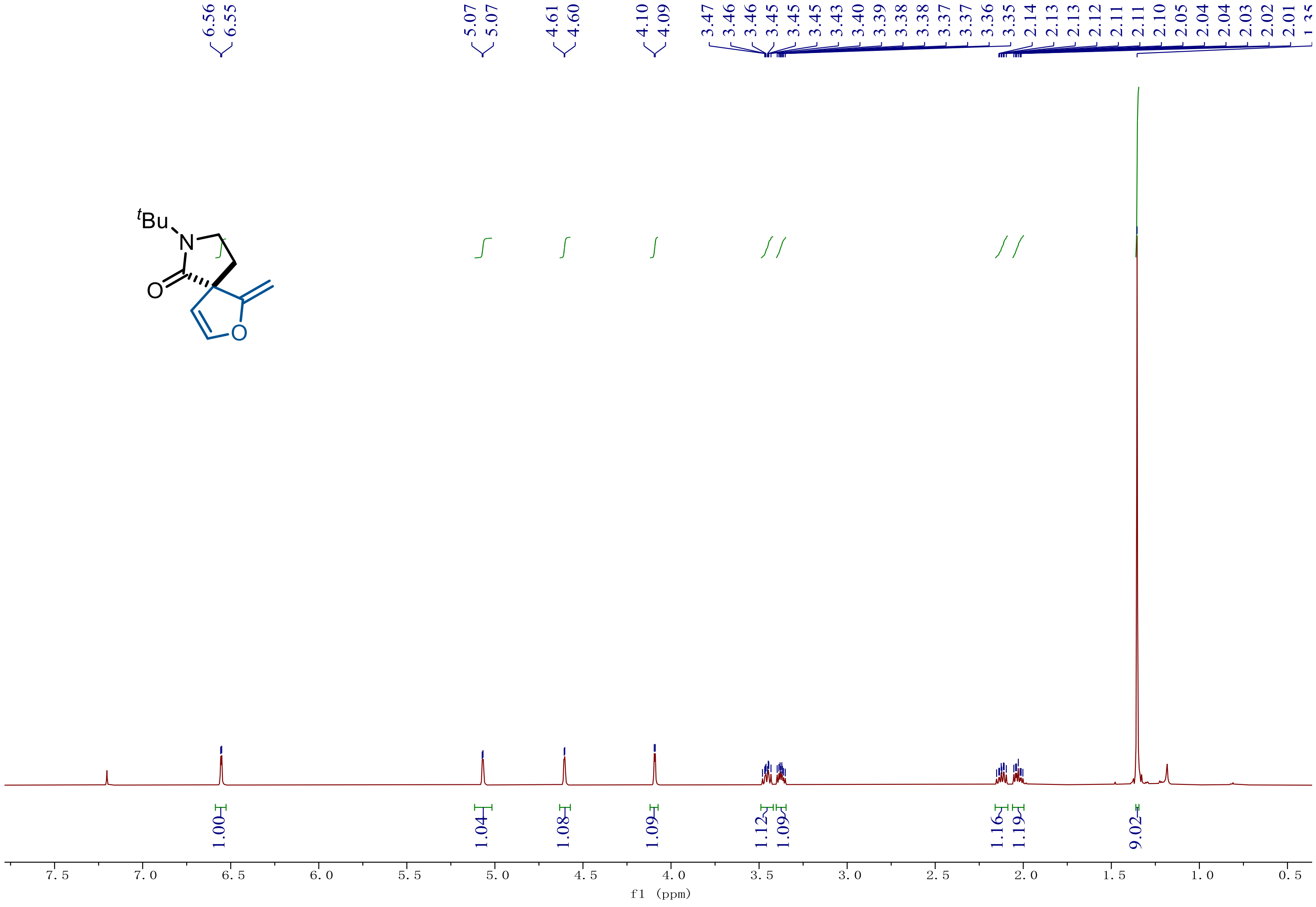

^{13}C NMR (126 MHz, CDCl$_3$) of **4o**

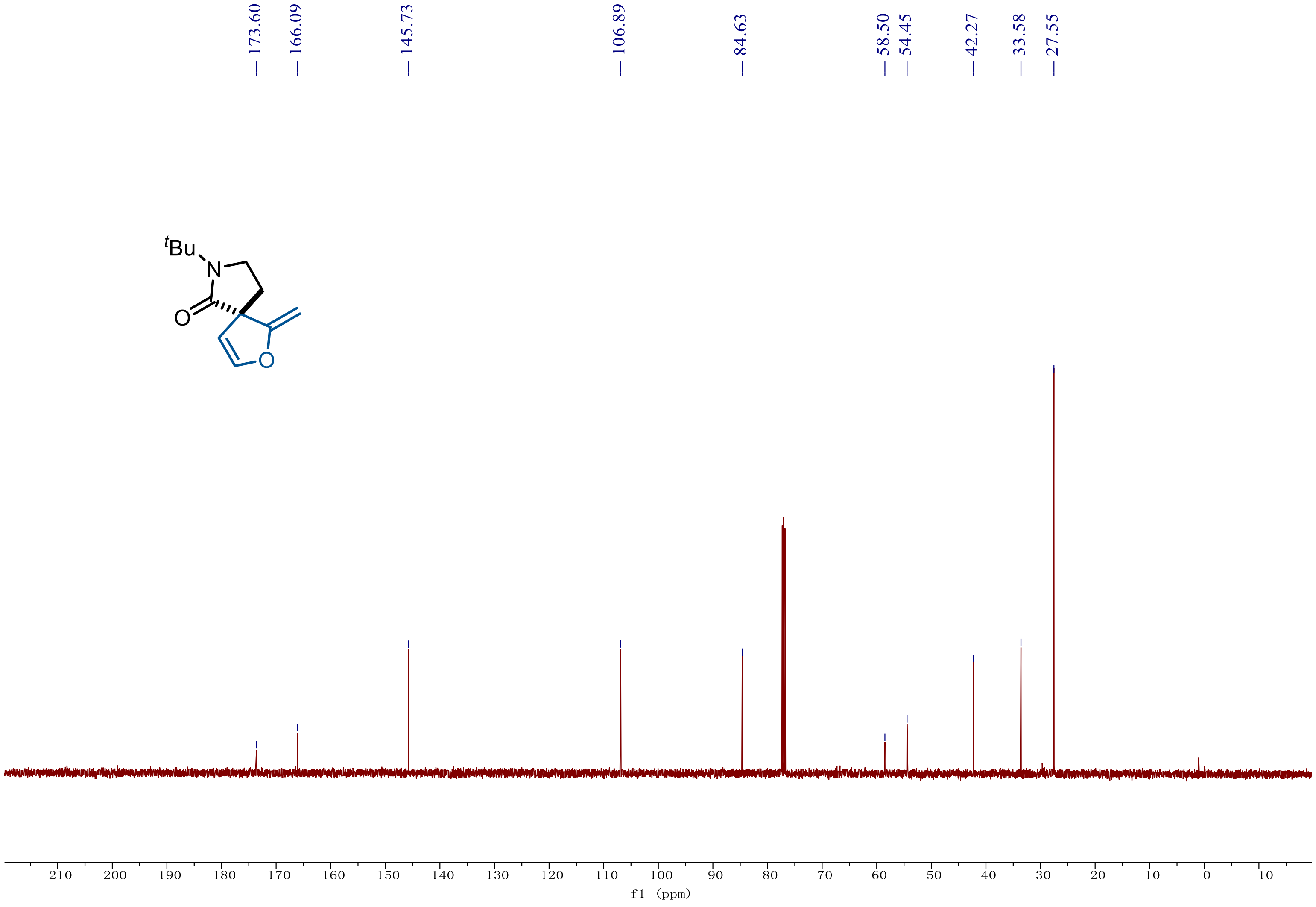

^{1}H NMR (400 MHz, $CDCl_3$) of **4p**

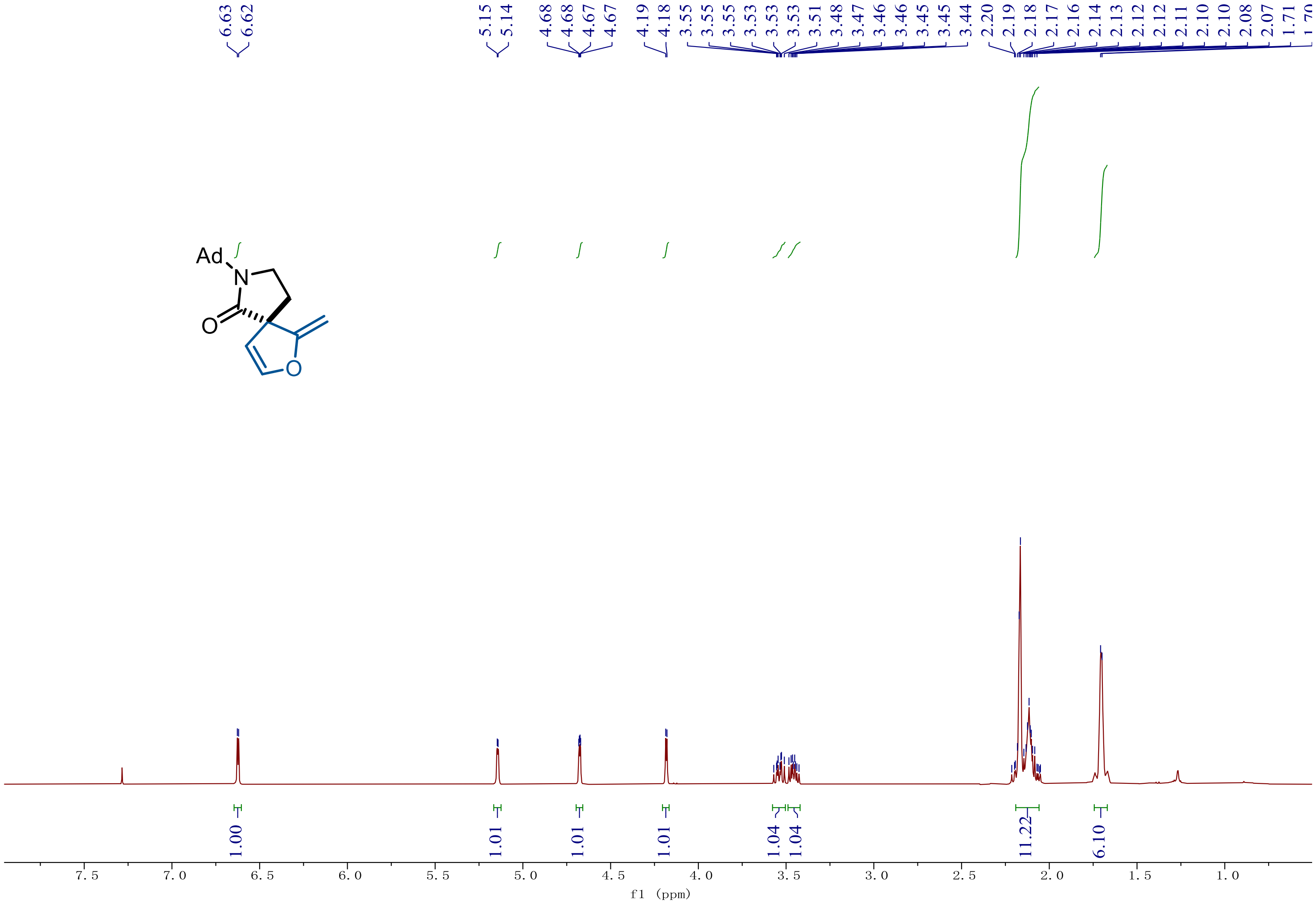

^{13}C NMR (101 MHz, $CDCl_3$) of **4p**

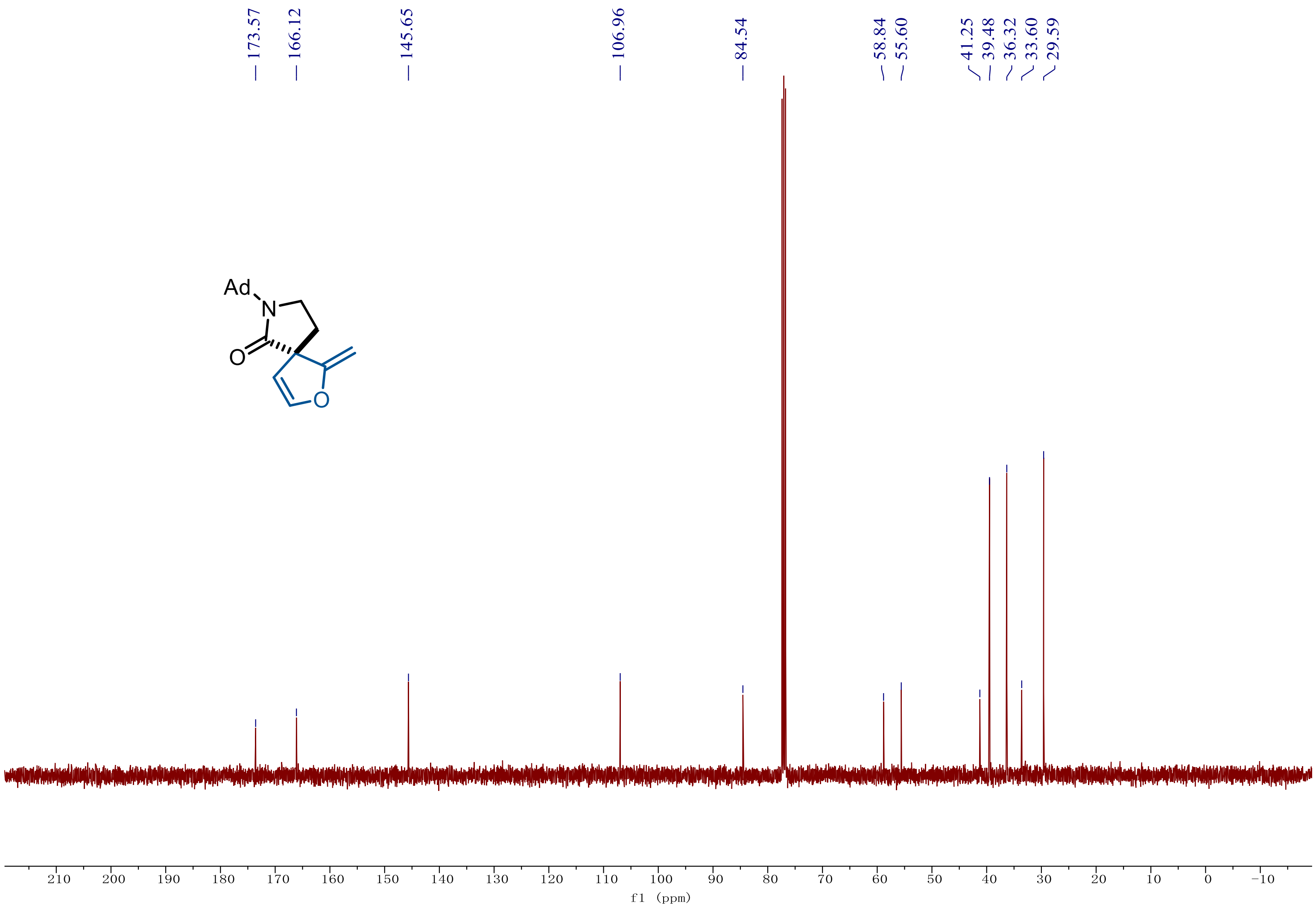

^{1}H NMR (500 MHz, CDCl$_3$) of **4q**

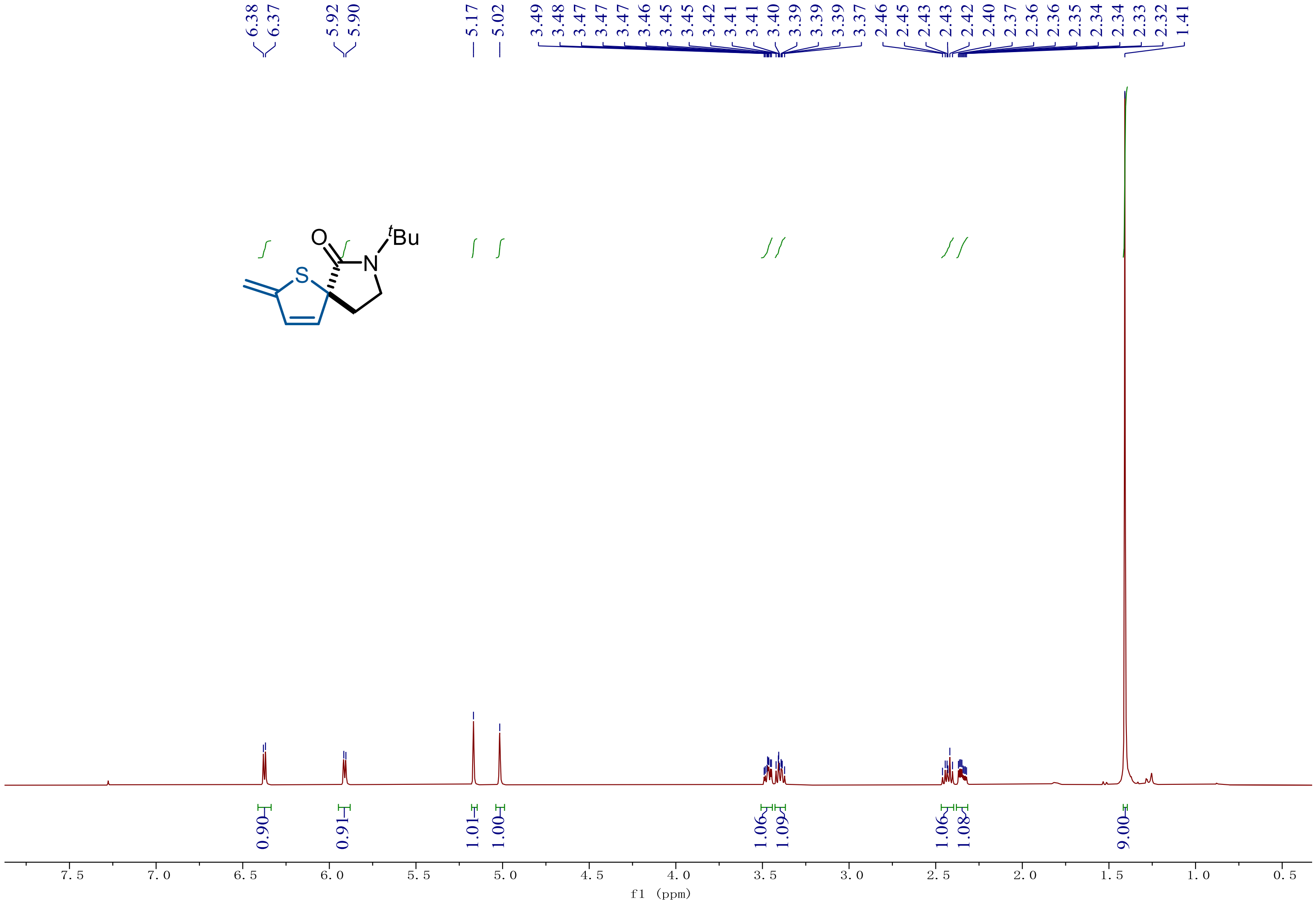

^{13}C NMR (126 MHz, $CDCl_3$) of **4q**

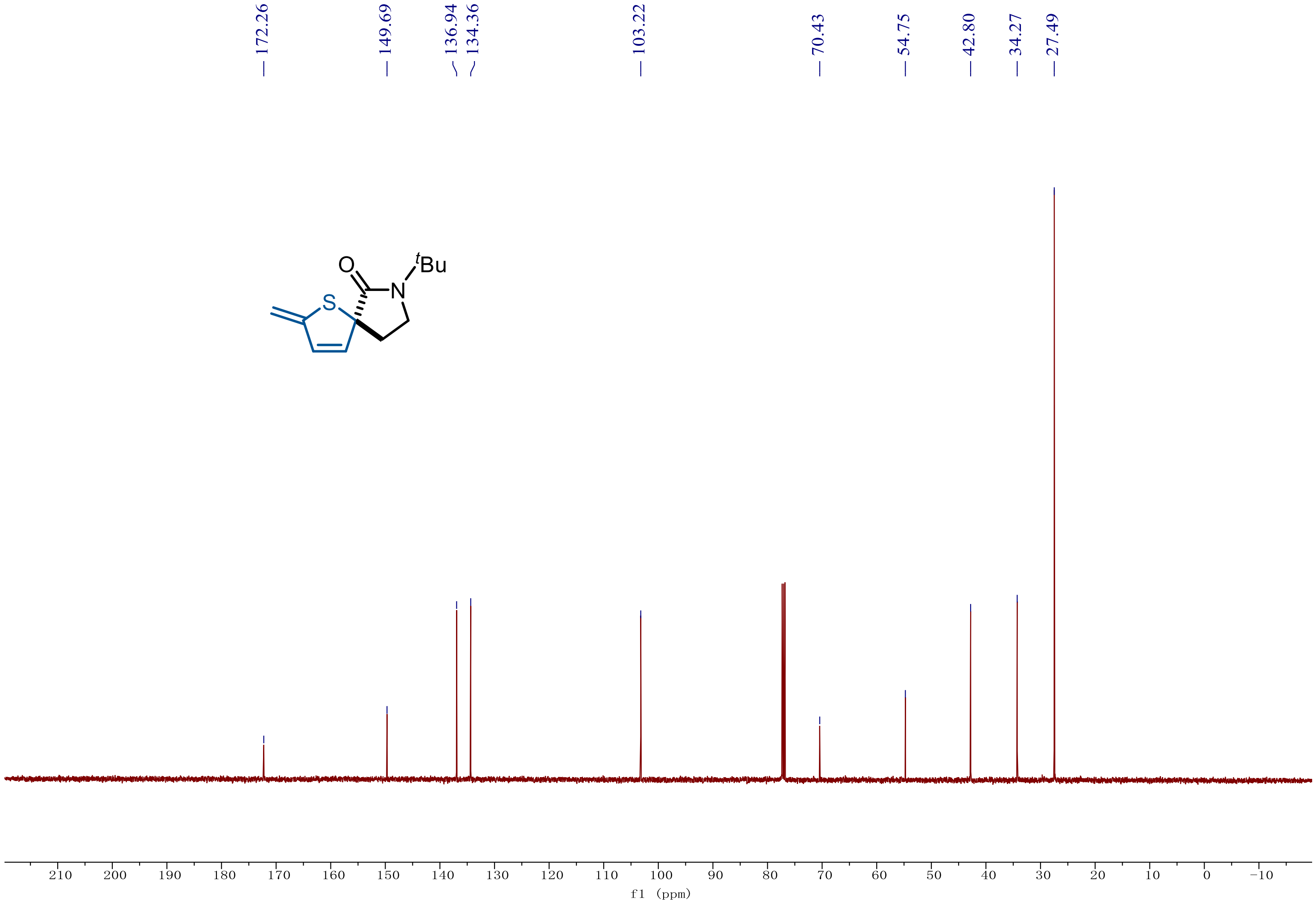

^{1}H NMR (500 MHz, $CDCl_3$) of **4r**

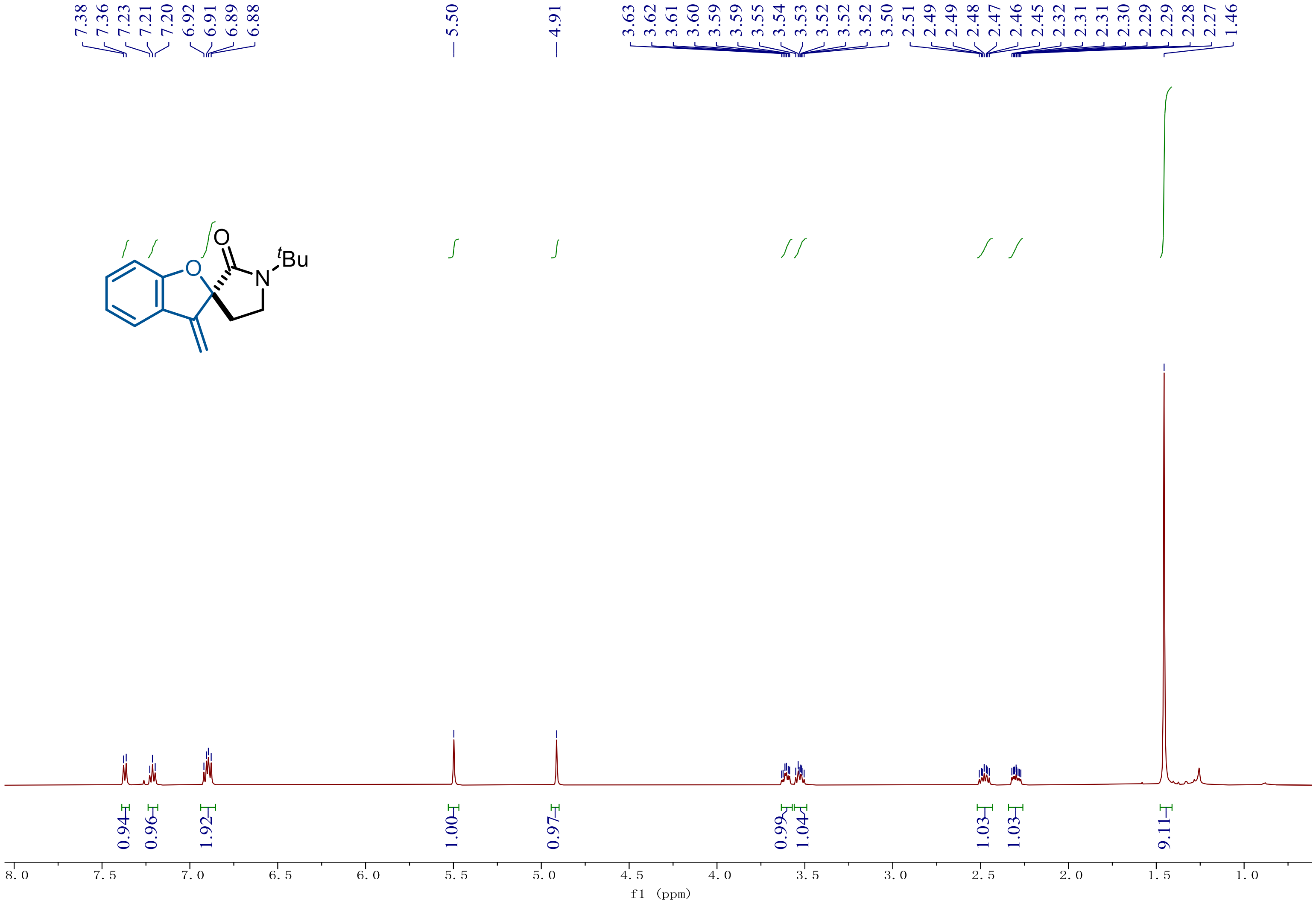

^{13}C NMR (126 MHz, $CDCl_3$) of **4r**

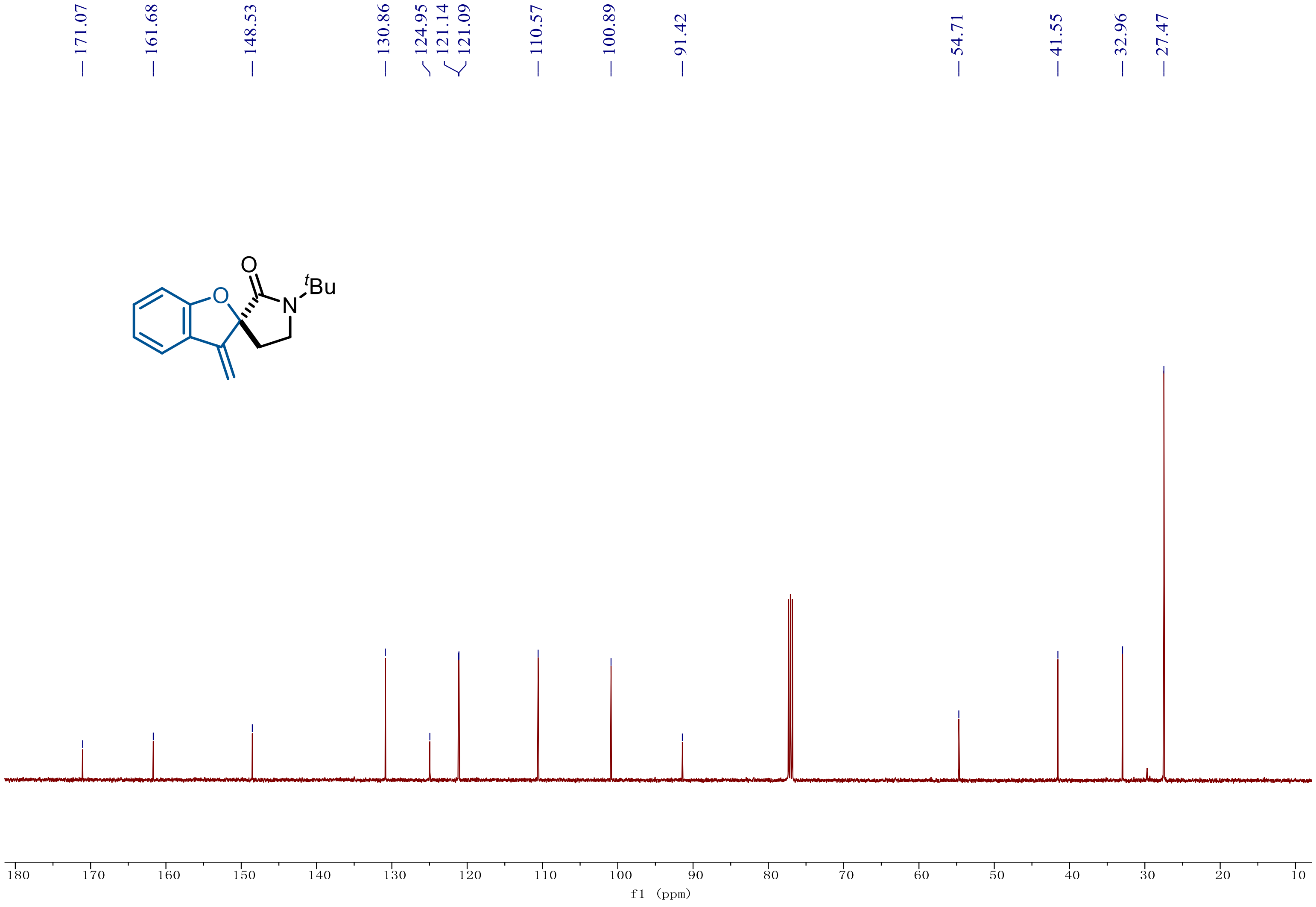

^1H NMR (400 MHz, $CDCl_3$) of **5**

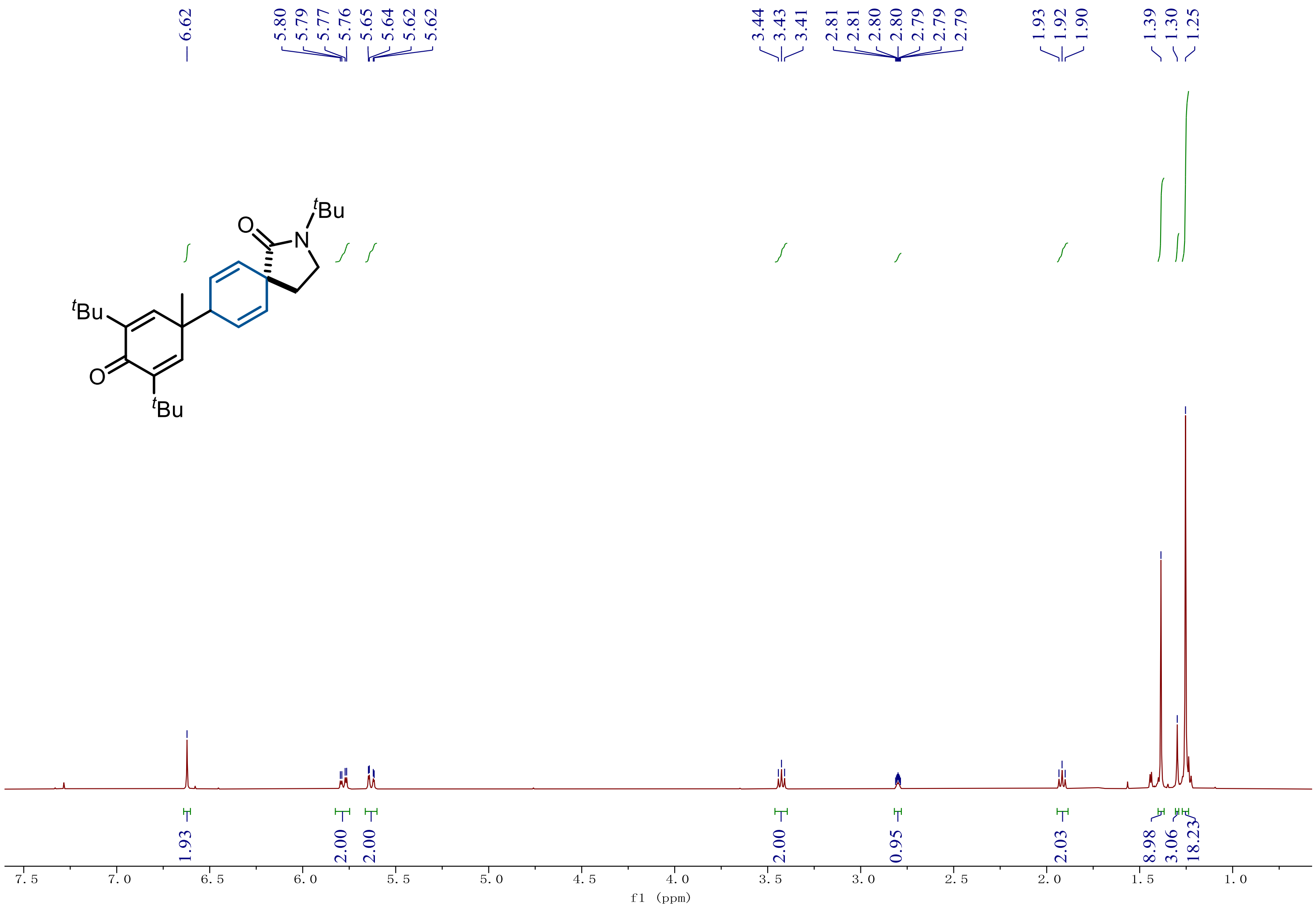

^{13}C NMR (101 MHz, $CDCl_3$) of **5**

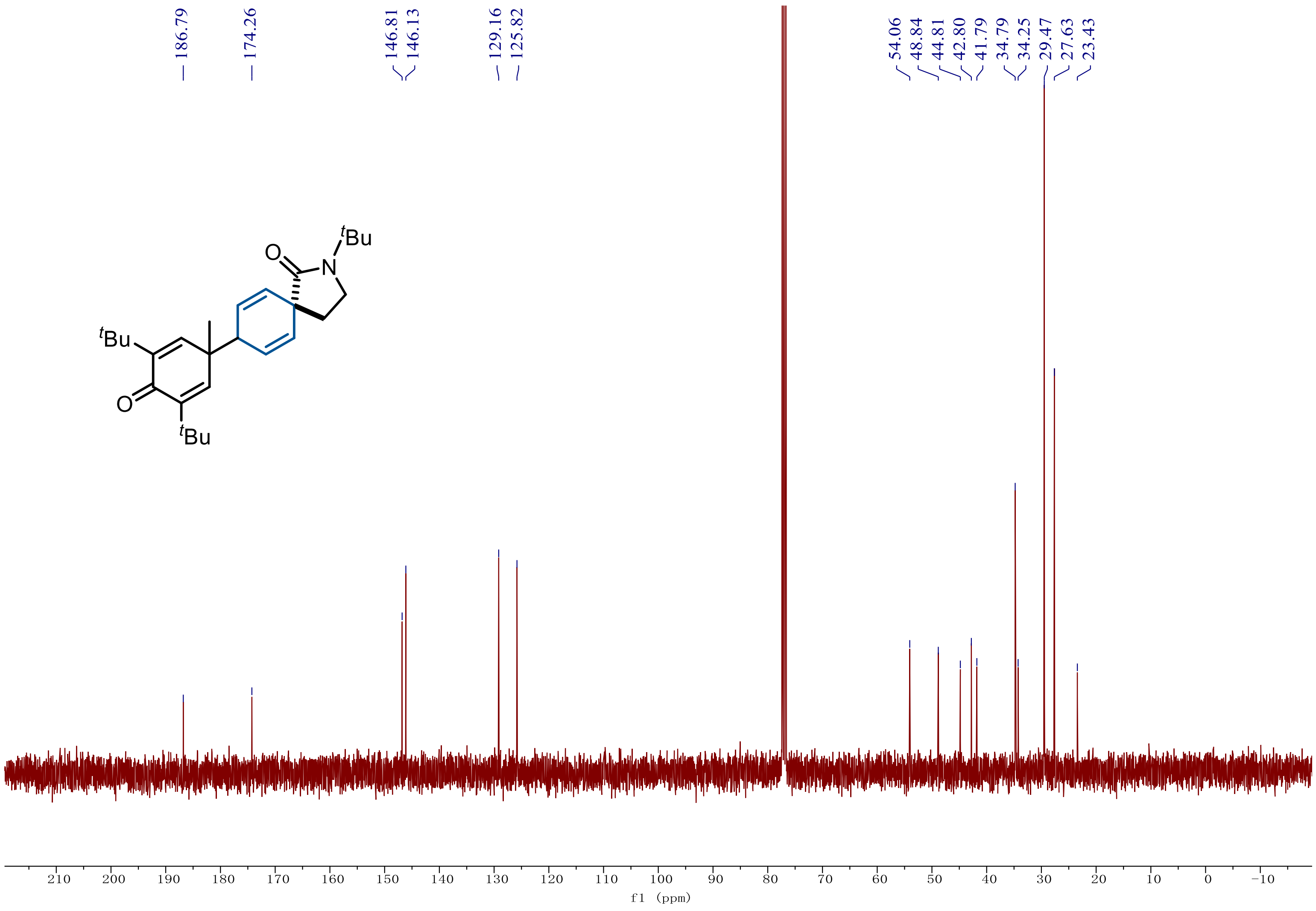

^{1}H NMR (400 MHz, $CDCl_3$) of **8**

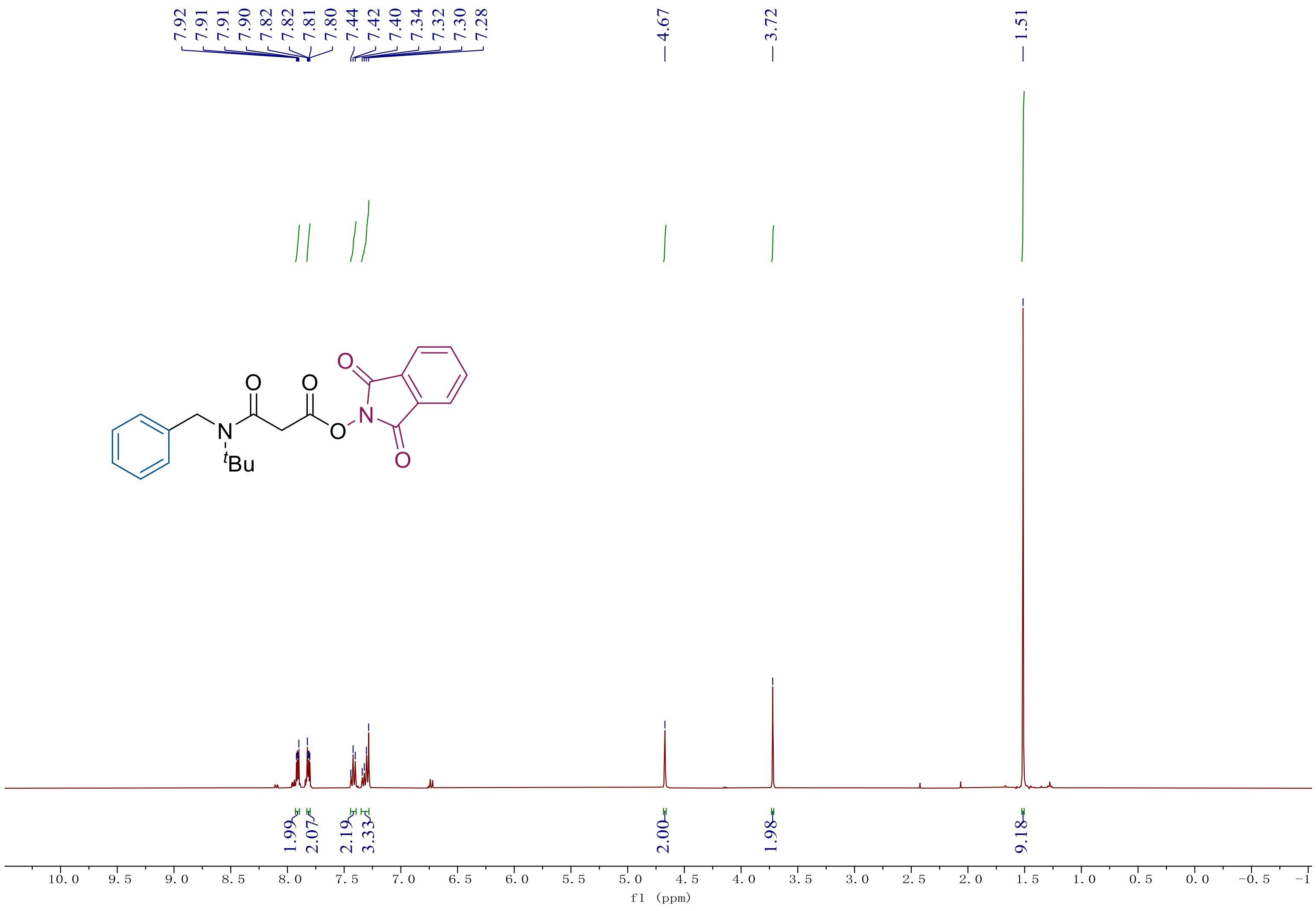

^{13}C NMR (101 MHz, $CDCl_3$) of **8**

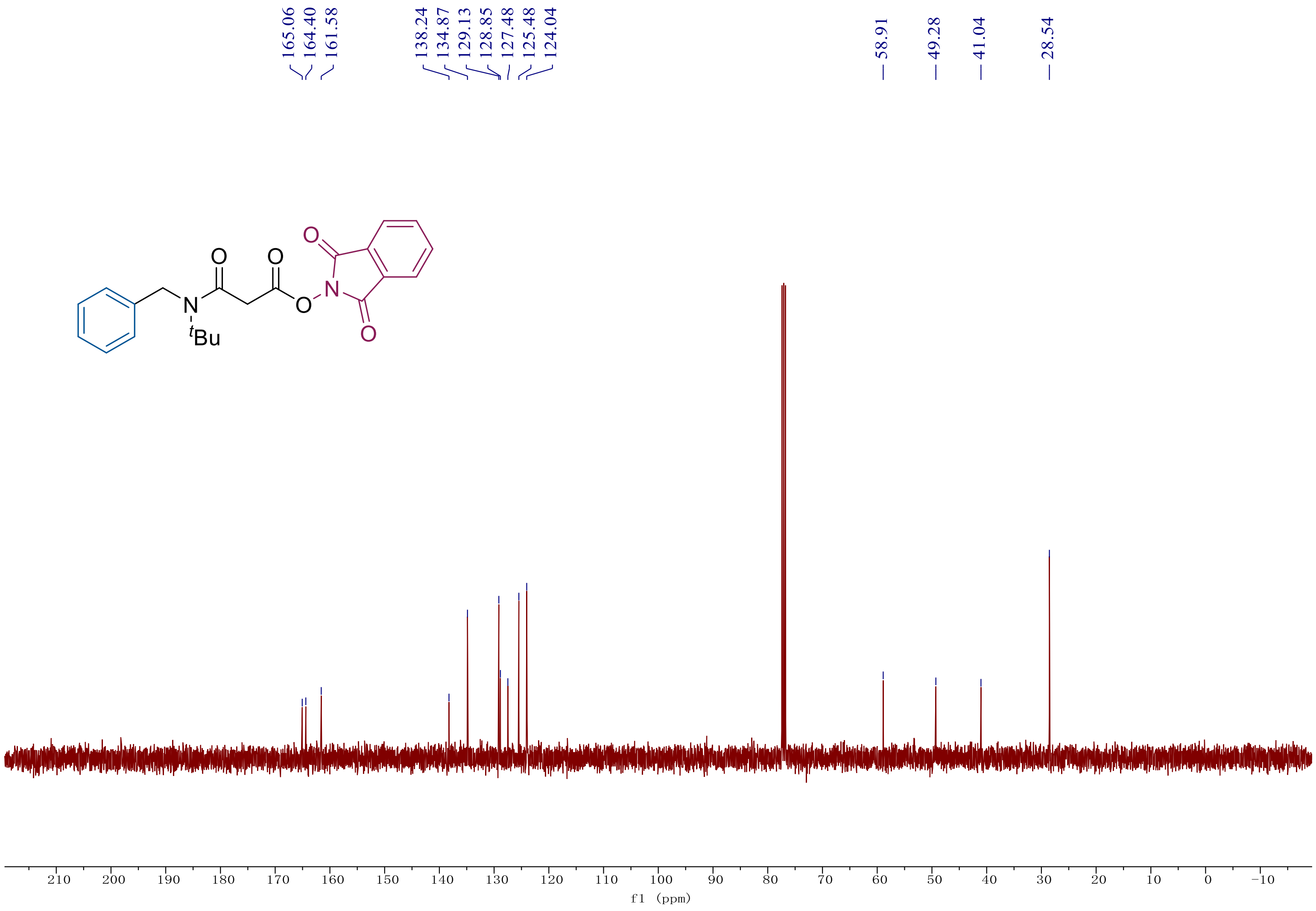

^{1}H NMR (500 MHz, $CDCl_3$) of **9**

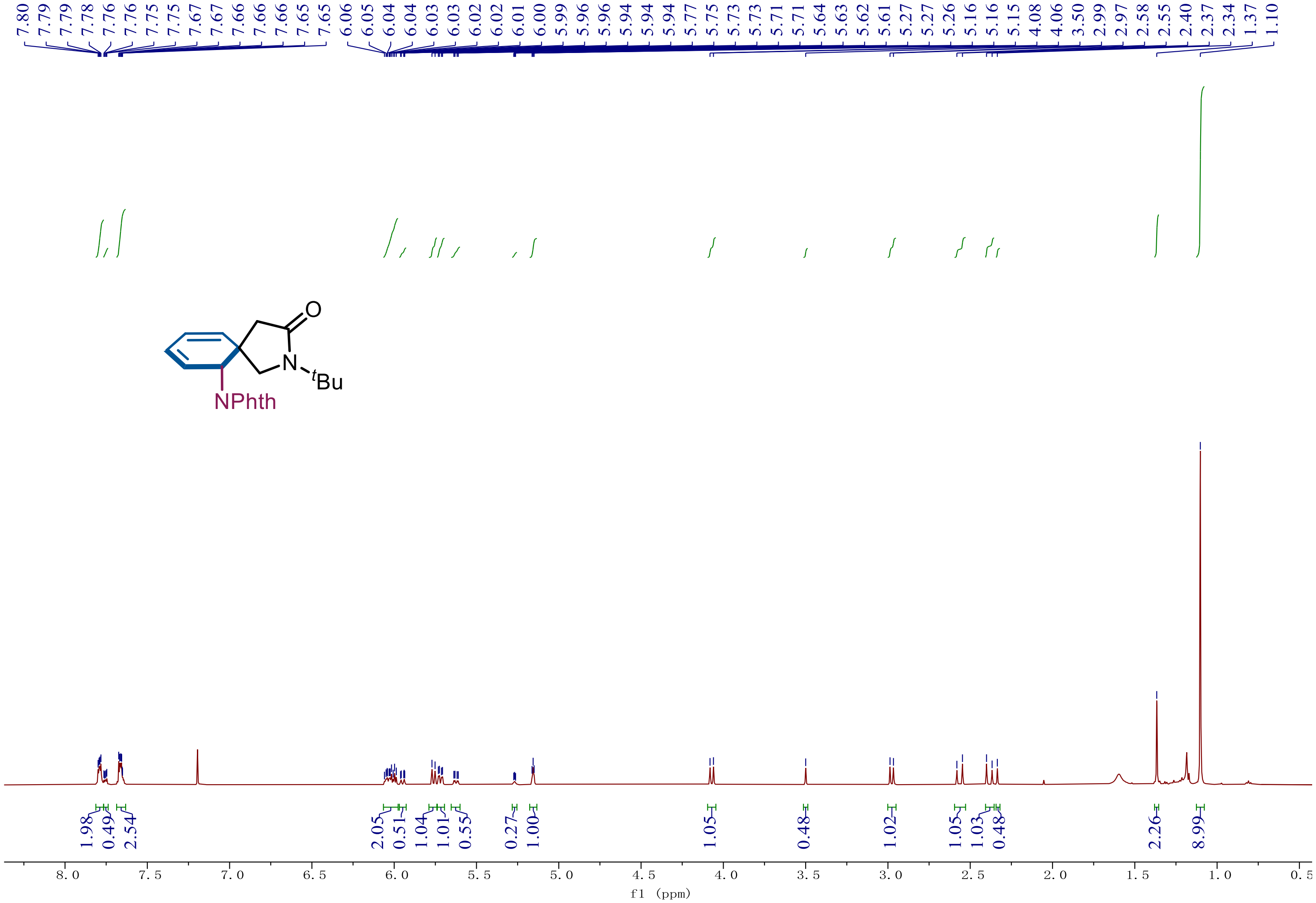

^{13}C NMR (126 MHz, $CDCl_3$) of **9**

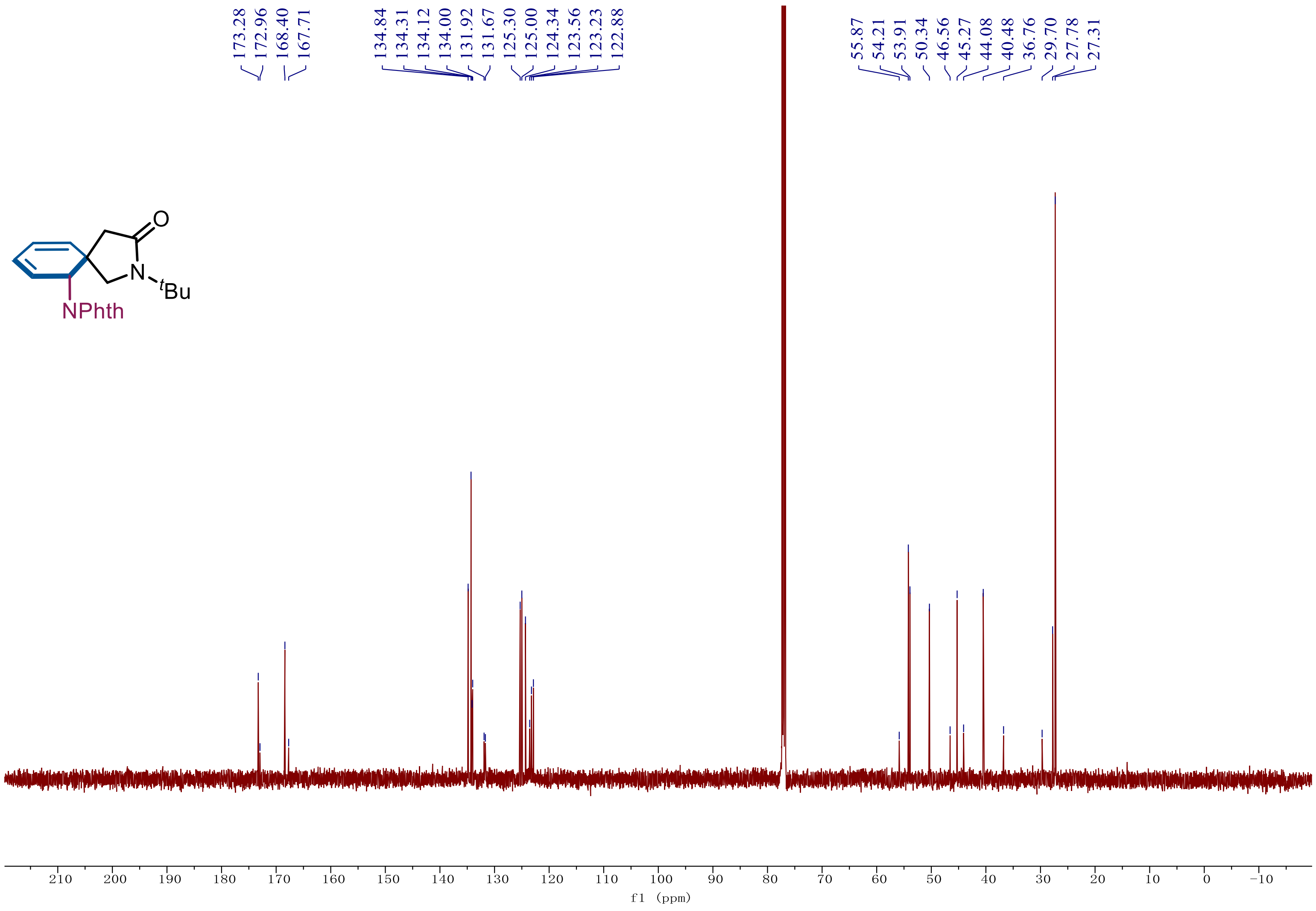

NOE of **9**

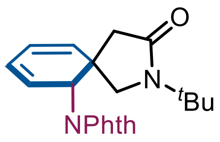

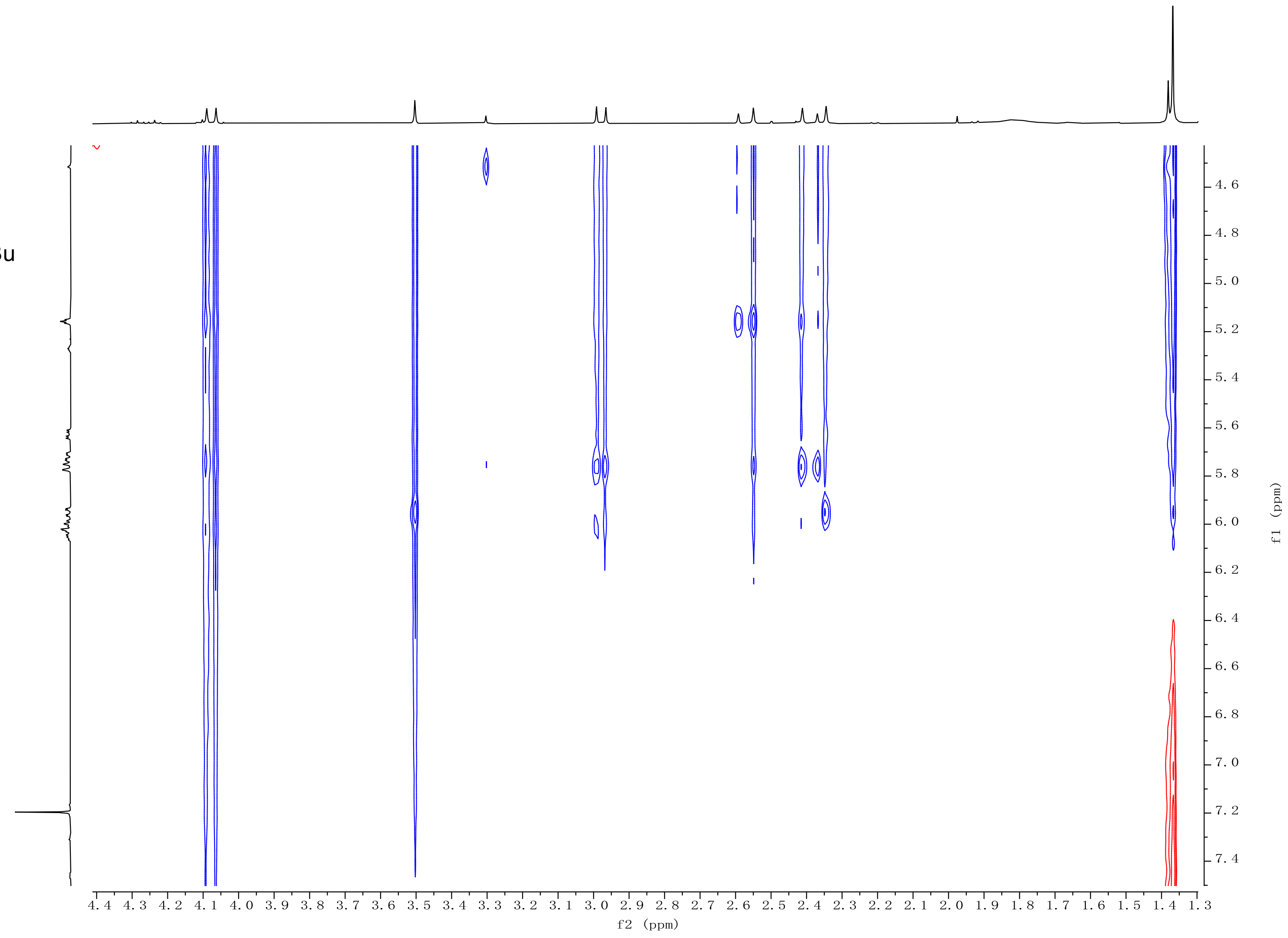

^{1}H NMR (400 MHz, $CDCl_3$) of **10**

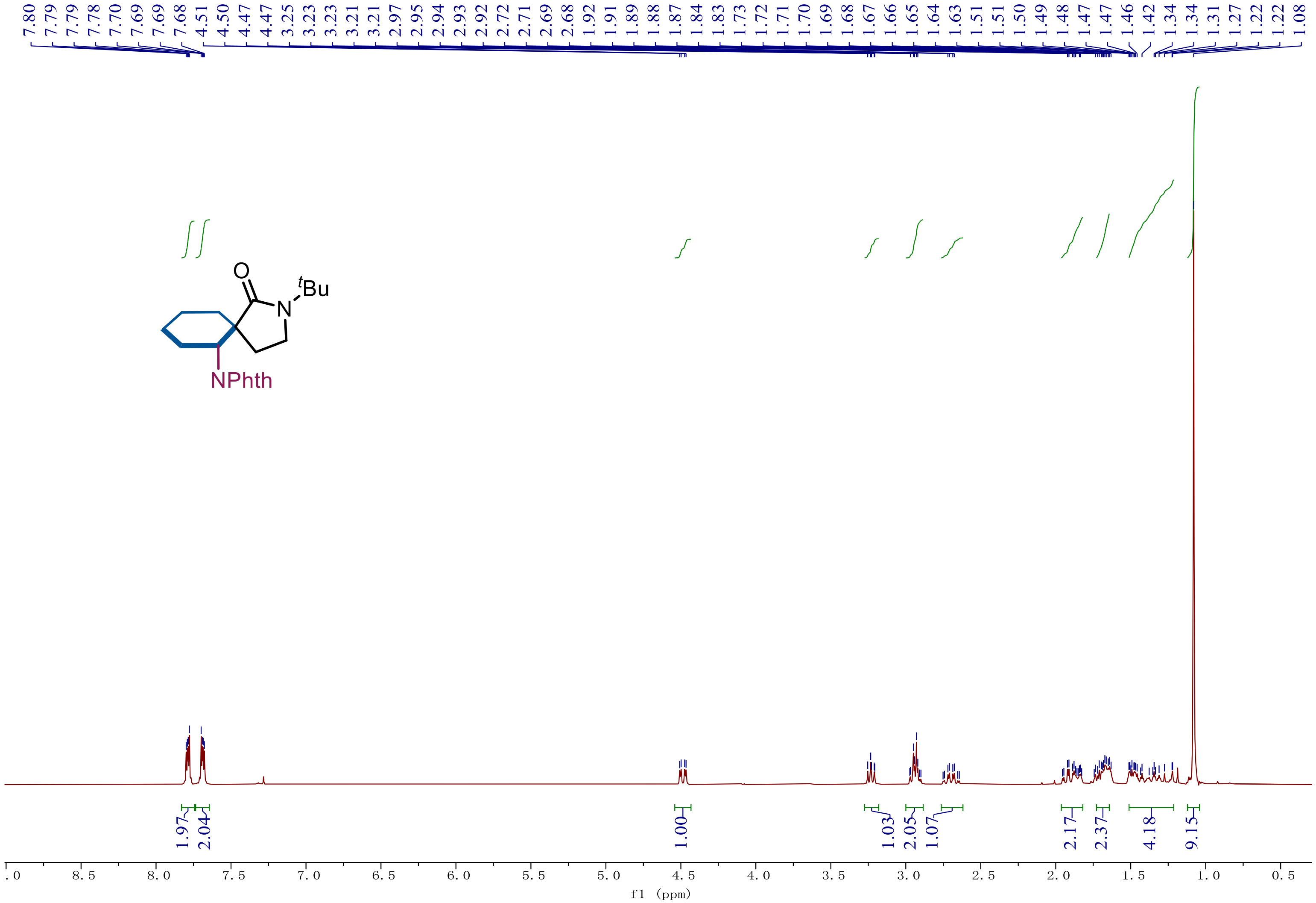

^{13}C NMR (101 MHz, $CDCl_3$) of **10**

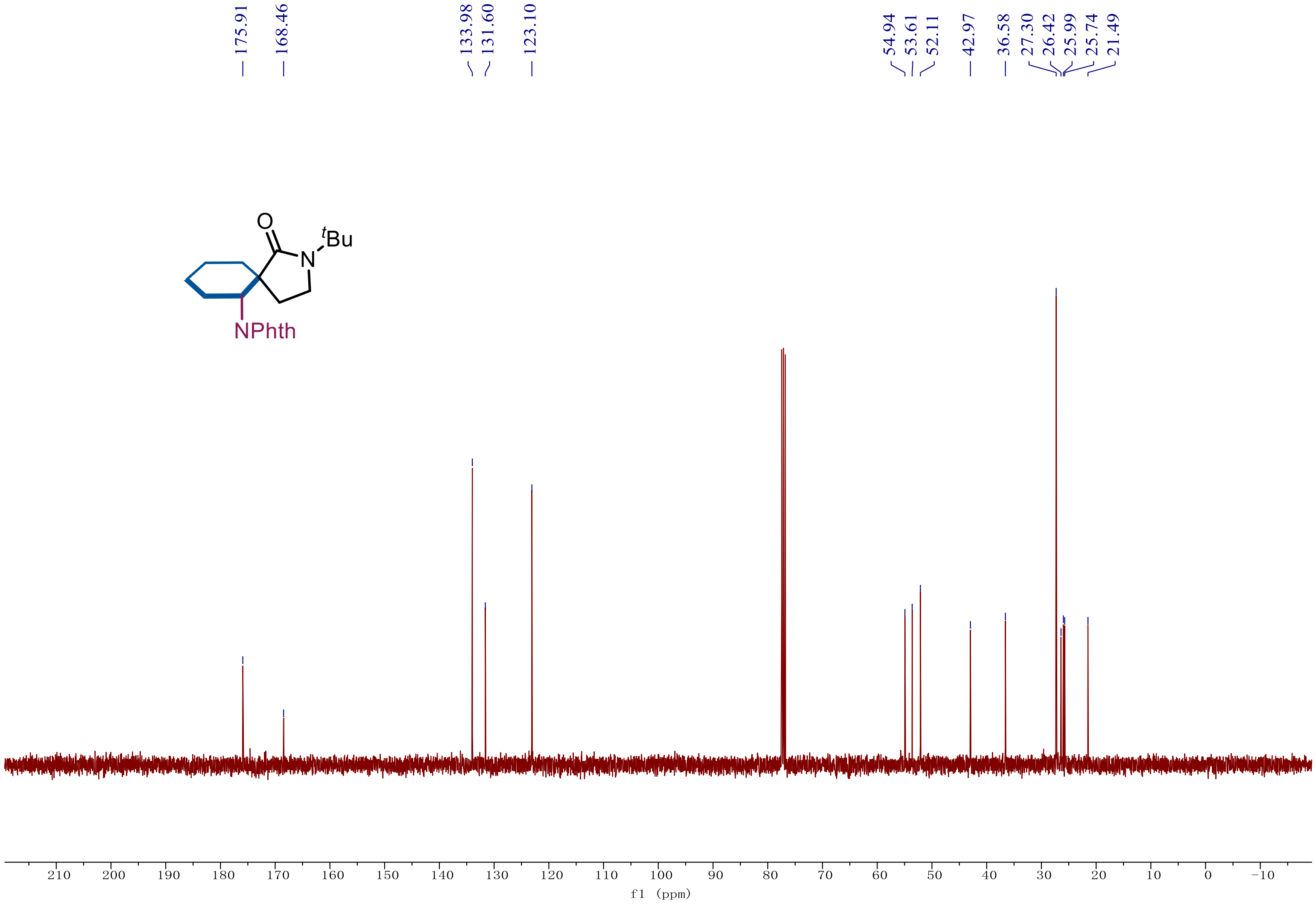

^{1}H NMR (400 MHz, $CDCl_3$) of **11**

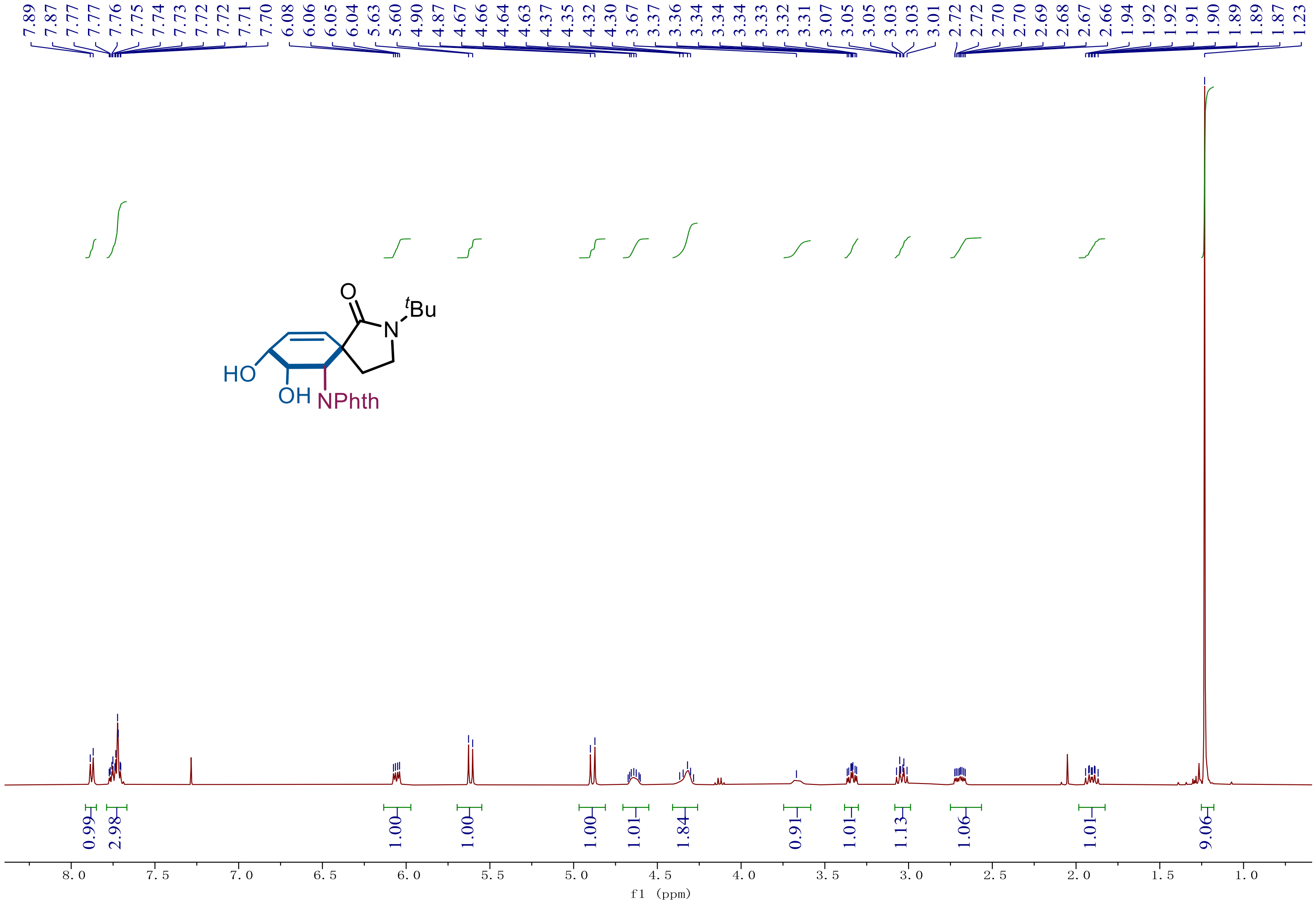

^{13}C NMR (101 MHz, $CDCl_3$) of **11**

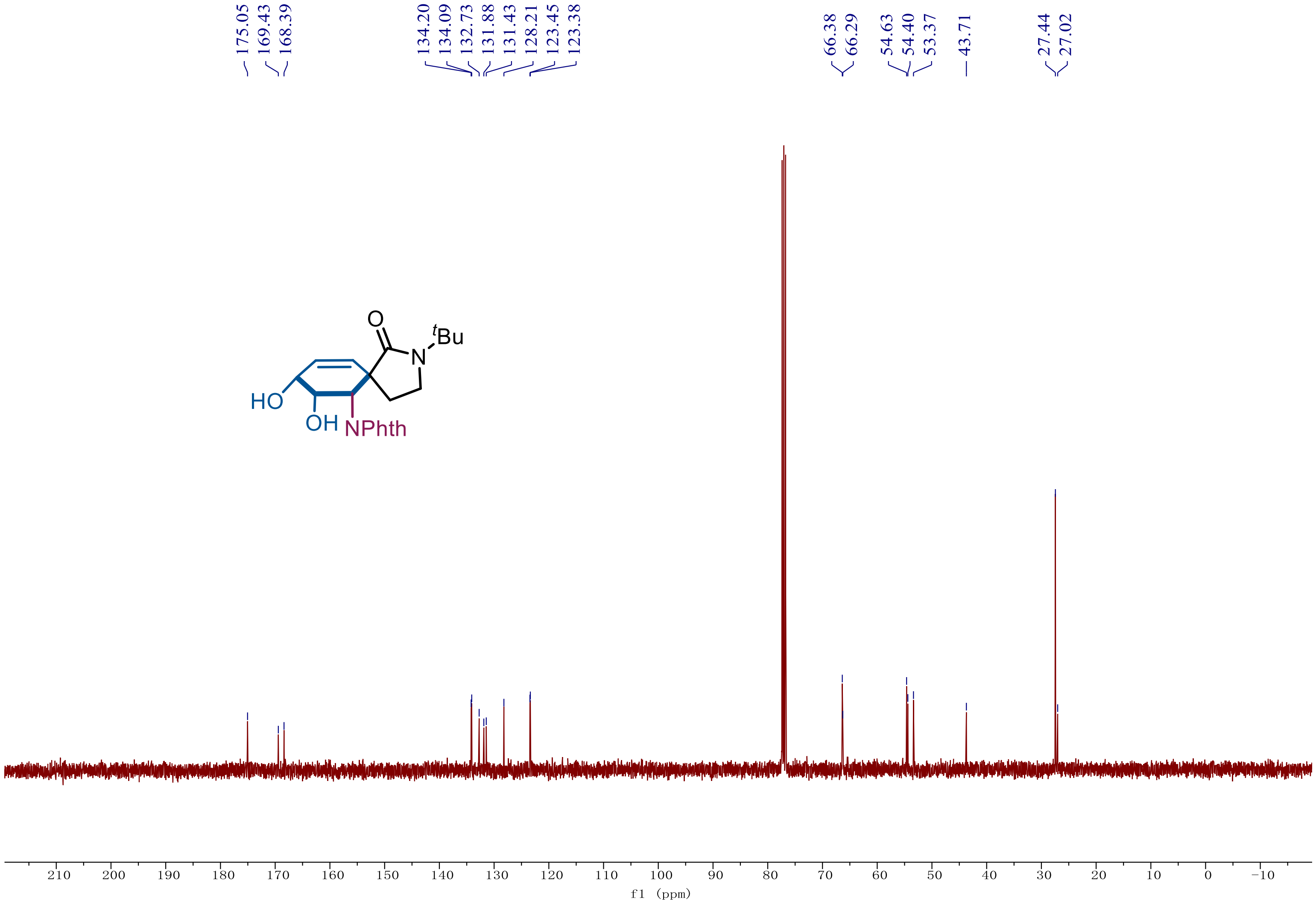

^1H NMR (400 MHz, $CDCl_3$) of **12**

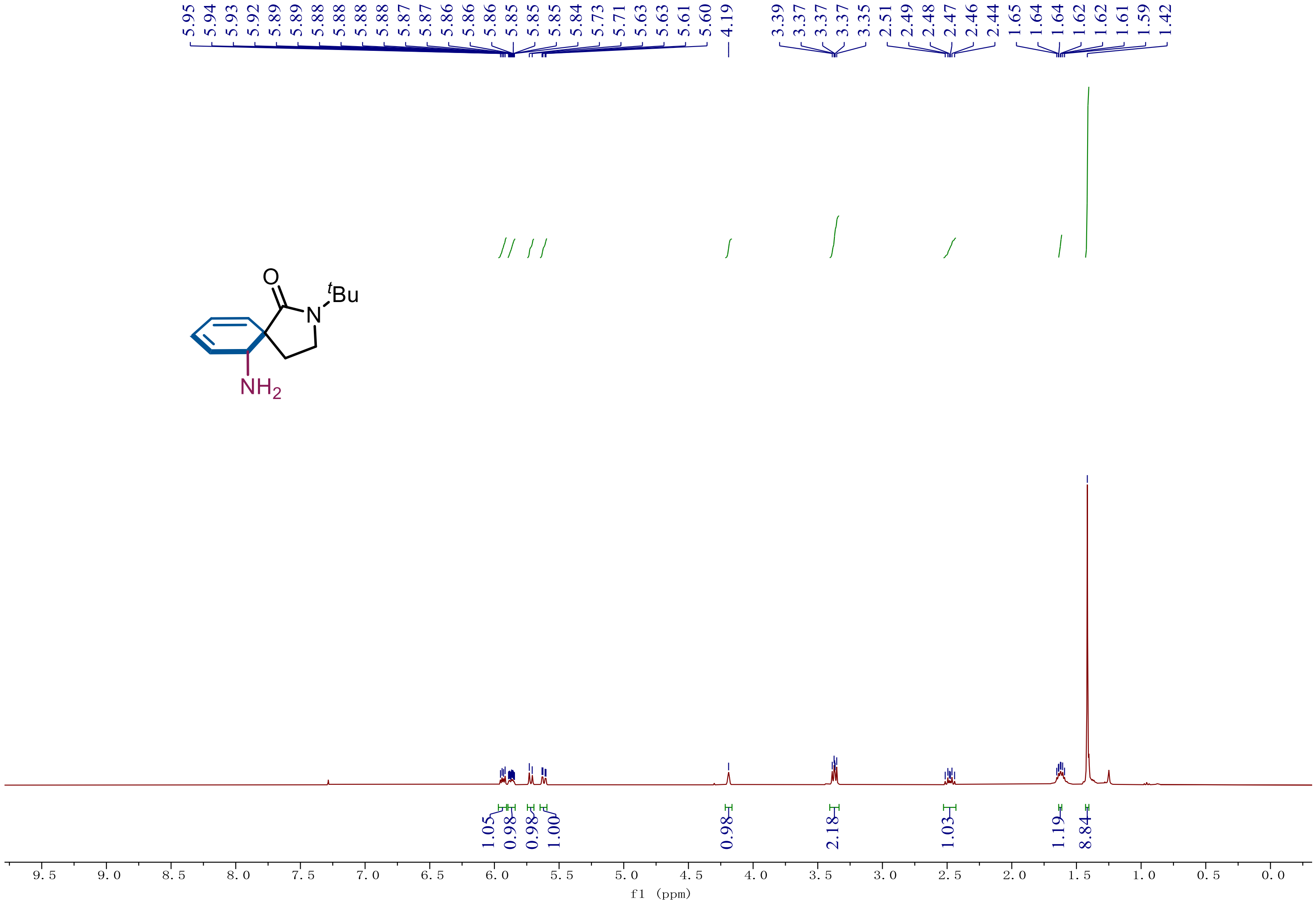

^{13}C NMR (101 MHz, CDCl$_3$) of **12**

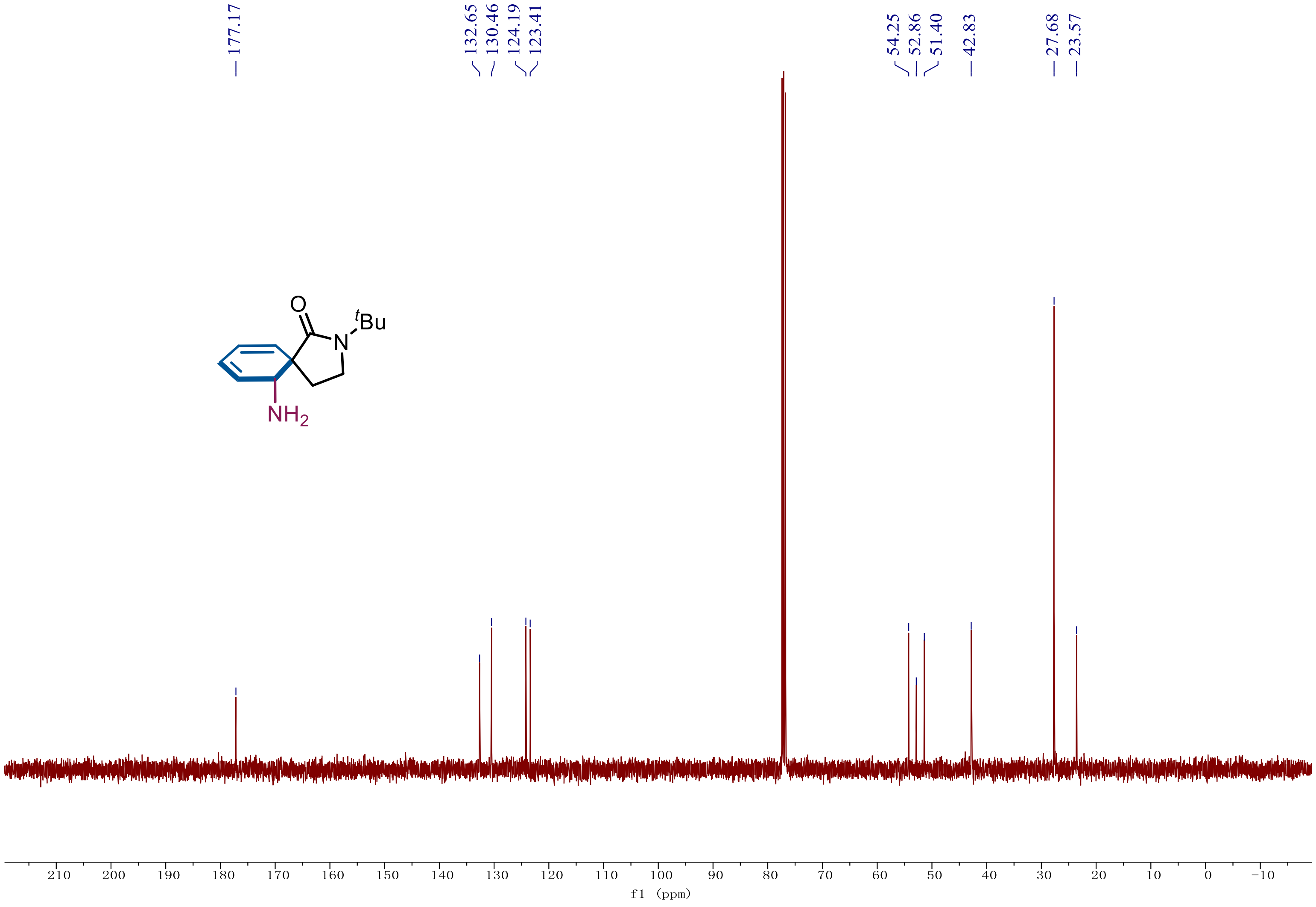

^{1}H NMR (400 MHz, CDCl$_3$) of **13**

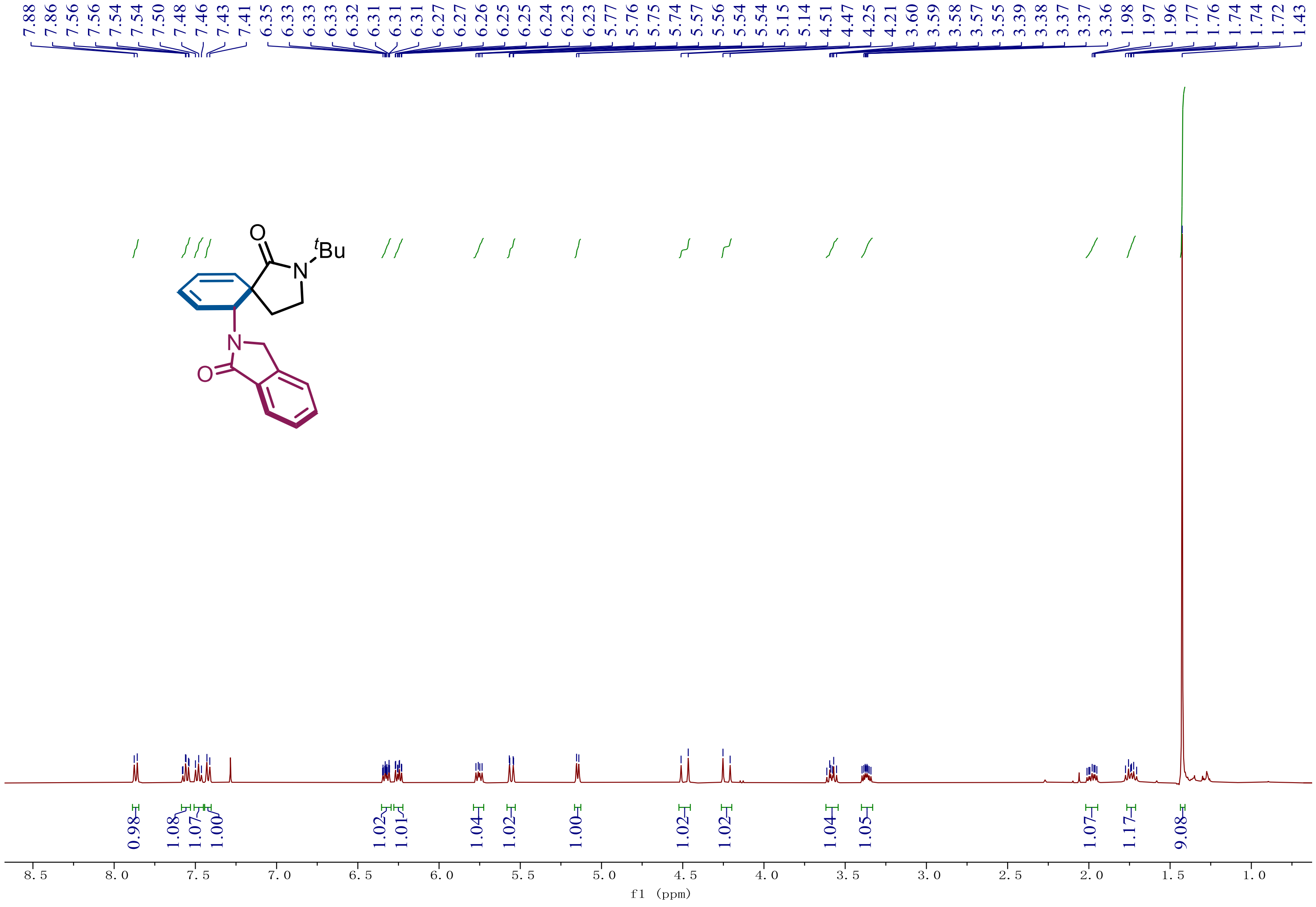

^{13}C NMR (101 MHz, $CDCl_3$) of **13**

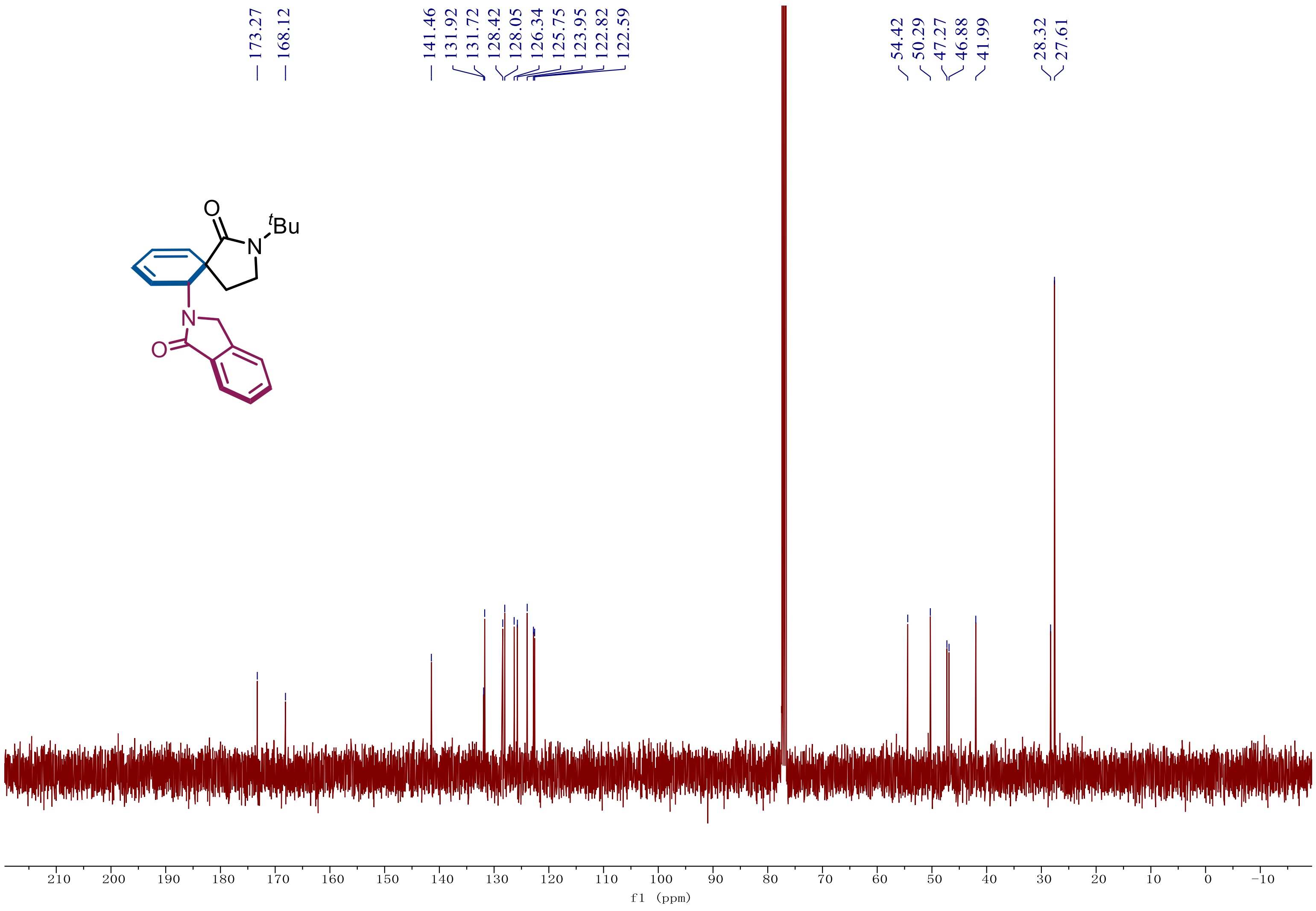

^{1}H NMR (400 MHz, $CDCl_3$) of **14-1**

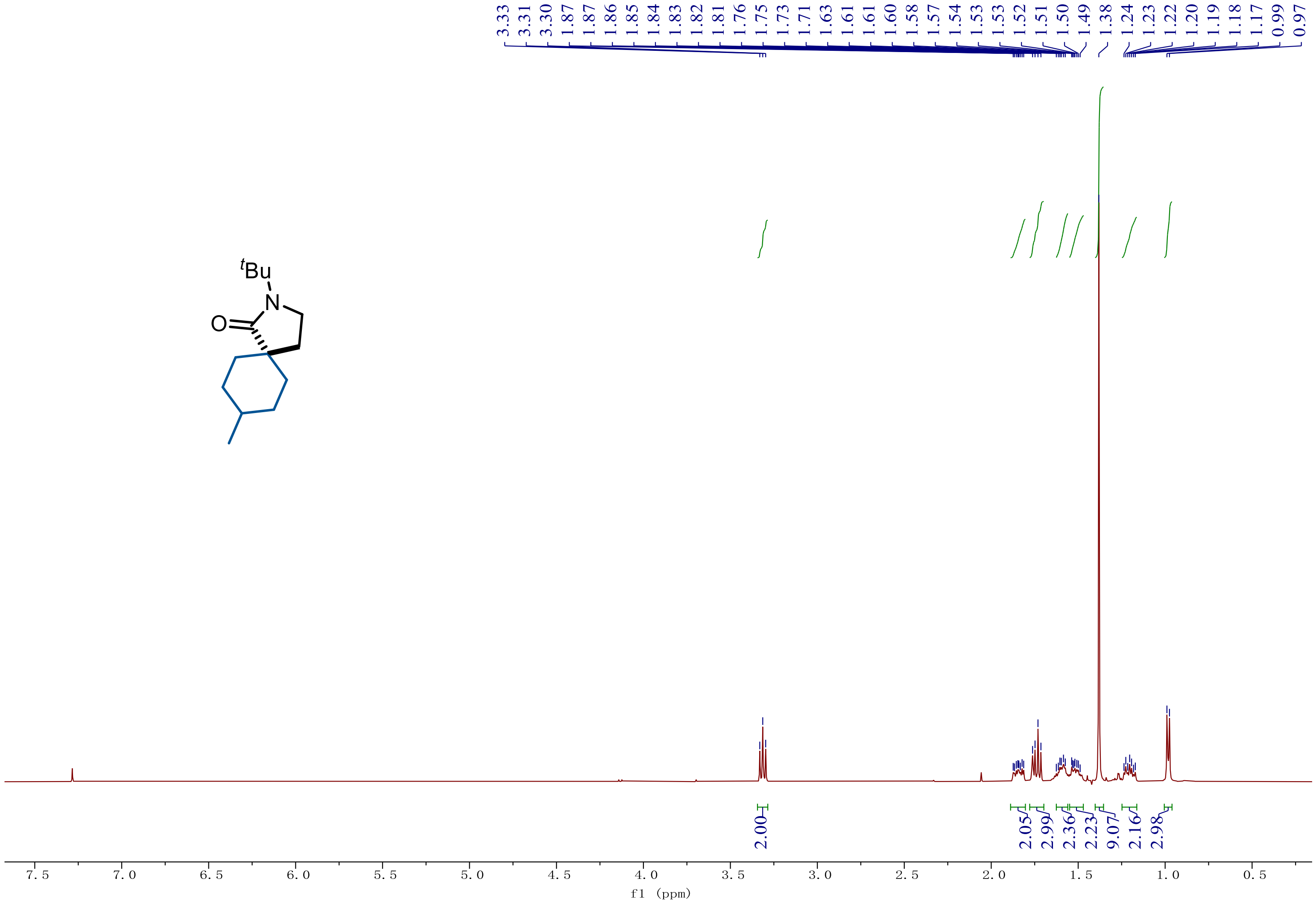

^{13}C NMR (101 MHz, CDCl$_3$) of **14-1**

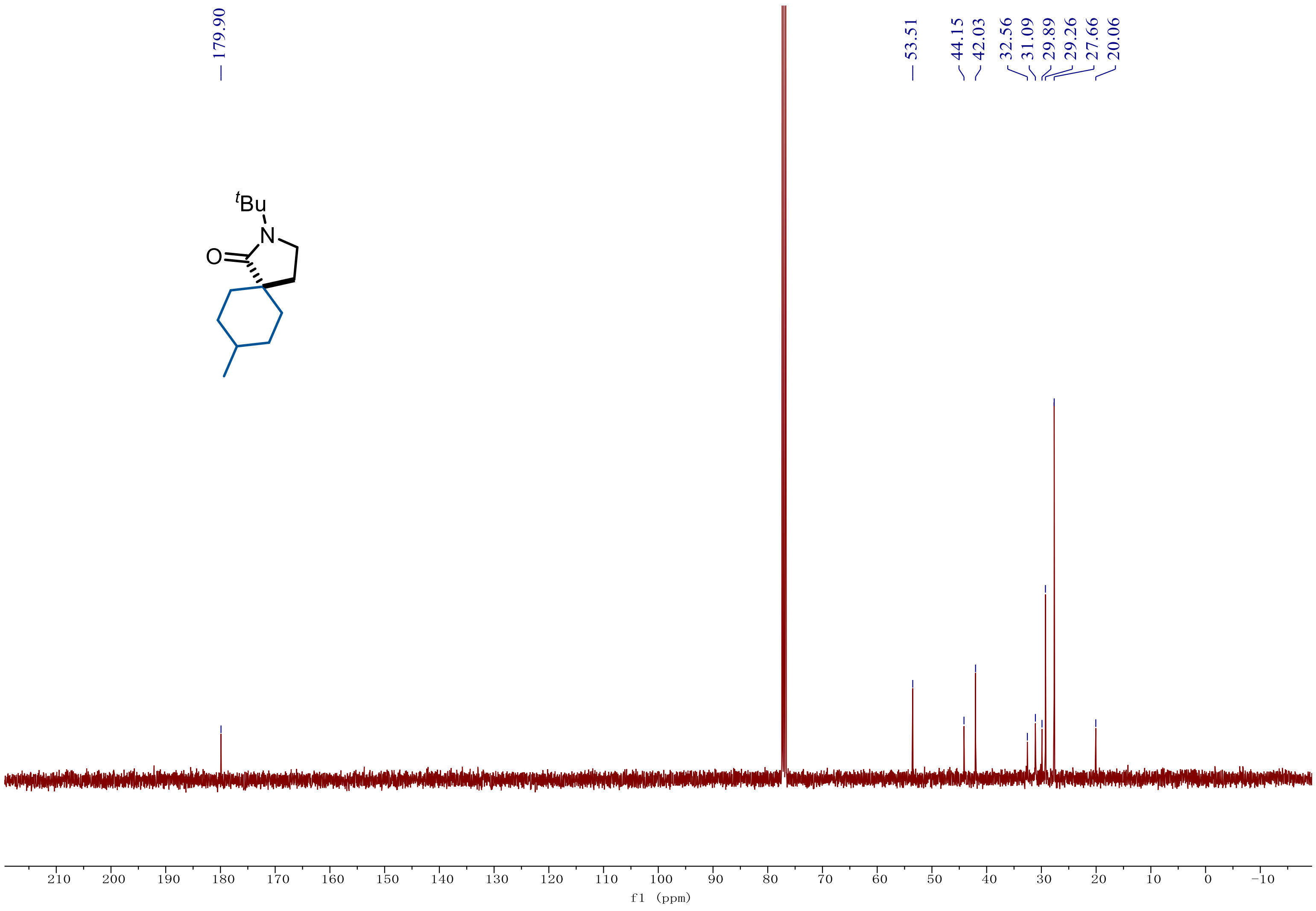

^1H NMR (400 MHz, $CDCl_3$) of **14-2**

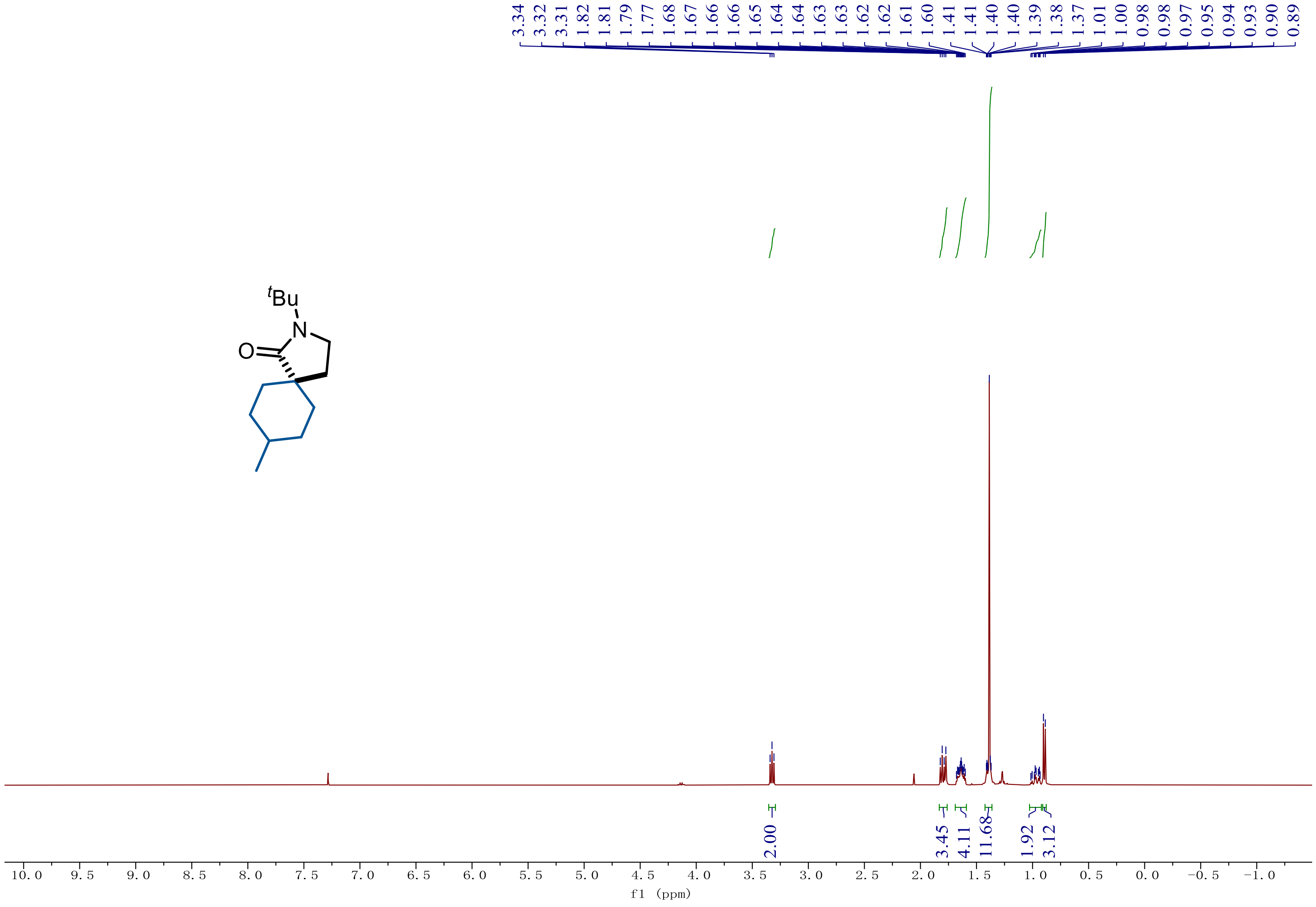

^{13}C NMR (101 MHz, $CDCl_3$) of **14-2**

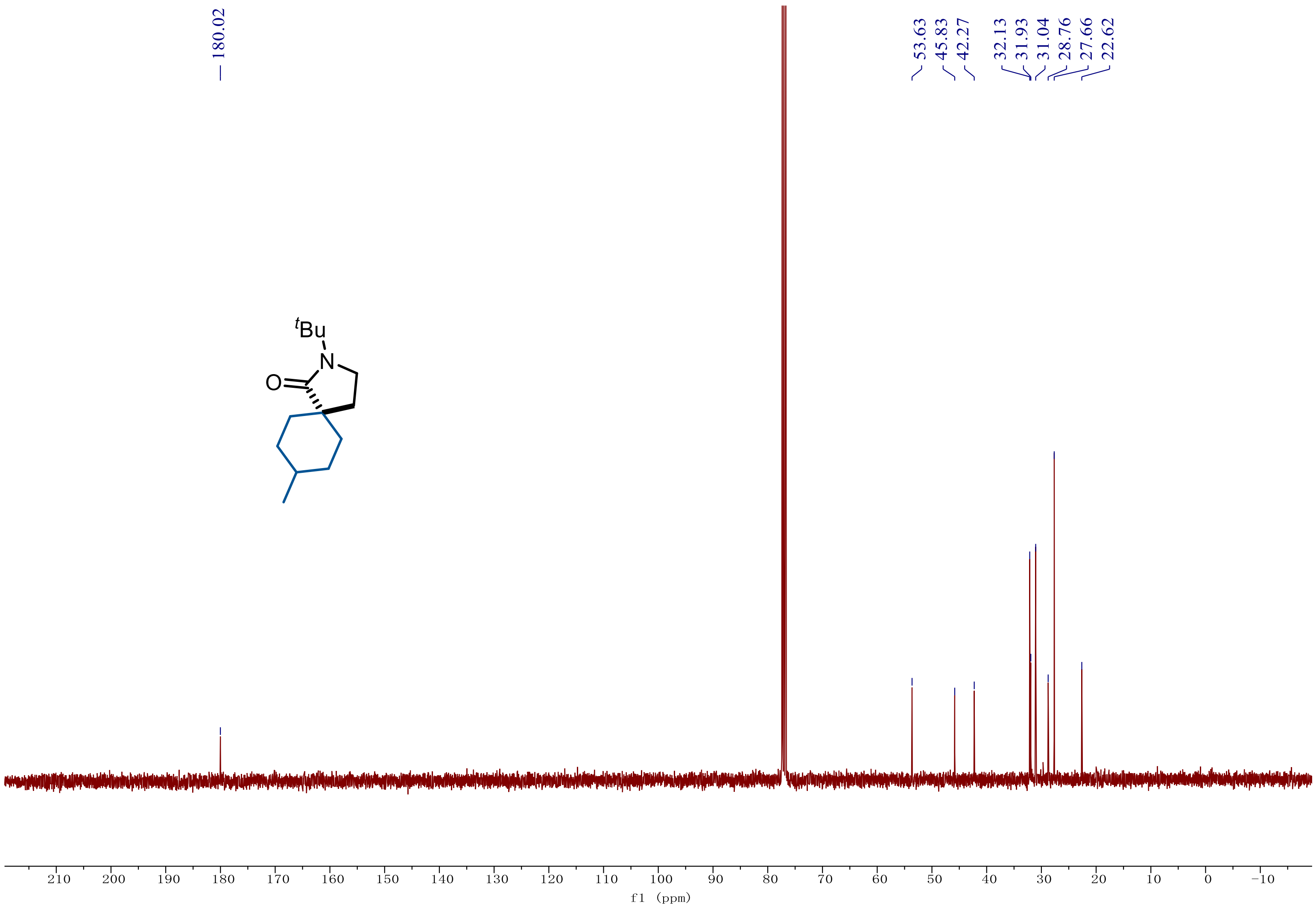

^{1}H NMR (500 MHz, $CDCl_3$) of **15**

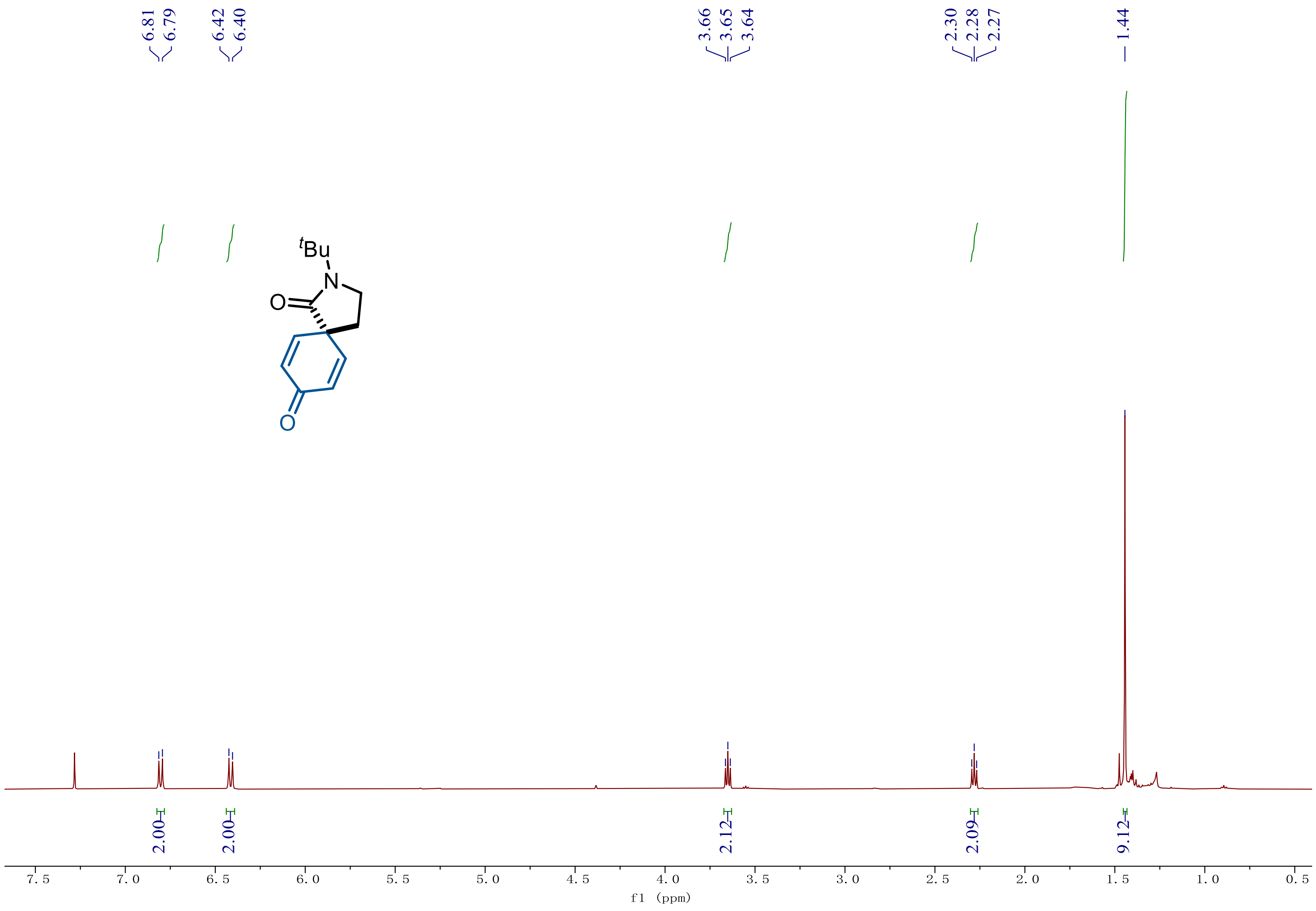

^{13}C NMR (126 MHz, $CDCl_3$) of **15**

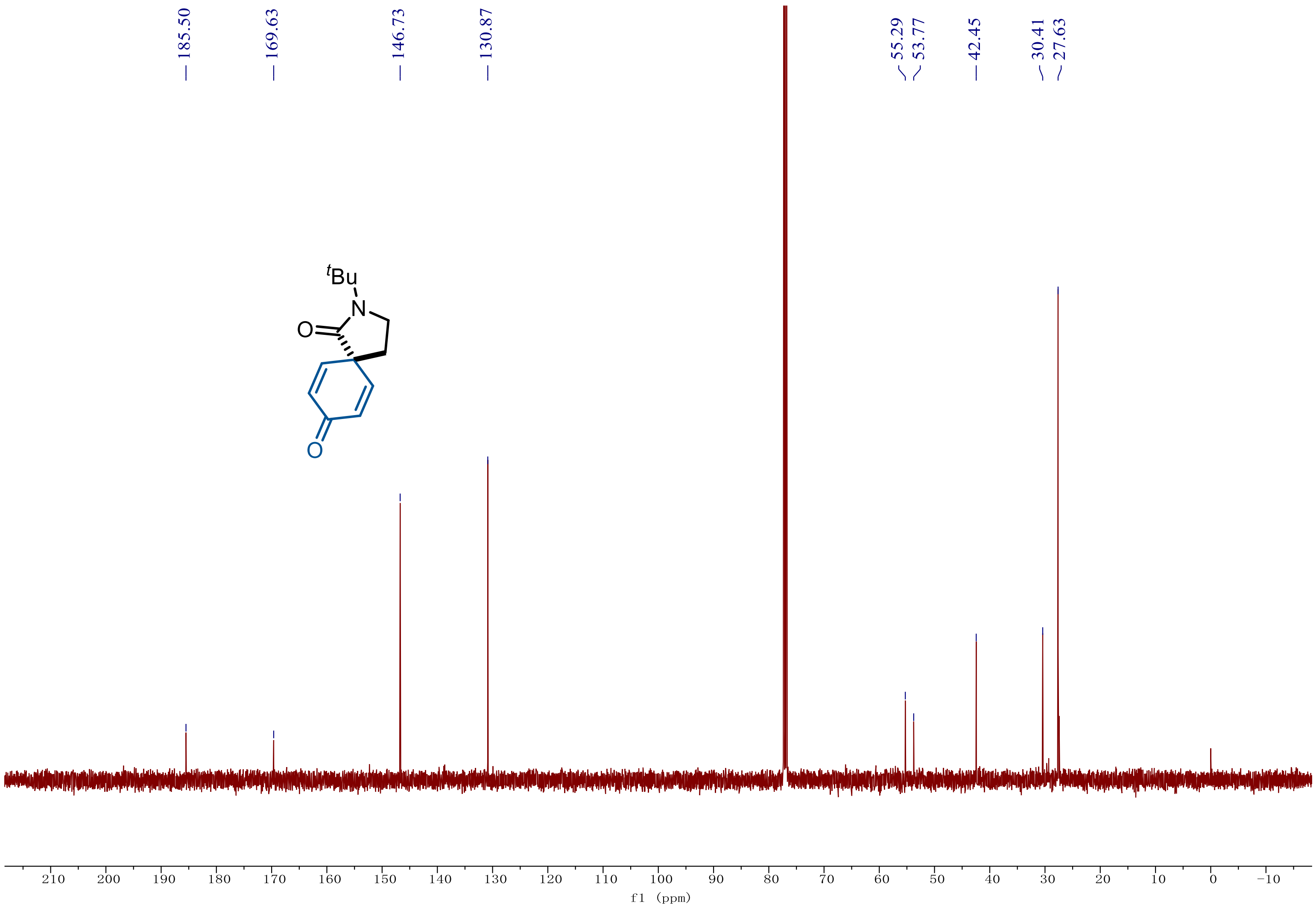

^{1}H NMR (500 MHz, $CDCl_3$) of **2ao**

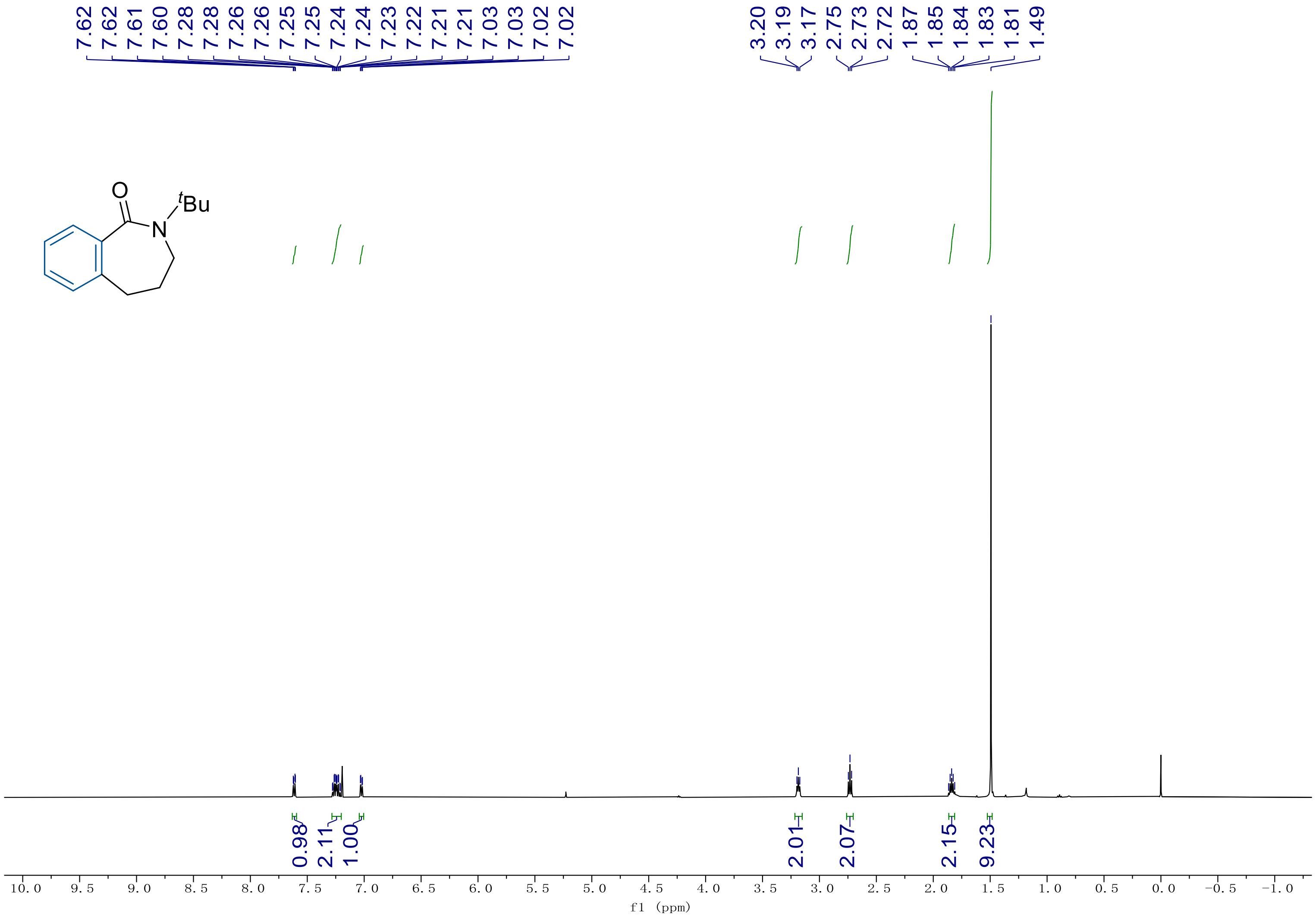

[13] C NMR (126 MHz, $CDCl_3$) of **2ao**

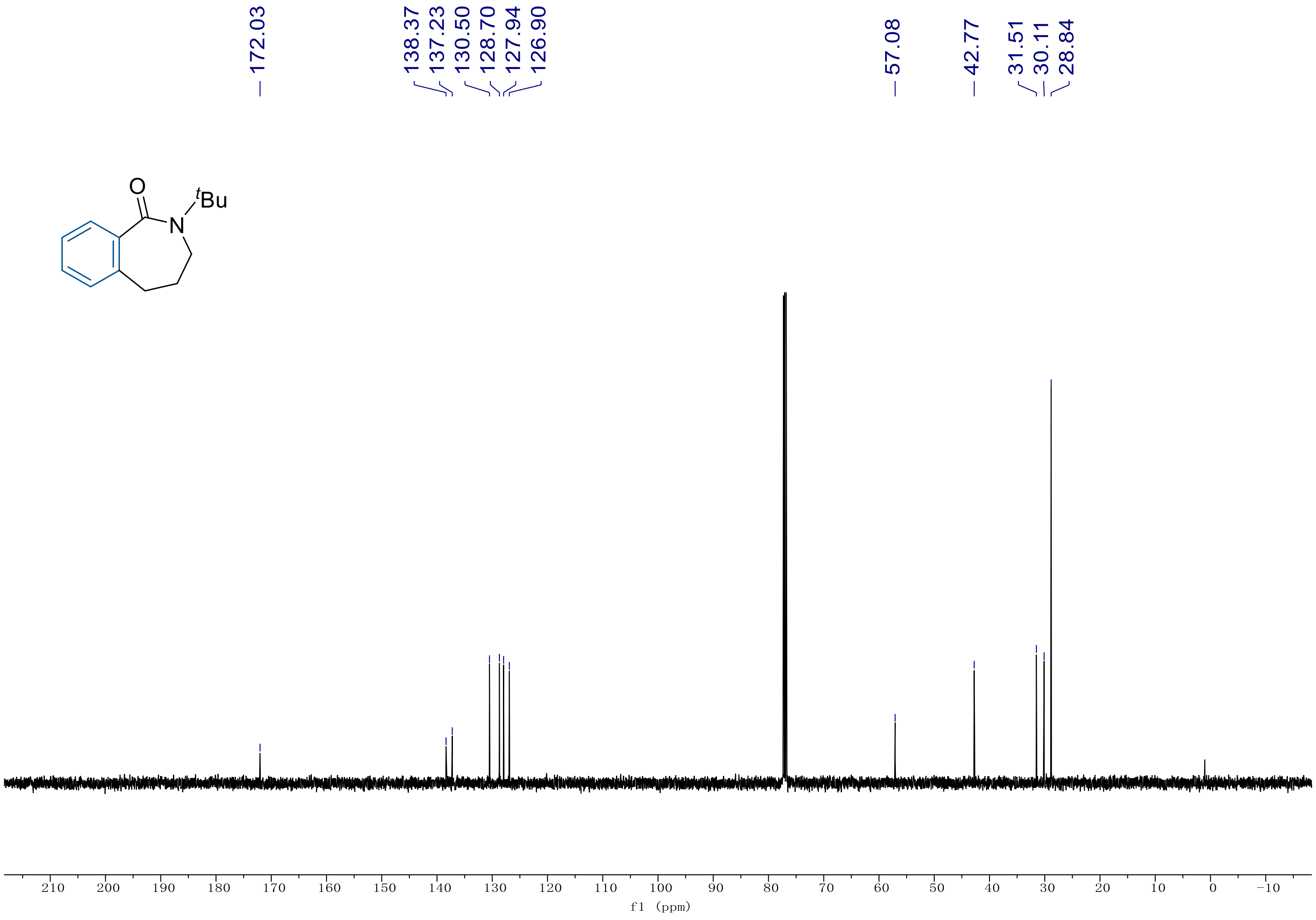

Supplement: Supplementary file 1 — Supporting Information [file ADVS-11-2307074-s001.pdf]
